# Photocatalysis Enables Chemodivergent Radical Polar Crossover: Ritter‐Type Amidation vs Heck‐Type Olefin Carbofunctionalizations

**DOI:** 10.1002/chem.202500666

**Published:** 2025-05-06

**Authors:** Mattia Lepori, Cassie Pratley, Indrasish Dey, Valeria Butera, Veronika Roider, Joshua P. Barham

**Affiliations:** ^1^ Fakultät für Chemie und Pharmazie Universität Regensburg Universitatsstraße 31 Regensburg 93053 Germany; ^2^ Department of Science and Biological Chemical and Pharmaceutical Technologies University of Palermo Palermo 90128 Italy; ^3^ Department of Pure and Applied Chemistry University of Strathclyde 295 Cathedral Street Glasgow G1 1XL UK

**Keywords:** chemodivergent, continuous flow, Heck reaction, photoredox catalysis, Ritter carboamidation

## Abstract

Three‐component alkene difunctionalization reactions constitute an ideal platform to rapidly build molecular complexity, enabling the simultaneous introduction of two distinct, orthogonal functional groups into the C═C bond in a single step. Herein, a photoredox catalyzed Ritter‐type carboamidation of electronically diverse styrenes harnessing non‐stabilized, nucleophilic primary radicals generated from readily‐accessible carboxylic acid‐derived redox active esters is reported. Furthermore, it is found that Heck‐type products are chemoselectively obtained by simply switching aryl olefin acceptors with 1,1‐diarylolefins. In the context of photocatalytic chemodivergent radical polar crossover, the synthesis of various trisubstituted alkenes was achieved, simultaneously revealing a divergence in the activation of redox‐active esters toward reduction. In‐depth mechanistic studies demonstrated both transformation pathways, while DFT calculations indicated the origin of product switchability. Both Ritter‐type and Heck‐type olefin carbofunctionalizations are scalable up to 4 mmol scale in batch and continuous flow, proving the synthetic utility of the methodology.

## Graphical abstract

One aryl makes the difference: switching styrenes with 1,1‐diaryl olefins as radical acceptors, the benzylic cation intermediate fate changes from Ritter‐type carboamidation to Heck‐type reaction. The decarboxylative photocatalytic chemodivergent methodology enabled broad scope and efficient scalability in both batch and flow. Mechanistic and computational studies demonstrated both radical generation pathways and the origin of product switchability.

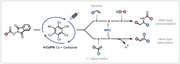

## 1 Introduction

Nitrogen‐containing organic compounds are prevalent in natural and pharmaceutical relevant motifs, with over 90% of drug molecules market incorporating nitrogen atoms.^[^ ^1^ ^]^ Given this significance, the development of strategies for C─N bond formation is of critical importance. Within this area, the Ritter reaction offers a straightforward approach for synthetic chemists, harnessing alkene‐ or alcohol‐derived carbocations and nitriles.^[^ ^2^, ^3^, ^4^ ^]^

**Figure 1:**
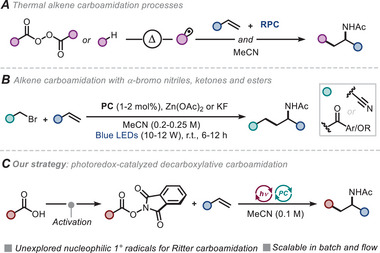
Synthetic strategies for olefin carboamidation.

Since its inception in 1948,^[^ ^5^, ^6^ ^]^ the Ritter reaction has undergone continuous development toward greener conditions and to broaden its precursor pool.^[^ ^7^, ^8^ ^]^ Notably, the advent of oxidative radical‐polar crossover (RPC),^[^ ^9^ ^]^ which sequentially exploits the reactivity of radicals and cations, has enabled the integration of the Ritter‐type process into atom and step‐economical multicomponent reactions (MCRs).^[^ ^10^ ^]^ In this regard, three‐component alkene difunctionalizations^[^ ^11^, ^12^ ^]^ represent a hot topic nowadays as *(i)* olefins are inexpensive and readily available organic feedstocks,^[^ ^13^ ^]^ and *(ii)* the introduction of two new, orthogonal functionalities into the C═C bond results in a rapid increase in molecular complexity, enhancing sp^3^ character and thereby contributing to the “Escape from Flatland” strategy in drug discovery.^[^ ^14^, ^15^ ^]^ In recent years, various alkene difunctionalization protocols have incorporated the Ritter‐type amidation as the final step to trap the carbocation, generated via Giese‐type addition and RPC of the radical intermediate, starting from both heteroatomic‐ (N,^[^ ^16^ ^]^ O,^[^ ^17^, ^18^ ^]^ P,^[^ ^19^ ^]^ and S)^[^ ^20^ ^]^ and carbon‐centered radical precursors. Regarding the latter, CF_3_ and aryl moieties insertions are well‐established using the Umemoto reagent^[^ ^21^ ^]^ or analogues^[^ ^22^, ^23^ ^]^ and diazonium or iodonium salts,^[^ ^24^, ^25^ ^]^ respectively. In the realm of alkene carboamidations, alkyl radicals can be generated via thermal processes (50–80 °C) from diacyl peroxide reactants in the presence of an iron catalyst or by activating the C(sp^3^)─H bond of alkane reactants using benzoyl peroxide and radical initiators (Figure 1A).^[^ ^26^, ^27^, ^28^, ^29^ ^]^ Building on these findings, the groups of Chen and Maity reported photoredox‐catalyzed carboamidation protocols using *α*‐bromo nitriles, ketones and esters as radical precursors (Figure 1B).^[^ ^30^, ^31^ ^]^ Noteworthy in these reports are the use of cost‐effective starting materials along with additives (KF or Zn(OAc)_2_) that suppress alkylbromination by‐products from atom transfer radical addition (ATRA).^[^ ^32^ ^]^ However, these conditions depend on electrophilic *α*‐cyano and keto/ester primary radicals, thus limiting their generality. In light of this, a photocatalytic complementary strategy that can harness non‐stabilized nucleophilic radicals^[^ ^33^ ^]^ is highly desirable.

With this target in mind and inspired by a recent net‐oxidative photoelectrochemical Ritter‐type protocol disclosed by our group,^[^ ^34^ ^]^ a redox‐neutral photocatalytic single electron transfer (SET) approach was envisioned starting from readily‐accessible carboxylic acids activated as *N*‐hydroxyphthalimide (NHPI)‐based redox‐active esters (RAEs),^[^ ^35^, ^36^, ^37^ ^]^ offering an intuitive retrosynthetic strategy.

In fact, due to their stability and ease of preparation, NHPI esters constitute the dominant choice as alkylating agents in decarboxylative three‐component styrene difunctionalizations, as demonstrated in examples with oxygen‐ (water, alcohols),^[^ ^38^, ^39^, ^40^, ^41^ ^]^ nitrogen‐ (amides, amines),^[^ ^39^, ^42^ ^]^ carbon‐ (cyano, indoles)^[^ ^43^, ^44^ ^]^ or halogen‐ (Cl, F) based nucleophiles.^[^ ^39^, ^45^ ^]^ However, a crucial remaining challenge in such MCRs lies in the use of primary radicals, where previous protocols generally focused on secondary and tertiary precursors. Herein, we report the achievement of highly reactive primary alkyl radicals for tandem RPC Ritter‐type process by leveraging an organophotocatalyst under visible light irradiation (Figure 1C). Furthermore, during the realization of this protocol, it was found that Heck‐type products were chemoselectively obtained by simply switching aryl olefin Giese‐type acceptors with 1,1‐diarylolefins (*cf*. Table 2). Thus, a transition metal‐ and additive‐free alkenylation of carbon‐centered radicals was optimized and applied to synthesize various trisubstituted alkenes. The proposed acid‐free Heck‐type conditions also highlighted a divergence in the activation mechanism of RAEs towards the initial reductive SET event in the two proposed (Ritter‐type/Heck‐type) transformations (*i.e*., Brønsted acid LUMO lowering vs *π*─*π* stacking complexation, *cf*. Figure 5B). Our unifying chemodivergent strategy was applied to late‐stage functionalizations (LSF) of relevant scaffolds and was efficiently scalable in both batch and flow (up to a multi‐mmol scale).

## 2 Results and Discussion

Our experimental investigation commenced with the optimization of the photoredox‐catalyzed styrene carboamidation reaction. After extensive screening of all relevant reaction parameters (for further information, see Section S5.1, Supporting Information), it was found that the difunctionalized product **4** could be obtained in 50% yield when an acetonitrile solution (0.1 M) of the 3‐phenylpropanoic acid NHPI derivative **1a** (1.0 equiv.) as a primary radical precursor, 4‐trifluoromethyl styrene **2a** (1.2 equiv.) as a radical acceptor, 1,2,3,5‐tetrakis (carbazol‐9‐yl)‐4,6‐dicyanobenzene (**4‐CzIPN**, 5 mol%) as a photocatalyst and trifluoroacetic acid (TFA, 10 equiv.) was irradiated with blue LEDs (40 W, 427 nm Kessil lamp) for 16 h (Table 1, Entry 1). Remarkably, the more expensive organophotocatalyst **4‐DPAIPN** or transition metal‐based photocatalyst Ir(ppy)_3_ gave inferior yields (Entries 2–3). Altering the equivalents of TFA did not affect the reaction efficiency, whereas its omission resulted in complete suppression of the process (Entry 4), consistent with the previously proposed role of the Brønsted acid in activating **1a** toward SET reduction^[^ ^38^, ^46^, ^47^, ^48^ ^]^ and its involvement in the Ritter‐type step (*cf*. Figure 5B).^[^ ^49^, ^50^ ^]^ Various other Brønsted acid additives were screened, all giving either lower yields or complex crude mixtures (see Table S2, Supporting Information). To explore the possibility to extend our methodology to acid‐sensitive substrates, different additives were tested (see Table S8, Supporting Information), revealing zinc trifluoroacetate hydrate Zn(CF_3_COO)_2_·xH_2_O (50 mol%) as a cost‐effective and suitable Lewis acid^[^ ^42^ ^]^ for the transformation (Entry 5). Reversing the stoichiometry of **1a** and **2a** did not affect the yield but increased the formation of hydroalkylation by‐product **S1a** (31% in Table 1, Entry 6). This strongly implicated that a hydrogen atom transfer (HAT) process was responsible for forming **S1a**,^[^ ^51^, ^52^ ^]^ where the incipient benzyl radical after Giese‐type addition (**II** in Figure 5B) receives a hydrogen atom from one of the activated CH_2_ groups in **1a**. Finally, no desired product was observed in absence of the photocatalyst (Entry 7).

**Table 1:** Optimization of Ritter‐type carboamidation conditions.

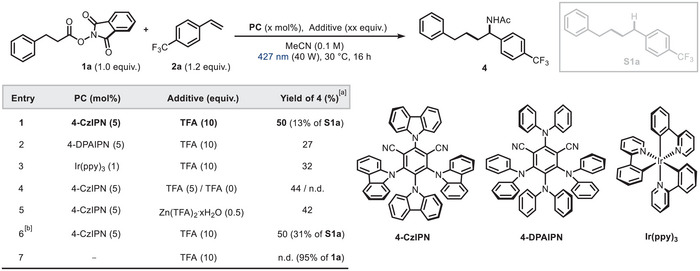

^[a]^ Yields were determined by ^1^H NMR using 1,1,2,2‐tetrachloroethane as an internal standard. Reactions performed on a 0.1 mmol scale in anhydrous MeCN.

^[b]^ **1a** (1.5 equiv.) and **2a** (1.0 equiv.) in MeCN (0.1 M). n.d. = not detected.

**Figure 2:**
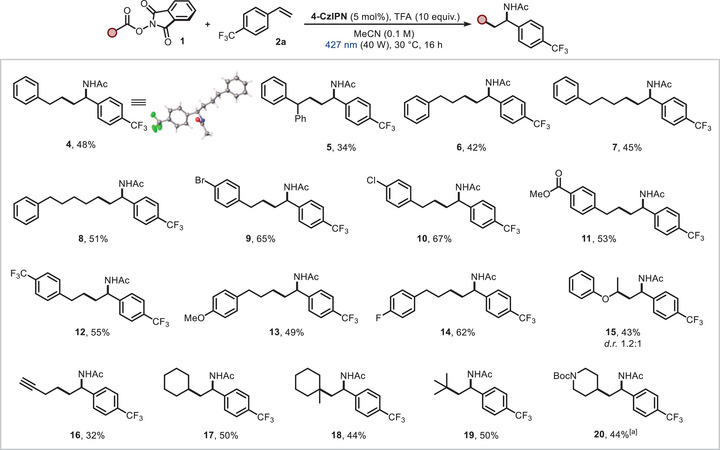
Scope of the Ritter‐type carboamidation of 4‐trifluoromethyl styrene (**2a**) using redox active esters. Reaction conditions: redox active ester **1** (0.3 mmol, 1.0 equiv.), 4‐trifluoromethyl styrene **2a** (0.36 mmol, 1.2 equiv.), **4‐CzIPN** (5 mol%), TFA (10 equiv.) in anhydrous MeCN (0.1 M). For further details, see Supporting Information. [a] Zn(CF_3_CO_2_)_2_·xH_2_O (50 mol%) used instead of TFA as an additive. Isolated yields are reported.

**Figure 3:**
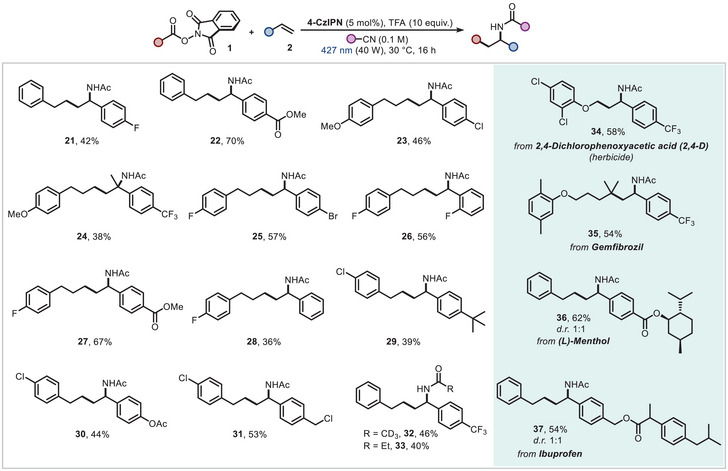
Scope of the Ritter‐type olefin carboamidation using redox active esters. Reaction conditions: redox active ester **1** (0.3 mmol, 1.0 equiv.), aryl olefin (0.36 mmol, 1.2 equiv.), **4‐CzIPN** (5 mol%), TFA (10 equiv.) in the corresponding nitrile (0.1 M) as reaction solvent. For further details, see Supporting Information. Isolated yields are reported.

Having established the optimal reaction conditions, we next investigated the scope of the three‐component carboamidation of **2a** with a variety of RAEs derived from readily available carboxylic acids (Figure 2). Initial efforts focused on modifications of the phenyl‐bearing alkyl chain in **1a**. Given the significance of alkyl chain length in influencing critical properties for agro‐ and biochemical applications,^[^ ^53^ ^]^ phenyl propanoic, butanoic, pentanoic, and hexanoic acid derivatives were successfully incorporated into the C═C double bonds, resulting in synthetically useful yields (products **4**, **6–8**), with the structure of **4** confirmed by single crystal XRD analysis. The relative underperformance of the 3,3‐diphenylpropanoic acid derivative to give product **5** is likely due to the reactive diaryl benzylic C(sp^3^)─H bond (BDE ≃ 84 kcal mol^−1^ for diphenylmethane),^[^ ^51^ ^]^ which may facilitate competitive side reactions or degradation pathways. Subsequently, various RAEs bearing functional groups at the *para*‐position of the arene moiety were subjected to the reaction conditions. Both electron‐withdrawing (Br, Cl, carboxylic ester, trifluoromethyl) and electron‐donating groups (methoxy) were well tolerated, affording the functionalized amide products in good yields (**9–14**). The protocol also efficiently engaged *α*‐oxy radicals and substrates with an alkynyl moiety (**15** and **16**). Importantly, examination of both secondary and tertiary radical precursors revealed the generality of our method, leading to corresponding difunctionalized products in satisfactory yields (**17–19**). Notably, although the conditions with TFA resulted in no detectable product when using the Boc‐protected piperidine‐4‐carboxylic acid derivative, the simple switch to the Zn additive (Table 1, Entry 5) tolerated the sensitive substrate, affording the product in a useful yield (**20**). Next, we aimed to further apply our conditions to different aromatic olefins (Figure 3). Several electron‐poor styrenes were successfully difunctionalized in the three‐component process, including both para‐ (F, Cl, Br, carboxylic methyl ester) and *ortho*‐ (F) substituted acceptors in moderate to good yields (**21–23**, **25–27**). To our delight, *α*‐methyl 4‐trifluorostyrene, styrene and *4‐tert‐butyl* styrene were engaged as SOMOphiles, presenting the amide products in moderate yields (**24**, **28** and **29**). 4‐Acetoxystyrene and 1‐(chloromethyl)‐4‐vinylbenzene yielded the desired products chemoselectively, without observing ester hydrolysis or dechlorination of the benzylic chloride moiety (**30–31**). As for the nitrile variation, a limitation of the scope was observed: in fact, only deuterated acetonitrile and propionitrile were found to be suitable reactants for altering the amidic portion (**32** and **33**). Other nitriles failed due to their inability to solubilize RAE substrates, even after irradiation with blue LEDs for 16 h. Intriguingly, the developed photocatalytic conditions were found to be applicable to agrochemically‐ and pharmaceutically‐relevant motifs. For example, 2,4‐dichlorophenoxy acetic acid and Gemfibrozil were converted into their NHPI derivatives and used to carboamidate **2a**. On the other hand, (*L*)‐Menthol and Ibuprofen containing olefins successfully reacted with RAE **1a**. Thus, a desired synergy of LSF with Ritter‐type processes was achieved in good yields (**34–37**).^[^ ^54^, ^55^ ^]^

While exploring the scope of aryl olefins, the reaction between **1a** and 1,1‐diphenylethylene (DPE, **3a**) gave no trace of the target carboamidated product in the crude reaction mixture. Instead, Heck‐type^[^ ^56^ ^]^ product **38** was observed in 55% yield (Table 2, Entry 1). This serendipitous outcome was unexpected, especially given that prior studies in the literature achieved photoredox‐catalyzed difunctionalization of DPE with nucleophiles such as water,^[^ ^38^, ^57^ ^]^ alcohols,^[^ ^39^ ^]^ fluoride,^[^ ^45^ ^]^ sulfoximines,^[^ ^58^ ^]^ and benzoates.^[^ ^59^ ^]^ Presumably, in these protocols the nucleophiles efficiently engage the incipient benzylic carbocation—generated via tandem radical addition and oxidative RPC steps—before deprotonation at the *β*‐position occurs.

**Table 2:** Optimization of Heck‐type reaction conditions.

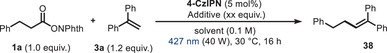			

Entry	Solvent (0.1 M)	Additive (equiv.)	Yield of **38** (%)^[^ ^a^ ^]^
1	Dry MeCN	TFA (10)	55
2	Dry MeCN	‐	38
3	Dry DMF	‐	52
4	DMF^[^ ^b^ ^]^	‐	**62**
5^[^ ^c^ ^]^	DMF	‐	59

^[a]^ Yields were determined by ^1^H NMR using 1,1,2,2‐tetrachloroethane as an internal standard. 0.1 mmol scale.

^[b]^ Analytical grade DMF (p.a.) was used as received from the supplier.

^[c]^ **1a** (1.2 equiv.) and **3a** (1.0 equiv.) in DMF (0.1 M). p.a. = per analysis.

This result underscored a limitation for the initially targeted Ritter‐type difunctionalization. However, in our view it presented an intriguing example of photocatalytic chemodivergence.^[^ ^60^, ^61^ ^]^

We became motivated to turn DPE's inability to undergo the three‐component transformation (consistent with preliminary observations reported by the groups of Akita^[^ ^23^ ^]^ and Song, Li)^[^ ^40^, ^42^ ^]^ into an advantage. Thus, we sought to optimize the Heck‐type reaction of 1,1‐diarylolefins (for further information, see Section S5.2, Supporting Information). To our surprise, the reaction proceeded in the absence of TFA, albeit in a lower yield (Table 2, Entry 2), indicating a divergence in the activation mechanism of RAEs (*cf*. Figure 5B). During the solvent screening, it was initially observed that anhydrous DMF without additives afforded **38** with comparable efficiency to that achieved under acidic conditions in acetonitrile (Entry 3). Intriguingly, the reaction demonstrated higher yields when using analytical grade DMF as received by the supplier, thereby obviating the tedious need to dry and distill the reaction solvent (Entry 4). Reversing the stoichiometry of **1a** and **3a** gave a similar, albeit marginally lower yield (Entry 5 vs Entry 4). Such conditions give flexibility to avoid unreacted excess DPE which can pose challenges in purification of certain substrates due to co‐elution with target products (e.g., radical precursors without aryl chromophores).

**Figure 4:**
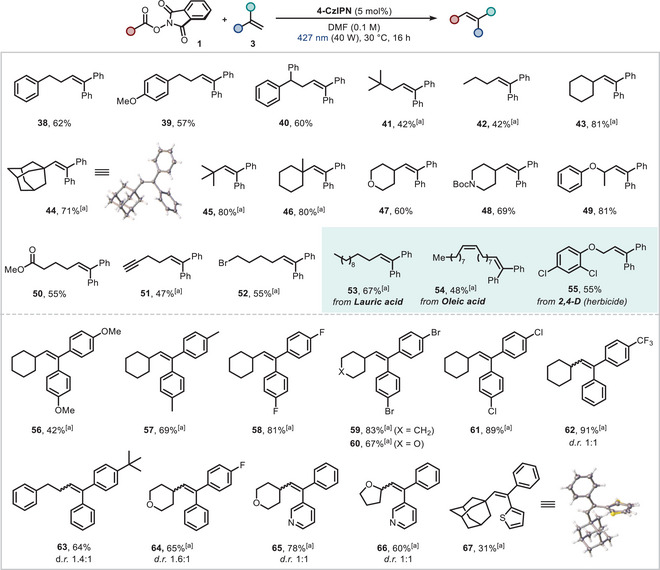
Scope of the Heck‐type reaction of 1,1‐diarylolefin using redox active esters. Reaction conditions: redox active ester **1** (0.mmol, 1.0 equiv.), 1,1‐diarylolefin **3** (0.36 mmol, 1.2 equiv.), **4‐CzIPN** (5 mol%) in DMF (p.a., 0.1 M). For further details see Supporting Information. [a] Redox active ester (0.36 mmol, 1.2 equiv.), 1,1‐diarylolefin (0.3 mmol, 1.0 equiv.) and **4‐CzIPN** (5 mol%) in DMF (p.a., 0.1 M). Isolated yields are reported. p.a. = per analysis.

With optimized conditions in hand (Entries 4 and 5), we set out to evaluate the generality of our method, which differs from the literature strategies to access similar Heck‐type products as it requires neither transition metal‐based photocatalysts,^[^ ^62^, ^63^, ^64^, ^65^, ^66^, ^67^, ^68^, ^69^, ^70^ ^]^ nor additives such as exogenous acids or bases.^[^ ^71^, ^72^, ^73^ ^]^ We began by applying the protocol to different RAEs (Figure 4). Firstly, slight modifications to the standard NHPI ester **2a** were explored, resulting in good yields of products (**39** and **40**) when employing *para*‐methoxy and gem‐diphenyl analogues.

Next, various alkyl radical precursors, including primary, secondary and tertiary radicals, were efficiently engaged in the alkenylation process, resulting in moderate to very good product yields (**41**–**46**). The structure of adamantane derivative **44** was confirmed via single crystal XRD. *γ*‐Radicals derived from tetrahydropyran and Boc‐protected piperidine, along with *α*‐oxy radicals, were successfully engaged under our mild reaction conditions (products **47**–**49**). The optimized protocol tolerated esters, alkynes and alkyl bromides (**50**–**52**), with no “back” radical cyclization of the incipient benzylic radical observed. Notably, bioactive molecules such as lauric acid and oleic acid, as well as the common herbicide 2,4‐dichlorophenoxy acetic acid, were successfully employed as reaction partners with **3a**, furnishing the corresponding functionalized products (**53**–**55**) in synthetically useful yields. We subsequently examined the scope of freshly prepared 1,1‐diarylolefins and found that a diverse set of *para*‐substituted symmetric examples were viable acceptors for the cyclohexyl radical. A trend became apparent between efficiency and electronics of the 1,1‐diarylolefins. Strongly and moderately electron donating groups (methoxy and methyl, respectively) gave moderate and good product yields (**56** and **57**), while high product yields resulted from electron withdrawing substituent (F, Br, Cl) bearing SOMOphiles (**58**, **59** and **61**). This is likely due to enhanced polarity matching in the Giese‐type radical addition step involving the nucleophilic radical.^[^ ^74^, ^75^ ^]^ 4,4′‐(Ethene‐1,1‐diyl)bis(bromobenzene) could also be coupled with *γ*‐tetrahydropyranyl radical, albeit in lower product yield (**60**). Asymmetric 1,1‐diarylolefins were successfully functionalized with both primary and secondary radicals (**62**–**64**). Finally, substrates containing heteroaromatic rings were well‐tolerated under the proposed conditions, enabling access to pyridine containing scaffolds not reported in the aforementioned photocatalytic methods (**65** and **66**). Notably, the thiophene‐bearing structure **67** was isolated as a single diastereoisomer, whose (*Z*)‐configuration was unambiguously confirmed by single‐crystal XRD analysis.

**Figure 5:**
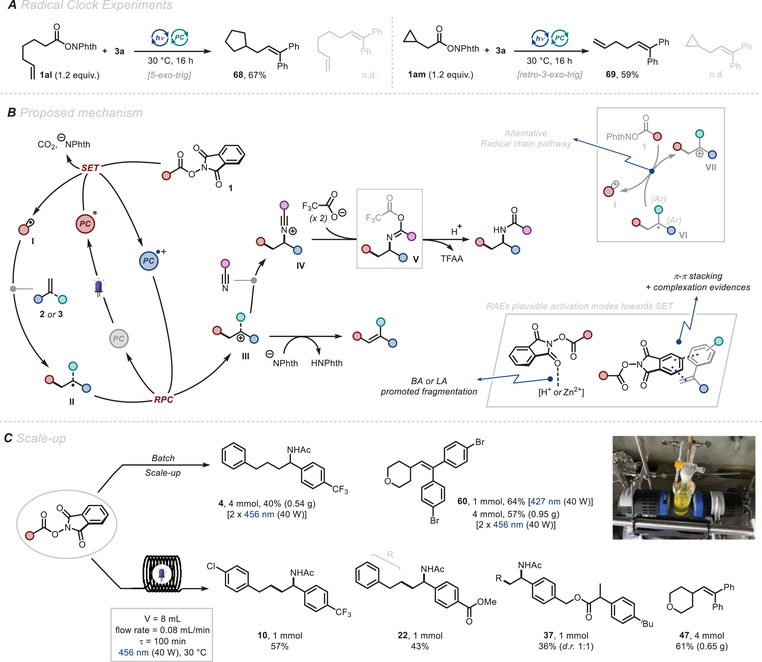
A) Radical clock experiments: redox active ester (1.2 equiv.) and **3a** (1.0 equiv.) in DMF (p.a., 0.1 M). 0.3 mmol scale. Isolated yields are reported. B) Proposed mechanism. C) Scale‐up in batch and continuous flow for the decarboxylative processes. For experimental details see Supporting Information.

Next, we conducted a series of experiments to shed light on the mechanism of the photocatalytic transformations. Radical pathways were convincingly demonstrated through radical clock experiments (Figure 5A). In fact, the isolation of compounds **68** and **69** starting from NHPI esters **1al** and **1am** provided strong evidences for the formation of cyclopentyl methyl and cyclopropyl methyl radicals via favored 5‐*exo*‐trig and retro‐3‐*exo*‐trig cyclization pathways, respectively.^[^ ^76^, ^77^ ^]^ Quantum yield measurements for both Ritter‐type carboamidation and Heck‐type reaction revealed that the transformations are photocatalytic in nature and that a radical chain is either absent or inefficient (*Φ* = 4.3 × 10^−3^ for Ritter‐type and *Φ* = 3.6 × 10^−3^ for Heck‐type, see Section S8.4, Supporting Information).^[^ ^78^ ^]^ On the basis of these results and those of previous studies,^[^ ^44^, ^46^, ^47^ ^]^ a proposed mechanism is presented in Figure 5B. Upon absorption of light, the excited state of **4‐CzIPN** (*E_1/2_ = −1.18 V vs SCE)^[^ ^79^ ^]^ is oxidatively quenched by the redox active ester **1**. This first step is promoted by LUMO lowering activation of **1** using TFA as Brønsted acid (or zinc as Lewis acid) under carboamidation conditions. Supportive of this proposal, cyclic voltammetry revealed a positive shift in the reduction potential of **1a** from −1.25 to −1.15 V vs SCE in the presence of 10 equivalents of TFA (see Section S8.2, Supporting Information). For the acid‐free Heck reaction, the SET event is proposed to be facilitated by a *π*─*π* stacking “Lewis acid‐type” activation, supported by detection of non‐covalent interactions between **1** and **3a** via NMR studies (see Section S8.3, Supporting Information).^[^ ^80^, ^81^ ^]^ The close interaction between the two reactants was further demonstrated by preliminary studies aimed at developing a catalyst‐free process. We observed that certain combinations of RAEs containing a phenyl group separated by an alkyl chain length of a specific size, together with **3a**, yielded Heck‐type products even in the absence of **4**‐**CzIPN** (for further details, see Section S5.3, Supporting Information). This implied an electron donor–acceptor (EDA) complex pathway may arise between 1,1‐diarylolefin and RAEs that contain phenyl groups tethered at a certain length. However, given the crucial role of the photocatalyst in delivering higher yields and the limited generality of the donor–acceptor‐type complex formation,^[^ ^82^, ^83^ ^]^ the method was not further explored for synthetic applicability.

Upon SET reduction, the entropically‐driven fragmentation of **1** generates CO_2_, phthalimide anion and alkyl radical **I**. The latter then engages in a Giese‐type addition to **2** or **3**, yielding benzylic radical intermediate **II** (*E*_1/2_ ≃ from +0.23 to +0.73 V vs SCE).^[^ ^84^, ^85^ ^]^ Subsequently, this is oxidized by **4‐CzIPN^•+^** (*E*_1/2_ = +1.49 V vs SCE) in the RPC step, yielding carbocation **III** and closing the photocatalyst cycle. At this point, for the carboamidation **III** is trapped by the nitrile, forming nitrilium ion **IV**. Hydrolysis of the latter may proceed via nucleophilic attack by trifluoroacetate, forming the trifluoroacetimidate **V**, which finally undergoes deacylation to yield the difunctionalized product and trifluoroacetic anhydride (TFAA).^[^ ^49^ ^]^ Alternatively, adventitious water present in TFA hydrolyses the nitrilium ion as per a classical Ritter reaction. For the Heck‐type process, deprotonation of **III** by the phthalimide anion yields the Heck‐type product. An alternative pathway for the Heck‐type reaction could involve a radical chain with inefficient propagation, deriving from SET between the incipient diaryl benzylic radical **VI** (*E*_1/2_ ≃ −1.34 V vs SCE)^[^ ^85^ ^]^ and **1**, leading to the formation of the benzylic cation **VII** and **I** (Figure 5B). Although very low quantum yields were measured that did not accord with this proposal, values of *Φ* < 1 do not completely rule out chain pathways.

**Figure 6:**
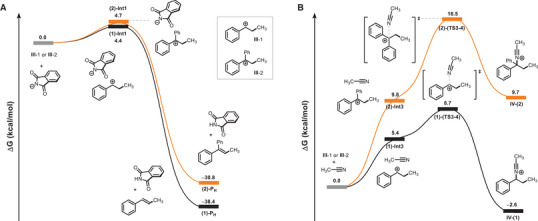
Calculated B3LYP‐D3 free energy profiles for the A) Heck‐type reaction and B) Ritter‐type carboamidation. Energies are in kcal/mol and are relative to the asymptote of the sum of reactants.

To gain deeper insight into the origin of the reactivity differences observed for the different radical acceptors, density functional theory (DFT) calculations were performed (see Section S13, Supporting Information). Specifically, we focused on steps originating from carbocation **III**, which are represented, for simplicity, following attack of a methyl radical and subsequent RPC. Calculations were performed at the B3LYP‐D3, 6–31 + G* theory level. In the Heck‐type reaction, the phthalimide anion approaches the styrene and the 1,1‐diarylbenzylic carbocation, referred to as **III**‐(1) and **III**‐(2), respectively, in Figure 6A. Our DFT results showed that this step is slightly endothermic due to the entropic effects associated with bringing the phthalimide anion into the vicinity of the carbocation. However, these effects are not significantly enhanced in the presence of an additional aryl ring in **III**, as indicated by the nearly identical calculated energies of 4.4 and 4.7 kcal/mol for (1)‐**Int1** and (2)‐**Int1**, respectively. The optimized geometries of these intermediates (see Figure S32A, Supporting Information) revealed that a hydrogen‐bond interaction is established between the nitrogen of the phthalimide base and the hydrogen of the CH_2_ group of **III**‐(1) and **III**‐(2), with the bond in the former being 0.05 Å longer than in the latter. On the other hand, the two aryl rings of the phthalimide anion and **III**‐(1) in (1)‐**Int1** are closer than in (2)‐**Int1**, suggesting a stronger *π*─*π* interaction between the base and the styrene carbocation. With the base in close proximity to the carbocations, the deprotonation step can proceed. Our DFT results indicated that this step is equally feasible for both styrene and 1,1‐diphenylethene, occurring spontaneously, as supported by the scan analysis (see Figure S33, Supporting Information). However, Mulliken charge analysis underlined a less positive charge on the hydrogen atom involved in the elimination step in (1)‐**Int1** with respect to (2)‐**Int1**, whose calculated values are 0.311 and 0.277 for (1)‐**Int1** and 0.321 in (2)‐**Int1**, respectively. The higher positive charge of the hydrogen in (2)‐**Int1** underlines a higher acidic character and therefore supports the preferred Heck‐typer reaction for 1,1‐diphenylethene. Both Heck alkenylation products, (1)‐**P_H_** and (2)‐**P_H_**, are highly exothermic, with the former being more thermodynamically favored than the latter. Similarly to (1)‐**Int1** and (2)‐**Int1**, *π*─*π* interactions are observed between the aryl moieties of the base and the carbocations, with these interactions being stronger in (1)‐**P_H_**, as supported by the shorter calculated distances. On the other hand, in the products, both studied substrates are neutral, leading to a decrease in electrostatic interaction strength. As a result, the steric effect becomes dominant, stabilizing the less hindered (1)‐**P_H_** product more effectively.

In the Ritter‐type step following the generation of intermediate **III**, the MeCN molecule approaches the carbocation to form the nitrilium ion **IV**, thereby yielding **Int3** (Figure 6B). DFT calculations indicated that the formation of **Int3** is more endothermic compared to the production of **Int1**. Specifically, a small energy difference of only 1.0 kcal/mol is observed between (1)‐**Int1** and (1)‐**Int3**, whereas this difference increases to 5.1 kcal mol^−1^ when comparing (2)‐**Int1** and (2)‐**Int3**.

The minimal energy difference in the former case suggests that both (1)‐**Int1** and (1)‐**Int3** are accessible. However, under acidic conditions, TFA protonates the phthalimide anion, favoring the formation of (1)‐**Int3**. Furthermore, the energy barrier for the nucleophilic attack of MeCN nitrogen atom to the **III**‐(1) carbocation is calculated to be 8.7 kcal/mol, while the barrier increases significantly to 18.5 kcal/mol when the **III**‐(2) carbocation is involved. This indicates that the formation of nitrilium ion **IV** is more favorable in the presence of the styrene carbocation. Interestingly, while no energy stabilization is observed for the coordination of the nitrilium ion to **III**‐(2) (*cf*. **IV**‐(2), Figure 6B), the production of this intermediate is exothermic in the presence of the styrene carbocation. Overall, the DFT results suggested that although the Heck‐type deprotonation is equally feasible for both styrene and diphenylolefin reactions, the Ritter‐type reaction is consistently less favorable for 1,1‐diphenylolefins compared to styrene derivatives. This disparity is likely due to lower electrophilicity and enhanced hindrance of the diarylbenzylic cation. These findings are consistent with the experimental observation of the absence of carboamidated products when 1,1‐diarylolefins are employed as SOMOphiles in the presence of MeCN (*cf*. Table 2, Entry 1).

Finally, we demonstrated the scalability of our divergent process, in both batch and continuous flow (Figure 5C). Regarding batch experiments, a simple set‐up involving two Kessil lamps (456 nm, 50% intensity) enabled production of half a gram of carboamidation product **4**. Additionally, the Heck‐type reaction was achieved on a 4 mmol, gram‐scale synthesis of **60**, a product highly attractive for post‐functionalization due to the presence of two C(sp^2^)─Br bonds. Continuous flow technology was leveraged,^[^ ^86^ ^]^ facilitating rapid and efficient scale‐up of both carboamidation and Heck‐type products. In fact, under optimized conditions, a residence time (*τ*) of just 100 min was sufficient, notably decreasing reaction time compared to the 16 h batch process (for further details see Section S9.2, Supporting Information). With this approach, amide products **10**, **22**, and **37** were successfully synthesized on a 1 mmol scale, while trisubstituted alkene product **47** was produced on a 4 mmol scale, demonstrating in both cases satisfactory overall space time yields (up to 36 mmol L^−1^ h^−1^).^[^ ^87^ ^]^

## 3 Conclusion

In summary, we have disclosed a visible‐light–mediated three component Ritter‐type olefin carboamidation using readily accessible carboxylic acid‐derived redox‐active esters. In this study, leveraging the organophotocatalyst **4‐CzIPN**, we successfully *(i)* tamed previously unexplored decarboxylative‐generated nucleophilic primary radicals,^[^ ^88^ ^]^ enabling their incorporation in the three‐component process with styrenes and nitriles, and *(ii)* developed a general retrosynthetic disconnection strategy to rapidly increase molecular complexity, offering a novel route to amidated precursors for the synthesis of pharmaceutically relevant scaffolds. Furthermore, a serendipitous chemodivergetcence has been explored when 1,1‐diarylolefins were utilized as reaction partners, leading to a straightforward and additive‐free photocatalytic Heck‐type reaction methodology. A plausible mechanism has been proposed, supported by mechanistic studies and DFT calculations, explaining both the activation pathways towards alkyl radical generation and the fate of intermediate **III** in yielding the target products depending on the radical acceptor partner. Both transformations demonstrated good functional group tolerance with respect to aryl olefins and redox‐active esters, resulting in broad applicability. Scalability in both batch and continuous‐flow processes has demonstrated the effectiveness and the synthetic utility of the presented protocols.

## Supporting Information

Additional references cited within the Supporting Information.^[^ ^89^, ^90^, ^91^, ^92^, ^93^, ^94^, ^95^, ^96^, ^97^, ^98^, ^99^, ^100^, ^101^, ^102^, ^103^, ^104^, ^105^, ^106^, ^107^, ^108^, ^109^, ^110^, ^111^, ^112^, ^113^, ^114^, ^115^, ^116^, ^117^, ^118^, ^119^, ^120^, ^121^, ^122^, ^123^, ^124^, ^125^, ^126^, ^127^ ^]^

## Conflict of Interests

The authors declare no conflict of interest.

## Additional Data

The X‐ray crystallographic coordinates for products reported in this study have been deposited at the Cambridge Crystallographic Data Centre (CCDC) under deposition numbers CCDC 2414659 (**4**), CCDC 2414660 (**44**) and CCDC 2414661 (**67**).

## Supporting information



Supporting Information

Supporting Information

Supporting Information

Supporting Information

## Data Availability

The data that support the findings of this study are available in the supplementary material of this article.
