# Supplementary material for: Photocatalysis Enables Chemodivergent Radical Polar Crossover: Ritter‐Type Amidation vs Heck‐Type Olefin Carbofunctionalizations
Source: Chemistry. 2025 May 6;31(33):e202500666. doi: 10.1002/chem.202500666 (PMC12160998; doi:10.1002/chem.202500666)

Table of Contents

1. General information 1
2. Chart of RAEs and olefins used in the study 2
3. Synthesis of starting materials 4
   3.1 General Procedure (**GP1**) for the synthesis of NHPI redox active esters 4
   3.2 General Procedure (**GP2**) for the synthesis of 1,1'-disubstituted olefins 4
   3.3 Synthesis of **2m** 5
   3.4 Synthesis of **2n** 5
4. Photoreaction setup 6
5. Optimization 7
   5.1 Optimization of the Ritter-type carboamidation 7
   5.2 Optimization of the Heck-type reaction 12
   5.3 Preliminary exploration of a Photocatalyst-free Heck-type reaction 14
6. General Procedure (**GP3**): Photoredox catalyzed Ritter-type carboamidation reaction 15
7. General Procedure (**GP4**): Photoredox catalyzed Heck-type reaction 15
8. Mechanistic investigations 16
   8.1 Radical clock experiments 16
   8.2 Cyclic voltammetry measurements 17
   8.3 NMR studies 19
   8.4 Quantum yield experiments 23
9. Scale-up procedures 25
   9.1 Scale-up in batch 25
   9.2 Scale-up in continuous flow 27
   9.3 Space time yields 30
10. Characterization data of synthesized compounds 31
   10.1 Characterization data of NHPI esters 31
   10.2 Characterization data of Ritter-type carboamidation products 33
   10.3 Characterization data of Heck-type products 47
11. X-Ray crystal data of products 59
12. Limitation of the scope 62
13. Computational studies 63
   13.1 Optimized structures of the intercepted stationary points 63
   13.2 Cartesian coordinates of the intercepted stationary points 64
   13.3 Scan analysis 67
14. References 68
15. NMR spectra of Ritter-type carboamidation products 76
16. NMR spectra of Heck-type products 124

1. General information

All reagents and solvents were used as received without further purification, unless stated otherwise. Reagents and solvents were bought from Sigma Aldrich, TCI, Fluka, FluoroChem, BLD-Pharma and Fisher Scientific and, if applicable, kept under nitrogen (N_2) atmosphere. Photocatalysts **4-CzIPN** and **3DPA2FBN** were prepared according to published procedures.[89,90] Anhydrous THF (stabilizer-free) and MeCN were obtained from a MBRAUN SPS purification system and stored under N_2 over 3 Å molecular sieves. DMF was distilled from CaH_2 under reduced pressure (9 mbar) and stored under N_2 over 4 Å molecular sieves. Disposable syringes were purchased from B.Braun. Product isolation was performed manually using flash chromatography, using silica gel 60 (Macherey Nagel 0.063 – 0.2 mm). TLC analysis was performed using Silica on aluminum foil-backed TLC plates (Macherey Nagel: Alugram Xtra SIL G UV254 Nr. 818333, thickness 0.2 mm) with visualization under ultraviolet light (254 nm and 365 nm) or with an appropriate TLC stain (cerium ammonium molybdate or potassium permanganate).

^{1}H NMR spectra were recorded on Bruker Avance 300 (300 MHz) or Bruker Avance 400 (400 MHz). Chemical shifts are reported in parts per million (ppm) using $CDCl_3$ (7.26 ppm) or CD_3CN (1.94 ppm) as an internal reference. The following abbreviations were adopted to describe the multiplicity: br. s (broad singlet), s (singlet), d (doublet), t (triplet), q (quartet), p (pentet), h (hextet), hept (heptet), dd (doublet of doublets), td (triplet of doublets), tt (triplet of triplets), dq (doublet of quartets), ddd (doublet of doublets of doublets), ddt (doublet of doublets of triplets), dtd (doublet of triplets of doublets, m (multiplet). Coupling constants (J) are reported in hertz (Hz). ^{13}C NMR spectra were recorded on Bruker Avance 300 (75 MHz), Bruker Avance 400 (101 MHz), Bruker Avance 500 (126 MHz) or Bruker Avance 600 (151 MHz) spectrometer. Chemical shifts for ^{13}C NMR were reported in parts per million (ppm), using $CDCl_3$ (77.0 ppm, center line signal of the triplet) as an internal reference. ^{19}F NMR spectra were recorded on Bruker Avance 400 (376 MHz) spectrometer.

Mass spectra were recorded by the Central Analytical Department of the University of Regensburg using Jeol AccuTOF GCX and Agilent Q-TOF 6540 UHD Spectrometers. High-resolution mass spectra were measured using atmospheric pressure chemical ionization (APCI), electron ionization (EI) or electrospray ionization (ESI) with a quadrupole time-of-flight (Q-TOF) detector.

For all the photochemical batch and flow experiments (optimization and scope), 3D-printed (PLA) reactors were fabricated which were internally coated with aluminum foil and equipped with a specific 3D-printed (PLA) lid serving as a vial/coil holder and a lamp holder (see Section 4 for details). Crimp cap vials (5 and 20 mL) and lids for batch photochemical reactions were purchased from WICOM.

2. Chart of RAEs and olefins used in the study

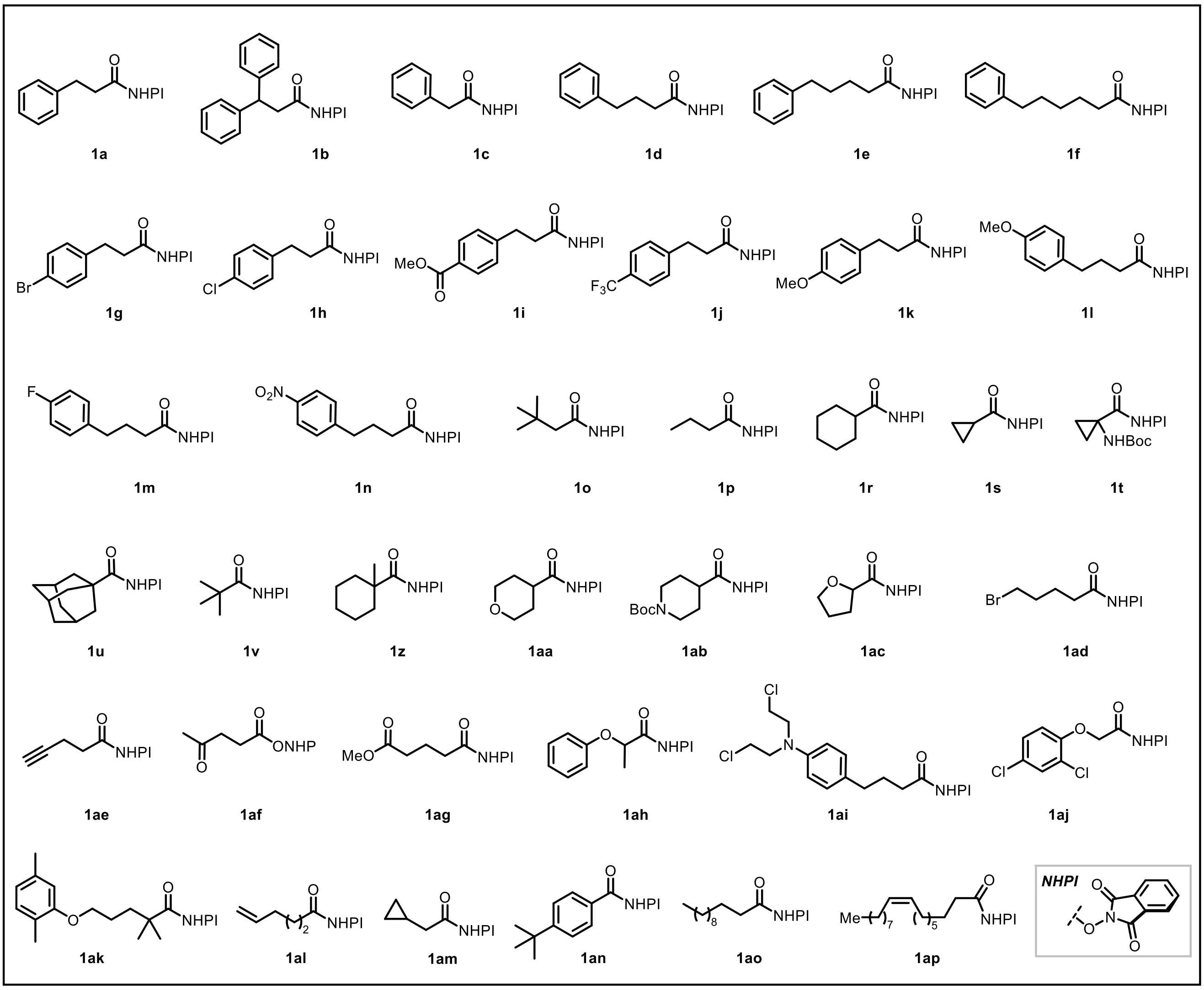

Figure S1: Structures of redox-active esters used in the substrate scope and optimization studies.

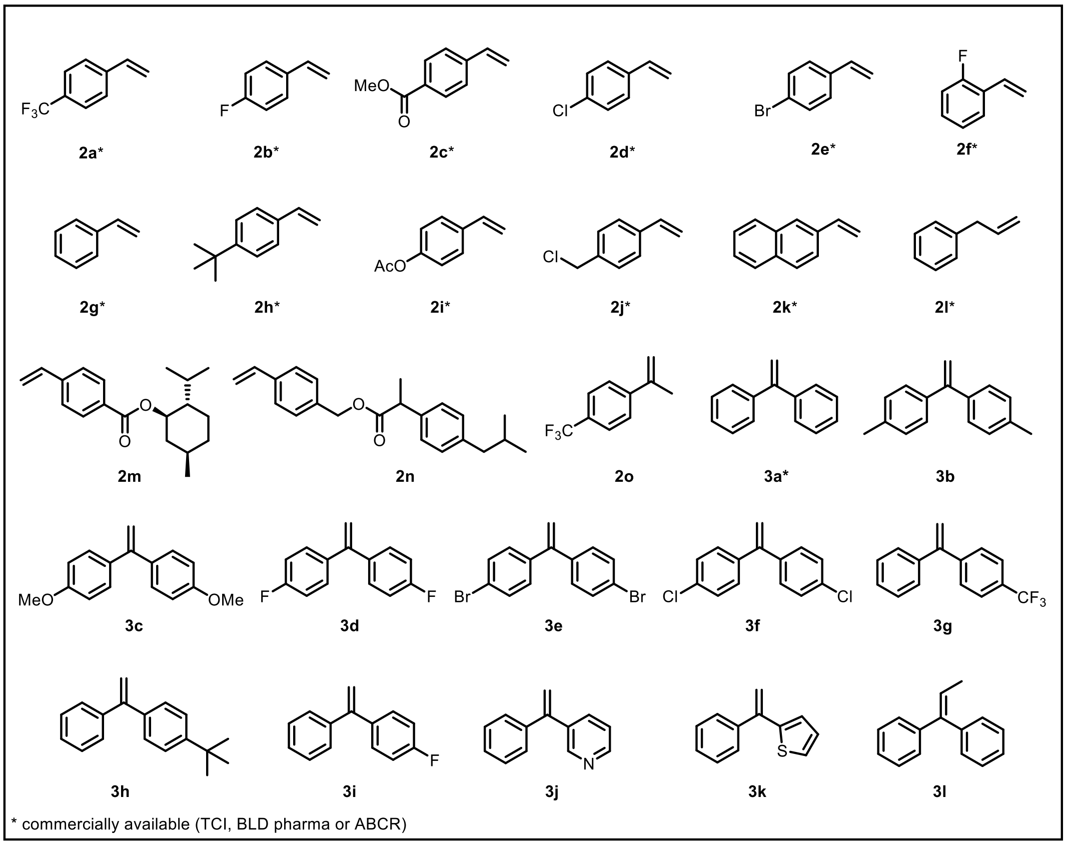

Figure S2: Structures of styrenes and 1,1'-diarylolefins used in the substrate scope and optimization studies.

3. Synthesis of starting materials

3.1 General Procedure (GP1) for the synthesis of NHPI redox active esters

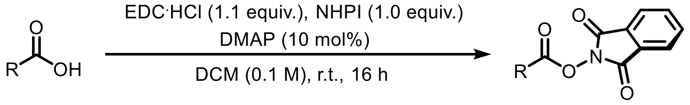

Compounds **1a-1ao** were synthesized adapting a procedure reported in the literature.[91] EDC·HCl (1.1 equiv.), N-hydroxyphthalimide (1.0 equiv.), 4-dimethylaminopyridine (10 mol%, 0.1 equiv.) and the corresponding carboxylic acid (1.0 equiv.) were added, followed by DCM (0.1 M). The resulting solution was stirred at room temperature for 16 h. The organic phase was washed twice with 1 N HCl, twice with sat. aq. $NaHCO_3$ solution, twice with sat. aq. NaCl solution and dried over anhydrous Na_2SO_4. The solvent was then removed under reduced pressure. When needed, purification via flash column chromatography on silica gel or recrystallization from ethanol afforded the desired product. For newly reported RAEs see Section 10.1 for characterization data.

3.2 General Procedure (GP2) for the synthesis of 1,1'-disubstituted olefins

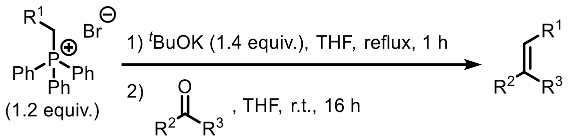

Olefins **2o** and **3b-3k** were synthesized adapting a procedure reported in the literature.[92] To a suspension of alkyl triphenylphosphonium bromide (1.2 equiv) in dry THF (5 mL/mmol of phosphonium) under N_2 atmosphere was added a solution of ^{t}BuOK (1.4 equiv. or 1.0 equiv. when an enolizable ketone was present) in THF (1 mL/mmol), and the reaction mixture turned to a bright yellow colour. The reaction mixture was then heated to reflux with an oil bath for 1 h. Then, the ketone (1 equiv.) was added portionwise and the resulting mixture was stirred at room temperature for 16 h. Upon completion, pentane (3 mL/mL of THF) was added and the precipitates were filtered over celite. The filtrate was concentrated under reduced pressure and the residue was purified via flash column chromatography on silica gel.

3.3 Synthesis of 2m

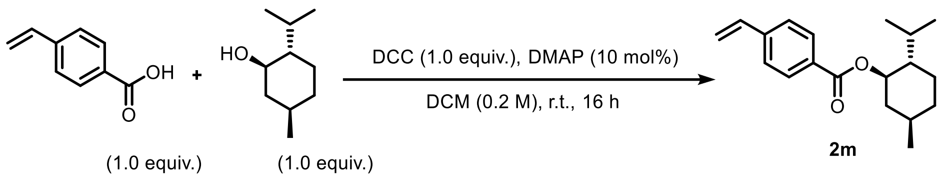

Compound **2m** was synthesized adapting a procedure reported in the literature.[93] To a stirring solution of (1R,2S,5R)-2-isopropyl-5-methylcyclohexan-1-ol (468 mg, 3 mmol, 1.0 equiv.), 4-vinylbenzoic acid (444 mg, 3 mmol, 1.0 equiv.) and 4-dimethylaminopyridine (37 mg, 10 mol%, 0.10 equiv.) in DCM (15 mL, 0.2 M) was added N,N'-Dicyclohexylcarbodiimide (619 mg, 3 mmol, 1.0 equiv.) dropwise. Then, the reaction mixture was stirred overnight at room temperature. Upon completion, DCM (10 mL) was added and the mixture was cooled (to -20 °C) and stirred for 15 minutes, then filtered. The solvent was removed under reduced pressure and the crude reaction mixture was purified via flash column chromatography on silica gel (Pentane:Ethyl Acetate 10:1) to afford **2m** as colorless oil (524 mg, 61% yield). Characterization data are in accordance with literature.[93]

^{1}H NMR (400 MHz, $CDCl_3$) δ 8.00 (d, *J* = 8.4 Hz, 2H), 7.46 (d, *J* = 8.3 Hz, 2H), 6.76 (dd, *J* = 17.6, 10.9 Hz, 1H), 5.86 (d, *J* = 16.8 Hz, 1H), 5.37 (d, *J* = 10.9 Hz, 1H), 4.93 (td, *J* = 10.9, 4.4 Hz, 1H), 2.16 – 2.09 (m, 2H), 2.00 – 1.91 (m, 1H), 1.76 – 1.71 (m, 2H), 1.60 – 1.52 (m, 2H), 1.17 – 1.04 (m, 2H), 0.92 (dd, *J* = 6.8, 5.3 Hz, 7H), 0.79 (d, *J* = 7.0 Hz, 3H).

3.4 Synthesis of 2n

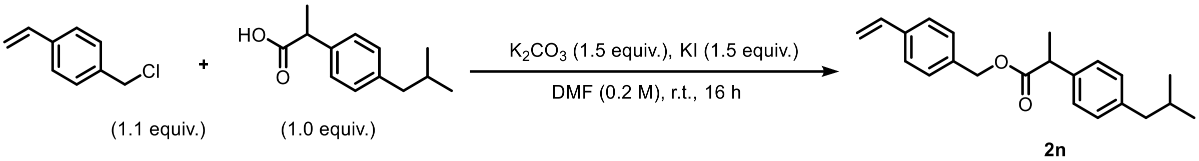

Compound **2n** was synthesized adapting a procedure reported in the literature.[94] To a solution of ibuprofen (618 mg, 3 mmol, 1.0 equiv.) in dry DMF (16 mL, 0.2 M) under N_2 atmosphere K_2CO_3 (621 mg, 4.5 mmol, 1.5 equiv.) and KI (747 mg, 4.5 mmol, 1.5 equiv.) were added. To the resulting vigorously stirring suspension, 4-vinylbenzyl chloride (503 mg, 3.3 mmol, 1.1 equiv.) was added and the mixture was stirred at room temperature for 16 h. Upon completion, ethyl acetate (50 mL) and water (10 mL) were added. The aqueous layer was extracted with ethyl acetate (3 x 50 mL) and the combined organic phase was washed with water (3 x 20 mL) and dried over anhydrous Na_2SO_4. The crude reaction mixture was then concentrated under reduced pressure and purified via flash column chromatography on silica gel (Pentane:Ethyl Acetate 10:1) to afford **2n** as a colorless oil (874 mg, 90% yield). Characterization data are in accordance with literature.[94]

^{1}H NMR (400 MHz, $CDCl_3$) δ 7.34 (d, *J* = 7.9 Hz, 2H), 7.22 – 7.17 (m, 4H), 7.09 (d, *J* = 7.7 Hz, 2H), 6.69 (dd, *J* = 17.6, 10.9 Hz, 1H), 5.76 (d, *J* = 1.1 Hz, 1H), 5.25 (d, *J* = 10.9 Hz, 1H), 5.09 (s, 2H), 3.75 (q, *J* = 7.1 Hz, 1H), 2.45 (d, *J* = 7.2 Hz, 2H), 1.90 – 1.79 (m, 1H), 1.51 (d, *J* = 6.3 Hz, 3H), 0.90 (d, *J* = 5.6 Hz, 6H).

4. Photoreaction setup

4 or 6 vials photoreactor for 5 mL vials used in the optimization (0.1 mmol scale)

Reaction vials were irradiated using the photoreactors shown below. A 40 W Kessil PR160L-427 nm with a linear reflector was used as an LED lamp, while the temperature was maintained at 30 – 35 °C via a fan positioned under the reactor and three fans around the reactor (left, right and behind) as shown in **Figure S3** and **Figure S4**. All the inside surfaces were covered with aluminum foil (**Figure S3** and **Figure S4**). The reactor was designed based on the model reported by Noël and co-workers in the literature.[95]

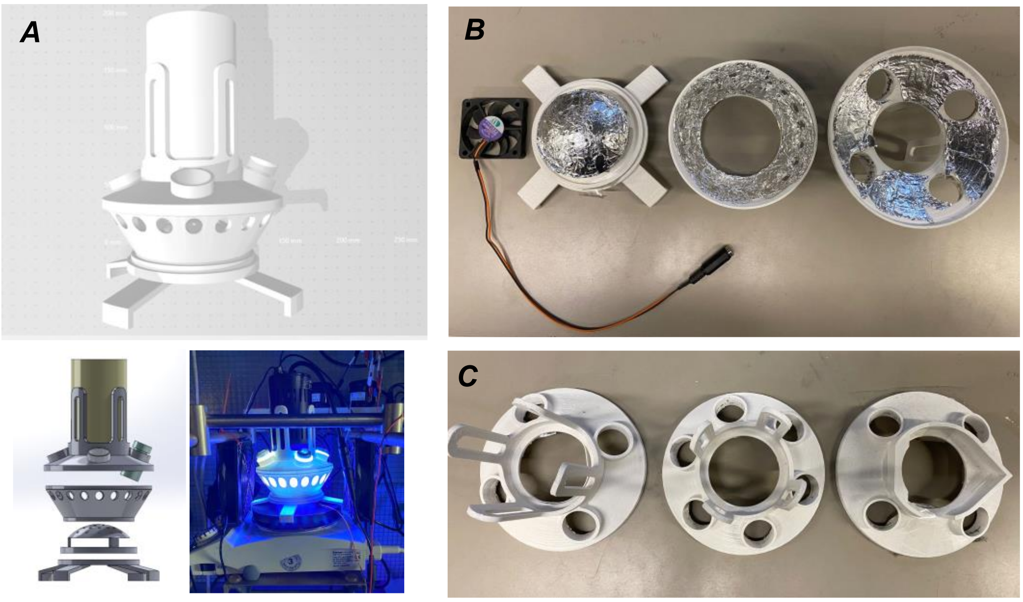

Figure S3: A) Overview of the 3D-printed reactor. B) Inside of the reactor. C) Different lids for 4, 6 reactions and different light sources.

4 vials photoreactor for 20 mL vials used in the substrate scope (0.3 mmol scale)

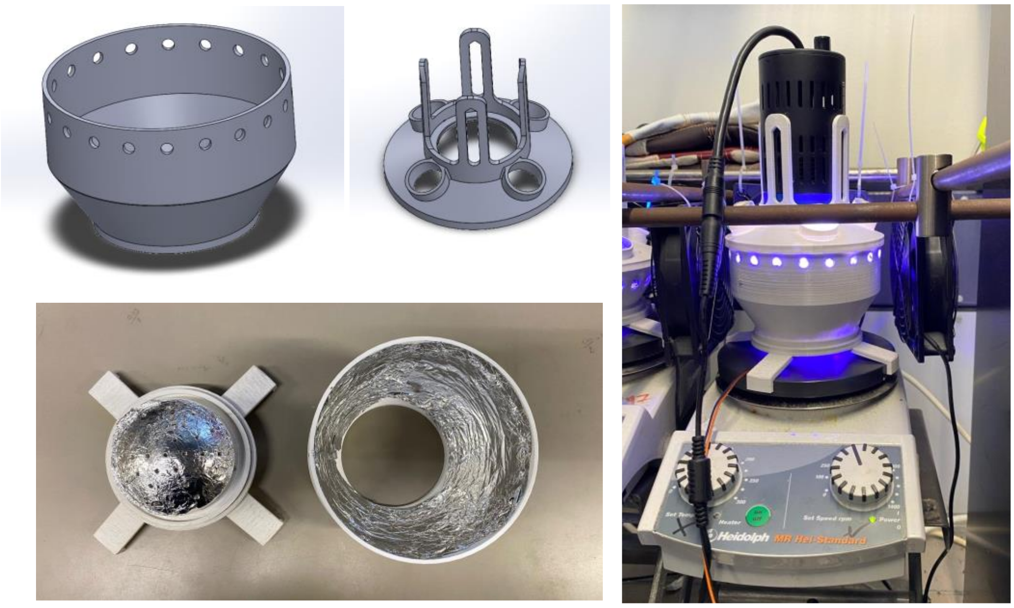

Figure S4: 20 mL vials 3D-printed photoreactor.

5. Optimization

5.1 Optimization of the Ritter-type carboamidation

Table S1: Photocatalyst (PC) screening.

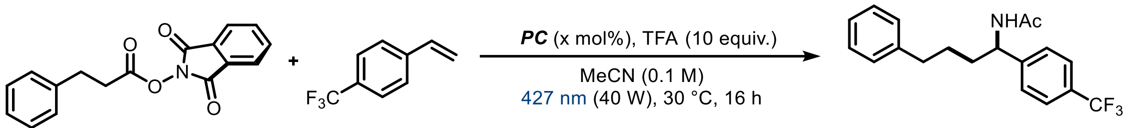

1a (1 equiv.) **2a** (1.2 equiv.) **4**

Entry	**PC** (x mol%)	Yield of **4** (%)[a]
1	**4-CzIPN** (5)	50
2	**4-DPAIPN** (5)	27
3	**3DPA2FBN** (5)	30
4	**3DPAFIPN** (5)	27
5	**Ir(ppy)$_3$** (1)	32
6	**Ir(dF(CF$_3$)ppy)$_2$(dtppy))PF$_6$** (1)	23
7	**4-CzIPN** (5)[b]	35
8	**4-CzIPN** (5)[c]	n.d.
9	no **PC**	n.d.[d]
10	**4-CzIPN** (5)[e]	50

[a] Determined via ^{1}H NMR using 1,1,2,2-tetrachloroethane as an internal standard.
[b] **1ha-4Cl** used instead of **1a** (which has 4 Cl atoms on the NHPI group)
[c] **TT-OTf** used instead of **1a**. [d] 98% recovery of **1a**. [e] 40 h irradiation instead of 16 h.

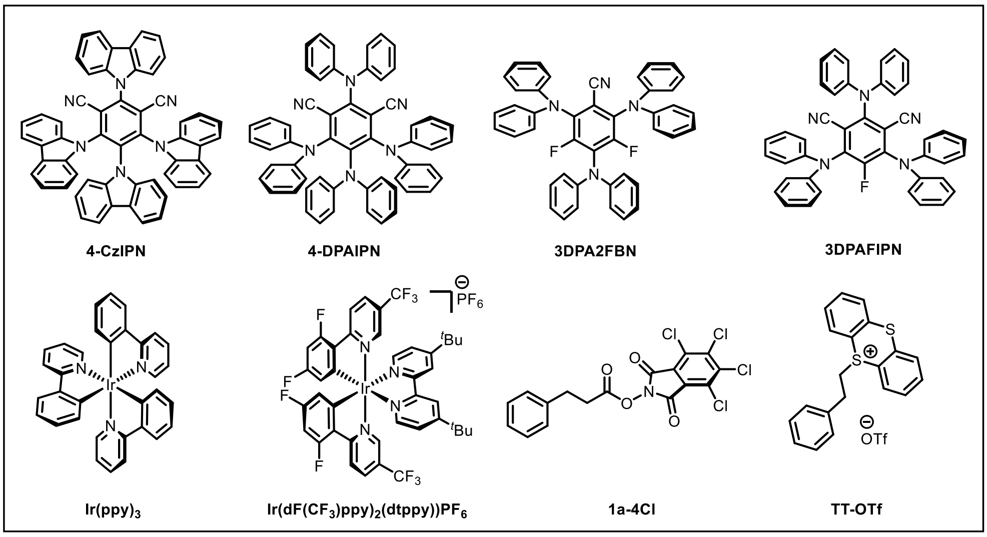

Figure S5: Structures of photocatalysts and primary carbon radical precursors attempted.

Table S2: Acid and water screening.

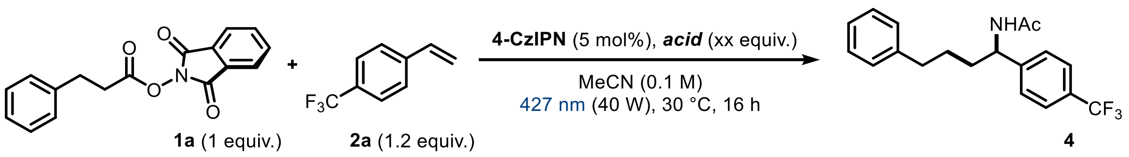

**Entry**	**Acid** (equiv.)	Yield of **4** (%)[a]
1	TFA (10)	50
2	AcOH (10)	10 (15)[b]
3	$PhCO_2H$ (5)	5 (11)[c]
4	TfOH (10)	n.d.
5	$(PhO)_2P(O)(OH)$ (2)	n.d.
6	H_2O (10)	n.d. (19)[d]

[a] Determined via ^{1}H NMR using 1,1,2,2-tetrachloroethane as an internal standard.
[b] In parenthesis, yield of **S2a**. [c] In parenthesis, yield of **S2b**. [d] In parenthesis, yield of **S2c**.

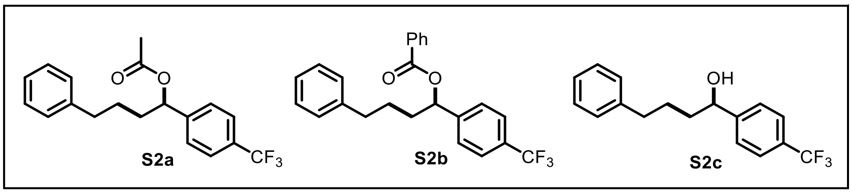

Table S3: TFA equivalents screening.

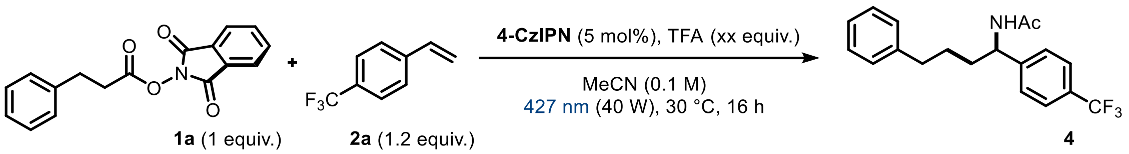

**Entry**	**TFA** (xx equiv.)	Yield of **4** (%)[a]
1	0	n.d.
2	1	39
3	5	44
4	10	50
5	20	50
6	50	44

[a] Determined via ^{1}H NMR using 1,1,2,2-tetrachloroethane as an internal standard.

Table S4: Reactants ratio screening.

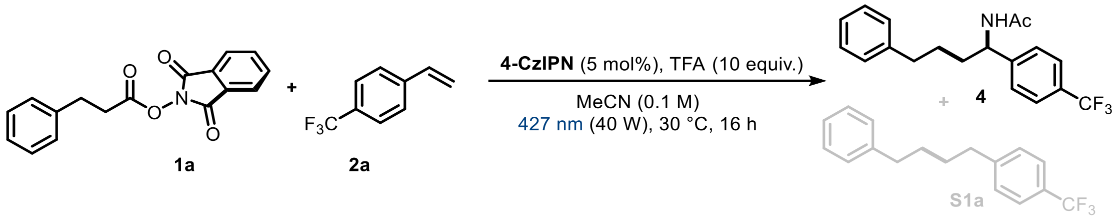

Entry	1a : 2a ratio	Yield of **4** (%)[a]	Yield of **S1a** (%)[a]
1	1:1	40	13
2	1:1.2	50	14
3	1:2	41	13
4	1.5:1	50	31
5	2:1	49	35

[a] Determined via ^{1}H NMR using 1,1,2,2-tetrachloroethane as an internal standard.

Although the hydroalkylation **S1a** by-product was not isolated, it was detected by GC-MS (**Figure S6**).[96] Quantification of **S1a** was then evaluated via ^{1}H NMR using the benzylic protons, with chemical shifts matching those reported in literature.[97]

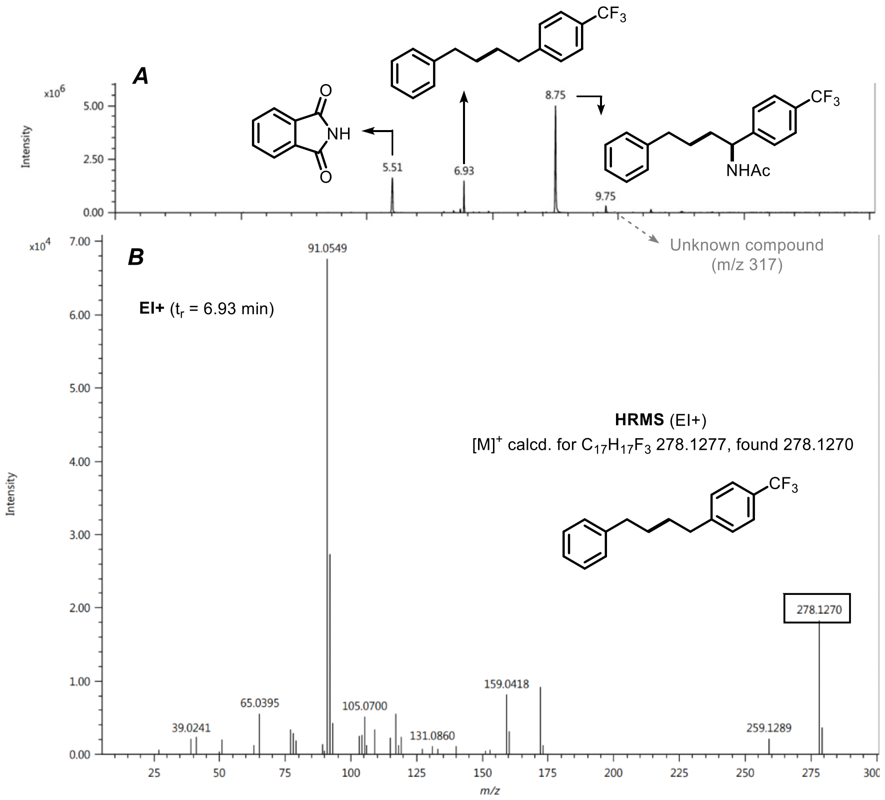

Figure S6: A) GC-MS chromatogram of a sample from a Ritter-type reaction crude. B) High resolution mass spectrum of the hydroalkylation by-product.

Hydrogen atom transfer from MeCN to generate **S1a** from **II** (*cf.* **Figure 5B** of the manuscript) was ruled out since no alkyldeuteration by-product was detected in the GC-track when reaction was conducted in CD_3CN, as shown in **Figure S7**.

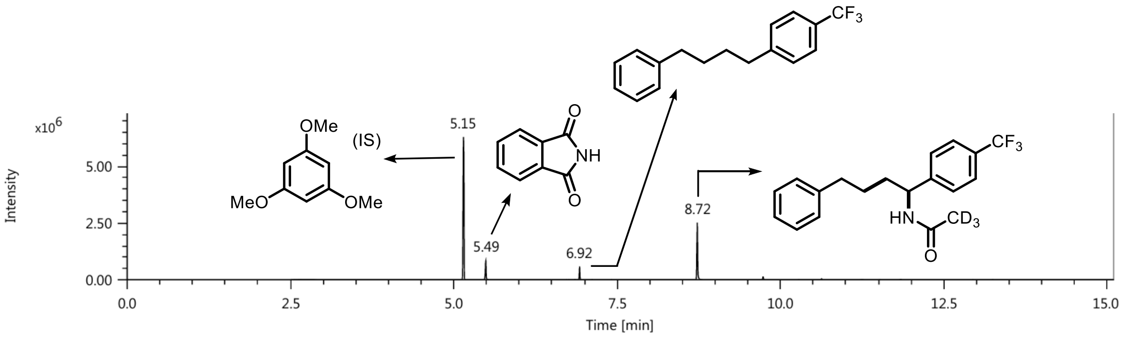

Figure S7: GC-MS chromatogram of a sample from a Ritter-type reaction crude conducted with CD_3CN as solvent.

Table S*5*: Light source wavelength screening.

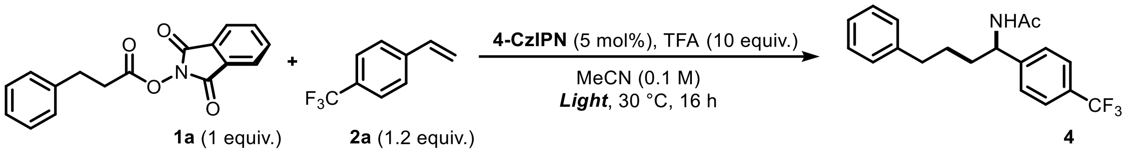

Entry	Light source	Yield of **4** (%)[a]
1	427 nm Kessil (40 W, full intensity)	50
2	456 nm Kessil (40 W, full intensity)	47
3	390 nm Kessil (52 W, full intensity)	44
4	450 nm LEDs (50 W)	39

[a] Determined via ^1H NMR using 1,1,2,2-tetrachloroethane as an internal standard.

Table S6: Light intensity screening.

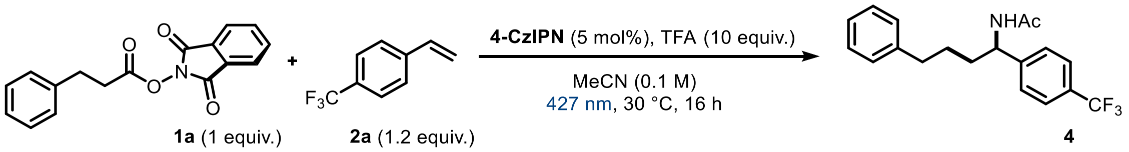

Entry	Kessil intensity (%)	Yield of **4** (%)[a]
1	25	40
2	50	47
3	100	50

[a] Determined via ^1H NMR using 1,1,2,2-tetrachloroethane as an internal standard.

Table S7: Additive screening.

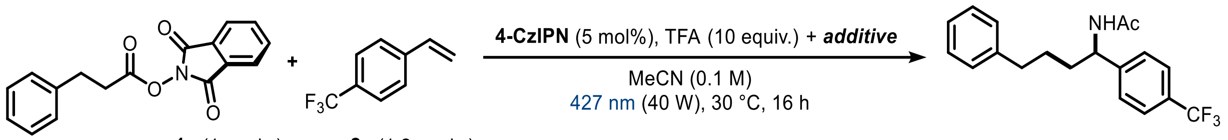

1a (1 equiv.) **2a** (1.2 equiv.) **4**

Entry	Additive	Yield of **4** (%)[a]
1	none	50
2	KF (2 equiv.)	38
3	$BF_3 \cdot OEt_2$ (10 equiv.)	n.d.
4	CF_3CO_2Na (5 equiv.)	36
5	$In(OTf)_3$ (20 mol%)	46

[a] Determined via ^1H NMR using 1,1,2,2-tetrachloroethane as an internal standard.

Table S8: Metal salts $M(OX)_n$ screening.

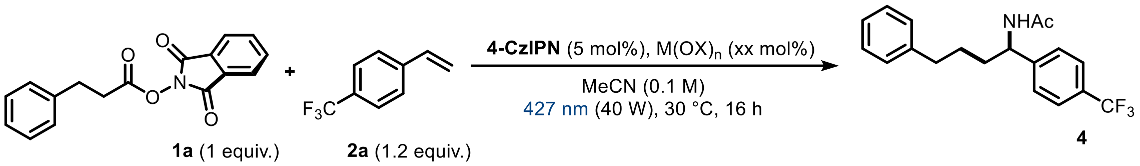

1a (1 equiv.) **2a** (1.2 equiv.) **4**

Entry	**$M(OX)_n$** (xx mol%)	Yield of **4** (%)[a]
1	$In(OTf)_3$ (20)	41
2	$In(OTf)_3$ (50)	43
3	$Cu(OTf)_2$ (50)	n.d.
4	LiOTf (50)	n.d.
5	$Zn(OTf)_2$ (50)	38
6	$Zn(CF_3COO)_2 \cdot xH_2O$ (50)	42

[a] Determined via ^1H NMR using 1,1,2,2-tetrachloroethane as an internal standard

5.2 Optimization of the Heck-type reaction

Table S9: Solvent screening (with or without TFA).

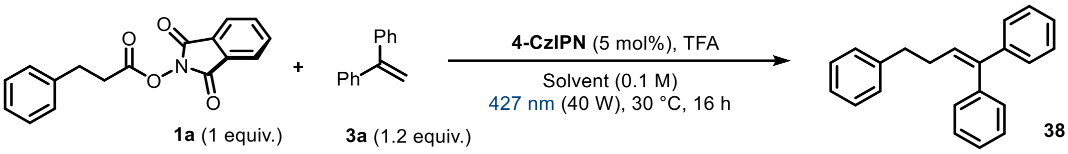

38

**Entry**	**Solvent**	TFA (10 equiv.)	Yield of **38** (%)[a]
1	Dry MeCN	yes	55
2	Dry MeCN	no	38
3	Dry DMF	no	52
4	Analytical grade DMF	yes	57
5	Analytical grade DMF	no	62
6	Analytical grade DMF[b]	no	61
7	Dry MeCN[c]	yes	n.d.

[a] Determined via ^{1}H NMR using 1,1,2,2-tetrachloroethane or 1,3,6-trimethoxybenzene as an internal standard.
[b] 456 nm Kessil (full intensity).
[c] No **PC** added; 99% recovery of **1a**.

Table S10: Reactants ratio screening.

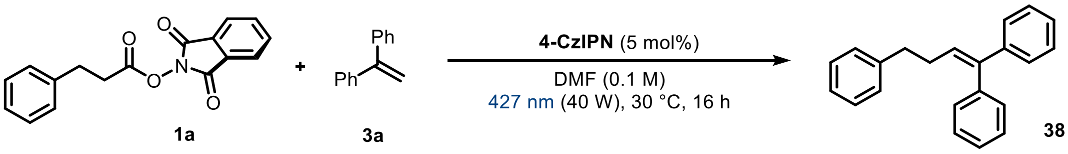

38

**Entry**	**1a : 3a ratio**	Conversion of **1a** (%)[a]	Conversion of **3a** (%)[a]	Yield of **38** (%)[a]
1	1:1.2	100	88	62
2	1:1	100	87	47
3	1.2:1	100	98	59
4	1.5:1	100	100	51

[a] Determined via ^{1}H NMR using 1,1,2,2-tetrachloroethane or 1,3,6-trimethoxybenzene as an internal standard.

As shown in **Figure S8** for the reaction of **1r** and **3a**, no Ritter-type carboamidation product (exact mass 321.2093) could be detected when reaction was conducted in MeCN and in presence of 10 equiv. of TFA (as per entry 1 in **Table 2** of the manuscript).

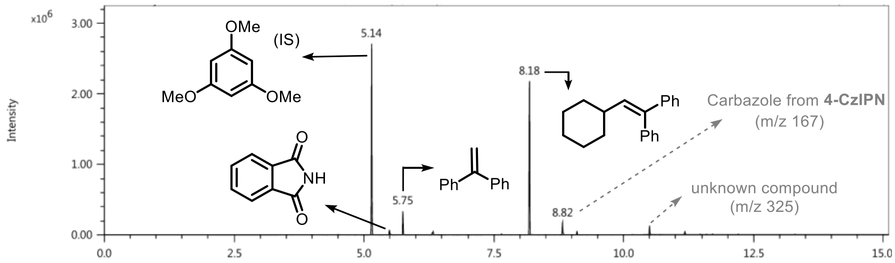

Figure S8: GC-MS chromatogram of a sample from a Heck-type reaction.

During the substrate scope evaluation, examination of the crude reaction mixture of the synthesis of **66** by GC-MS (**Figure S9A**) revealed a peak with a mass spectrum and fragmentation pattern matching the literature data for (Z)-heptadec-8-ene (**Figure S9B**).[98] Supporting the decarboxylative reduction product formation, TLC analysis showed a spot (R_f = 0.8 in pentane) that was not visible under UV light but appeared with $KMnO_4$ staining. This clearly indicates that in the Heck-type reaction, the remaining mass balance can be accounted for by the decarboxylation reduction by-product. As for the reaction of standard NHPI ester **1a**, the by-product was not detected in the crude after evaporation of DMF, presumably due to its relative volatility (ethylbenzene, b.p. 136 °C).

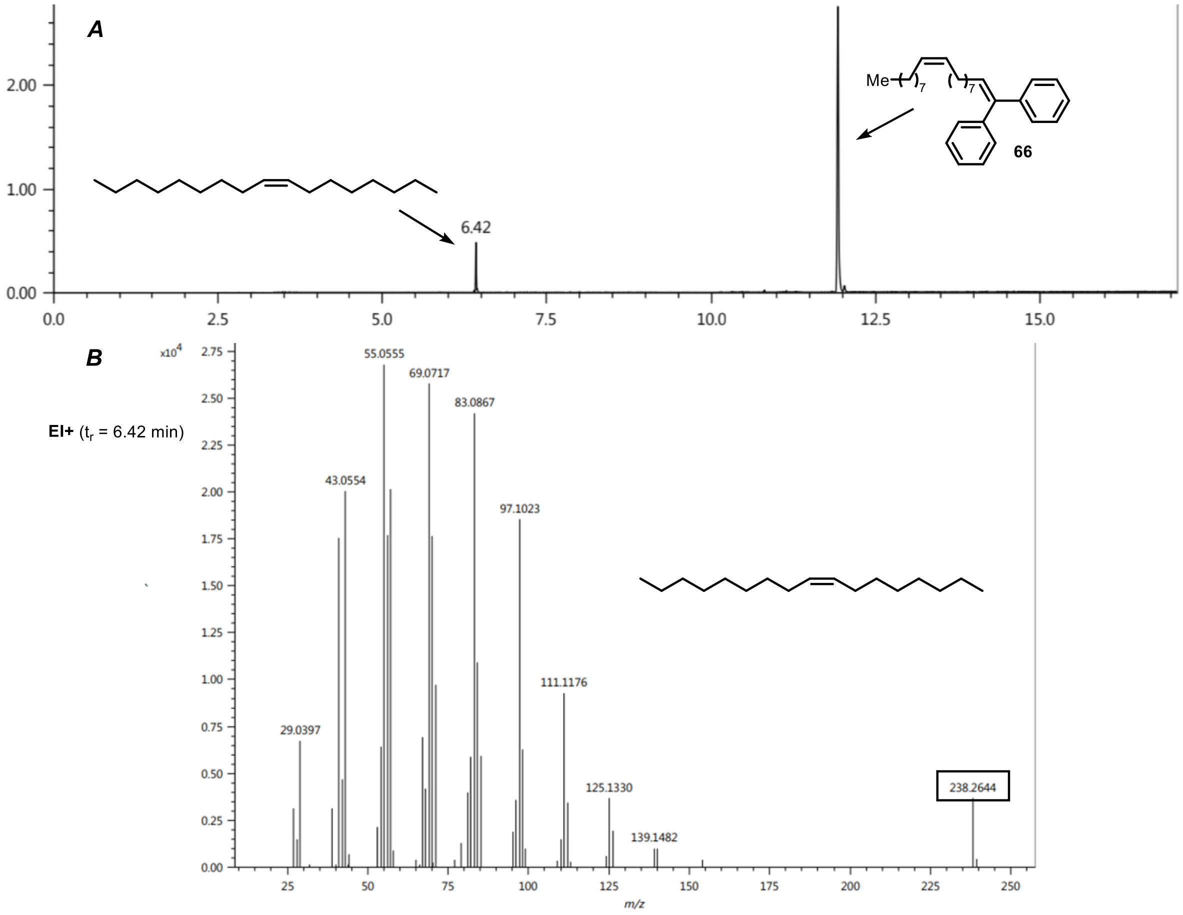

Figure S9: A) GC-MS chromatogram of a sample from Heck-type reaction with oleic acid NHPI ester and 1,1-diphenyl ethylene. B) Electron ionization mass spectrum of the decarboxylative reduction by-product.

5.3 Preliminary exploration of a Photocatalyst-free Heck-type reaction

Table S11: Photocatalyst-free Heck-type reaction.

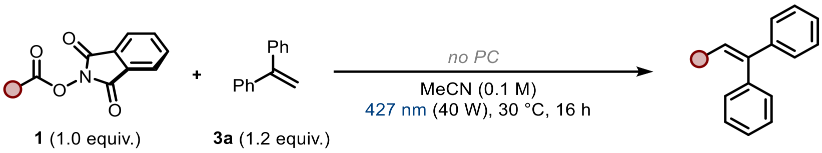

1 (1.0 equiv.) **3a** (1.2 equiv.)

Entry	NHPI ester	Conversion of **3a** (%)[a]	Yield (%)[a]
1	**1a** $[-(CH_2)_2Ph]$	<5	n.d. (> 95 of **1a**)
2	**1d** $[-(CH_2)_3Ph]$	52	31
3	**1e** $[-(CH_2)_4Ph]$	51	26
4	**1f** $[-(CH_2)_5Ph]$	50	16
5	**1d** (in DMA)	91	42
6	**1d** (in DMF)	84	43
7	**1d** (in DMSO)	84	44
8	**1d** (in acetone)	66	18
9	**1a-4Cl**[b]	88	34

[a] Determined via ^{1}H NMR using 1,1,2,2-tetrachloroethane as an internal standard. [b] 4 Cl atoms on the NHPI group

Interestingly, while the reaction using RAE **1a** (chain length = 2x CH_2) without photocatalyst did not proceed (entry 1), the reaction using **1d** (chain length = 3x CH_2) did (entry 2). As the alkyl chain length separating the NHPI group and Ph group increased (RAEs **1e** and **1f**; entries 3 and 4), the yield of Heck-type product decreased despite similar conversion. This suggests that non-covalent interactions between RAE and 1,1-diarylolefin **3a** may give rise to an electron donor-acceptor complex, assisted by a π-stacking interaction of the Ph tether of the RAE. Photoexcitation of which may give rise to inner-sphere SET and a pair of radical ions. The RAE undergoes decarboxylative cleavage to give a primary radical, which adds to the radical cation of the diarylolefin.

6. General Procedure (GP3): Photoredox catalyzed Ritter-type carboamidation reaction

In a typical experiment, an oven-dried 20 mL crimp cap vial equipped with a stirring bar was charged with redox active ester (0.30 mmol, 1.0 equiv.), styrene (0.36 mmol, 1.2 equiv., *if solid*) and **4-CzIPN** (11.7 mg, 5 mol%). After closing the vial, it was evacuated and back-filled with N_2 three times. Dry MeCN (3 mL) was added under N_2 atmosphere (0.1 M) and the resulting reaction mixture was bubbled with N_2 for 5 minutes. Afterwards, styrene (0.36 mmol, 1.2 equiv., *if liquid*) and TFA (3 mmol, 10 equiv.) were added via syringe. Alternatively (when stated in the characterization section), $Zn(CF_3COO)_2 \cdot xH_2O$ (43 mg, 0.5 equiv.) was used as additive instead of TFA. The reaction mixture was stirred and irradiated (427 nm Kessil, 100% light intensity) in the photochemical reactor (see Section 4) for 16 h. The temperature was maintained around 30 °C during the course of the reaction. Then, the vial was removed from the photochemical reactor and the mixture was added to a separatory funnel containing sat. aq. $NaHCO_3$ solution (10 mL) and ethyl acetate (10 mL). The aqueous layer was extracted with ethyl acetate (3 x 10 mL) and the combined organic phase was washed with sat. aq. NaCl (10 mL) and dried over anhydrous Na_2SO_4. The crude reaction mixture was then concentrated under reduced pressure and purified via flash column chromatography on silica gel.

7. General Procedure (GP4): Photoredox catalyzed Heck-type reaction

In a typical experiment, an oven-dried 20 mL crimp cap vial equipped with a stirring bar was charged with redox active ester (0.30 or 0.36 mmol, 1.0 or 1.2 equiv. as specified in the characterization section), 1,1-diaryl olefin (0.30 or 0.36 mmol, 1.0 or 1.2 equiv. as specified in the characterization section, if solid) and **4-CzIPN** (11.7 mg, 5 mol%). After closing the vial, it was evacuated and back-filled with N_2 three times. Analytical grade DMF (3 mL) was added under N_2 atmosphere (0.1 M) and the resulting reaction mixture was bubbled with N_2 for 5 minutes. In the case of liquid diarylolefins, these were added after degassing, via microsyringe, through the vial cap. The reaction mixture was stirred and irradiated (427 nm Kessil, 100% light intensity) in the photochemical reactor (see Section 4) for 16 h. The temperature was maintained around 30 °C during the course of the reaction. Then, the vial was removed from the photochemical reactor and the mixture was added to a separatory funnel containing sat. aq. NaCl solution (10 mL) and ethyl acetate (10 mL). The aqueous layer was extracted with ethyl acetate (3 x 10 mL) and the combined organic phase was washed with sat. aq. NaCl (10 mL) and dried over anhydrous Na_2SO_4. The crude reaction mixture was then concentrated under reduced pressure and purified via flash column chromatography on silica gel.

8. Mechanistic investigations

8.1 Radical clock experiments

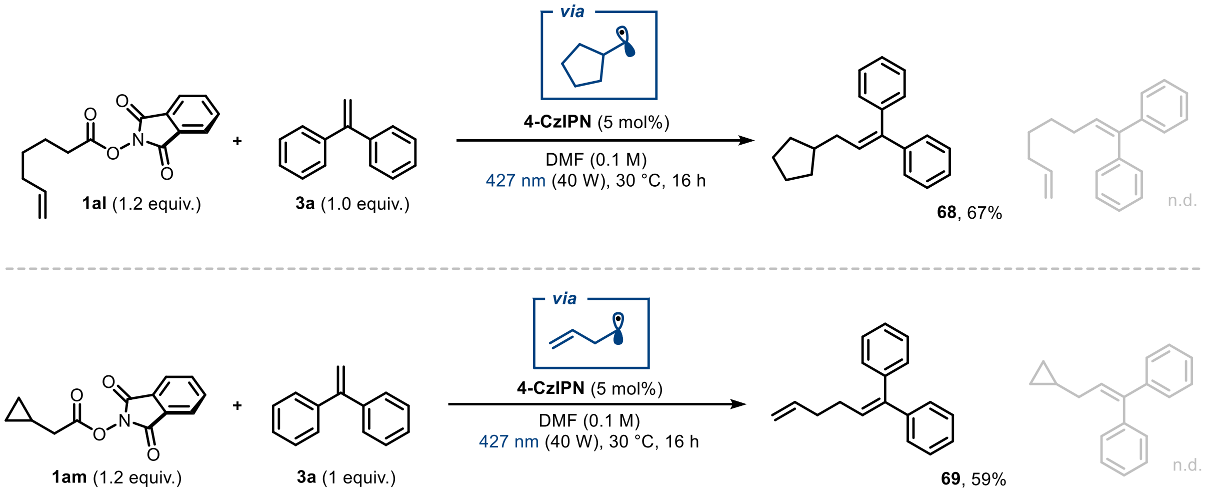

Figure S10: Conditions and results for Heck-type reactions involving radical-clock containing NHPI esters.

Procedure: An oven-dried 20 mL crimp cap vial equipped with a stirring bar was charged with **1al** or **1am** (0.36 mmol, 1.2 equiv.) and **4-CzIPN** (11.7 mg, 5 mol%). After closing the vial, it was evacuated and back-filled with N_2 three times. Analytical grade DMF (3 mL) was added under N_2 atmosphere (0.1 M) and the resulting reaction mixture was bubbled with N_2 for 5 minutes. **3a** (0.3 mmol, 1.0 equiv.) was added via microsyringe through the vial cap. The reaction mixture was stirred and irradiated in the photochemical reactor (*cf.* Section 4) for 16 h. The temperature was maintained around 30 °C during the course of the reaction. Then, the vial was removed from the photochemical reactor and the mixture was added to a separatory funnel containing sat. aq. NaCl solution (10 mL) and ethyl acetate (10 mL). The aqueous layer was extracted with ethyl acetate (3 x 10 mL) and the combined organic phase was washed with sat. aq. NaCl (10 mL) and dried over anhydrous Na_2SO_4. The crude reaction mixture was then concentrated under reduced pressure and purified via flash column chromatography on silica gel. For characterization data of **68** and **69** see Section 10.3.

8.2 Cyclic voltammetry measurements

CV measurements were collected using a Bio-Logic SAS potentiostat (model: SP-200). Glassy carbon was used as working electrode and glassy carbon as counter electrode. Ag/$AgNO_3$ was used as a reference electrode. Tetrabutylammonium tetrafluoroborate (0.1 M, Fluka) was used as supporting electrolyte and ferrocene was used as an external reference for the reduction potentials. Prior to the measurement, the solvent (MeCN or DMF) was degassed with argon (Ar) and all the measurements were performed under Ar atmosphere with a scan rate of 100 mV/s.

Calculations of the potentials and conversion to SCE are done in accordance with literature.

$$E = (E \textit{ vs } Fc^+/Fc + 0.380\text{ V}) \textit{ vs } \text{SCE for MeCN}$$ [99]

$$E = (E \textit{ vs } Fc^+/Fc + 0.470\text{ V}) \textit{ vs } \text{SCE for DMF}$$ [100]

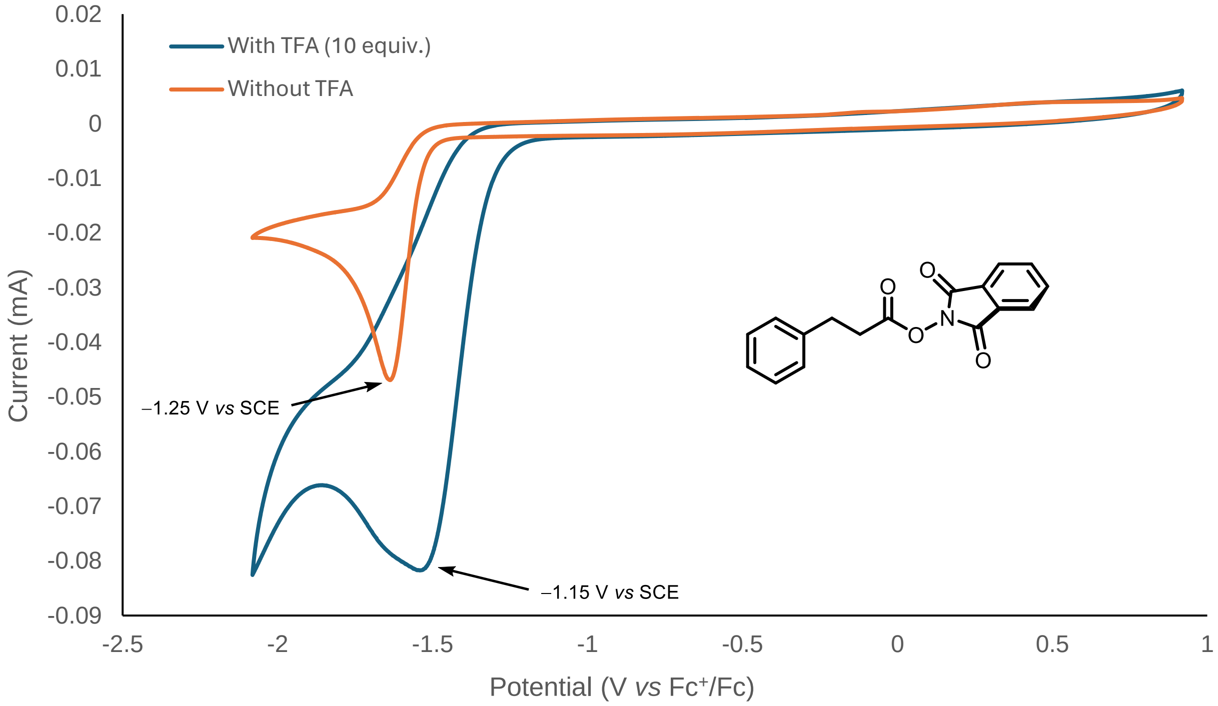

Figure S11: Cyclic voltammetry analysis of **1a** in absence (orange) and presence (blue) of 10 equiv. of TFA.

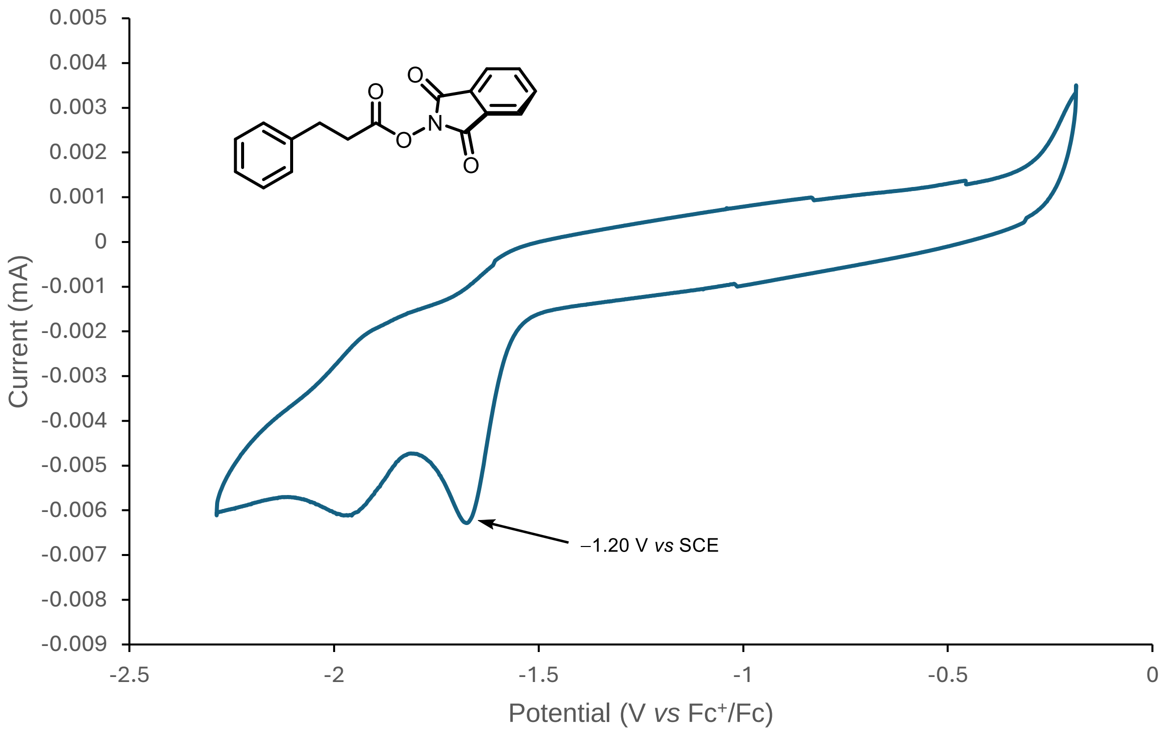

Figure S12: Cyclic voltammetry analysis of **1a** in DMF.

8.3 NMR studies

^{1}H NMR titrations were performed to investigate non-covalent interactions between NHPI esters and 1,1-diarylolefins. Solutions of NHPI ester and DPE were prepared in $CDCl_3$ and MeCN-d_3 (0.6 mL) with varying ratios using 0.1 mol/L as the starting concentration. Using 1,3-dioxoisoindolin-2-yl 3-phenylpropanoate (**1a**) and 1,1-diphenylethylene (**3a**), the following ratios (**Table S12**) were prepared and the comparative ^{1}H NMRs shown in **Figure S13** and **Figure S14**.

Table S12: Ratios of NHPI ester **1a** and 1,1'-diphenylethene **3a** used in NMR titrations

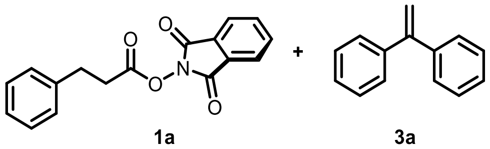

1a **3a**

Entry	**1a**:**3a**
1	1:10
2	1:1
3	10:1
4	**3a**
5	**1a**

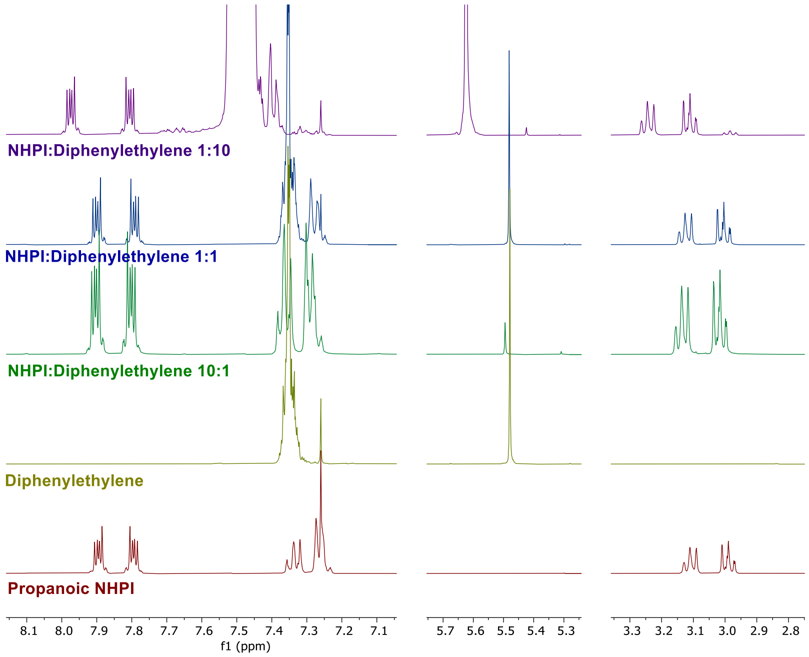

Figure S13: ^{1}H NMR titration between **1a** and **3a** in $CDCl_3$ in the ratios 1:10, 1:1, 10:1, respectively.

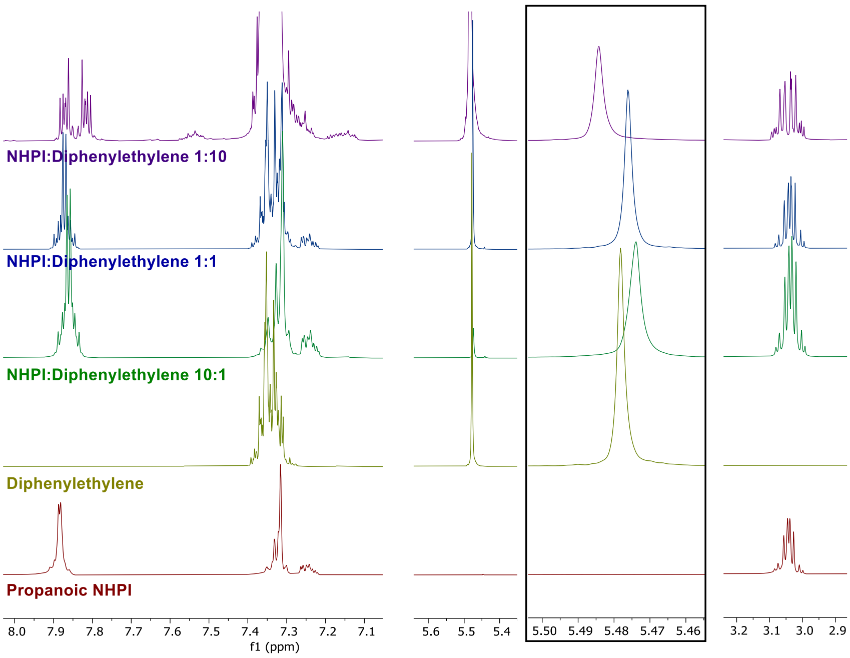

Figure S14: ^{1}H NMR titration between **1a** and **3a** in CD_3CN in the ratios 1:10, 1:1, 10:1, respectively. The spectrum has been magnified between δ 5.46 – 5.50 to show a shift in the protons.

Given the promising initial results obtained in the regard of photocatalyst-free Heck-type reaction between **1d** and **3a** (see Section 5.3), NMR studies were carried out also using **1d** (**Table S13**). The comparative ^{1}H NMRs are shown in **Figure S15** and **Figure S16**.

Table S13: Ratios of NHPI ester **1d** and 1,1'-diphenylethene **3a** used in NMR titrations

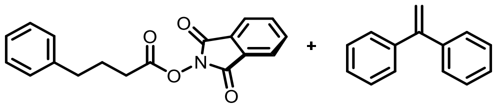

1d **3a**

Entry	**1d**:**3a**
1	1:10
2	1:1
3	10:1
4	**3a**
5	**1d**

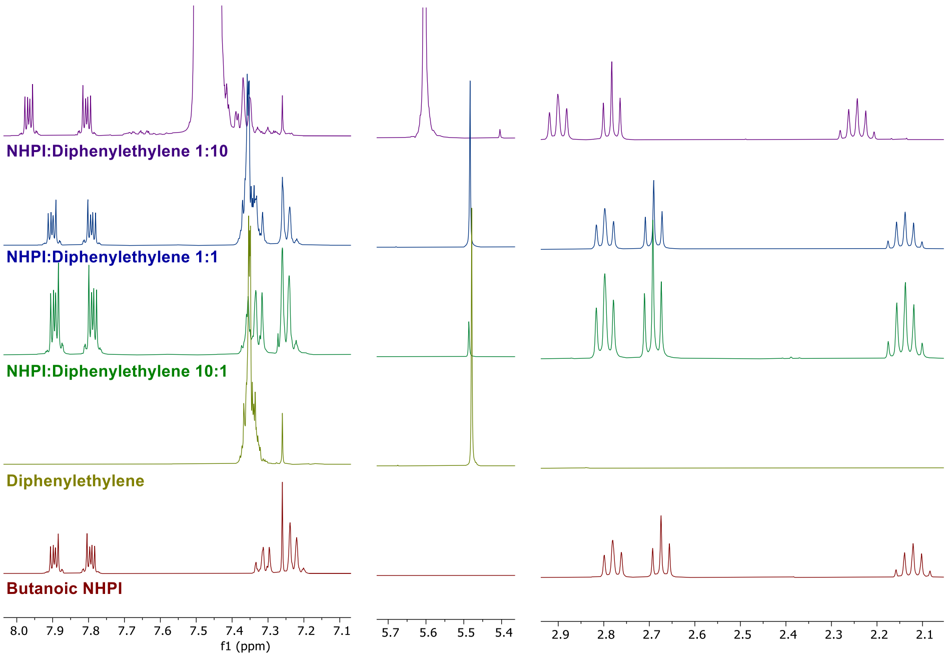

Figure S15: ^{1}H NMR titration between **1d** and **3a** in $CDCl_3$ in the ratios 1:10, 1:1, 10:1, respectively.

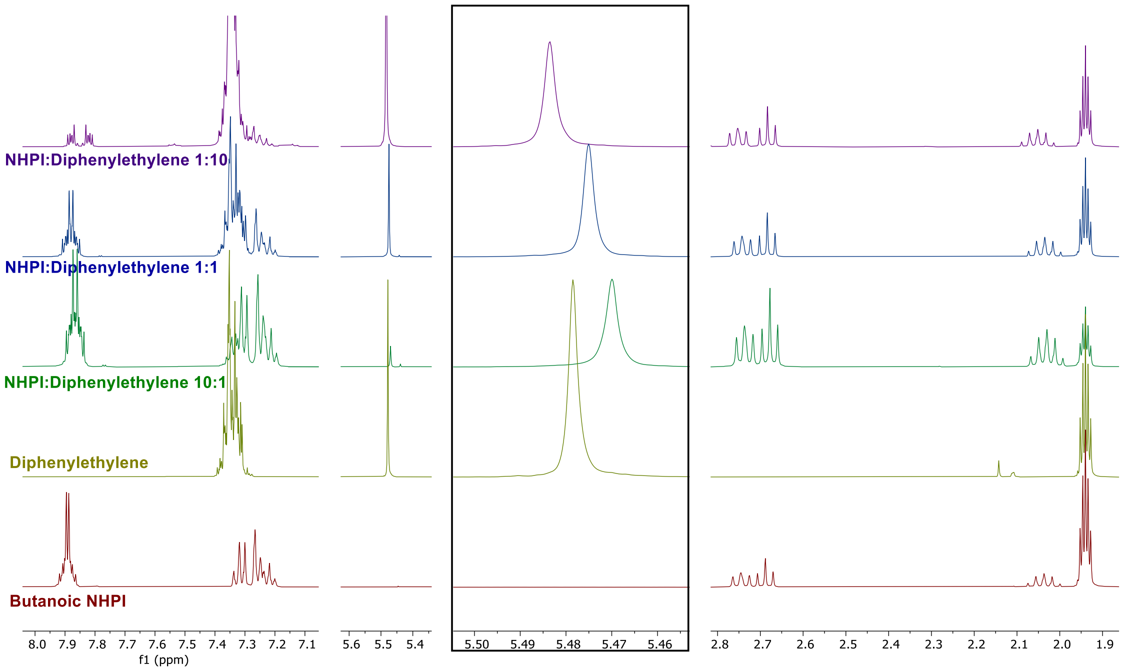

Figure S16: ^{1}H NMR titration between **1d** and **3a** in CD_3CN in the ratios 1:10, 1:1, 10:1, respectively. The spectrum has been magnified between δ 5.46 – 5.50 to show a shift in the protons.

By comparing the obtained ^{1}H NMRs, a change in the chemical shifts of certain protons was observed which was particularly prominent for the CH_2 of the alkenyl group of the 1,1-diphenylethylene (δ 5.48) in both $CDCl_3$ and MeCN-d_3. This downfield shift was more intense in $CDCl_3$ where a shift from δ 5.48 (DPE reference, yellow) to δ 5.62 (**1a**:**3a**, 1:10, purple) was observed in **Figure S13**. A similar shift was apparent in **Figure S15**, from δ 5.48 (DPE reference, yellow) to δ 5.60 (**1d**:**3a**, 1:10, in purple). Consistent but smaller shifts were observed in MeCN-d_3 and are magnified in **Figure S14** and **Figure S16**.

A similar change in the chemical shift of the alkenyl protons due to complexation has been reported by Liu and co-workers (DMSO-d_6).[101] When the NHPI ester was in excess and its loading was increased (green traces), an upfield shift was observed when compared to the reference DPE. Other notable shifts could be observed, such as the downfield shift of NHPI ester aromatic protons (δ 7.7 – 8.1) when DPE is in excess (purple traces) in $CDCl_3$. Interestingly, the splitting pattern for the NHP ester aromatic protons (δ 7.7 – 8.1) changes upon increasing the concentration of DPE in solution.

These observations point to a non-covalent preassembly between NHPI esters and DPEs, that can *i)* rationalize the non-requirement for TFA as an NHPI activator, since the π-stacking assembly may serve for this purpose instead, and *ii)* explain the catalyst-free activity of certain NHPI esters in the Heck reaction, where an electron-donor acceptor (EDA) complex may be formed between the NHPI ester and DPE. This EDA complex may be further stabilized by a secondary π-stacking event from a pendant aryl group on the NHPI ester's alkyl chain, provided the chain length is long enough to establish this interaction (to explain why **1d** / **1e** / **1f** react in this way, while **1a** does not react under catalyst-free conditions).

8.4 Quantum yield experiments

Quantum yield experiments were carried out using the Quantum Yield Determination Set-up (QYDS) shown in **Figure S17**.[102,103] The experiments were conducted within a black box for safety as well as to prevent external light sources influencing the result. A high-power LED (λ = 455 nm, Osram, LD-CQ7P-1U3U) was used as the excitation source. The current for the light source was controlled by a RND 320-KA3005P power supply. A Thorlabs aspheric condenser lens (f = 32 mm, 50 mm diameter) and a Thorlabs plano-convex lens (f = 100 mm, 50 mm diameter) were used. The light bundle was imaged through an aperture (8 mm × 8 mm square) in front of the cuvette holder onto the middle section of the cuvette. A shutter was placed between the lens system and the aperture to interrupt the incoming light beam during the measurement, as needed. The power of light used was detected by a Thorlabs power meter of type S175C.

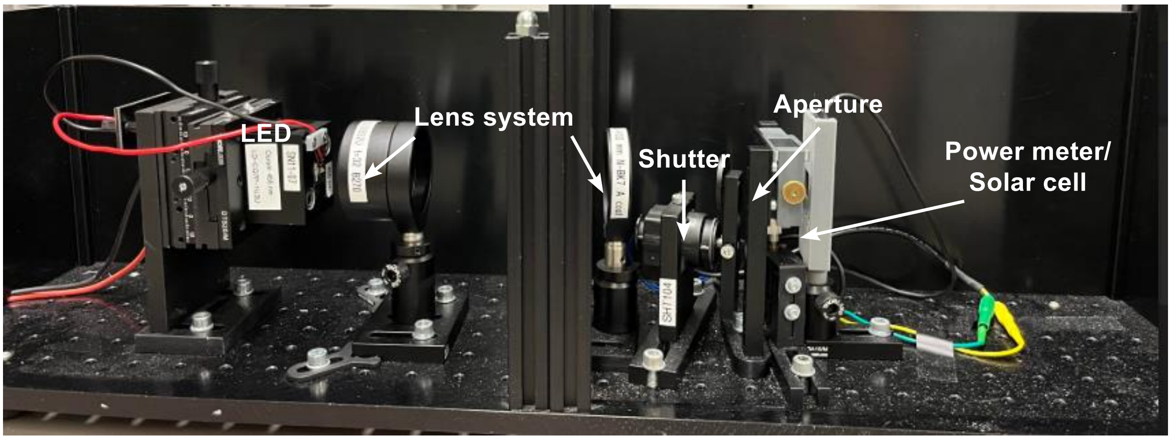

Figure S17: Set-up used in this study for Quantum yields measurement.

Reaction mixtures were prepared in a 10.0 mm x 10.0 mm quartz cuvette with a screw-lid. The volume of the sample solutions was 3 mL and these were equipped with a magnetic stirrer bar for continuous stirring throughout the measurement. Before each experiment, 3 reference power measurements were taken at 30 s intervals, using a cuvette containing the solvent of choice (3 mL), irradiated with the desired input power settings for the experiment, then the average of the 3 measurements was calculated [current = 1.0 A, power (MeCN) = 555 mW, power (DMF) = 737 mW].

As a general procedure, the cuvette was charged with redox active ester and **4-CzIPN** (5 mol%). After sealing the cuvette, N_2 was flushed through for 5 min. Then the solvent (3 mL) was added under N_2 atmosphere (0.1 M) and the resulting reaction mixture was bubbled with N_2 for 5 minutes with stirring. Afterwards, the styrene and TFA (for Ritter reaction) were added via syringe. The cuvette was placed in the QYDS and the shutter was opened. Each sample was irradiated for 3 h (10,800 sec) before being removed from the machine.

For the Ritter carboamidation, the reaction mixture was added to a separatory funnel containing sat. aq. $NaHCO_3$ solution (10 mL) and ethyl acetate (10 mL). The aqueous layer was extracted with ethyl acetate (3 x 10 mL) and the combined organic phase was washed with sat. aq. NaCl (10 mL) and dried over anhydrous Na_2SO_4. The crude reaction mixture was then concentrated under reduced pressure.

For the Heck-type reaction, the reaction mixture was added to a separatory funnel containing sat. aq. NaCl solution (10 mL) and ethyl acetate (10 mL). The aqueous layer was extracted with ethyl acetate (3 x 10 mL) and the combined organic phase was dried over anhydrous Na_2SO_4. The crude reaction mixture was then concentrated under reduced pressure.

The resulting samples (Heck-type and Ritter-type) were diluted with $CDCl_3$ (0.6 mL). CH_2Br_2 (10 μL, 0.1425 mmol) was added and the ^{1}H NMR measured. Using the internal standard (δ 4.94 ppm [s, 2H]), the conversion of starting materials to the desired product was calculated and the quantum yield was then calculated using the equation below:

$$\phi = \frac{N_{prod}}{N_{ph.abs}} = N_A \cdot h \cdot c \frac{c_{prod} \cdot V}{P_{abs} \cdot \Delta t \cdot \lambda_{LED}}$$

Where ϕ = quantum yield, N_A = Avogadro constant (mol^{-1}) , h = Planck's constant (J·s), c = speed of light ($m\ s^{-1}$), c_{prod} = product concentration ($mmol\ L^{-1}$), V = solution volume (mL), P_{abs} = absorbed power (mW), Δt = illumination time (s), λ_{LED} = wavelength (nm).

Calculated ϕ for the Ritter carboamidation was 4.26E-03 (**Figure S18A**) and for the Heck-type reaction was 3.26E-03 (**Figure S18B**). These values are markedly less than 1, which are inconsistent with a radical chain mechanism, although such measurements cannot fully rule out a chain mechanism with an inefficient propagation step. For the Heck process, butanoic NHPI ester **1d** was chosen based on the proposal that the complexation (and plausible chain) may be more prominent in this case, according also to the preliminary results obtained for the photocatalyst-free transformation (for further information, see section 5.3).

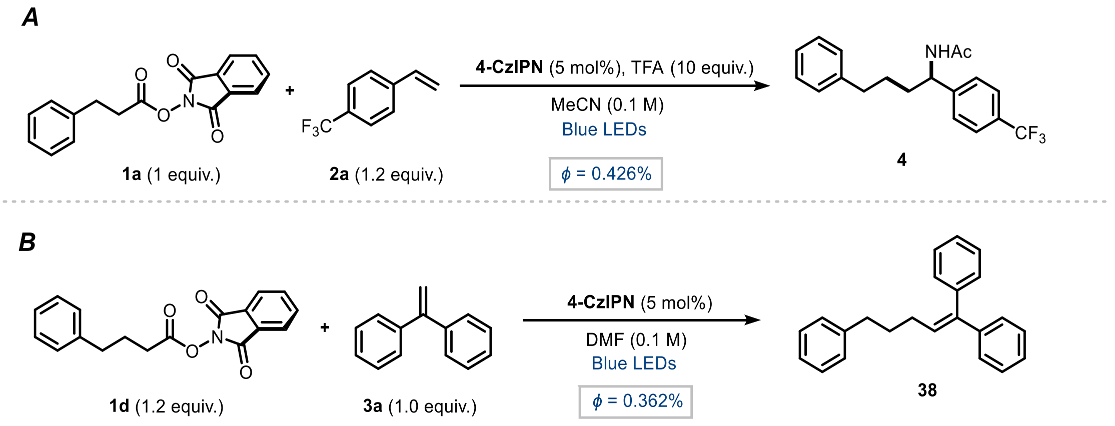

Figure S18: A) Ritter-type reaction quantum yield. B) Heck-type reaction quantum yield.

9. Scale-up procedures

9.1 Scale-up in batch

Ritter-type carboamidation scale-up (4 mmol)

An oven dried 100 mL Schlenk tube, equipped with a magnetic stirring bar, was charged with **1a** (1.18 g, 4 mmol, 1.0 equiv.) and **4-CzIPN** (157 mg, 5 mol%). After closing the tube with a glass cap, it was evacuated and back-filled with N_2 three times. Dry MeCN (40 mL) was added under N_2 atmosphere (0.1 M) through a rubber septum and the resulting reaction mixture was bubbled with N_2 for 5 minutes. **2a** (0.7 mL, 4.8 mmol, 1.2 equiv.) and TFA (3.1 mL, 40 mmol, 10 equiv.) were added via syringe. The reaction mixture was stirred and irradiated using the two Kessil lamps as shown in **Figure S19** (456 nm, 50% light intensity, one with a linear reflector aligned horizontally and one aligned vertically to the flask, 1 cm away from the flask) for 16 h. A high power CPU fan was used to maintain the temperature at 30 °C during the course of the reaction. Then, the vial was removed from the photochemical reactor and the mixture was added to a separatory funnel containing sat. aq. $NaHCO_3$ solution (50 mL) and ethyl acetate (50 mL). The aqueous layer was extracted with ethyl acetate (3 x 50 mL) and the combined organic phase was washed with sat. aq. NaCl (50 mL) and dried over anhydrous Na_2SO_4. The crude reaction mixture was then concentrated under reduced pressure and purified via flash column chromatography on silica gel (from Pentane:Ethyl Acetate 2:1 to 1:1) to afford **4** as a yellowish solid (540 mg, 40% yield).

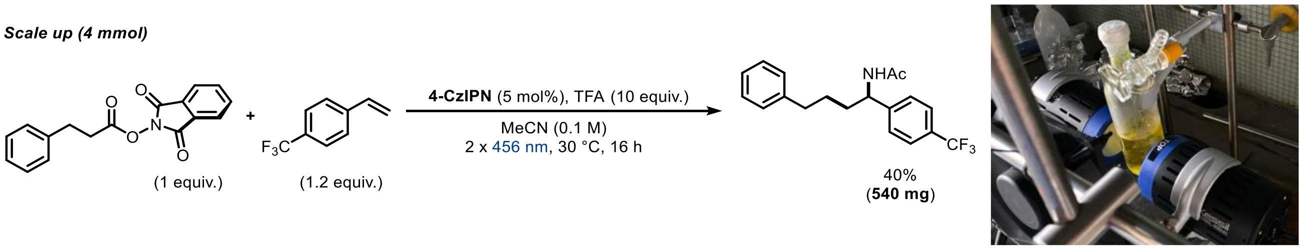

Figure S19: Ritter-type carboamidation setup for a 4 mmol scale reaction.

Heck-type reaction scale-up (1 mmol)

An oven dried 50 mL Schlenk tube, equipped with a magnetic stirring bar, was charged with **1aa** (330 mg, 1.2 mmol, 1.2 equiv.), **3e** (228 mg, 1.0 mmol, 1 equiv.) and **4-CzIPN** (39 mg, 5 mol%). After closing the tube with a glass cap, it was evacuated and back-filled with N_2 three times. Analytical grade DMF (10 mL) was added under N_2 atmosphere (0.1 M) through a rubber septum and the resulting reaction mixture was bubbled with N_2 for 5 minutes. The reaction mixture was stirred and irradiated using a 40 W Kessil lamp as shown in **Figure S20** (427 nm, 100% light intensity, with a linear reflector aligned horizontally to the flask, 1 cm away from the flask) for 16 h. A high power CPU fan was used to maintain the temperature at 30 °C during the course of the reaction. Then, the mixture was added to a separatory funnel containing sat. aq. NaCl solution (30 mL) and ethyl acetate (30 mL). The aqueous layer was extracted with ethyl acetate (3 x 30 mL) and the combined organic phase was washed with sat. aq. NaCl (30 mL) and dried over anhydrous Na_2SO_4. The crude reaction mixture was then concentrated under reduced pressure and purified via flash column

chromatography on silica gel (*n*-Hexane:Ethyl Acetate 10:1) to afford **57** as a white solid (273 mg, 64% yield).

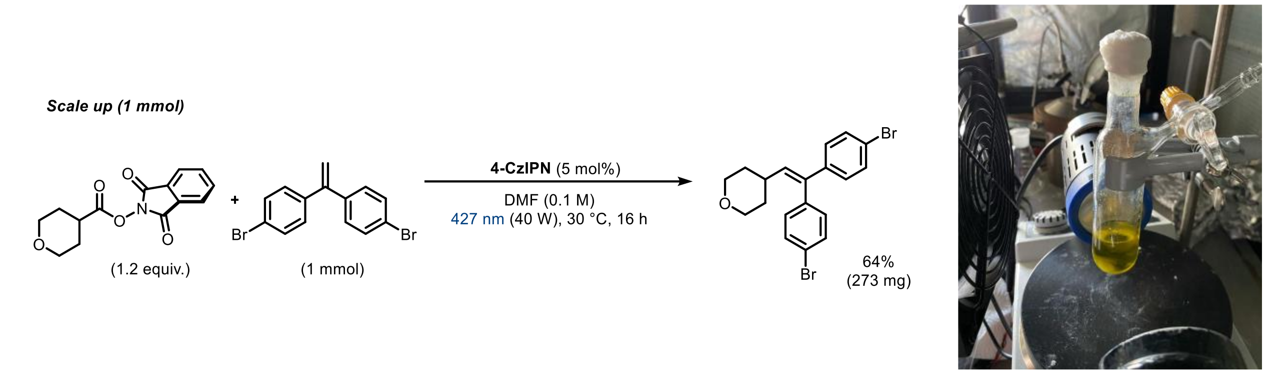

Figure S20: Heck-type reaction setup for a 1 mmol scale reaction.

Heck-type reaction scale-up (4 mmol)

An oven dried 100 mL Schlenk tube, equipped with a magnetic stirring bar, was charged with **1aa** (1.32 g, 4.8 mmol, 1.2 equiv.), **3e** (1.35 g, 1.0 mmol, 1 equiv.) and **4-CzIPN** (157 mg, 5 mol%). After closing the tube with a glass cap, it was evacuated and back-filled with N_2 three times. Analytical grade DMF (40 mL) was added under N_2 atmosphere (0.1 M) through a rubber septum and the resulting reaction mixture was bubbled with N_2 for 5 minutes. The reaction mixture was stirred and irradiated using the two 40 W Kessil lamps as shown in **Figure S21** (456 nm, 50% light intensity, one with a linear reflector aligned horizontally and one aligned vertically to the flask, 1 cm away from the flask) for 16 h. A high power CPU fan was used to maintain the temperature at 30 °C during the course of the reaction. Then, the mixture was added to a separatory funnel containing sat. aq. NaCl solution (50 mL) and ethyl acetate (50 mL). The aqueous layer was extracted with ethyl acetate (3 x 50 mL) and the combined organic phase was washed with sat. aq. NaCl (50 mL) and dried over anhydrous Na_2SO_4. The crude reaction mixture was then concentrated under reduced pressure and purified via flash column chromatography on silica gel (*n*-Hexane:Ethyl Acetate 10:1) to afford **57** as a white solid (950 mg, 57% yield).

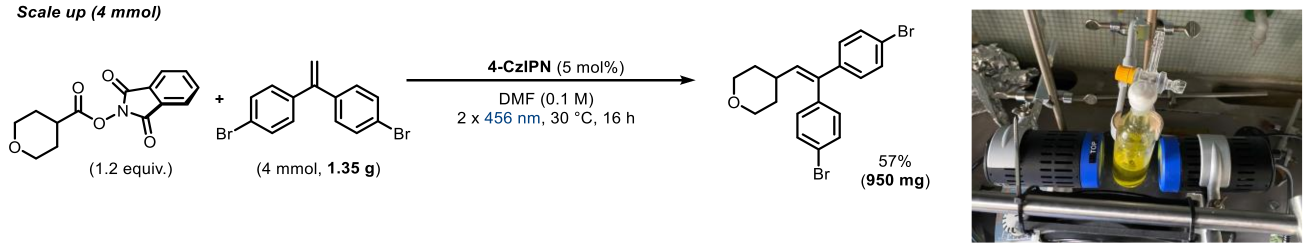

Figure S21: Heck-type reaction setup for 4 mmol scale.

9.2 Scale-up in continuous flow

The reaction coil was irradiated using the photoreactor shown in **Figure S22**. A 40 W Kessil PR160L-456 nm with a linear reflector was used as LED lamp, while the temperature was maintained at 30 – 35 °C via a high power CPU fan positioned under the reactor. All the inside surfaces were covered with aluminum foil. The reactor was designed based on the model reported by Noël and co-workers in the literature.[104]

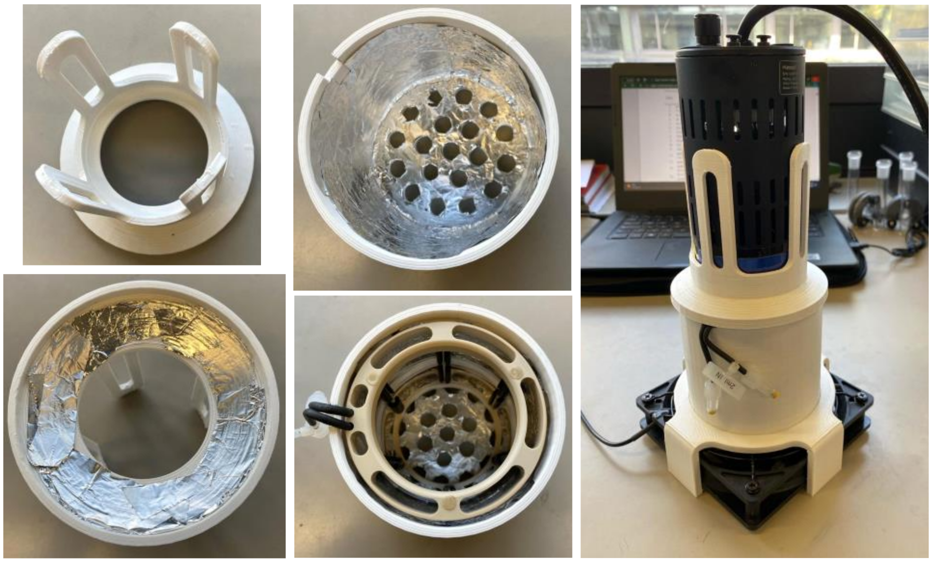

Figure S22: Dissection and assembly of the 3D-printed reactor used for continuous flow experiments.

The complete flow setup consists of an SF-10 slurry-capable peristaltic pump of the Vapourtec UV-150 R-series as the dosing unit and the 3D-printed photochemical reactor with the reaction coil, the Kessil lamp and the high-power fan (**Figure S23**).

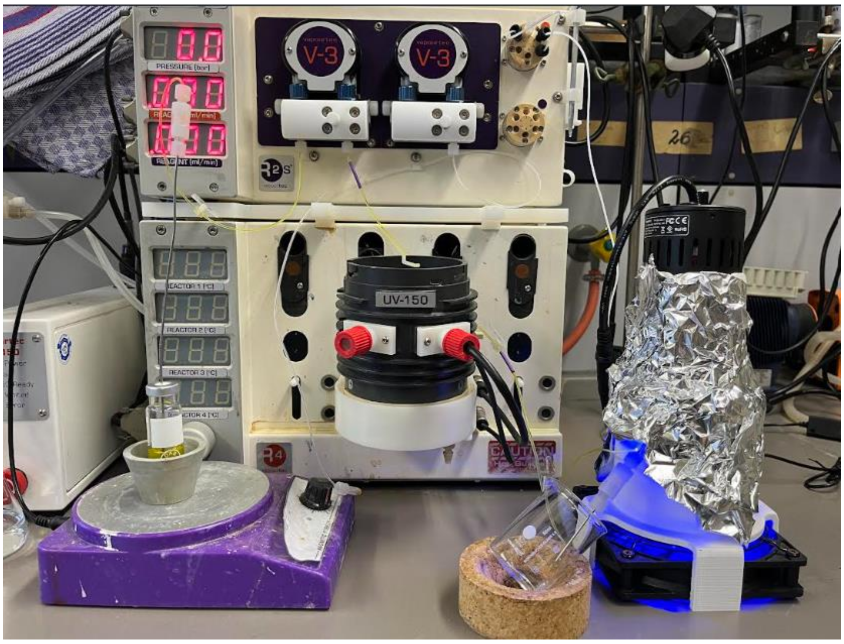

Figure S23: Continuous flow photochemical setup.

Ritter-type carboamidation scale-up in continuous flow (1 mmol scale)

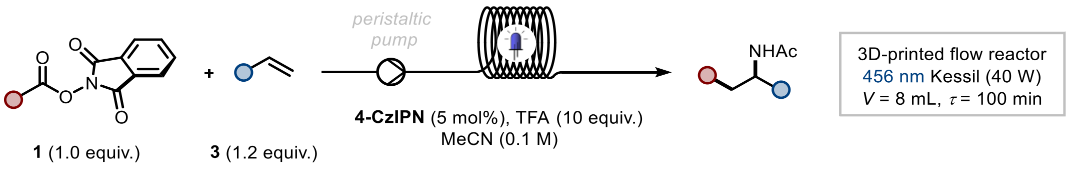

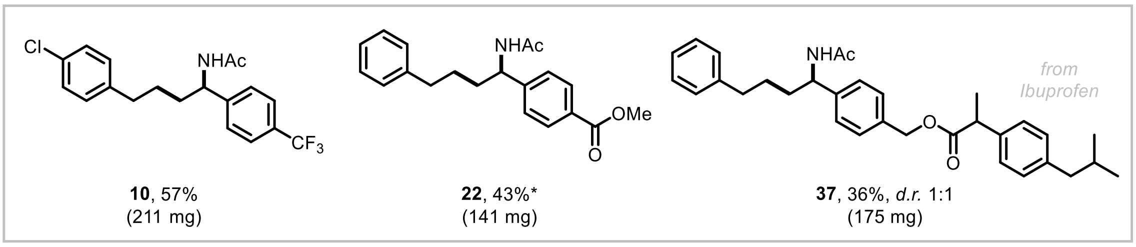

Figure S24: Conditions, product structures and yields of Ritter-type carboamidations in flow. *See note below.

An oven-dried 20 mL crimp cap vial, equipped with a magnetic stirring bar, was charged with redox active ester (1.0 mmol, 1.0 equiv.), styrene (1.2 mmol, 1.2 equiv., *if solid*) and **4-CzIPN** (39.4 mg, 5 mol%). After closing the vial, it was evacuated and back-filled with N_2 three times. Dry MeCN (10 mL) was added under N_2 atmosphere (0.1 M) and the resulting reaction mixture was bubbled with N_2 for 5 minutes. Afterwards, styrene (1.2 mmol, 1.2 equiv., *if liquid*) and TFA (10 mmol, 10 equiv.) were added via syringe. The Kessil lamp (456 nm, 100% light intensity) was turned on and a high power CPU fan was used to maintain the temperature at 30 °C during the course of the reaction. The reaction solution was pumped through the photoreactor (internal volume = 8 mL) with a flow rate of 0.08 mL/min (residence time τ = 100 min). After steady-state was reached, the outflow solution was collected into a glass vial. Then, the resulting mixture was added to a separatory funnel containing sat. aq. $NaHCO_3$ solution (20 mL) and ethyl acetate (20 mL). The aqueous layer was extracted with ethyl acetate (3 x 20 mL) and the combined organic phase was washed with sat. aq. NaCl (20 mL) and dried over anhydrous Na_2SO_4. The crude reaction mixture was then concentrated under reduced pressure and purified via flash column chromatography on silica gel (Pentane:Ethyl Acetate) to afford the desired products **10**, **22** and **37**.

*A note should be added for the compound **22**: incomplete solubilization of methyl 4-vinylbenzoate was observed both prior to and during the reaction. Although this did not result in any issues with physical processability of the flow (*i.e.* no clogging observed), it likely resulted in a loss in the yield. The batch reaction (0.3 mmol, 16 h) exhibited the same issue, but after approximately 4 hours, all the olefin was solubilized in the reaction media.

Heck-type reaction scale-up in continuous flow (4 mmol)

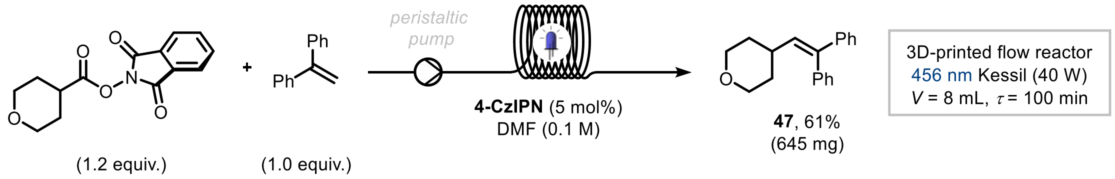

Figure S25: Conditions, product structure and yield of the Heck-type reaction in continuous flow.

An oven dried 100 mL round bottom flask, equipped with a magnetic stirring bar, was charged with **1aa** (1.32 g, 4.8 mmol, 1.2 equiv.) and **4-CzIPN** (157.7 mg, 5 mol%). After sealing the flask, it was evacuated and back-filled with N_2 three times. Analytical grade DMF (40 mL) was added under N_2 atmosphere (0.1 M) and the resulting reaction mixture was bubbled with N_2 for 10 minutes. Afterwards, **3a** (0.846 mL, 4.0 mmol, 1.0 equiv.) was added via syringe. The Kessil lamp (456 nm, 100% light intensity) was turned on and used with a high power CPU fan to maintain the temperature at 30 °C during the course of the reaction. The reaction solution was pumped through the photoreactor (internal volume = 8 mL) with a flow rate of 0.08 mL/min (residence time τ = 100 min). After steady-state was reached, the outflow solution was collected into a round bottom flask. Then, the resulting mixture was added to a separatory funnel containing sat. aq. NaCl solution (40 mL) and ethyl acetate (40 mL). The aqueous layer was extracted with ethyl acetate (3 x 40 mL). The combined organic phase was washed with further sat. aq. NaCl solution (40 mL). The organics were collected and dried over anhydrous Na_2SO_4. The crude reaction mixture was then concentrated under reduced pressure and purified via flash column chromatography on silica gel (Pentane:Ethyl Acetate 20:1) to afford **47** as a white solid (645 mg, 61%).

9.3 Space time yields

The Space Time Yield (STY) was calculated using the following equation:[105]

$$STY = \frac{product\ (mmol)}{reactor\ volume\ (L) \times operation\ time(h)}$$

Where units have been converted to the following: product moles (mmol), reactor volume (L) and operation time (h). For the continuous flow experiments, only the reaction volume of the coil exposed to light irradiation was used, not the length of tubing of the entire flow setup. STYs were evaluated in both batch and flow for different reaction scales for the substrates shown below (**Figure S26**).

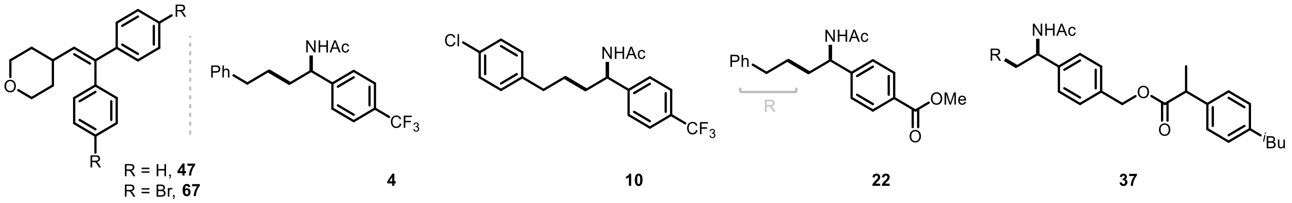

R = H, **47**
R = Br, **67**

4 **10** **22** **37**

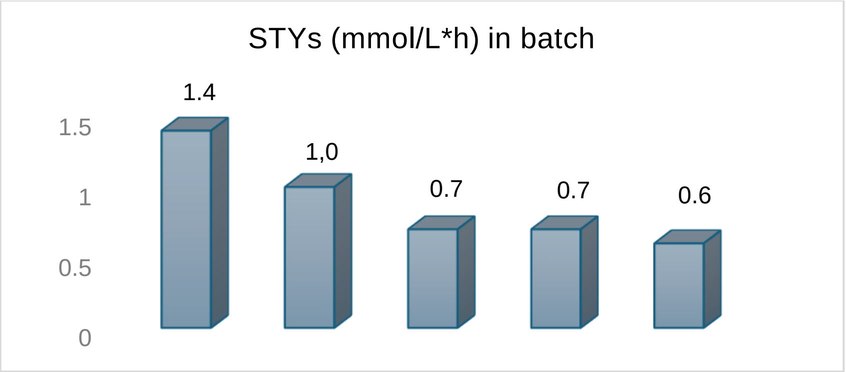

product	67	4	10	22	37
scale	4 mmol	4 mmol	0.3 mmol	0.3 mmol	0.3 mmol

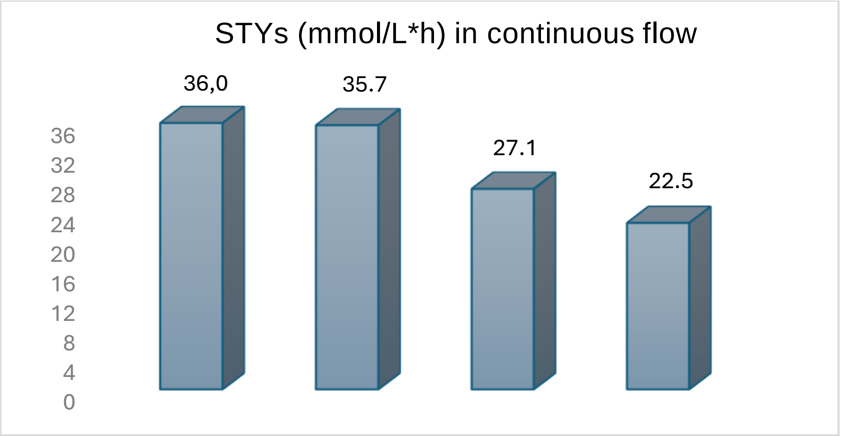

product	47	10	22	37
scale	4 mmol	1.0 mmol	1.0 mmol	1.0 mmol

Figure S26: Space-time-yields comparison for batch and flow reactions of selected Ritter-type or Heck-type products.

10. Characterization data of synthesized compounds

10.1 Characterization data of NHPI esters

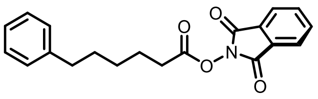

***1,3-Dioxoisoindolin-2-yl 6-phenylhexanoate* (1f).** Prepared according to GP1 from 6-phenylhexanoic acid (150 mg, 1.29 mmol). Purified via flash column chromatography on silica gel (Pentane:Ethyl Acetate 5:1) to afford the product as a white solid (380 mg, 87% yield).

^{1}H NMR (400 MHz, $CDCl_3$) δ 7.89 (dd, J = 5.5, 3.1 Hz, 2H), 7.79 (dd, J = 5.5, 3.1 Hz, 2H), 7.29 – 7.26 (m, 2H), 7.20 – 7.16 (m, 3H), 2.66 (q, J = 7.3 Hz, 4H), 1.88 – 1.77 (m, 2H), 1.75 – 1.65 (m, 2H), 1.55 – 1.42 (m, 2H).
^{13}C NMR (101 MHz, $CDCl_3$) δ 169.7, 162.1, 142.5, 134.9, 129.1, 128.5, 128.4, 125.9, 124.1, 35.8, 31.1, 31.0, 28.6, 24.7.
HRMS (ESI+) m/z: $[M+Na]^+$ calcd. for $C_{20}H_{19}NO_4$ 360.1206, found 360.1208.

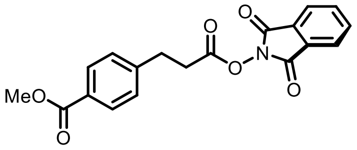

***Methyl 4-(3-((1,3-dioxoisoindolin-2-yl)oxy)-3-oxopropyl)benzoate* (1i).** Prepared according to GP1 from 3-(4-(methoxycarbonyl)phenyl)propanoic acid (500 mg, 2.4 mmol) to afford the product as a white solid (620 mg, 73% yield).

^{1}H NMR (400 MHz, $CDCl_3$) δ 8.01 (d, *J* = 8.3 Hz, 2H), 7.89 (dd, *J* = 5.5, 3.1 Hz, 2H), 7.79 (dd, *J* = 5.5, 3.1 Hz, 2H), 7.34 (d, *J* = 7.9 Hz, 2H), 3.91 (s, 3H), 3.16 (t, *J* = 7.7 Hz, 2H), 3.01 (t, *J* = 7.5 Hz, 2H).
^{13}C NMR (101 MHz, $CDCl_3$) δ 168.8, 167.1, 162.0, 144.6, 135.0, 130.2, 129.0, 128.9, 128.5, 124.2, 52.2, 32.4, 30.6.
HRMS (ESI+) m/z: $[M+Na]^+$ calcd. for $C_{19}H_{15}NO_6$ 376.0792, found 376.0793.

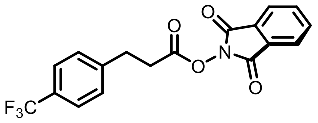

***1,3-Dioxoisoindolin-2-yl 3-(4-(trifluoromethyl)phenyl)propanoate* (1j).** Prepared according to GP1 from 3-(4-(trifluoromethyl)phenyl)propanoic acid (436 mg, 2 mmol) to afford the product as a white solid (639 mg, 88% yield).

^{1}H NMR (400 MHz, $CDCl_3$) δ 7.89 (dd, *J* = 5.5, 3.1 Hz, 2H), 7.79 (dd, *J* = 5.5, 3.1 Hz, 2H), 7.59 (d, *J* = 8.0 Hz, 2H), 7.39 (d, *J* = 8.0 Hz, 2H), 3.16 (t, *J* = 7.6 Hz, 2H), 3.01 (t, *J* = 7.6 Hz, 2H).
^{13}C NMR (101 MHz, $CDCl_3$) δ 168.7, 162.0, 143.3, 135.0, 129.2 (q, *J* = 32.5 Hz), 129.0, 128.9, 125.8 (q, *J* = 3.8 Hz), 124.2, 121.6 (d, *J* = 272.0 Hz), 32.4, 30.4.

^{19}F NMR (376 MHz, $CDCl_3$) δ −62.97.
HRMS (ESI+) m/z: $[M+Na]^+$ calcd. for $C_{18}H_{12}F_3NO_4$ 386.0611, found 386.0607.

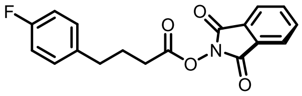

1,3-Dioxoisoindolin-2-yl 4-(4-fluorophenyl)butanoate **(1m)*.*** Prepared according to GP1 from 4-(4-fluorophenyl)butanoic acid (400 mg, 2.2 mmol). Purified via flash column chromatography on silica gel (Pentane:Ethyl Acetate 4:1) to afford the product as a white solid (632 mg, 88% yield).

^{1}H NMR (400 MHz, $CDCl_3$) δ 7.90 – 7.87 (dd, *J* = 5.5, 3.1 Hz, 2H), 7.79 (dd, *J* = 5.5, 3.1 Hz, 2H), 7.18 (dd, *J* = 8.5, 5.5 Hz, 2H), 6.99 (t, *J* = 8.7 Hz, 2H), 2.75 (t, *J* = 7.6 Hz, 2H), 2.66 (t, *J* = 7.3 Hz, 2H), 2.08 (dq, *J* = 8.6, 7.3 Hz, 2H).
^{13}C NMR (101 MHz, $CDCl_3$) δ 169.5, 162.1, 161.6 (d, *J* = 245 Hz), 136.4 (d, *J* = 3.2 Hz), 134.9, 130.1 (d, *J* = 7.8 Hz), 129.0, 124.1, 115.4 (d, *J* = 21.2 Hz), 33.8, 30.2, 26.5.
^{19}F NMR (376 MHz, $CDCl_3$) δ −117.63.
HRMS (ESI+) m/z: $[M+Na]^+$ calcd. for $C_{18}H_{14}FNO_4$ 350.0799, found 350.0804.

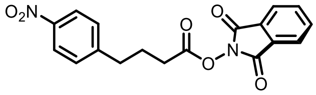

1,3-Dioxoisoindolin-2-yl 4-(4-nitrophenyl)butanoate **(1n)*.*** Prepared according to GP1 from 4-(4-nitrophenyl)butanoic acid (400 mg, 1.9 mmol). Purified via flash column chromatography on silica gel (Pentane:Ethyl Acetate 1:1) to afford the product as a white solid (670 mg, 76% yield).

^{1}H NMR (400 MHz, $CDCl_3$) δ 8.17 (d, *J* = 8.7 Hz, 2H), 7.89 (dd, *J* = 5.5, 3.1 Hz, 2H), 7.80 (dd, *J* = 5.5, 3.1 Hz, 2H), 7.40 (d, *J* = 8.6 Hz, 2H), 2.93 – 2.85 (t, *J* = 8.6 Hz, 2H), 2.69 (t, *J* = 7.1 Hz, 2H), 2.14 (dq, *J* = 9.1, 7.2 Hz, 2H).
^{13}C NMR (101 MHz, $CDCl_3$) δ 169.2, 162.1, 148.6, 146.8, 135.0, 129.5, 129.0, 124.1, 124.0, 34.4, 30.3, 26.0.
HRMS (ESI+) m/z: $[M+H]^+$ calcd. for $C_{18}H_{14}N_2O_6$ 355.0930, found 355.0927.

10.2 Characterization data of Ritter-type carboamidation products

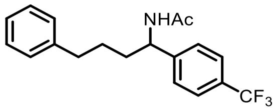

N-(4-phenyl-1-(4-(trifluoromethyl)phenyl)butyl)acetamide **(4)*.*** Prepared according to GP3 from **1a** (89 mg, 0.3 mmol, 1.0 equiv.) and **2a** (54 µL, 0.36 mmol, 1.2 equiv.). Purified via flash column chromatography on silica gel (from Pentane:Ethyl Acetate 2:1 to 1:1) to afford the product as a yellowish solid (48 mg, 48% yield).

^{1}H NMR (400 MHz, $CDCl_3$) δ 7.57 (d, *J* = 8.0 Hz, 2H), 7.35 (d, *J* = 8.0 Hz, 2H), 7.30 – 7.25 (m, 2H), 7.19 (m, 1H), 7.18 – 7.08 (m, 2H), 5.73 (d, *J* = 8.1 Hz, 1H), 5.02 (q, *J* = 7.6 Hz, 1H), 2.63 (td, *J* = 7.4, 3.5 Hz, 2H), 1.97 (s, 3H), 1.86 – 1.73 (m, 2H), 1.73 – 1.61 (m, 1H), 1.64 – 1.52 (m, 1H).
^{13}C NMR (151 MHz, $CDCl_3$) δ 169.5, 146.5, 141.7, 129.7 (q, *J* = 32.5 Hz), 128.6, 128.5, 127.0, 126.1, 125.8 (q, *J* = 3.7 Hz), 124.2 (q, *J* = 272.0 Hz), 53.2, 35.5, 35.5, 28.0, 23.5.
^{19}F NMR (376 MHz, $CDCl_3$) δ −63.03.
HRMS (ESI+) m/z $[M+H]^+$ calcd. for $C_{19}H_{20}F_3NO$ 336.1570 , found 336.1573.

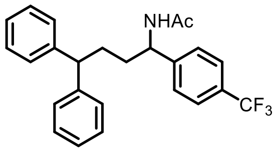

N-(4,4-diphenyl-1-(4-(trifluoromethyl)phenyl)butyl)acetamide **(5)*.*** Prepared according to GP3 from **1b** (111 mg, 0.3 mmol, 1.0 equiv.) and **2a** (54 µL, 0.36 mmol, 1.2 equiv.). Purified via flash column chromatography on silica gel (from Pentane:Ethyl Acetate 2:1 to 1:1) to afford the product as a yellowish solid (42 mg, 34% yield).

^{1}H NMR (400 MHz, $CDCl_3$) δ 7.56 (d, *J* = 8.1 Hz, 2H), 7.32 – 7.25 (m, 6H), 7.18 (dd, *J* = 9.1, 7.1 Hz, 6H), 5.67 (d, *J* = 8.1 Hz, 1H), 5.02 (q, *J* = 7.6 Hz, 1H), 3.88 (t, *J* = 7.8 Hz, 1H), 2.17 – 2.04 (m, 1H), 2.01 – 1.96 (m, 1H), 1.95 (s, 3H), 1.79 – 1.71 (m, 2H).
^{13}C NMR (151 MHz, $CDCl_3$) δ 169.4, 146.3, 144.4, 144.4, 129.8 (q, *J* = 32.3 Hz), 128.7, 128.7, 127.9, 127.1, 126.5, 126.5, 125.8 (q, *J* = 3.8 Hz), 124.2 (q, *J* = 272.2 Hz), 53.3, 51.2, 34.5, 32.2, 23.5.
^{19}F NMR (376 MHz, $CDCl_3$) δ −63.02.
HRMS (ESI+) m/z $[M+H]^+$ calcd. for $C_{25}H_{24}F_3NO$ 412.1883, found 412.1891.

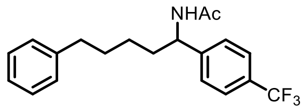

N-(5-phenyl-1-(4-(trifluoromethyl)phenyl)pentyl)acetamide **(6)*.*** Prepared according to GP3 from **1d** (93 mg, 0.3 mmol, 1.0 equiv.) and **2a** (54 µL, 0.36 mmol, 1.2 equiv.). Purified via flash column chromatography on silica gel (from Pentane:Ethyl Acetate 2:1 to 1:1) to afford the product as a yellowish solid (43 mg, 42% yield).

^{1}H NMR (400 MHz, $CDCl_3$) δ 7.56 (d, *J* = 8.1 Hz, 2H), 7.35 (d, *J* = 8.0 Hz, 2H), 7.28 (d, *J* = 1.0 Hz, 2H), 7.21 – 7.16 (m, 1H), 7.15 – 7.08 (m, 2H), 5.95 (d, *J* = 8.1 Hz, 1H), 4.96 (q, *J* = 7.6 Hz, 1H), 2.57 (t, *J* = 7.6 Hz, 2H), 1.96 (s, 3H), 1.85 – 1.73 (m, 2H), 1.69 – 1.57 (m, 2H), 1.40 – 1.26 (m, 2H).
^{13}C NMR (151 MHz, $CDCl_3$) δ 169.7, 146.7, 142.2, 129.5 (q, *J* = 32.4 Hz), 128.5, 128.4, 127.0, 125.9, 125.7 (q, *J* = 3.8 Hz), 124.2 (q, *J* = 271.9 Hz), 53.3, 36.0, 35.7, 31.1, 25.7, 23.3.
^{19}F NMR (376 MHz, $CDCl_3$) δ −62.98.
HRMS (ESI+) m/z $[M+H]^+$ calcd. for $C_{20}H_{22}F_3NO$ 350.1726, found 350.1729.

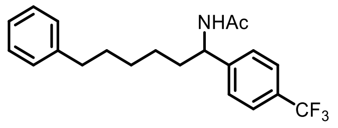

N-(6-phenyl-1-(4-(trifluoromethyl)phenyl)hexyl)acetamide **(7).** Prepared according to GP3 from **1e** (97 mg, 0.3 mmol, 1.0 equiv.) and **2a** (54 µL, 0.36 mmol, 1.2 equiv.). Purified via flash column chromatography on silica gel (from Pentane:Ethyl Acetate 2:1 to 1:1) to afford the product as a yellowish solid (49 mg, 45% yield).

^{1}H NMR (400 MHz, $CDCl_3$) δ 7.58 (d, *J* = 8.1 Hz, 2H), 7.37 (d, *J* = 8.0 Hz, 2H), 7.29 – 7.23 (m, 2H), 7.20 – 7.12 (m, 3H), 5.74 (d, *J* = 8.0 Hz, 1H), 4.96 (q, *J* = 7.6 Hz, 1H), 2.58 (t, *J* = 7.6 Hz, 2H), 1.99 (s, 3H), 1.81 – 1.71 (m, 2H), 1.66 – 1.53 (m, 2H), 1.40 – 1.22 (m, 4H).
^{13}C NMR (151 MHz, $CDCl_3$) δ 169.5, 146.7, 142.6, 129.7 (q, *J* = 32.4 Hz), 128.5, 128.4, 127.0, 125.8, 125.7 (q, *J* = 3.8 Hz), 124.2 (q, *J* = 272.0 Hz), 53.4, 36.2, 35.9, 31.3, 29.0, 26.2, 23.5.
^{19}F NMR (376 MHz, $CDCl_3$) δ −63.01.
HRMS (ESI+) m/z $[M+H]^+$ calcd. for $C_{21}H_{24}F_3NO$ 364.1883, found 364.1888.

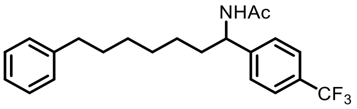

N-(7-phenyl-1-(4-(trifluoromethyl)phenyl)heptyl)acetamide **(8).** Prepared according to GP3 from **1f** (101 mg, 0.3 mmol, 1.0 equiv.) and **2a** (54 µL, 0.36 mmol, 1.2 equiv.). Purified via flash column chromatography on silica gel (from Pentane:Ethyl Acetate 2:1 to 1:1) to afford the product as a yellowish solid (58 mg, 51% yield).

^{1}H NMR (400 MHz, $CDCl_3$) δ 7.58 (d, *J* = 8.1 Hz, 2H), 7.37 (d, *J* = 8.2 Hz, 2H), 7.29 – 7.24 (m, 2H), 7.20 – 7.13 (m, 3H), 5.81 (d, *J* = 8.0 Hz, 1H), 4.97 (q, *J* = 7.6 Hz, 1H), 2.58 (t, *J* = 7.6 Hz, 2H), 1.99 (s, 3H), 1.78 – 1.72 (m, 2H), 1.66 – 1.54 (m, 2H), 1.36 – 1.28 (m, 6H).
^{13}C NMR (151 MHz, $CDCl_3$) δ 169.6, 146.7, 142.8, 129.6 (q, *J* = 32.5 Hz), 128.5, 128.4, 127.0, 125.7 (q, *J* = 3.8 Hz), 125.7, 124.2 (q, *J* = 271.8 Hz), 53.4, 36.2, 36.0, 31.4, 29.3, 29.1, 26.2, 23.5.
^{19}F NMR (376 MHz, $CDCl_3$) δ −62.99.
HRMS (ESI+) m/z $[M+H]^+$ calcd. for $C_{22}H_{27}F_3NO$ 378.2039, found 378.2044.

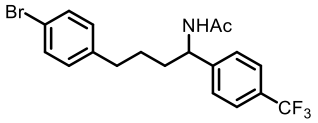

N*-(4-(4-bromophenyl)-1-(4-(trifluoromethyl)phenyl)butyl)acetamide** **(9)*. Prepared according to GP3 from **1g** (112 mg, 0.3 mmol, 1.0 equiv.) and **2a** (54 µL, 0.36 mmol, 1.2 equiv.). Purified via flash column chromatography on silica gel (Pentane:Ethyl Acetate 1:1) to afford the product as a white solid (80 mg, 65% yield).

^{1}H NMR (400 MHz, $CDCl_3$) δ 7.58 (d, J = 8.0 Hz, 2H), 7.38 (d, J = 8.3 Hz, 2H), 7.35 (d, J = 8.5 Hz, 2H), 6.99 (d, J = 8.1 Hz, 2H), 5.70 (d, J = 8.2 Hz, 1H), 5.01 (q, J = 7.6 Hz, 1H), 2.58 (td, J = 7.4, 3.4 Hz, 2H), 1.98 (s, 3H), 1.82 – 1.74 (m, 2H), 1.70 – 1.63 (m, 1H), 1.60 – 1.50 (m, 1H).
^{13}C NMR (151 MHz, $CDCl_3$) δ 169.5, 146.3, 140.6, 131.6, 130.3, 130.0 (t, J = 32.4 Hz), 127.0, 125.9 (q, J = 3.7 Hz), 124.2 (q, J = 272.1 Hz), 119.9, 53.1, 35.4, 34.9, 27.8, 23.5.
^{19}F NMR (376 MHz, $CDCl_3$) δ −63.05.
HRMS (EI+) m/z $[M]^+$ calcd. for $C_{19}H_{19}{}^{79}BrF_3NO$ 413.0597, found 413.0592.

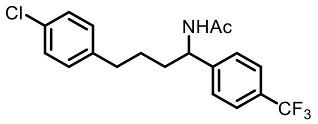

N*-(4-(4-chlorophenyl)-1-(4-(trifluoromethyl)phenyl)butyl)acetamide** **(10)*. Prepared according to GP3 from **1h** (99 mg, 0.3 mmol, 1.0 equiv.) and **2a** (54 µL, 0.36 mmol, 1.2 equiv.). Purified via flash column chromatography on silica gel (Pentane:Ethyl Acetate 1:1) to afford the product as a yellowish solid (74 mg, 67% yield).

^{1}H NMR (400 MHz, $CDCl_3$) δ 7.57 (d, J = 8.1 Hz, 2H), 7.35 (d, J = 8.0 Hz, 2H), 7.23 (d, J = 8.4 Hz, 2H), 7.05 (d, J = 8.3 Hz, 2H), 5.78 (d, J = 8.5 Hz, 1H), 5.01 (q, J = 7.6 Hz, 1H), 2.60 (td, J = 7.4, 3.3 Hz, 2H), 1.97 (s, 3H), 1.81 – 1.74 (m, 2H), 1.70 – 1.61 (m, 1H), 1.57 – 1.52 (m, 1H).
^{13}C NMR (151 MHz, $CDCl_3$) δ 169.5, 146.3, 140.1, 131.9, 130.1 (q, J = 32.5 Hz), 129.9, 128.7, 127.0, 125.9 (q, J = 3.8 Hz), 124.2 (q, J = 272.1 Hz), 53.1, 35.4, 34.8, 27.9, 23.5.
^{19}F NMR (376 MHz, $CDCl_3$) δ −63.05.
HRMS (EI+) m/z $[M]^+$ calcd. for $C_{19}H_{19}{}^{35}ClF_3NO$ 369.1102, found 369.1095.

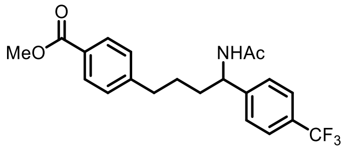

Methyl 4-(4-acetamido-4-(4-(trifluoromethyl)phenyl)butyl)benzoate **(11)*.*** Prepared according to GP3 from **1i** (106 mg, 0.3 mmol, 1.0 equiv.) and **2a** (54 µL, 0.36 mmol, 1.2 equiv.). Purified via flash column chromatography on silica gel (Pentane:Ethyl Acetate 1:1) to afford the product as a white solid (63 mg, 53% yield).

^{1}H NMR (400 MHz, $CDCl_3$) δ 7.94 (d, J = 8.0 Hz, 2H), 7.57 (d, J = 8.0 Hz, 2H), 7.35 (d, J = 8.0 Hz, 2H), 7.19 (d, J = 8.0 Hz, 2H), 5.71 (d, J = 8.2 Hz, 1H), 5.03 (q, J = 7.6 Hz, 1H), 3.90 (s, 3H), 2.68 (dt, J = 7.1, 3.8 Hz, 2H), 1.98 (s, 3H), 1.84 – 1.76 (m, 2H), 1.73 – 1.66 (m, 1H), 1.61 – 1.54 (m, 1H).

^{13}**C NMR** (151 MHz, $CDCl_3$) δ 169.5, 167.2, 147.2, 146.3, 129.9, 129.7 (q, *J* = 32.4 Hz), 128.6, 128.2, 127.02, 125.9 (q, *J* = 3.8 Hz), 124.2 (q, *J* = 272.1 Hz), 53.1, 52.2, 35.5, 35.5, 27.6, 23.5.
^{19}**F NMR** (376 MHz, $CDCl_3$) δ −63.07.
HRMS (ESI+) m/z $[M+H]^+$ calcd. for $C_{21}H_{22}F_3NO_3$ 394.1625, found 394.1628.

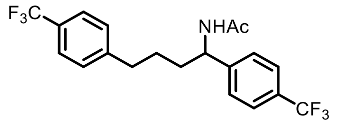

***N-(1,4-bis(4-(trifluoromethyl)phenyl)butyl)acetamide* (12).** Prepared according to GP3 from **1j** (109 mg, 0.3 mmol, 1.0 equiv.) and **2a** (54 µL, 0.36 mmol, 1.2 equiv.). Purified via flash column chromatography on silica gel (Pentane:Ethyl Acetate 1:1) to afford the product as a yellowish solid (66 mg, 55% yield).

^{1}**H NMR** (400 MHz, $CDCl_3$) δ 7.58 (d, *J* = 8.1 Hz, 2H), 7.52 (d, *J* = 8.0 Hz, 2H), 7.36 (d, *J* = 8.1 Hz, 2H), 7.23 (d, *J* = 7.9 Hz, 2H), 5.74 (d, *J* = 8.2 Hz, 1H), 5.03 (q, *J* = 7.6 Hz, 1H), 2.69 (td, *J* = 7.7, 3.7 Hz, 2H), 1.98 (s, 3H), 1.83 – 1.77 (m, 2H), 1.71 – 1.66 (m, 1H), 1.60 – 1.55 (m, 1H).
^{13}**C NMR** (151 MHz, $CDCl_3$) δ 169.5, 146.2, 145.8, 129.9 (q, *J* = 32.5 Hz), 128.8, 128.5 (d, *J* = 32.4 Hz), 127.0, 125.9 (q, *J* = 3.8 Hz), 125.5 (q, *J* = 3.7 Hz), 124.4 (q, *J* = 271.8 Hz), 124.2 (q, *J* = 271.8 Hz), 53.1, 35.4, 35.3, 27.7, 23.5.
^{19}**F NMR** (376 MHz, $CDCl_3$) δ −62.86, −63.07.
HRMS (ESI+) m/z $[M+H]^+$ calcd. for $C_{19}H_{20}F_3NO$ 404.1444, found 404.1447.

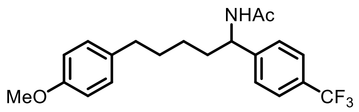

***N-(5-(4-methoxyphenyl)-1-(4-(trifluoromethyl)phenyl)pentyl)acetamide* (13).** Prepared according to GP3 from **1l** (102 mg, 0.3 mmol, 1.0 equiv.) and **2a** (54 µL, 0.36 mmol, 1.2 equiv.). Purified via flash column chromatography on silica gel (Pentane:Ethyl Acetate 1:1) to afford the product as a yellowish solid (56 mg, 49% yield).

^{1}**H NMR** (400 MHz, $CDCl_3$) δ 7.57 (d, *J* = 8.1 Hz, 2H), 7.36 (d, *J* = 8.0 Hz, 2H), 7.04 (d, *J* = 8.6 Hz, 2H), 6.81 (d, *J* = 8.6 Hz, 2H), 5.82 (d, *J* = 8.0 Hz, 1H), 4.96 (q, *J* = 7.6 Hz, 1H), 3.78 (s, 3H), 2.52 (t, *J* = 7.6 Hz, 2H), 1.97 (s, 3H), 1.82 – 1.74 (m, 2H), 1.64 – 1.53 (m, 2H), 1.39 – 1.20 (m, 2H).
^{13}**C NMR** (151 MHz, $CDCl_3$) δ 169.5, 157.9, 146.7, 134.3, 129.6 (d, *J* = 32.7 Hz), 129.3, 127.0, 125.72 (q, *J* = 3.9 Hz), 124.2 (d, *J* = 271.9 Hz), 113.9, 55.4, 53.3, 36.0, 34.8, 31.3, 25.7, 23.4.
^{19}**F NMR** (376 MHz, $CDCl_3$) δ −63.00.
HRMS (ESI+) m/z $[M+H]^+$ calcd. for $C_{21}H_{24}F_3NO_2$ 380.1832, found 380.1836.

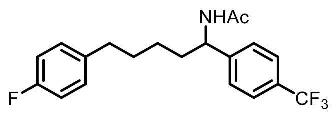

***N*-(5-(4-fluorophenyl)-1-(4-(trifluoromethyl)phenyl)pentyl)acetamide** **(14).** Prepared according to GP3 from **1m** (98 mg, 0.3 mmol, 1.0 equiv.) and **2a** (54 μL, 0.36 mmol, 1.2 equiv.). Purified via flash column chromatography on silica gel (Pentane:Ethyl Acetate 1:1) to afford the product as a yellowish solid (71 mg, 62% yield).

^{1}H NMR (400 MHz, $CDCl_3$) δ 7.57 (d, *J* = 8.0 Hz, 2H), 7.36 (d, *J* = 8.0 Hz, 2H), 7.07 (dd, *J* = 8.5, 5.6 Hz, 2H), 6.94 (t, *J* = 8.7 Hz, 2H), 5.83 (d, *J* = 8.1 Hz, 1H), 4.97 (q, *J* = 7.6 Hz, 1H), 2.54 (t, *J* = 7.7 Hz, 2H), 1.97 (s, 3H), 1.81 – 1.75 (m, 2H), 1.64 – 1.58 (m, 2H), 1.36 – 1.26 (m, 2H).
^{13}C NMR (151 MHz, $CDCl_3$) δ 169.6, 161.3 (d, *J* = 243.6 Hz), 146.6, 137.8 (d, *J* = 3.2 Hz), 129.8 (d, *J* = 7.6 Hz), 129.5 (q, *J* = 32.6 Hz), 127.0, 125.8 (q, *J* = 3.7 Hz), 124.2 (q, *J* = 271.9 Hz), 115.1 (d, *J* = 21.1 Hz), 53.2, 36.0, 34.9, 31.2, 25.7, 23.4.
^{19}F NMR (376 MHz, $CDCl_3$) δ −63.01, −118.32.
HRMS (ESI+) m/z $[M+H]^+$ calcd. for $C_{20}H_{21}F_4NO$ 368.1632, found 368.1636.

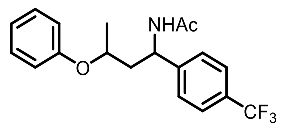

***N*-(3-phenoxy-1-(4-(trifluoromethyl)phenyl)butyl)acetamide** **(15).** Prepared according to GP3 from **1ah** (93 mg, 0.3 mmol, 1.0 equiv.) and **2a** (54 μL, 0.36 mmol, 1.2 equiv.). Purified via flash column chromatography on silica gel (Pentane:Ethyl Acetate 1:1) to afford the two diastereomers as an inseparable mixture as a yellowish oil (45 mg, 43% yield). *d.r.* ratio of **15** was determined via ^{1}H NMR to be 1.2:1.

^{1}H NMR (400 MHz, $CDCl_3$) δ 7.55 (d, *J* = 7.4 Hz, 2H), 7.40 – 7.27 (m, 4H), 7.02 – 6.95 (m, 1H), 6.89 (d, *J* = 7.2 Hz, 0.5H), 6.84 (d, *J* = 7.9 Hz, 1H), 6.78 (d, *J* = 7.9 Hz, 1H), 6.30 (d, *J* = 6.9 Hz, 0.4H), 5.30 (q, *J* = 7.1 Hz, 0.5H), 5.17 (q, *J* = 7.1 Hz, 0.4H), 4.37 – 4.32 (m, 0.4H), 4.31 – 4.26 (m, 0.5H), 2.29 – 2.20 (m, 1H), 2.09 (ddd, *J* = 14.8, 6.7, 3.0 Hz, 0.5H), 2.01 (s, 1.5H), 1.98 – 1.94 (m, 0.4H), 1.88 (s, 1.2H), 1.29 (d, *J* = 6.1 Hz, 1.3H), 1.24 (d, *J* = 6.1 Hz, 1.6H).
^{13}C NMR (151 MHz, $CDCl_3$) δ 169.8, 169.5, 157.3, 156.7, 146.6, 145.8, 130.1 (q, *J* = 32.6 Hz), 129.9, 129.82, 129.6 (q, *J* = 32.6 Hz), 126.9, 126.7, 125.8 (q, *J* = 3.7 Hz), 125.7 (q, *J* = 3.7 Hz), 124.2 (q, *J* = 271.9 Hz), 124.2 (q, *J* = 271.9 Hz), 121.8, 121.4, 116.2, 116.0, 72.1, 71.4, 52.3, 51.5, 43.3, 42.1, 23.5, 23.4, 20.2, 19.7.
^{19}F NMR (376 MHz, $CDCl_3$) δ −62.98, −63.01.
HRMS (ESI+) m/z $[M+H]^+$ calcd. for $C_{19}H_{20}F_3NO_2$ 352.1519, found 352.1525.

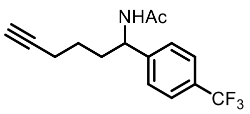

N*-(1-(4-(trifluoromethyl)phenyl)hex-5-yn-1-yl)acetamide** **(16)*. Prepared according to GP3 from **1ae** (73 mg, 0.3 mmol, 1.0 equiv.) and **2a** (54 µL, 0.36 mmol, 1.2 equiv.). Purified via flash column chromatography on silica gel (from Pentane:Ethyl Acetate 2:1 to 1:1) to afford the product as a yellowish solid (27 mg, 32% yield).

^{1}H NMR (400 MHz, $CDCl_3$) δ 7.59 (d, *J* = 7.8 Hz, 2H), 7.40 (d, *J* = 8.5 Hz, 2H), 5.83 (d, *J* = 8.2 Hz, 1H), 5.02 (q, *J* = 7.6 Hz, 1H), 2.23 (td, *J* = 6.9, 2.7 Hz, 2H), 1.99 (s, 3H), 1.97 (t, *J* = 2.6 Hz, 1H), 1.92 (q, *J* = 7.7 Hz, 2H), 1.60 – 1.45 (m, 2H).
^{13}C NMR (151 MHz, $CDCl_3$) δ 169.6, 146.3, 129.9 (q, *J* = 32.4 Hz), 127.0, 125.9 (q, *J* = 3.8 Hz), 124.2 (d, *J* = 272.1 Hz), 83.7, 69.3, 52.9, 35.0, 25.1, 23.5, 18.2.
^{19}F NMR (376 MHz, $CDCl_3$) δ −63.05.
HRMS (ESI+) m/z $[M+H]^+$ calcd. for $C_{15}H_{16}F_3NO$ 284.1257, found 284.1257.

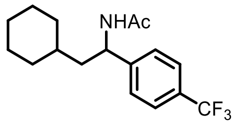

N*-(2-cyclohexyl-1-(4-(trifluoromethyl)phenyl)ethyl)acetamide** **(17)*. Prepared according to GP3 from **1r** (82 mg, 0.3 mmol, 1.0 equiv.) and **2a** (54 µL, 0.36 mmol, 1.2 equiv.). Purified via flash column chromatography on silica gel (from Pentane:Ethyl Acetate 2:1 to 1:1) to afford the product as a yellowish oil (47 mg, 50% yield).

^{1}H NMR (400 MHz, $CDCl_3$) δ 7.56 (d, *J* = 8.1 Hz, 2H), 7.38 (d, *J* = 8.1 Hz, 2H), 5.94 (d, *J* = 8.0 Hz, 1H), 5.09 (q, *J* = 7.9 Hz, 1H), 1.98 (s, 3H), 1.81 – 1.60 (m, 7H), 1.31 – 1.08 (m, 4H), 0.96 – 0.84 (m, 2H).
^{13}C NMR (151 MHz, $CDCl_3$) δ 169.4, 147.1, 129.3 (d, *J* = 32.3 Hz), 126.7, 125.5 (q, *J* = 3.8 Hz), 124.0 (d, *J* = 271.9 Hz), 50.7, 44.0, 34.3, 33.3, 32.8, 26.3, 26.0, 25.9, 23.2.
^{19}F NMR (376 MHz, $CDCl_3$) δ −62.98.
HRMS (ESI+) m/z $[M+H]^+$ calcd. for $C_{17}H_{22}F_3NO$ 314.1726, found 314.1725.

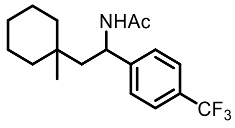

N*-(2-(1-methylcyclohexyl)-1-(4-(trifluoromethyl)phenyl)ethyl)acetamide** **(18)*. Prepared according to GP3 from **1u** (86 mg, 0.3 mmol, 1.0 equiv.) and **2a** (54 µL, 0.36 mmol, 1.2 equiv.). Purified via flash column chromatography on silica gel (from Pentane:Ethyl Acetate 2:1 to 1:1) to afford the product as a yellowish oil (43 mg, 44% yield).

^{1}H NMR (400 MHz, $CDCl_3$) δ 7.56 (d, *J* = 8.0 Hz, 2H), 7.38 (d, *J* = 8.0 Hz, 2H), 5.85 (d, *J* = 8.1 Hz, 1H), 5.15 (q, *J* = 7.6 Hz, 1H), 1.96 (s, 3H), 1.69 (d, *J* = 7.3 Hz, 2H), 1.49 – 1.37 (m, 5H), 1.35 – 1.23 (m, 5H), 0.95 (s, 3H).

^{13}**C NMR** (151 MHz, $CDCl_3$) δ 169.1, 148.8, 129.3 (q, *J* = 32.4 Hz), 126.8, 125.7 (q, *J* = 3.7 Hz), 124.2 (d, *J* = 266.8 Hz), 50.0, 38.4, 38.2, 33.5, 26.4, 23.5, 22.1, 22.0.
^{19}**F NMR** (376 MHz, $CDCl_3$) δ −62.97.
HRMS (ESI+) m/z $[M+H]^+$ calcd. for $C_{18}H_{24}F_3NO$ 328.1883, found 328.1885.

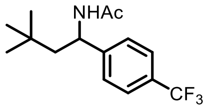

N-(3,3-dimethyl-1-(4-(trifluoromethyl)phenyl)butyl)acetamide **(19)*.*** Prepared according to GP3 from **1z** (74 mg, 0.3 mmol, 1.0 equiv.) and **2a** (54 μL, 0.36 mmol, 1.2 equiv.). Purified via flash column chromatography on silica gel (from Pentane:Ethyl Acetate 2:1 to 1:1) to afford the product as a yellowish solid (43 mg, 50% yield).

Characterization data are in accordance with literature.[106]
^{1}**H NMR** (400 MHz, $CDCl_3$) δ 7.55 (d, *J* = 8.1 Hz, 2H), 7.38 (d, *J* = 8.6 Hz, 2H), 6.00 (d, *J* = 8.1 Hz, 1H), 5.14 (d, *J* = 7.0 Hz, 1H), 1.96 (s, 3H), 1.68 (d, *J* = 6.9 Hz, 2H), 0.95 (s, 9H).
^{13}**C NMR** (151 MHz, $CDCl_3$) δ 169.3, 148.6, 129.4 (q, *J* = 32.4 Hz), 126.8, 125.7 (q, *J* = 3.9 Hz), 124.2 (d, *J* = 272.1 Hz), 50.9, 50.7, 31.0, 30.0, 23.5.
^{19}**F NMR** (376 MHz, $CDCl_3$) δ −62.98.
HRMS (ESI+) m/z $[M+H]^+$ calcd. for $C_{15}H_{20}F_3NO$ 288.1570, found 288.1572.

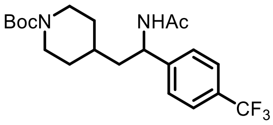

Tert-butyl 4-(2-acetamido-2-(4-(trifluoromethyl)phenyl)ethyl)piperidine-1-carboxylate **(20)*.*** Prepared according to GP3 from **1ab** (112 mg, 0.3 mmol, 1.0 equiv.) and **2a** (54 μL, 0.36 mmol, 1.2 equiv.). $Zn(CF_3COO)_2 \cdot xH_2O$ (43 mg, 0.5 equiv.) was used as additive instead of TFA. Purified via flash column chromatography on silica gel (from Pentane:Ethyl Acetate 1:1 to 1:2) to afford the product as a yellowish oil (54 mg, 44% yield).

^{1}**H NMR** (400 MHz, $CDCl_3$) δ 7.57 (d, *J* = 8.1 Hz, 2H), 7.38 (d, *J* = 8.0 Hz, 2H), 5.94 (d, *J* = 8.3 Hz, 1H), 5.12 (td, *J* = 8.5, 6.5 Hz, 1H), 4.14 – 3.95 (m, 2H), 2.67 – 2.56 (m, 2H), 1.97 (s, 3H), 1.84 – 1.63 (m, 5H), 1.44 (m, 9H), 1.24 – 1.02 (m, 2H).
^{13}**C NMR** (151 MHz, $CDCl_3$) δ 169.5, 154.9, 146.7, 129.8 (q, *J* = 32.5 Hz), 127.0, 125.8 (q, *J* = 3.8 Hz), 124.2 (d, *J* = 271.9 Hz), 79.6, 50.5, 44.1, 43.4, 43.1, 33.0, 32.3, 31.9, 28.6, 23.5.
^{19}**F NMR** (376 MHz, $CDCl_3$) δ −63.03.
HRMS (ESI+) m/z $[M+Na]^+$ calcd. for $C_{21}H_{29}F_3N_2O_3$ 437.2022, found 437.2031.

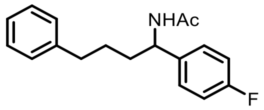

***N*-(1-(4-fluorophenyl)-4-phenylbutyl)acetamide** **(21).** Prepared according to GP3 from **1a** (89 mg, 0.3 mmol, 1.0 equiv.) and **2b** (43 µL, 0.36 mmol, 1.2 equiv.). Purified via flash column chromatography on silica gel (from Pentane:Ethyl Acetate 2:1 to 1:1) to afford the product as a yellowish solid (36 mg, 42% yield).

^{1}H NMR (400 MHz, $CDCl_3$) δ 7.28 – 7.24 (m, 2H), 7.22 – 7.18 (m, 3H), 7.14 – 7.11 (m, 2H), 7.00 (t, *J* = 8.7 Hz, 2H), 5.81 (d, *J* = 8.2 Hz, 1H), 4.95 (q, *J* = 7.7 Hz, 1H), 2.62 (t, *J* = 7.5 Hz, 2H), 1.96 (s, 3H), 1.81 – 1.76 (m, 2H), 1.63 – 1.56 (m, 2H).
^{13}C NMR (126 MHz, $CDCl_3$) δ 169.8, 162.1 (d, *J* = 245.8 Hz), 141.9, 137.9 (d, *J* = 3.1 Hz), 128.5, 128.5, 128.3 (d, *J* = 8.1 Hz), 126.0, 115.6 (d, *J* = 21.4 Hz), 53.0, 35.6, 35.5, 28.0, 23.4.
^{19}F NMR (376 MHz, $CDCl_3$) δ −115.66.
HRMS (EI+) m/z $[M]^+$ calcd. for $C_{18}H_{20}FNO$ 285.1523, found 285.1523.

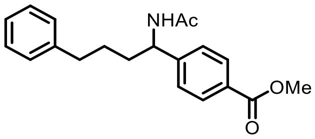

Methyl 4-(1-acetamido-4-phenylbutyl)benzoate **(22).** Prepared according to GP3 from **1a** (89 mg, 0.3 mmol, 1.0 equiv.) and **2c** (58 mg, 0.36 mmol, 1.2 equiv.). Purified via flash column chromatography on silica gel (Pentane:Ethyl Acetate 1:1) to afford the product as a yellowish solid (69 mg, 70% yield).

^{1}H NMR (400 MHz, $CDCl_3$) δ 7.97 (d, *J* = 8.4 Hz, 2H), 7.32 – 7.23 (m, 4H), 7.19 – 7.15 (m, 1H), 7.11 (d, *J* = 7.0 Hz, 2H), 5.95 (d, *J* = 8.2 Hz, 1H), 5.02 (q, *J* = 7.6 Hz, 1H), 3.90 (s, 3H), 2.61 (td, *J* = 7.5, 2.8 Hz, 2H), 1.96 (s, 3H), 1.80 (m, 2H), 1.68 – 1.62 (m, 1H), 1.58 – 1.55 (m, 1H).
^{13}C NMR (101 MHz, $CDCl_3$) δ 169.5, 166.9, 147.6, 141.8, 130.1, 129.3, 128.5, 128.5, 126.7, 126.0, 53.2, 52.2, 35.6, 35.5, 28.0, 23.4.
HRMS (EI+) m/z $[M]^+$ calcd. for $C_{20}H_{23}NO$ 325.1672, found 325.1671.

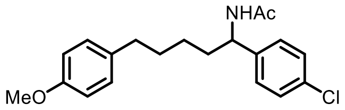

***N*-(1-(4-chlorophenyl)-5-(4-methoxyphenyl)pentyl)acetamide** **(23).** Prepared according to GP3 from **1l** (102 mg, 0.3 mmol, 1.0 equiv.) and **2d** (43 µL, 0.36 mmol, 1.2 equiv.). Purified via flash column chromatography on silica gel (Pentane:Ethyl Acetate 1:1) to afford the product as a yellowish solid (47 mg, 46% yield).

^{1}H NMR (400 MHz, $CDCl_3$) δ 7.27 (d, *J* = 11.9 Hz, 2H), 7.17 (d, *J* = 8.4 Hz, 2H), 7.03 (d, *J* = 8.6 Hz, 2H), 6.80 (d, *J* = 8.6 Hz, 2H), 5.79 (d, *J* = 8.2 Hz, 1H), 4.88 (q, *J* = 7.6 Hz, 1H), 3.77 (s, 3H), 2.50 (t, *J* = 7.6 Hz, 2H), 1.94 (s, 3H), 1.76 – 1.73 (m, 2H), 1.60 – 1.55 (m, 2H), 1.34 – 1.30 (m, 1H), 1.25 – 1.22 (m, 1H).

^{13}C NMR (101 MHz, $CDCl_3$) δ 169.4, 157.8, 141.1, 134.4, 133.1, 129.3, 128.9, 128.1, 113.8, 55.4, 53.0, 36.0, 34.8, 31.3, 25.7, 23.5.
HRMS (EI+) m/z $[M]^+$ calcd. for $C_{20}H_{24}{}^{35}ClNO_2$ 345.1490, found 345.1488.

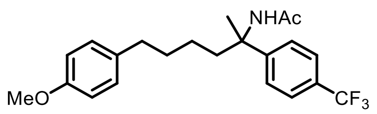

***N-(6-(4-methoxyphenyl)-2-(4-(trifluoromethyl)phenyl)hexan-2-yl)acetamide* (24).** Prepared according to GP3 from **1l** (102 mg, 0.3 mmol, 1.0 equiv.) and **2o** (67 mg, 0.36 mmol, 1.2 equiv.). Purified via flash column chromatography on silica gel (Pentane:Ethyl Acetate 1:1) to afford the product as a colorless oil (45 mg, 38% yield).

^{1}H NMR (400 MHz, $CDCl_3$) δ 7.56 (d, J = 8.3 Hz, 2H), 7.41 (d, J = 8.2 Hz, 2H), 7.02 (d, J = 8.6 Hz, 2H), 6.80 (d, J = 8.6 Hz, 2H), 5.69 (s, 1H), 3.78 (s, 3H), 2.53 – 2.47 (m, 2H), 2.06 – 2.01 (m, 1H), 1.98 (s, 3H), 1.84 – 1.77 (m, 1H), 1.69 (s, 3H), 1.58 – 1.50 (m, 2H), 1.22 – 1.14 (m, 2H).
^{13}C NMR (151 MHz, $CDCl_3$) δ 169.2, 157.9, 150.0, 134.3, 129.3, 128.8 (q, J = 32.3 Hz), 125.65, 125.4 (q, J = 3.8 Hz), 124.4 (q, J = 271.8 Hz), 113.9, 58.6, 55.4, 42.1, 34.7, 31.7, 25.7, 24.3, 23.3.
^{19}F NMR (376 MHz, $CDCl_3$) δ −62.91.
HRMS (EI+) m/z $[M]^+$ calcd. for $C_{22}H_{26}F_3NO_2$ 393.1910, found 393.1914.

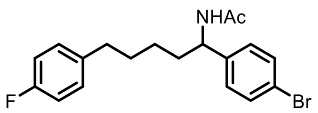

***N-(1-(4-bromophenyl)-5-(4-fluorophenyl)pentyl)acetamide* (25).** Prepared according to GP3 from **1m** (98 mg, 0.3 mmol, 1.0 equiv.) and **2e** (47 μL, 0.36 mmol, 1.2 equiv.). Purified via flash column chromatography on silica gel (Pentane:Ethyl Acetate 1:1) to afford the product as a yellowish solid (64 mg, 57% yield).

^{1}H NMR (400 MHz, $CDCl_3$) δ 7.44 (d, J = 8.4 Hz, 2H), 7.13 (d, J = 8.4 Hz, 2H), 7.09 – 7.04 (m, 2H), 6.94 (t, J = 8.7 Hz, 2H), 5.69 (d, J = 8.2 Hz, 1H), 4.88 (q, J = 7.6 Hz, 1H), 2.53 (t, J = 7.7 Hz, 2H), 1.96 (s, 3H), 1.80 – 1.73 (m, 2H), 1.62 – 1.57 (m, 1H), 1.34 – 1.27 (m, 1H).
^{13}C NMR (126 MHz, $CDCl_3$) δ 169.4, 161.3 (d, J = 243.3 Hz), 141.5, 137.9 (d, J = 3.1 Hz), 131.9, 129.8 (d, J = 7.6 Hz), 128.5, 121.3, 115.1 (d, J = 21.0 Hz), 53.0, 35.9, 34.9, 31.3, 25.7, 23.5.
^{19}F NMR (376 MHz, $CDCl_3$) δ −118.37.
HRMS (EI+) m/z $[M]^+$ calcd. for $C_{19}H_{21}{}^{79}BrFNO$ 377.0785, found 377.0785.

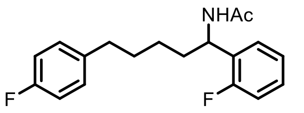

***N-(1-(2-fluorophenyl)-5-(4-fluorophenyl)pentyl)acetamide* (26).** Prepared according to GP3 from **1m** (98 mg, 0.3 mmol, 1.0 equiv.) and **2f** (43 μL, 0.36 mmol, 1.2 equiv.). Purified via flash column chromatography on silica gel (Pentane:Ethyl Acetate 1:1) to afford the product as a yellowish solid (53 mg, 56% yield).

^{1}H NMR (400 MHz, $CDCl_3$) δ 7.23 (t, *J* = 7.2 Hz, 2H), 7.10 – 7.01 (m, 4H), 6.93 (t, *J* = 8.7 Hz, 2H), 6.00 (d, *J* = 8.9 Hz, 1H), 5.10 (q, *J* = 7.9 Hz, 1H), 2.53 (t, *J* = 7.6 Hz, 2H), 1.97 (s, 3H), 1.85 – 1.80 (m, 2H), 1.62 – 1.57 (m, 2H), 1.37 – 1.32 (m, 1H), 1.27 – 1.22 (m, 1H).
^{13}C NMR (126 MHz, $CDCl_3$) δ 169.3, 161.3 (d, *J* = 243 Hz), 161.1 (d, *J* = 243 Hz), 138.0 (d, *J* = 3.3 Hz), 129.8 (d, *J* = 7.9 Hz), 129.3 (d, *J* = 5.4 Hz), 129.2 (d, *J* = 5.4 Hz), 129.1 (d, *J* = 8.8 Hz), 124.4 (d, *J* = 3.1 Hz), 116.0 (d, *J* = 21.9 Hz), 115.1 (d, *J* = 21.0 Hz), 50.5, 35.4 (d, *J* = 1.9 Hz), 34.9, 31.2, 25.9, 23.6.
^{19}F NMR (376 MHz, $CDCl_3$) δ −118.54, −118.56.
HRMS (EI+) m/z $[M]^+$ calcd. for $C_{19}H_{21}F_2NO$ 317.1586, found 317.1578.

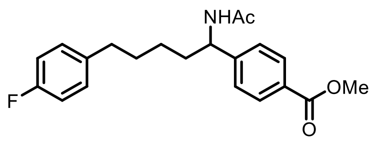

Methyl 4-(1-acetamido-5-(4-fluorophenyl)pentyl)benzoate **(27).** Prepared according to GP3 from **1m** (98 mg, 0.3 mmol, 1.0 equiv.) and **2c** (58 mg, 0.36 mmol, 1.2 equiv.). Purified via flash column chromatography on silica gel (Pentane:Ethyl Acetate 1:1) to afford the product as a yellowish solid (73 mg, 67% yield).

^{1}H NMR (400 MHz, $CDCl_3$) δ 7.98 (d, *J* = 8.3 Hz, 2H), 7.31 (d, *J* = 8.3 Hz, 2H), 7.06 (dd, *J* = 8.5, 5.6 Hz, 2H), 6.92 (t, *J* = 8.7 Hz, 2H), 5.95 (d, *J* = 8.2 Hz, 1H), 4.97 (q, *J* = 7.6 Hz, 1H), 3.90 (s, 3H), 2.52 (t, *J* = 7.7 Hz, 2H), 1.96 (s, 3H), 1.80 – 1.75 (m, 2H), 1.61 – 1.57 (m, 2H), 1.35 – 1.30 (m, 1H), 1.26 – 1.22 (m, 1H).
^{13}C NMR (151 MHz, $CDCl_3$) δ 169.5, 166.9, 161.3 (d, *J* = 243.2 Hz), 147.7, 137.9 (d, *J* = 3.1 Hz), 130.1, 129.7 (d, *J* = 7.8 Hz), 129.3, 126.7, 115.1 (d, *J* = 21.1 Hz), 53.3, 52.2, 36.0, 34.9, 31.2, 25.7, 23.4.
^{19}F NMR (376 MHz, $CDCl_3$) δ −118.36.
HRMS (EI+) m/z $[M]^+$ calcd. for $C_{21}H_{24}FNO_3$ 357.1735, found 357.1728.

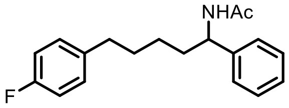

N-(5-(4-fluorophenyl)-1-phenylpentyl)acetamide **(28).** Prepared according to GP3 from **1m** (98 mg, 0.3 mmol, 1.0 equiv.) and **2g** (41 µL, 0.36 mmol, 1.2 equiv.). Purified via flash column chromatography on silica gel (Pentane:Ethyl Acetate 1:1) to afford the product as a yellowish oil (32 mg, 36% yield).

^{1}H NMR (400 MHz, $CDCl_3$) δ 7.35 – 7.31 (m, 2H), 7.28 – 7.25 (m, 3H), 7.07 (dd, *J* = 8.5, 5.5 Hz, 2H), 6.93 (t, *J* = 8.7 Hz, 2H), 5.72 (d, *J* = 8.4 Hz, 1H), 4.95 (q, *J* = 7.7 Hz, 1H), 2.53 (t, *J* = 7.6 Hz, 2H), 1.96 (s, 3H), 1.85 – 1.77 (m, 2H), 1.63 – 1.57 (m, 2H), 1.36 – 1.32 (m, 1H), 1.28 – 1.24 (m, 1H).
^{13}C NMR (101 MHz, $CDCl_3$) δ 169.3, 161.3 (d, *J* = 243.1 Hz), 142.3, 138.1 (d, *J* = 3.3 Hz), 129.8 (d, *J* = 7.7 Hz), 128.8, 127.6, 126.7, 115.1 (d, *J* = 21.0 Hz), 53.5, 36.0, 35.0, 31.3, 25.8, 23.6.
^{19}F NMR (376 MHz, $CDCl_3$) δ −118.52.
HRMS (EI+) m/z $[M]^+$ calcd. for $C_{19}H_{22}FNO$ 299.1680, found 299.1684.

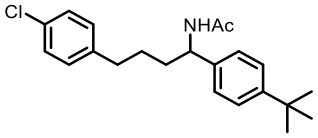

N-(1-(4-(tert-butyl)phenyl)-4-(4-chlorophenyl)butyl)acetamide **(29).** Prepared according to GP3 from **1h** (99 mg, 0.3 mmol, 1.0 equiv.) and **2h** (66 µL, 0.36 mmol, 1.2 equiv.). Purified via flash column chromatography on silica gel (Pentane:Ethyl Acetate 1:1) to afford the product as a yellowish oil (42 mg, 39% yield).

^{1}H NMR (400 MHz, $CDCl_3$) δ 7.34 (d, *J* = 8.4 Hz, 2H), 7.21 (d, *J* = 8.4 Hz, 2H), 7.17 (d, *J* = 8.3 Hz, 2H), 7.05 (d, *J* = 8.4 Hz, 2H), 5.70 (d, *J* = 8.4 Hz, 1H), 4.97 (q, *J* = 7.7 Hz, 1H), 2.59 (t, *J* = 7.6 Hz, 2H), 1.95 (s, 3H), 1.83 – 1.76 (m, 2H), 1.68 – 1.60 (m, 1H), 1.56 – 1.53 (m, 1H), 1.30 (s, 9H).
^{13}C NMR (101 MHz, $CDCl_3$) δ 169.3, 150.5, 140.6, 139.0, 131.6, 129.9, 128.5, 126.4, 125.8, 53.0, 35.5, 34.9, 34.6, 31.5, 28.0, 23.6.
HRMS (EI+) m/z $[M]^+$ calcd. for $C_{22}H_{28}{}^{35}ClNO$ 357.1854, found 357.1845.

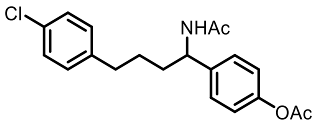

4-(1-Acetamido-4-(4-chlorophenyl)butyl)phenyl acetate **(30).** Prepared according to GP3 from **1h** (99 mg, 0.3 mmol, 1.0 equiv.) and **2i** (55 µL, 0.36 mmol, 1.2 equiv.). Purified via flash column chromatography on silica gel (from Pentane:Ethyl Acetate 1:1 to 1:2) to afford the product as a yellowish solid (47 mg, 44% yield).

^{1}H NMR (400 MHz, $CDCl_3$) δ 7.30 – 7.23 (m, 4H), 7.07 (dd, *J* = 8.5, 6.5 Hz, 4H), 5.79 (d, *J* = 8.4 Hz, 1H), 5.01 (q, *J* = 7.7 Hz, 1H), 2.61 (td, *J* = 7.4, 1.4 Hz, 2H), 2.31 (s, 3H), 1.97 (s, 3H), 1.83 – 1.76 (m, 2H), 1.67 – 1.62 (m, 1H), 1.57 – 1.55 (m, 1H).
^{13}C NMR (101 MHz, $CDCl_3$) δ 169.6, 169.3, 150.0, 140.4, 139.7, 131.7, 129.9, 128.5, 127.8, 121.9, 52.6, 35.4, 34.9, 27.9, 23.5, 21.2.
HRMS (EI+) m/z $[M]^+$ calcd. for $C_{20}H_{22}{}^{35}ClNO_3$ 359.1283, found 359.1280.

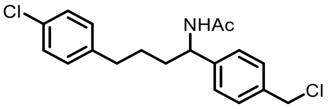

N-(1-(4-(chloromethyl)phenyl)-4-(4-chlorophenyl)butyl)acetamide **(31).** Prepared according to GP3 from **1h** (99 mg, 0.3 mmol, 1.0 equiv.) and **2j** (55 µL, 0.36 mmol, 1.2 equiv.). Purified via flash column chromatography on silica gel (Pentane:Ethyl Acetate 1:1) to afford the product as a yellowish solid (55 mg, 53% yield).

^{1}H NMR (400 MHz, $CDCl_3$) δ 7.35 (d, *J* = 8.2 Hz, 2H), 7.23 (dd, *J* = 8.1, 5.8 Hz, 4H), 7.05 (d, *J* = 8.4 Hz, 2H), 5.65 (d, *J* = 8.3 Hz, 1H), 4.98 (q, *J* = 7.7 Hz, 1H), 4.56 (s, 2H), 2.59 (t, *J* = 7.5 Hz, 2H), 1.96 (s, 3H), 1.78 (ddt, *J* = 9.7, 3.7, 2.0 Hz, 2H), 1.63 – 1.49 (m, 2H).
^{13}C NMR (101 MHz, $CDCl_3$) δ 169.3, 142.5, 140.4, 136.9, 131.7, 129.9, 129.2, 128.6, 127.1, 53.1, 46.0, 35.4, 34.9, 27.9, 23.6.
HRMS (EI+) m/z $[M]^+$ calcd. for $C_{19}H_{21}{}^{35}Cl_2NO$ 349.0995, found 349.0992.

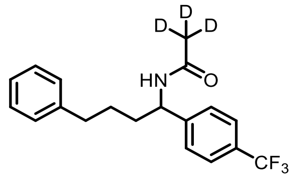

***N-(4-phenyl-1-(4-(trifluoromethyl)phenyl)butyl)acetamide-2,2,2-d3* (32).** Prepared according to GP3 from **1a** (89 mg, 0.3 mmol, 1.0 equiv.) and **2a** (54 µL, 0.36 mmol, 1.2 equiv.). CD_3CN (3 mL) was used as solvent. Purified via flash column chromatography on silica gel (from Pentane:Ethyl Acetate 2:1 to 1:1) to afford the product as a yellowish solid (48 mg, 46% yield).

^{1}H NMR (400 MHz, $CDCl_3$) δ 7.57 (d, J = 8.0 Hz, 2H), 7.35 (d, J = 8.0 Hz, 2H), 7.29 – 7.26 (m, 2H), 7.19 (t, J = 7.4 Hz, 1H), 7.13 (d, J = 7.4 Hz, 2H), 5.84 (d, J = 8.0 Hz, 1H), 5.01 (q, J = 7.6 Hz, 1H), 2.63 (td, J = 7.4, 3.5 Hz, 2H), 1.83 – 1.77 (m, 2H), 1.69 – 1.64 (m, 1H), 1.60 – 1.55 (m, 1H).
^{13}C NMR (151 MHz, $CDCl_3$) δ 169.6, 146.5, 141.7, 129.7 (q, J = 32.5 Hz), 128.6, 128.5, 127.0, 126.1, 125.8 (q, J = 3.9 Hz), 124.2 (q, J = 271.9 Hz), 53.2, 35.52, 35.5, 28.0, 23.1 – 22.4 (m).
^{19}F NMR (376 MHz, $CDCl_3$) δ −63.02.
HRMS (ESI+) m/z $[M+H]^+$ calcd. for $C_{19}H_{17}D_3F_3NO$ 339.1758, found 339.1763.

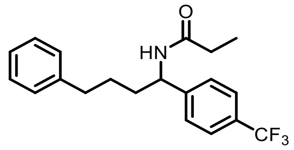

***N-(4-phenyl-1-(4-(trifluoromethyl)phenyl)butyl)propionamide* (33).** Prepared according to GP3 from **1a** (89 mg, 0.3 mmol, 1.0 equiv.) and **2a** (54 µL, 0.36 mmol, 1.2 equiv.). Propionitrile (3 mL) was used as solvent. Purified via flash column chromatography on silica gel (Pentane:Ethyl Acetate 2:1) to afford the product as a yellowish solid (43 mg, 40% yield).

^{1}H NMR (400 MHz, $CDCl_3$) δ 7.57 (d, J = 8.1 Hz, 2H), 7.35 (d, J = 8.0 Hz, 2H), 7.29 – 7.25 (m, 2H), 7.21 – 7.16 (m, 1H), 7.13 (d, J = 6.9 Hz, 2H), 5.65 (d, J = 8.0 Hz, 1H), 5.03 (q, J = 7.6 Hz, 1H), 2.64 (dt, J = 7.6, 3.7 Hz, 2H), 2.20 (qd, J = 7.6, 2.8 Hz, 2H), 1.84 – 1.77 (m, 2H), 1.69 – 1.56 (m, 2H), 1.13 (t, J = 7.6 Hz, 3H).
^{13}C NMR (101 MHz, $CDCl_3$) δ 173.2, 146.7, 141.7, 129.7 (q, J = 32.5 Hz), 128.6, 128.5, 127.0, 126.1, 125.8 (q, J = 3.7 Hz), 124.2 (q, J = 272.2 Hz), 53.0, 35.6, 35.5, 29.8, 28.0, 9.9.
^{19}F NMR (376 MHz, $CDCl_3$) δ −63.01.
HRMS (EI+) m/z $[M]^+$ calcd. for $C_{20}H_{22}F_3NO$ 349.1648, found 349.1648.

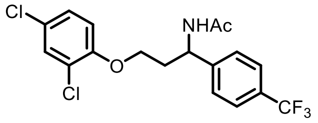

***N-(3-(2,4-dichlorophenoxy)-1-(4-(trifluoromethyl)phenyl)propyl)acetamide* (34).** Prepared according to GP3 from **1aj** (109 mg, 0.3 mmol, 1.0 equiv.) and **2a** (54 µL, 0.36 mmol, 1.2 equiv.). Purified via flash column chromatography on silica gel (Pentane:Ethyl Acetate 1:1) to afford the product as a yellowish solid (70 mg, 58% yield).

^{1}**H NMR** (400 MHz, $CDCl_3$) δ 7.55 (d, *J* = 8.1 Hz, 2H), 7.41 (d, *J* = 2.5 Hz, 1H), 7.37 (d, *J* = 8.1 Hz, 2H), 7.21 (d, *J* = 7.7 Hz, 1H), 7.17 (dd, *J* = 8.8, 2.5 Hz, 1H), 6.72 (d, *J* = 8.8 Hz, 1H), 5.40 (td, *J* = 7.2, 3.8 Hz, 1H), 4.04 (dt, *J* = 9.1, 4.4 Hz, 2H), 3.80 (td, *J* = 9.6, 3.3 Hz, 2H), 2.49 – 2.38 (m, 1H), 2.30 – 2.20 (m, 1H), 2.06 (s, 3H).
^{13}**C NMR** (151 MHz, $CDCl_3$) δ 169.9, 152.5, 145.1, 130.1, 129.7 (q, *J* = 32.5 Hz), 128.1, 126.7, 126.6, 125.8 (q, *J* = 3.7 Hz), 124.2 (d, *J* = 271.9 Hz), 122.9, 113.4, 66.3, 51.8, 34.6, 23.4.
^{19}**F NMR** (376 MHz, $CDCl_3$) δ −63.04.
HRMS (ESI+) m/z $[M+H]^+$ calcd. for $C_{18}H_{16}{}^{35}Cl_2F_3NO_2$ 406.0583, found 406.0587.

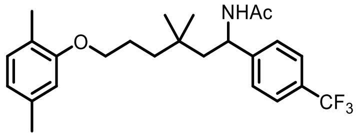

***N*-(6-(2,5-dimethylphenoxy)-3,3-dimethyl-1-(4-(trifluoromethyl)phenyl)hexyl)acetamide (35).** Prepared according to GP3 from **1ak** (120 mg, 0.3 mmol, 1.0 equiv.) and **2a** (54 μL, 0.36 mmol, 1.2 equiv.). Purified via flash column chromatography on silica gel (Pentane:Ethyl Acetate 2:1) to afford the product as a yellowish oil (70 mg, 54% yield).

^{1}**H NMR** (400 MHz, $CDCl_3$) δ 7.57 (d, *J* = 8.0 Hz, 2H), 7.39 (d, *J* = 8.0 Hz, 2H), 7.01 (d, *J* = 7.5 Hz, 1H), 6.67 (d, *J* = 7.4 Hz, 1H), 6.61 (s, 1H), 5.79 (d, *J* = 8.1 Hz, 1H), 5.16 (td, *J* = 7.7, 5.4 Hz, 1H), 3.89 (t, *J* = 6.4 Hz, 2H), 2.31 (s, 3H), 2.18 (s, 3H), 1.96 (s, 3H), 1.76 – 1.68 (m, 4H), 1.48 – 1.42 (m, 2H), 0.97 (s, 6H).
^{13}**C NMR** (151 MHz, $CDCl_3$) δ 169.0, 157.1, 148.5, 136.7, 130.5, 129.5 (q, *J* = 32.4 Hz), 126.8, 125.8 (q, *J* = 3.8 Hz), 124.2 (q, *J* = 273.3 Hz), 123.3, 120.9, 112.2, 68.5, 50.5, 48.6, 39.0, 33.4, 27.6, 24.4, 23.6, 21.5, 16.0.
^{19}**F NMR** (376 MHz, $CDCl_3$) δ −62.98.
HRMS (EI+) m/z $[M]^+$ calcd. for $C_{25}H_{32}F_3NO_2$ 435.2380, found 435.2368.

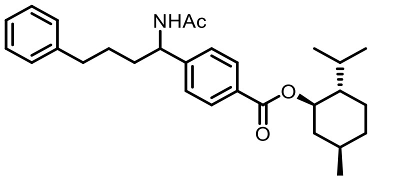

***(1R,2S,5R)-2-Isopropyl-5-methylcyclohexyl 4-(1-acetamido-4-phenylbutyl)benzoate* (36).** Prepared according to GP3 from **1a** (89 mg, 0.3 mmol, 1.0 equiv.) and **2m** (103 mg, 0.36 mmol, 1.2 equiv.). Purified via flash column chromatography on silica gel (Pentane:Ethyl Acetate 1:1) to afford the two diastereomers as an inseparable mixture as a yellowish oil (84 mg, 63% yield). *d.r.* ratio of **36** was determined via ^{1}H NMR to be 1:1.

^{1}**H NMR** (400 MHz, $CDCl_3$) δ 7.99 (d, *J* = 8.3 Hz, 2H), 7.31 (d, *J* = 8.0 Hz, 2H), 7.25 (d, *J* = 7.9 Hz, 2H), 7.19 – 7.15 (m, 1H), 7.11 (d, *J* = 1.2 Hz, 2H), 5.91 (d, *J* = 7.9 Hz, 1H), 5.02 (q, *J* = 7.6 Hz, 1H), 4.92 (td, *J* = 10.9, 4.4 Hz, 1H), 2.62 (t, *J* = 7.4 Hz, 2H), 2.14 – 2.07 (m, 1H), 1.96 (s, 1.5H), 1.96 (s, 1.5H), 1.83 – 1.80 (m, 2H), 1.73 – 1.70 (m, 3H), 1.57 – 1.53 (m, 4H), 1.12 – 1.07 (m, 2H), 0.92 (dd, *J* = 6.8, 5.5 Hz, 7H), 0.78 (d, *J* = 6.9 Hz, 3H).
^{13}**C NMR** (101 MHz, $CDCl_3$) δ 169.4, 165.9, 147.4, 141.8, 130.1, 130.0, 128.5, 126.6, 126.6, 126.0, 75.0, 53.3, 47.4, 41.1, 35.6, 35.5, 34.4, 31.6, 28.0, 26.6, 23.8, 23.5, 22.2, 20.9, 16.6.

HRMS (EI+) m/z $[M]^+$ calcd. for $C_{29}H_{39}NO_3$ 449.2924, found 449.2919.

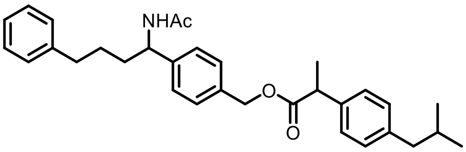

4-(1-Acetamido-4-phenylbutyl)benzyl 2-(4-isobutylphenyl)propanoate **(37)*.*** Prepared according to GP3 from **1a** (89 mg, 0.3 mmol, 1.0 equiv.) and **2n** (116 mg, 0.36 mmol, 1.2 equiv.). Purified via flash column chromatography on silica gel (Pentane:Ethyl Acetate 1:1) to afford the two diastereomers as an inseparable mixture as a yellowish oil (81 mg, 56% yield). *d.r.* ratio of **37** was determined via ^{1}H NMR analysis to be 1:1.

^{1}H NMR (400 MHz, $CDCl_3$) δ 7.29 – 7.24 (m, 2H), 7.22 – 7.17 (m, 6H), 7.14 – 7.04 (m, 5H), 5.71 (d, *J* = 8.3 Hz, 1H), 5.07 (q, *J* = 12.6 Hz, 2H), 4.97 (q, *J* = 7.6 Hz, 1H), 3.74 (q, *J* = 7.1 Hz, 1H), 2.62 (t, *J* = 7.5 Hz, 2H), 2.46 (d, *J* = 7.2 Hz, 2H), 1.95 (s, 3H), 1.85 – 1.78 (m, 3H), 1.70 – 1.60 (m, 2H), 1.51 (d, *J* = 7.2 Hz, 3H), 0.91 (d, *J* = 6.6 Hz, 6H).
^{13}C NMR (101 MHz, $CDCl_3$) δ 174.7, 169.3, 142.1, 142.0, 140.7, 137.7, 135.4, 129.4, 128.5, 128.5, 128.3, 127.3, 126.8, 126.0, 66.1, 53.2, 45.3, 45.1, 35.6, 35.6, 30.3, 28.0, 23.5, 22.5, 18.6.
HRMS (ESI+) m/z $[M+H]^+$ calcd. for $C_{32}H_{39}NO_3$ 486.3003, found 486.3010.

10.3 Characterization data of Heck-type products

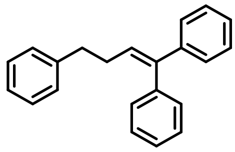

But-1-ene-1,1,4-triyltribenzene **(38).** Prepared according to GP4 from **1a** (89 mg, 0.3 mmol, 1.0 equiv.) and **3a** (63 μL, 0.36 mmol, 1.2 equiv.). Purified via flash column chromatography on silica gel (from Pentane to Pentane:Ethyl Acetate 95:5) to afford the product as a white solid (53 mg, 62% yield).

Characterization data are in accordance with literature.[107]
^{1}H NMR (400 MHz, $CDCl_3$) δ 7.36 – 7.28 (m, 4H), 7.24 – 7.08 (m, 11H), 6.13 (t, *J* = 7.4 Hz, 1H), 2.76 (t, *J* = 7.7 Hz, 2H), 2.45 (q, *J* = 7.6 Hz, 2H).
^{13}C NMR (101 MHz, $CDCl_3$) δ 142.8, 142.4, 141.8, 140.2, 130.0, 129.0, 128.7, 128.4, 128.3, 128.2, 127.4, 127.0, 127.0, 126.0, 36.3, 31.8.
HRMS (EI+) m/z $[M]^+$ calcd. for $C_{22}H_{20}$ 284.1560, found 284.1556.

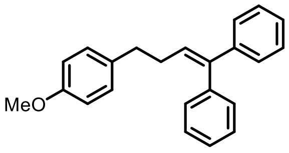

(4-(4-Methoxyphenyl)but-1-ene-1,1-diyl)dibenzene **(39).** Prepared according to GP4 from **1k** (97 mg, 0.3 mmol, 1.0 equiv.) and **3a** (63 μL, 0.36 mmol, 1.2 equiv.). Purified via flash column chromatography on silica gel (Pentane:Ethyl Acetate 99:1) to afford the product as a white solid (54 mg, 57% yield).

Characterization data are in accordance with literature.[108]
^{1}H NMR (400 MHz, $CDCl_3$) δ 7.37 – 7.27 (m, 4H), 7.26 – 7.15 (m, 4H), 7.11 – 7.03 (m, 4H), 6.81 (d, *J* = 8.6 Hz, 2H), 6.11 (t, *J* = 7.4 Hz, 1H), 3.78 (s, 3H), 2.70 (t, *J* = 7.4 Hz, 2H), 2.46 – 2.33 (q, *J* = 7.3 Hz, 2H).
^{13}C NMR (101 MHz, $CDCl_3$) δ 157.9, 142.8, 142.3, 140.3, 133.9, 130.0, 129.5, 129.1, 128.3, 128.2, 127.4, 127.0, 127.0, 113.9, 55.4, 35.4, 32.0.
HRMS (EI+) m/z $[M]^+$ calcd. for $C_{23}H_{22}O$ 314.1665, found 314.1661.

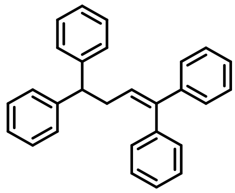

But-1-ene-1,1,4,4-tetrayltetrabenzene **(40).** Prepared according to GP4 from **1b** (111 mg, 0.3 mmol, 1.0 equiv.) and **3a** (63 μL, 0.36 mmol, 1.2 equiv.). Purified via flash column chromatography

on silica gel (from Pentane to Pentane:Ethyl Acetate 95:5) to afford the product as a colorless oil (63 mg, 60% yield).

^{1}H NMR (400 MHz, $CDCl_3$) δ 7.37 – 7.28 (m, 4H), 7.18 (m, 12H), 7.09 – 7.05 (m, 4H), 5.99 (t, *J* = 7.2 Hz, 1H), 4.10 (t, *J* = 7.9 Hz, 1H), 2.88 (t, *J* = 7.5 Hz, 2H).
^{13}C NMR (101 MHz, $CDCl_3$) δ 144.5, 142.9, 142.8, 140.2, 130.0, 128.5, 128.3, 128.2, 128.1, 128.0, 127.4, 127.1, 127.0, 126.3, 51.8, 36.0.
HRMS (EI+) m/z $[M]^+$ calcd. for $C_{28}H_{28}$ 360.1873, found 360.1872.

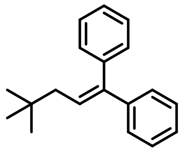

(4,4-Dimethylpent-1-ene-1,1-diyl)dibenzene* (41)*. Prepared according to GP4 from **1p** (94 mg, 0.36 mmol, 1.2 equiv.) and **3a** (52 µL, 0.3 mmol, 1.0 equiv.). Purified via flash column chromatography on silica gel (Pentane) to afford the product as a colorless oil (31 mg, 42% yield).

Characterization data are in accordance with literature.[109]
^{1}H NMR (400 MHz, $CDCl_3$) δ 7.39 – 7.28 (m, 3H), 7.27 – 7.13 (m, 7H), 6.20 (t, *J* = 7.6 Hz, 1H), 2.03 (d, *J* = 7.6 Hz, 2H), 0.92 (s, 9H).
^{13}C NMR (101 MHz, $CDCl_3$) δ 143.3, 142.7, 140.6, 130.3, 128.4, 128.2, 127.5, 127.3, 126.9, 126.9, 43.5, 31.8, 29.6.
HRMS (EI+) m/z $[M]^+$ calcd. for $C_{19}H_{22}$ 250.1716, found 250.1721.

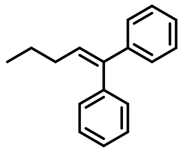

Pent-1-ene-1,1-diyldibenzene* (42)*. Prepared according to GP4 from **1o** (84 mg, 0.36 mmol, 1.2 equiv.) and **3a** (52 µL, 0.3 mmol, 1.0 equiv.). Purified via flash column chromatography on silica gel (Pentane) to afford the product as a colorless oil (35 mg, 52% yield).

Characterization data are in accordance with literature.[110]
^{1}H NMR (300 MHz, $CDCl_3$) δ 7.42 – 7.27 (m, 6H), 7.26 – 7.18 (m, 4H), 6.12 (t, *J* = 7.4 Hz, 1H), 2.12 (q, *J* = 7.4 Hz, 2H), 1.50 (h, *J* = 7.4 Hz, 2H), 0.93 (t, *J* = 7.4 Hz, 3H).
^{13}C NMR (101 MHz, $CDCl_3$) δ 143.1, 141.7, 140.5, 130.3, 130.1, 128.2, 128.2, 127.4, 126.9, 126.9, 32.0, 23.3, 14.0.
HRMS (EI+) m/z $[M]^+$ calcd. for $C_{17}H_{18}$ 222.1403, found: 222.1407.

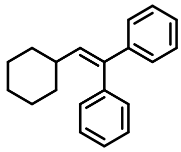

***(2-Cyclohexylethene-1,1-diyl)dibenzene* (43).** Prepared according to GP4 from **1r** (98 mg, 0.36 mmol, 1.2 equiv.) and **3a** (52 µL, 0.3 mmol, 1.0 equiv.). Purified via flash column chromatography on silica gel (Pentane) to afford the product as a colorless oil (63 mg, 81% yield).

Characterization data are in accordance with literature.[111]
^{1}H NMR (400 MHz, $CDCl_3$) δ 7.40 – 7.29 (m, 3H), 7.26 – 7.15 (m, 7H), 5.91 (d, *J* = 10.0 Hz, 1H), 2.20 – 2.03 (m, 1H), 1.72 – 1.58 (m, 5H), 1.21 – 1.13 (m, 5H).
^{13}C NMR (101 MHz, $CDCl_3$) δ 143.1, 140.4, 139.7, 136.1, 129.9, 128.3, 128.2, 127.3, 126.9, 126.9, 38.5, 33.5, 26.1, 25.7.
HRMS (EI+) m/z $[M]^+$ calcd. for $C_{20}H_{22}$ 262.1716, found 262.1720.

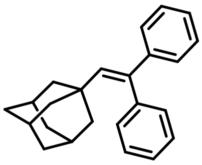

***1-(2,2-Diphenylvinyl)adamantane* (44).** Prepared according to GP4 from **1u** (117 mg, 0.36 mmol, 1.2 equiv.) and **3a** (52 µL, 0.3 mmol, 1.0 equiv.). Purified via flash column chromatography on silica gel (Pentane) to afford the product as a white solid (67 mg, 71% yield).

Characterization data are in accordance with literature.[109]
^{1}H NMR (300 MHz, $CDCl_3$) δ 7.36 – 7.30 (m, 3H), 7.22 – 7.16 (m, 7H), 5.85 (s, 1H), 1.85 (m, 3H), 1.62 – 1.56 (m, 12H).
^{13}C NMR (75 MHz, $CDCl_3$) δ 144.4, 141.2, 140.8, 138.9, 130.5, 128.1, 127.7, 127.0, 126.8, 126.6, 43.3, 36.8, 36.5, 28.7.
HRMS (EI+) m/z $[M]^+$ calcd. for $C_{24}H_{26}$ 314.2029, found 314.2034.

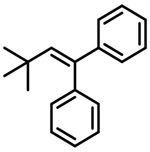

***(3,3-Dimethylbut-1-ene-1,1-diyl)dibenzene* (45).** Prepared according to GP4 from **1v** (89 mg, 0.36 mmol, 1.2 equiv.) and **3a** (52 µL, 0.3 mmol, 1.0 equiv.). Purified via flash column chromatography on silica gel (Pentane) to afford the product as a colorless oil (56 mg, 80% yield).

Characterization data are in accordance with literature.[109]
^{1}H NMR (400 MHz, $CDCl_3$) δ 7.36 – 7.28 (m, 3H), 7.22 – 7.16 (m, 7H), 6.09 (s, 1H), 0.97 (s, 9H).
^{13}C NMR (101 MHz, $CDCl_3$) δ 144.2, 140.9, 140.3, 139.2, 130.5, 128.1, 127.9, 127.0, 126.9, 126.7, 34.1, 31.5.
HRMS (EI+) m/z $[M]^+$ calcd. for $C_{18}H_{20}$ 236.1560, found 236.1566.

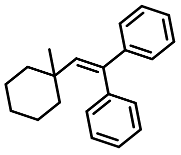

***(2-(1-Methylcyclohexyl)ethene-1,1-diyl)dibenzene* (46).** Prepared according to GP4 from **1z** (103 mg, 0.36 mmol, 1.2 equiv.) and **3a** (52 µL, 0.3 mmol, 1.0 equiv.). Purified via flash column chromatography on silica gel (Pentane) to afford the product as a colorless oil (66 mg, 80% yield).

Characterization data are in accordance with literature.[110]
^{1}H NMR (400 MHz, $CDCl_3$) δ 7.38 – 7.32 (m, 3H), 7.29 – 7.22 (m, 7H), 6.07 (s, 1H), 1.51 – 1.44 (m, 7H), 1.33 – 1.21 (m, 1H), 1.14 – 1.09 (m, 2H), 1.04 (s, 3H).
^{13}C NMR (101 MHz, $CDCl_3$) δ 144.6, 141.1, 140.5, 139.3, 130.1, 128.1, 127.9, 127.0, 126.9, 126.7, 39.6, 37.3, 29.6, 26.3, 23.2.
HRMS (EI+) m/z $[M]^+$ calcd. for $C_{21}H_{24}$ 276.1873, found 276.1868.

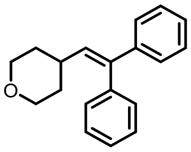

***4-(2,2-Diphenylvinyl)tetrahydro-2H-pyran* (47).** Prepared according to GP4 from **1aa** (82 mg, 0.3 mmol, 1.0 equiv.) and **3a** (63 µL, 0.36 mmol, 1.2 equiv.). Purified via flash column chromatography on silica gel (Pentane:Ethyl Acetate 20:1) to afford the product as a white solid (48 mg, 60% yield).

Characterization data are in accordance with literature.[109]
^{1}H NMR (400 MHz, $CDCl_3$) δ 7.44 – 7.32 (m, 3H), 7.27 – 7.15 (m, 7H), 5.91 (d, *J* = 9.8 Hz, 1H), 3.92 (m, 2H), 3.36 – 3.25 (m, 2H), 2.38 (m, 1H), 1.63 – 1.55 (m, 4H).
^{13}C NMR (101 MHz, $CDCl_3$) δ 142.5, 141.1, 140.3, 133.8, 129.8, 128.4, 128.3, 127.3, 127.2, 127.2, 67.5, 35.7, 33.0.
HRMS (EI+) m/z $[M]^+$ calcd. for $C_{19}H_{20}O$ 264.1509, found 264.1517.

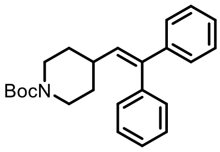

***Tert-butyl 4-(2,2-diphenylvinyl)piperidine-1-carboxylate* (48).** Prepared according to GP4 from **1ab** (112 mg, 0.3 mmol, 1.0 equiv.) and **3b** (63 µL, 0.36 mmol, 1.2 equiv.). Purified via flash column chromatography on silica gel (Pentane:Ethyl Acetate 20:1) to afford the product as a colorless oil (76 mg, 69% yield).

Characterization data are in accordance with literature.[113]
^{1}H NMR (400 MHz, $CDCl_3$) δ 7.48 – 7.36 (m, 3H), 7.33 – 7.19 (m, 7H), 5.92 (d, *J* = 9.8 Hz, 1H), 4.22 – 4.03 (m, 2H), 2.66 (t, *J* = 13 Hz, 2H), 2.40 – 2.25 (m, 1H), 1.67 (m, 2H), 1.51 (s, 9H), 1.43 (m, 2H).
^{13}C NMR (101 MHz, $CDCl_3$) δ 155.0, 142.5, 141.2, 140.3, 133.5, 129.7, 128.4, 128.2, 127.3, 127.2, 127.2, 79.4, 43.4, 36.7, 32.3, 28.6.
HRMS (ESI+) m/z $[M+Na]^+$ calcd. for $C_{24}H_{29}NO_2$ 386.2090, found 386.2094.

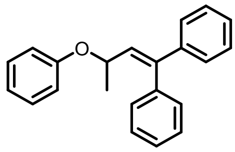

***(3-Phenoxybut-1-ene-1,1-diyl)dibenzene* (49).** Prepared according to GP4 from **1ah** (93 mg, 0.3 mmol, 1.0 equiv.) and **3a** (52 μL, 0.3 mmol, 1.0 equiv.). Purified via flash column chromatography on silica gel (from Pentane to Pentane:Ethyl Acetate 20:1) to afford the product as a colorless oil (73 mg, 81% yield).

Characterization data are in accordance with literature.[114]
^{1}H NMR (400 MHz, $CDCl_3$) δ 7.49 – 7.36 (m, 3H), 7.28 – 7.12 (m, 9H), 6.95 – 6.85 (m, 1H), 6.73 (d, *J* = 6.6 Hz, 2H), 6.12 (d, *J* = 8.7 Hz, 1H), 4.85 (dq, *J* = 8.9, 6.3 Hz, 1H), 1.54 (d, *J* = 6.3 Hz, 3H).
^{13}C NMR (101 MHz, $CDCl_3$) δ 157.8, 143.6, 141.6, 139.4, 130.4, 129.8, 129.3, 128.5, 128.3, 127.8, 127.8, 127.6, 120.8, 116.4, 71.8, 22.0.
HRMS (ESI+) m/z $[M]^+$ calcd. for $C_{22}H_{20}O$ 300.1509, found 300.1505.

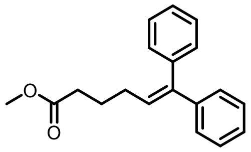

***Methyl 6,6-diphenylhex-5-enoate* (50).** Prepared according to GP4 from **1ag** (87 mg, 0.3 mmol, 1.0 equiv.) and **3a** (63 μL, 0.36 mmol, 1.2 equiv.). Purified via flash column chromatography on silica gel (Pentane:Ethyl Acetate 40:1) to afford the product as a colorless oil (46 mg, 55% yield).

Characterization data are in accordance with literature.[115]
^{1}H NMR (400 MHz, $CDCl_3$) δ 7.44 – 7.23 (m, 3H), 7.27 – 7.12 (m, 7H), 6.06 (t, *J* = 7.5 Hz, 1H), 3.63 (s, 3H), 2.31 (t, *J* = 7.5 Hz, 2H), 2.16 (q, *J* = 7.4 Hz, 2H), 1.80 (p, *J* = 7.3 Hz, 2H).
^{13}C NMR (101 MHz, $CDCl_3$) δ 174.1, 142.7, 142.7, 140.1, 130.0, 128.7, 128.3, 128.2, 127.3, 127.1, 51.6, 33.7, 29.3, 25.3.
HRMS (ESI+) m/z $[M+H]^+$ calcd. for $C_{19}H_{20}O_2$ 281.1536, found 281.1541.

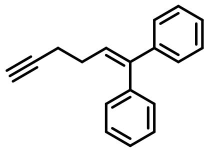

***Hex-1-en-5-yne-1,1-diyldibenzene* (51).** Prepared according to GP4 from **1ae** (88 mg, 0.36 mmol, 1.2 equiv.) and **3a** (52 μL, 0.3 mmol, 1.0 equiv.). Purified via flash column chromatography on silica gel (Pentane:Ethyl Acetate 99:1) to afford the product as a colorless oil (33 mg, 47% yield).

Characterization data are in accordance with literature.[116]
^{1}H NMR (400 MHz, $CDCl_3$) δ 7.41 – 7.31 (m, 3H), 7.28 – 7.18 (m, 7H), 6.16 (t, *J* = 7.0 Hz, 1H), 2.41 – 2.32 (m, 4H), 1.99 (t, *J* = 2.5 Hz, 1H).
^{13}C NMR (101 MHz, $CDCl_3$) δ 143.2, 142.6, 140.0, 130.0, 128.4, 128.2, 127.6, 127.5, 127.2, 127.2, 84.0, 69.0, 28.9, 19.1.
HRMS (EI+) m/z $[M]^+$ calcd. for $C_{18}H_{16}$ 232.1247, found 232.1246.

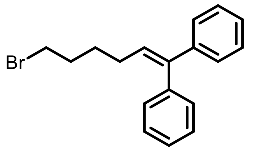

***(6-Bromohex-1-ene-1,1-diyl)dibenzene* (52).** Prepared according to GP4 from **1ad** (117 mg, 0.36 mmol, 1.2 equiv.) and **3a** (52 μL, 0.3 mmol, 1.0 equiv.). Purified via flash column chromatography on silica gel (Pentane:Ethyl Acetate 99:1) to afford the product as a yellowish oil (52 mg, 55% yield).

Characterization data are in accordance with literature.[117]
^{1}H NMR (400 MHz, $CDCl_3$) δ 7.45 – 7.26 (m, 7H), 7.27 – 7.17 (m, 3H), 6.10 (t, *J* = 7.5 Hz, 1H), 3.38 (t, *J* = 6.8 Hz, 2H), 2.18 (q, *J* = 7.4 Hz, 2H), 1.89 (p, *J* = 6.9 Hz, 2H), 1.62 (t, *J* = 7.5 Hz, 2H).
^{13}C NMR (101 MHz, $CDCl_3$) δ 142.7, 142.4, 140.2, 130.0, 129.2, 128.4, 128.2, 127.3, 127.1, 127.1, 33.8, 32.4, 28.9, 28.5.
HRMS (EI+) m/z $[M]^+$ calcd. for $C_{18}H_{19}{}^{79}Br$ 314.0665, found 314.0657.

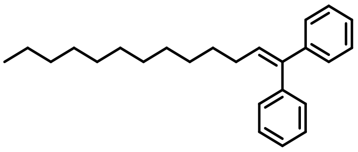

***Tridec-1-ene-1,1-diyldibenzene* (53).** Prepared according to GP4 from **1ao** (124 mg, 0.36 mmol, 1.2 equiv.) and **3a** (52 μL, 0.3 mmol, 1.0 equiv.). Purified via flash column chromatography on silica gel (Pentane) to afford the product as a colorless oil (67 mg, 64% yield).

^{1}H NMR (400 MHz, $CDCl_3$) δ 7.42 – 7.27 (m, 4H), 7.23 – 7.18 (m, 6H), 6.10 (t, *J* = 7.5 Hz, 1H), 2.12 (q, *J* = 7.4 Hz, 2H), 1.49 – 1.40 (m, 2H), 1.27 (m, 16H), 0.89 (t, *J* = 6.8 Hz, 3H).
^{13}C NMR (101 MHz, $CDCl_3$) δ 143.1, 141.5, 140.5, 130.5, 130.1, 128.2, 128.2, 127.3, 126.9, 126.9, 32.1, 30.1, 29.9, 29.8, 29.8, 29.8, 29.6, 29.5, 29.4, 22.9, 14.3.
HRMS (EI+) m/z $[M]^+$ calcd. for $C_{25}H_{34}$ 334.2655, found 334.2650.

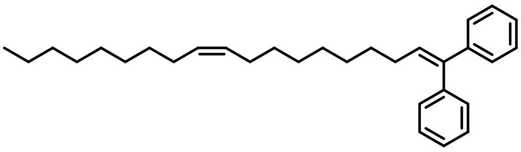

***(Z)-Nonadeca-1,10-diene-1,1-diyldibenzene* (54).** Prepared according to GP4 from **1ap** (154 mg, 0.36 mmol, 1.2 equiv.) and **3a** (52 μL, 0.3 mmol, 1.0 equiv.). Purified via flash column chromatography on silica gel (Pentane) to afford the product as a colorless oil (60 mg, 48% yield).

^{1}H NMR (400 MHz, $CDCl_3$) δ 7.39 – 7.29 (m, 3H), 7.25 – 7.18 (m, 7H), 6.09 (t, *J* = 7.5 Hz, 1H), 5.37 – 5.34 (m, 2H), 2.11 (q, *J* = 7.4 Hz, 2H), 2.04 – 1.99 (m, 4H), 1.44 (t, *J* = 7.3 Hz, 2H), 1.28 (m, 20H), 0.89 (t, *J* = 6.8 Hz, 3H).
^{13}C NMR (101 MHz, $CDCl_3$) δ 143.1, 141.6, 140.5, 130.5, 130.1, 130.0. 130.0, 128.2, 128.2, 127.3, 126.9, 126.9, 32.1, 30.1, 29.9, 29.9, 29.7, 29.5, 29.5, 29.4, 29.4, 27.4, 27.4, 22.8, 14.3.
HRMS (EI+) m/z $[M]^+$ calcd. for $C_{31}H_{44}$ 416.3438, found 416.3434.

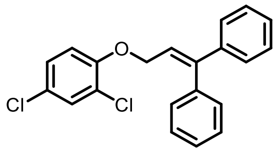

***(3-(2,4-Dichlorophenoxy)prop-1-ene-1,1-diyl)dibenzene* (55).** Prepared according to GP4 from **1aj** (109 mg, 0.3 mmol, 1.0 equiv.) and **3a** (63 µL, 0.36 mmol, 1.2 equiv.). Purified via flash column chromatography on silica gel (from Pentane:Ethyl Acetate 99:1 to 98:2) to afford the product as a colorless oil (58 mg, 55% yield).

^{1}H NMR (400 MHz, $CDCl_3$) δ 7.38 – 7.34 (m, 3H), 7.20 – 7.17 (m, 8H), 7.06 (dd, *J* = 8.8, 2.6 Hz, 1H), 6.63 (d, *J* = 8.8 Hz, 1H), 6.29 (t, *J* = 6.6 Hz, 1H), 4.62 (d, *J* = 6.6 Hz, 2H).
^{13}C NMR (101 MHz, $CDCl_3$) δ 153.1, 146.6, 141.4, 138.9, 130.1, 130.1, 129.8, 128.6, 128.4, 128.1, 127.9, 127.6, 125.9, 124.1, 122.9, 114.9, 67.5.
HRMS (EI+) m/z $[M]^+$ calcd. for $C_{21}H_{16}{}^{35}Cl_2O$ 354.0573, found 354.0573.

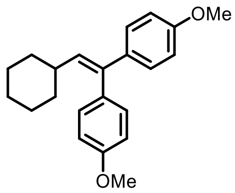

***4,4'-(2-Cyclohexylethene-1,1-diyl)bis(methoxybenzene)* (56).** Prepared according to GP4 from **1r** (98 mg, 0.36 mmol, 1.2 equiv.) and **3c** (72 mg, 0.3 mmol, 1.0 equiv.). Purified via flash column chromatography on silica gel (Pentane:Ethyl Acetate 30:1) to afford the product as a colorless oil (40 mg, 42% yield).

Characterization data are in accordance with literature.[111]
^{1}H NMR (400 MHz, $CDCl_3$) δ 7.15 (d, *J* = 8.8 Hz, 2H), 7.10 (d, *J* = 8.7 Hz, 2H), 6.91 (d, *J* = 8.7 Hz, 2H), 6.80 (d, *J* = 8.8 Hz, 2H), 5.78 (d, *J* = 10.0 Hz, 1H), 3.85 (s, 3H), 3.79 (s, 3H), 2.16 (m, 1H), 1.73 – 1.63 (m, 5H), 1.18 (m, 5H).
^{13}C NMR (101 MHz, $CDCl_3$) δ 158.6, 158.4, 138.6, 136.1, 134.3, 133.2, 130.8, 128.3, 113.5, 113.4, 55.3, 55.2, 38.3, 33.5, 25.7.
HRMS (EI+) m/z $[M]^+$ calcd. for $C_{22}H_{26}O_2$ 322.1927, found 322.1919.

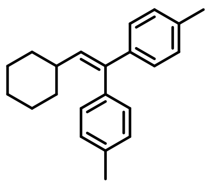

***4,4'-(2-Cyclohexylethene-1,1-diyl)bis(methylbenzene)* (57).** Prepared according to GP4 from **1r** (98 mg, 0.36 mmol, 1.2 equiv.) and **3b** (63 mg, 0.3 mmol, 1.0 equiv.). Purified via flash column chromatography on silica gel (Pentane) to afford the product as a white solid (61 mg, 69% yield).

Characterization data are in accordance with literature.[111]
^{1}H NMR (400 MHz, $CDCl_3$) δ 7.19 (d, *J* = 7.8 Hz, 2H), 7.16 – 7.04 (m, 6H), 5.85 (d, *J* = 10.0 Hz, 1H), 2.41 (s, 3H), 2.33 (s, 3H), 2.17 (m, 1H), 1.75 – 1.56 (m, 5H), 1.19 (m, 5H).

^{13}C NMR (101 MHz, $CDCl_3$) δ 140.6, 139.4, 137.9, 136.5, 136.4, 135.2, 129.8, 128.9, 128.8, 127.3, 38.4, 33.6, 26.2, 25.8, 21.4, 21.2.
HRMS (EI+) m/z $[M]^+$ calcd. for $C_{22}H_{26}$ 290.2029, found 290.2022.

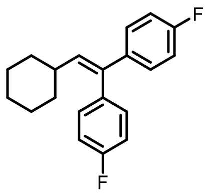

***4,4'-(2-Cyclohexylethene-1,1-diyl)bis(fluorobenzene)* (58).** Prepared according to GP4 from **1r** (98 mg, 0.36 mmol, 1.2 equiv.) and **3d** (65 mg, 0.3 mmol, 1.0 equiv.). Purified via flash column chromatography on silica gel (Pentane) to afford the product as a colorless oil (72 mg, 81% yield).

Characterization data are in accordance with literature.[111]
^{1}H NMR (400 MHz, $CDCl_3$) δ 7.14 (td, *J* = 8.8, 5.5 Hz, 4H), 7.07 (t, *J* = 8.7 Hz, 2H), 6.94 (t, *J* = 8.7 Hz, 2H), 5.85 (d, *J* = 10.0 Hz, 1H), 2.13 – 2.05 (m, 1H), 1.72 – 1.63 (m, 5H), 1.22 – 1.17 (m, 5H).
^{13}C NMR (101 MHz, $CDCl_3$) δ 162.1 (d, *J* = 244 Hz), 162.0 (d, *J* = 244 Hz), 139.0 (d, *J* = 3.3 Hz), 137.8, 136.4 (d, *J* = 3.5 Hz), 136.3, 131.4 (d, *J* = 7.8 Hz), 128.8 (d, *J* = 7.8 Hz), 115.3 (d, *J* = 21.2 Hz), 115.0 (d, *J* = 21.3 Hz), 38.5, 33.4, 26.1, 25.7.
^{19}F NMR (376 MHz, $CDCl_3$) δ −115.95, −116.55.
HRMS (EI+) m/z $[M]^+$ calcd. for $C_{20}H_{20}F_2$ 298.1528, found 298.1519.

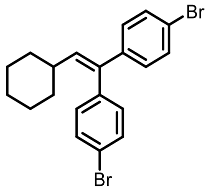

***4,4'-(2-Cyclohexylethene-1,1-diyl)bis(bromobenzene)* (59).** Prepared according to GP4 from **1r** (98 mg, 0.36 mmol, 1.2 equiv.) and **3e** (101 mg, 0.3 mmol, 1.0 equiv.). Purified via flash column chromatography on silica gel (Pentane) to afford the product as a white solid (104 mg, 84% yield).

Characterization data are in accordance with literature.[111]
^{1}H NMR (400 MHz, $CDCl_3$) δ 7.50 (d, *J* = 8.4 Hz, 2H), 7.36 (d, *J* = 8.6 Hz, 2H), 7.06 – 7.02 (m, 4H), 5.89 (d, *J* = 10.1 Hz, 1H), 2.12 – 2.02 (m, 1H), 1.71 – 1.63 (m, 5H), 1.17 (m, 5H).
^{13}C NMR (101 MHz, $CDCl_3$) δ 141.5, 139.0, 137.7, 137.2, 131.7, 131.6, 131.3, 128.9, 121.3, 121.1, 38.6, 33.3, 26.0, 25.6.
HRMS (EI+) m/z $[M]^+$ calcd. for $C_{20}H_{20}{}^{79}Br_2$ 417.9926, found 417.9920.

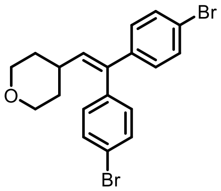

4-(2,2-Bis(4-bromophenyl)vinyl)tetrahydro-2H-pyran **(60).** Prepared according to GP4 from **1aa** (99 mg, 0.36 mmol, 1.2 equiv.) and **3e** (101 mg, 0.3 mmol, 1.0 equiv.). Purified via flash column chromatography on silica gel (*n*-Hexane:Ethyl Acetate 10:1) to afford the product as a white solid (84 mg, 67% yield).

^{1}H NMR (400 MHz, $CDCl_3$) δ 7.52 (d, *J* = 8.4 Hz, 2H), 7.38 (d, *J* = 8.6 Hz, 2H), 7.03 (t, *J* = 8.6 Hz, 4H), 5.89 (d, *J* = 10 Hz, 1H), 3.94 – 3.90 (m, 2H), 3.33 – 3.26 (m, 2H), 2.31 (m, 1H), 1.55 (m, 4H).
^{13}C NMR (101 MHz, $CDCl_3$) δ 140.9, 139.1, 138.6, 134.8, 131.8, 131.4, 131.4, 128.9, 121.6, 121.5, 67.3, 35.9, 32.8.
HRMS (EI+) m/z $[M]^+$ calcd. for $C_{19}H_{18}{}^{79}Br_2O$ 419.9719, found 419.9707.

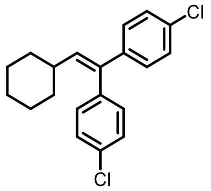

4,4'-(2-Cyclohexylethene-1,1-diyl)bis(chlorobenzene) **(61).** Prepared according to GP4 from **1r** (98 mg, 0.36 mmol, 1.2 equiv.) and **3f** (74 mg, 0.3 mmol, 1.0 equiv.). Purified via flash column chromatography on silica gel (Pentane) to afford the product as a white solid (89 mg, 89% yield).

Characterization data are in accordance with literature.[111]
^{1}H NMR (400 MHz, $CDCl_3$) δ 7.35 (d, *J* = 8.4 Hz, 2H), 7.21 (d, *J* = 8.6 Hz, 2H), 7.09 (dd, *J* = 8.5, 6.2 Hz, 4H), 5.89 (d, *J* = 10.1 Hz, 1H), 2.14 – 2.01 (m, 1H), 1.73 – 1.59 (m, 5H), 1.17 (m, 5H).
^{13}C NMR (101 MHz, $CDCl_3$) δ 141.1, 138.6, 137.6, 137.1, 133.1, 132.9, 131.2, 128.7, 128.6, 128.4, 38.6, 33.3, 26.0, 25.7.
HRMS (EI+) m/z $[M]^+$ calcd. for $C_{20}H_{20}{}^{35}Cl_2$ 330.0937, found 330.0940.

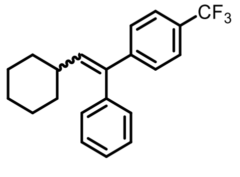

1-(2-Cyclohexyl-1-phenylvinyl)-4-(trifluoromethyl)benzene **(62).** Prepared according to GP4 from **1r** (98 mg, 0.36 mmol, 1.2 equiv.) and **3g** (74 mg, 0.3 mmol, 1.0 equiv.). Purified via flash column chromatography on silica gel (Pentane) to afford the two diastereomers as an inseparable mixture as a colorless oil (90 mg, 91% yield). *d.r.* ratio of **62** was determined via ^{1}H NMR to be 1:1.

^{1}H NMR (400 MHz, $CDCl_3$) δ 7.64 (d, *J* = 8.0 Hz, 1H), 7.50 (d, *J* = 8.3 Hz, 1H), 7.43 – 7.34 (m, 1H), 7.31 (d, *J* = 8.2 Hz, 2H), 7.28 – 7.22 (m, 2H), 7.18 (m, 2H), 5.99 (d, *J* = 10 Hz, 0.5H), 5.98 (d, *J* = 10 Hz, 0.5H), 2.19 – 1.93 (m, 1H), 1.74 – 1.61 (m, 5H), 1.27 – 1.11 (m, 5H).

^{13}C NMR (151 MHz, $CDCl_3$) δ 146.6, 144.5, 142.3, 139.9, 138.8, 138.6, 138.2, 137.1, 130.3, 129.8, 129.1 (q, *J* = 32 Hz), 128.9 (q, *J* = 32 Hz), 128.5, 128.4, 127.5, 127.3, 125.3 (q, *J* = 3.8 Hz), 125.1 (q, *J* = 3.7 Hz), 123.6 (q, *J* = 267 Hz), 38.6, 38.5, 33.4, 33.3, 26.1, 26.0, 25.7, 25.6.
^{19}F NMR (376 MHz, $CDCl_3$) δ −62.86, −62.87.
HRMS (EI+) m/z $[M]^+$ calcd. for $C_{21}H_{21}F_3$ 330.1590, found 330.1596.

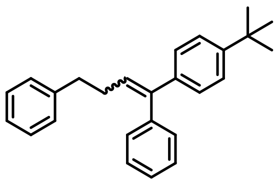

***(1-(4-(Tert-butyl)phenyl)but-1-ene-1,4-diyl)dibenzene* (63).** Prepared according to GP4 from **1a** (89 mg, 0.3 mmol, 1.0 equiv.) and **3h** (85 mg, 0.36 mmol, 1.2 equiv.). Purified via flash column chromatography on silica gel (from Pentane to Pentane:Ethyl Acetate 99:1) to afford the two diastereomers as an inseparable mixture as a colorless oil (64 mg, 63% yield). *d.r.* ratio of **63** was determined via ^{1}H NMR to be 1.4:1.

^{1}H NMR (400 MHz, $CDCl_3$) δ 7.39 – 7.27 (m, 5H), 7.23 – 7.03 (m, 9H), 6.26 – 6.04 (m, 1H), 2.77 (q, *J* = 7.3 Hz, 2H), 2.46 (m, 2H), 1.37 (s, 4H), 1.32 (s, 5H).
^{13}C NMR (101 MHz, $CDCl_3$) δ 150.0, 149.8, 143.2, 142.3, 142.1, 141.9, 141.9, 140.3, 139.7, 137.1, 129.9, 129.6, 128.9, 128.7, 128.4, 128.2, 128.1, 127.5, 126.9, 129.6, 126.9, 125.9, 125.1, 125.1, 36.4, 36.4, 34.7, 34.6, 31.8, 31.6, 31.5.
HRMS (EI+) m/z $[M]^+$ calcd. for $C_{26}H_{28}$ 340.2186, found 340.2189.

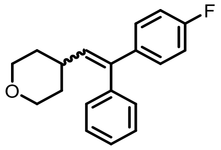

***4-(2-(4-Fluorophenyl)-2-phenylvinyl)tetrahydro-2H-pyran* (64).** Prepared according to GP4 from **1aa** (99 mg, 0.36 mmol, 1.2 equiv.) and **3i** (59 mg, 0.3 mmol, 1.0 equiv.). Purified via flash column chromatography on silica gel (from *n*-Hexane:Ethyl Acetate 9:1 to 8:1) to afford to afford the two diastereomers as an inseparable mixture as a white solid (54 mg, 65% yield). *d.r.* ratio of **64** was determined via ^{1}H NMR to be 1.6:1.

^{1}H NMR (400 MHz, $CDCl_3$) δ 7.39 – 7.36 (m, 2H), 7.18 – 7.14 (m, 6H), 6.94 (t, *J* = 8.8 Hz, 1H), 5.90 (d, *J* = 9.8 Hz, 0.39H), 5.83 (d, *J* = 9.8 Hz, 0.61H), 3.93 – 3.89 (m, 2H), 3.32 – 3.27 (m, 2H), 2.36 (m, 1H), 1.58 – 1.56 (m, 4H).
^{13}C NMR (101 MHz, $CDCl_3$) δ 162.2 (d, *J* = 244 Hz), 162.1 (d, *J* = 244 Hz), 142.3, 140.2, 140.1, 138.7 (d, *J* = 3.5 Hz), 136.1 (d, *J* = 3.5 Hz), 134.2, 133.6, 133.6, 131.3 (d, *J* = 7.8 Hz), 129.7, 128.9 (d, *J* = 7.9 Hz), 128.5, 128.3, 127.4, 127.3, 127.2, 115.4 (d, *J* = 21.3 Hz), 115.0 (d, *J* = 21.4 Hz), 67.5, 35.8, 35.8, 33.0, 33.0.
^{19}F NMR (376 MHz, $CDCl_3$) δ −115.75, −116.29.
HRMS (EI+) m/z $[M]^+$ calcd. for $C_{19}H_{19}FO$ 282.1414, found 282.1420.

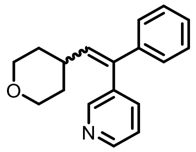

3-(1-Phenyl-2-(tetrahydro-2H-pyran-4-yl)vinyl)pyridine **(65).** Prepared according to GP4 from **1aa** (99 mg, 0.36 mmol, 1.2 equiv.) and **3j** (54 mg, 0.3 mmol, 1.0 equiv.). Purified via flash column chromatography on silica gel (from *n*-Hexane:Ethyl Acetate 2:1 to 1:1) to afford the two diastereomers as an inseparable mixture as a pale yellow solid (62 mg, 78% yield). *d.r.* ratio of **65** was determined via ^{1}H NMR to be 1:1.

^{1}H NMR (400 MHz, $CDCl_3$) δ 8.59 (dd, *J* = 4.9, 1.7 Hz, 0.5H), 8.49 (ddd, *J* = 7.2, 2.3, 0.9 Hz, 1H), 8.45 (dd, *J* = 4.8, 1.6 Hz, 0.5H), 7.46 – 7.31 (m, 5H), 7.19 – 7.13 (m, 2H), 5.99 (d, *J* = 10.0 Hz, 0.5H), 5.92 (d, *J* = 9.8 Hz, 0.5H), 3.94 – 3.87 (m, 2H), 3.30 (m, 2H), 2.50 – 2.35 (m, 0.5H), 2.35 – 2.23 (m, 0.5H), 1.58 (m, 4H).
^{13}C NMR (101 MHz, $CDCl_3$) δ 150.4, 148.7, 148.5, 148.3, 141.7, 139.1, 138.3, 138.1, 137.6, 137.2, 135.9, 135.6, 135.5, 134.5, 129.6, 128.7, 128.5, 127.6, 127.6, 127.3, 123.5, 123.0, 67.3, 67.3, 35.88, 35.8, 32.9, 32.9.
HRMS (ESI+) m/z $[M+H]^+$ calcd. for $C_{18}H_{19}NO$ 266.1539, found 266.1543.

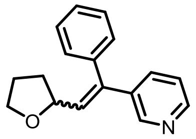

3-(1-Phenyl-2-(tetrahydrofuran-2-yl)vinyl)pyridine **(66).** Prepared according to GP4 from **1ac** (94 mg, 0.36 mmol, 1.2 equiv.) and **3j** (54 mg, 0.3 mmol, 1.0 equiv.). Purified via flash column chromatography on silica gel (from *n*-Hexane:Ethyl Acetate 2:1 to 1:1) to afford the two diastereomers as an inseparable mixture as a yellowish oil (45 mg, 60% yield). *d.r.* ratio of **66** was determined via ^{1}H NMR to be 1:1.

^{1}H NMR (400 MHz, $CDCl_3$) δ 8.59 – 8.52 (m, 1H), 8.49 – 8.46 (m, 1H), 7.56 – 7.46 (m, 1H), 7.40 – 7.27 (m, 3H), 7.24 – 7.16 (m, 3H), 6.15 (d, *J* = 9.1 Hz, 0.5H), 6.09 (d, *J* = 9.0 Hz, 0.5H), 4.36 – 4.29 (m, 0.5H), 4.29 – 4.20 (m, 0.5H), 3.94 (dtd, *J* = 8.0, 6.7, 1.2 Hz, 1H), 3.75 (dtd, *J* = 7.8, 5.6, 2.8 Hz, 1H), 2.09 – 1.96 (m, 2H), 1.90 (m, 1H), 1.76 (m, 1H).
^{13}C NMR (101 MHz, $CDCl_3$) δ 150.7, 148.9, 148.8, 148.7, 141.3, 140.9, 140.4, 138.4, 137.7, 137.5, 135.3, 134.9, 131.7, 131.6, 130.0, 128.5, 128.5, 128.0, 127.9, 127.7, 123.3, 123.1, 76.5, 76.4, 68.35, 68.3, 33.3, 33.2, 26.6.
HRMS (EI+) m/z $[M]^+$ calcd. for $C_{17}H_{17}NO$ 251.1305, found 251.1308.

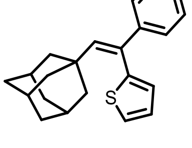

2-((Z)-2-(Adamantan-1-yl)-1-phenylvinyl)thiophene **(67).** Prepared according to GP4 from **1u** (117 mg, 0.36 mmol, 1.2 equiv.) and **3k** (56 mg, 0.3 mmol, 1.0 equiv.). Purified via flash column chromatography on silica gel (*n*-Hexane) to afford the product as a white solid (30 mg, 31% yield).

^{1}**H NMR** (400 MHz, $CDCl_3$) δ 7.33 (dd, *J* = 5.1, 1.2 Hz, 1H), 7.28 – 7.27 (m, 4H), 7.23 – 7.21 (m, 1H), 7.04 (dd, *J* = 5.1, 3.4 Hz, 1H), 6.93 (dd, *J* = 3.4, 1.2 Hz, 1H), 5.99 (s, 1H), 1.94 – 1.91 (m, 3H), 1.72 – 1.65 (m, 12H).
^{13}**C NMR** (101 MHz, $CDCl_3$) δ 145.0, 144.2, 141.6, 131.5, 128.3, 128.1, 127.0, 126.8, 126.4, 125.6, 42.8, 36.8, 36.7, 28.7.
HRMS (EI+) m/z $[M]^+$ calcd. for $C_{22}H_{24}S$ 320.1593, found 320.1597.

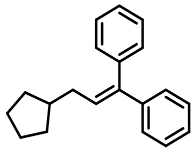

(3-Cyclopentylprop-1-ene-1,1-diyl)dibenzene **(68).** Prepared according to GP4 from **1al** (98 mg, 0.36 mmol, 1.2 equiv.) and **3a** (52 µL, 0.3 mmol, 1.0 equiv.). Purified via flash column chromatography on silica gel (Pentane) to afford the product as a colorless oil (52 mg, 67% yield).

1H NMR (400 MHz, $CDCl_3$) δ 7.44 – 7.24 (m, 3H), 7.25 – 7.12 (m, 7H), 6.13 (t, *J* = 7.4 Hz, 1H), 2.14 (t, *J* = 7.3 Hz, 2H), 2.01 – 1.88 (m, 1H), 1.77 – 1.69 (m, 2H), 1.61 – 1.46 (m, 4H), 1.21 – 1.07 (m, 2H).
^{13}**C NMR** (101 MHz, $CDCl_3$) δ 143.2, 141.6, 140.6, 130.2, 129.8, 128.2, 128.2, 127.4, 126.9, 126.9, 40.8, 35.9, 32.5, 25.2.
HRMS (EI+) m/z $[M]^+$ calcd. for $C_{20}H_{22}$ 262.1716, found 262.1729.

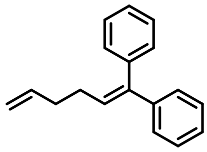

Hexa-1,5-diene-1,1-diyldibenzene **(69).** Prepared according to GP4 from **1am** (88 mg, 0.36 mmol, 1.2 equiv.) and **3a** (52 µL, 0.3 mmol, 1.0 equiv.). Purified via flash column chromatography on silica gel (Pentane) to afford the product as a colorless oil (41 mg, 59% yield).

Characterization data are in accordance with literature.[118]
^{1}**H NMR** (400 MHz, $CDCl_3$) δ 7.41 – 7.25 (m, 4H), 7.25 – 7.17 (m, 6H), 6.11 (t, *J* = 7.4 Hz, 1H), 5.91 – 5.69 (m, 1H), 5.09 – 4.93 (m, 2H), 2.28 – 2.17 (m, 4H).
^{13}**C NMR** (101 MHz, $CDCl_3$) δ 142.9, 142.1, 140.3, 138.3, 130.1, 129.3, 128.3, 128.2, 127.4, 127.0, 127.0, 115.1, 34.2, 29.3.
HRMS (EI+) m/z $[M]^+$ calcd. for $C_{18}H_{18}$ 234.1403, found 234.1413.

11. X-Ray crystal data of products

Single crystal x-ray diffraction data were recorded on Agilent Technologies Supernova, Xcalibur Gemini Ultra, Agilent GV 50 or Rigaku GV 50 diffractometers with Cu-K_a radiation (l = 1.54184 Å). Suitable crystals were mounted on a Lindemann tube oil and kept at a steady temperature of T = 123 K during data collection. Empirical multi-scan[119,120] and analytical absorption corrections[121,122] were applied to the data. Structures were solved using SHELXT[123] using dual methods and Olex2 as the graphical interface,[124] and least-squares refinements on F2 were carried out using SHELXL.[120-122] Hydrogen atoms were localized in idealized positions and refined isotropically with a riding model.

CCDC 2414659 (**4**) 2414660 (**44**) 2414661 (**67**) contain the supplementary crystallographic data for this paper. These data are provided free of charge by the Cambridge Crystallographic Data Centre.

Table S14. Crystallographic data and structure refinements for compounds **4**, **44** and **67**.

**Entry**	**4**	**44**	**67**
Empirical formula	$C_{19}H_{19}F_3NO$	$C_{24}H_{26}$	$C_{22}H_{24}S$
ρcalc **/ (g/cm^{-3})**	1.330	1.182	1.254
μ **/ mm^{-1}**	0.884	0.492	1.641
**Formula weight**	334.35	314.45	320.47
**Crystal colour**	clear colourless	clear colourless	colourless
**Crystal shape**	needle-shaped	prism-shaped	prism-shaped
**Crystal size/mm^3**	0.10×0.03×0.02	0.12×0.11×0.08	0.06×0.05×0.03
**Temperature / K**	123.00(10)	123.00(10)	123.00(10)
**Crystal system**	orthorhombic	triclinic	monoclinic
**Space group**	$Pna2_1$	P-1	$P2_1/c$
a **/Å**	17.0316(13)	9.3326(2)	28.690(3)
b **/ Å**	19.6011(12)	13.3334(2)	6.4774(4)
c **/ Å**	5.0025(3)	15.9179(3)	18.5591(19)
α **/ °**	90	68.086(2)	90
β **/ °**	90	74.542(2)	100.253(9)
γ **/ °**	90	87.5050(10)	90
**Volume / Å^3**	1670.03(19)	1767.69(6)	3393.9(5)
Z	4	4	8

Z'	1	2	2
**Wavelength / Å**	1.54184	1.54184	1.54184
**Radiation**	Cu K_α	Cu K_α	Cu K_α
θ_{min}/°	3.438	3.108	3.131
θ_{max}/°	73.646	73.942	73.425
**Reflections collected**	9617	65422	22013
**Independent reflections**	2795	6967	6430
**Reflections I≥2 σ (I)**	2134	6302	3883
R_{int}	0.0298	0.0221	0.0586
**Parameters**	218	433	657
**Restraints**	1	0	208
**Largest peak**	0.340	0.231	0.238
**Deepest hole**	-0.219	-0.204	-0.233
**GooF**	1.028	1.052	1.002
wR_2 **(all data)**	0.1299	0.0911	0.1101
wR_2	0.1176	0.0888	0.0912
R_1 **(all data)**	0.0680	0.0391	0.0998
R_1	0.0456	0.0354	0.0442

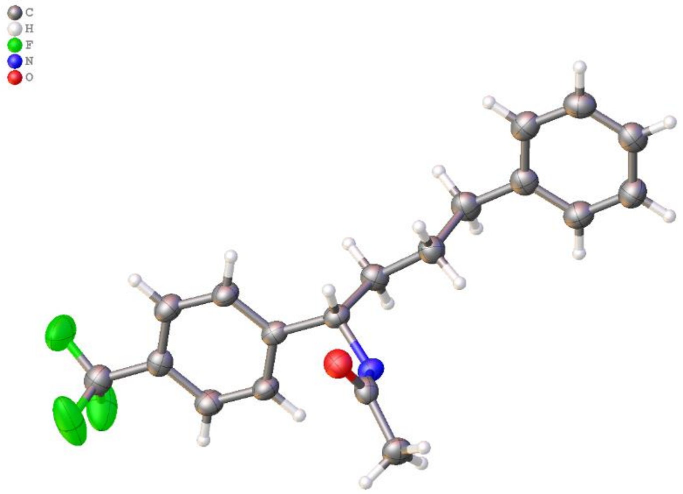

Figure S27: Crystal structure of **4**. Thermal ellipsoids are set at the 50% probability level. C atoms are shown in grey, H atoms in white, F atoms in green, N atoms in blue and O atoms in red.

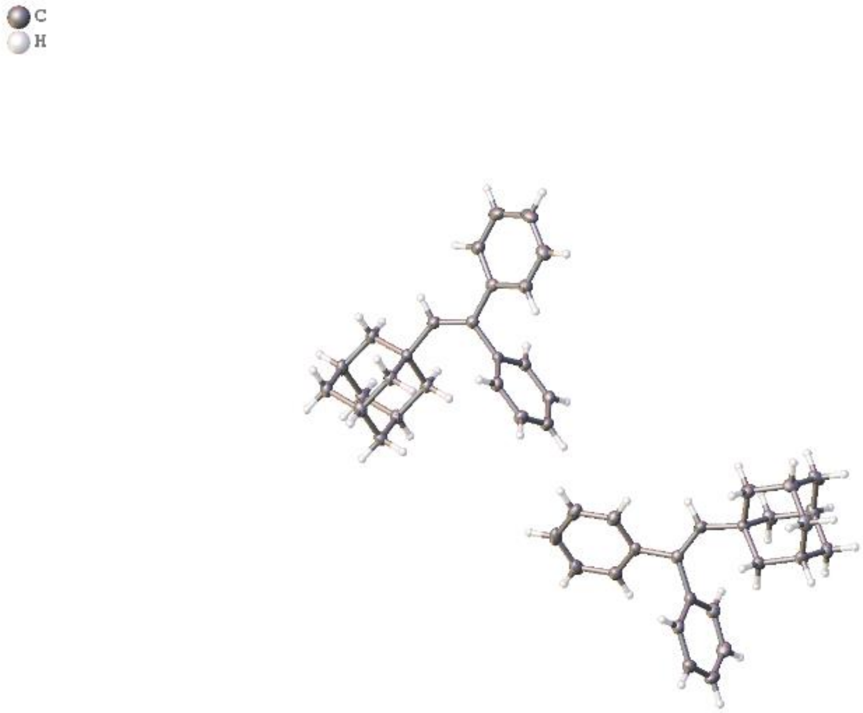

Figure S28: Crystal structure of **44**. Thermal ellipsoids are set at the 50% probability level. C atoms are shown in grey, H atoms in white.

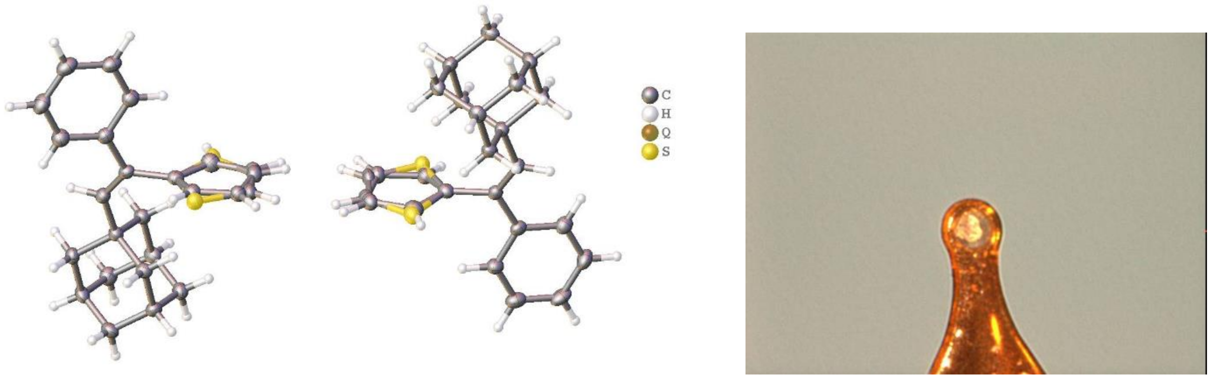

Figure S29: Left: Crystal structure of **67**. Thermal ellipsoids are set at the 50% probability level. C atoms are shown in grey, H atoms in white, S atoms in yellow. Right: picture of crystal of **67** in the diffractometer.

12. Limitation of the scope

Ritter-type carboamidation

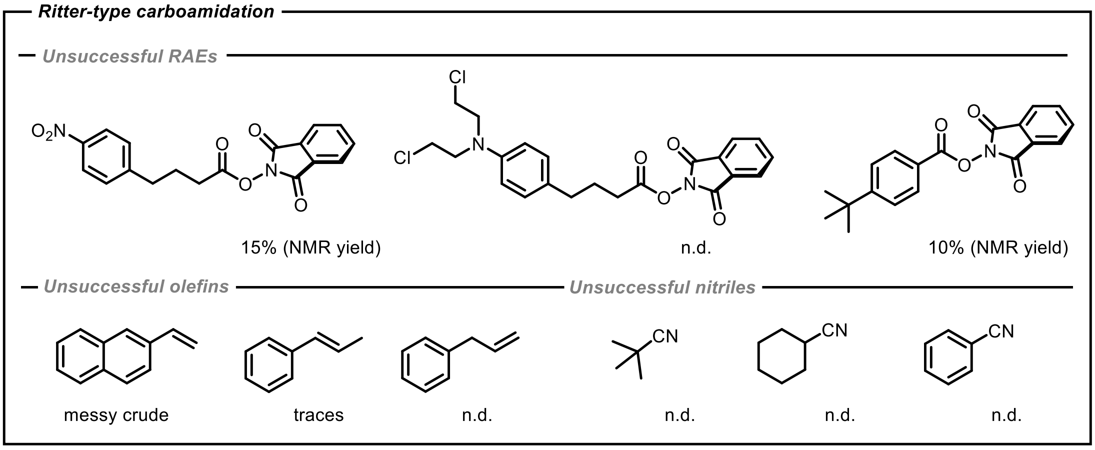

Figure S30: Unsuccessful redox-active esters (top), olefins (bottom left) and nitriles (bottom right) in the Ritter-type reaction when subjected to the optimized reaction conditions. ^{1}H NMR yields determined using 1,1,2,2-tetrachloroethane as an internal standard.

Heck-type reaction

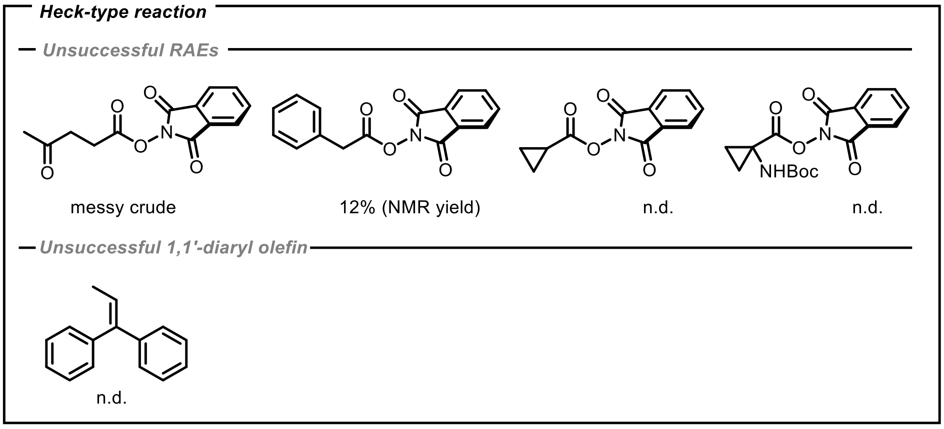

Figure S31: Unsuccessful redox-active esters (top) and an unsuccessful 1,1'-diaryl olefin (bottom) in the Heck-type reaction when subjected to the optimized reaction conditions. ^{1}H NMR yields determined using 1,1,2,2-tetrachloroethane as an internal standard.

13. Computational studies

All the calculations have been performed using GAUSSIAN 16 code[125] and the widely used B3LYP functional, which has been proved suitable for different kind of systems,[126] has been selected. Grimme D3 dispersion corrections[127] have been added to take into account the effects of dispersion interactions. The standard 6-31+G* basis sets of Pople and co-workers were employed for the all the atoms. We performed the geometry optimizations to intercept all the reactants and stationary points along the potential energy surfaces, whose character of minima or transition states has been determined by frequency analysis. It was carefully checked that the vibrational mode associated with the imaginary frequency corresponds to the correct movement of involved atoms. The impact of solvation has been carefully evaluated using polarizable continuum model (PCM) in acetonitrile. Scan calculations have been performed using the same computational protocol.

13.1 Optimized structures of the intercepted stationary points

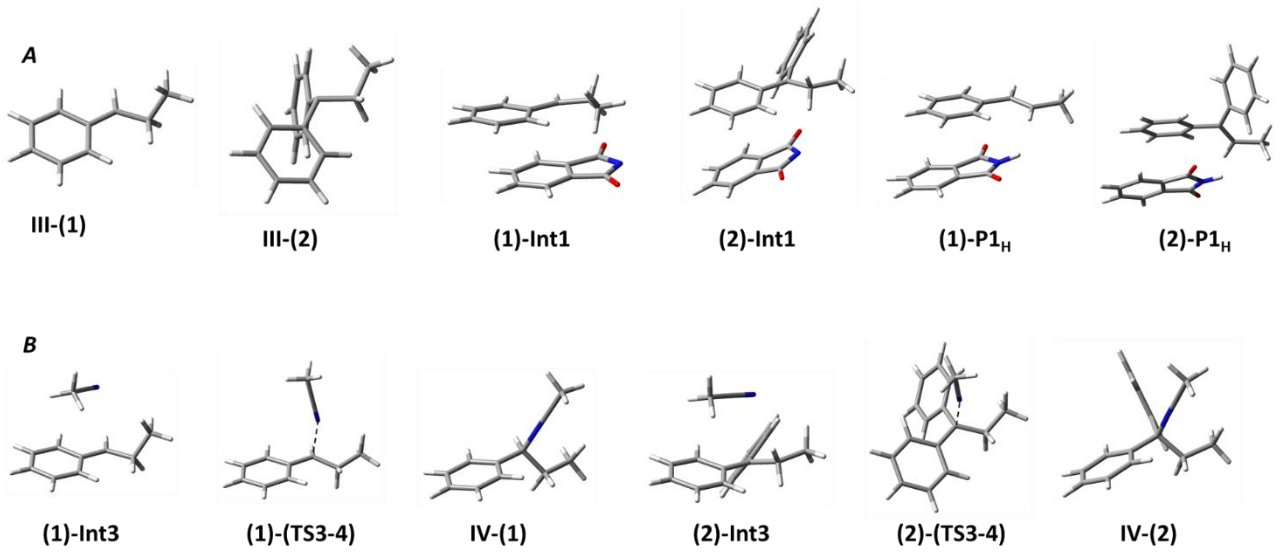

Figure S32. Optimized structures of all the intercepted stationary points intercepted along A) the Heck reaction profile and B) the Ritter reaction leading to the nitrilium ion **IV.**

13.2 Cartesian coordinates of the intercepted stationary points

III-(1)

Charge = +1, Multiplicity = 1

C	1.28205900	0.58492500	-0.33904100
C	-0.05952000	0.24830600	-0.16598700
C	-0.51883000	-1.10816500	-0.11038500
C	-1.01203000	1.31314400	-0.03866000
C	-1.86273700	-1.37280100	0.06194300
H	0.18656600	-1.92569500	-0.20137100
C	-2.35449200	1.03234900	0.13206800
H	-0.66387800	2.34090700	-0.08001000
C	-2.77624000	-0.30723700	0.18189100
H	-2.21839900	-2.39665700	0.10592000
H	-3.07800300	1.83480800	0.22775900
H	-3.83146900	-0.52720200	0.31653400
C	2.44580300	-0.30151500	-0.48764900
H	2.18150700	-1.35924200	-0.52468600
H	2.91798500	-0.03395800	-1.44704300
C	3.48360500	-0.03179700	0.63801700
H	4.36408500	-0.65382900	0.45418200
H	3.79407100	1.01767800	0.65141200
H	3.06813300	-0.29091800	1.61604100
H	1.51370300	1.65085100	-0.35192600

G = - -349.261370 Hartree

III-(2)

Charge = +1, Multiplicity = 1

C	-2.40738700	0.45469500	0.80430000
C	-1.21649700	0.04630600	0.14338900
C	-1.28289900	-1.06683000	-0.74176200
C	-2.48088800	-1.74027500	-0.94382700
C	-3.63280000	-1.33951200	-0.25670400
C	-3.59081000	-0.24431200	0.61967700
H	-2.38761200	1.28291100	1.50307300
H	-0.40725800	-1.34892000	-1.31526700
H	-2.52176000	-2.57000400	-1.64243500
H	-4.56647400	-1.87328100	-0.40848700
H	-4.48462600	0.05747800	1.15651700
C	0.01736600	0.77682800	0.29762500
C	1.28719500	0.11434100	0.18868700
C	2.44120200	0.81897500	-0.25659600
C	1.42839100	-1.25300600	0.56174600
C	3.65925500	0.16808100	-0.37465500
H	2.36381300	1.85694100	-0.55967000
C	2.66227800	-1.88389500	0.47674100
H	0.58039600	-1.78643700	0.97591800
C	3.77449800	-1.18101900	-0.00323200
H	4.52489800	0.70489400	-0.74946700
H	2.76325400	-2.91780200	0.79104800
H	4.73695300	-1.67898400	-0.07712100
C	-0.03814000	2.25841300	0.51075100
H	-0.77868900	2.47936600	1.28727800
H	0.91837900	2.63675000	0.87421100
C	-0.46189900	3.00163100	-0.78411900
H	-0.49055000	4.07593200	-0.57975800
H	0.24983300	2.82008900	-1.59533200
H	-1.45374200	2.67854700	-1.11263400

G = -580.270220 Hartree

(1)-Int1

Charge = 0, Multiplicity = 1

C	-2.06159300	1.67224800	-0.18401400
C	-3.06912200	0.70437500	-0.16138700
C	-2.78767600	-0.69520800	-0.28357700
C	-4.42365200	1.13693000	0.00924400
C	-3.82703600	-1.60512600	-0.23404300
H	-1.76550800	-1.04772900	-0.39884800
C	-5.45136200	0.21238000	0.04835000
H	-4.63273800	2.19847000	0.10456600
C	-5.15118500	-1.15511300	-0.07263500
H	-3.62453500	-2.66787400	-0.31841500
H	-6.47928300	0.53565800	0.17416100
H	-5.95840600	-1.88150700	-0.03855700
C	-0.61866300	1.43866700	-0.33555500
H	-0.30309200	0.92498500	0.59675600
H	-0.43936800	0.67878500	-1.10804800
C	0.22855400	2.69268500	-0.56774300
H	1.28780900	2.42075700	-0.60270600
H	-0.03244400	3.17436100	-1.51602400
H	0.08962300	3.41870000	0.24058800
C	2.33512000	-0.79691100	-0.44044100
C	2.90382100	-0.14316200	0.65213000
C	4.11743500	0.52196000	0.54931000
C	4.75723100	0.51411500	-0.70315900
C	4.18465000	-0.14265000	-1.80189700
C	2.95378600	-0.81219600	-1.68271700
C	1.02484700	-1.36254000	0.05111300
C	1.93375100	-0.32707500	1.79718000
H	4.55637800	1.03372800	1.40148600
H	5.70867600	1.02548500	-0.82387100
H	4.70036600	-0.13127400	-2.75855200
H	2.50454100	-1.31934300	-2.53230600
O	0.21823500	-1.98074500	-0.66697100
O	2.10470300	0.12453900	2.93929400
N	0.84165300	-1.04854500	1.37169500
H	-2.38053500	2.70599700	-0.04162300

G = - -861.849981 Hartree

(2)-Int1

Charge = 0, Multiplicity = 1

C	-3.40074600	-0.30406700	-1.09630900
C	-2.93099300	0.05947000	0.17623900
C	-3.58981600	1.09504300	0.86228100
C	-4.69881500	1.73307300	0.30227500
C	-5.16130100	1.35653200	-0.96465400
C	-4.50625100	0.33756200	-1.66347600
H	-2.88875200	-1.08885000	-1.64670500
H	-3.22760600	1.39840600	1.84153600
H	-5.20009200	2.52568700	0.85223800
H	-6.02056100	1.85663700	-1.40402900
H	-4.85128800	0.04401500	-2.65175200
C	-1.74377800	-0.61523000	0.78200700
C	-0.60866500	0.26177700	1.19135100
C	0.21038200	-0.05414900	2.29236100
C	-0.31193800	1.43103000	0.46475300
C	1.29950500	0.74994400	2.63410400
H	-0.01344400	-0.93155100	2.89231800
C	0.77693200	2.23587200	0.80596000
H	-0.92737100	1.69929500	-0.38881800
C	1.59077900	1.89846400	1.89120900
H	1.91950800	0.47946800	3.48514900
H	0.99684100	3.11991600	0.21317900
H	2.44330700	2.51952200	2.15216800
C	-1.67506300	-1.95704700	0.93553800
H	-0.74406900	-2.37462000	1.31703400
C	-2.74597700	-2.97069400	0.64688500
H	-2.47775200	-3.59544200	-0.21723200
H	-2.85439600	-3.65301700	1.50061000
H	-3.71819600	-2.51428800	0.44235600
C	2.49243500	0.11636700	-1.33529400
C	2.87605100	-0.75105900	-0.30666400
C	4.03522300	-0.54060800	0.42577900
C	4.81379400	0.57847300	0.09360900
C	4.43059800	1.44632600	-0.93944700
C	3.25524500	1.22478200	-1.67309400
C	1.20204000	-0.37425100	-1.89898300
C	1.84448000	-1.82118600	-0.19278900
H	4.32334000	-1.21161400	1.22901300
H	5.72645800	0.77892300	0.64727200
H	5.05253000	2.30592100	-1.17188000
H	2.94945200	1.89791500	-2.46801600
O	0.51290600	0.10492900	-2.78518700
O	1.78556700	-2.76011000	0.58507900
N	0.90997500	-1.54235000	-1.19229800
H	0.02976200	-2.03695600	-1.27933500

G = -1092.914502 Hartree

(1)-P_H

Charge = 0, Multiplicity = 1

C	-1.07350000	-1.50114700	1.18673700
C	-1.56897000	-1.33350800	-0.12264700
C	-0.80849000	-1.85856300	-1.18531700
C	0.40776800	-2.50657600	-0.95584700
C	0.89062200	-2.65186400	0.34709600
C	0.14037600	-2.14762300	1.41658700
H	-1.63644700	-1.11919000	2.03320300
H	-1.16802100	-1.73796200	-2.20478600
H	0.98188600	-2.88682100	-1.79684500
H	1.84186800	-3.14439400	0.52917400
H	0.50675800	-2.25224600	2.43466100
C	-2.82444700	-0.62551200	-0.42831000
C	-3.56301500	0.11962600	0.41322400
H	-1.51230300	2.03662700	-0.27684900
H	-3.24379800	0.24445400	1.44840600
C	-4.83156700	0.82772700	0.03404400
H	-5.67402300	0.47917000	0.64760500
H	-4.74577500	1.90917500	0.20933500
H	-5.08601800	0.66938000	-1.02002100
C	1.52302400	0.91564600	-0.75054300
C	1.46567400	1.04542500	0.64137300
C	2.53285000	0.68050900	1.44932100
C	3.67768200	0.17406000	0.81574100
C	3.73519900	0.04378900	-0.57968900
C	2.64988200	0.41592200	-1.38726400
C	0.22226500	1.37862600	-1.31365400
C	0.12619000	1.59253300	1.00154000
H	2.47990000	0.77677200	2.52934300
H	4.53262600	-0.12577100	1.41481300
H	4.63379500	-0.35515500	-1.04129700
H	2.68632300	0.31063700	-2.46712400
O	-0.15167600	1.40713200	-2.47521200
O	-0.34207200	1.82874800	2.10386300
N	-0.52849500	1.80023900	-0.21406000
H	-3.15657200	-0.70780300	-1.46475400

G = -861.917675Hartree

(2)-P_H

Charge = +1, Multiplicity = 1

C	-3.40074600	-0.30406700	-1.09630900
C	-2.93099300	0.05947000	0.17623900
C	-3.58981600	1.09504300	0.86228100
C	-4.69881500	1.73307300	0.30227500
C	-5.16130100	1.35653200	-0.96465400
C	-4.50625100	0.33756200	-1.66347600
H	-2.88875200	-1.08885000	-1.64670500
H	-3.22760600	1.39840600	1.84153600
H	-5.20009200	2.52568700	0.85223800
H	-6.02056100	1.85663700	-1.40402900
H	-4.85128800	0.04401500	-2.65175200
C	-1.74377800	-0.61523000	0.78200700
C	-0.60866500	0.26177700	1.19135100
C	0.21038200	-0.05414900	2.29236100
C	-0.31193800	1.43103000	0.46475300
C	1.29950500	0.74994400	2.63410400
H	-0.01344400	-0.93155100	2.89231800
C	0.77693200	2.23587200	0.80596000
H	-0.92737100	1.69929500	-0.38881800
C	1.59077900	1.89846400	1.89120900
H	1.91950800	0.47946800	3.48514900
H	0.99684100	3.11991600	0.21317900
H	2.44330700	2.51952200	2.15216800
C	-1.67506300	-1.95704700	0.93553800
H	-0.74406900	-2.37462000	1.31703400
C	-2.74597700	-2.97069400	0.64688500
H	-2.47775200	-3.59544200	-0.21723200
H	-2.85439600	-3.65301700	1.50061000
H	-3.71819600	-2.51428800	0.44235600
C	2.49243500	0.11636700	-1.33529400
C	2.87605100	-0.75105900	-0.30666400
C	4.03522300	-0.54060800	0.42577900
C	4.81379400	0.57847300	0.09360900
C	4.43059800	1.44632600	-0.93944700
C	3.25524500	1.22478200	-1.67309400
C	1.20204000	-0.37425100	-1.89898300
C	1.84448000	-1.82118600	-0.19278900
H	4.32334000	-1.21161400	1.22901300
H	5.72645800	0.77892300	0.64727200
H	5.05253000	2.30592100	-1.17188000
H	2.94945200	1.89791500	-2.46801600
O	0.51290600	0.10492900	-2.78518700
O	1.78556700	-2.76011000	0.58507900
N	0.90997500	-1.54235000	-1.19229800
H	0.02976200	-2.03695600	-1.27933500

G = -1092.914502 Hartree

(1)-Int3

Charge = +1, Multiplicity = 1

C	0.93130300	1.12076000	0.70154900
C	-0.39510700	0.82433800	0.38427700
C	-1.01399300	1.24632000	-0.83604100
C	-1.15419600	0.04640100	1.31832900
C	-2.32559200	0.90114300	-1.09816400
H	-0.45417500	1.83006300	-1.55720800
C	-2.46662900	-0.28893500	1.04402500
H	-0.68178000	-0.28094400	2.23932300
C	-3.04828900	0.13708600	-0.16235000
H	-2.80002200	1.21414800	-2.02210400
H	-3.04341500	-0.87850700	1.74852200
H	-4.07857200	-0.13030200	-0.37930000
C	1.90992400	1.90085300	-0.06905600
H	1.49749500	2.32899400	-0.98382700
H	2.21328200	2.73348500	0.58757300
C	3.18086000	1.06157800	-0.37493000
H	3.91505400	1.70894800	-0.86234100
H	3.61988000	0.65619300	0.54120300
H	2.94018700	0.23109200	-1.04358300
H	1.30405900	0.71643400	1.64244900
N	1.82100800	-1.96662400	0.86919600
C	1.15867900	-2.29117000	-0.02875500
C	0.31218800	-2.66983700	-1.15595700
H	0.71909200	-3.55642000	-1.65132600
H	-0.70017100	-2.88986600	-0.80353700
H	0.26713800	-1.84816100	-1.87778100

G = -482.001641 Hartree

(2)-Int3

Charge = +1, Multiplicity = 1

C	0.22737500	0.21887000	0.82234200
C	-0.98753500	-0.51517000	0.56600800
C	-2.10188200	-0.45840100	1.44931200
C	-1.11961900	-1.26359200	-0.63790100
C	-3.26876800	-1.14645000	1.15622100
H	-2.03436800	0.08731500	2.38305000
C	-2.30232400	-1.93035200	-0.93375000
H	-0.31335500	-1.26150000	-1.36219900
C	-3.37406100	-1.88173000	-0.03593500
H	-4.10125600	-1.11779600	1.85209900
H	-2.39431900	-2.47638100	-1.86720700
H	-4.29631700	-2.40764700	-0.26516600
C	0.16421200	1.38749700	1.76341300
H	-0.74017400	1.96603600	1.54881700
H	-0.03357300	0.90109500	2.73618400
C	1.35555800	2.32921600	1.95119200
H	1.15173400	2.96318100	2.81965500
H	2.28904000	1.79058700	2.13868000
H	1.48969100	2.98528100	1.08791100
N	-0.19534200	2.58607100	-1.29433900
C	-1.29090600	2.28298100	-1.53502900
C	-2.66711300	1.88712600	-1.81733100
H	-3.18978700	2.69300800	-2.34135900
H	-2.67650700	0.98888700	-2.44164800
H	-3.19049000	1.67382900	-0.88016200
C	1.46490800	-0.25749600	0.24784400
C	2.42642400	0.61668700	-0.32062400
C	1.73596500	-1.65382900	0.26263100
C	3.59537600	0.10738400	-0.86975300
H	2.21028000	1.67232500	-0.40310800
C	2.93538700	-2.14516400	-0.23928000
H	1.02649600	-2.33127700	0.72521000
C	3.86183700	-1.26857800	-0.81559300
H	4.30477300	0.78077300	-1.34073500
H	3.14566100	-3.20887700	-0.18768600

H	4.79086400	-1.65532900	-1.22455700

G = -713.003517 Hartree

(1)-TS(3-4)

Charge = +1, Multiplicity = 1

C	-0.52205700	-1.04077900	0.53510300
C	0.76941900	-0.49461800	0.23724700
C	1.36292700	-0.60399200	-1.04419300
C	1.47766000	0.15483500	1.27842400
C	2.63380600	-0.09110800	-1.26547600
H	0.82803800	-1.08233300	-1.85725300
C	2.75056000	0.66334700	1.05055200
H	1.01888300	0.24856700	2.25883500
C	3.32682300	0.54083600	-0.22079800
H	3.09016000	-0.17547600	-2.24669700
H	3.29372100	1.15404200	1.85195200
H	4.32013800	0.94177400	-0.40168800
C	-1.21699200	-2.07977200	-0.26510000
H	-1.11164600	-1.89736100	-1.33837300
H	-0.61610400	-2.98444500	-0.05570100
C	-2.67440000	-2.33926500	0.12284300
H	-3.05131700	-3.21402300	-0.41537700
H	-2.77081100	-2.53123200	1.19732800
H	-3.30100400	-1.47959900	-0.13248900
N	-1.79953000	0.63450000	0.13238400
C	-2.11417600	1.72824000	-0.08414700
C	-2.52473400	3.09808500	-0.35791700
H	-3.04554000	3.14152400	-1.31914700
H	-3.19539700	3.44694500	0.43302500
H	-1.64290500	3.74499200	-0.39539400
H	-0.83251400	-0.96972600	1.57505900

G = -481.996493 Hartree

(2)-TS(3-4)

Charge = +1, Multiplicity = 1

C	0.04226800	0.16990600	0.70709700
C	-1.22057300	-0.49559600	0.32165500
C	-2.26555000	-0.69712600	1.24517900
C	-1.39187500	-0.93709100	-1.00850500
C	-3.43613100	-1.34479200	0.85268100
H	-2.16628000	-0.37844600	2.27598000
C	-2.56985400	-1.56285800	-1.40171000
H	-0.60456700	-0.76482500	-1.73530500
C	-3.59352300	-1.77408100	-0.46917700
H	-4.22582200	-1.51217900	1.57893200
H	-2.69193700	-1.88658700	-2.43099000
H	-4.51100600	-2.27011200	-0.77316300
C	0.15544500	0.74254000	2.10421200
H	-0.76431100	1.28000900	2.34825400
H	0.17882700	-0.15151800	2.74748700
C	1.36811100	1.61768700	2.42459600
H	1.30302300	1.93540400	3.46981800
H	2.31193400	1.08232700	2.29067100
H	1.38505200	2.51519400	1.79853900
N	-0.20543300	1.89146100	-0.25997100
C	-0.67164600	2.73505400	-0.89929900
C	-1.24329200	3.80022000	-1.70673100
H	-1.34013400	4.70738400	-1.10276800
H	-0.59152100	4.00000000	-2.56265400
H	-2.23125500	3.49468500	-2.06530300
C	1.29383000	-0.35296200	0.08026500
C	2.35157200	0.46732900	-0.34770600
C	1.45033300	-1.75167400	0.02521100
C	3.53158300	-0.09654200	-0.82857000
H	2.23897500	1.54474500	-0.33299300
C	2.64649300	-2.31104900	-0.43021100
H	0.64853300	-2.40247300	0.35784800
C	3.68686000	-1.48739700	-0.86496300
H	4.33046600	0.55181800	-1.17669300
H	2.75385700	-3.39157200	-0.45551300
H	4.60988300	-1.92348300	-1.23648800

G = -712.989704 Hartree

IV-(1)

Charge = +1, Multiplicity = 1

C	-0.63291600	-0.70881000	0.40846700
C	0.79375300	-0.23199400	0.18701800
C	1.15204900	0.44117300	-0.98907200
C	1.76709400	-0.51052600	1.15301700
C	2.47647500	0.83211200	-1.19513900
H	0.40115200	0.66623300	-1.74212700
C	3.09515900	-0.12627700	0.94098400
H	1.49196500	-1.02838500	2.06845800
C	3.45094200	0.54631100	-0.23155800
H	2.74734400	1.35697600	-2.10696800
H	3.84638900	-0.34831600	1.69373300
H	4.48171200	0.84887900	-0.39451700
C	-1.10129800	-1.78657700	-0.59249000
H	-1.01503100	-1.38797300	-1.60933200
H	-0.37004900	-2.59746700	-0.50268100
C	-2.51502800	-2.30909600	-0.32580200
H	-2.75069700	-3.11749000	-1.02546200
H	-2.60646100	-2.70468900	0.69258200
H	-3.27047100	-1.52621600	-0.45838300
H	-0.74572400	-1.08697600	1.42958200
N	-1.53583000	0.43071100	0.34440600
C	-2.24433100	1.33203000	0.27734600
C	-3.13826100	2.46630800	0.20052800
H	-4.17267000	2.10829300	0.21418900
H	-2.96089200	3.12161800	1.05908600
H	-2.94758600	3.01260800	-0.72880100

G = -482.014410 Hartree

VI-(2)

Charge = +1, Multiplicity = 1

C	-0.05759600	0.43134400	0.48034200
C	-1.29406100	-0.43209300	0.14580500
C	-2.57160900	0.14860400	0.14257500
C	-1.16746100	-1.80613700	-0.08836800
C	-3.70348100	-0.63132700	-0.09890100
H	-2.69344500	1.21294900	0.32543400
C	-2.30417300	-2.58828900	-0.31996300
H	-0.18974200	-2.27451500	-0.09213000
C	-3.57313700	-2.00517400	-0.32924700
H	-4.68470600	-0.16503700	-0.10491200
H	-2.19041700	-3.65395200	-0.49837000
H	-4.45380000	-2.61358600	-0.51553500
C	-0.11220900	0.87487300	1.97439000
H	-1.10041400	1.31272200	2.14542900
H	-0.08445600	-0.04467800	2.56655900
C	0.97666600	1.85488100	2.41915000
H	0.85183900	2.07173700	3.48499800
H	1.98240900	1.45236300	2.26984900
H	0.90736000	2.80553700	1.87895800
N	-0.17850600	1.66956200	-0.30011100
C	-0.28655600	2.66254300	-0.86751200
C	-0.42627200	3.90513700	-1.59271200
H	0.44192700	4.54009300	-1.38987100
H	-0.48678900	3.68991300	-2.66425400
H	-1.34013300	4.41010800	-1.26370900
C	1.26109300	-0.23601000	0.05686000
C	1.66488800	-0.21427400	-1.28572800
C	2.02534200	-0.95647900	0.98329000
C	2.82178800	-0.88237700	-1.69151000
H	1.07453100	0.31360100	-2.02919700
C	3.18069900	-1.63025000	0.57589400
H	1.73035000	-1.01079400	2.02498800
C	3.58572000	-1.59259900	-0.76043800
H	3.12007200	-0.85091800	-2.73564000
H	3.76151800	-2.18268700	1.30913500
H	4.48558100	-2.11403800	-1.07451400

G = -713.003668 Hartree

13.3 Scan analysis

The relaxed scan calculation, shown in **Figure S33A**, involves 50 steps in which the initial N–H distance between the nitrogen atom of the base and the hydrogen atom of the CH_2 group of **III**-(1) is decreased by 0.03 Å to the final bond distance of 1.02 Å in (**1)-P$_H$**. The scan calculation underlines the absence of any maximum along the curve, thus suggesting that the proton transfer step is barrierless. On the other hand, the scan plot of the proton transfer from **III**-(2) to the phtalimide anion shows a low uphill behavior that reaches its maximum at the scan step 26. In the corresponding optimized structure, the C–H bond distance is elongate to 1.24 Å while the N–H distance is shortened to 1.58 Å. However, the associated energy barrier is just ~2 kcal/mol, thus suggesting that also for **III**-(2) this step is a feasible process, but the higher steric hindrance of **III**-(2) slightly hinders the proton transfer in comparison to **III**-(1).

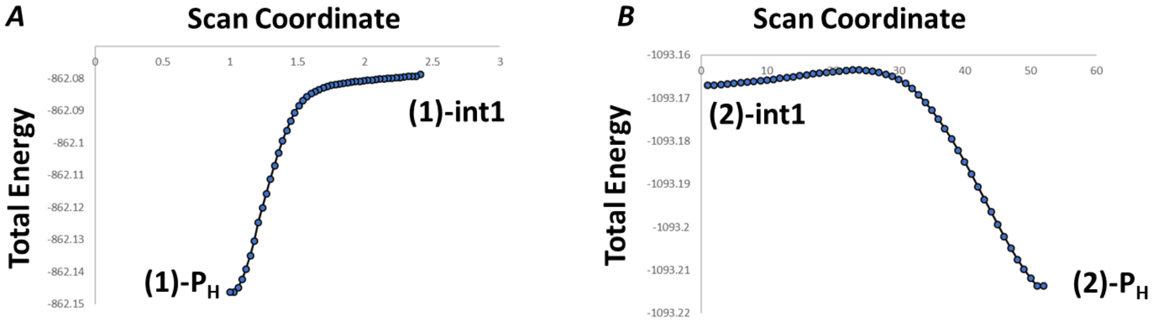

Figure S33: Calculated scan plots for the proton transfer in presence of A) **III**-(1) and B) **III**-(2).

14. References

[89] S. Engle, *Org. Synth.* **2019**, *96*, 455–473.

[90] H. E. Askey, J. D. Grayson, J. D. Tibbetts, J. C. Turner-Dore, J. M. Holmes, G. Kociok-Kohn, G. L. Wrigley, A. J. Cresswell, *J. Am. Chem. Soc.* **2021**, *143*, 15936–15945.

[91] J. Brauer, E. Quraishi, L. M. Kammer, T. Opatz, *Chem. Eur. J.* **2021**, *27*, 18168–18174.

[92] M. M. López, N. Jamey, A. Pinet, B. Figadère, L. Ferrié, *Org. Lett.* **2021**, *23*, 1626–1631.

[93] J. Xu, B. Liu, *Chem. Eur. J.* **2024**, *30*, e202400612.

[94] D. Yin, L. Lu, Y. Jiang, Y. Dou, M. C. Fu, Y. Zhu, S. Fan, *J. Org. Chem.* **2024**, *89*, 13085–13092.

[95] T. M. Masson, S. D. A. Zondag, J. H. A. Schuurmans, T. Noël, *React. Chem. Eng.* **2024**, *9*, 2218–2225.

[96] S. Luo, H. D. M. Pham, C. C. Li, Z. Qiu, R. Cheng, R. Z. Khaliullin, C. J. Li, *Org. Lett.* **2024**, *26*, 3004–3009.

[97] Z. Zuo, Z. Huang, *Org. Chem. Front.* **2016**, *3*, 434–438.

[98] E. J. Ko, G. P. Savage, C. M. Williams, J. Tsanaktsidis, *Org. Lett.* **2011**, *13*, 1944–1947.

[99] V. V Pavlishchuk, A. W. Addison, *Inorganica Chim. Acta* **2000**, *298*, 97–102.

[100] J. R. Aranzaes, M.-C. Daniel, D. Astruc, *Can. J. Chem.* **2006**, *84*, 288–299.

[101] Z. Chen, F. Xue, T. Liu, B. Wang, Y. Zhang, W. Jin, Y. Xia, C. Liu, *Green Chem.* **2022**, *24*, 3250–3256.

[102] O. Z. Esezobor, W. Zeng, L. Niederegger, M. Grübel, C. R. Hess, *J. Am. Chem. Soc.* **2022**, *144*, 2994–3004.

[103] K. Rickmeyer, M. Huber, C. R. Hess, *Chem. Commun.* **2024**, *60*, 819–822.

[104] T. Wan, L. Capaldo, J. Djossou, A. Staffa, F. J. de Zwart, B. de Bruin, T. Noël, *Nat. Commun.* **2024**, *15*, 4028.

[105] C. A. Hone, C. O. Kappe, *Chemistry–Methods* **2021**, *1*, 454–467.

[106] B. Zhao, R. Shang, G.-Z. Wang, S. Wang, H. Chen, Y. Fu, *ACS Catal.* **2020**, *10*, 1334–1343.

[107] T. W. Liwosz, S. R. Chemler, *Org. Lett.* **2013**, *15*, 3034–3037.

[108] T. Satoh, A. Kondo, J. Musashi, *Tetrahedron* **2004**, *60*, 5453–5460.

[109] Q. Q. Zhou, S. J. S. Düsel, L. Q. Lu, B. König, W. J. Xiao, *Chem. Commun.* **2019**, *55*, 107–110.

[110] W. Wei, X. J. Dai, H. Wang, C. Li, X. Yang, C. J. Li, *Chem. Sci.* **2017**, *8*, 8193–8197.

[111] S. Ni, Y. Zhang, C. Xie, H. Mei, J. Han, Y. Pan, *Org. Lett.* **2015**, *17*, 5524–5527.

[112] Z. Zong, W. Wang, X. Bai, H. Xi, Z. Li, *Asian J. Org. Chem.* **2015**, *4*, 622–625.

[113] M. Koy, F. Sandfort, A. Tlahuext-Aca, L. Quach, C. G. Daniliuc, F. Glorius, *Chem. Eur. J.* **2018**, *24*, 4552–4555.

[114] S. J. Burlingham, D. Guijarro, I. Bosque, R. Chinchilla, J. C. Gonzalez-Gomez, *Org. Biomol. Chem.* **2022**, *20*, 7923–7928.

[115] C. Wang, P. Liang, M. Li, B. Wang, Y. Wang, X. Li, W. Wei, X. Gou, Y. Ding, Z. Zhang, Y. Li, X. Liu, Y. Liang, *Angew. Chem., Int. Ed.* **2023**, *62*, e202304447.

[116] J. Xie, J. Li, V. Weingand, M. Rudolph, A. S. K. Hashmi, *Chem. Eur. J.* **2016**, *22*, 12646–12650.

[117] S. M. Duque-Benítez, L. A. Ríos-Vásquez, R. Ocampo-Cardona, D. L. Cedeño, M. A. Jones, I. D. Vélez, S. M. Robledo, *Molecules* **2016**, *21*, 1–16.

[118] A. Jiménez-Aquino, E. Ferrer Flegeau, U. Schneider, S. Kobayashi, *Chem. Commun.* **2011**, *47*, 9456–9458.

[119] SCALE3ABS, CrysAlisPro, Agilent Technologies Inc. Oxford and GB, **2015**.

[110] G. M. Sheldrick, SADABS, Bruker AXS, Madison and USA, **2007**.

[121] R. C. Clark, J. S. Reid, *Acta Cryst. A* **1995**, *51*, 887-897.

[122] CrysAlisPro, version 171.39.37b, Agilent Technologies Inc., Oxford and GB, **2017**.

[123] G. M. Sheldrick, *Acta Cryst. C* **2015**, *71*, 3-8.

[124] O. V. Dolomanov, L. J. Bourhis, R. J. Gildea, J. A. K. Howard, H. Puschmann, *J. Appl. Crystallogr.* **2009**, *42*, 339–341.

[125] M. J. Frisch, G. W. Trucks, H. B. Schlegel, G. E. Scuseria, M. A. Robb, J. R. Cheeseman, G. Scalmani., V. Barone, G. A. Petersson, H. Nakatsuji, X. Li; M. Caricato, A. V. Marenich, J. Bloino, B. G. Janesko, R. Gomperts, B. Mennucci, H. P. Hratchian, J. V. Ortiz, A. F. Izmaylov, J. L. Sonnenberg, D. Williams-Young, F. Ding, F. Lipparini, F. Egidi, J. Goings, B. Peng, A. Petrone, T. Henderson, D. Ranasinghe, V. G. Zakrzewski, J. Gao, N. Rega, G. Zheng, W. Liang, M. Hada, M. Ehara, K. Toyota, R. Fukuda, J. Hasegawa, M. Ishida, T. Nakajima, Y. Honda, O. Kitao, H. Nakai, T. Vreven, K. Throssell, J. A. Montgomery, Jr., J. E. P.,F. Ogliaro, M. J. Bearpark, J. J. Heyd, E. N. Brothers, K., N. Kudin, V. N. Staroverov, T. A. Keith, R. Kobayashi, J. Normand, K. Raghavachari, A. P. Rendell, J. C. Burant, S. S. Iyengar, J. Tomasi, M. Cossi, J. M. Millam, M. Klene, C. Adamo, R. Cammi, J. W. Ochterski, R. L. Martin, K. Morokuma, O. Farkas, J. B. Foresman, D. J. Fox. Citation | Gaussian.com. Gaussian 16, Revision C.01. https://gaussian.com/citation/ (accessed 2022-10-17).

[126] S. Grimme, J. Antony, S. Ehrlich, H. Krieg, *J. Chem. Phys.* **2010**, *132*, 154104 .

[127] V. Butera, *Phys. Chem. Chem. Phys.* **2024**, *26*, 7950–7970.

14. NMR spectra of NHPI esters

^{1}H NMR (400 MHz, $CDCl_3$) of **1f**

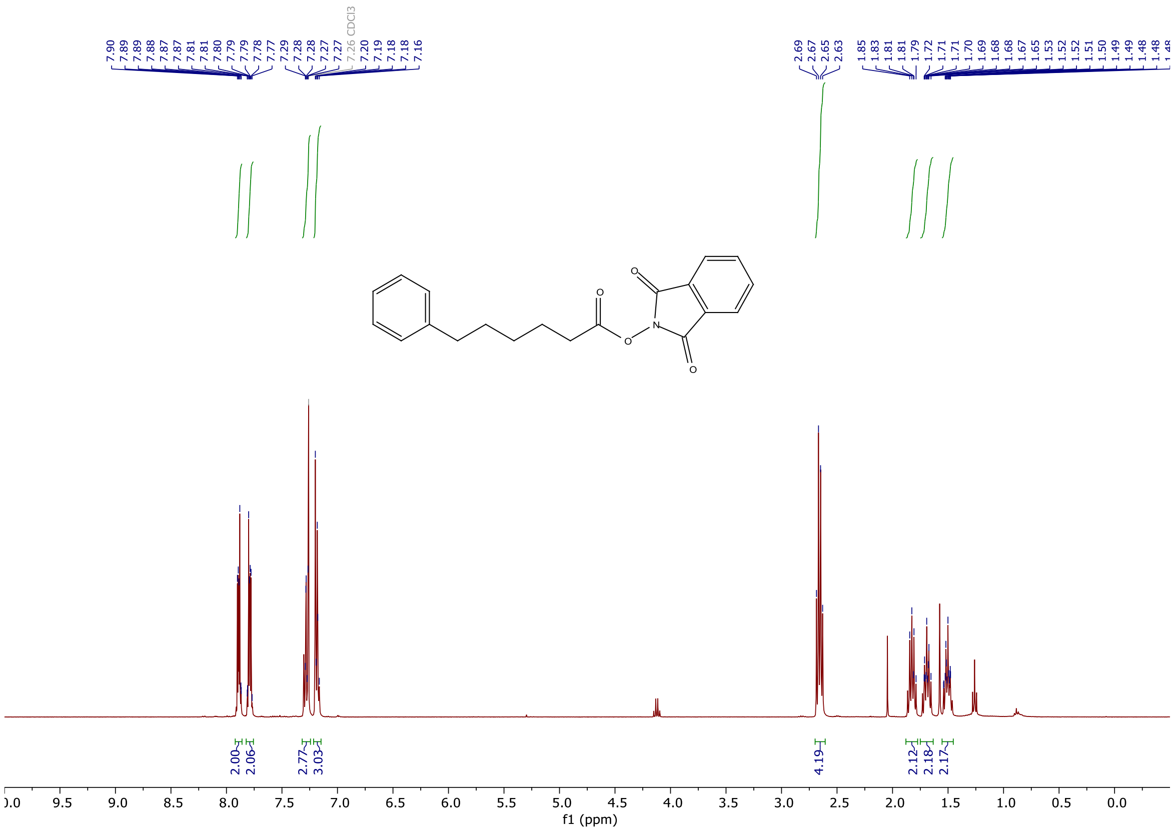

^{13}C NMR (101 MHz, $CDCl_3$) of **1f**

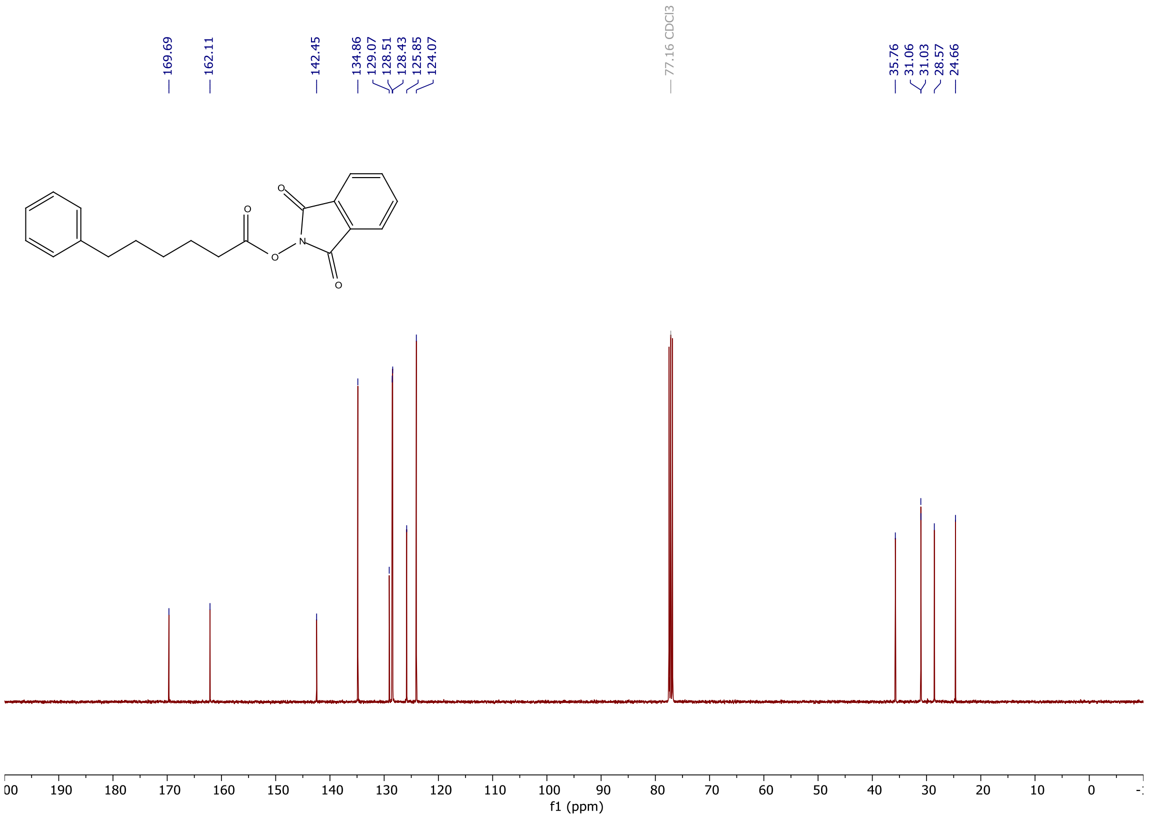

^1H NMR (400 MHz, $CDCl_3$) of **1i**

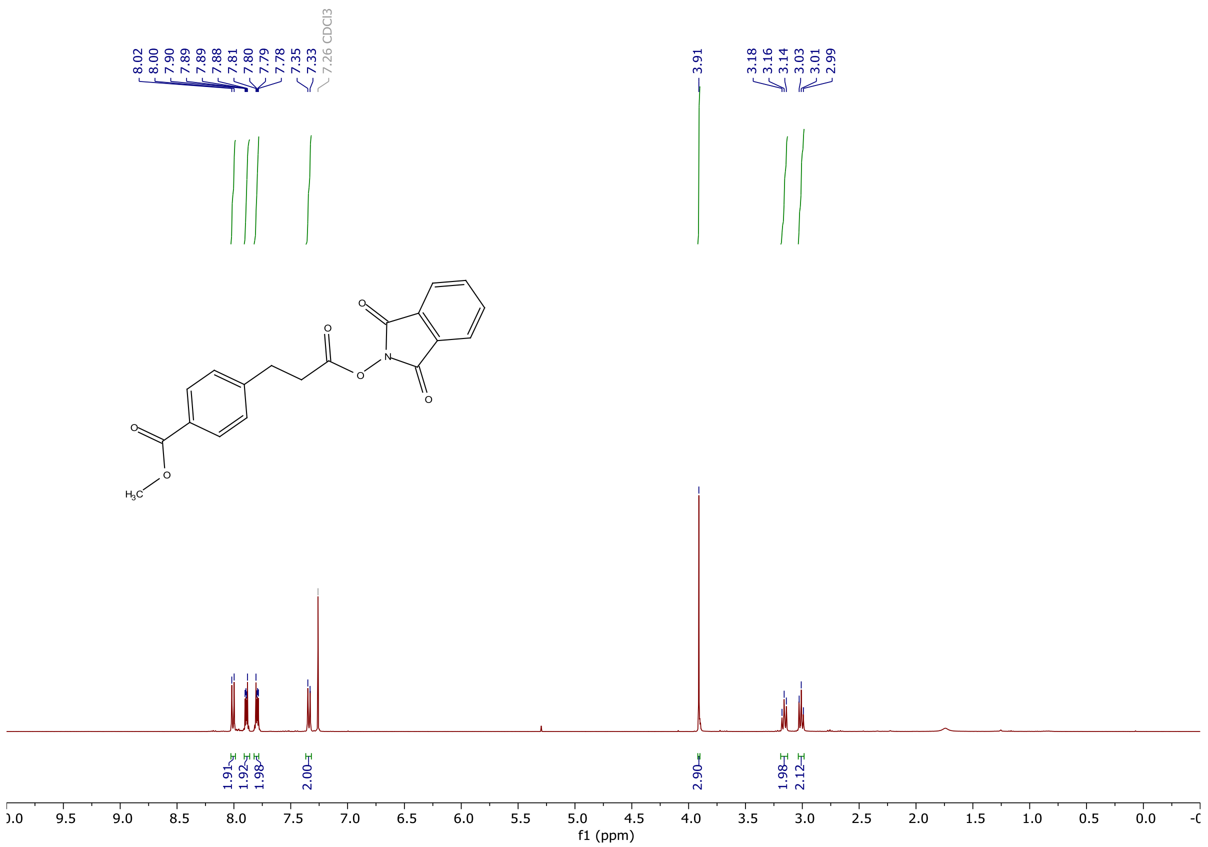

^{13}C NMR (101 MHz, $CDCl_3$) of **1i**

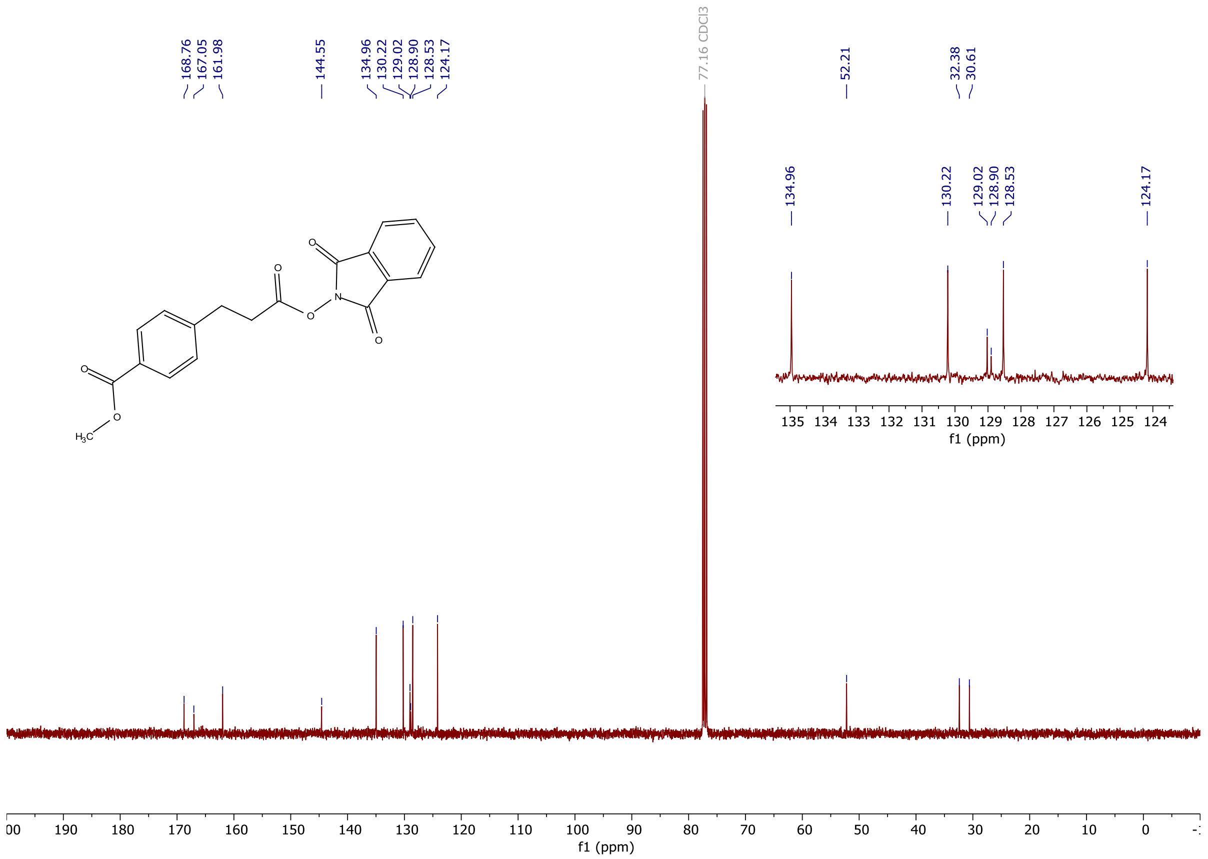

^{1}H NMR (400 MHz, $CDCl_3$) of **1j**

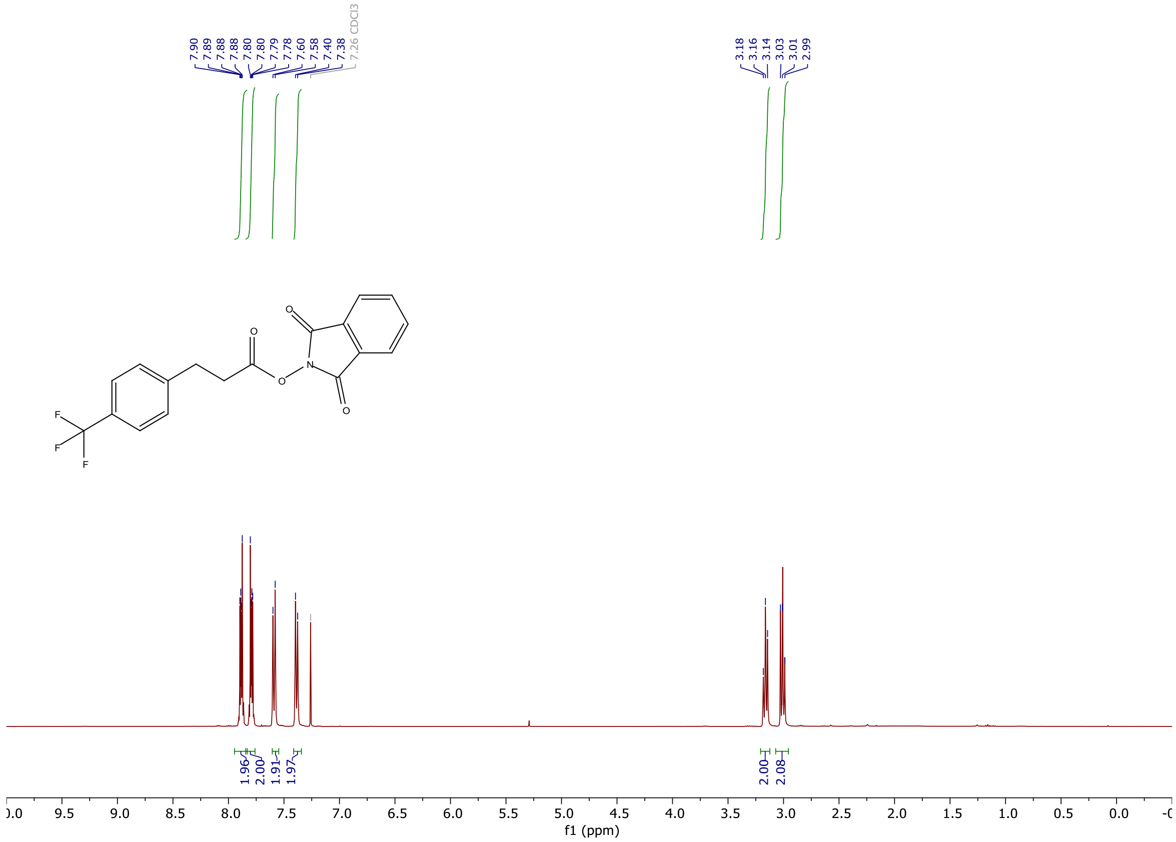

^{13}C NMR (101 MHz, $CDCl_3$) of **1j**

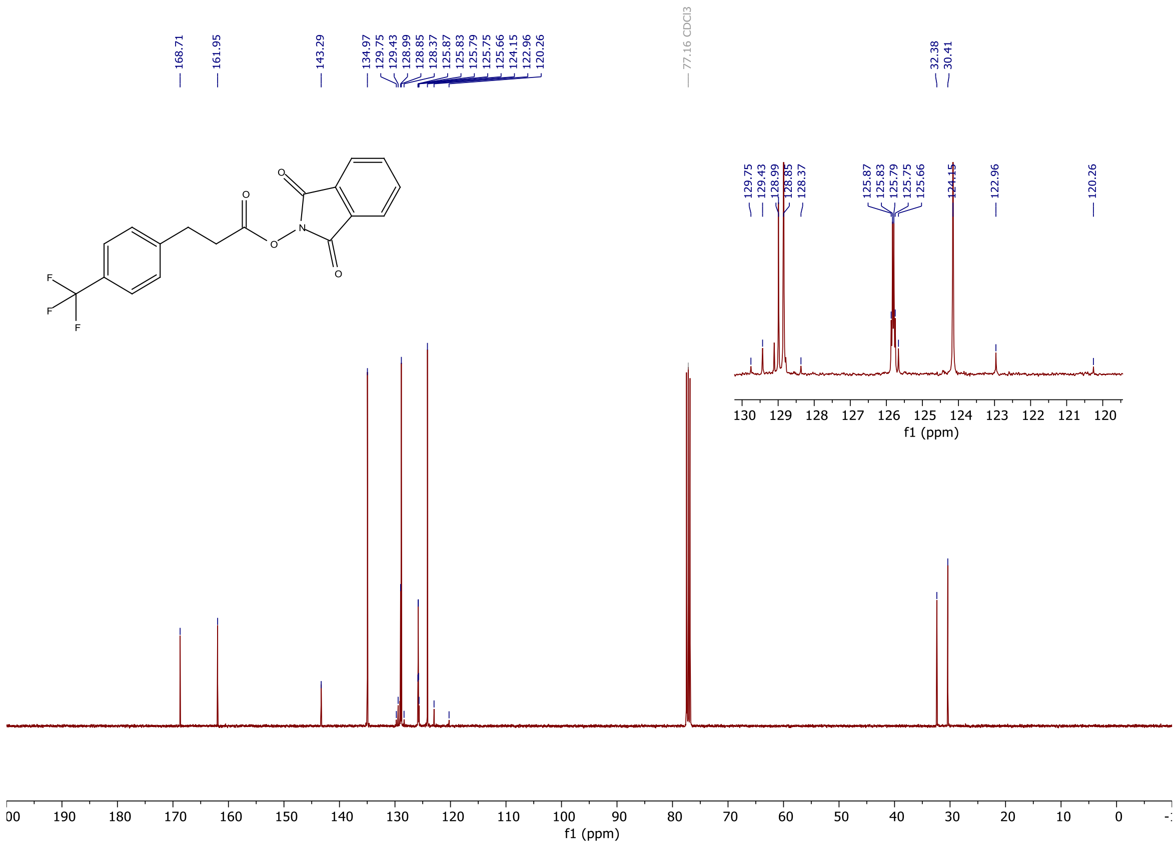

^{19}F NMR (376 MHz, $CDCl_3$) of **1j**

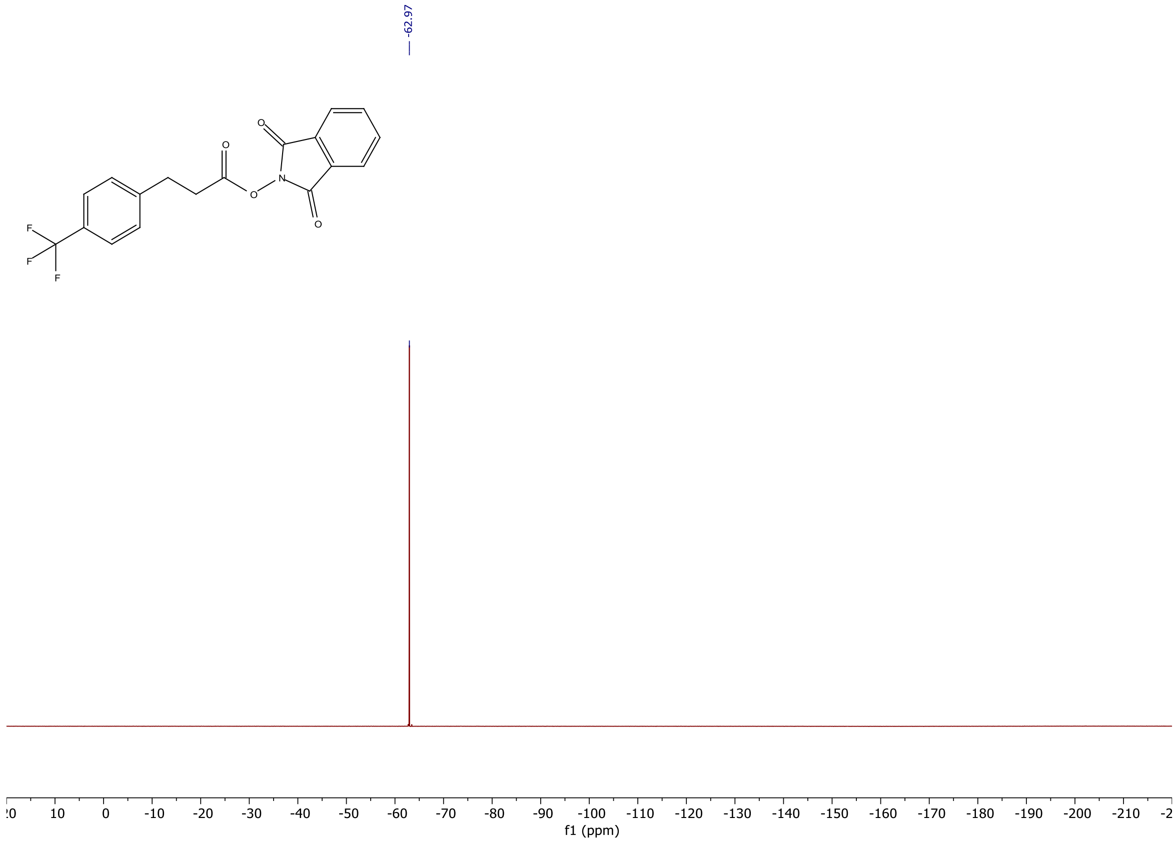

^{1}H NMR (400 MHz, $CDCl_3$) of **1m**

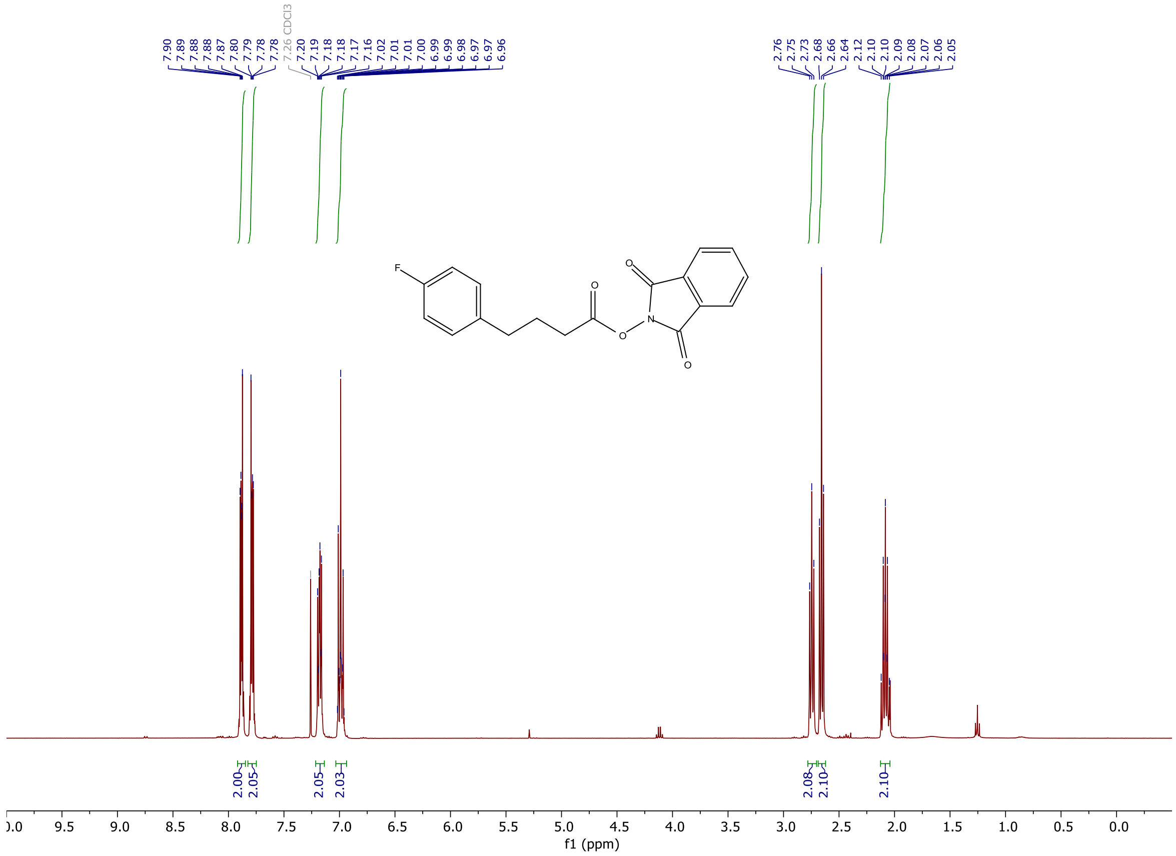

^{13}C NMR (101 MHz, $CDCl_3$) of **1m**

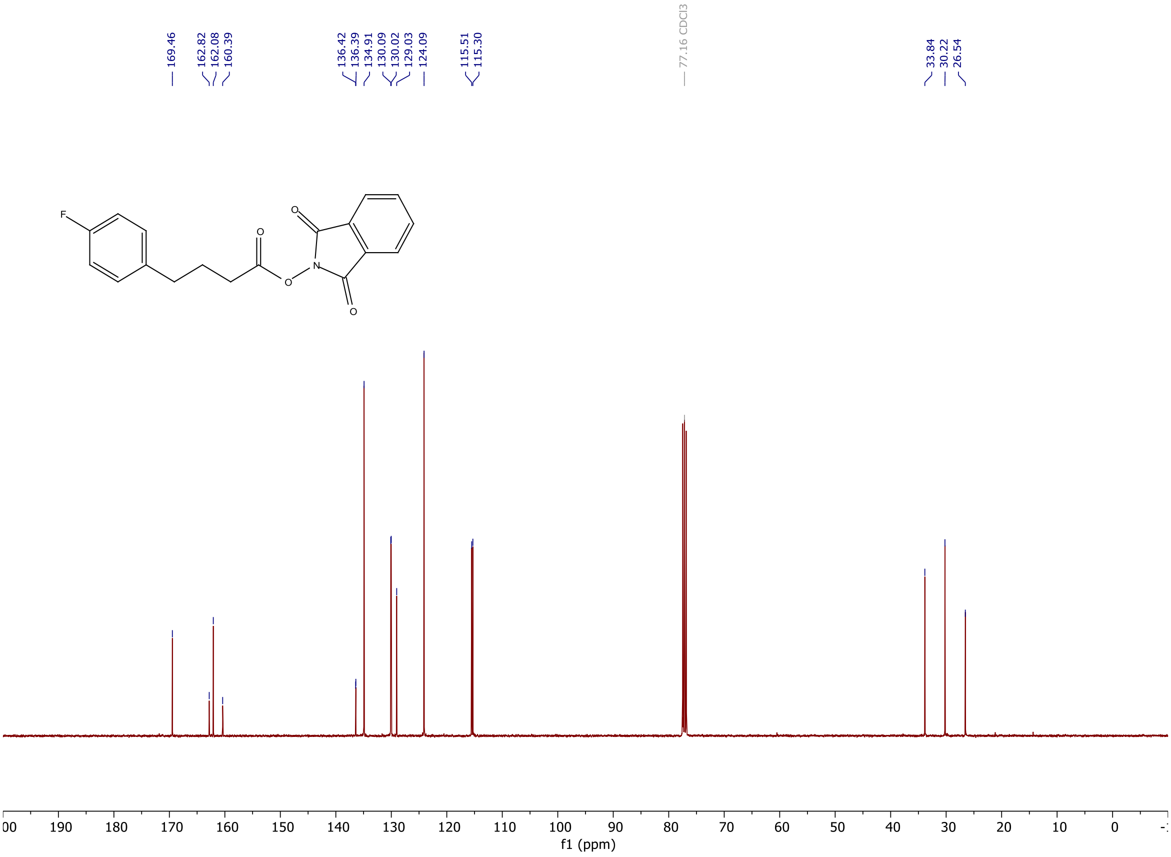

^{19}F NMR (376 MHz, $CDCl_3$) of **1m**

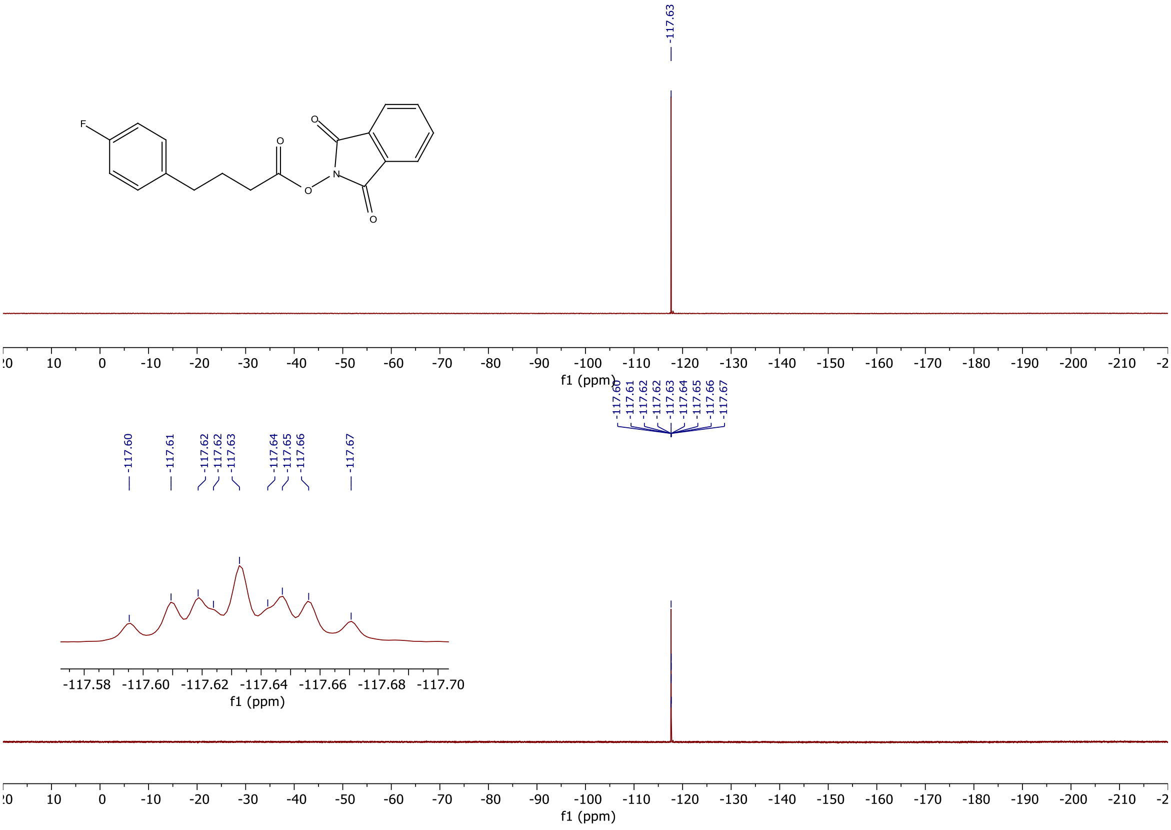

^{1}H NMR (400 MHz, $CDCl_3$) of **1n**

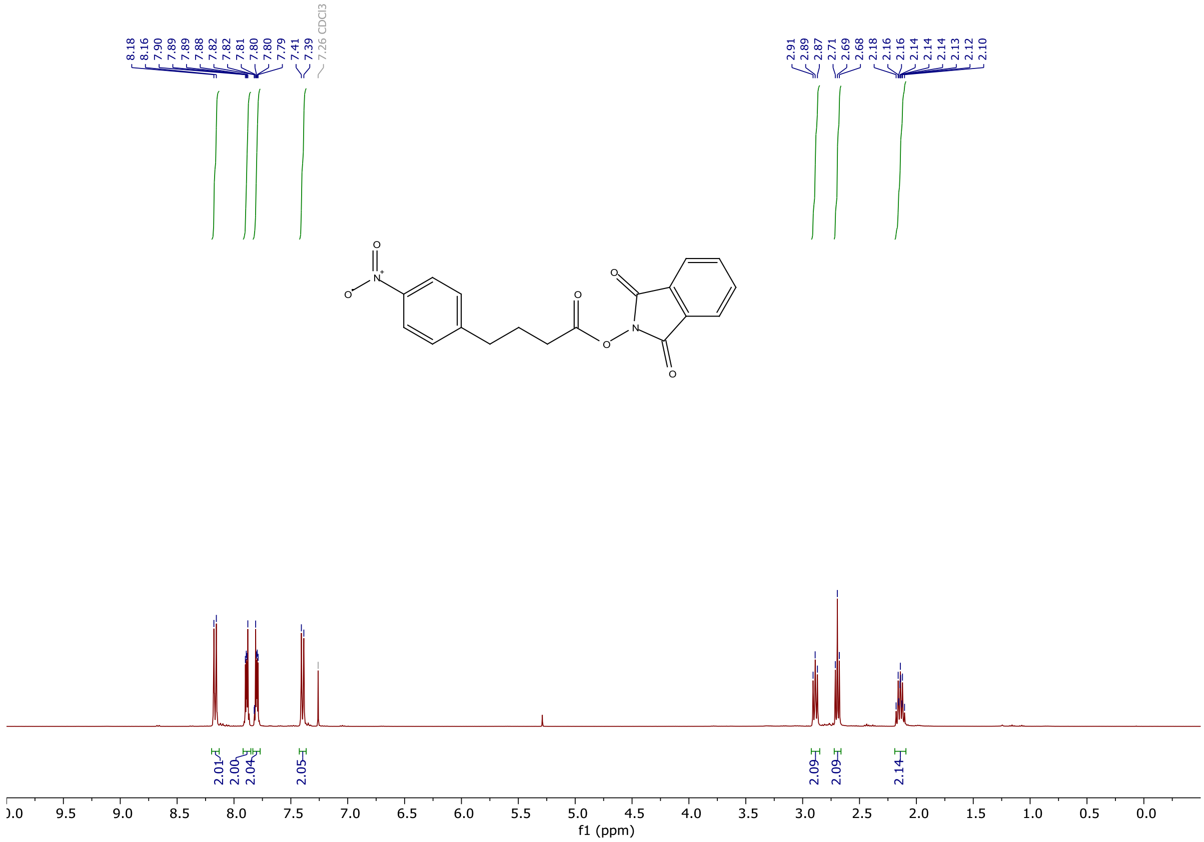

^{13}C NMR (101 MHz, $CDCl_3$) of **1n**

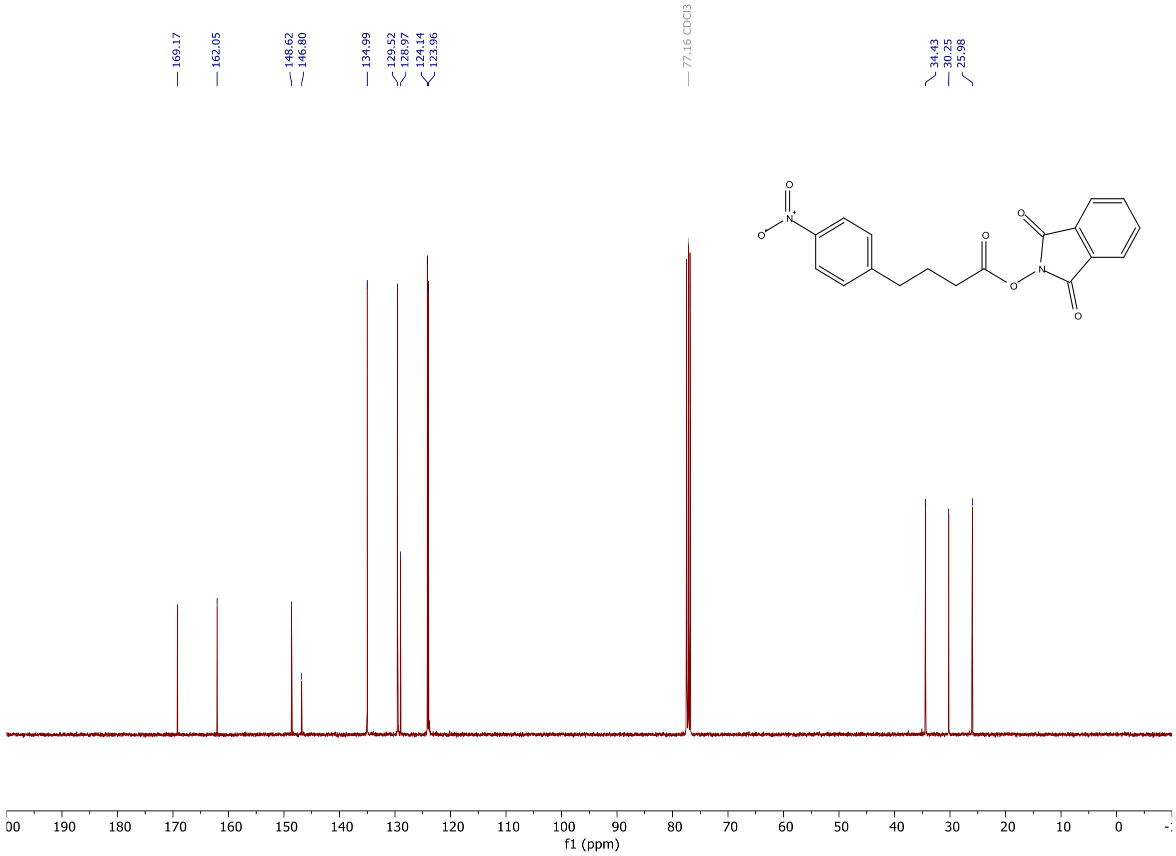

15. NMR spectra of Ritter-type carboamidation products

^{1}H NMR (400 MHz, $CDCl_3$) of **4**

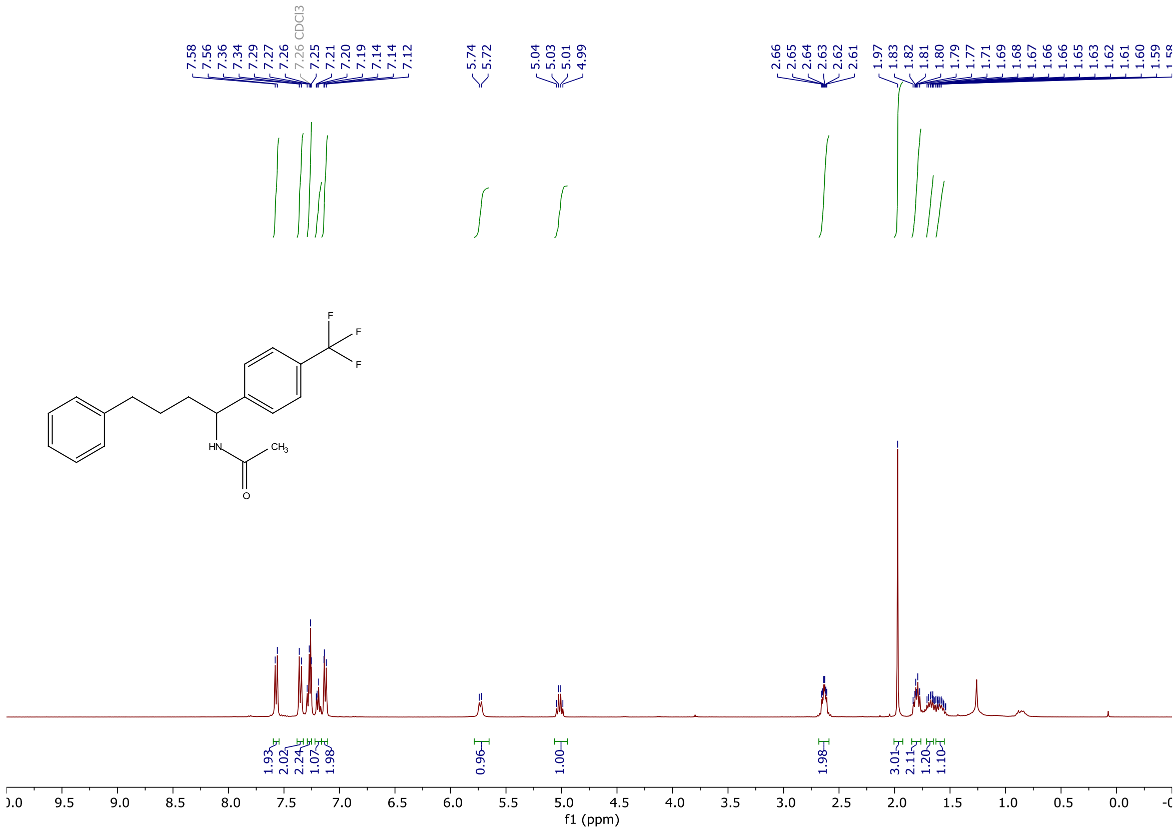

^{13}C NMR (151 MHz, $CDCl_3$) of **4**

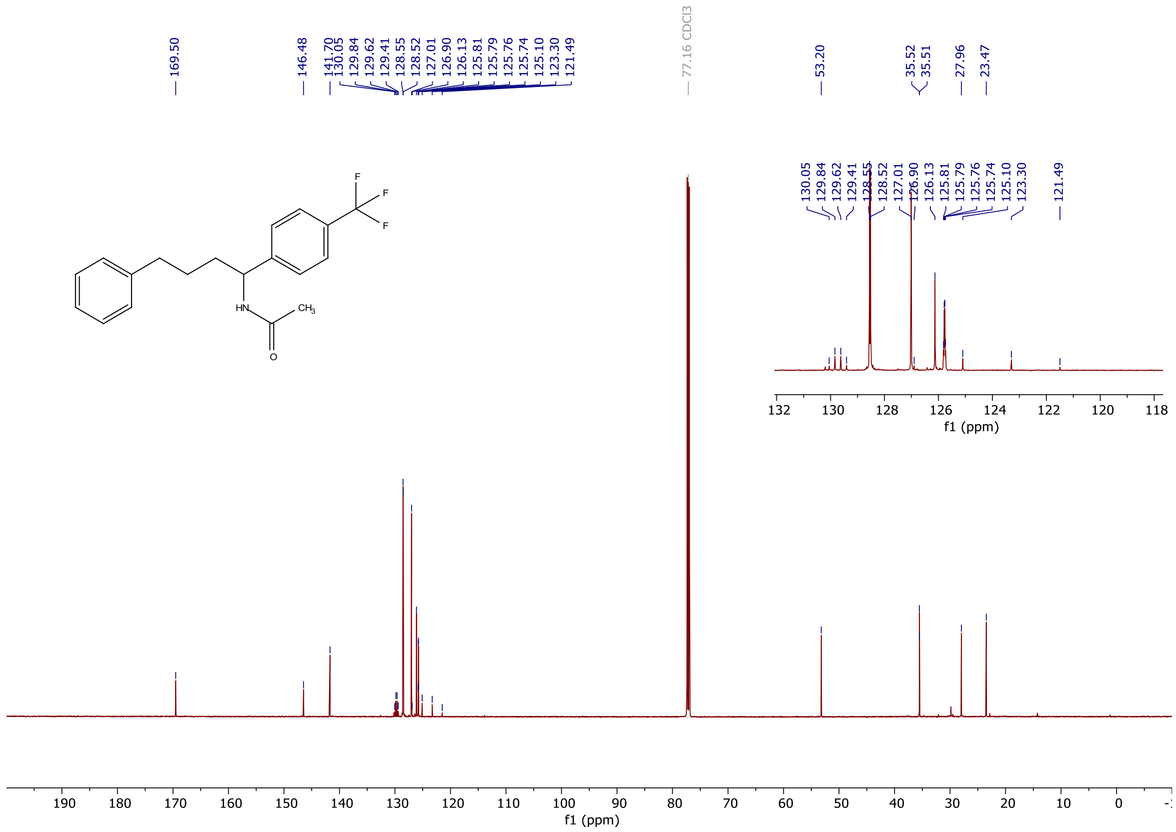

^{19}F NMR (376 MHz, $CDCl_3$) of **5**

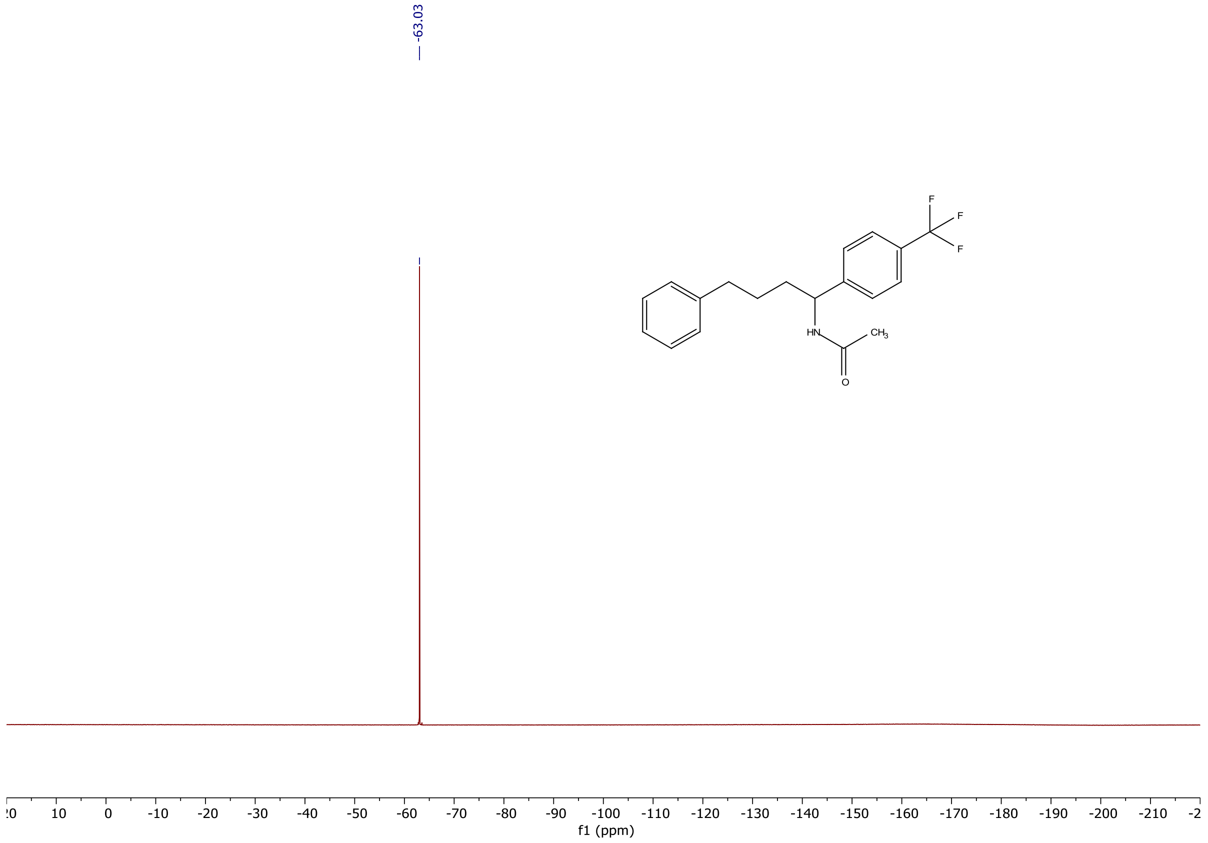

^{1}H NMR (400 MHz, $CDCl_3$) of **5**

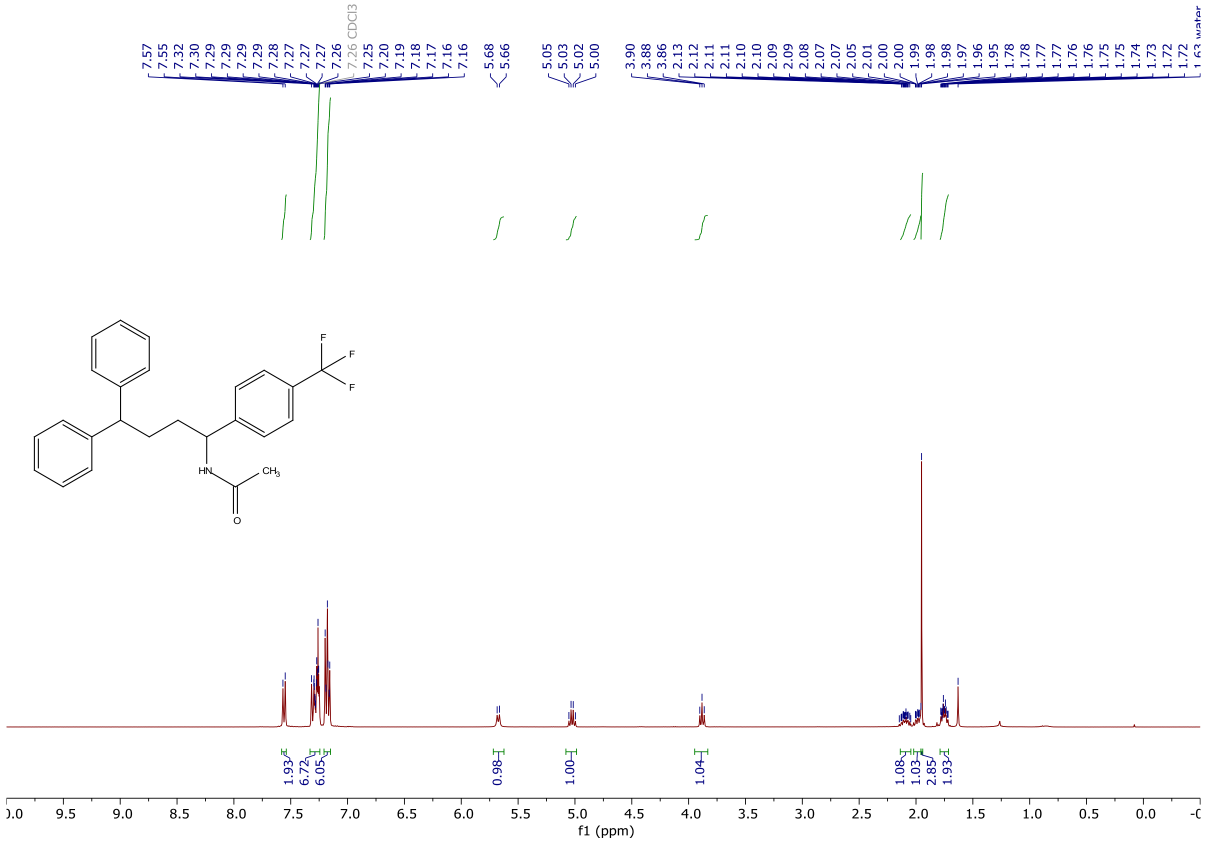

^{13}C NMR (151 MHz, $CDCl_3$) of **5**

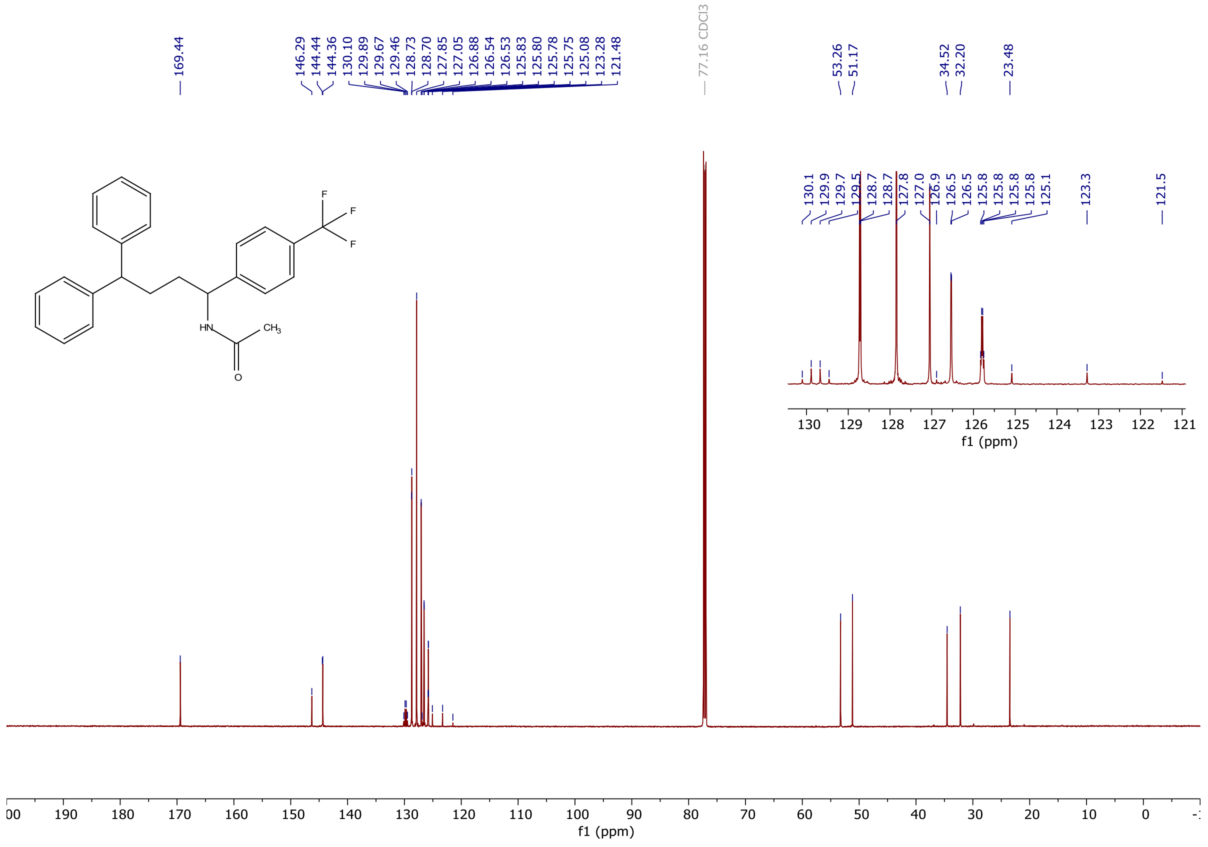

^{19}F NMR (376 MHz, $CDCl_3$) of **5**

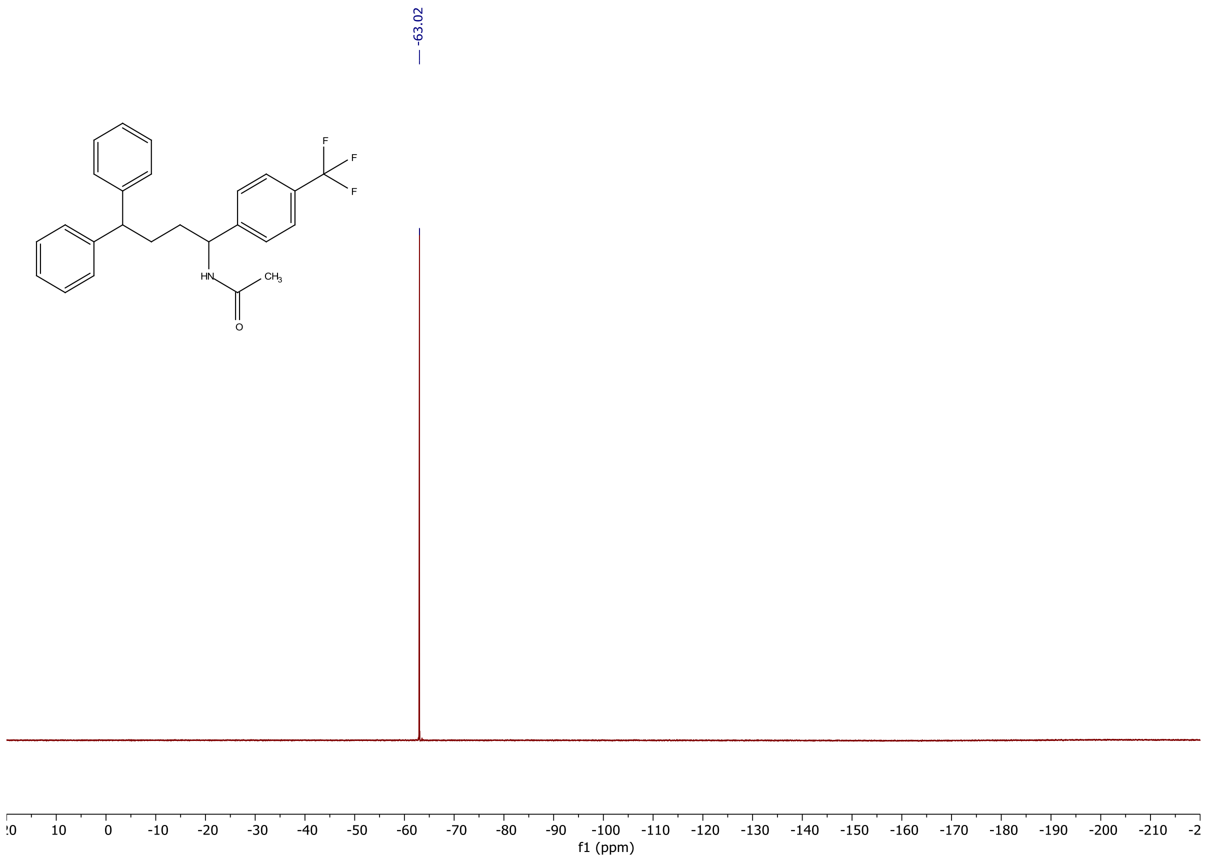

^{1}H NMR (400 MHz, $CDCl_3$) of **6**

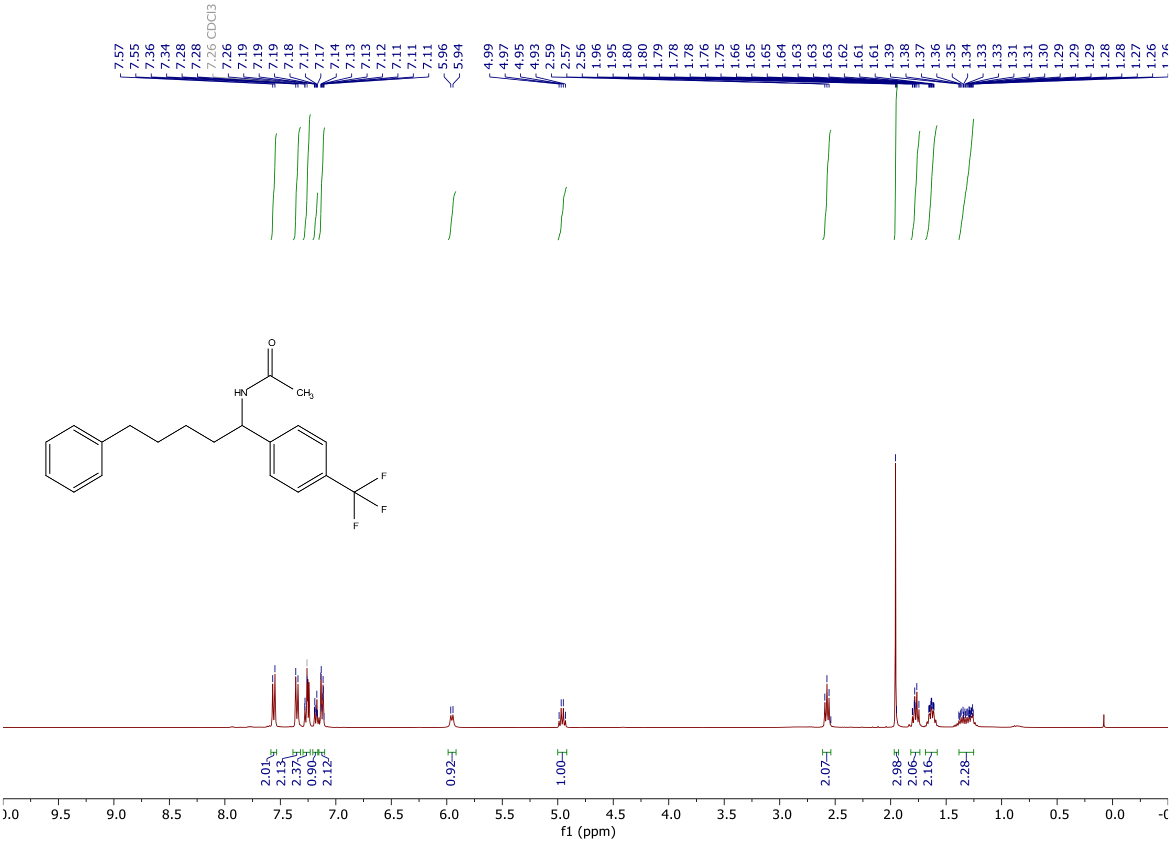

^{13}C NMR (151 MHz, $CDCl_3$) of **6**

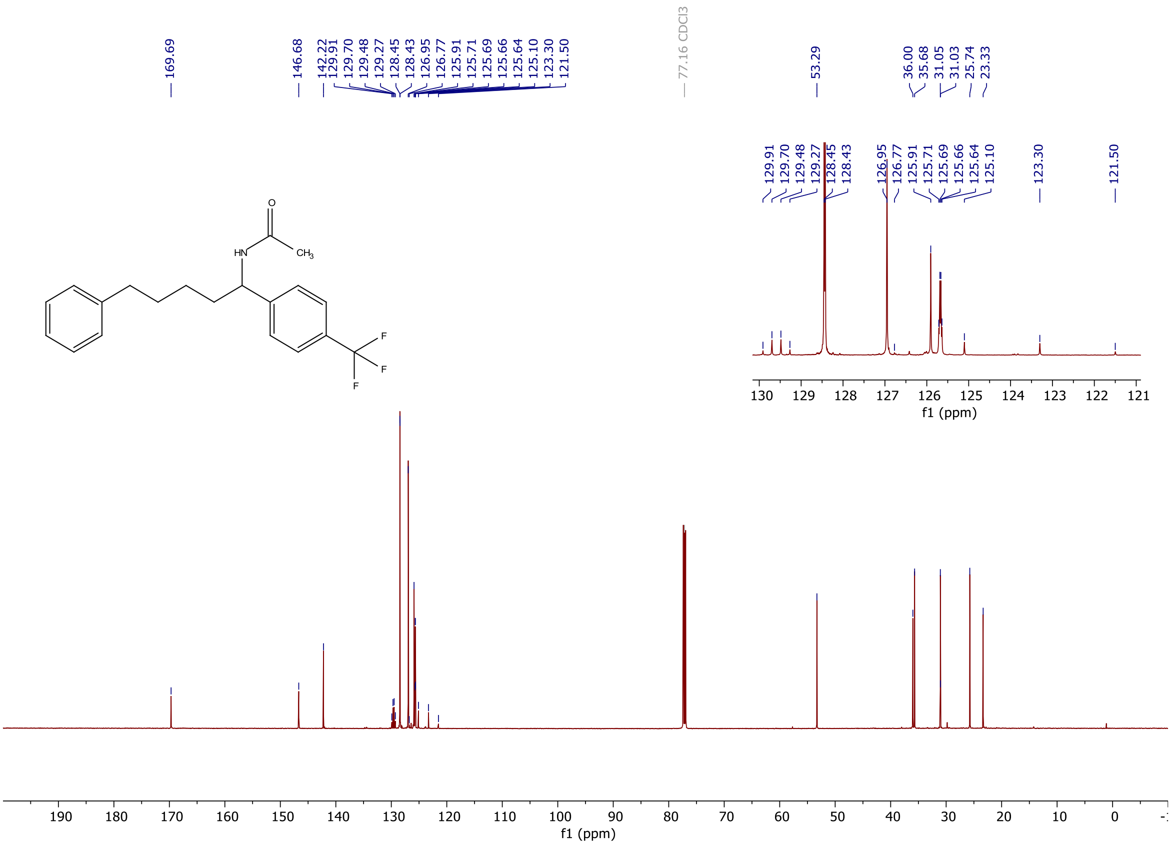

^{19}F NMR (376 MHz, $CDCl_3$) of **6**

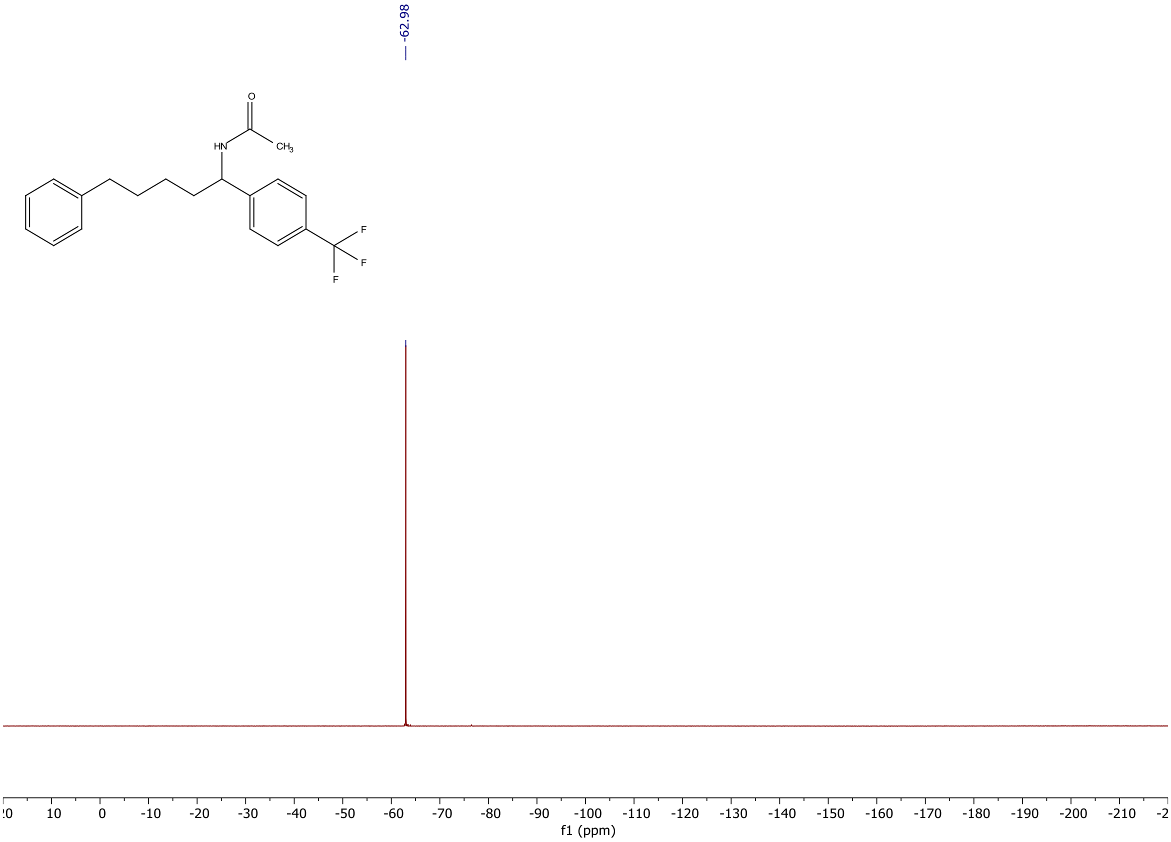

^{1}H NMR (400 MHz, $CDCl_3$) of **7**

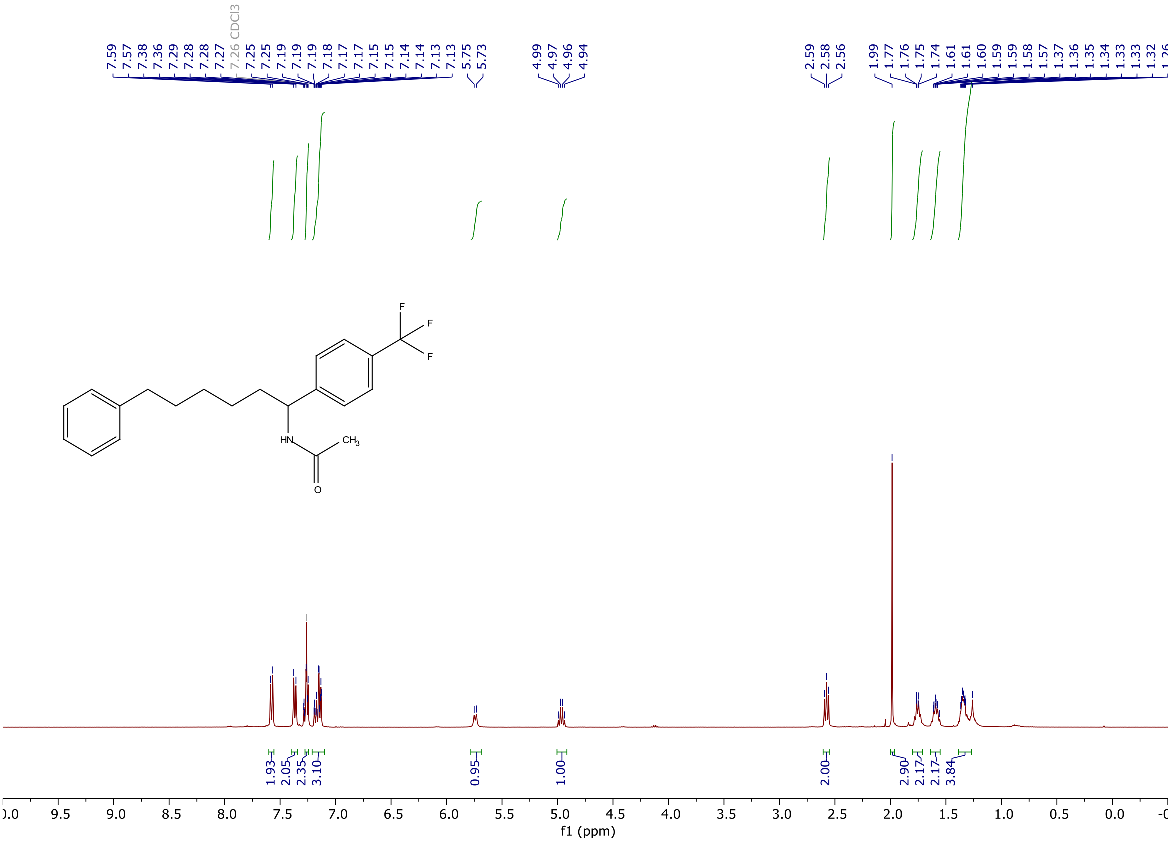

^{13}C NMR (151 MHz, $CDCl_3$) of **7**

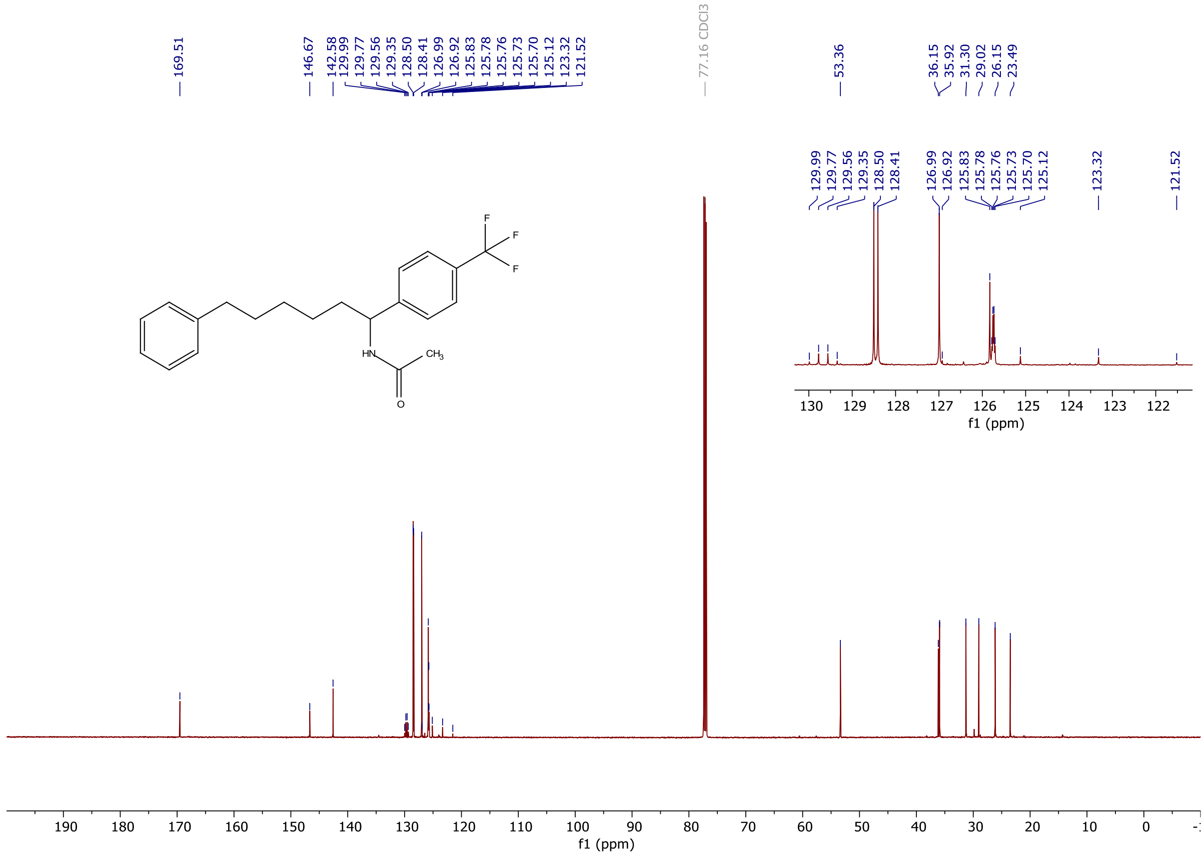

^{19}F NMR (376 MHz, $CDCl_3$) of **7**

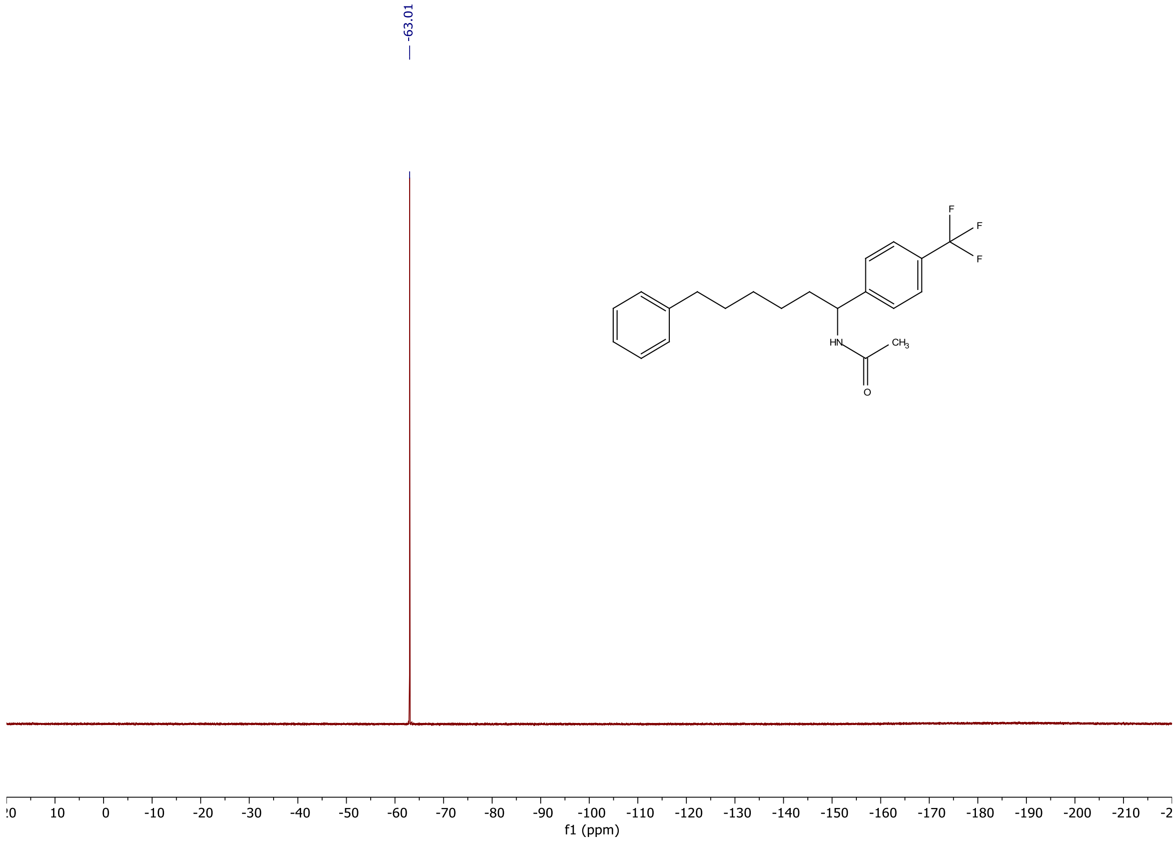

^1H NMR (400 MHz, $CDCl_3$) of **8**

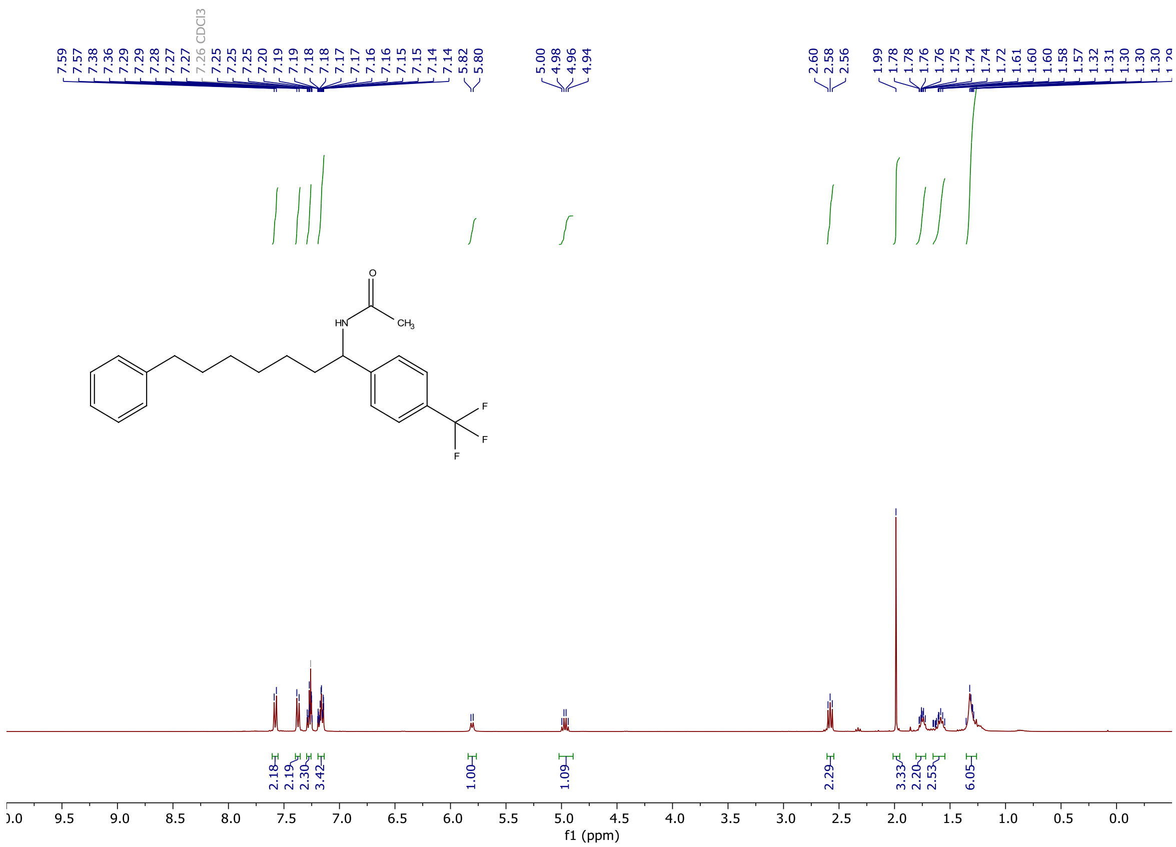

^{13}C NMR (151 MHz, $CDCl_3$) of **8**

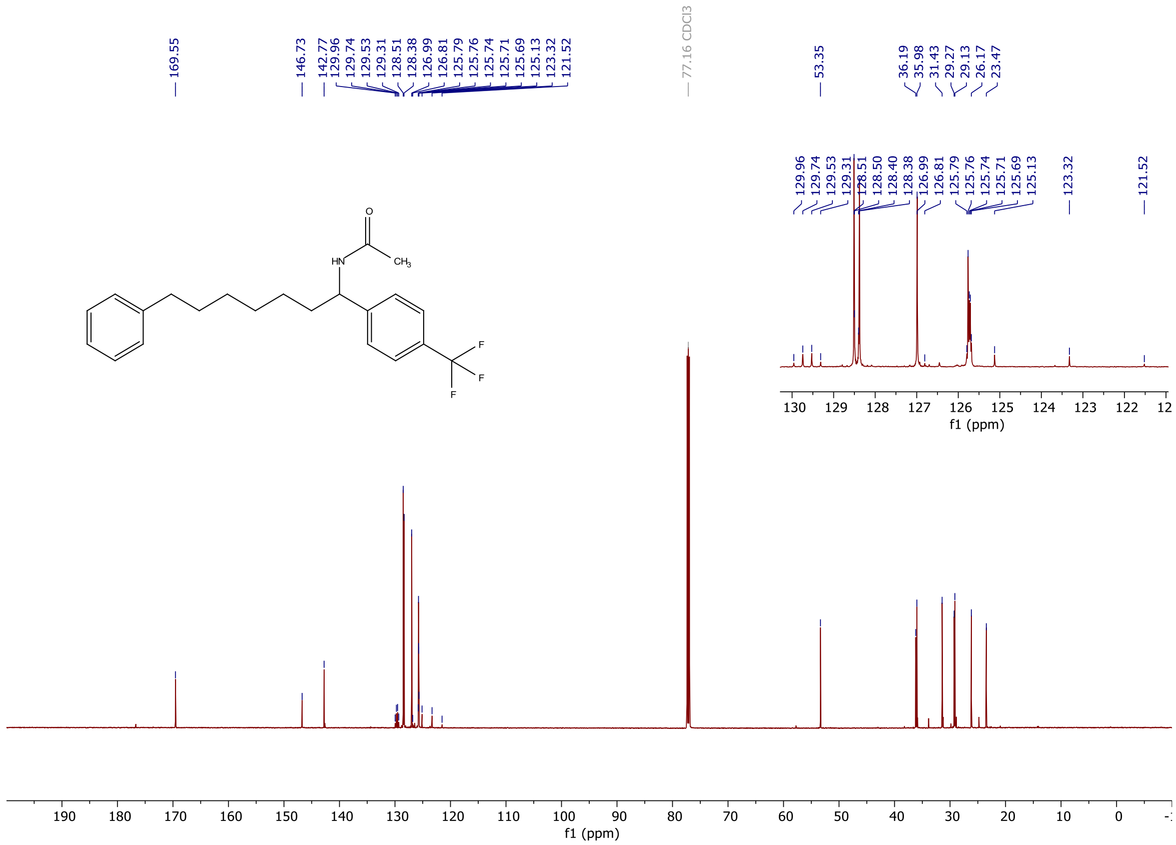

^{19}F NMR (376 MHz, $CDCl_3$) of **8**

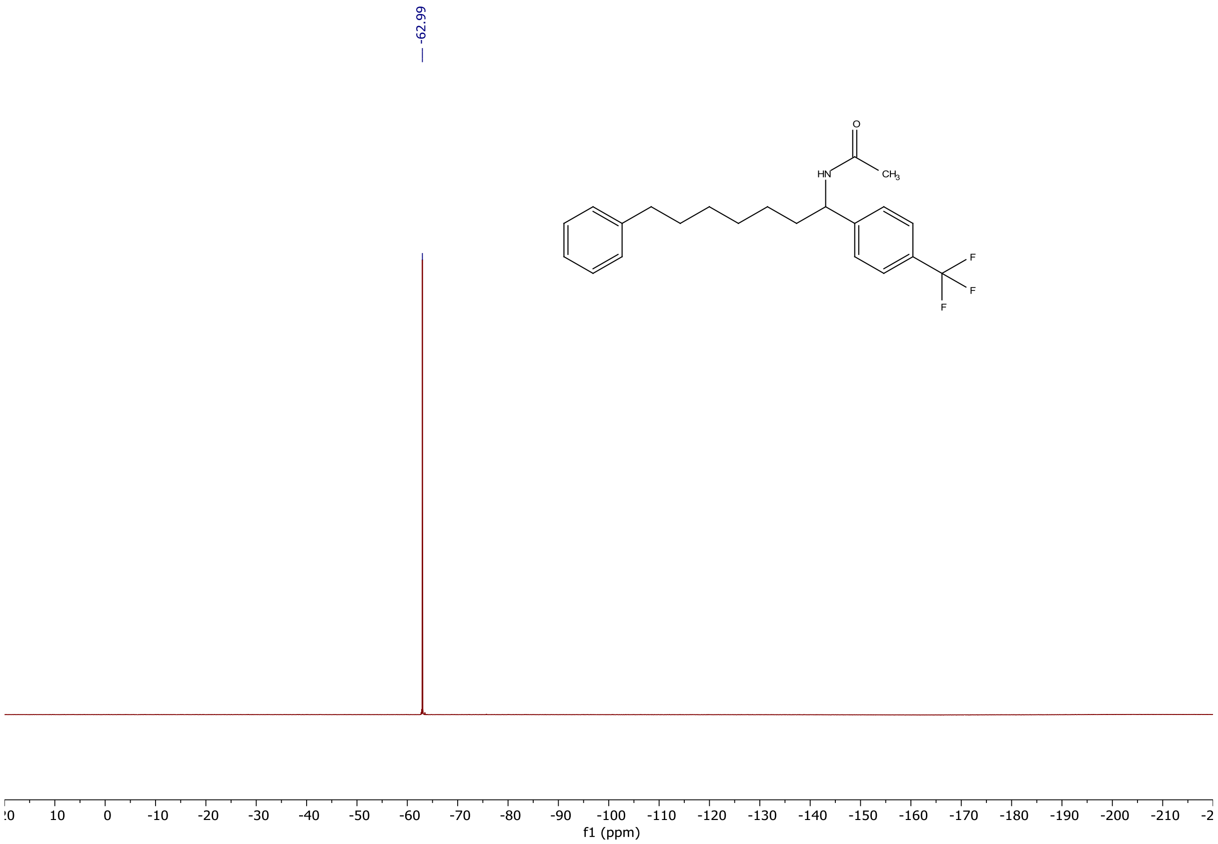

^{1}H NMR (400 MHz, $CDCl_3$) of **9**

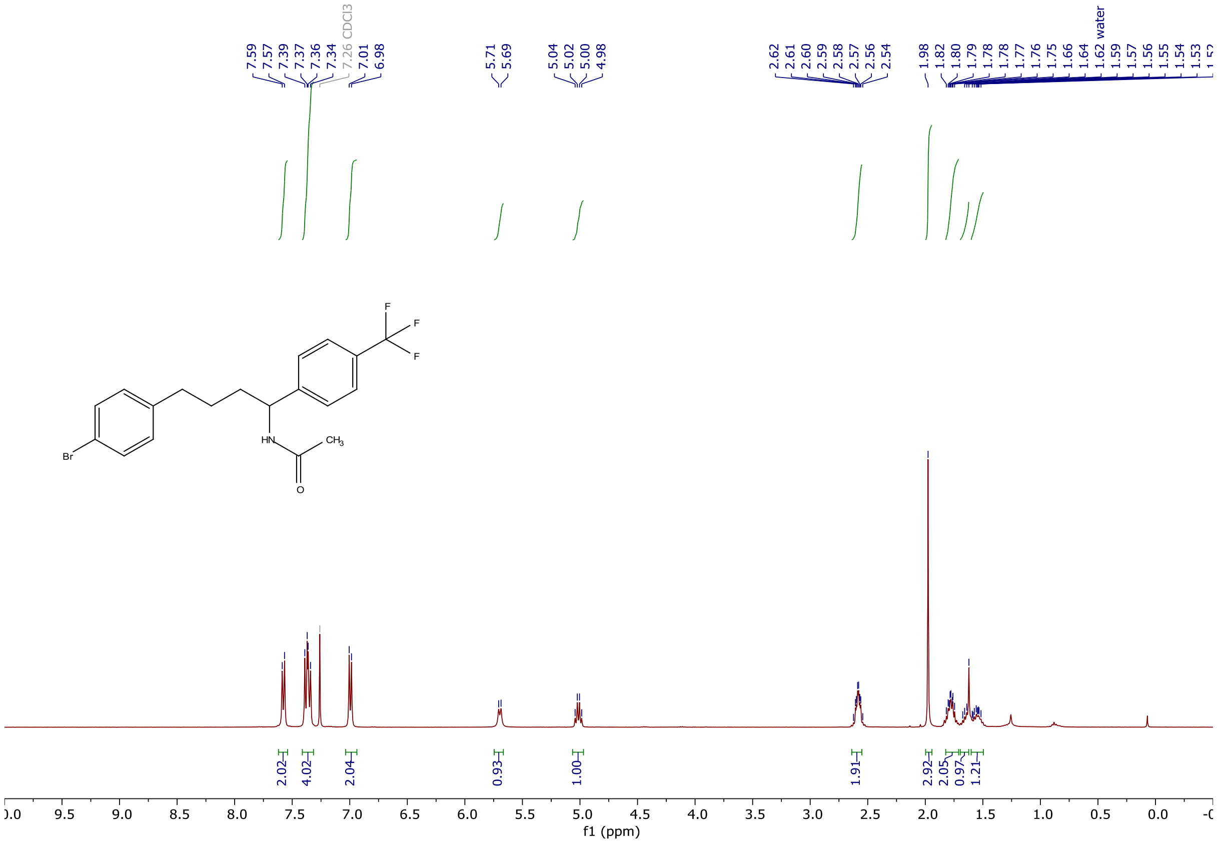

^{13}C NMR (151 MHz, $CDCl_3$) of **9**

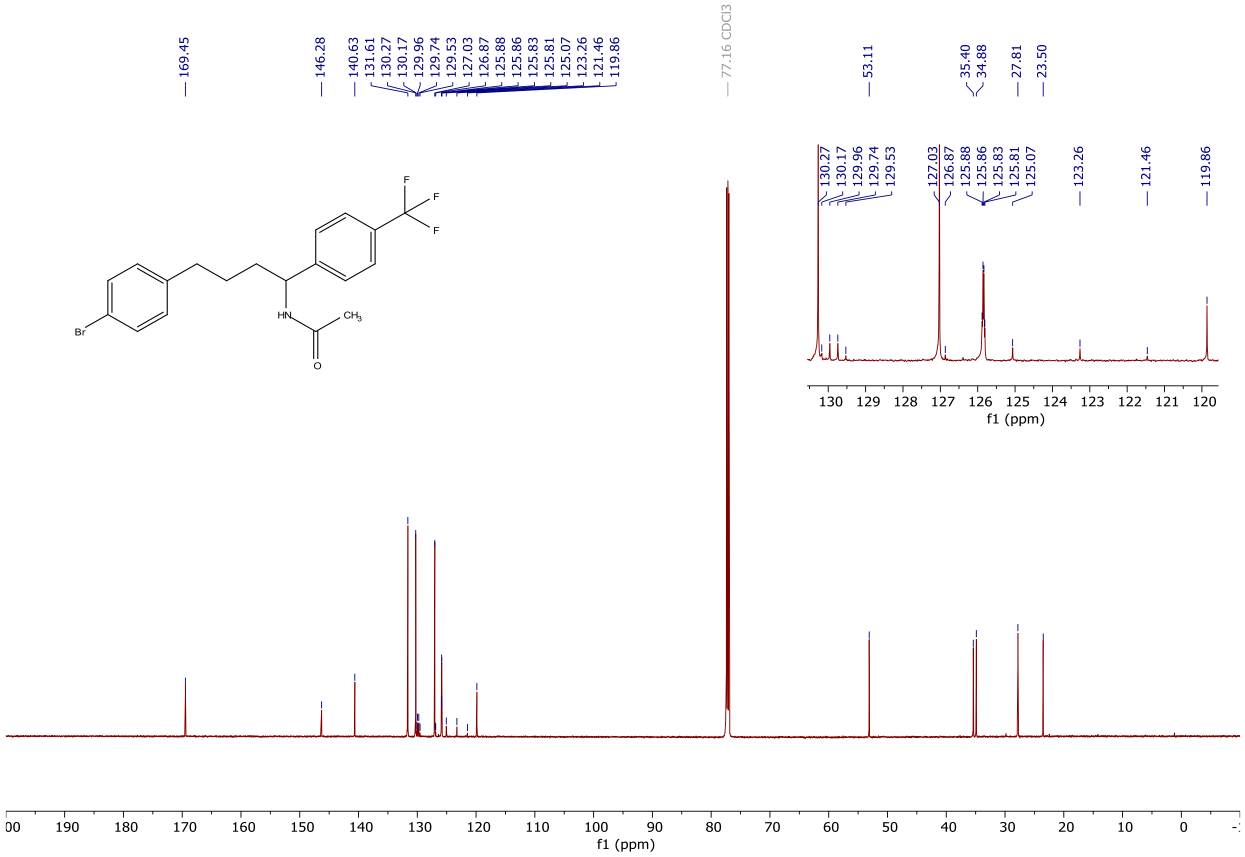

^{19}F NMR (376 MHz, $CDCl_3$) of **9**

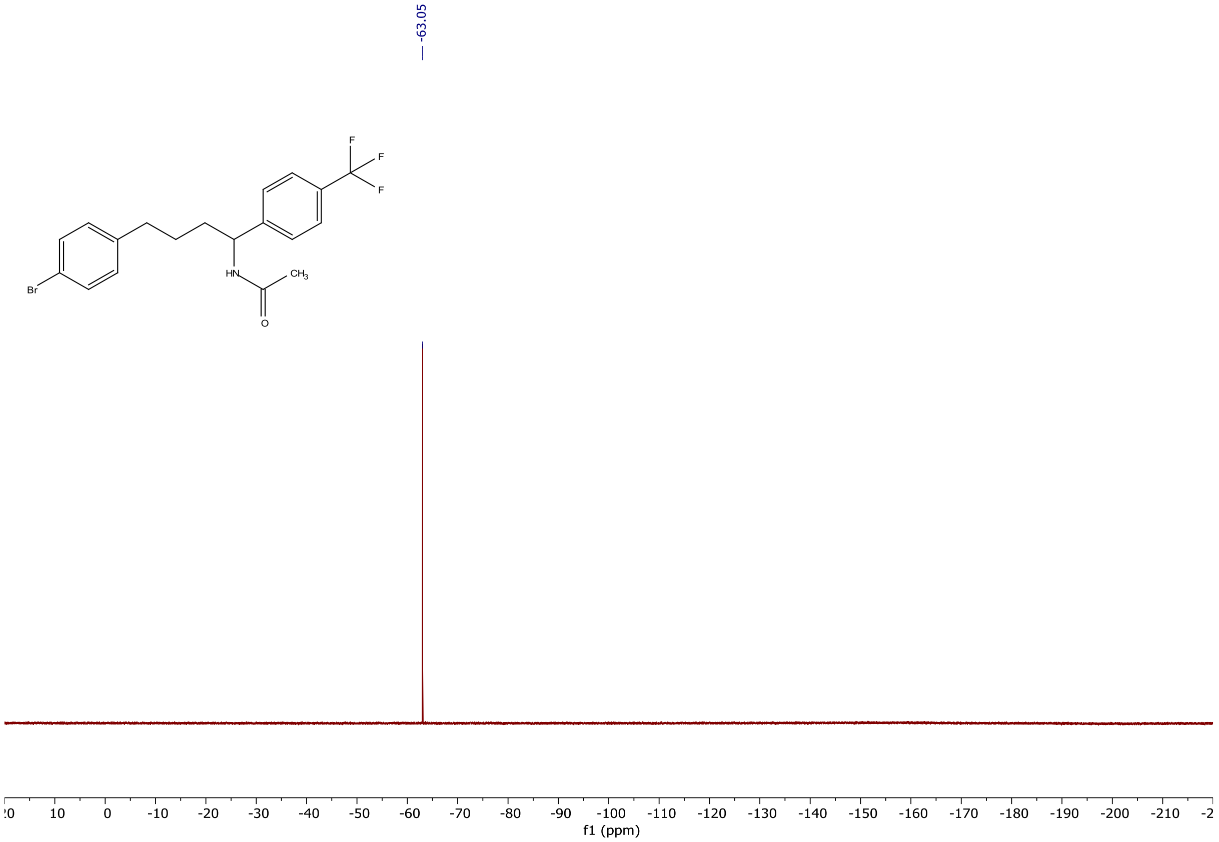

^{1}H NMR (400 MHz, $CDCl_3$) of **10**

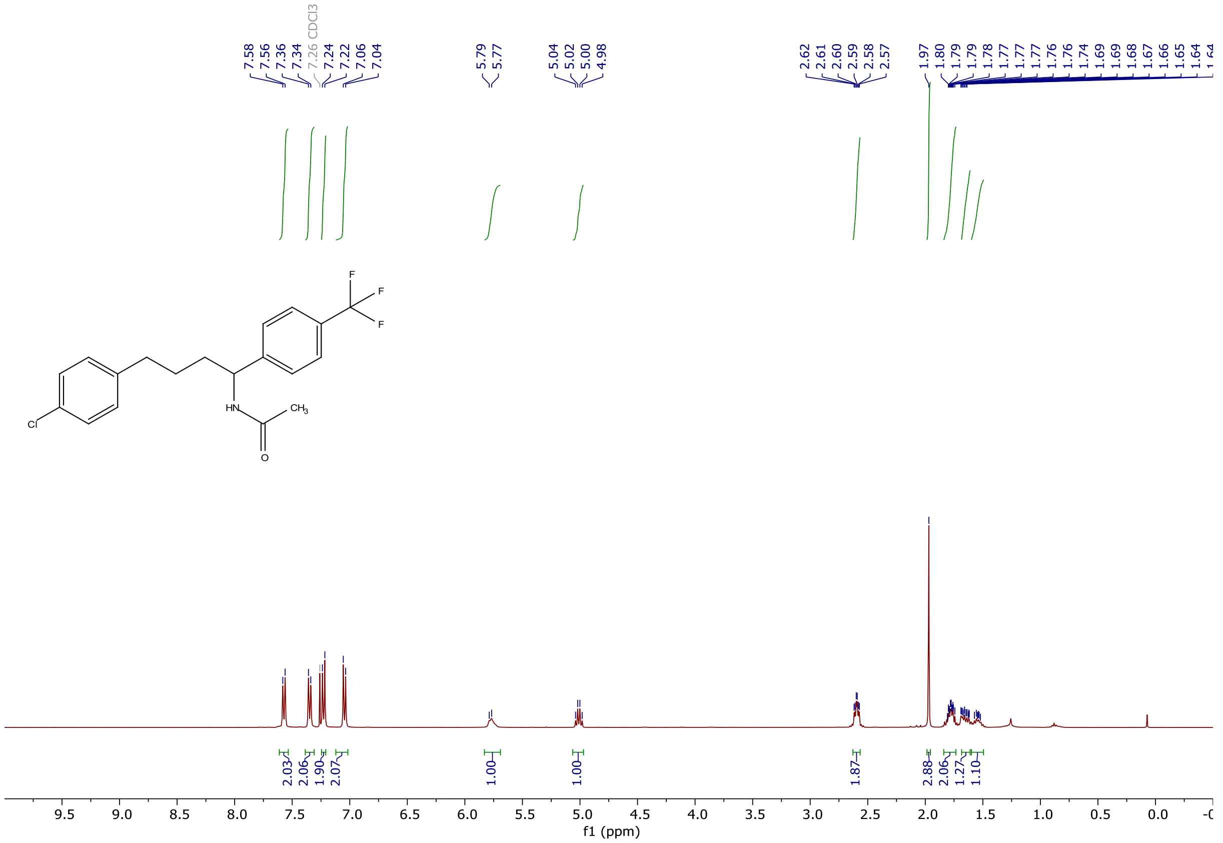

^{13}C NMR (151 MHz, $CDCl_3$) of **10**

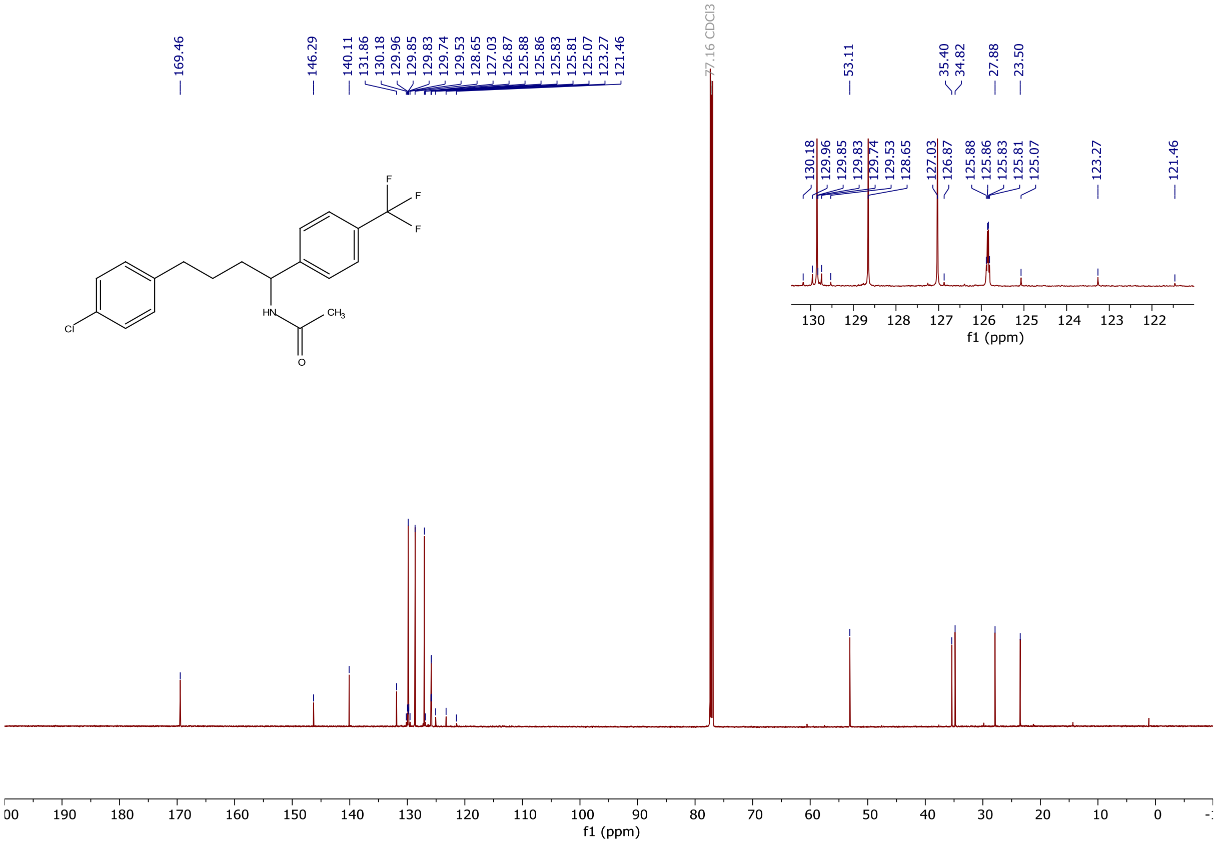

^{19}F NMR (376 MHz, $CDCl_3$) of **10**

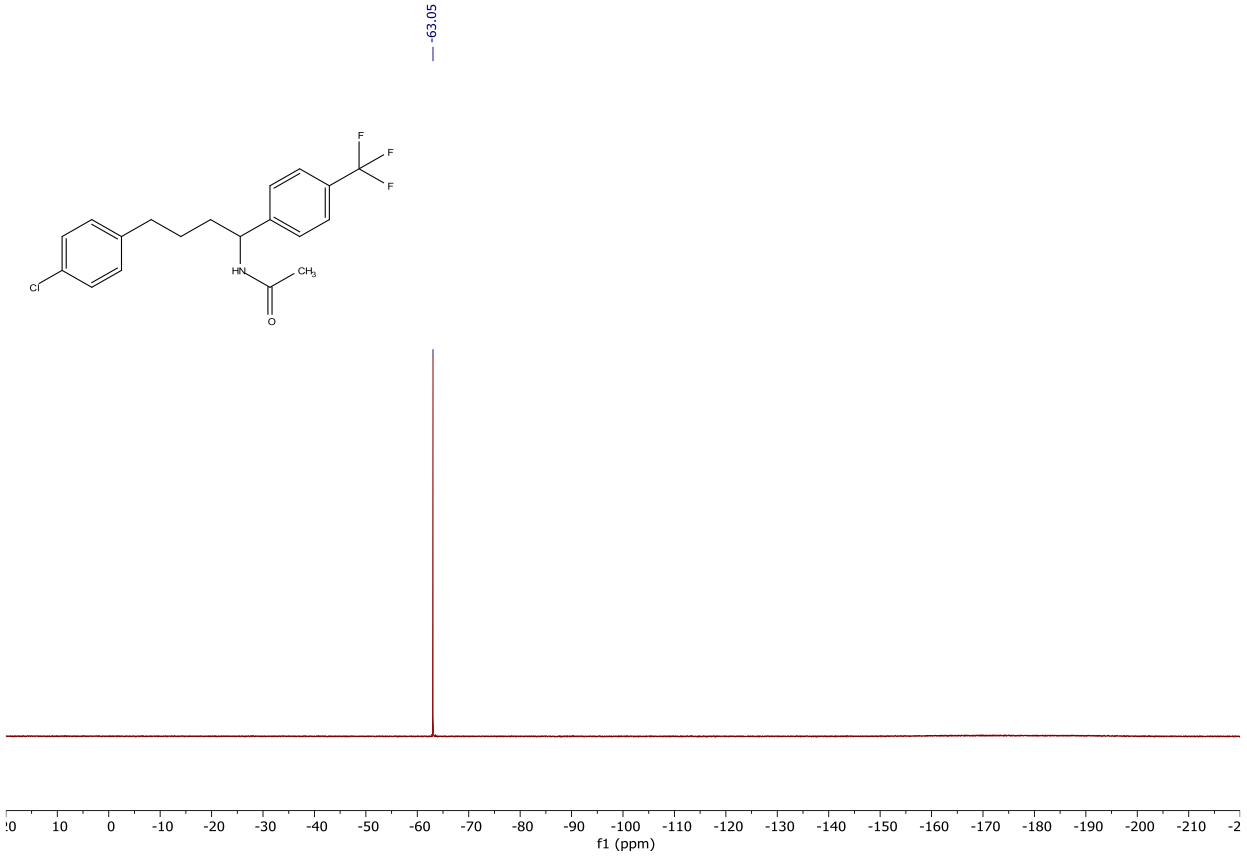

^{1}H NMR (400 MHz, $CDCl_3$) of **11**

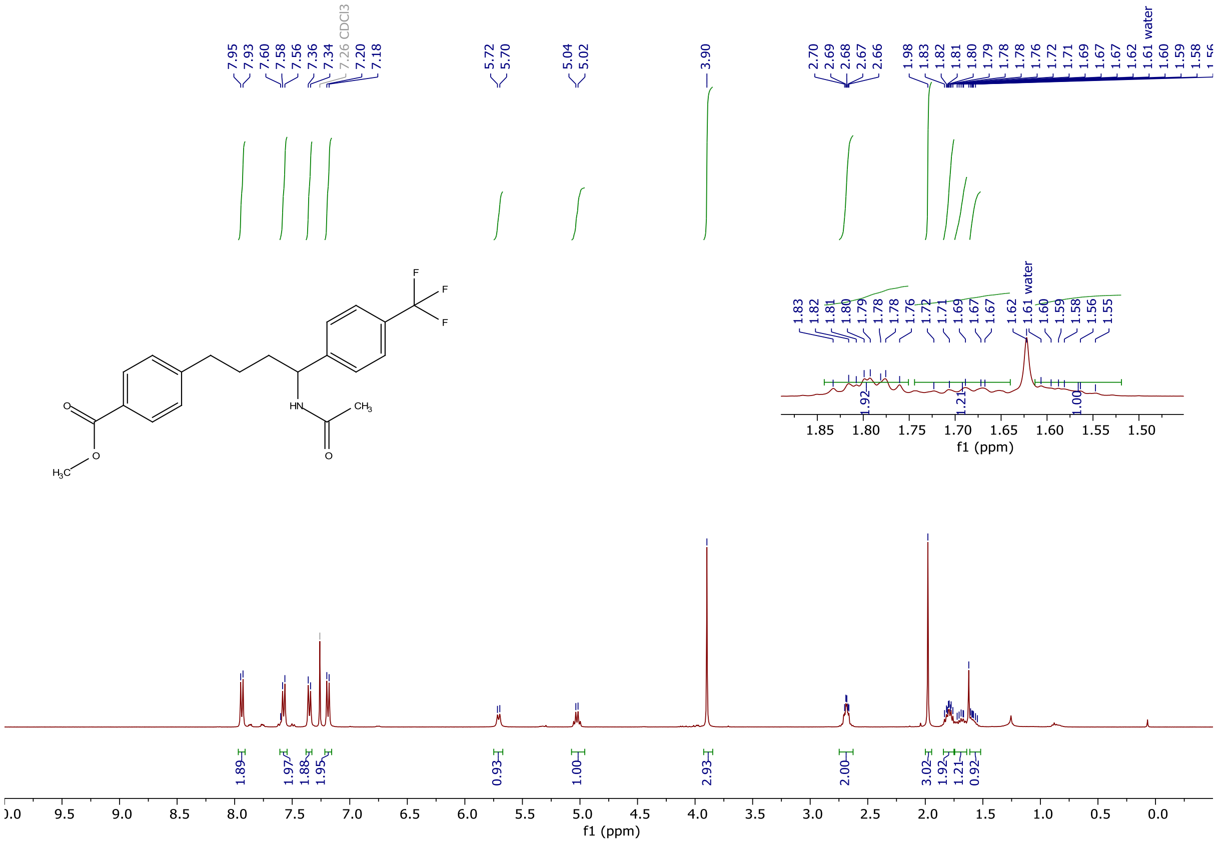

^{13}C NMR (151 MHz, $CDCl_3$) of **11**

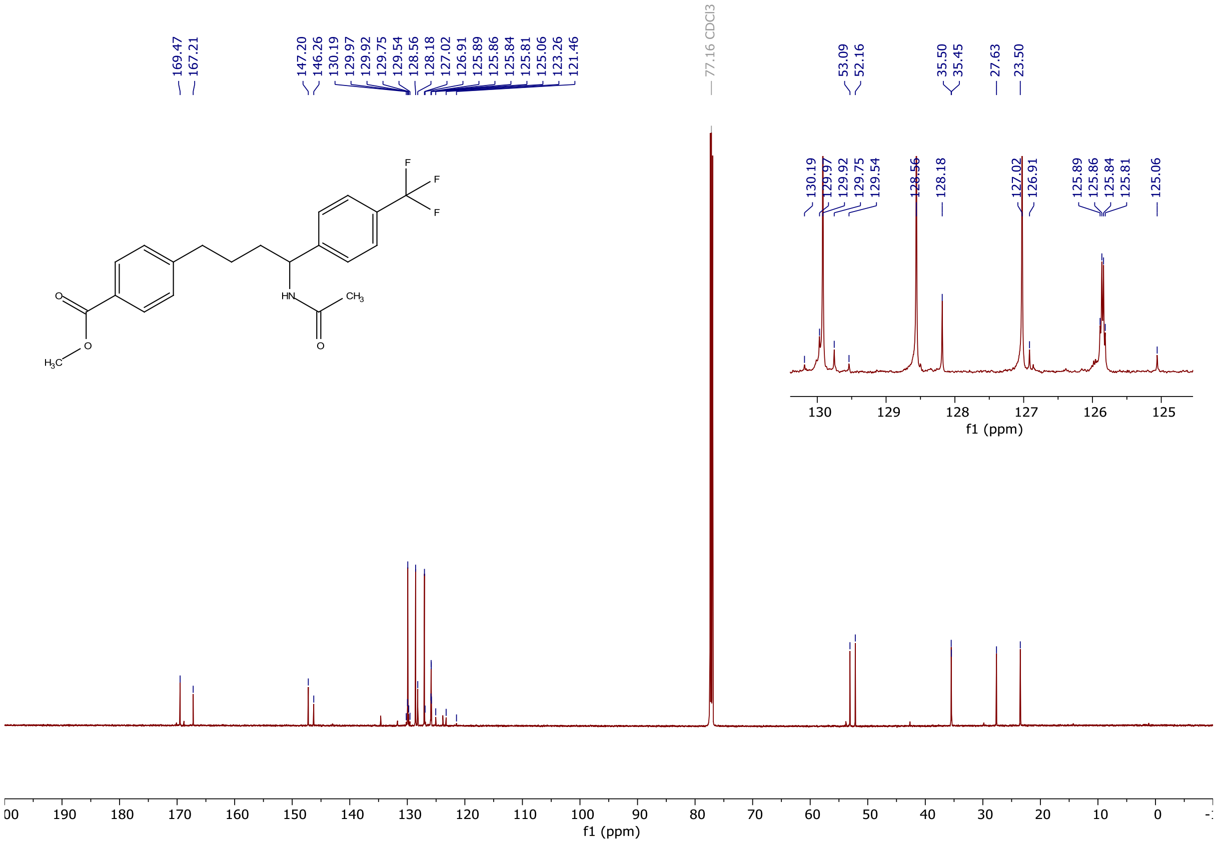

^{19}F NMR (376 MHz, $CDCl_3$) of **11**

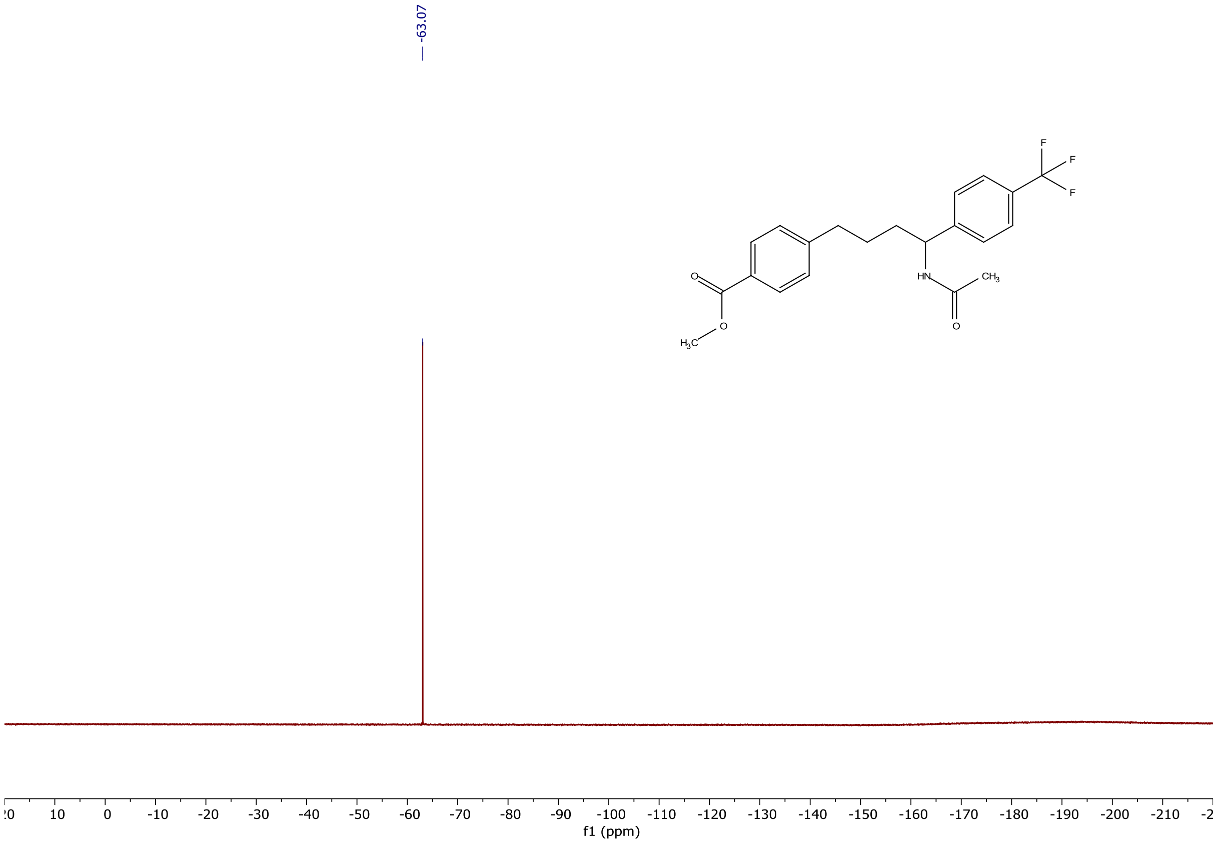

^{1}H NMR (400 MHz, $CDCl_3$) of **12**

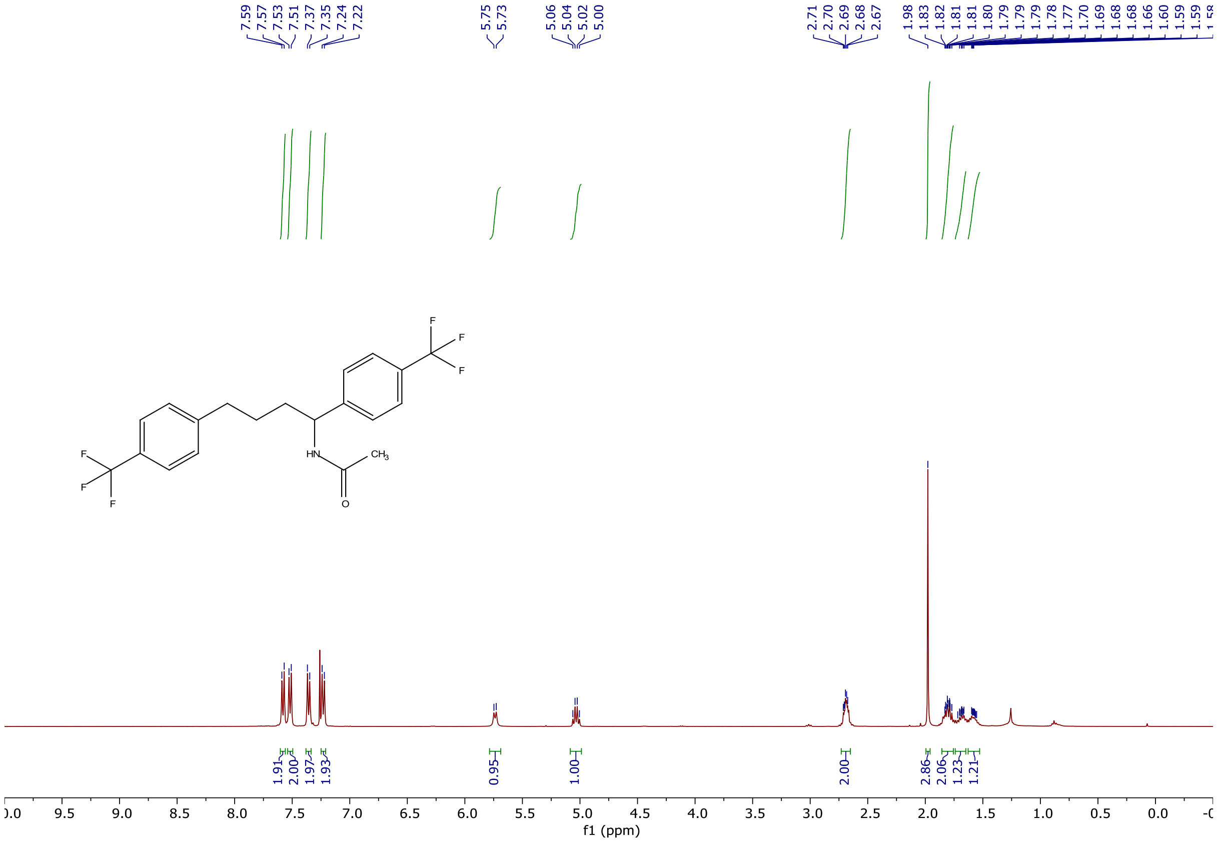

^{13}C NMR (151 MHz, $CDCl_3$) of **12**

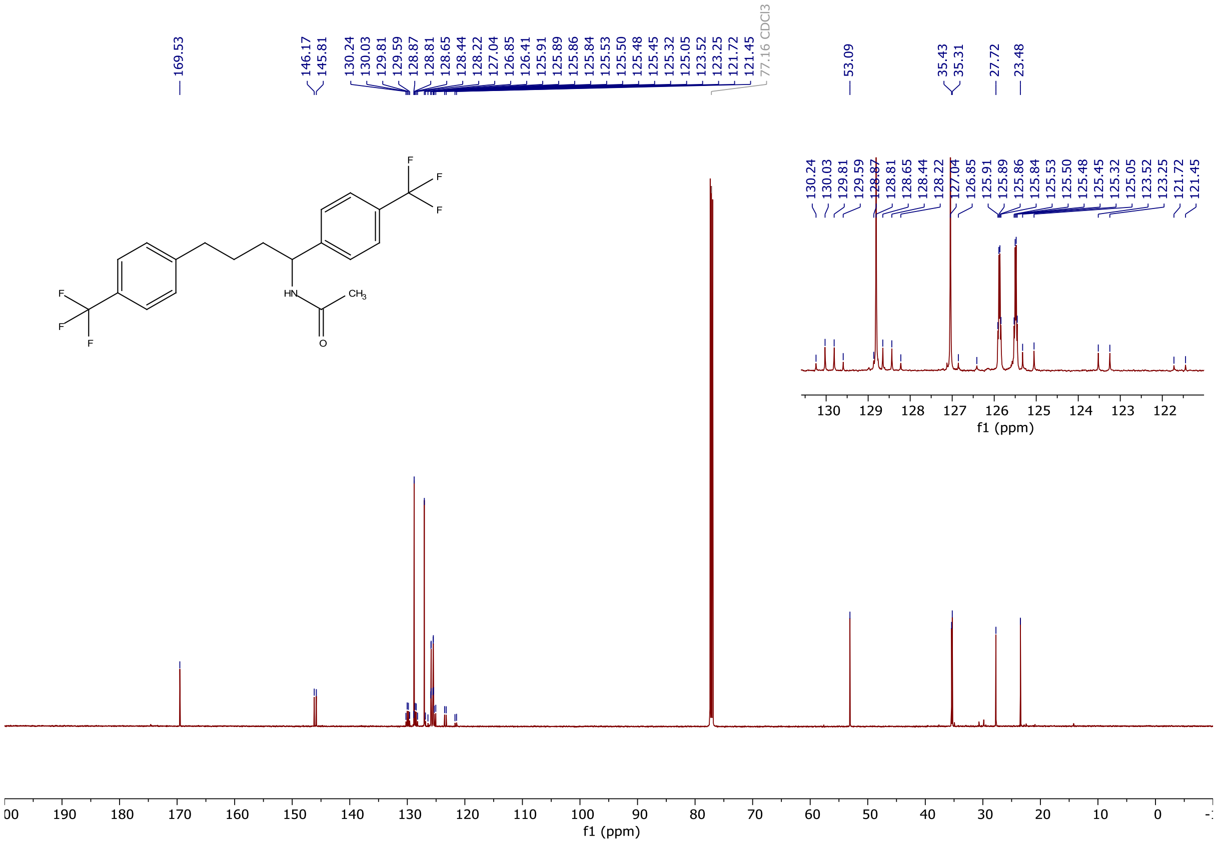

^{19}F NMR (376 MHz, $CDCl_3$) of **12**

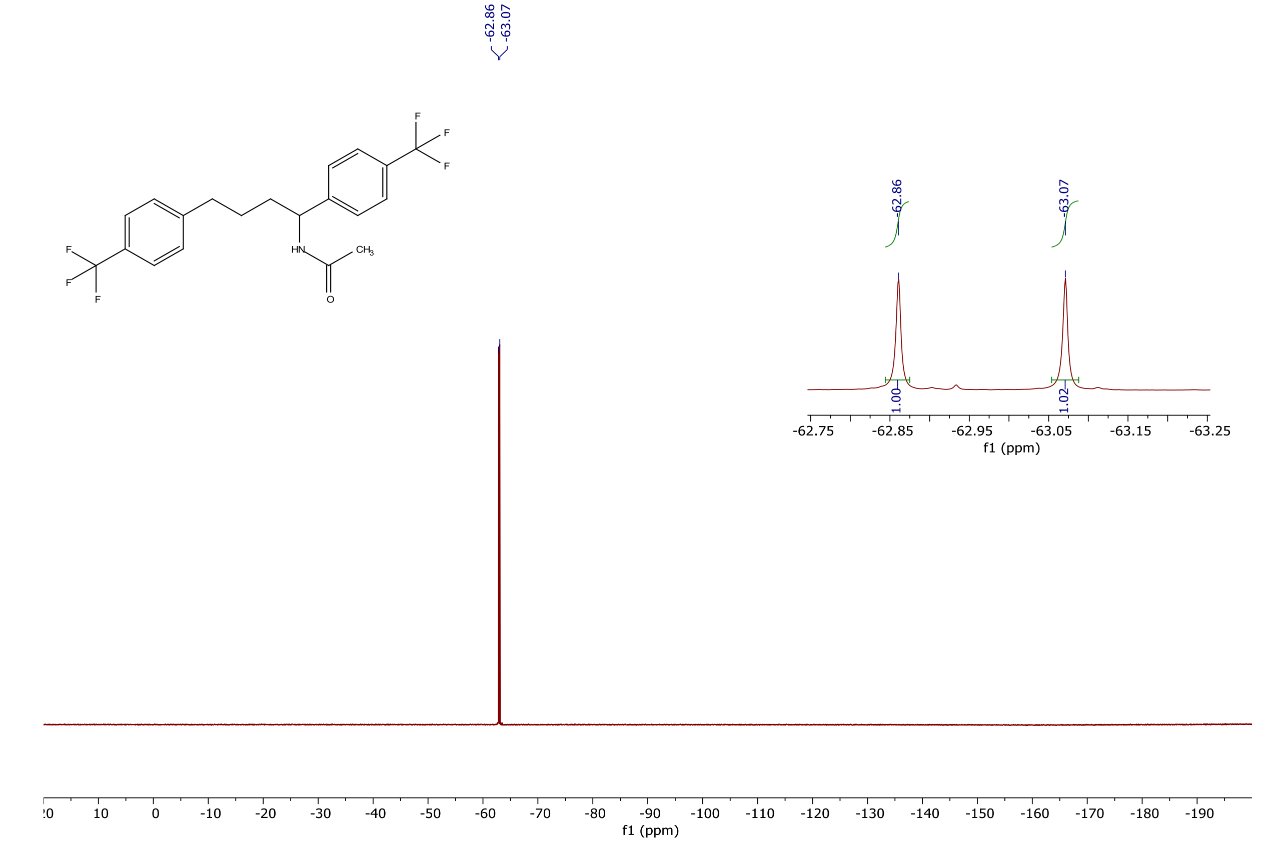

^{1}H NMR (400 MHz, $CDCl_3$) of **13**

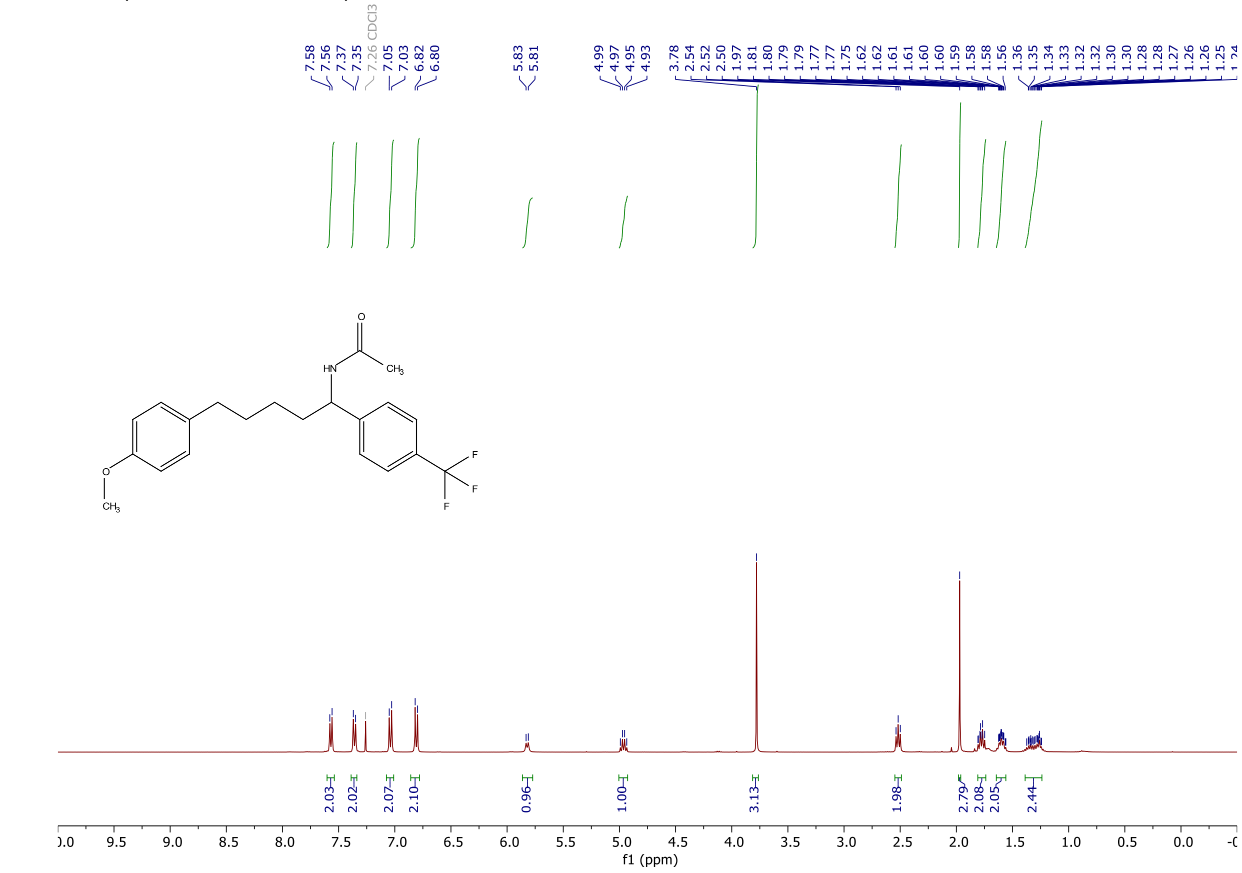

^{13}C NMR (151 MHz, $CDCl_3$) of **13**

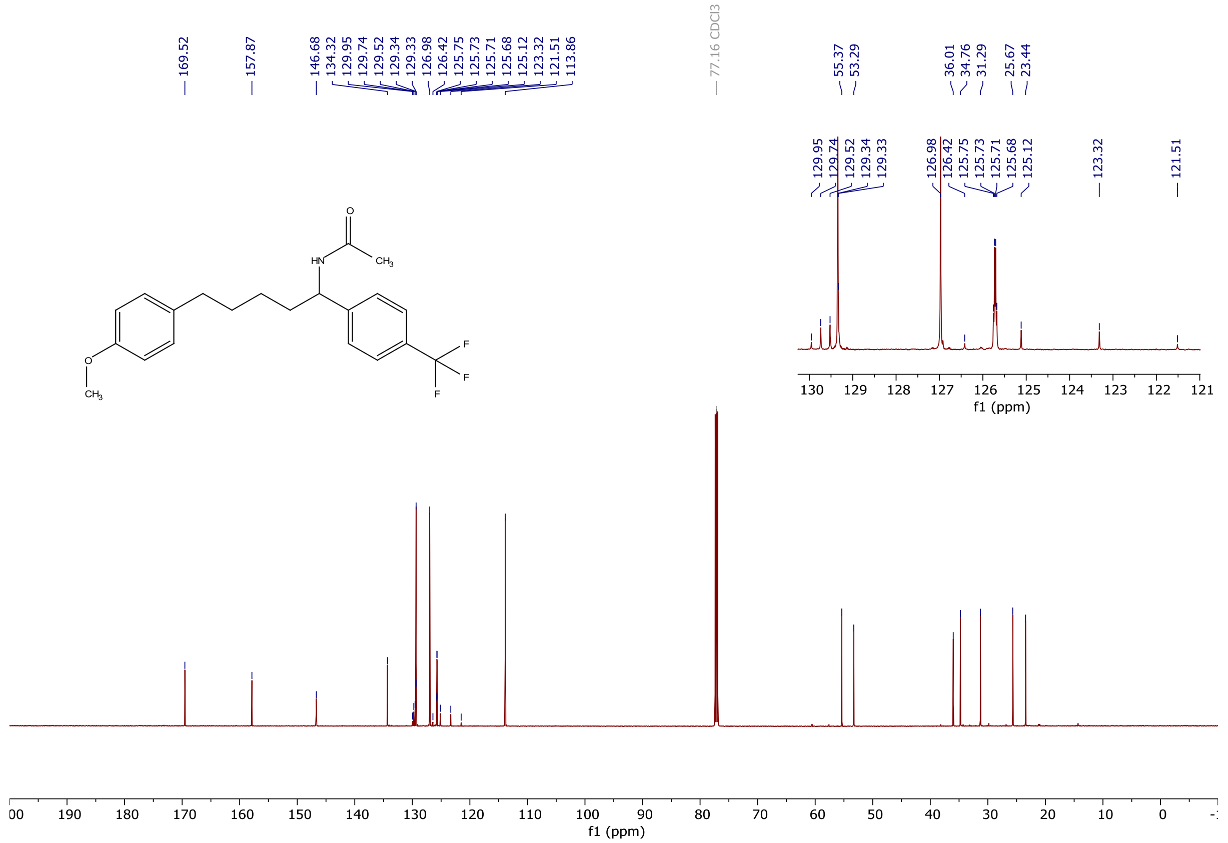

^{19}F NMR (376 MHz, $CDCl_3$) of **13**

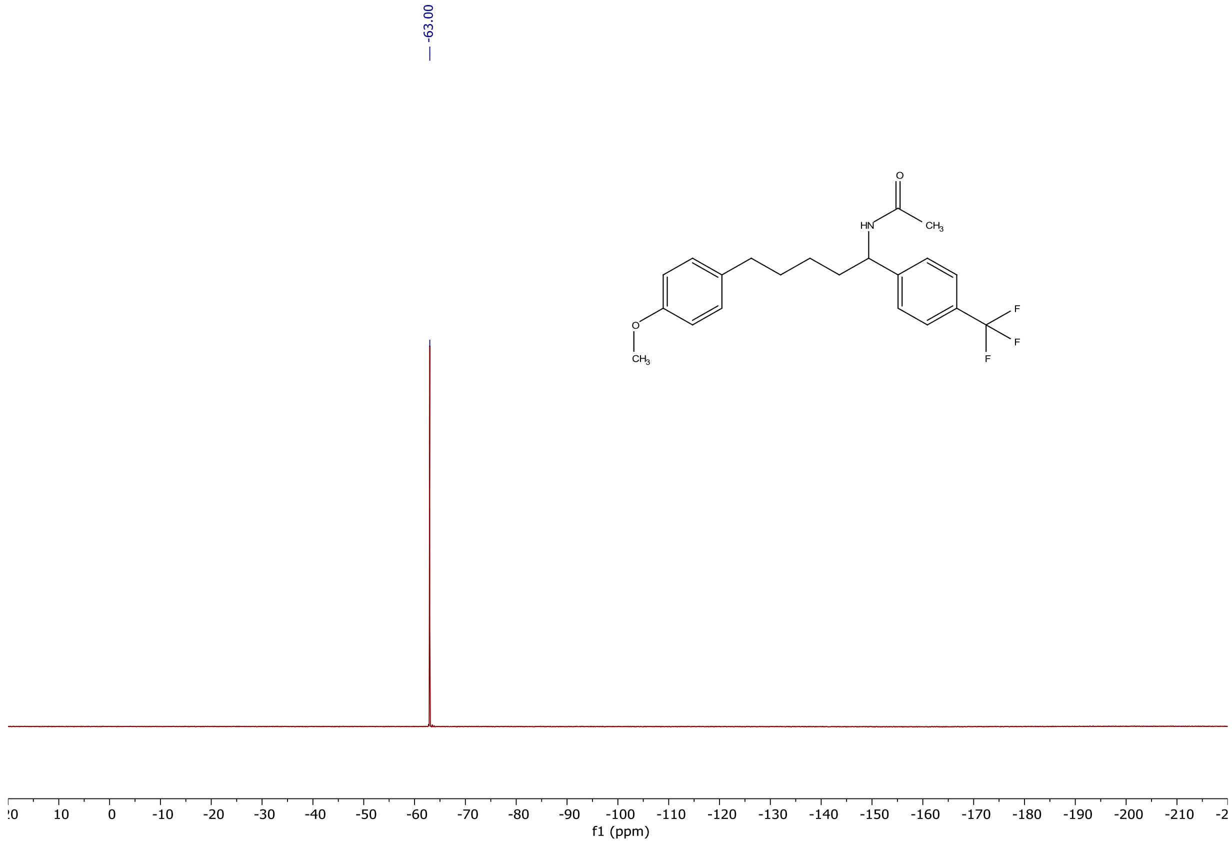

^{1}H NMR (400 MHz, $CDCl_3$) of **14**

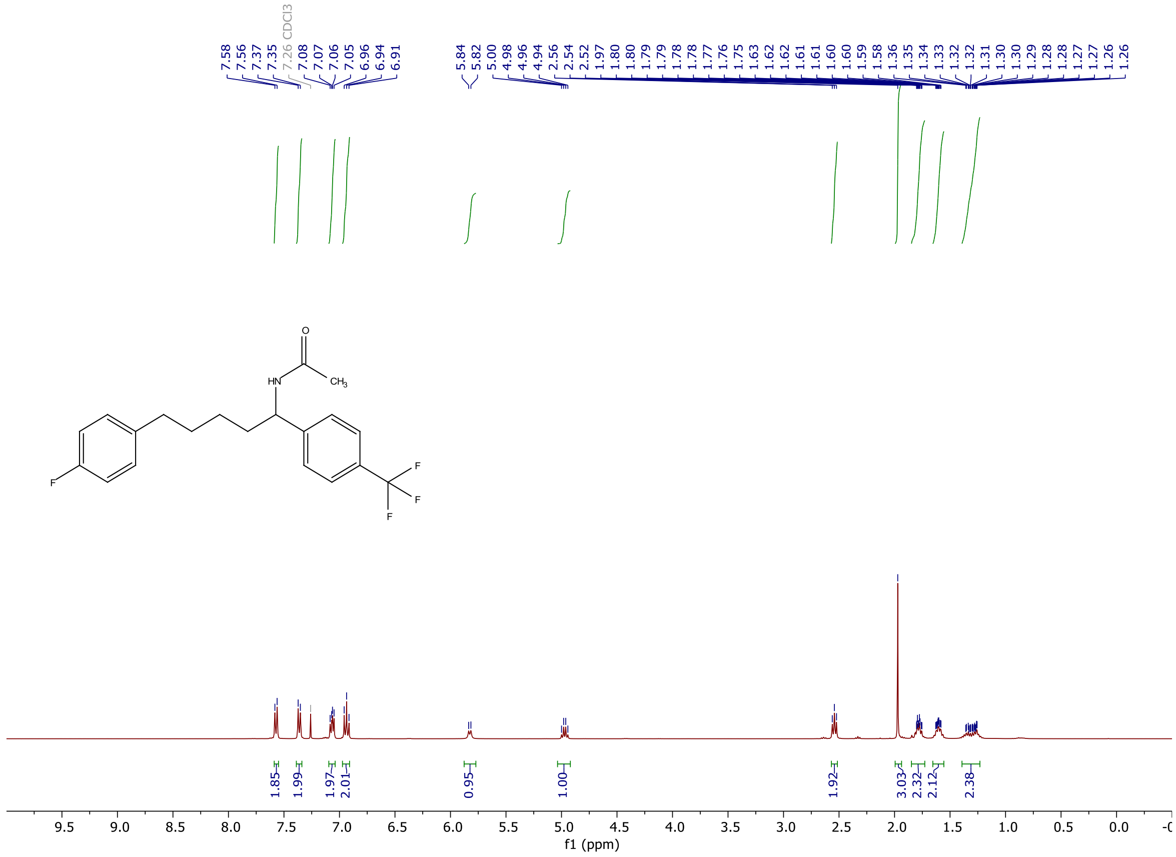

^{13}C NMR (151 MHz, $CDCl_3$) of **14**

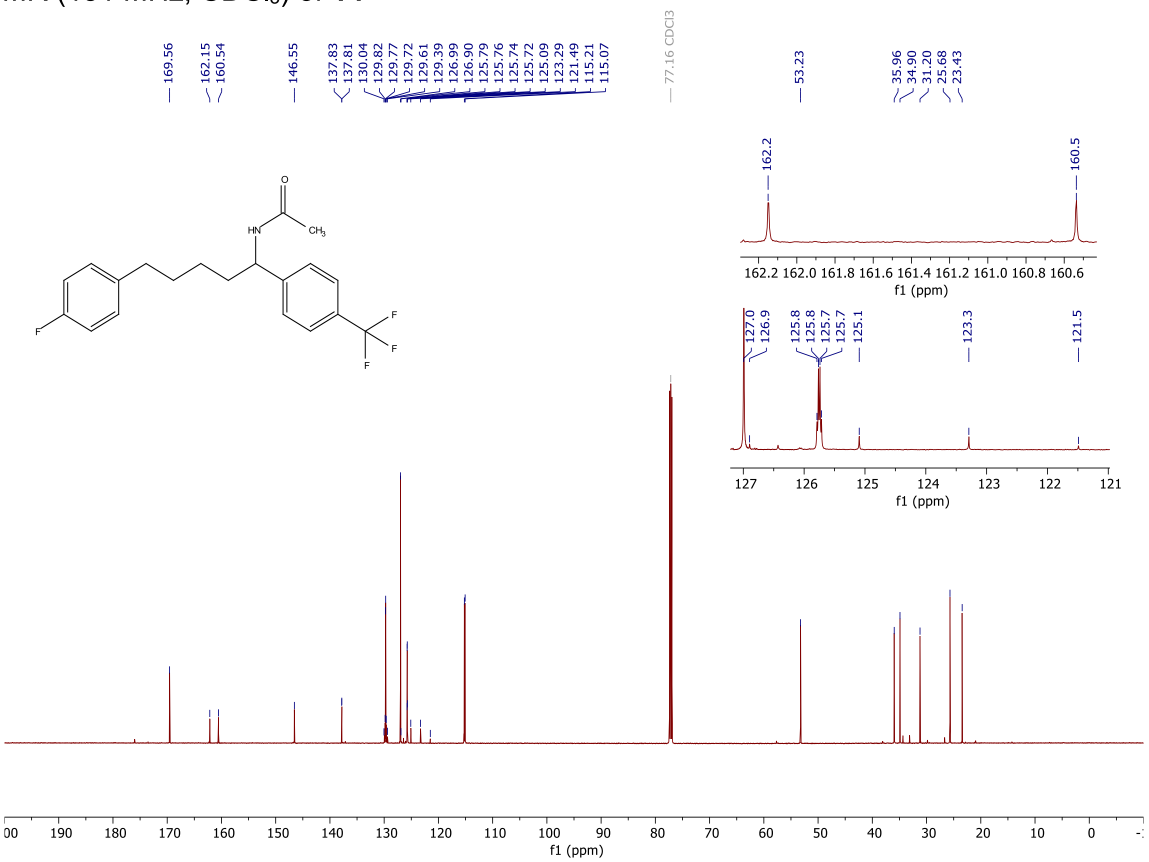

^{19}F NMR (376 MHz, $CDCl_3$) of **14**

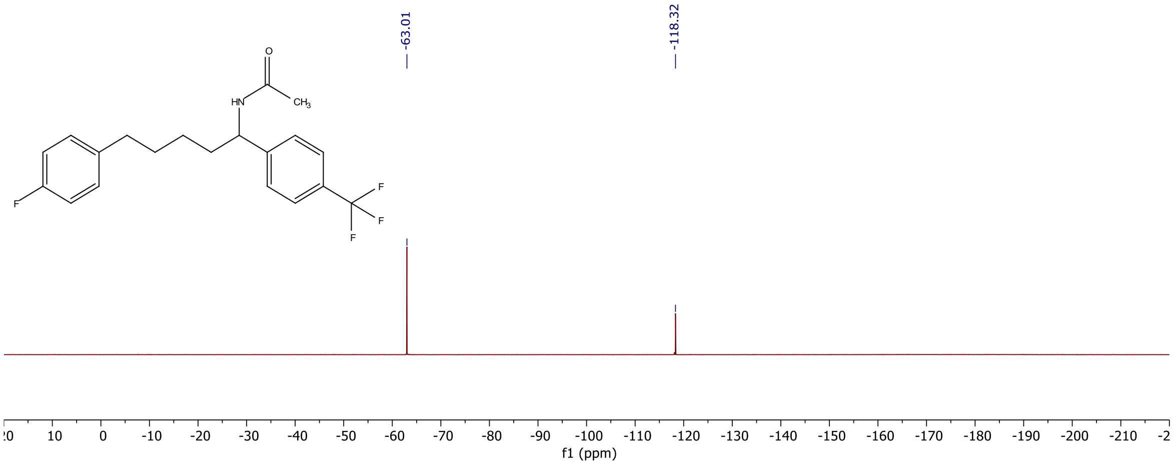

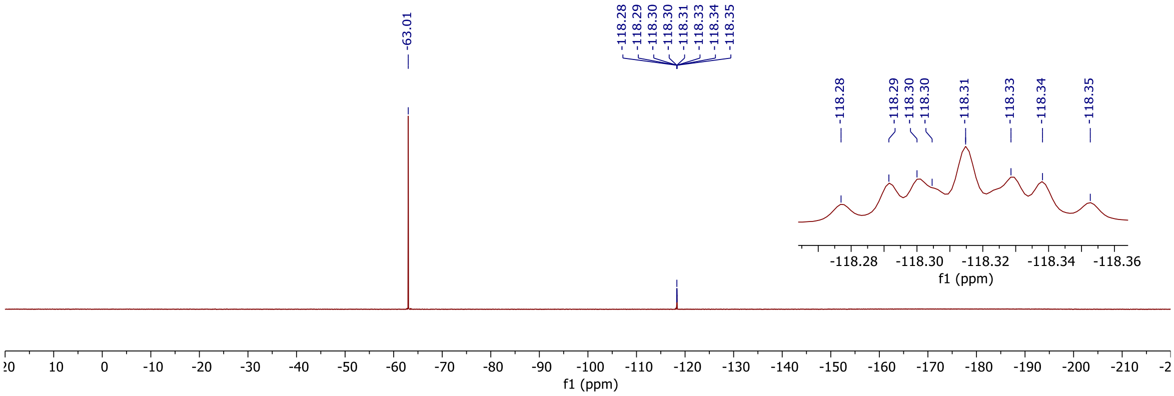

^{1}H NMR (400 MHz, $CDCl_3$) of **15**

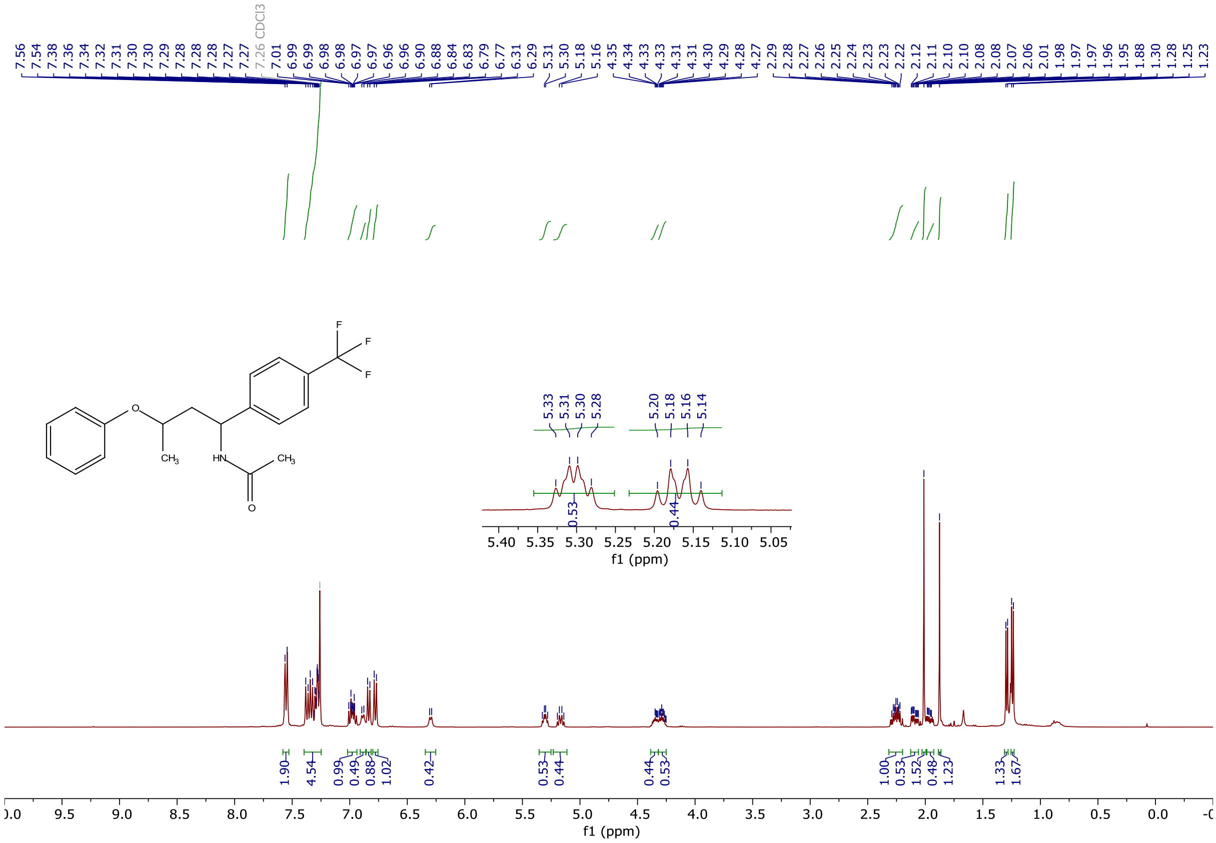

^{13}C NMR (151 MHz, $CDCl_3$) of **15**

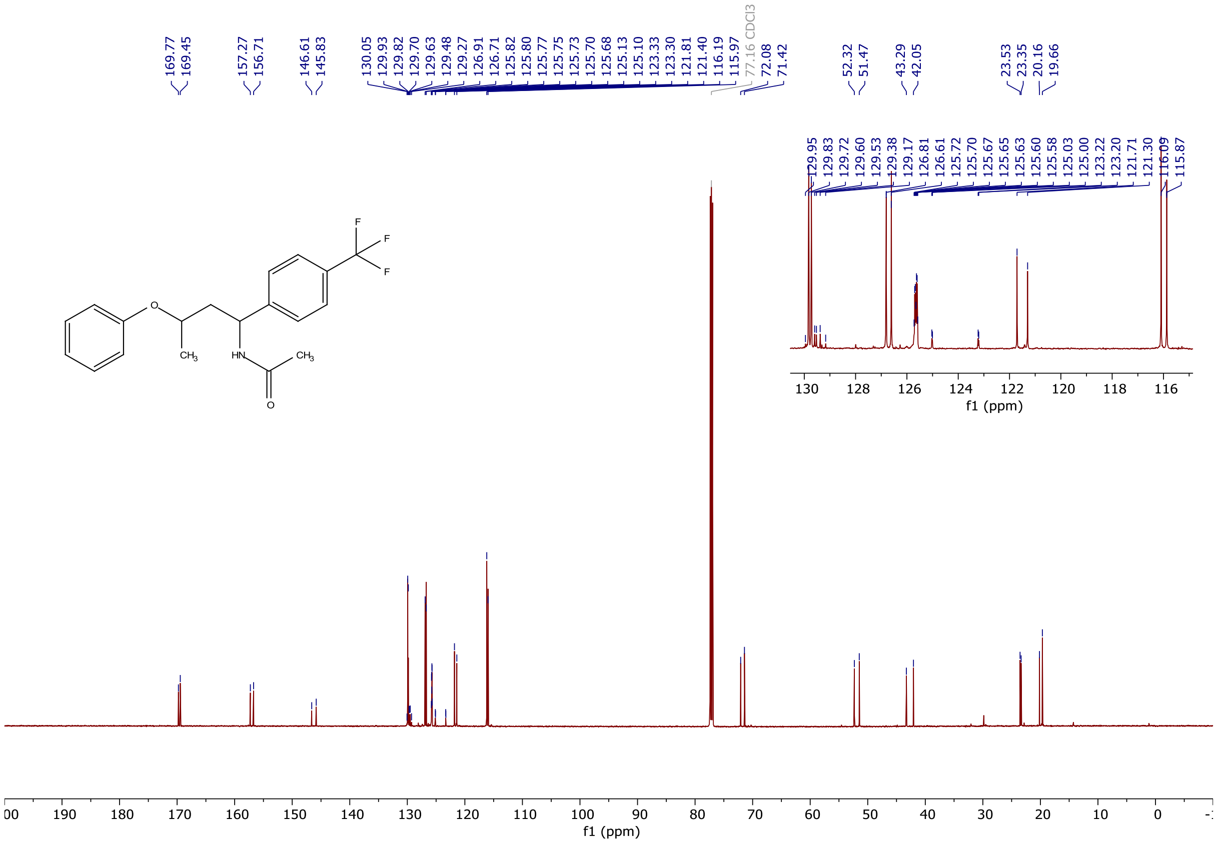

^{19}F NMR (376 MHz, $CDCl_3$) of **15**

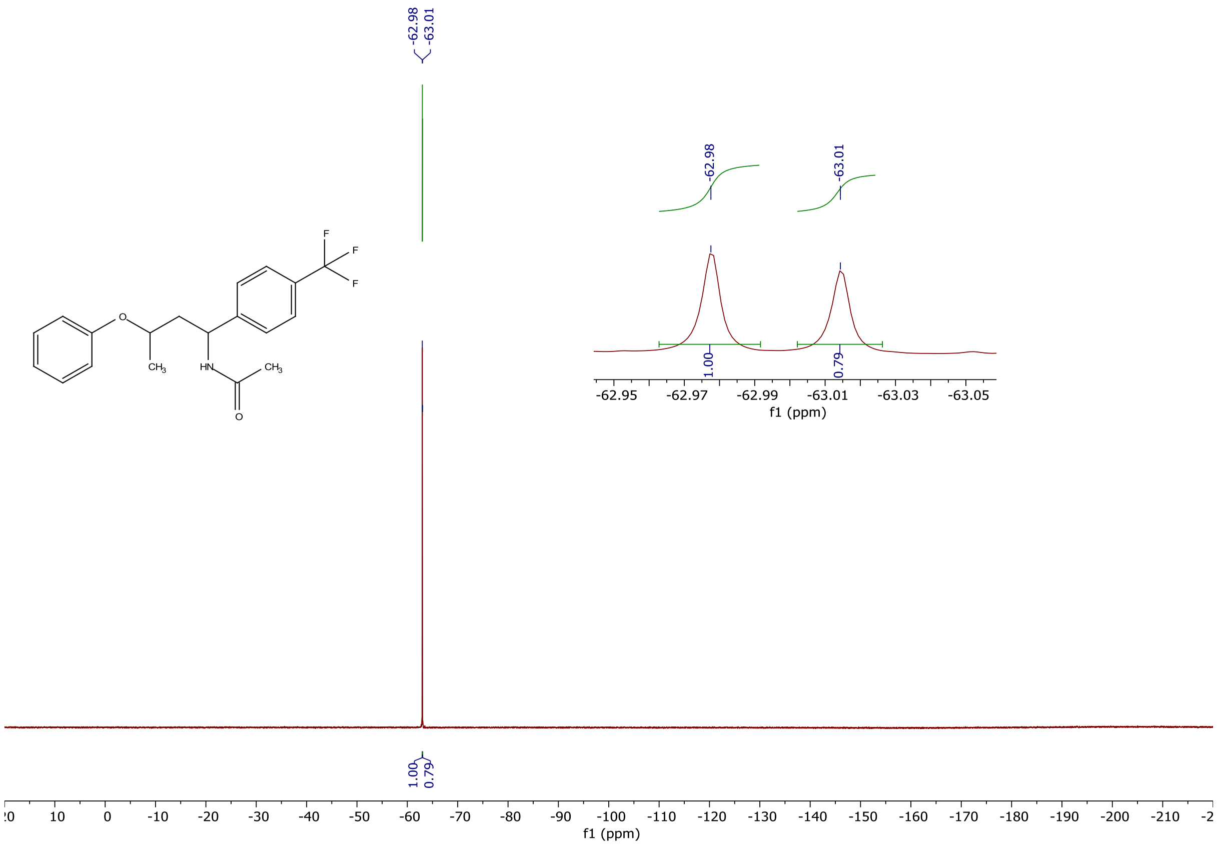

^{1}H NMR (400 MHz, $CDCl_3$) of **16**

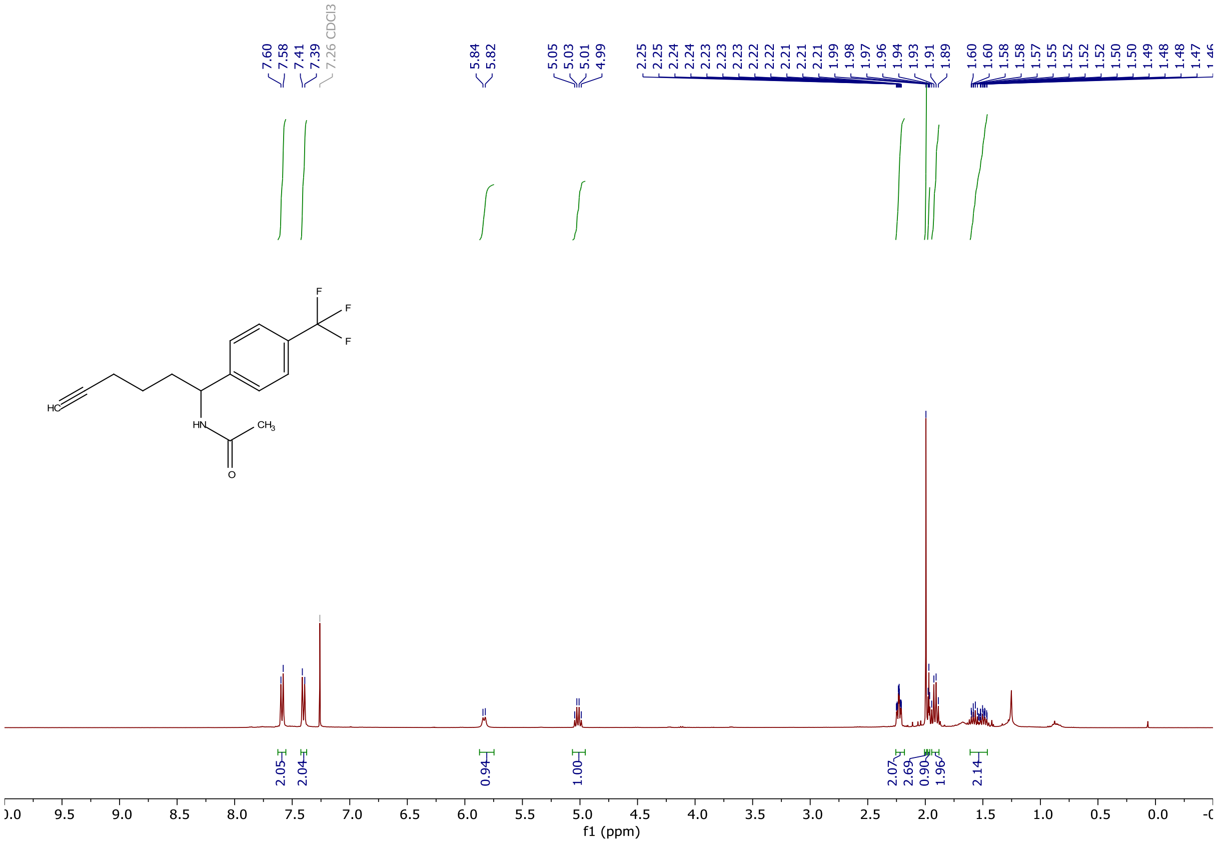

^{13}C NMR (151 MHz, $CDCl_3$) of **16**

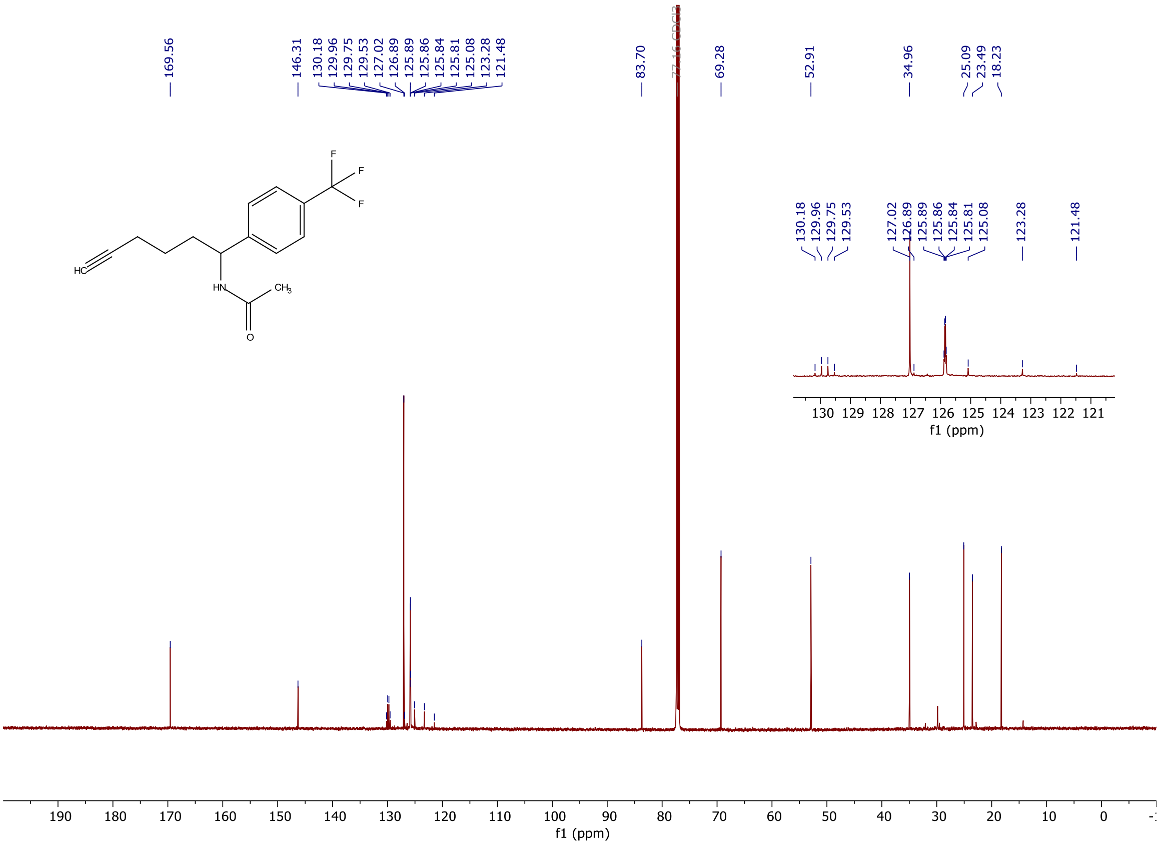

^{19}F NMR (376 MHz, $CDCl_3$) of **16**

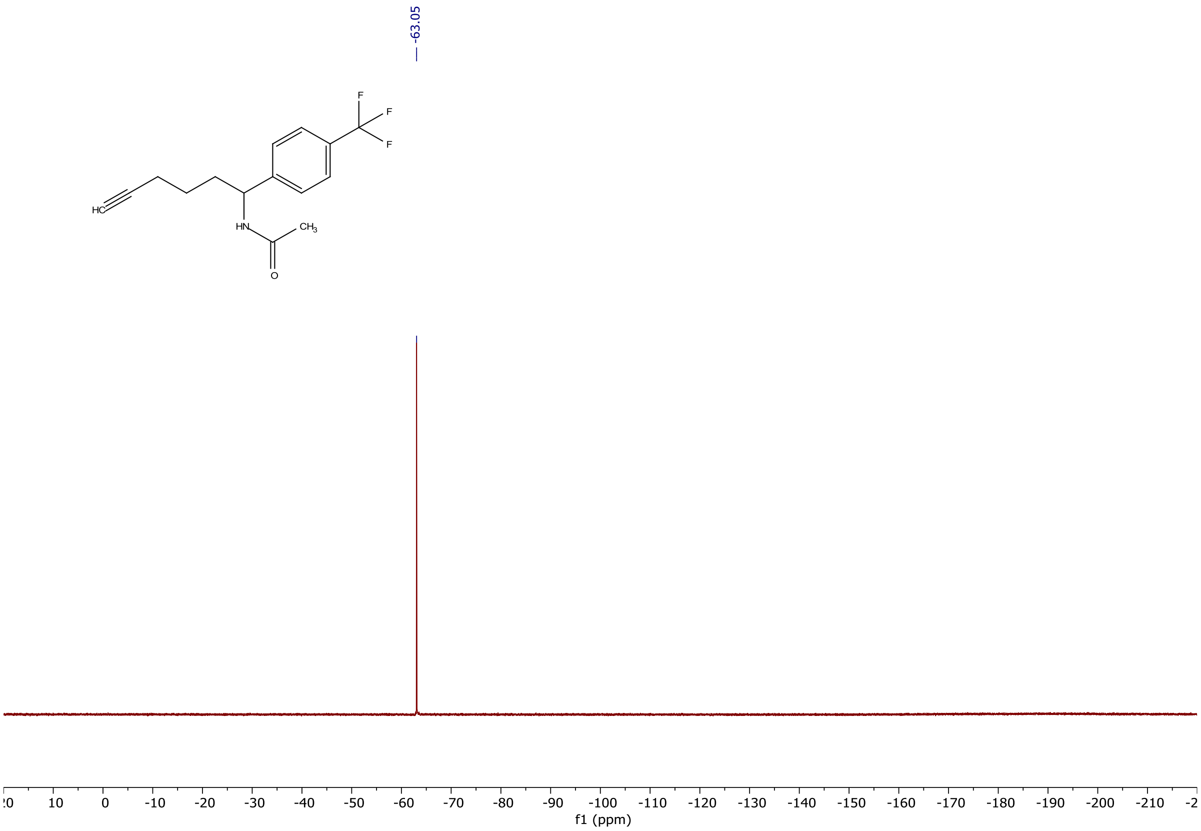

^{1}H NMR (400 MHz, $CDCl_3$) of **17**

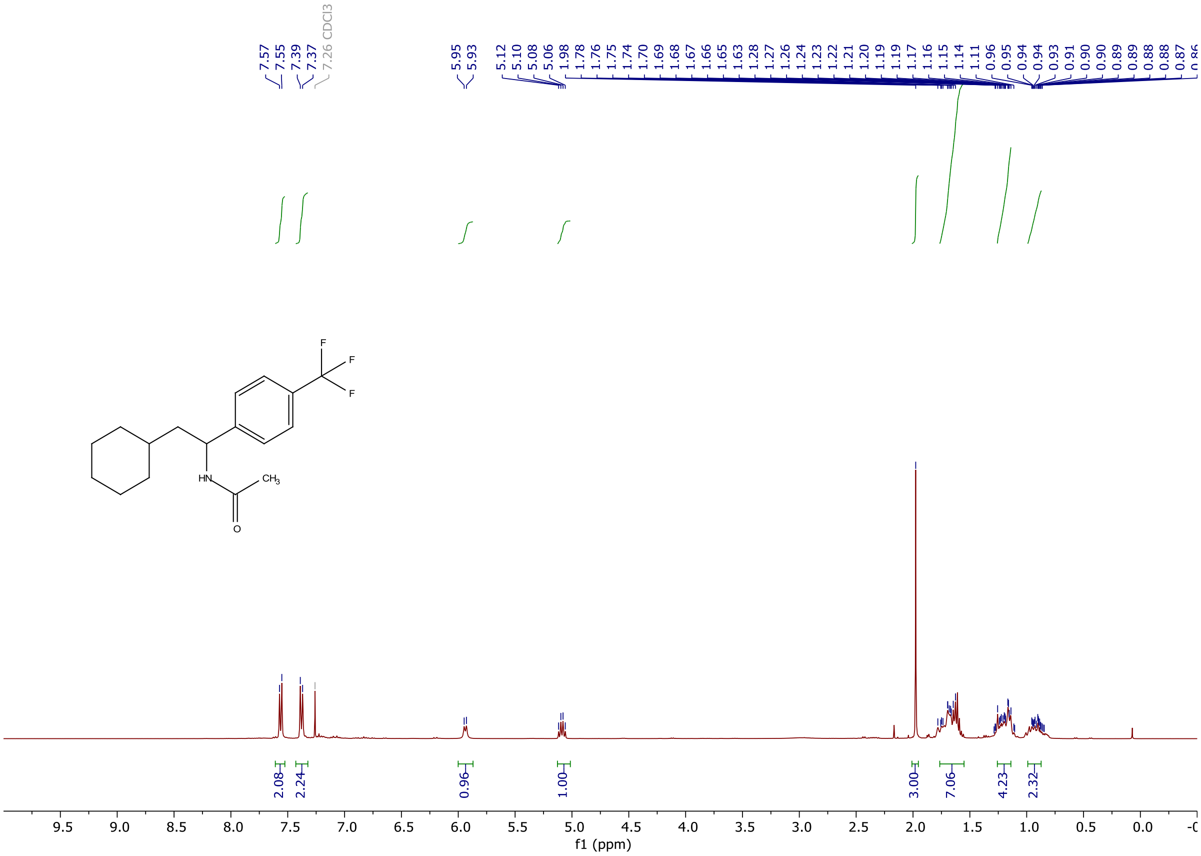

^{13}C NMR (151 MHz, $CDCl_3$) of **17**

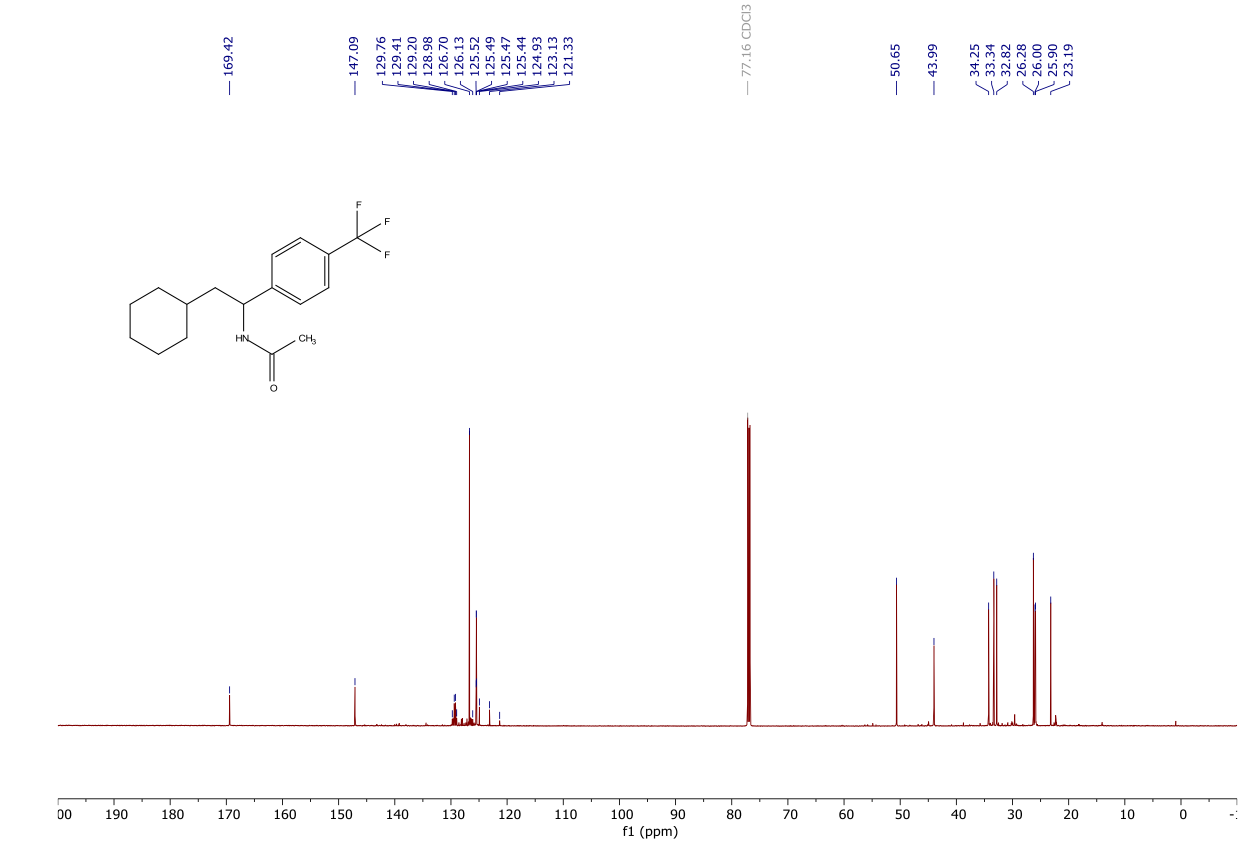

^{19}F NMR (376 MHz, $CDCl_3$) of **17**

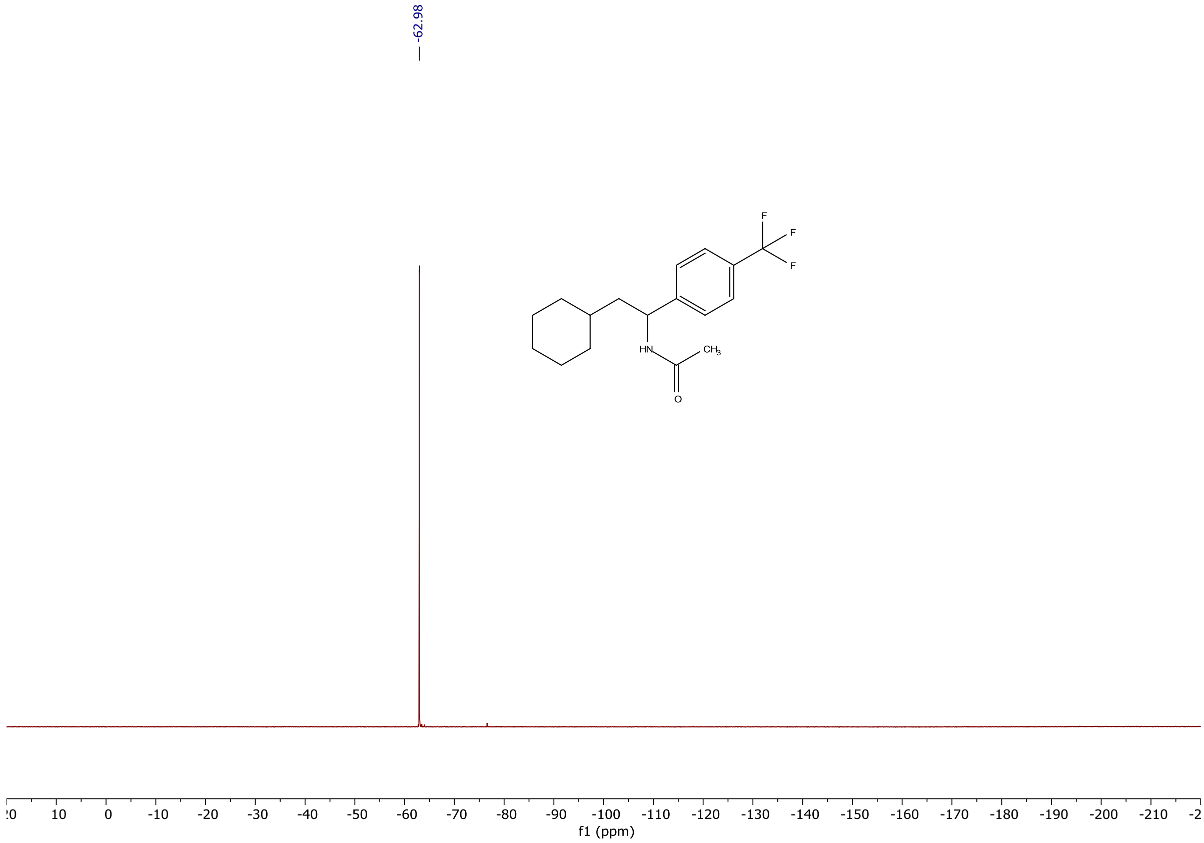

^{1}H NMR (400 MHz, $CDCl_3$) of **18**

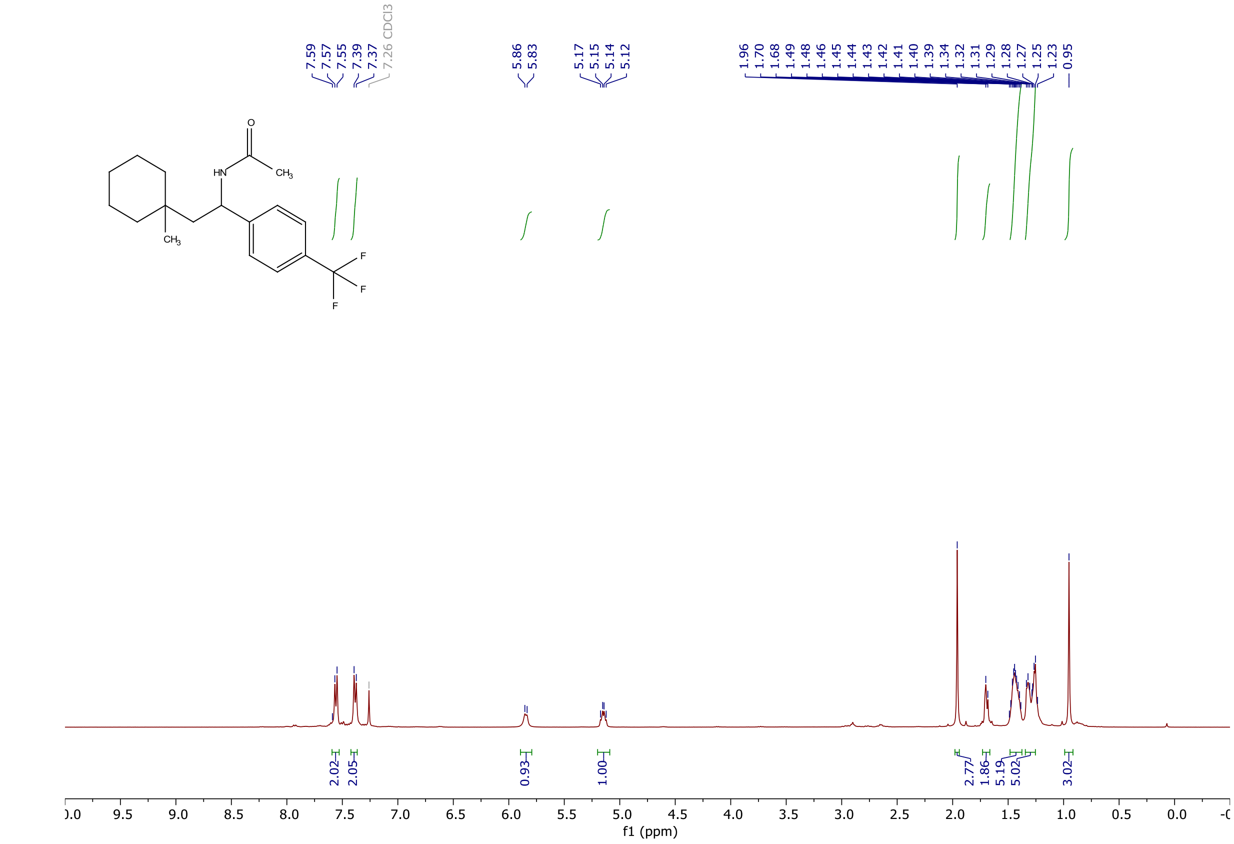

^{13}C NMR (151 MHz, $CDCl_3$) of **18**

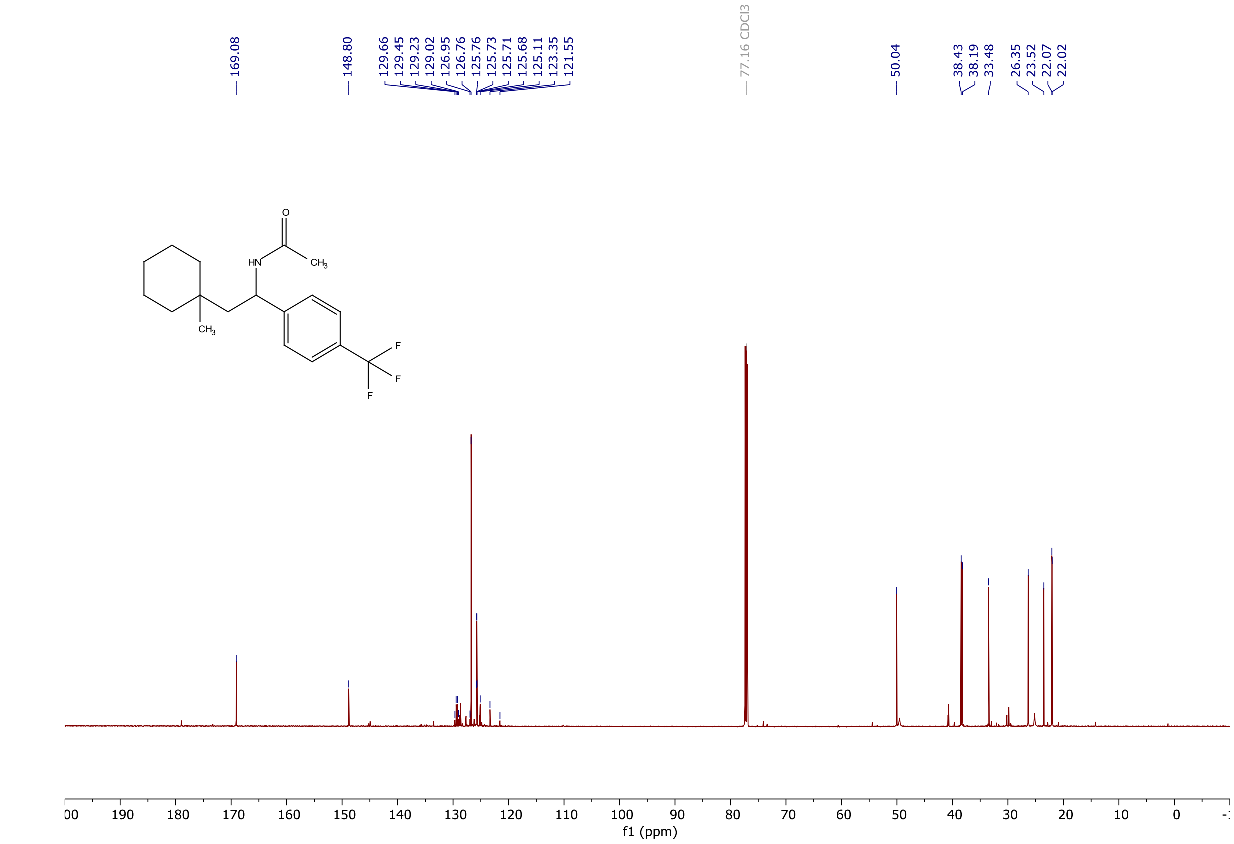

^{19}F NMR (376 MHz, $CDCl_3$) of **18**

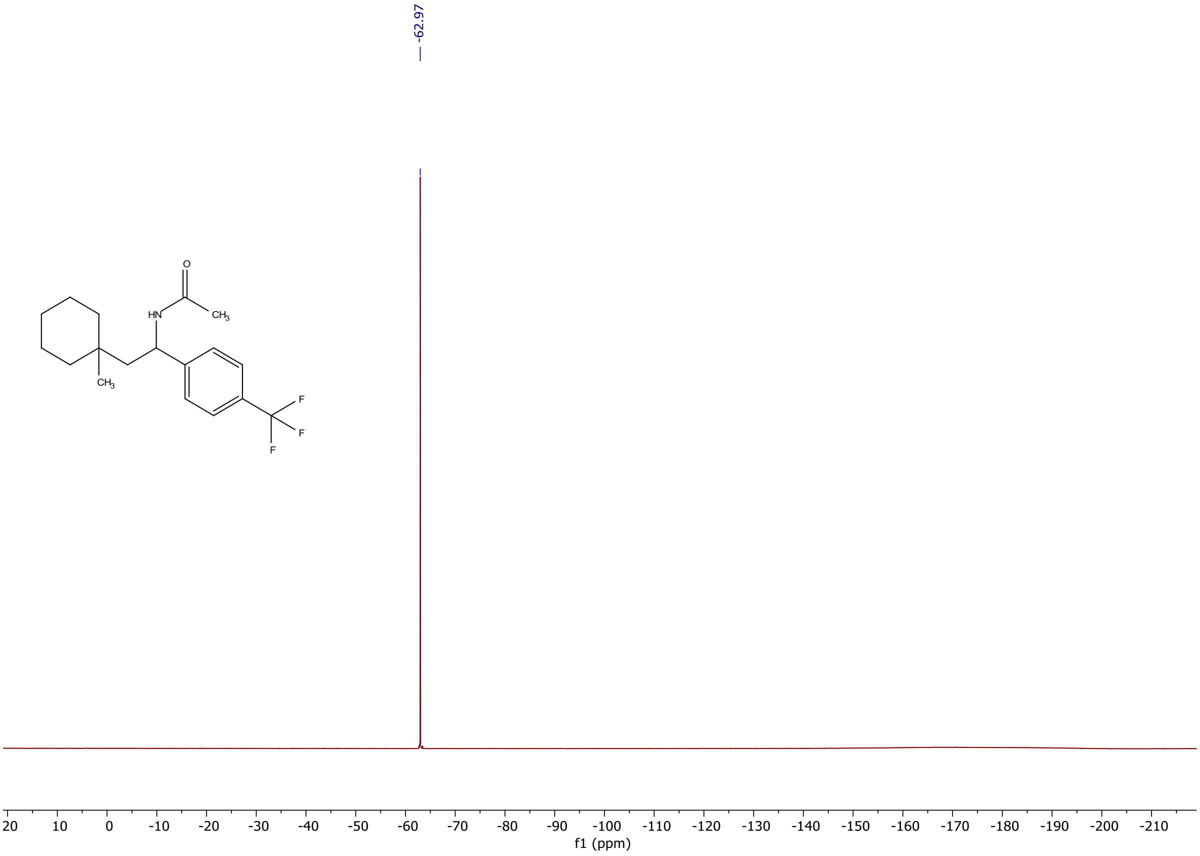

^{1}H NMR (400 MHz, $CDCl_3$) of **19**

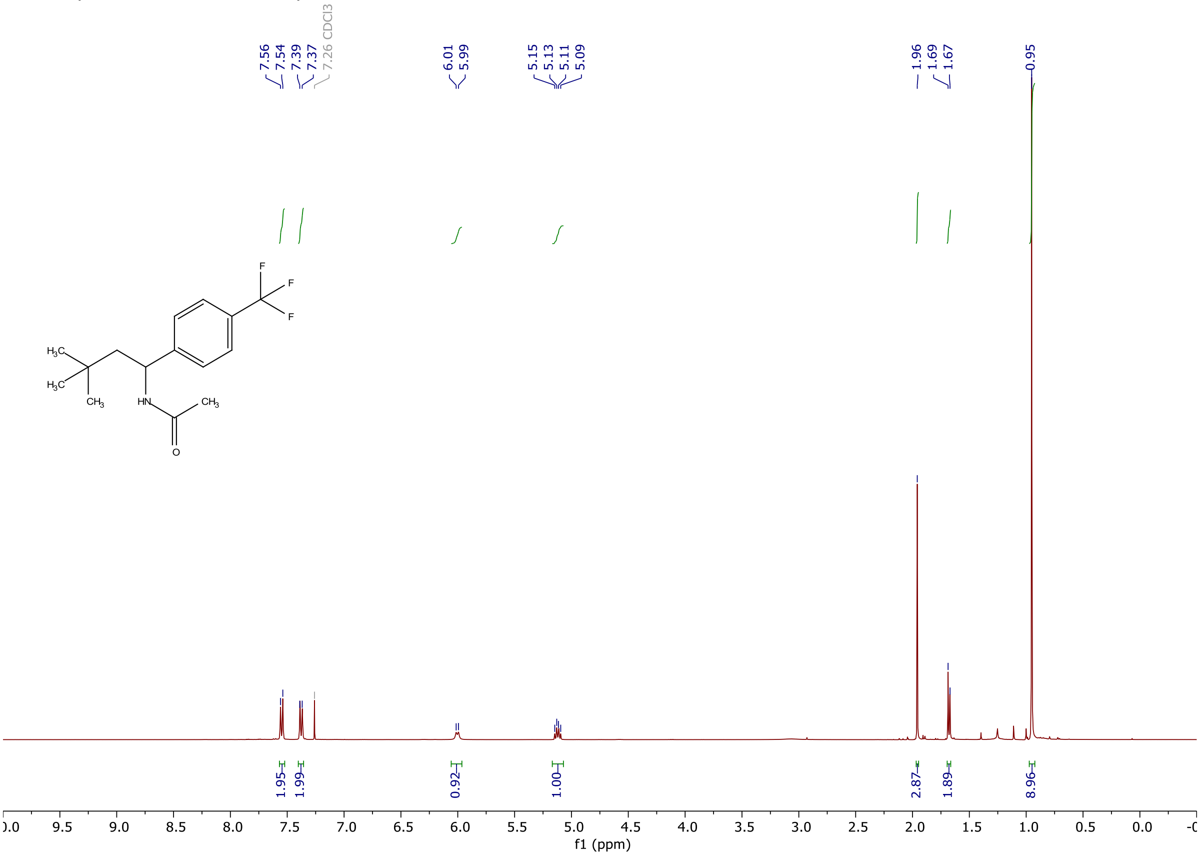

^{13}C NMR (151 MHz, $CDCl_3$) of **19**

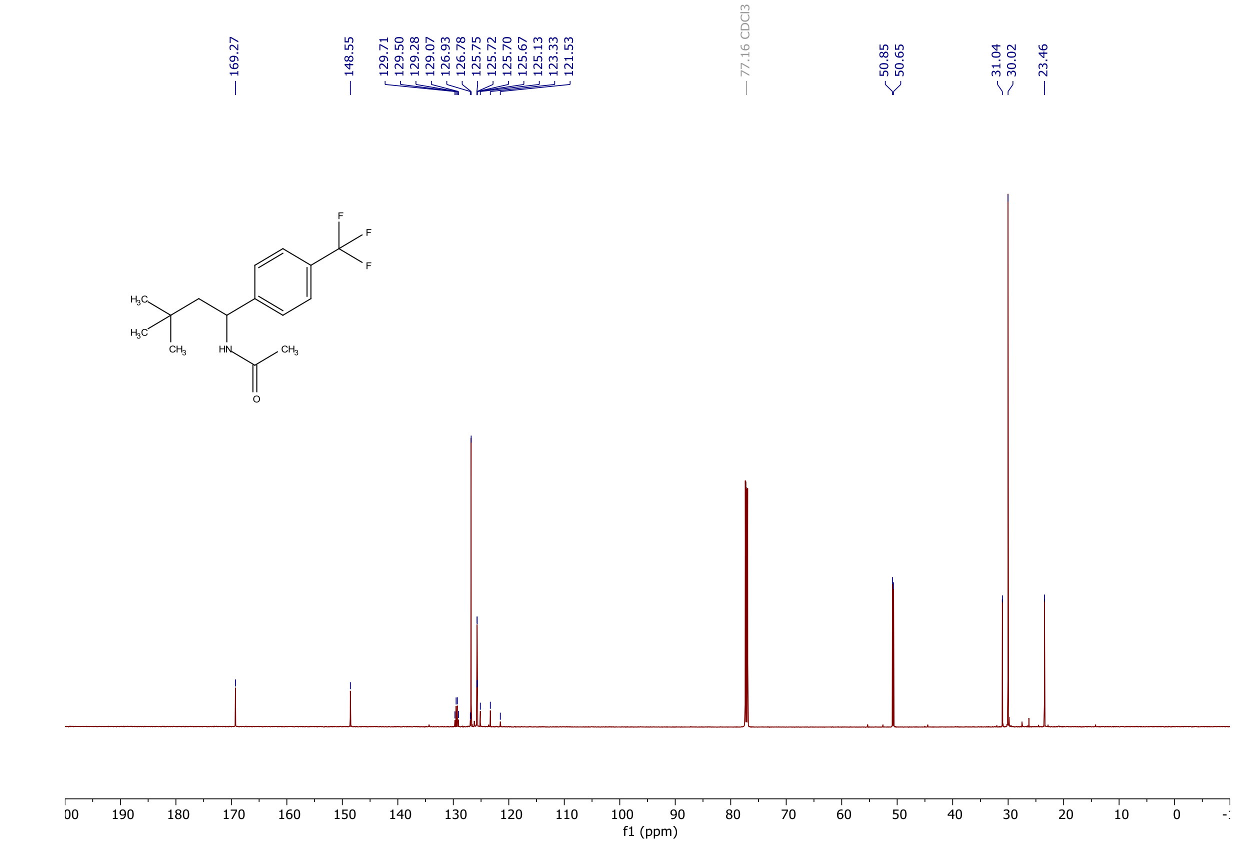

^{19}F NMR (376 MHz, $CDCl_3$) of **19**

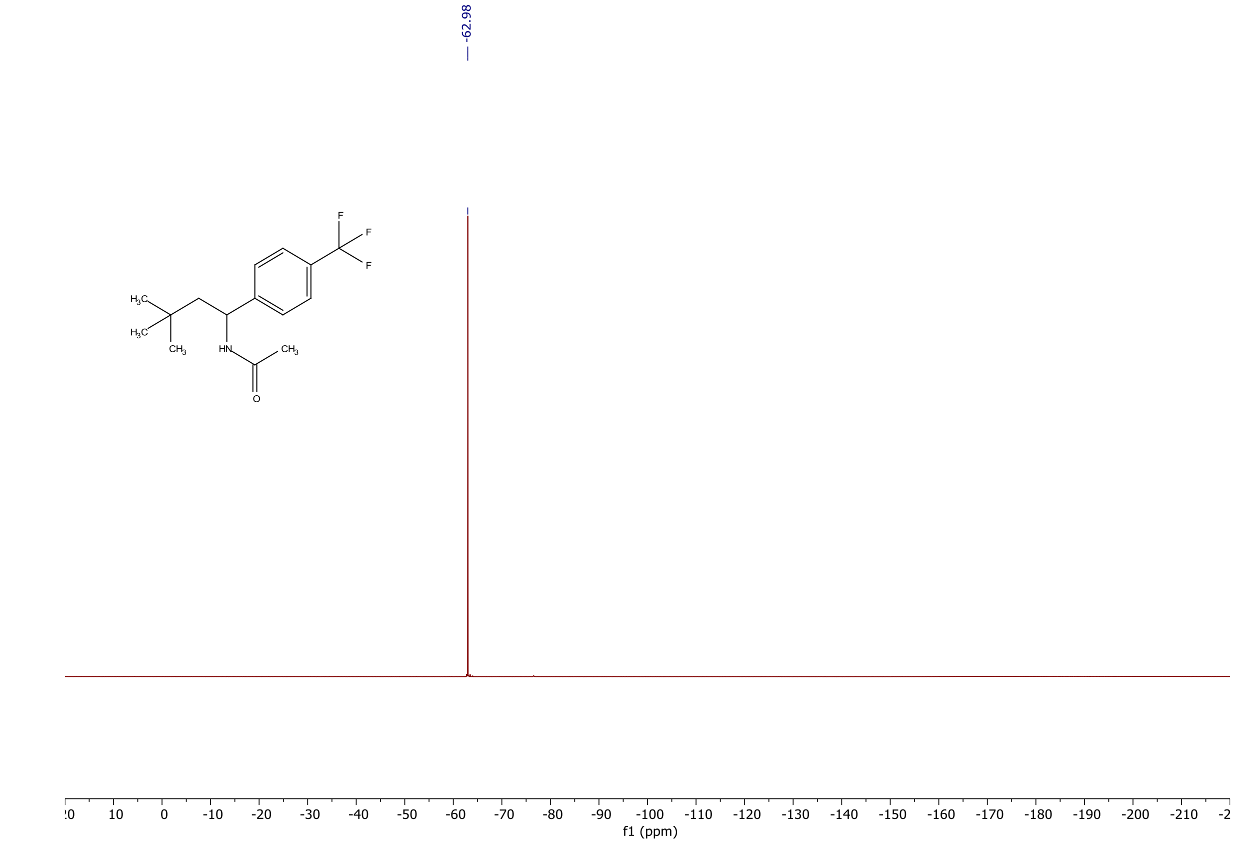

^{1}H NMR (400 MHz, $CDCl_3$) of **20**

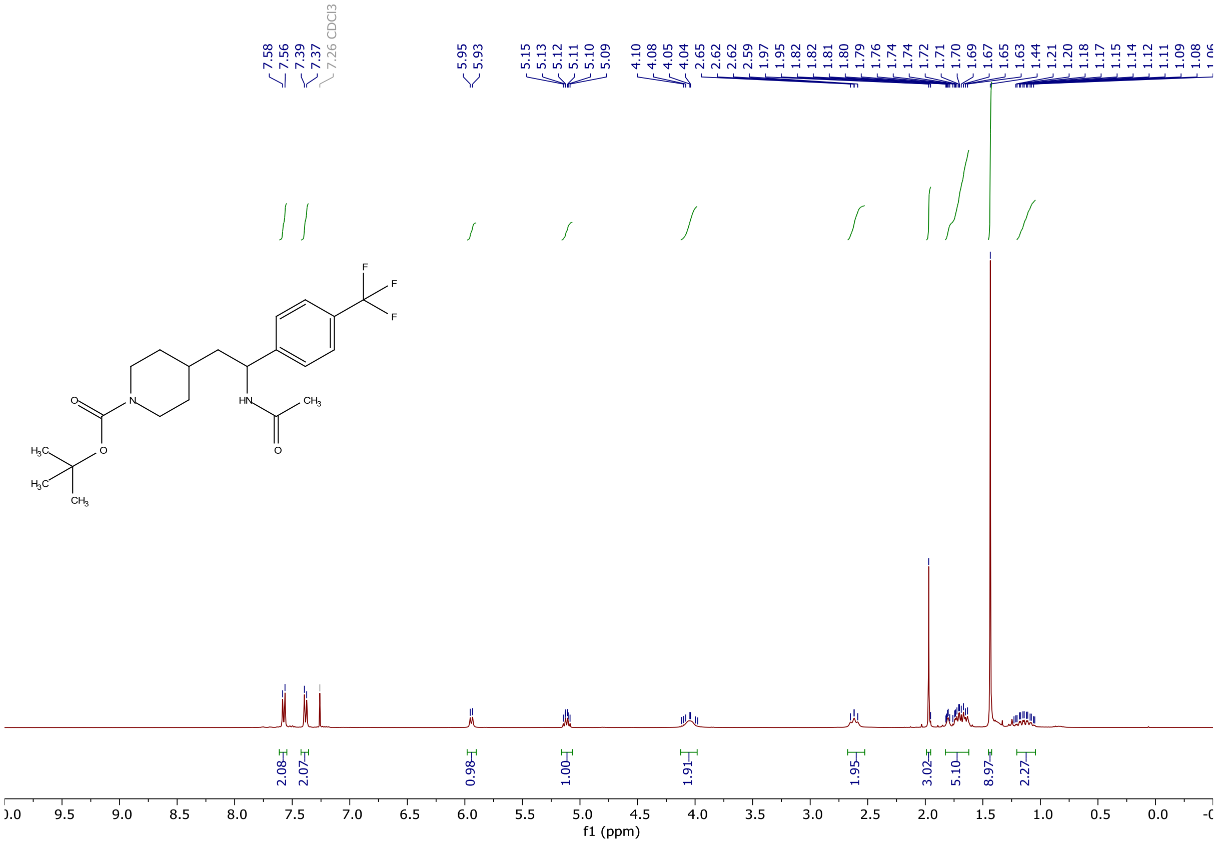

^{13}C NMR (151 MHz, $CDCl_3$) of **20**

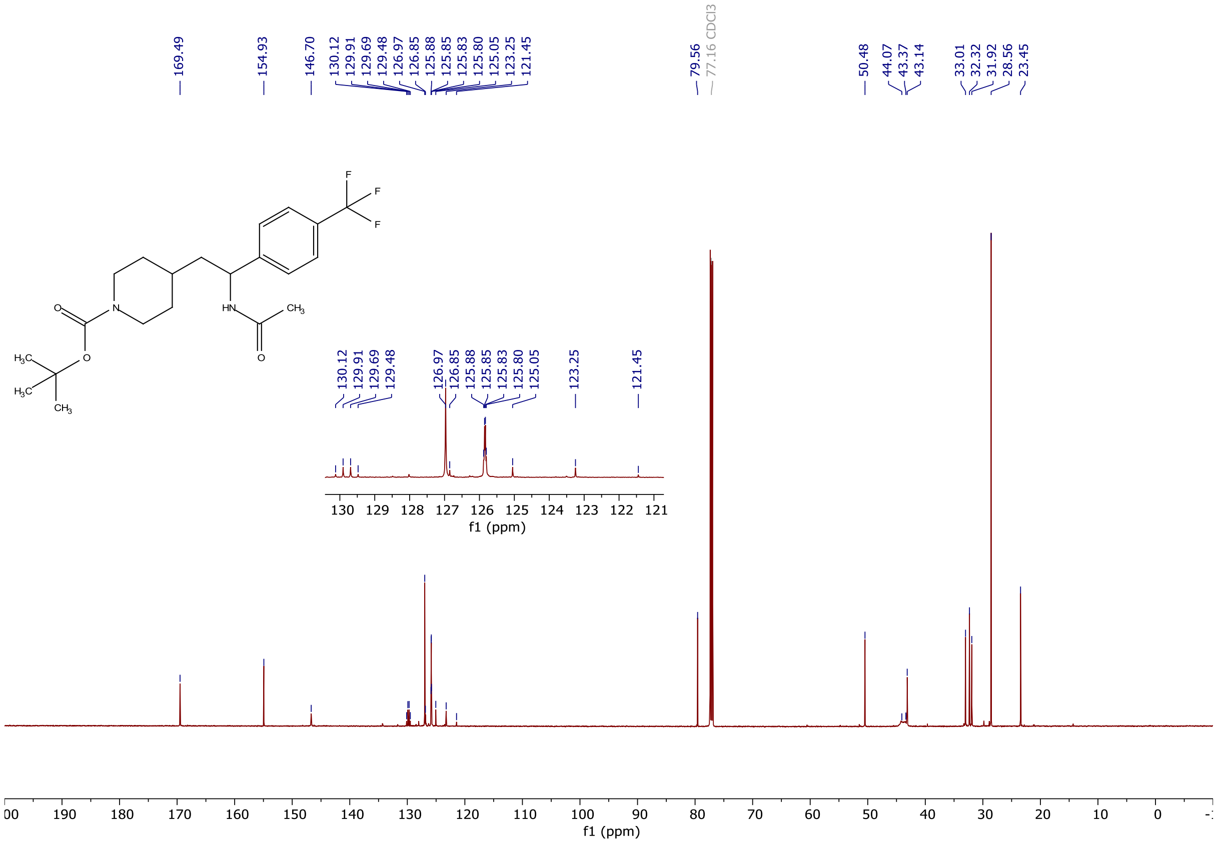

^{19}F NMR (376 MHz, $CDCl_3$) of **20**

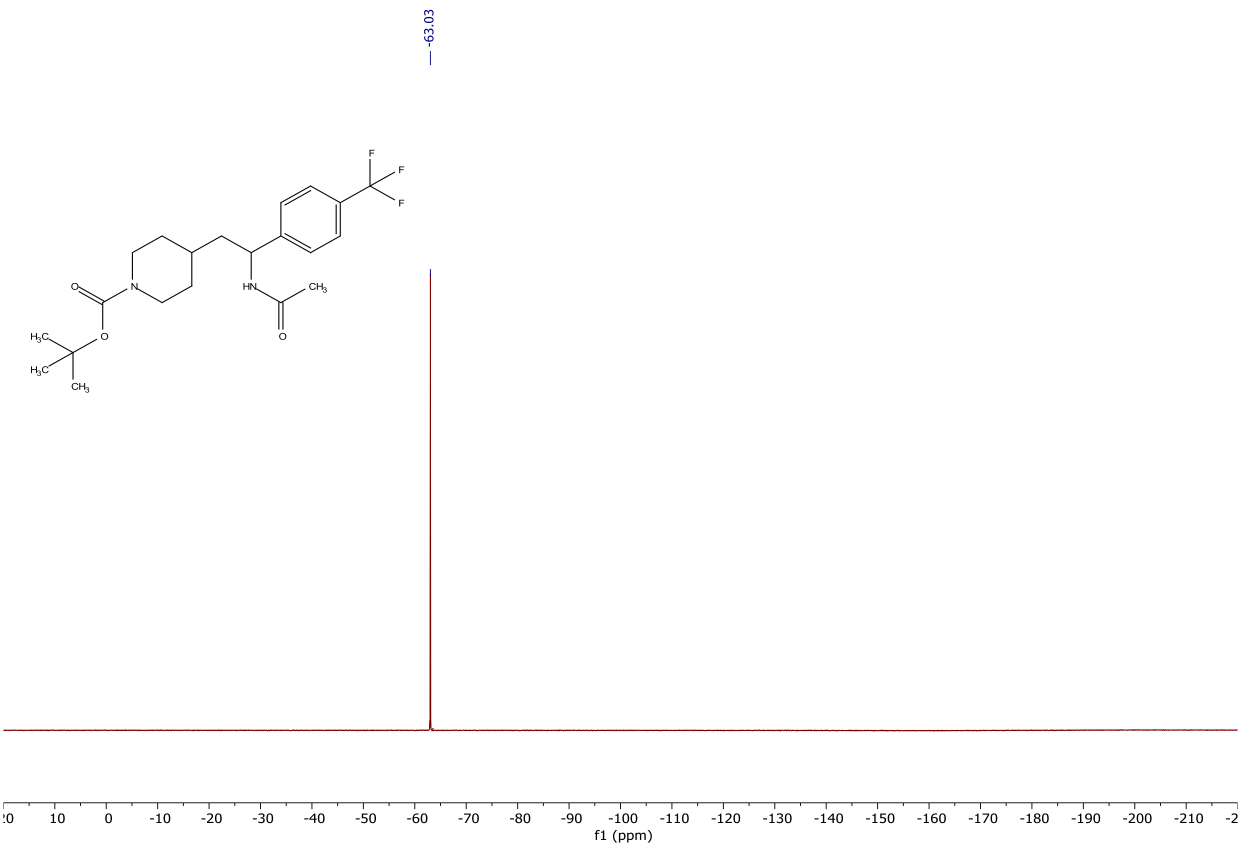

^{1}H NMR (400 MHz, $CDCl_3$) of **21**

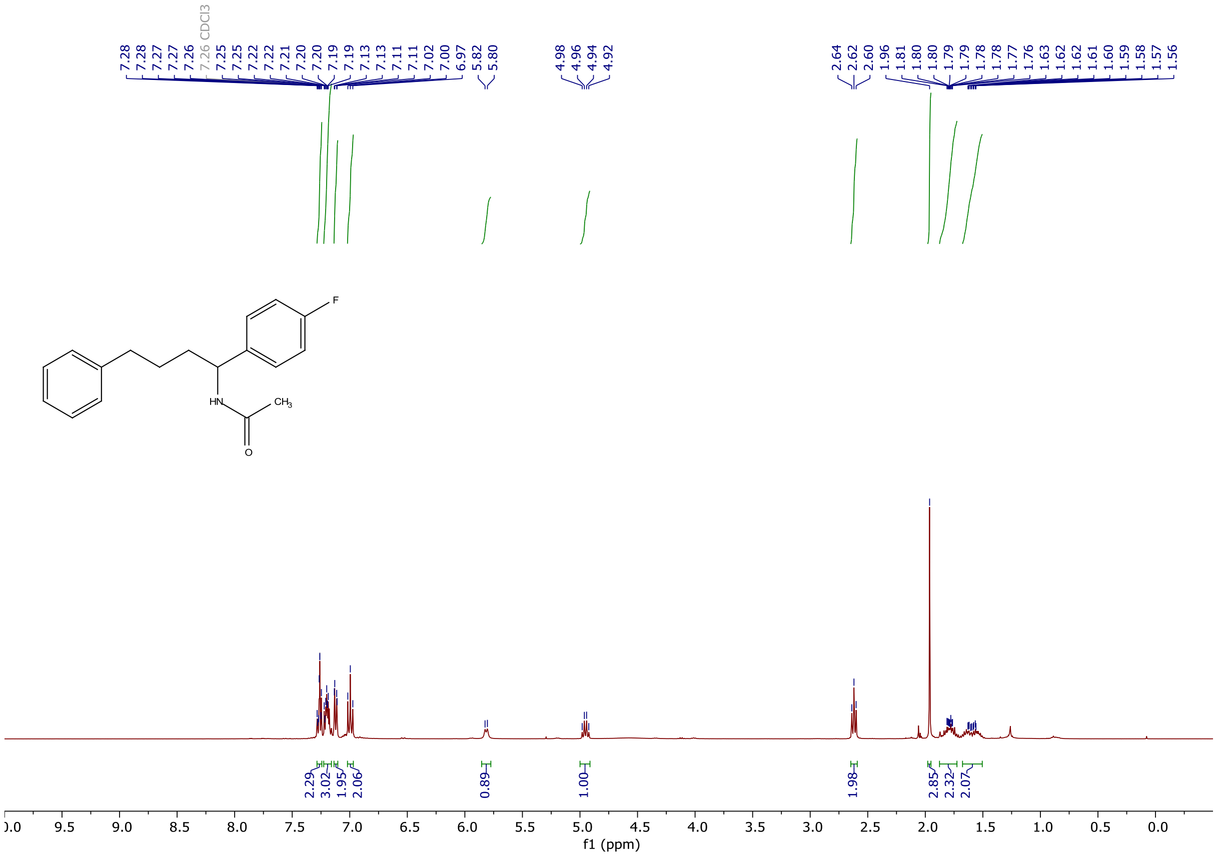

^{13}C NMR (126 MHz, $CDCl_3$) of **21**

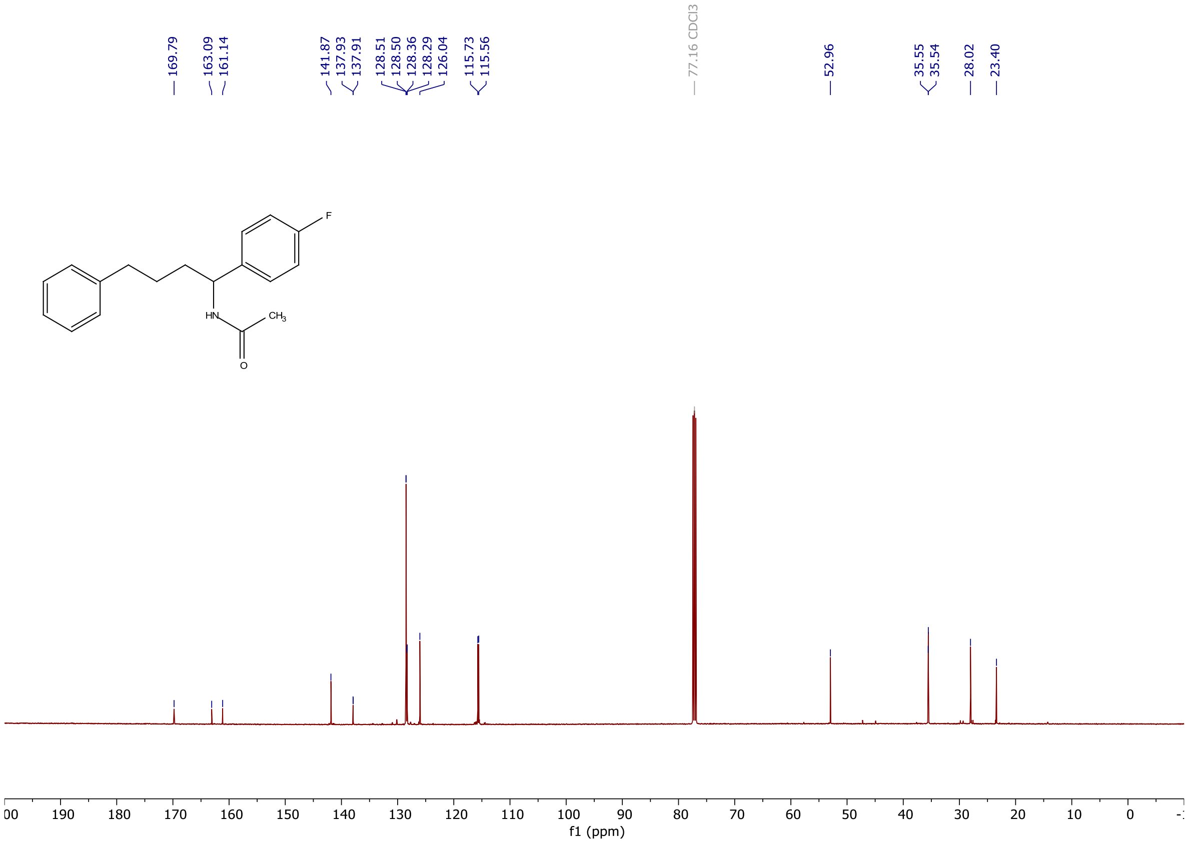

^{19}F NMR (376 MHz, $CDCl_3$) of **21**

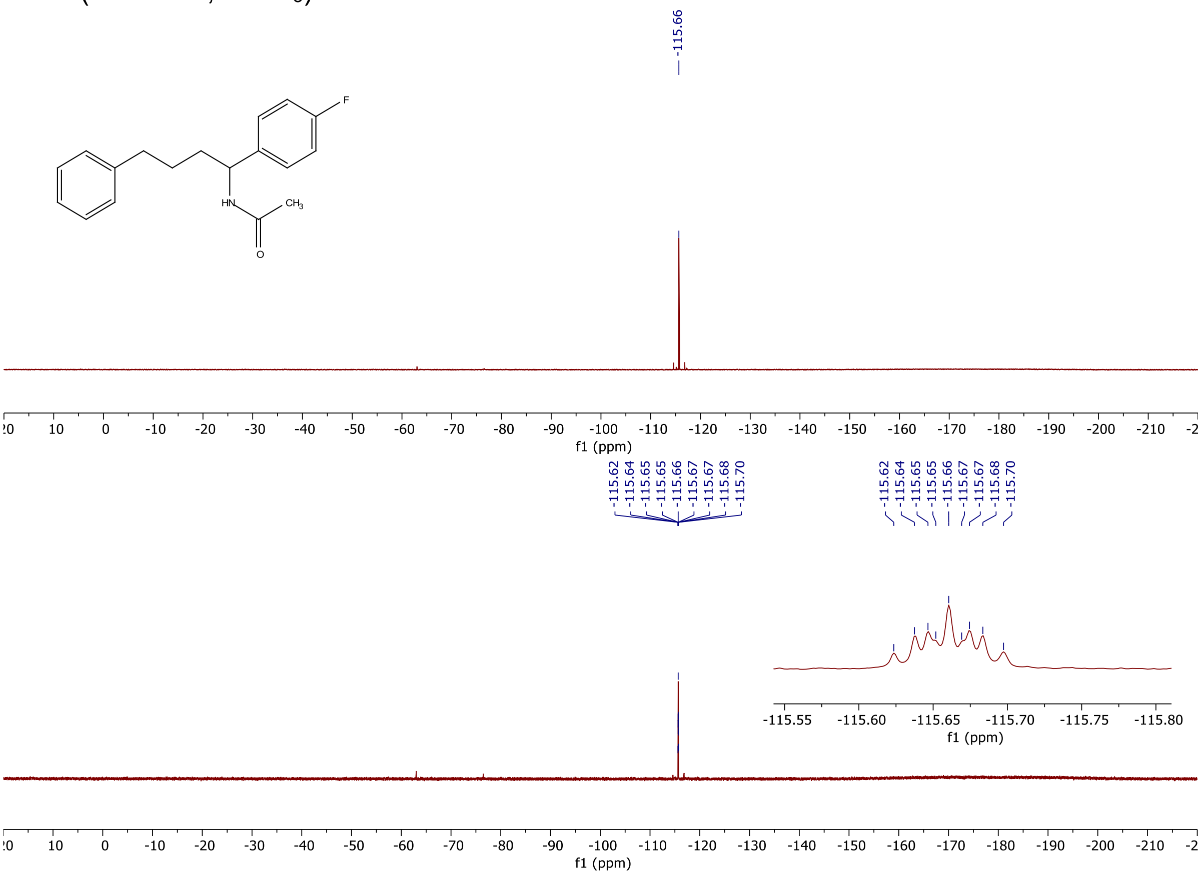

^{1}H NMR (400 MHz, $CDCl_3$) of **22**

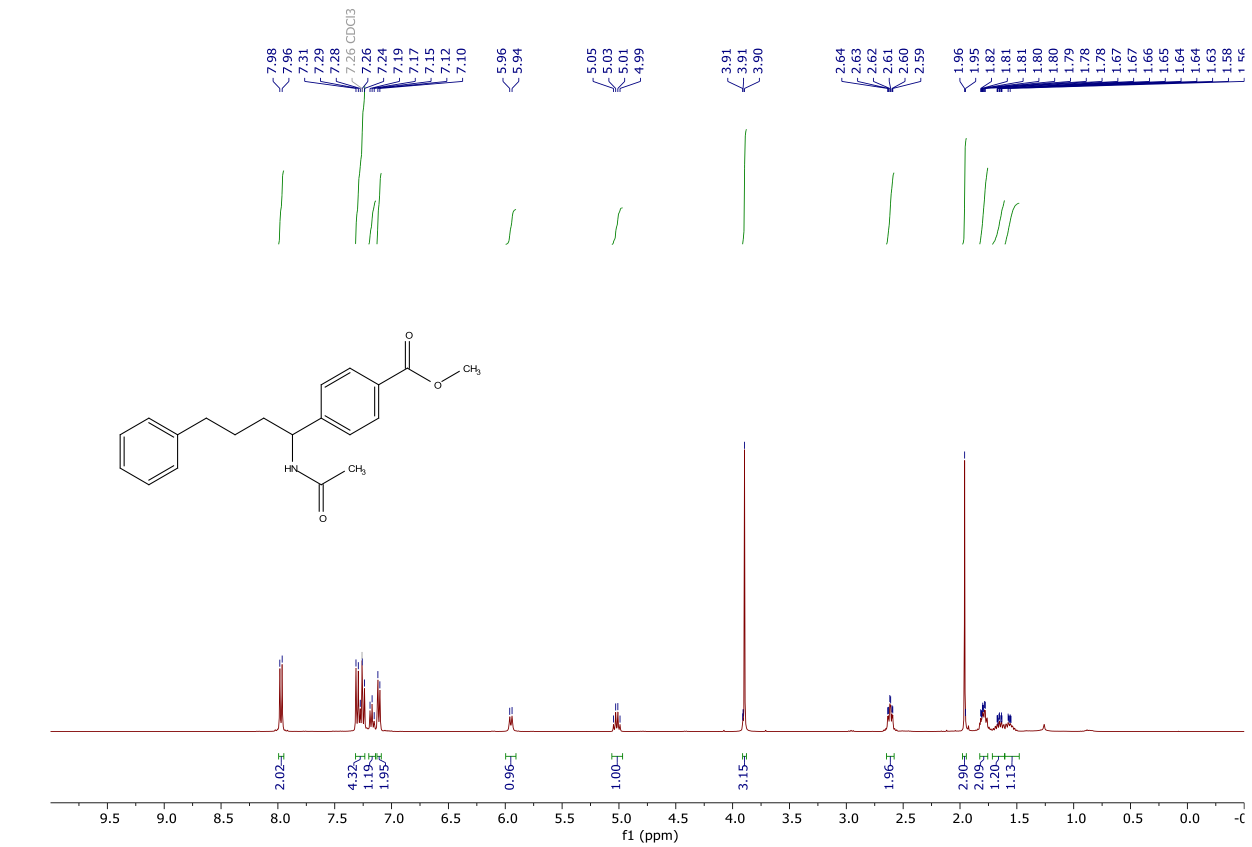

^{13}C NMR (101 MHz, $CDCl_3$) of **22**

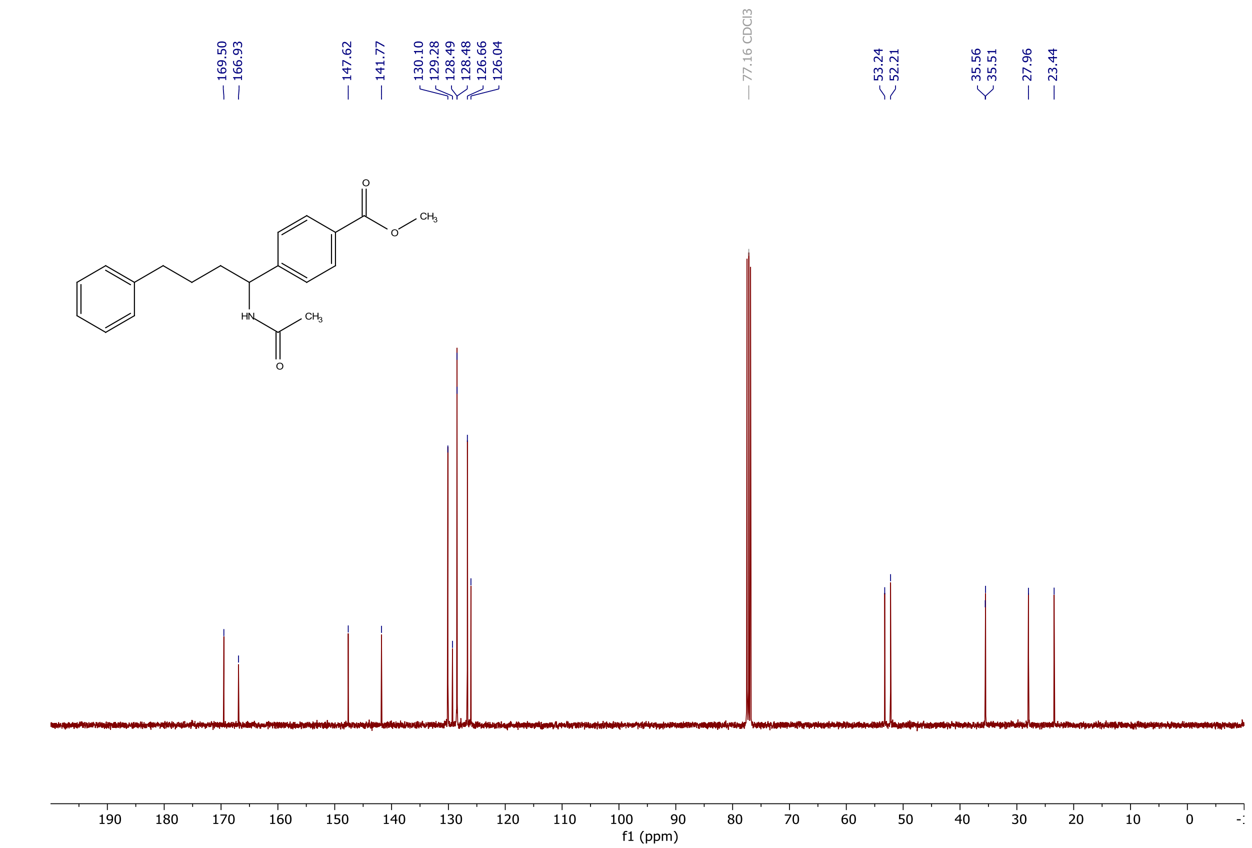

^{1}H NMR (400 MHz, $CDCl_3$) of **23**

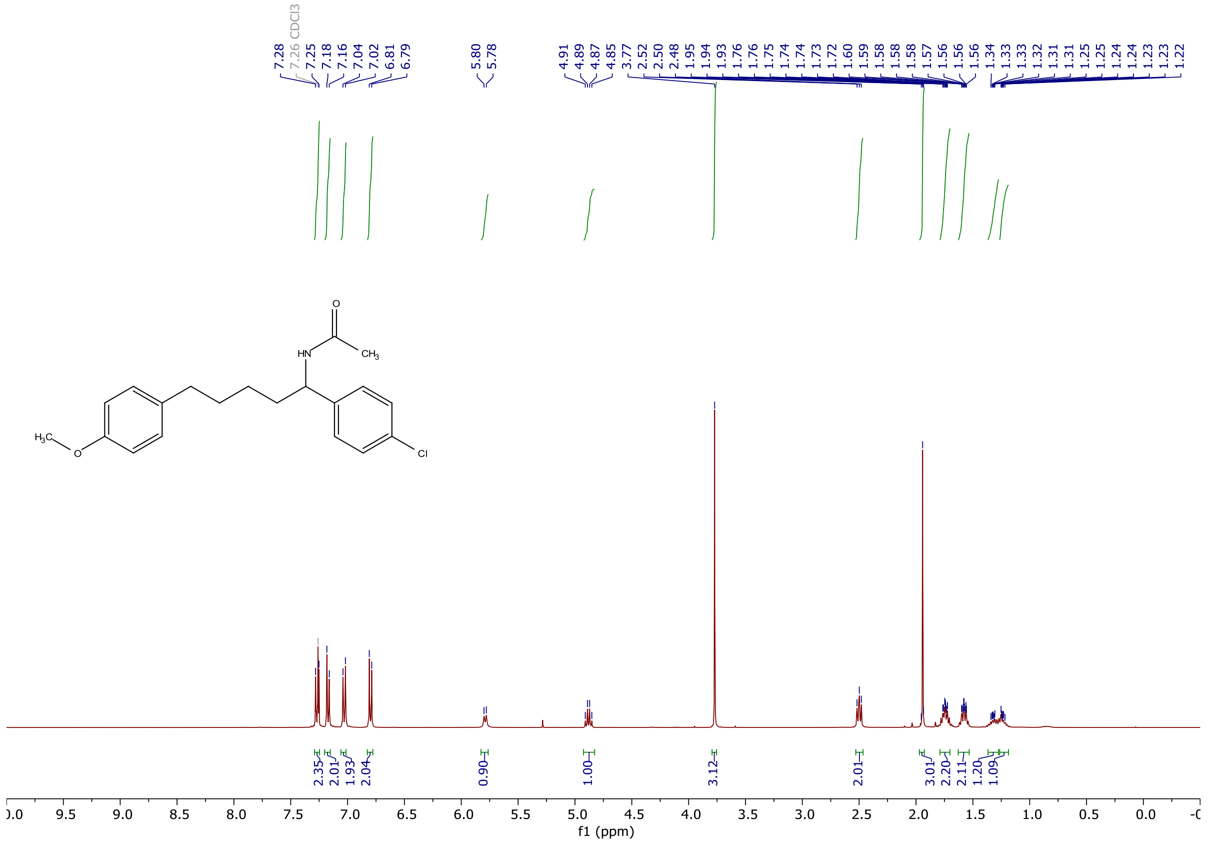

^{13}C NMR (101 MHz, $CDCl_3$) of **23**

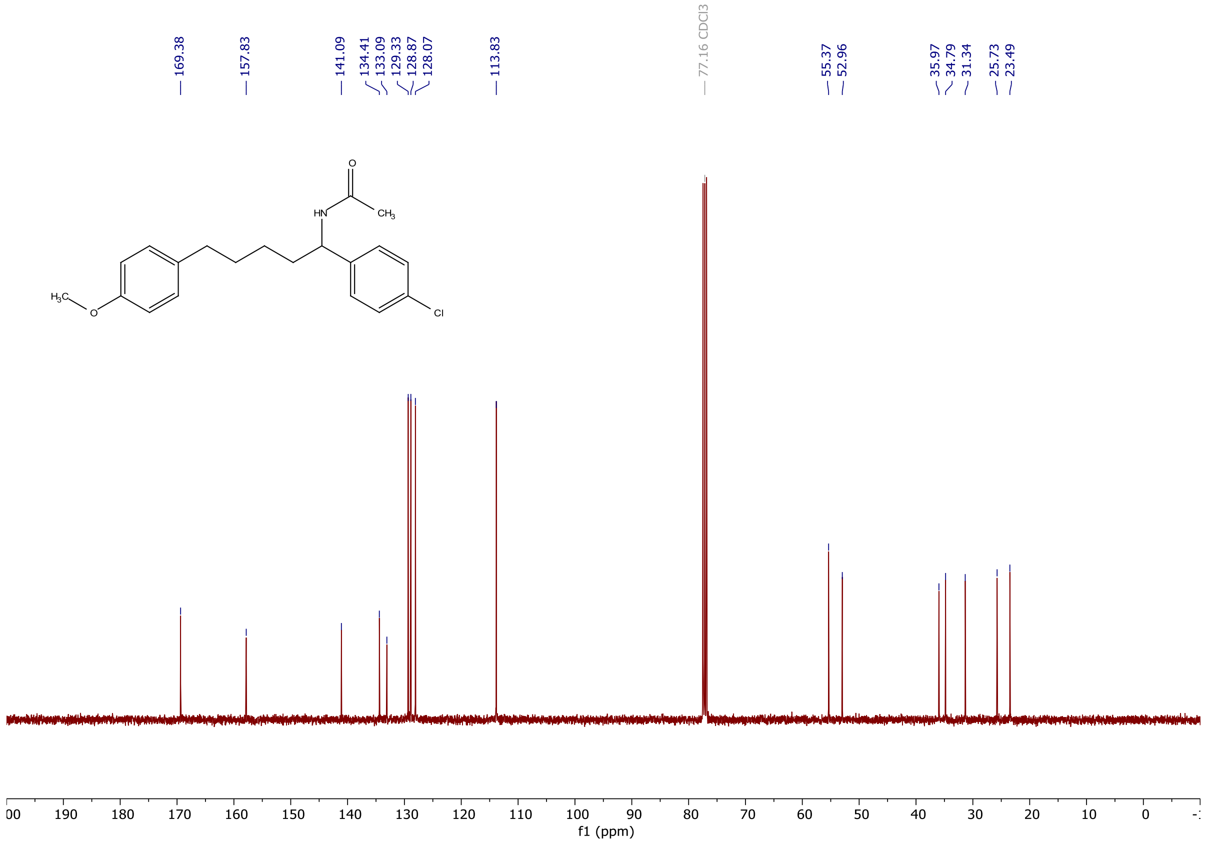

^{1}H NMR (400 MHz, $CDCl_3$) of **24**

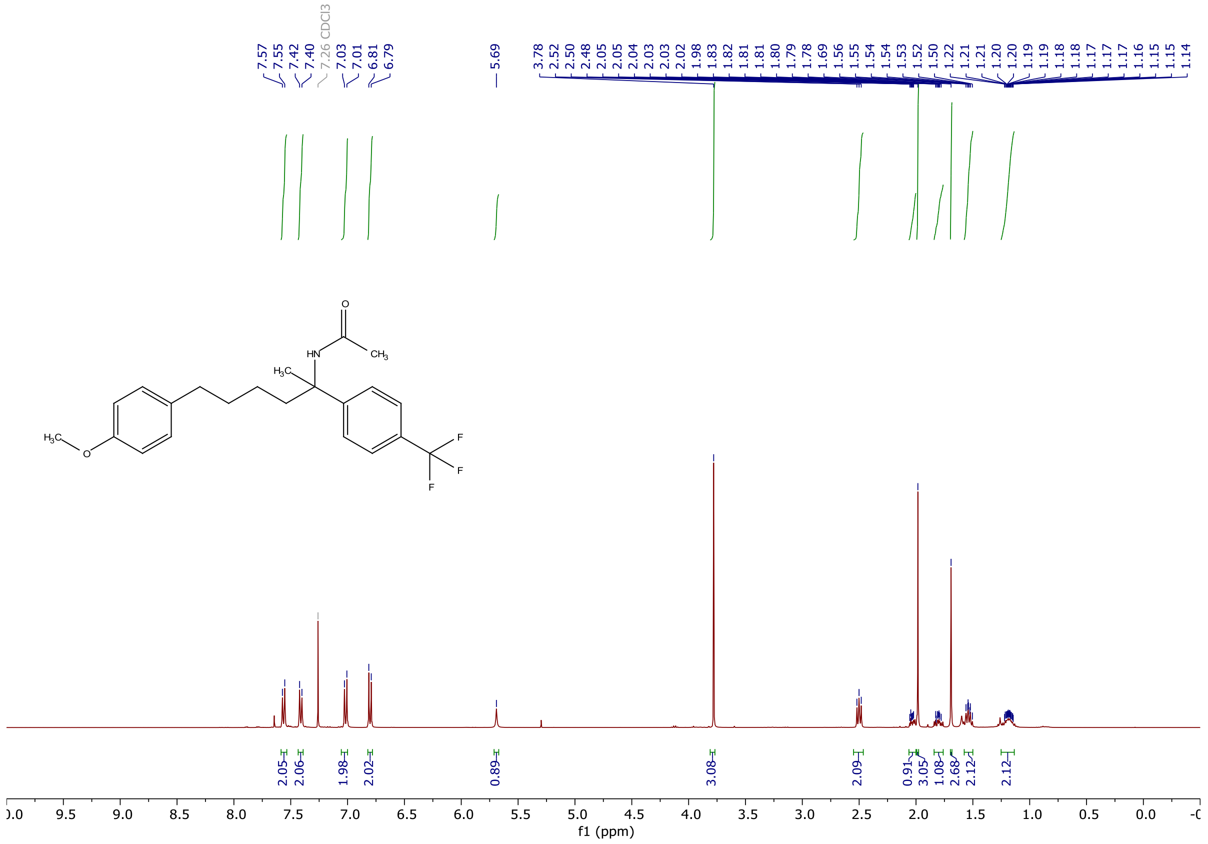

^{13}C NMR (151 MHz, $CDCl_3$) of **24**

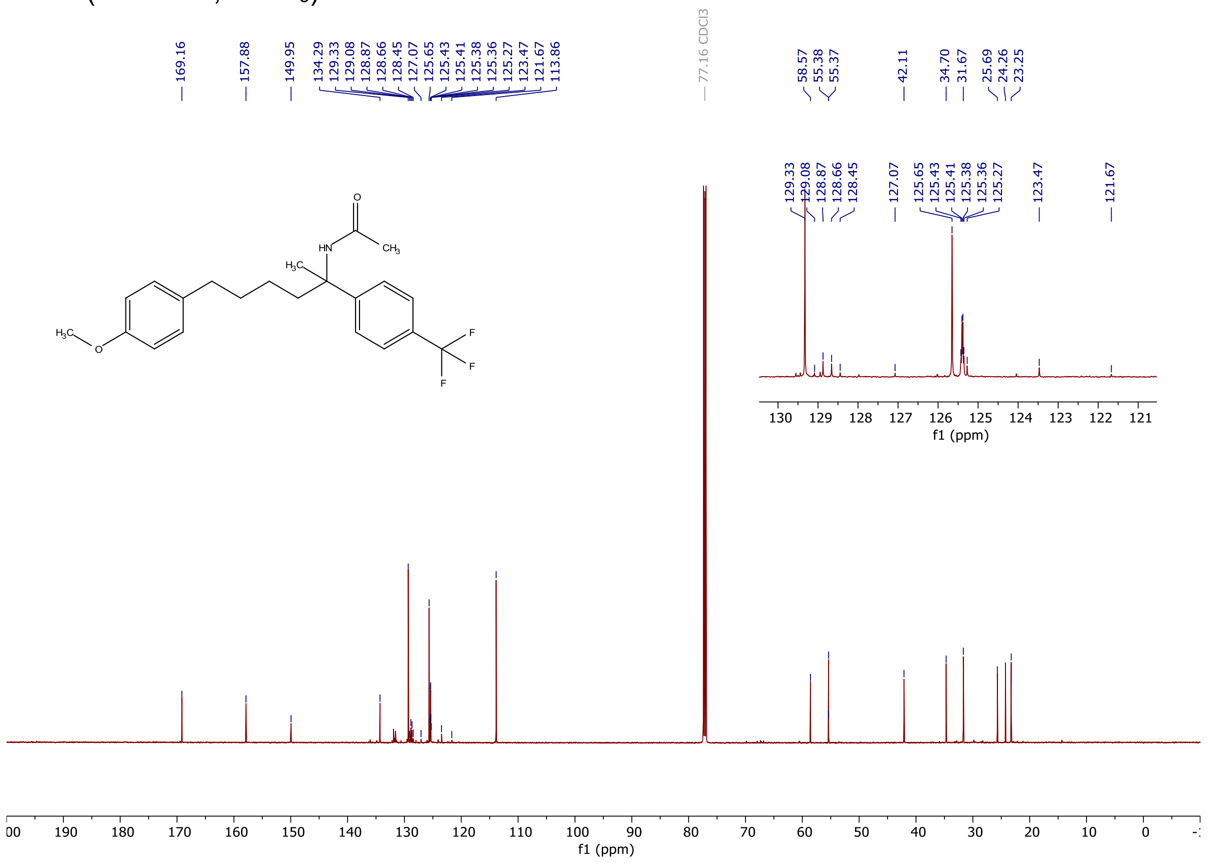

^{19}F NMR (376 MHz, $CDCl_3$) of **24**

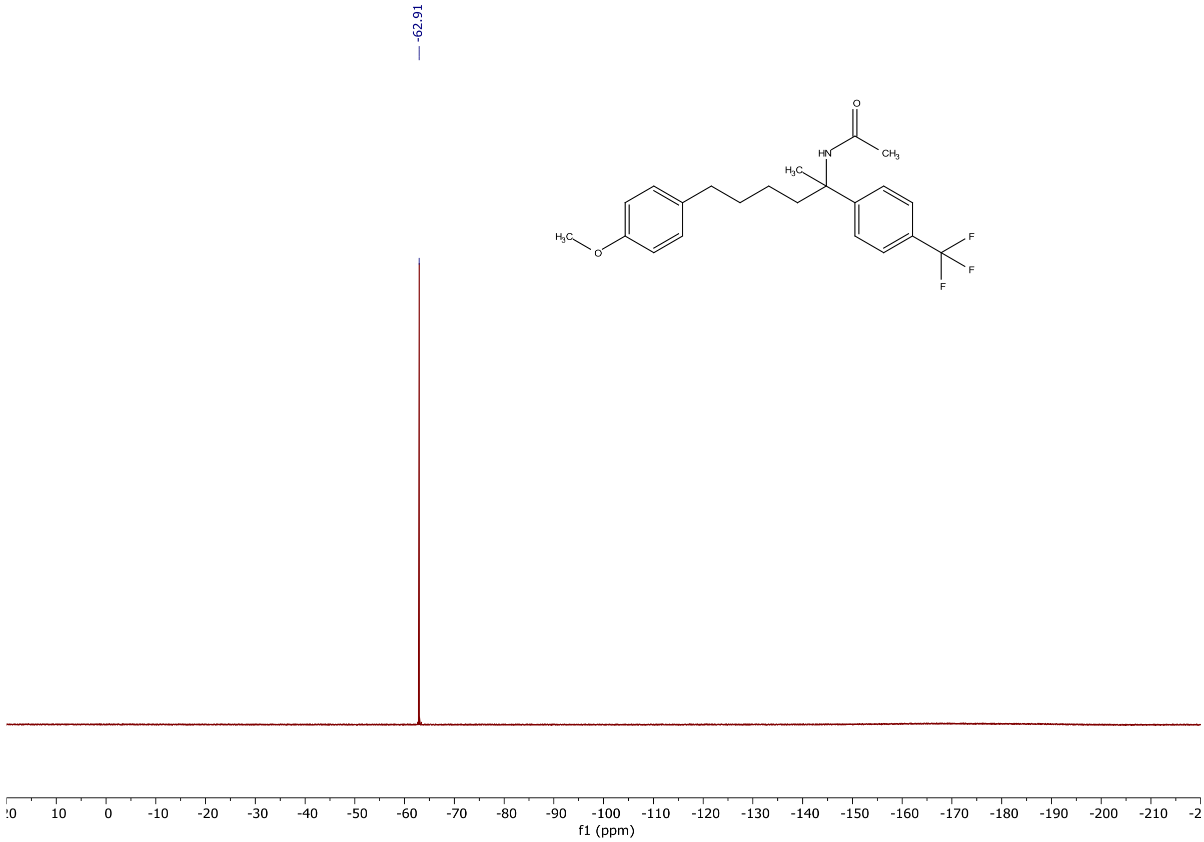

^{1}H NMR (400 MHz, $CDCl_3$) of **25**

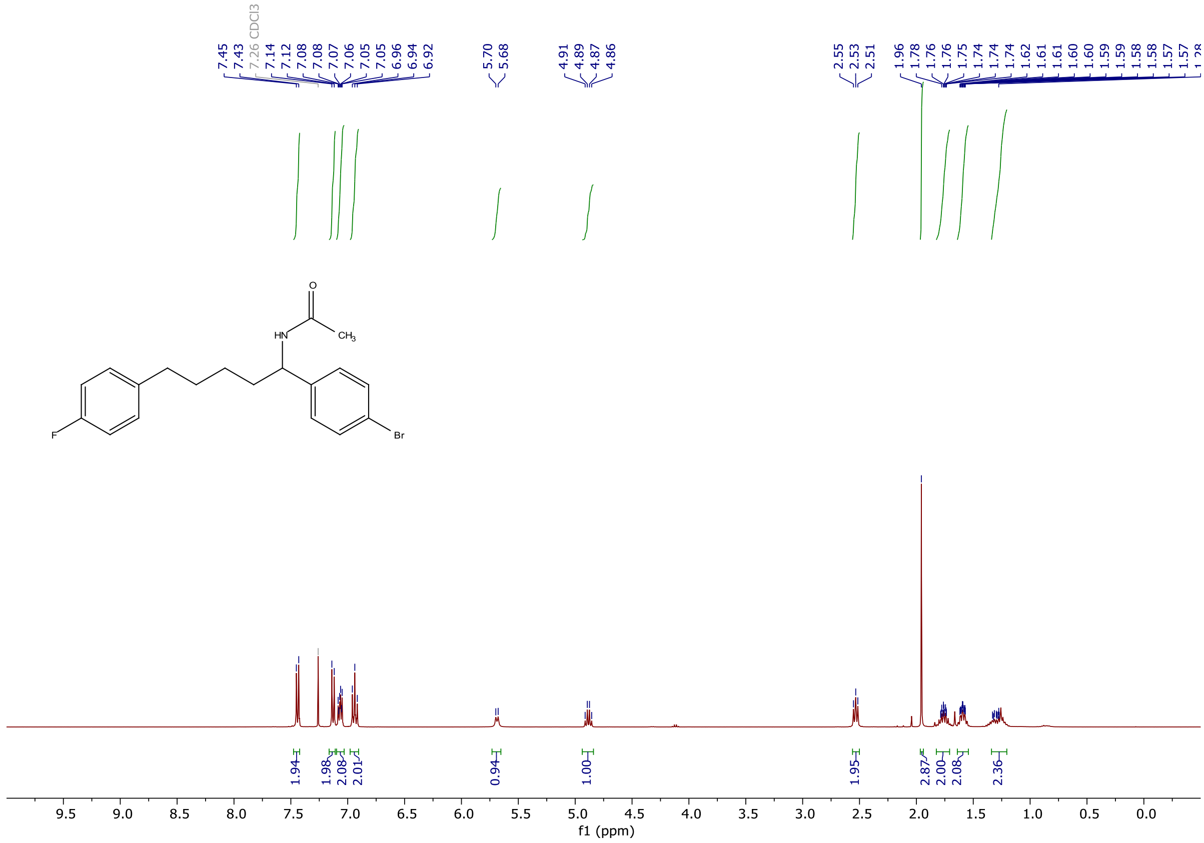

^{13}C NMR (126 MHz, $CDCl_3$) of **25**

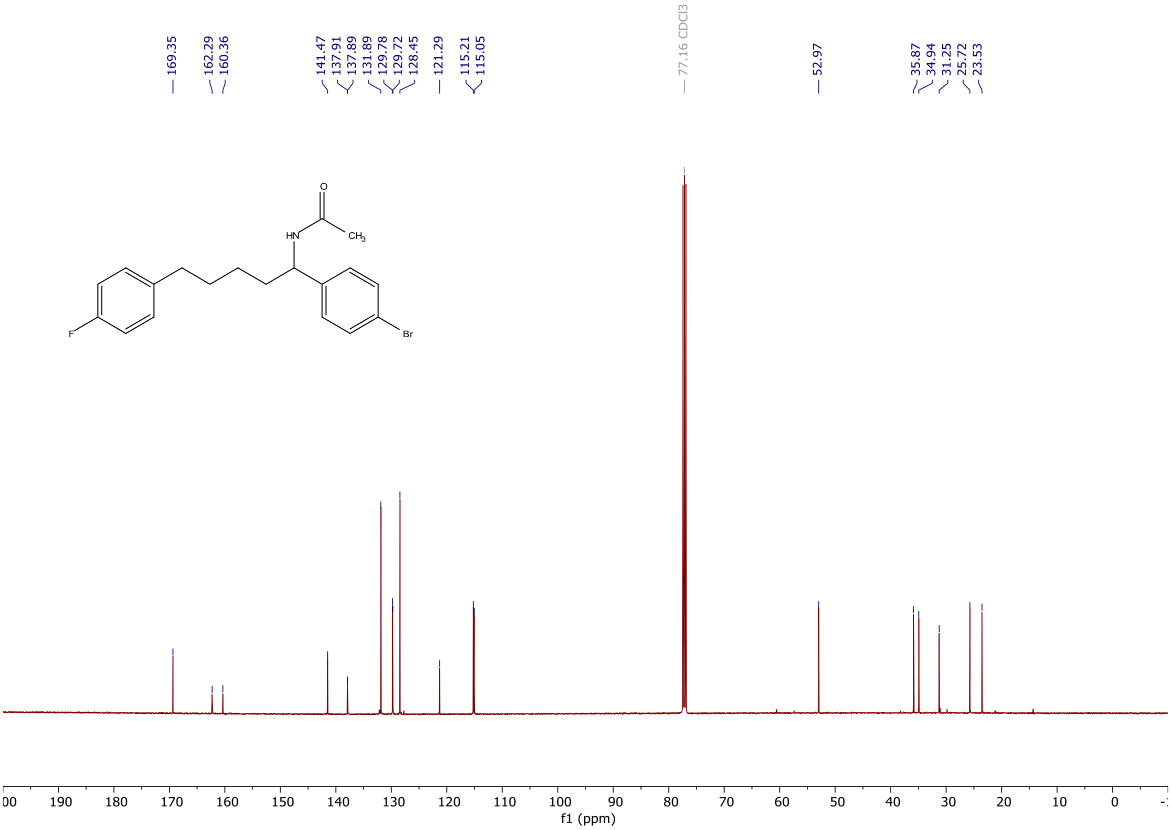

^{19}F NMR (376 MHz, $CDCl_3$) of **25**

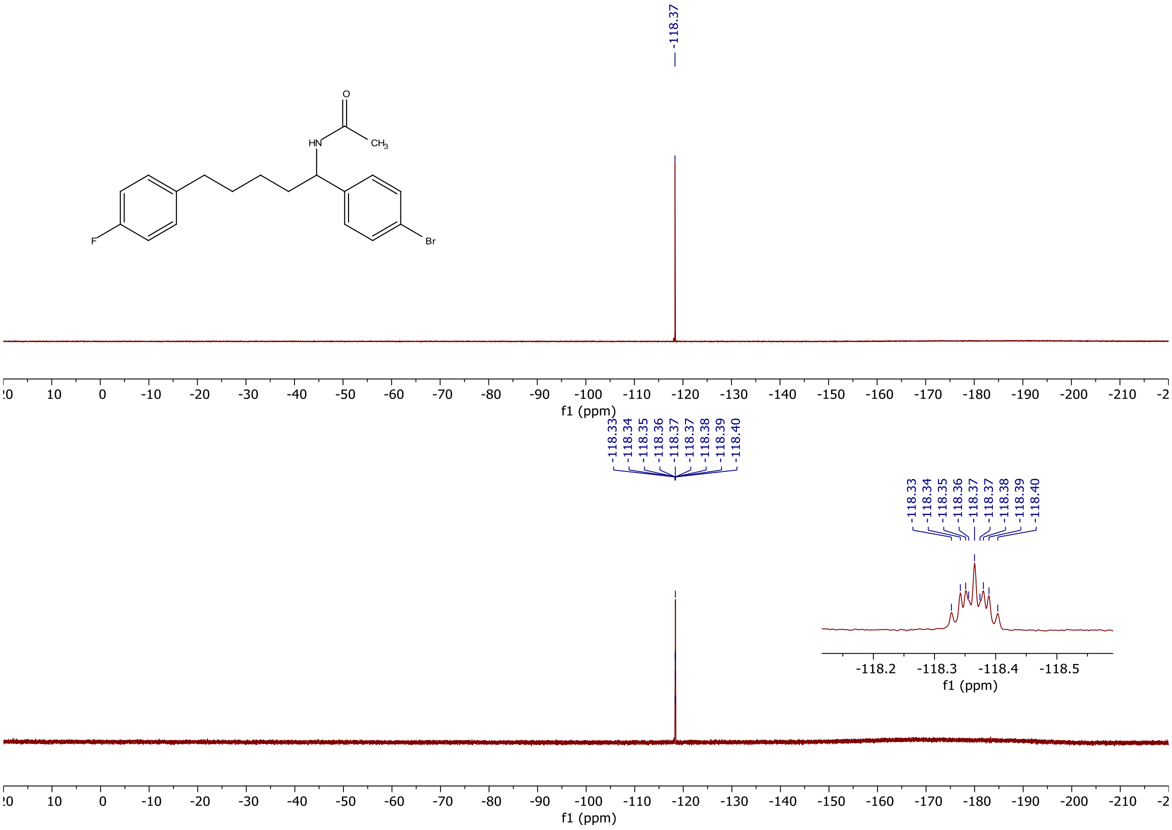

^{1}H NMR (400 MHz, $CDCl_3$) of **26**

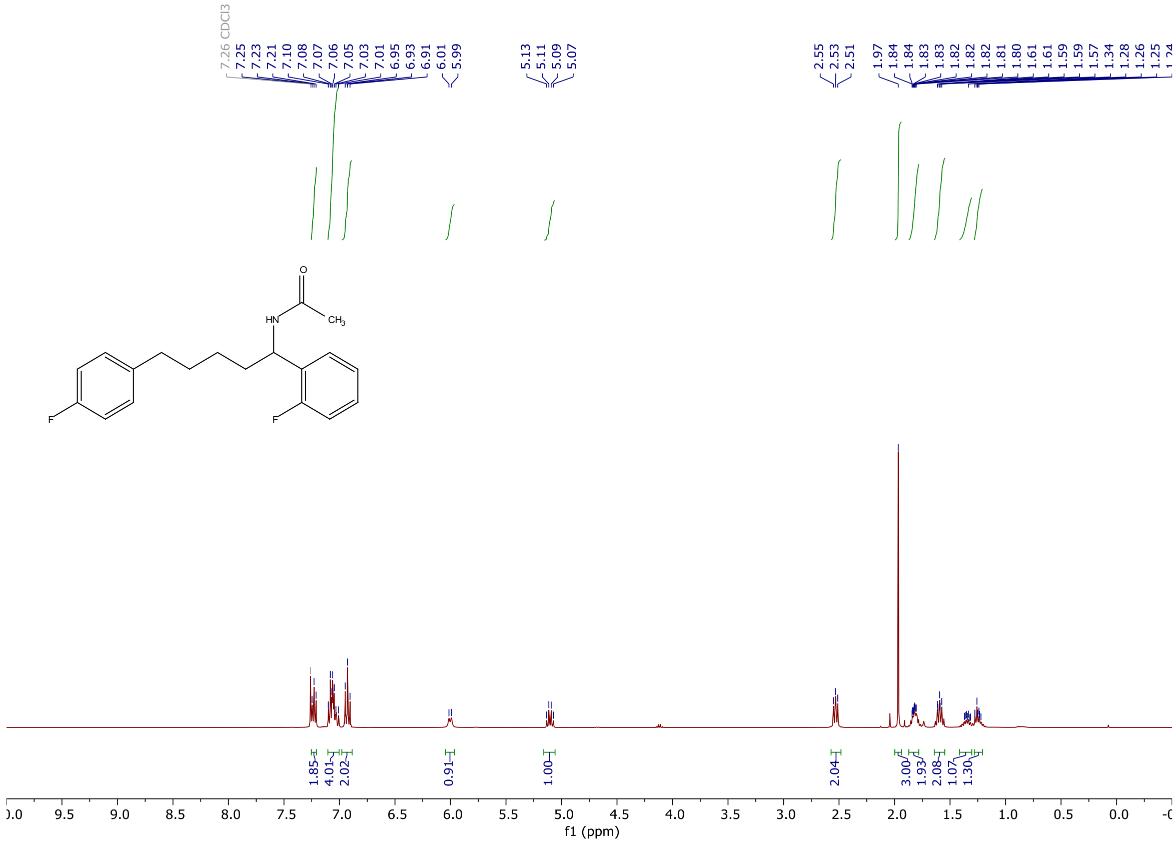

^{13}C NMR (126 MHz, $CDCl_3$) of **26**

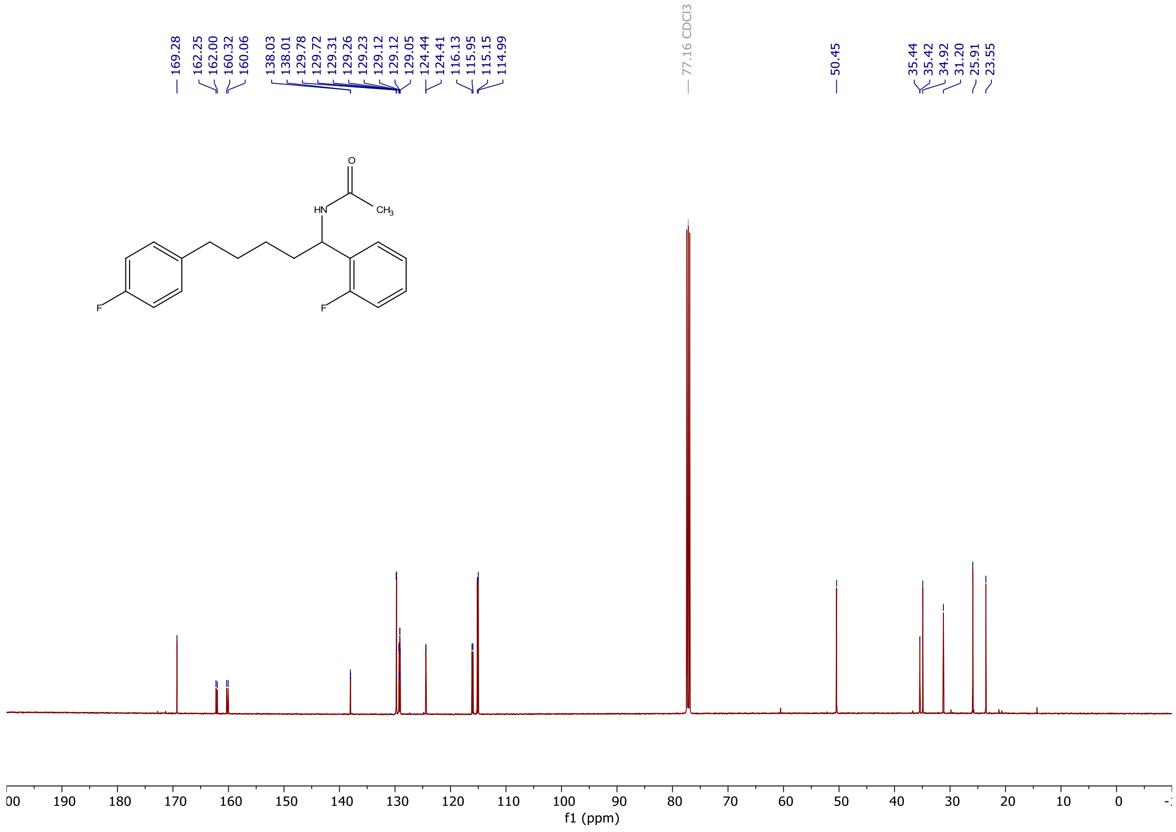

^{19}F NMR (376 MHz, $CDCl_3$) of **26**

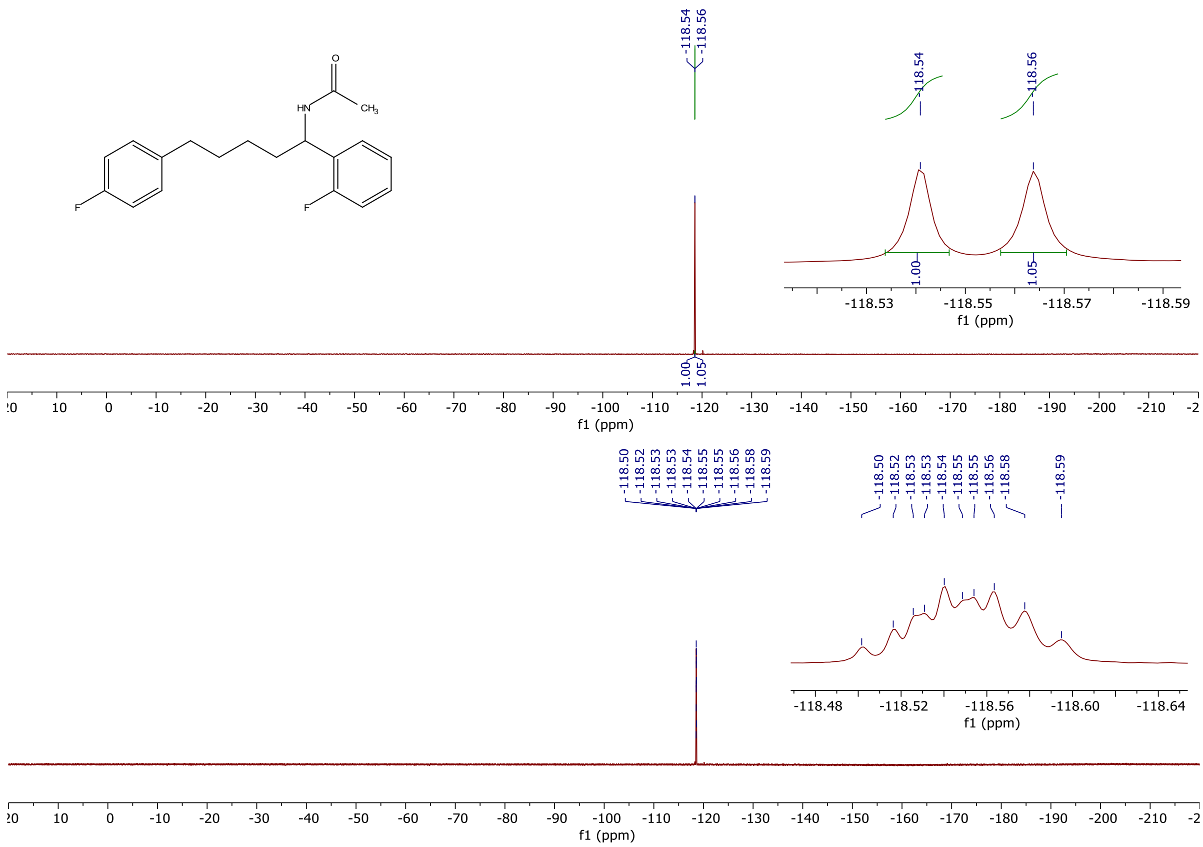

^{1}H NMR (400 MHz, $CDCl_3$) of **27**

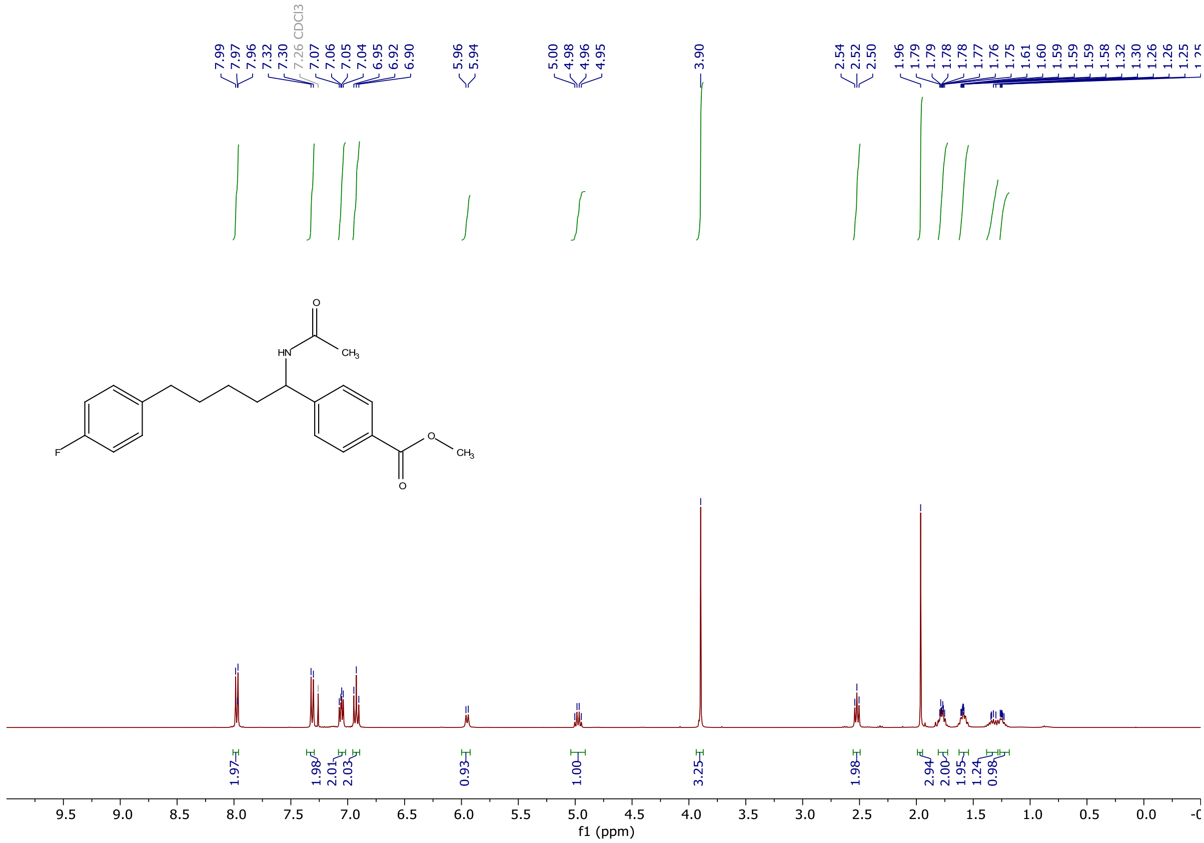

^{13}C NMR (101 MHz, $CDCl_3$) of **27**

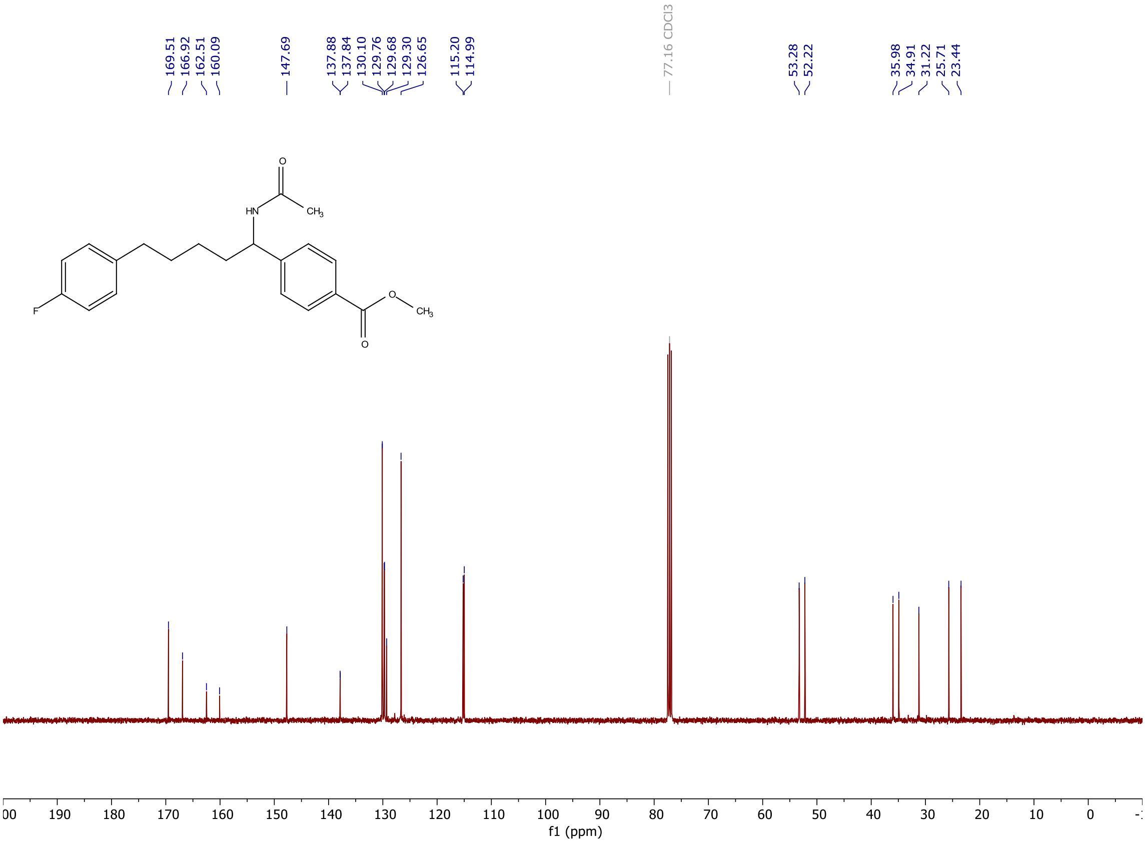

^{19}F NMR (376 MHz, $CDCl_3$) of **27**

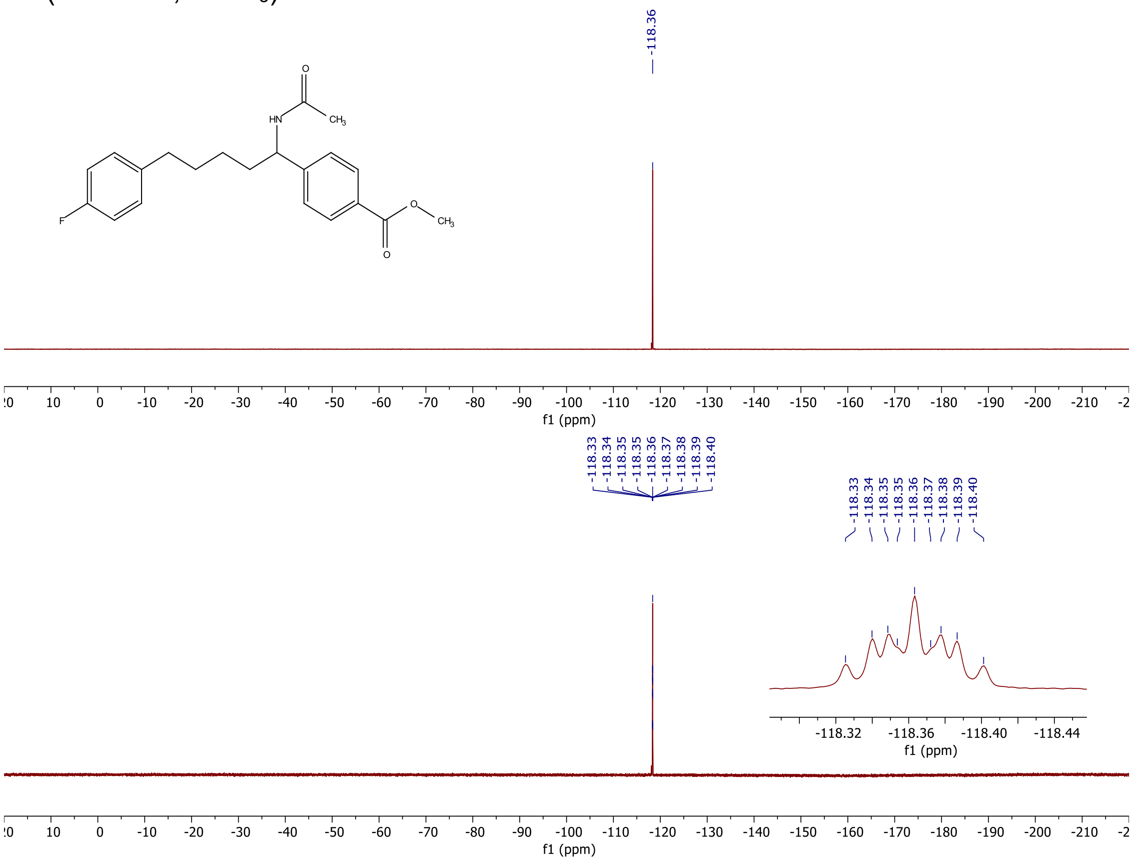

^{1}H NMR (400 MHz, $CDCl_3$) of **28**

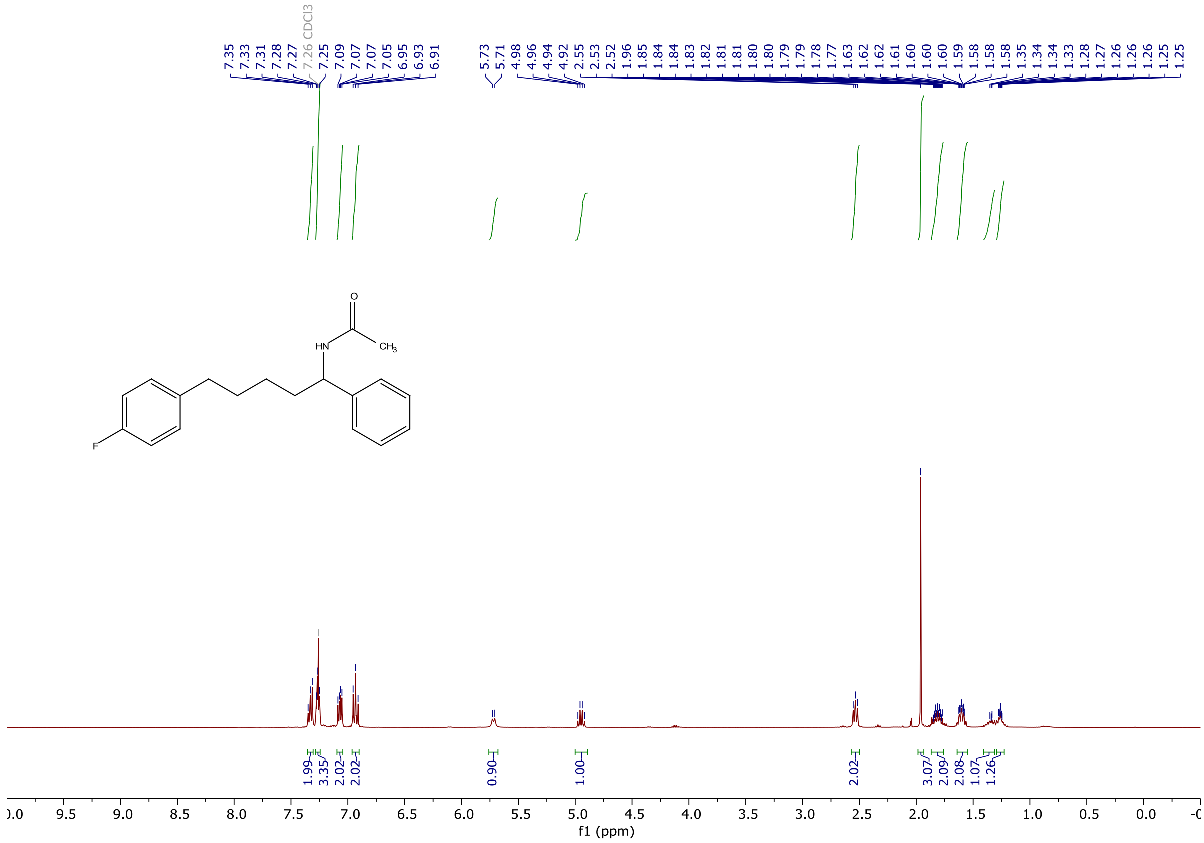

^{13}C NMR (101 MHz, $CDCl_3$) of **28**

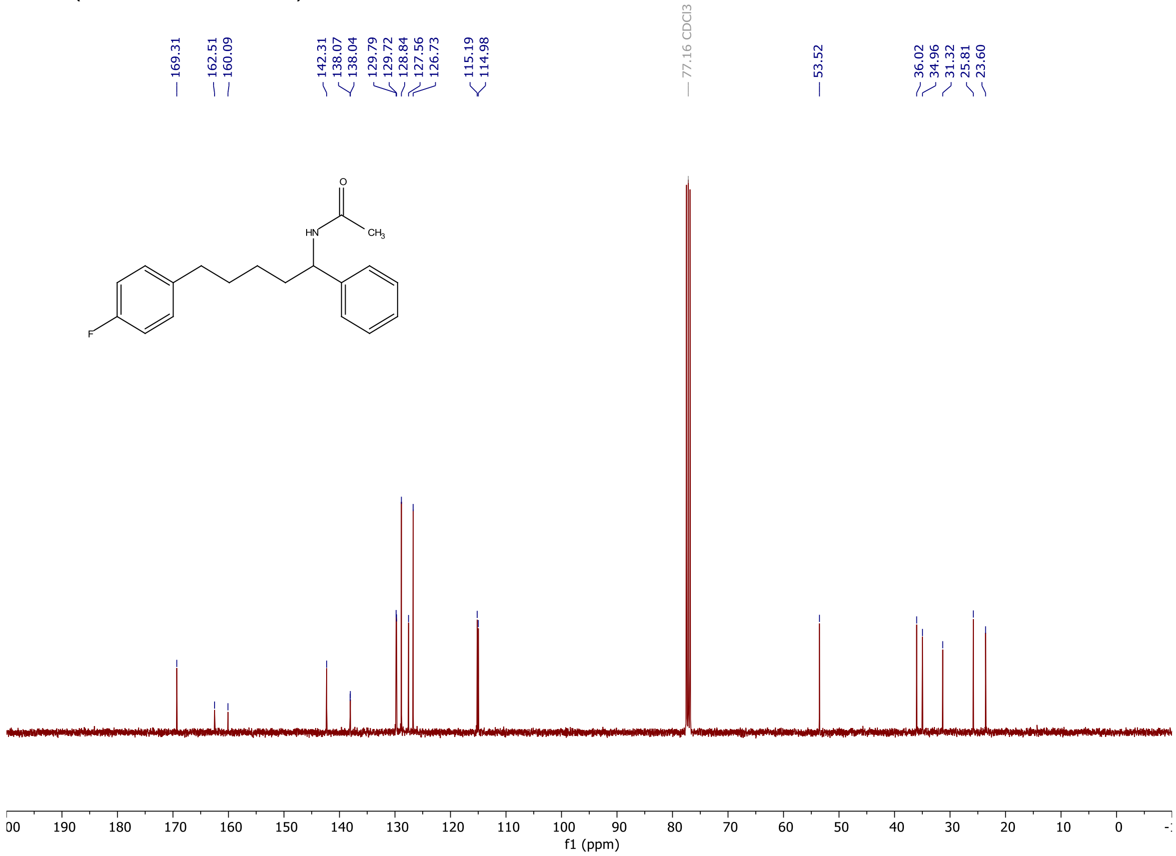

^{19}F NMR (376 MHz, $CDCl_3$) of **28**

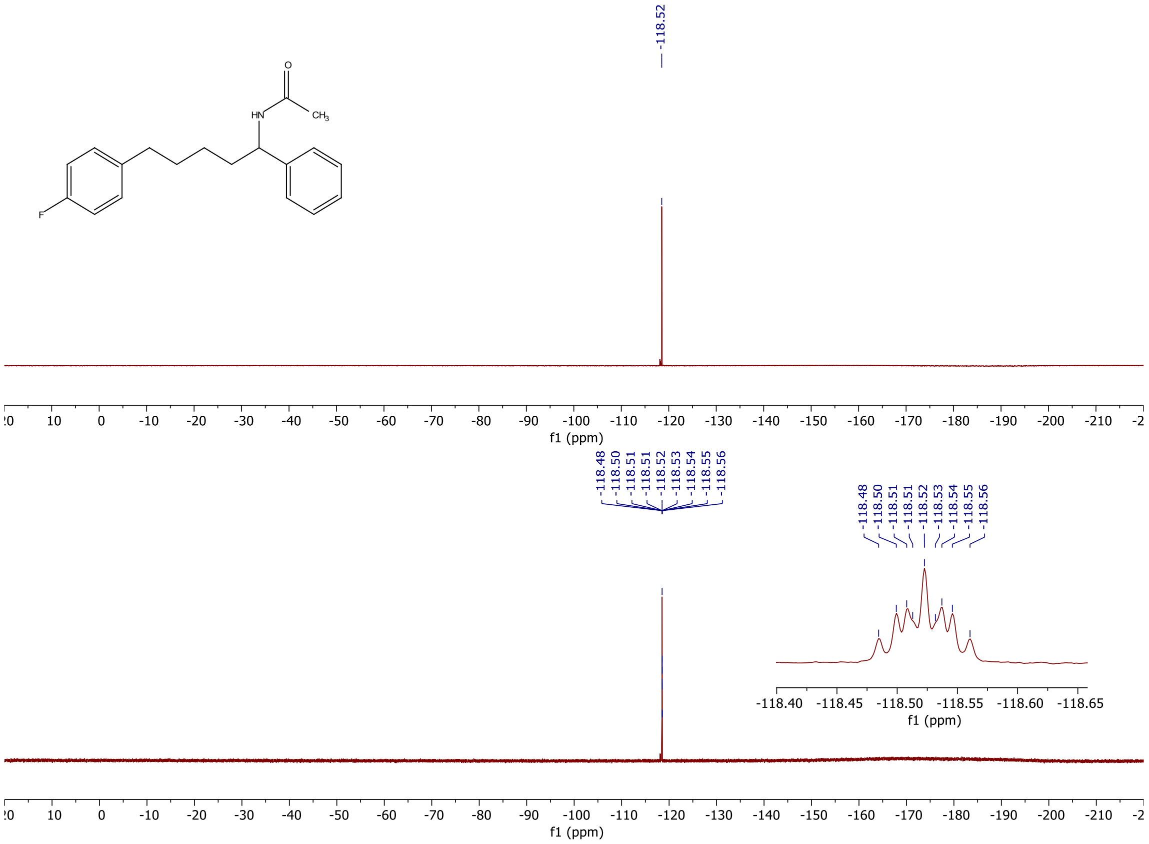

^{1}H NMR (400 MHz, $CDCl_3$) of **29**

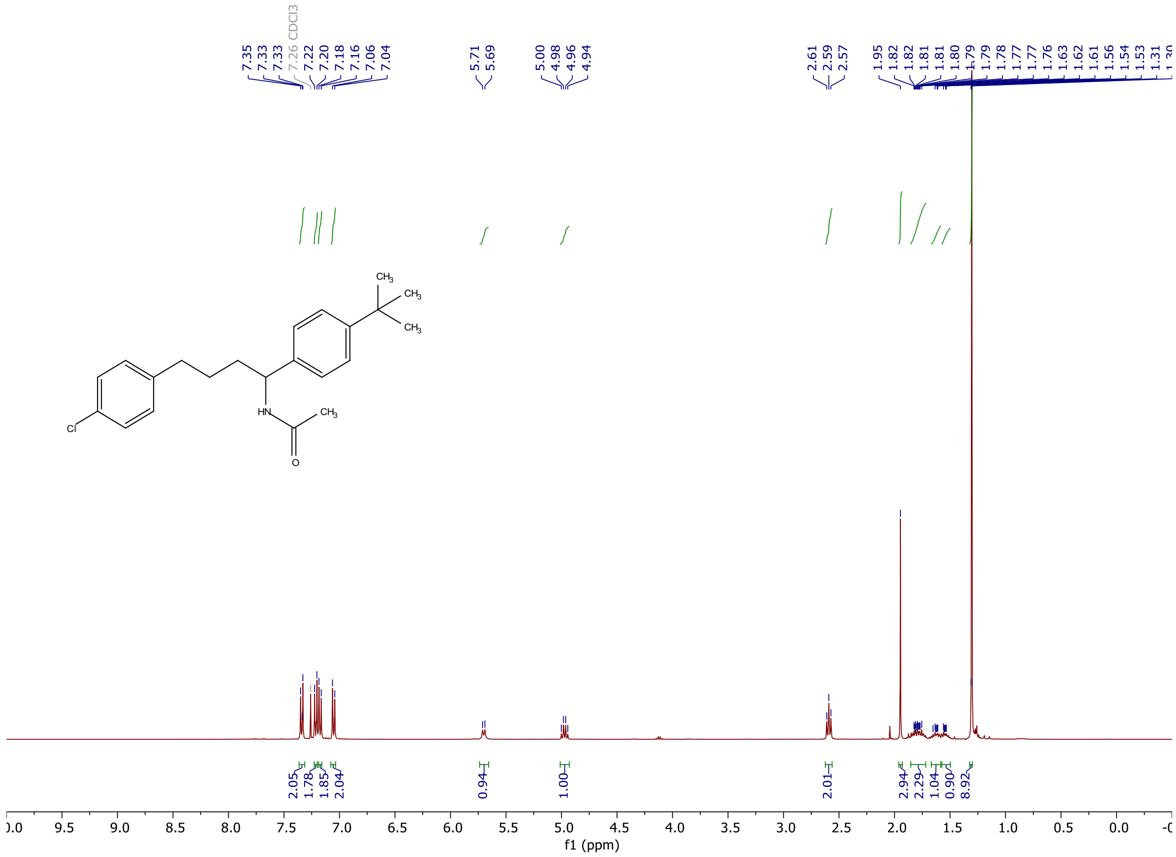

^{13}C NMR (101 MHz, $CDCl_3$) of **29**

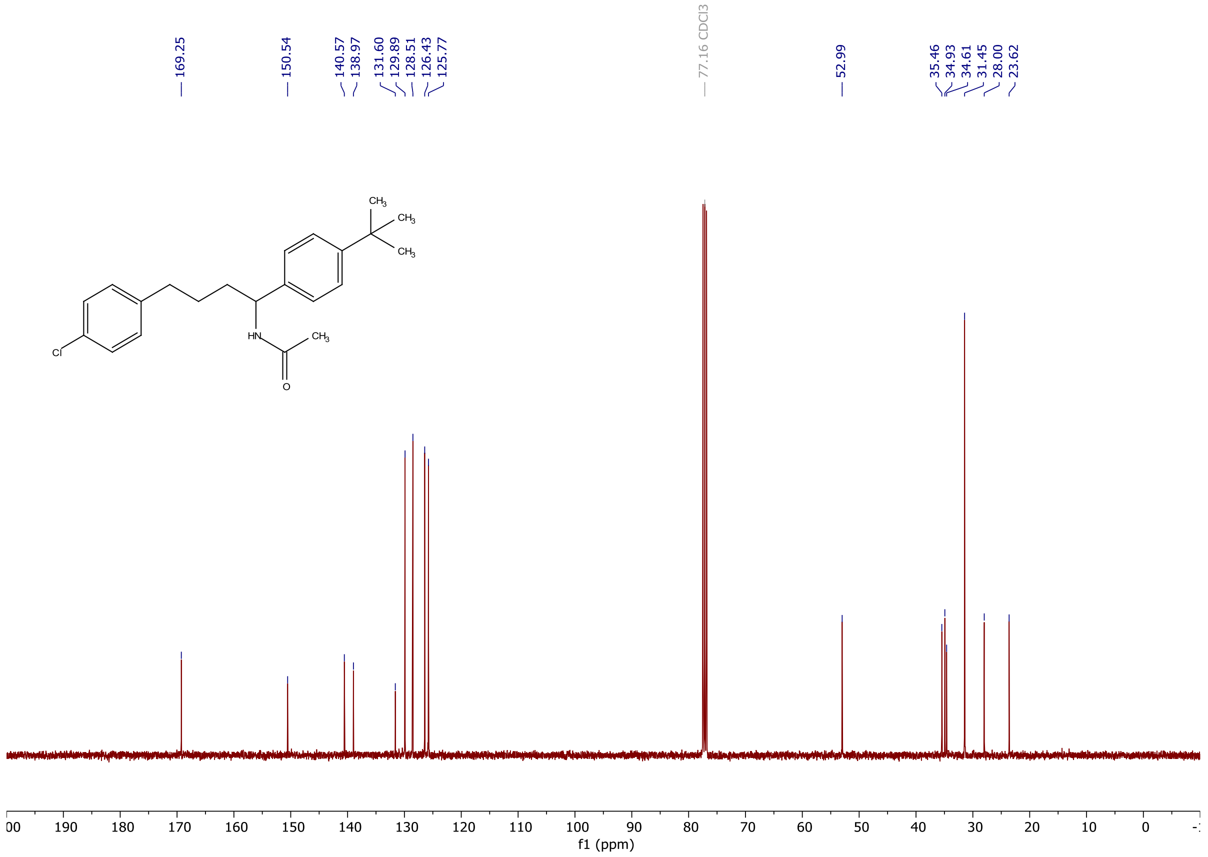

^{1}H NMR (400 MHz, $CDCl_3$) of **30**

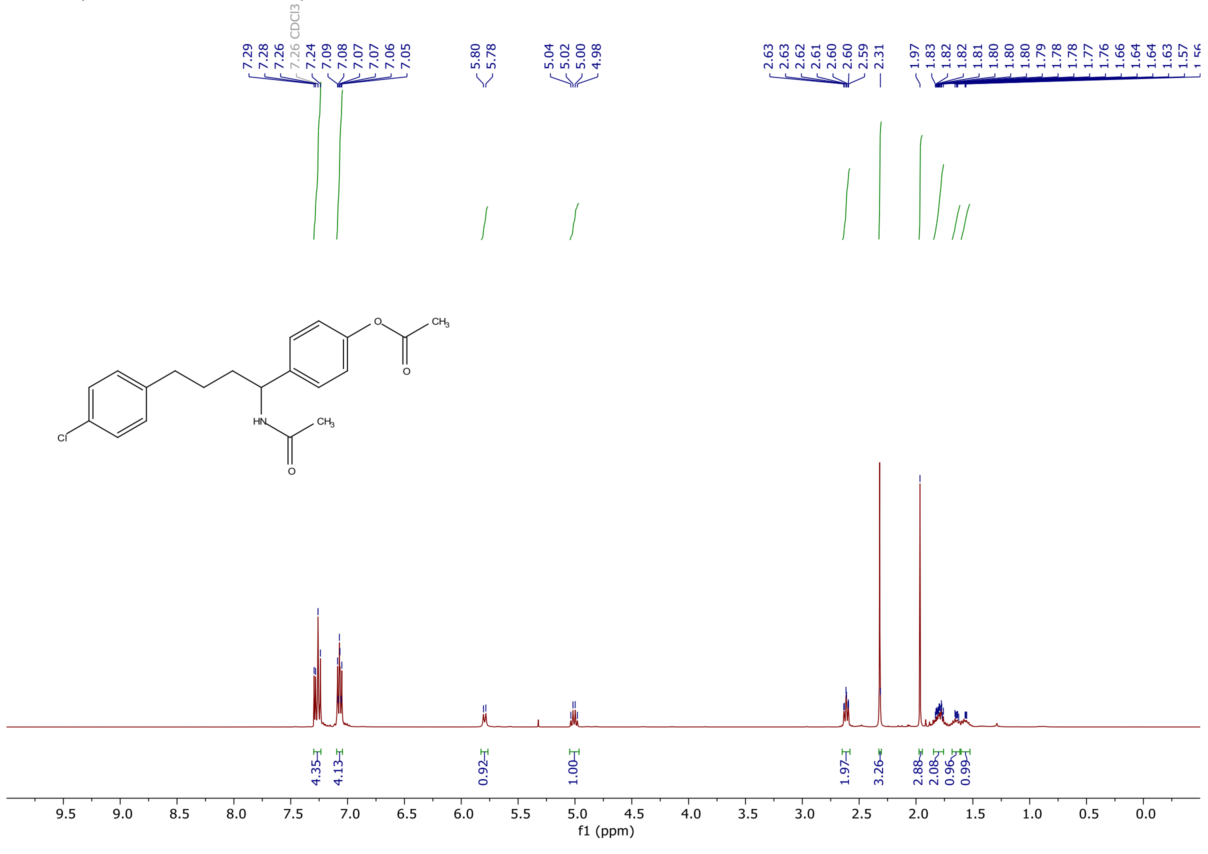

^{13}C NMR (101 MHz, $CDCl_3$) of **30**

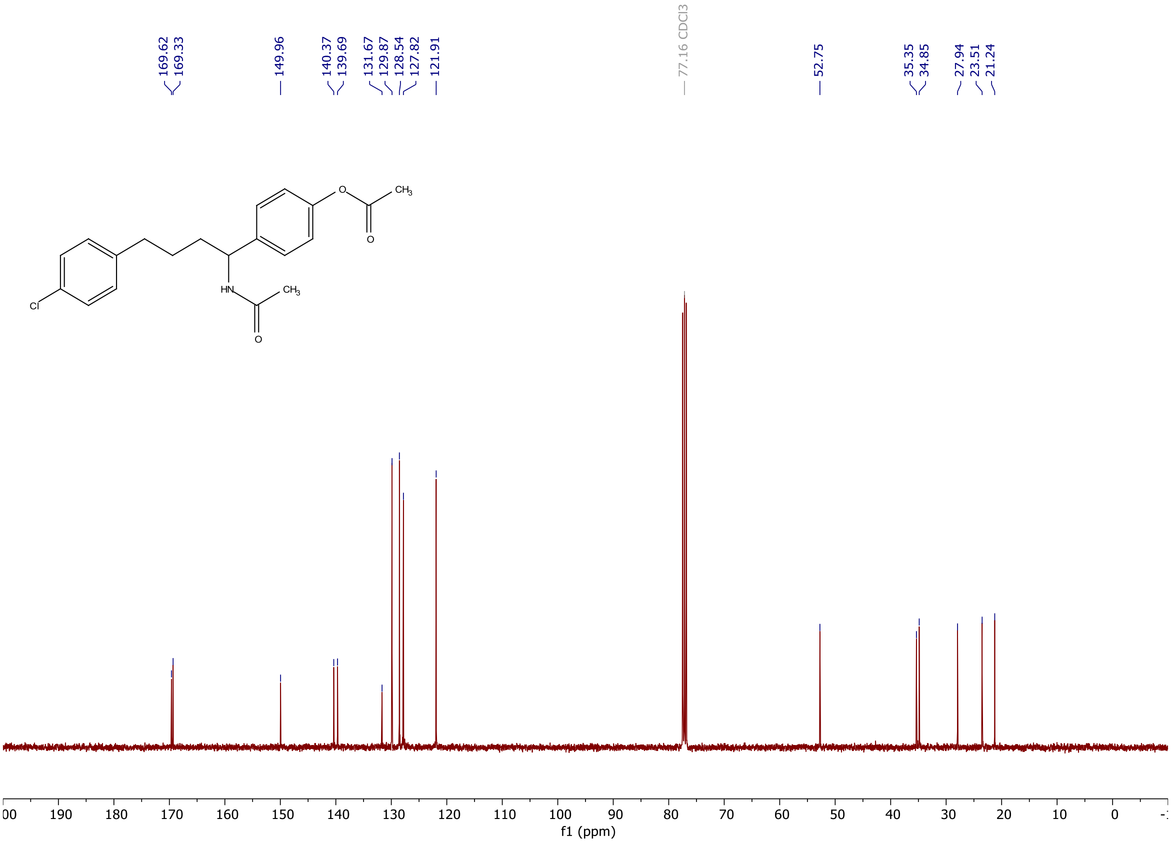

^{1}H NMR (400 MHz, $CDCl_3$) of **31**

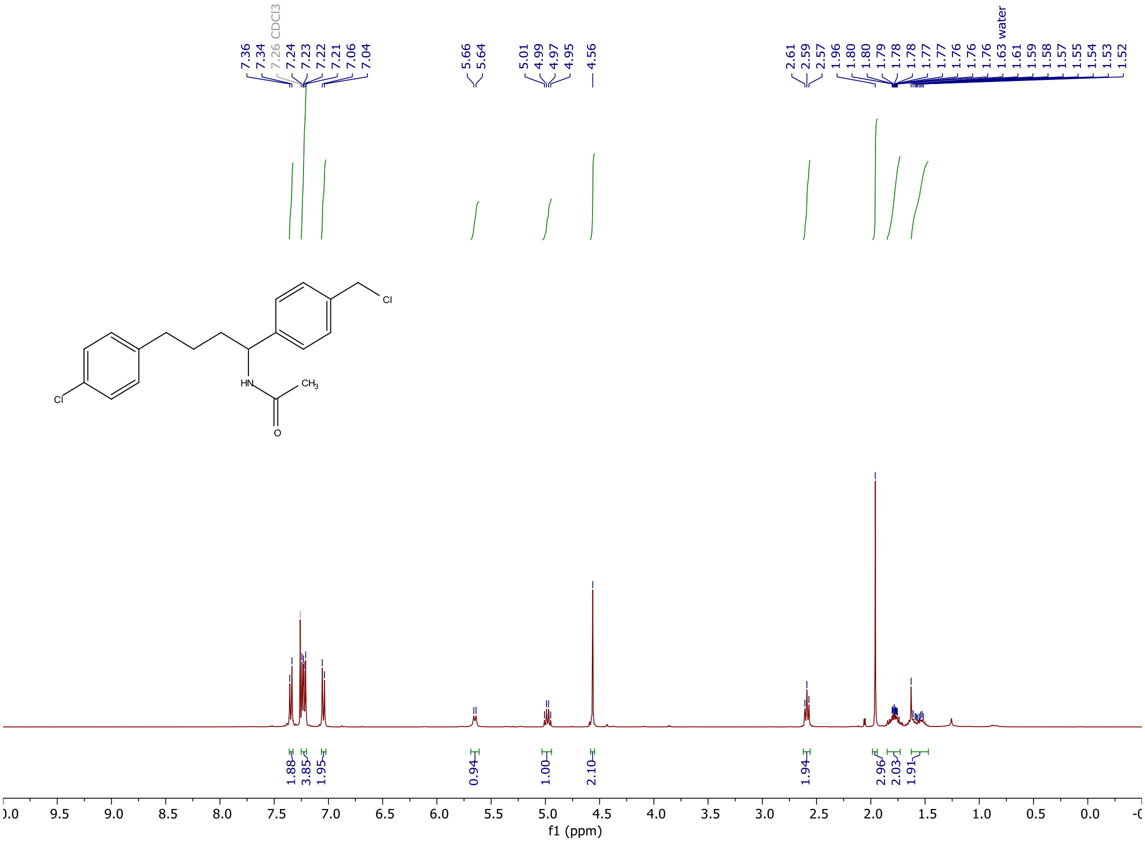

^{13}C NMR (101 MHz, $CDCl_3$) of **31**

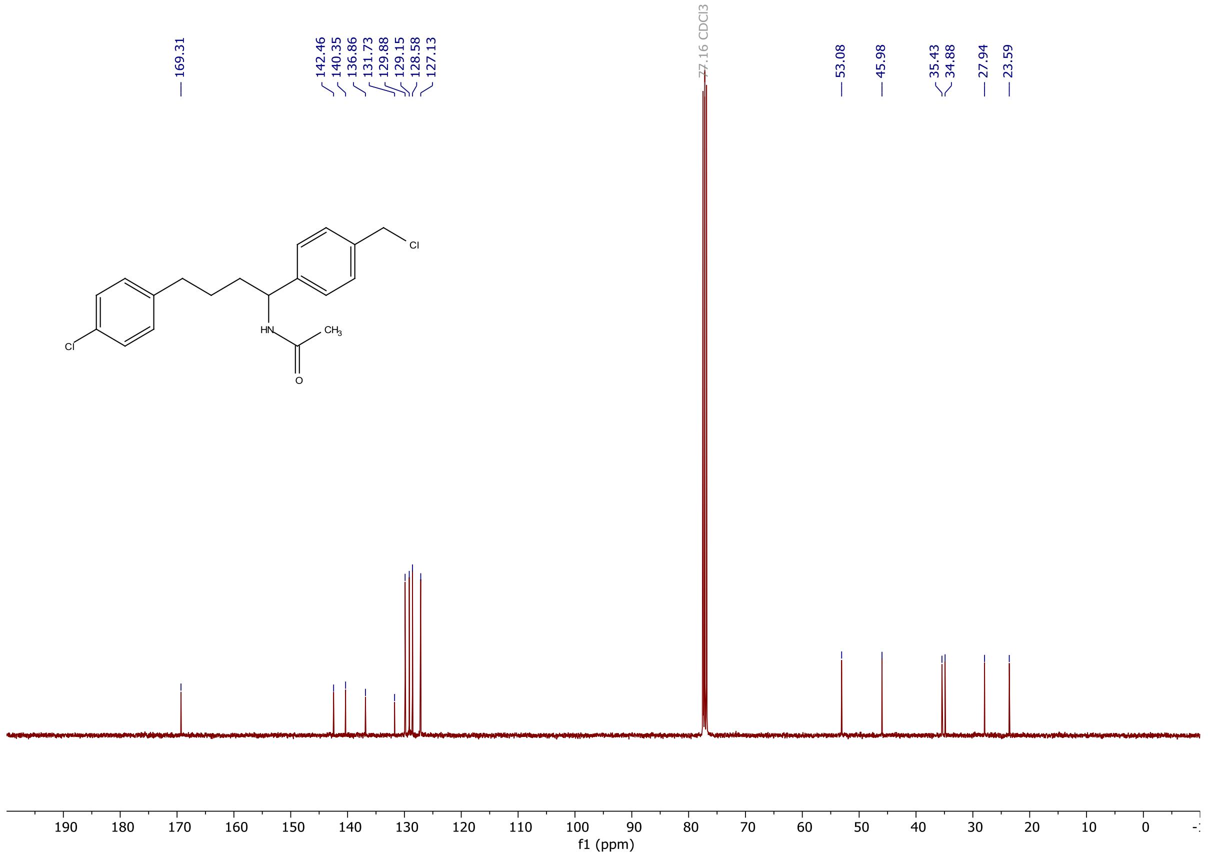

^{1}H NMR (400 MHz, $CDCl_3$) of **32**

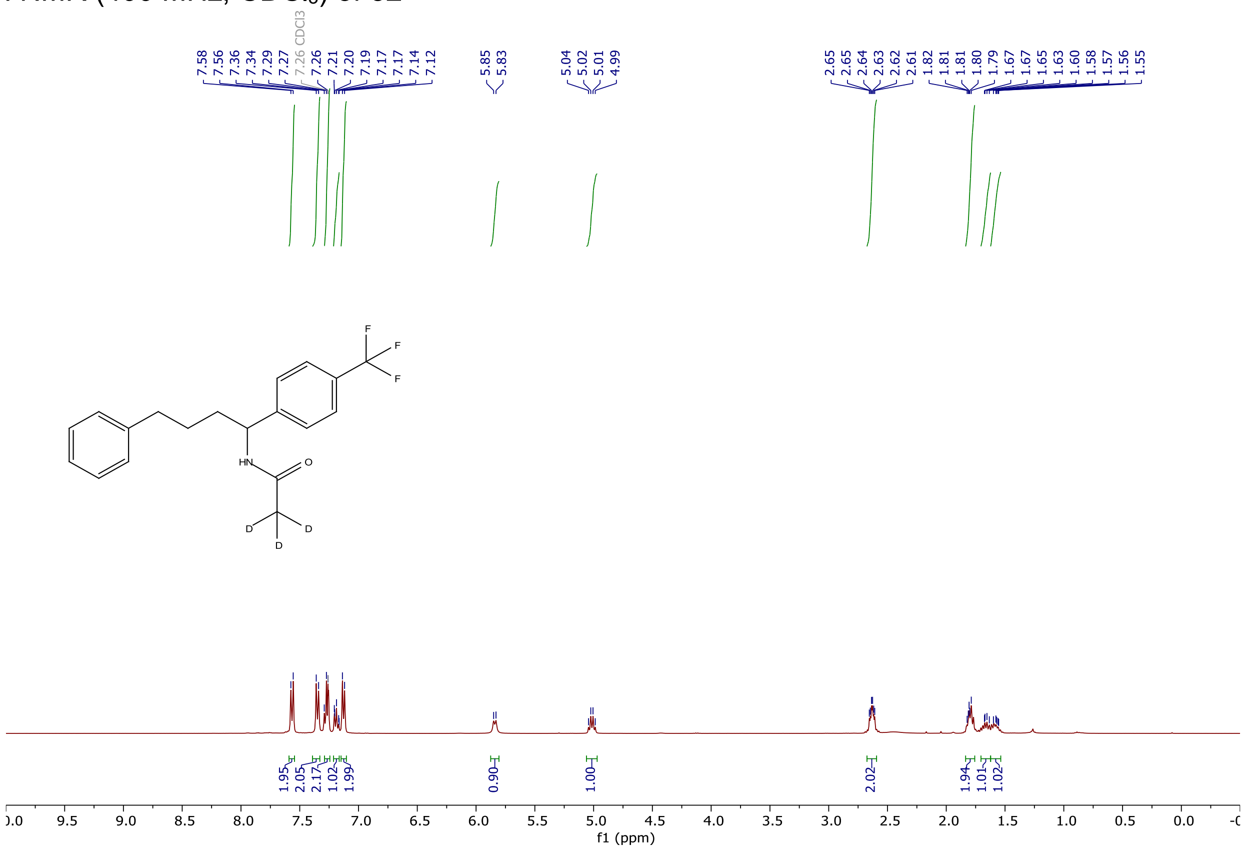

^{13}C NMR (151 MHz, $CDCl_3$) of **32**

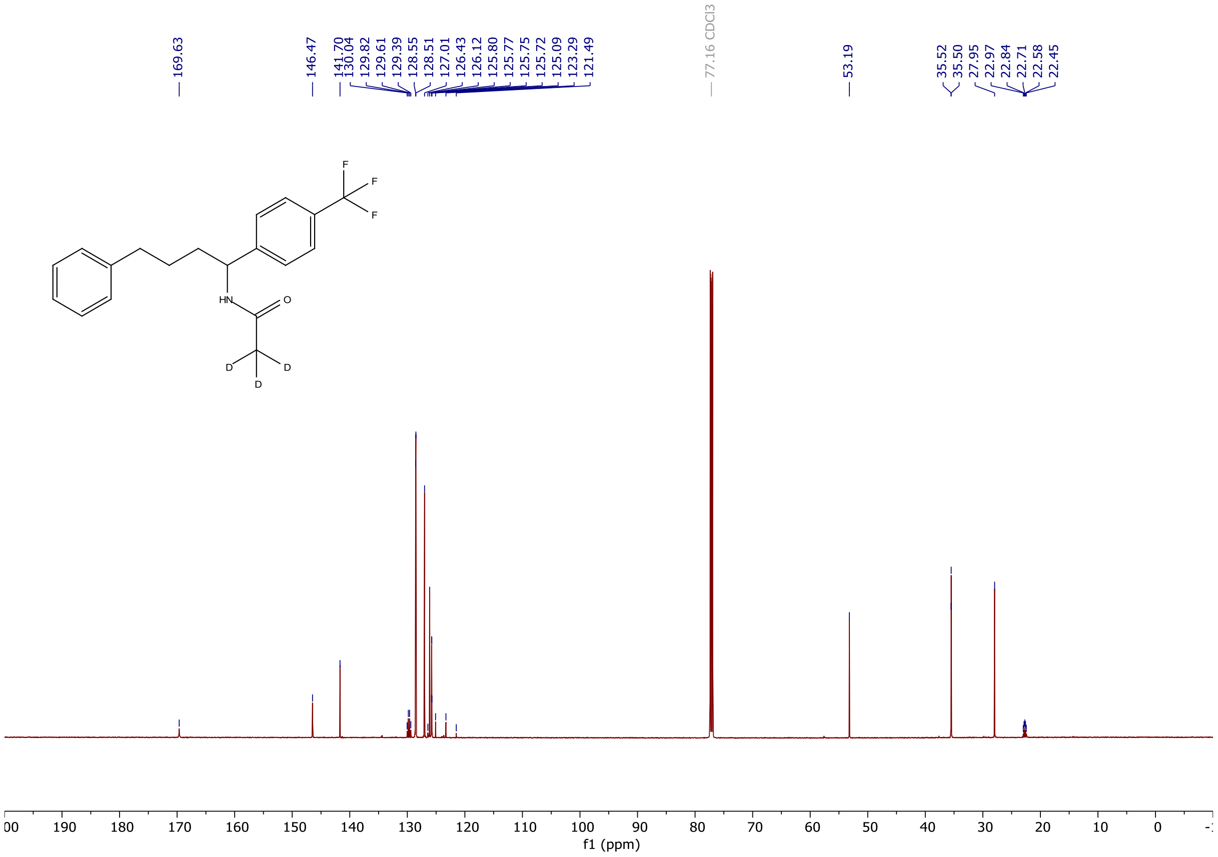

^{19}F NMR (376 MHz, $CDCl_3$) of **32**

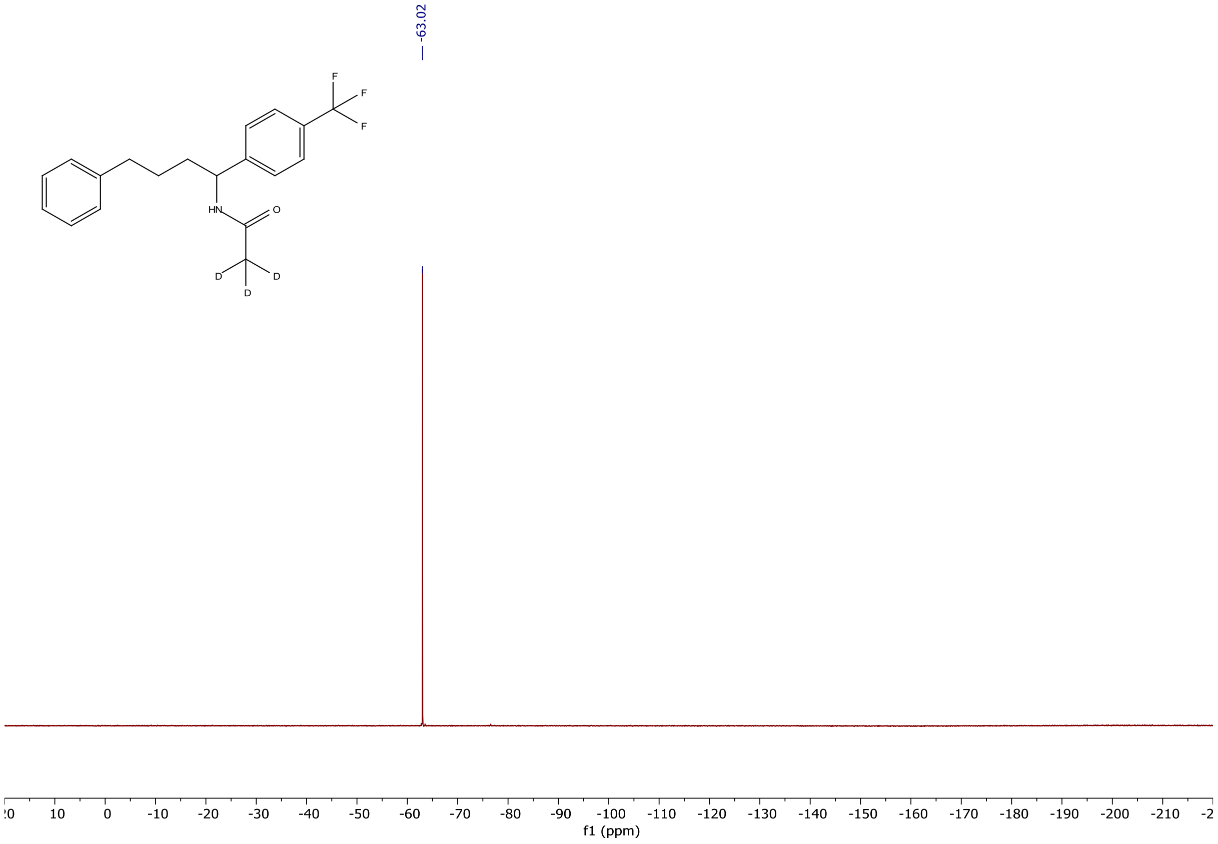

^{1}H NMR (400 MHz, $CDCl_3$) of **33**

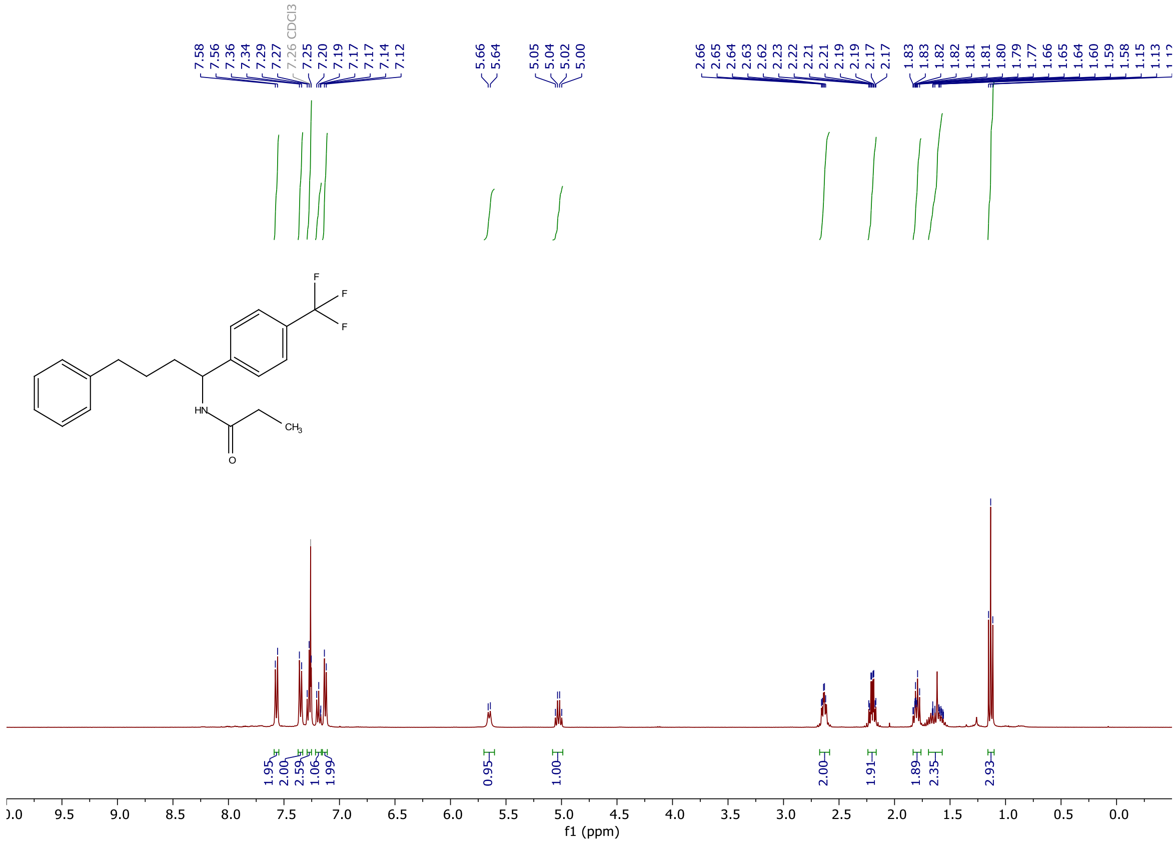

^{13}C NMR (151 MHz, $CDCl_3$) of **33**

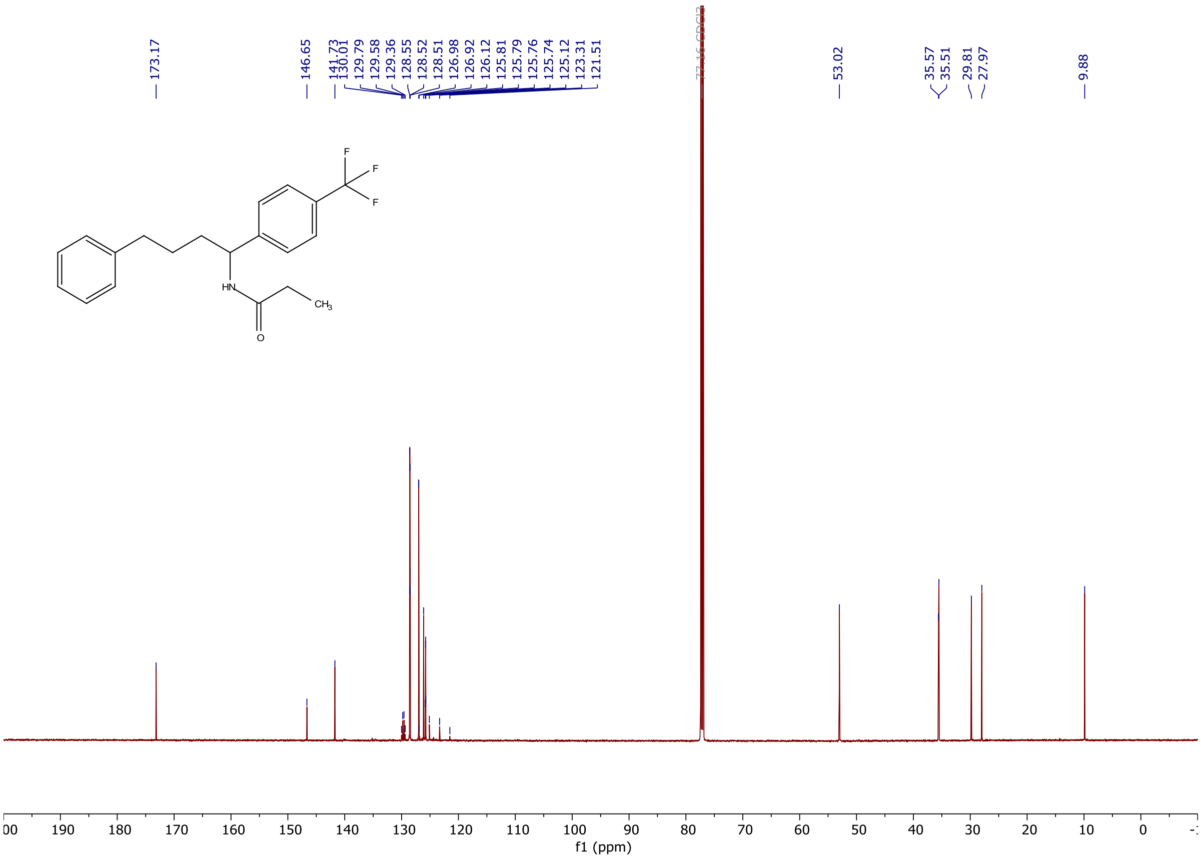

^{19}F NMR (376 MHz, $CDCl_3$) of **33**

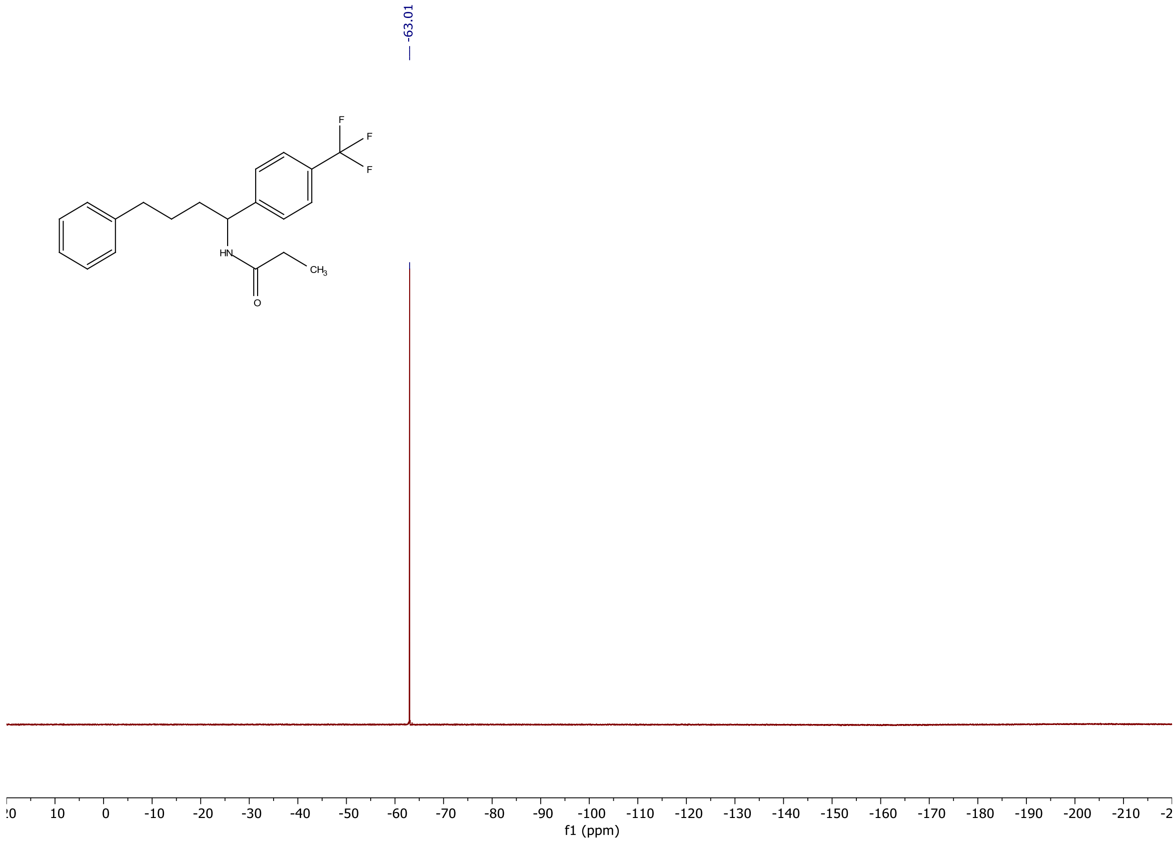

^{1}H NMR (400 MHz, $CDCl_3$) of **34**

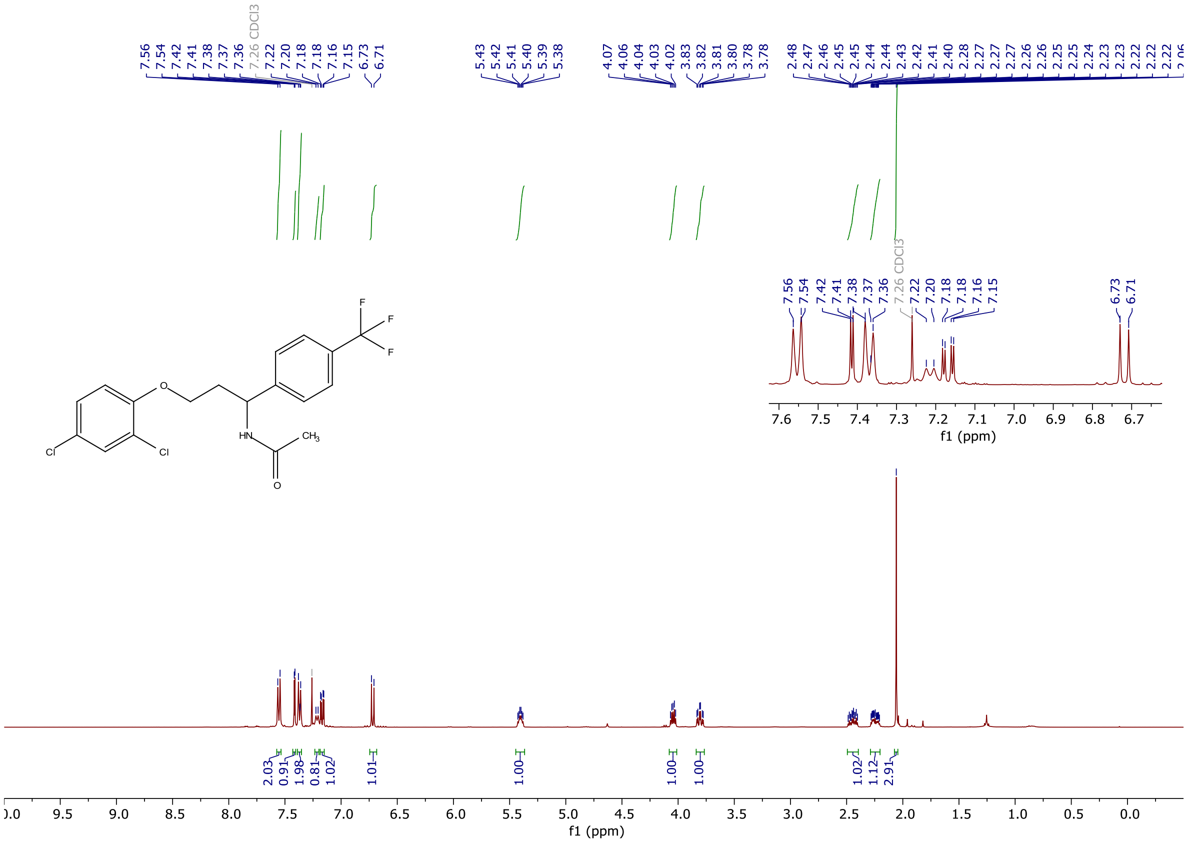

^{13}C NMR (151 MHz, $CDCl_3$) of **34**

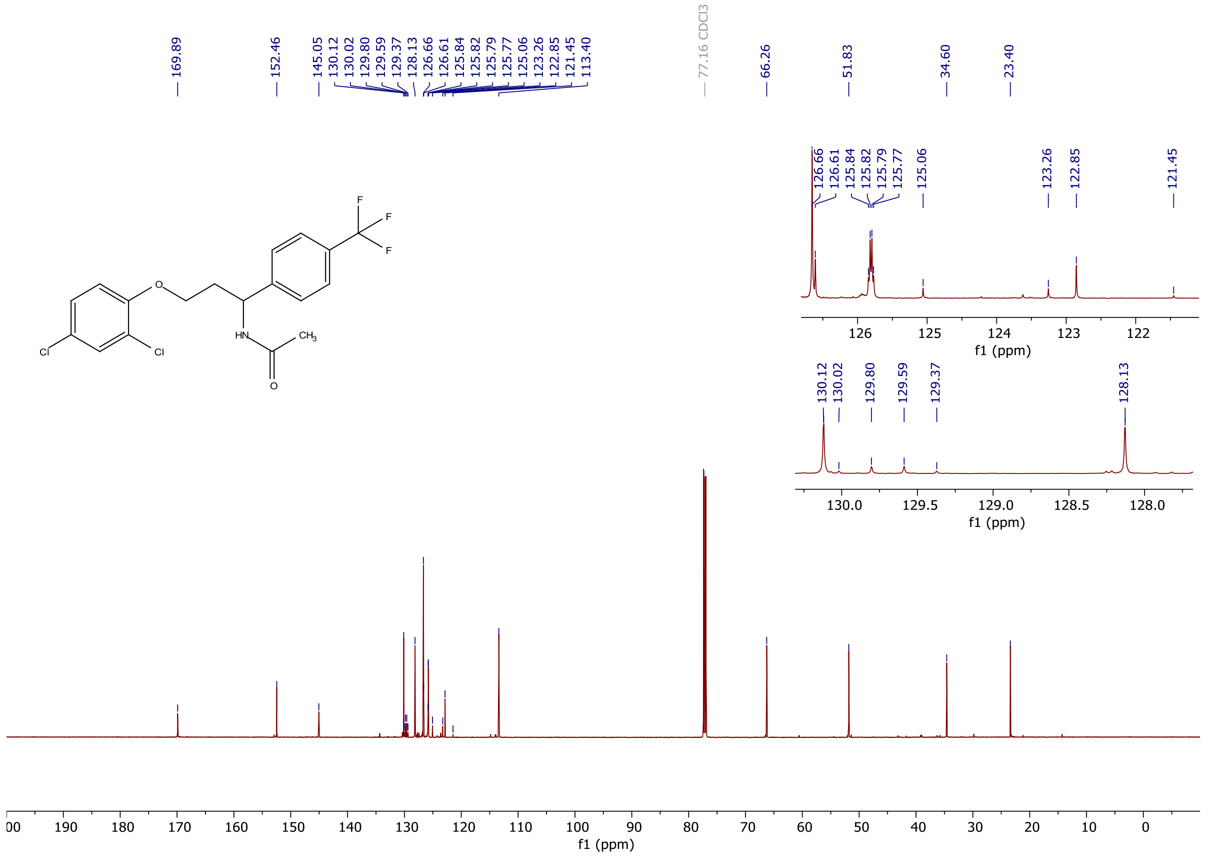

^{19}F NMR (376 MHz, $CDCl_3$) of **34**

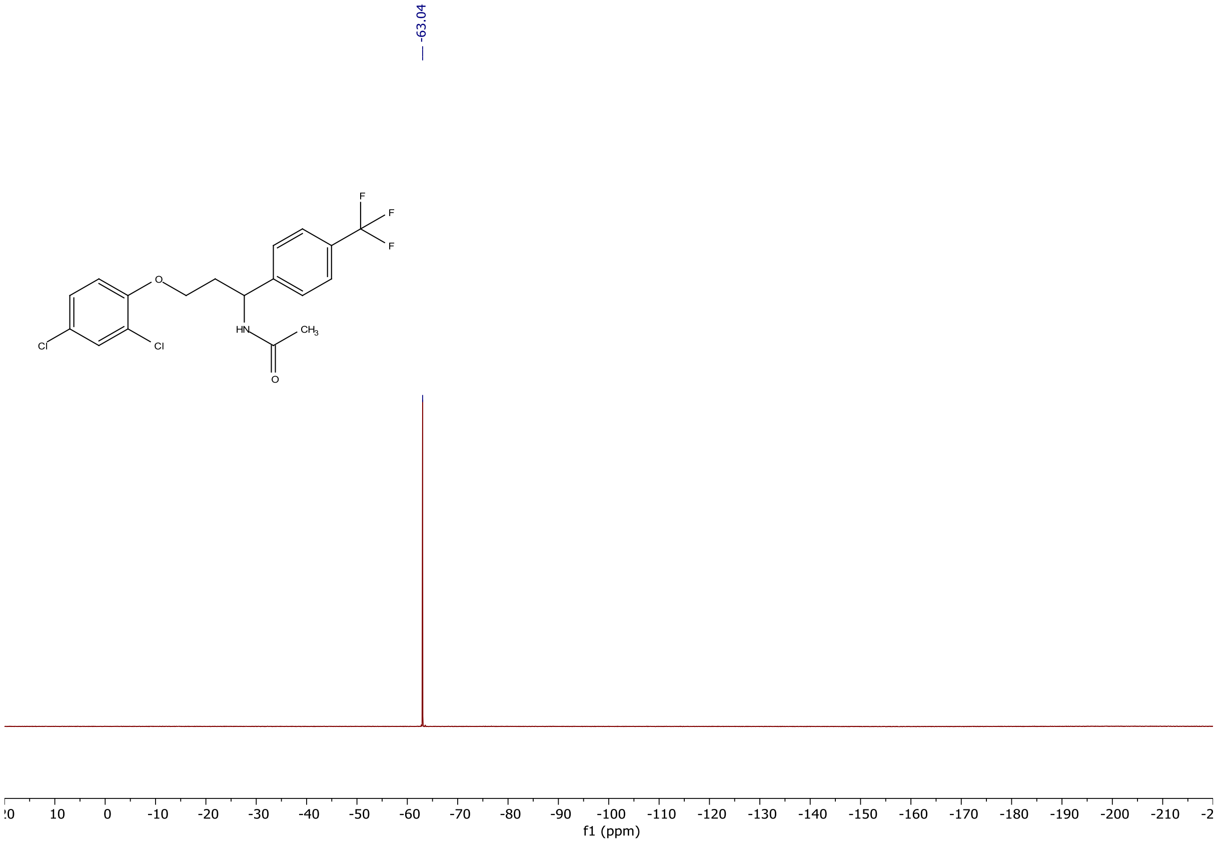

^{1}H NMR (400 MHz, $CDCl_3$) of **35**

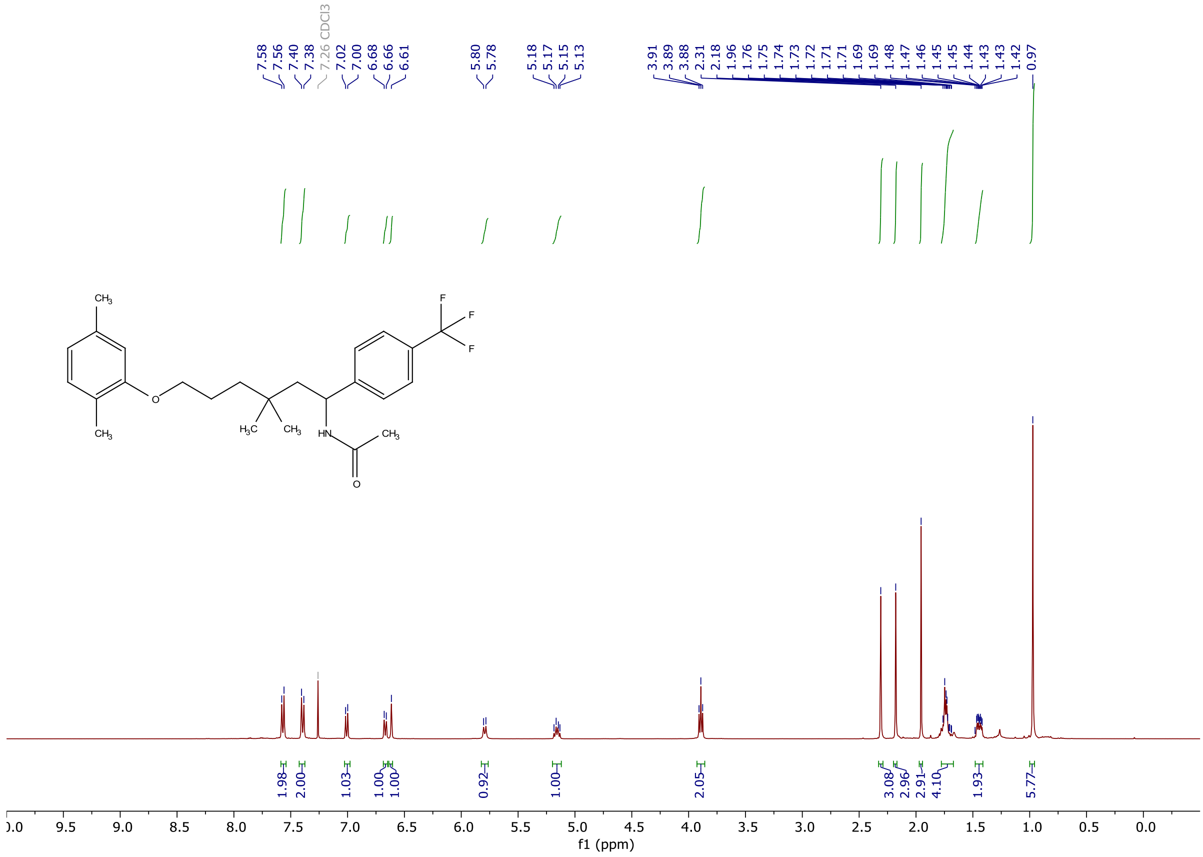

^{13}C NMR (151 MHz, $CDCl_3$) of **35**

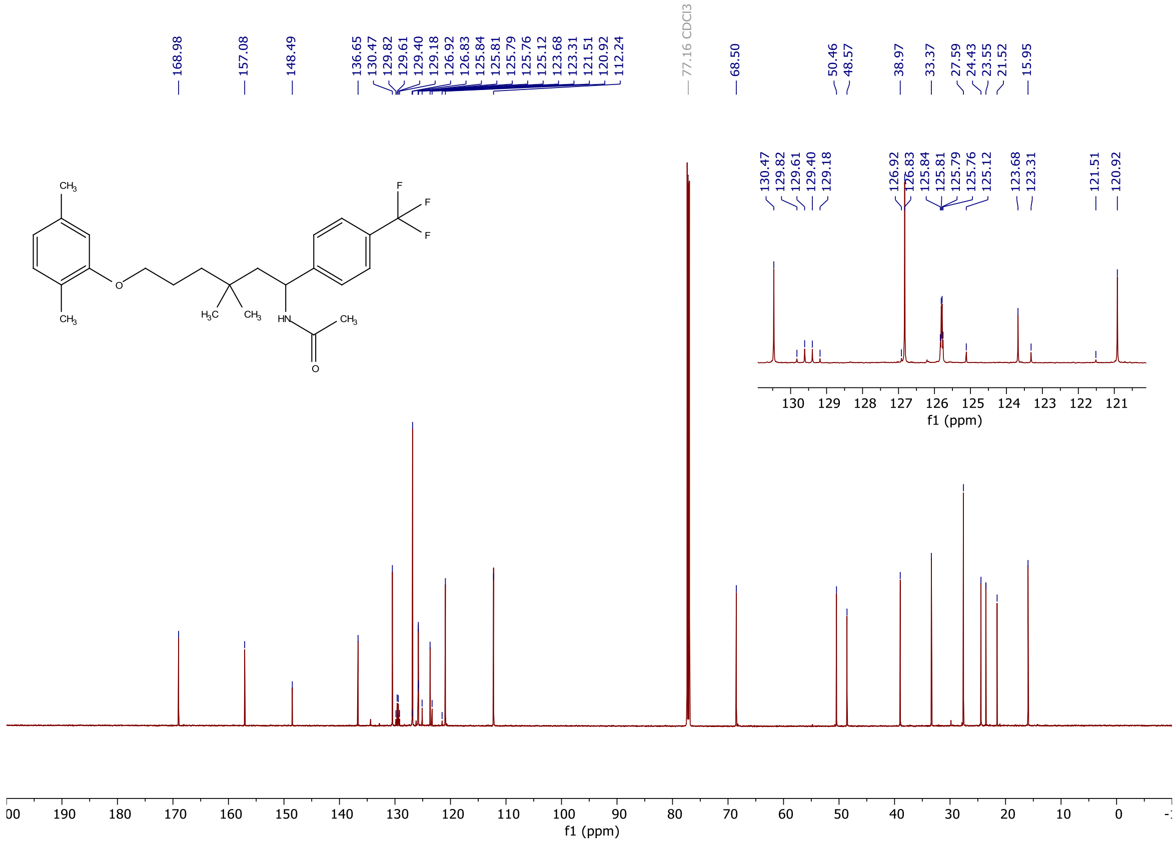

^{19}F NMR (376 MHz, $CDCl_3$) of **35**

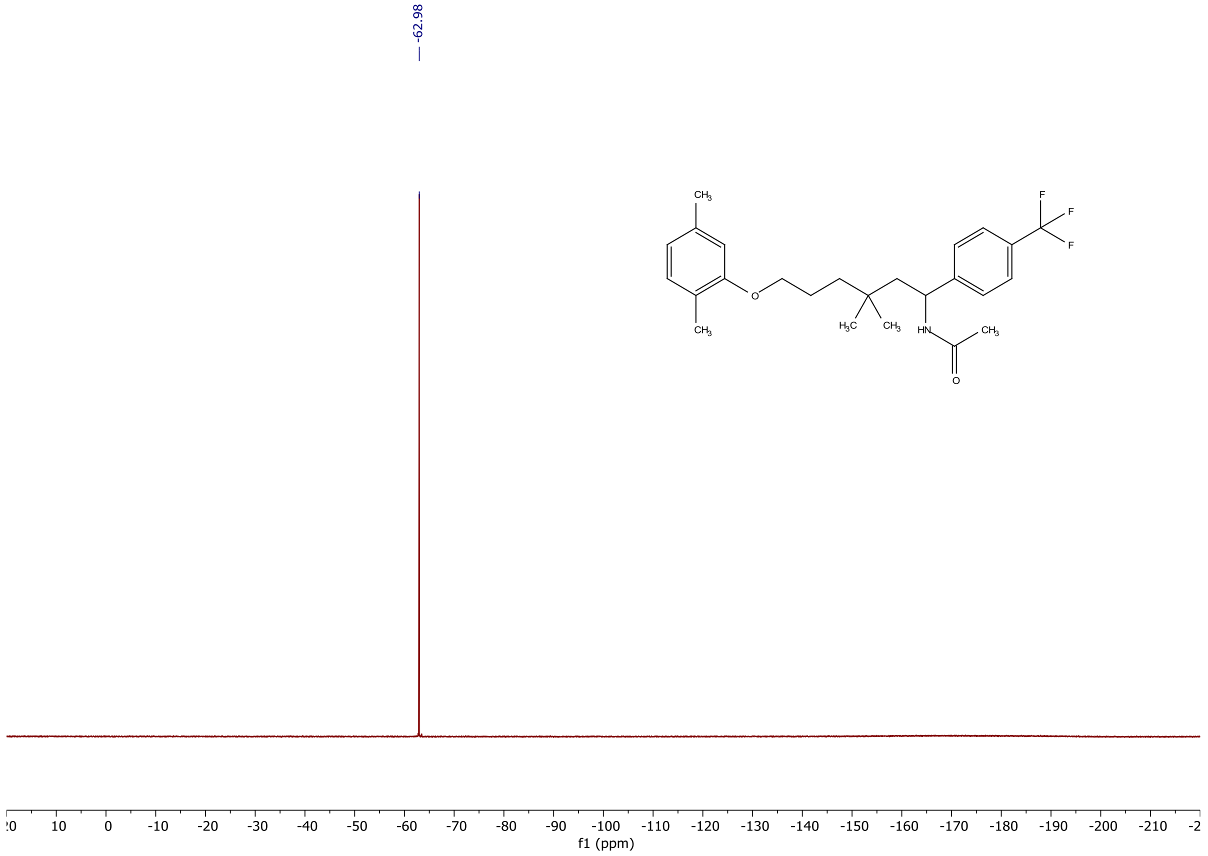

^{1}H NMR (400 MHz, $CDCl_3$) of **36**

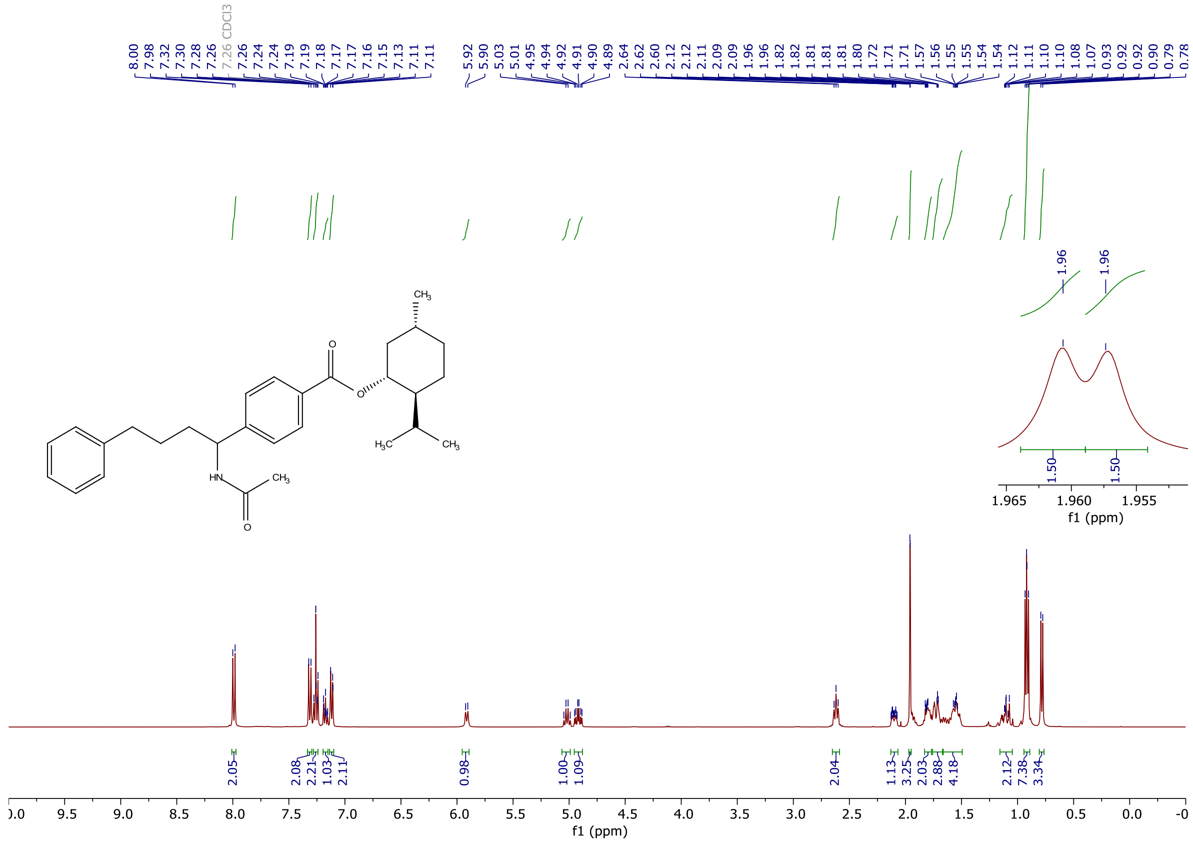

^{13}C NMR (101 MHz, $CDCl_3$) of **36**

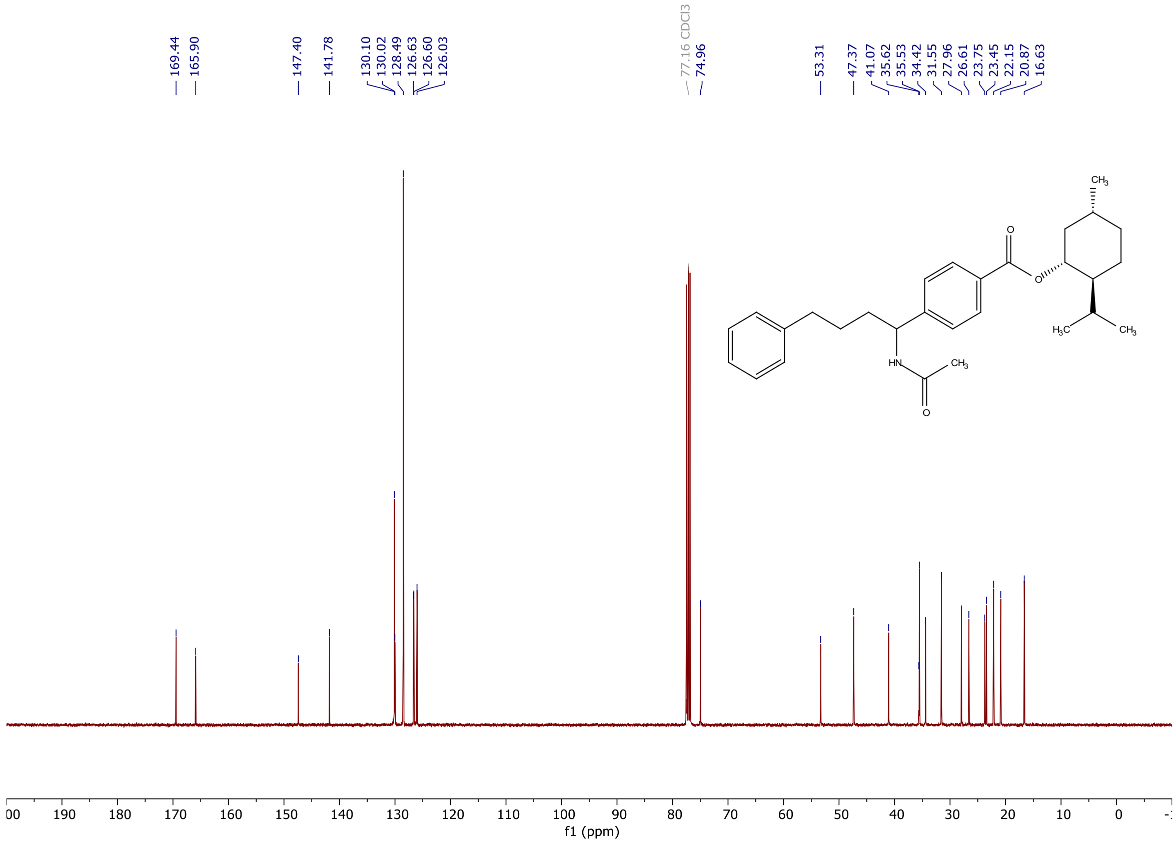

^{1}H NMR (400 MHz, $CDCl_3$) of **37**

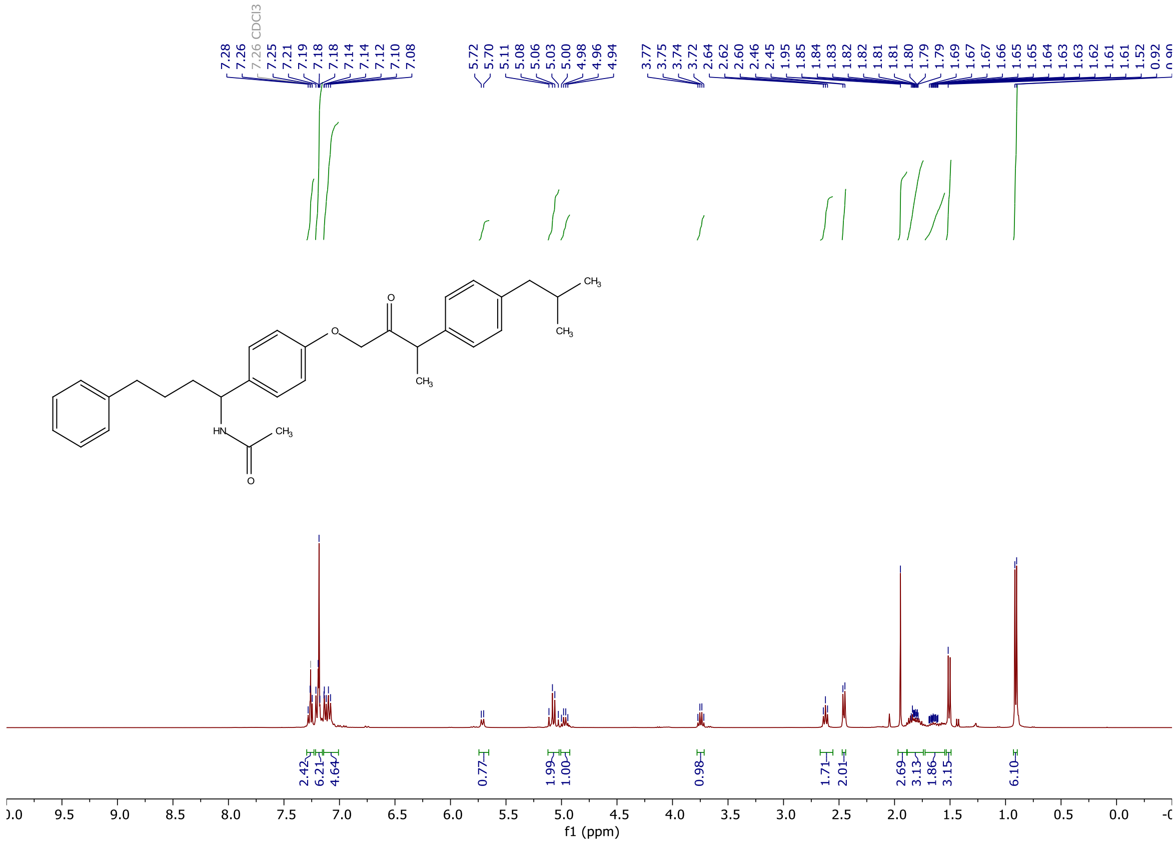

^{13}C NMR (101 MHz, $CDCl_3$) of **37**

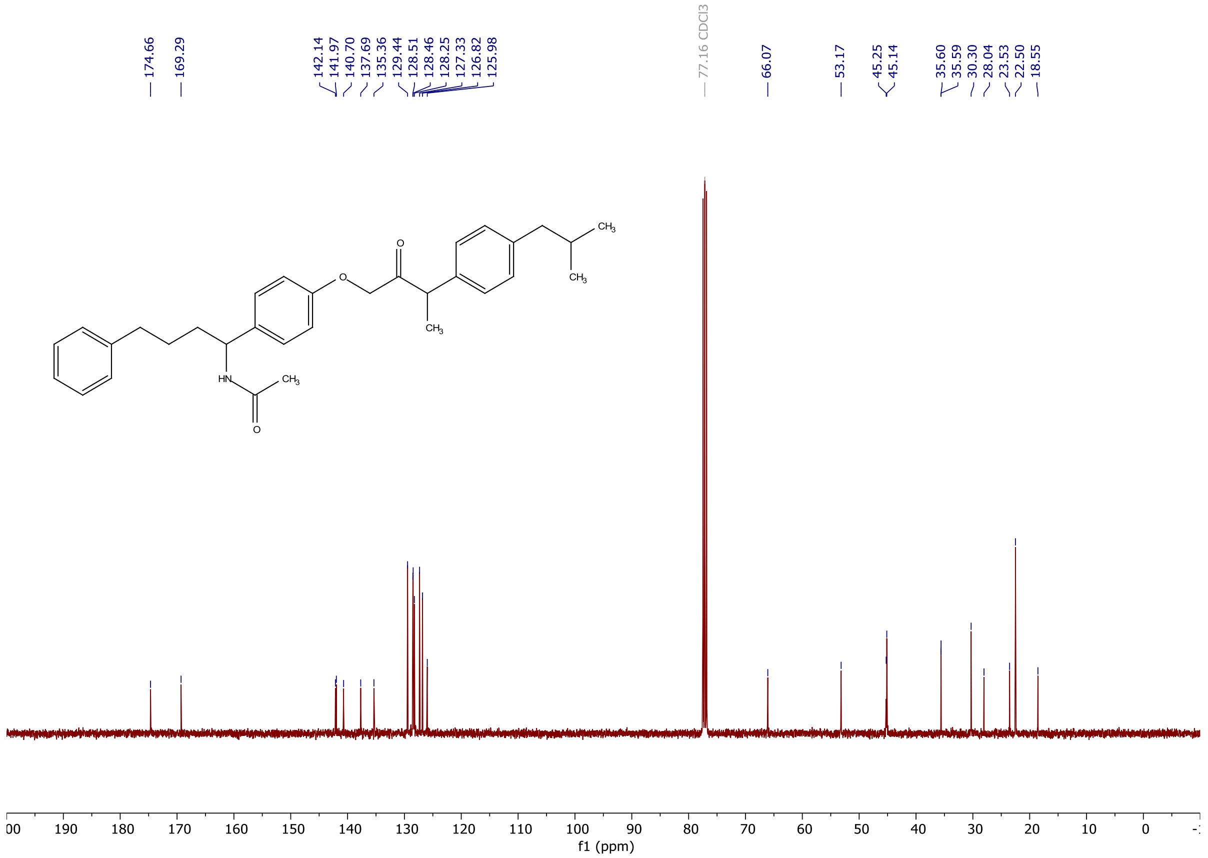

16. NMR spectra of Heck-type products

^{1}H NMR (400 MHz, $CDCl_3$) of **38**

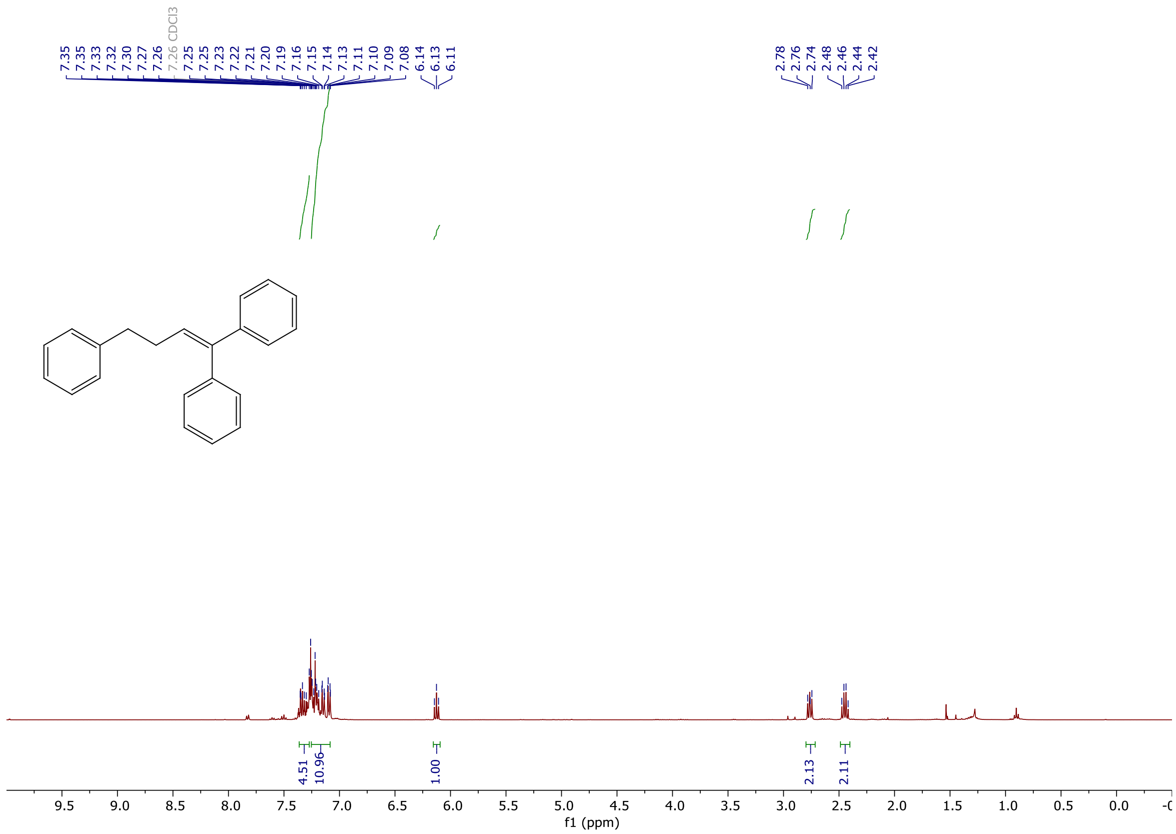

^{13}C NMR (101 MHz, $CDCl_3$) of **38**

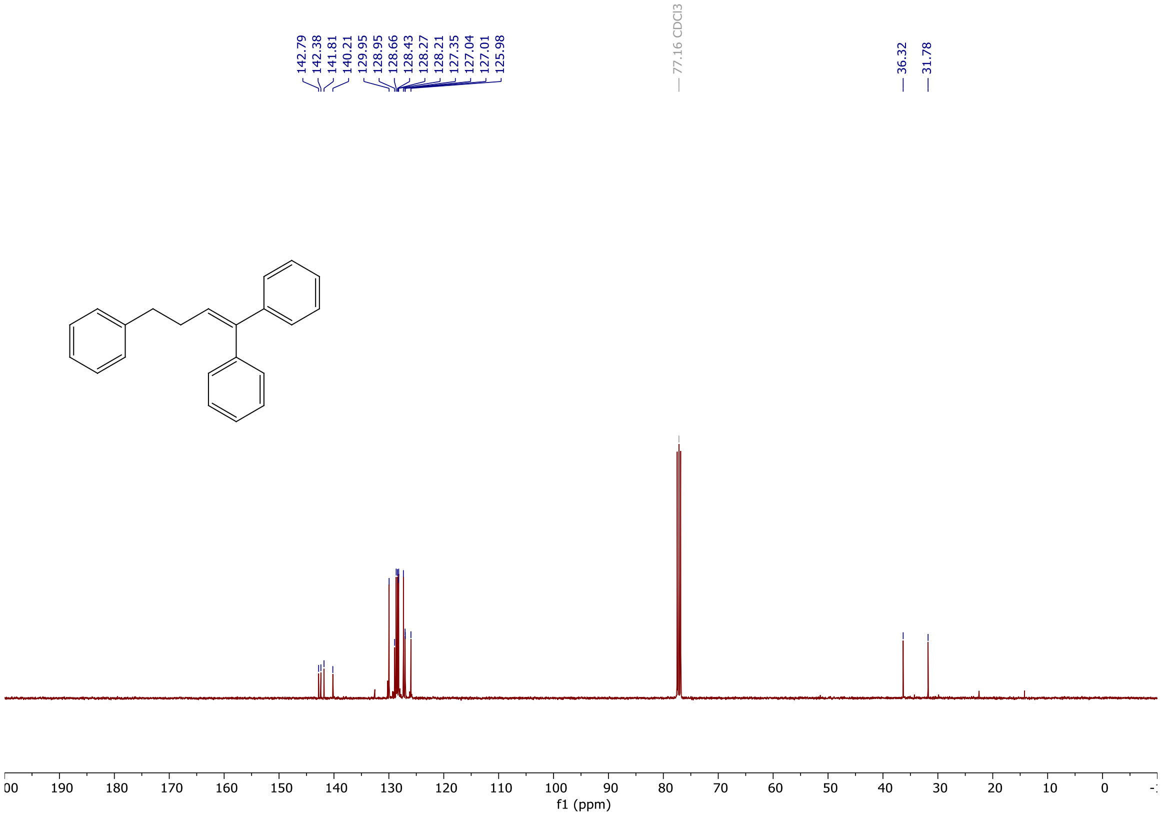

^{1}H NMR (400 MHz, $CDCl_3$) of **39**

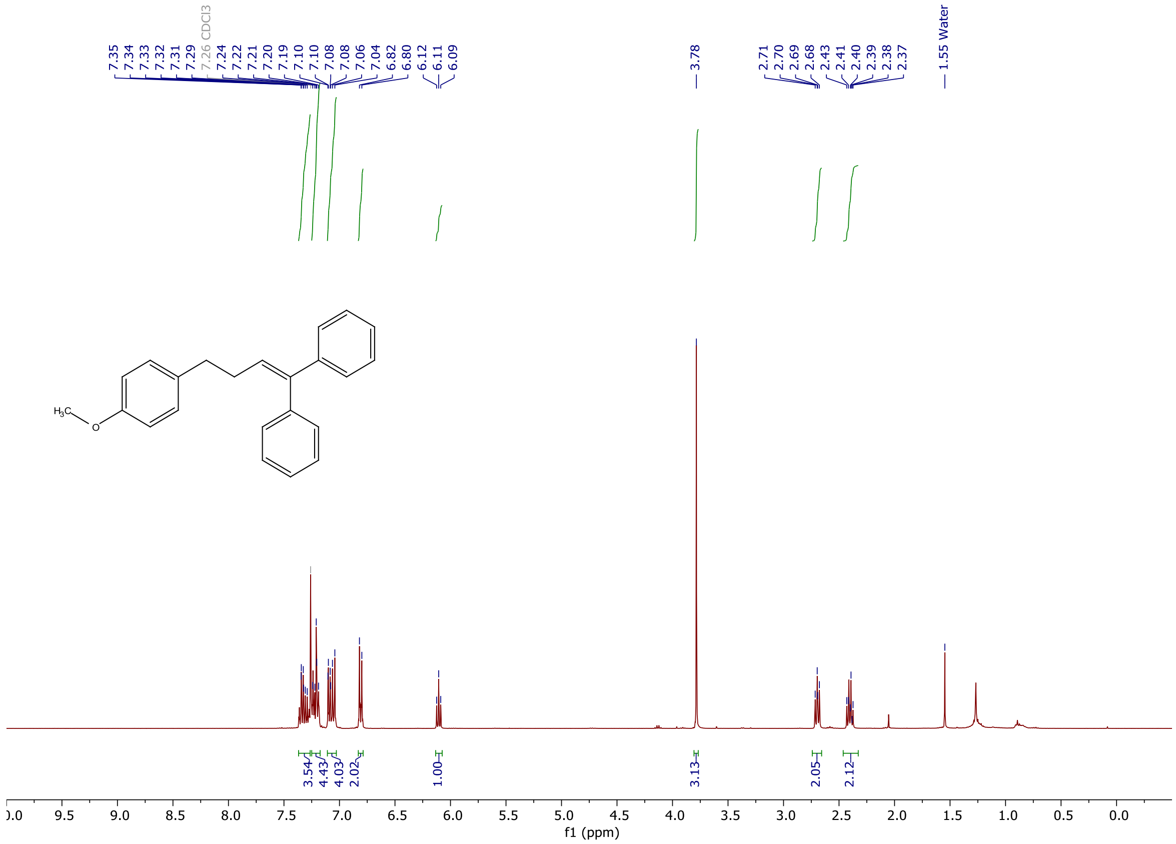

^{13}C NMR (101 MHz, $CDCl_3$) of **39**

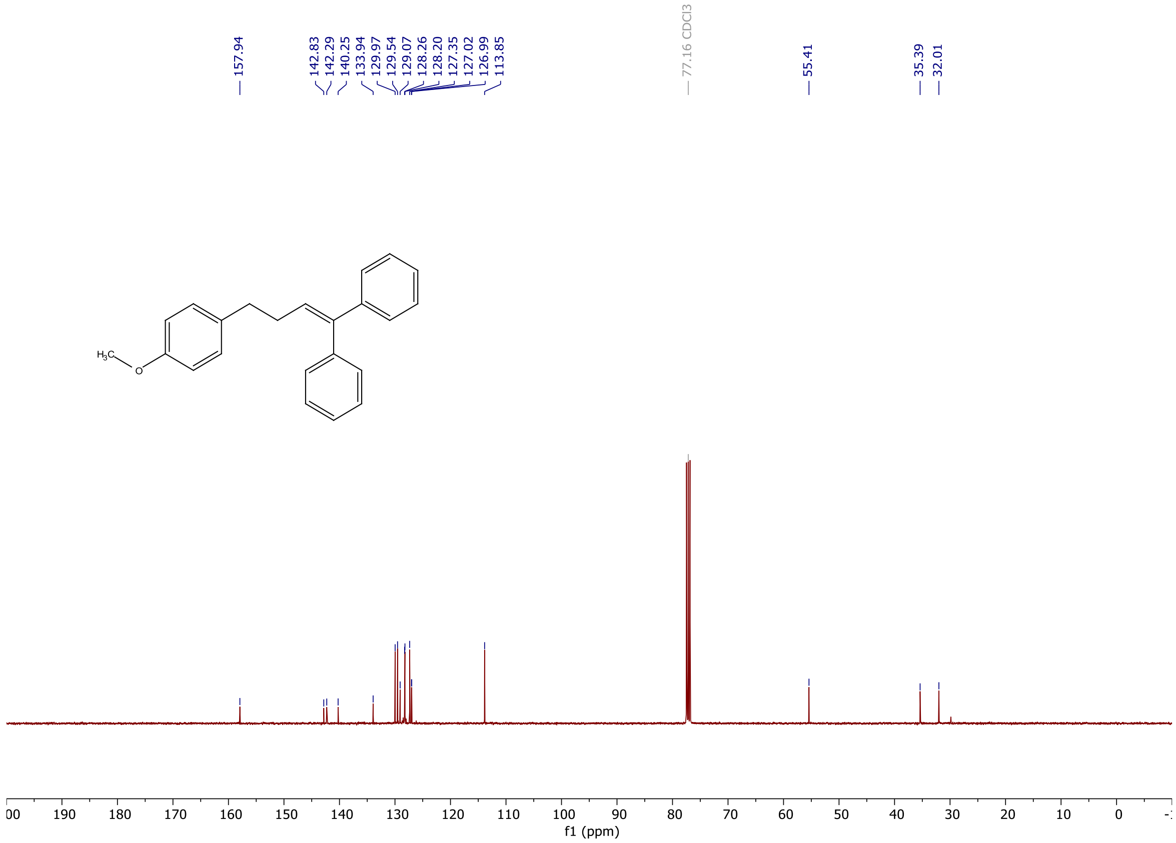

^{1}H NMR (400 MHz, $CDCl_3$) of **40**

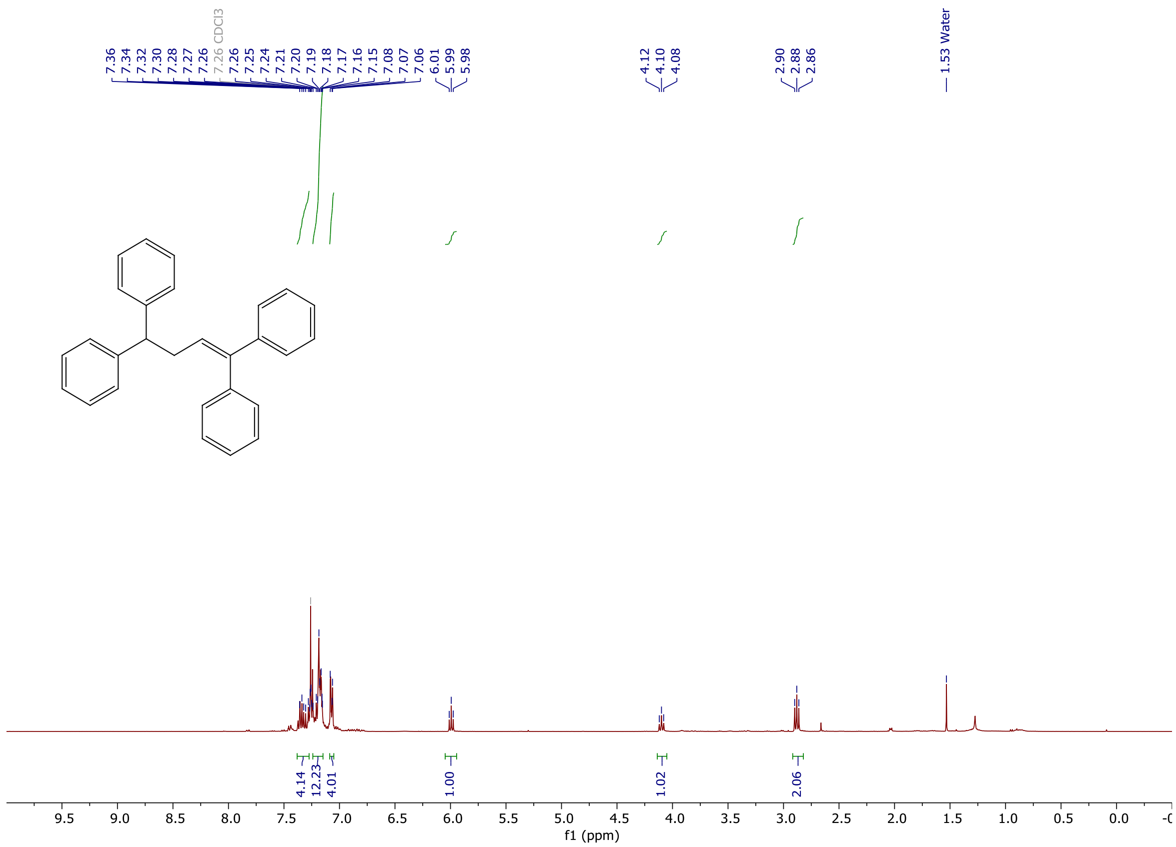

^{13}C NMR (101 MHz, $CDCl_3$) of **40**

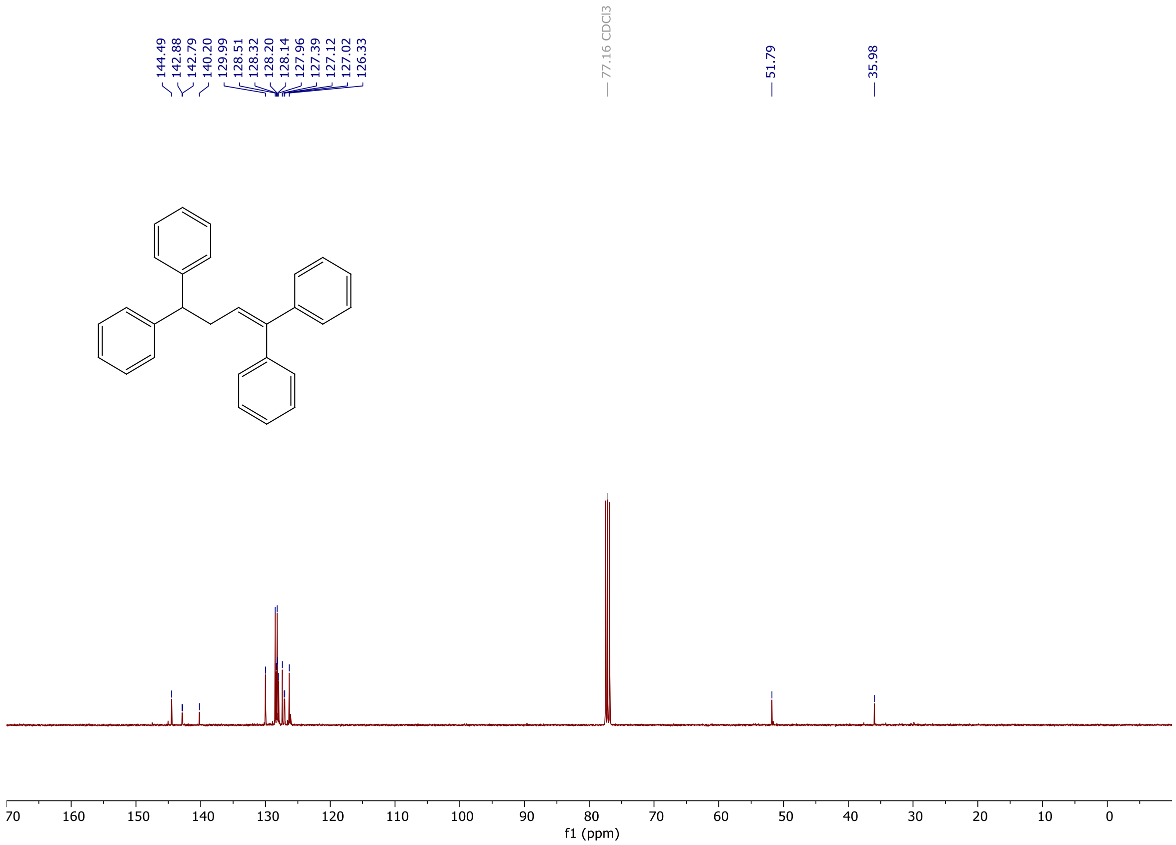

^{1}H NMR (400 MHz, $CDCl_3$) of **41**

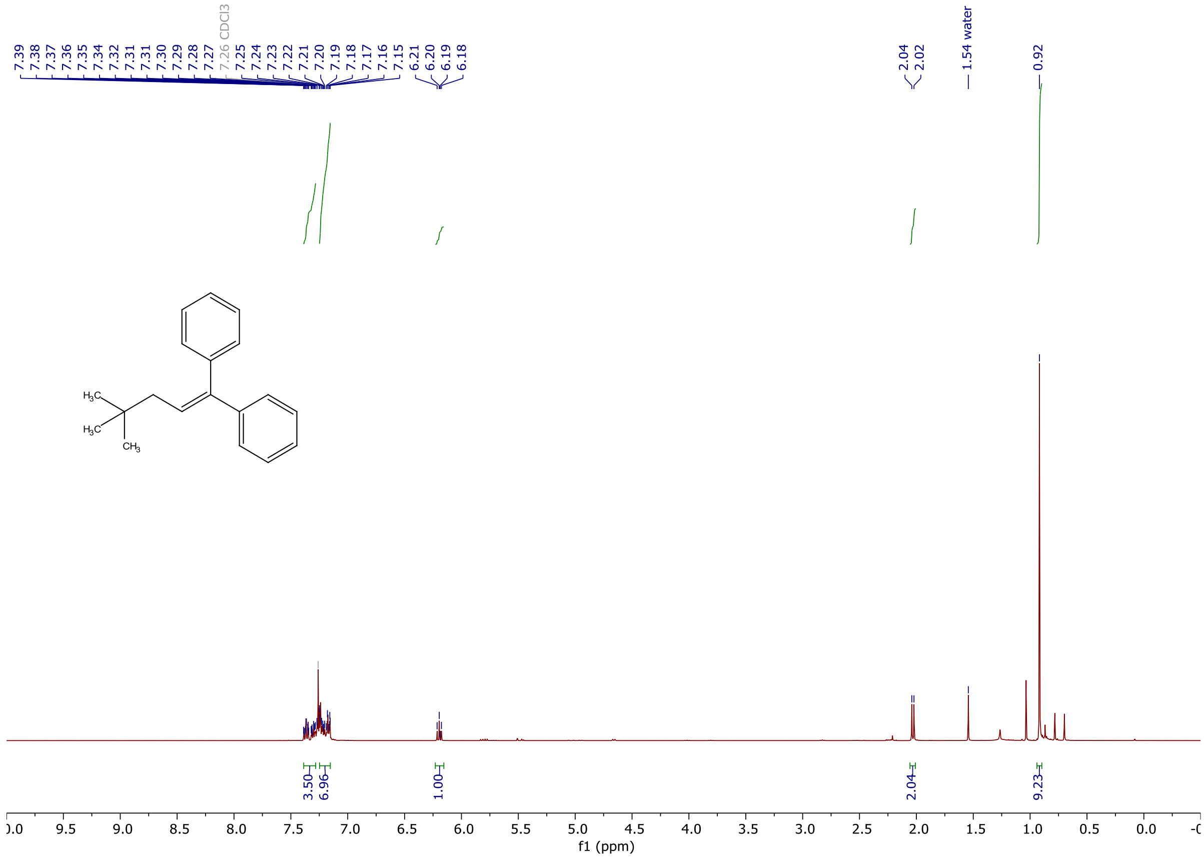

^{13}C NMR (101 MHz, $CDCl_3$) of **41**

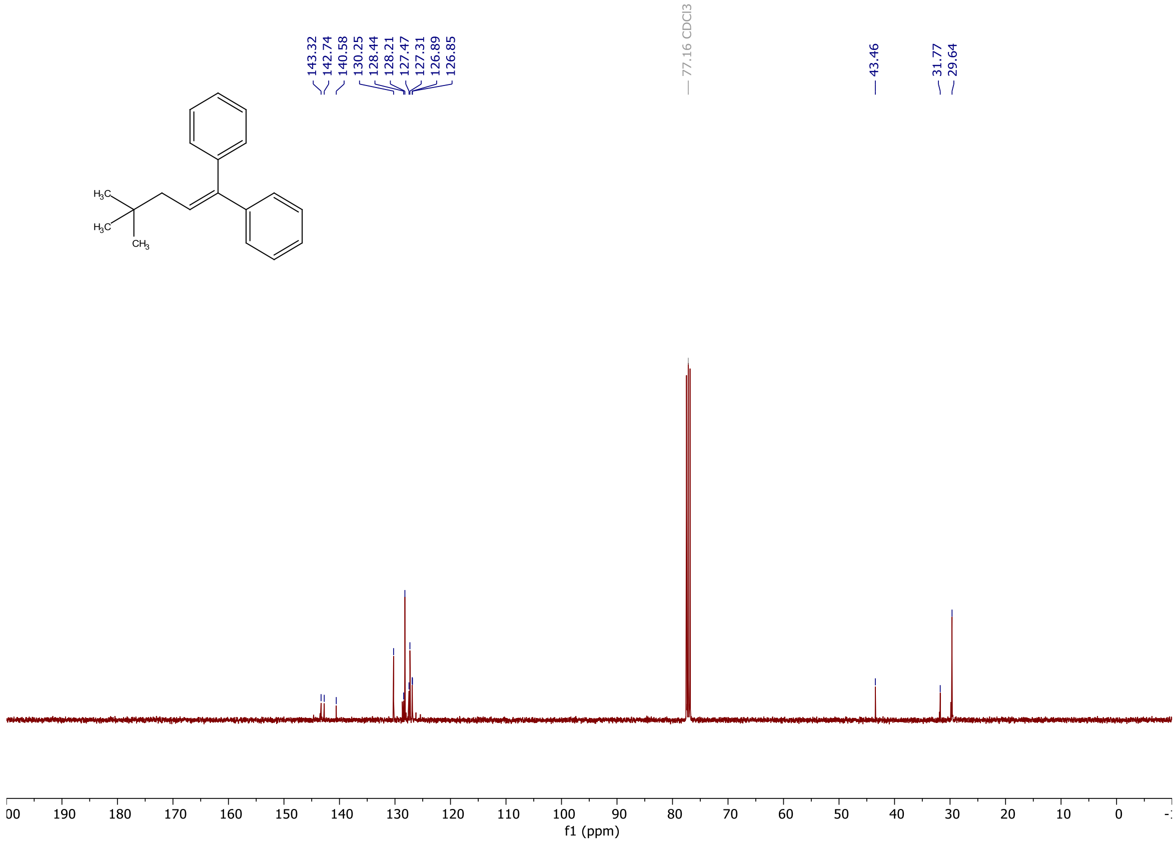

^{1}H NMR (300 MHz, $CDCl_3$) of **42**

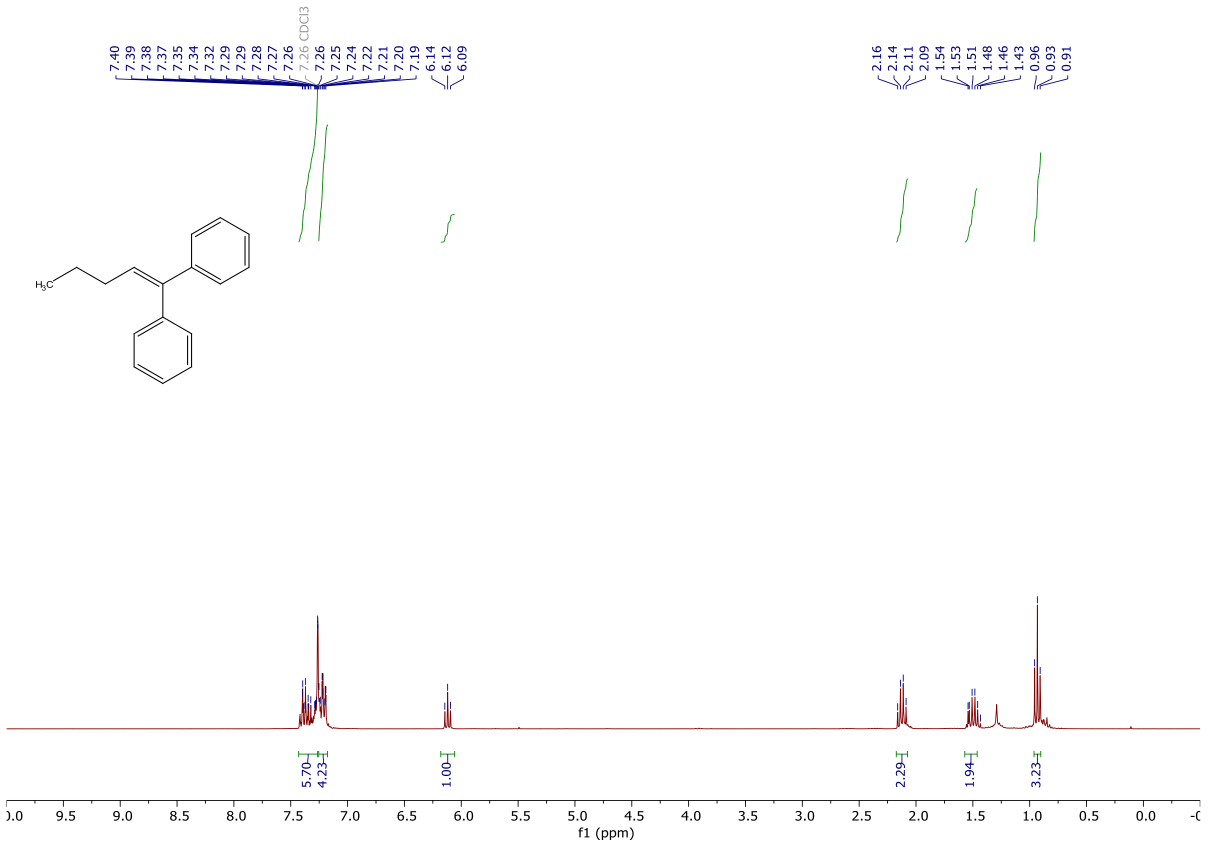

^{13}C NMR (101 MHz, $CDCl_3$) of **42**

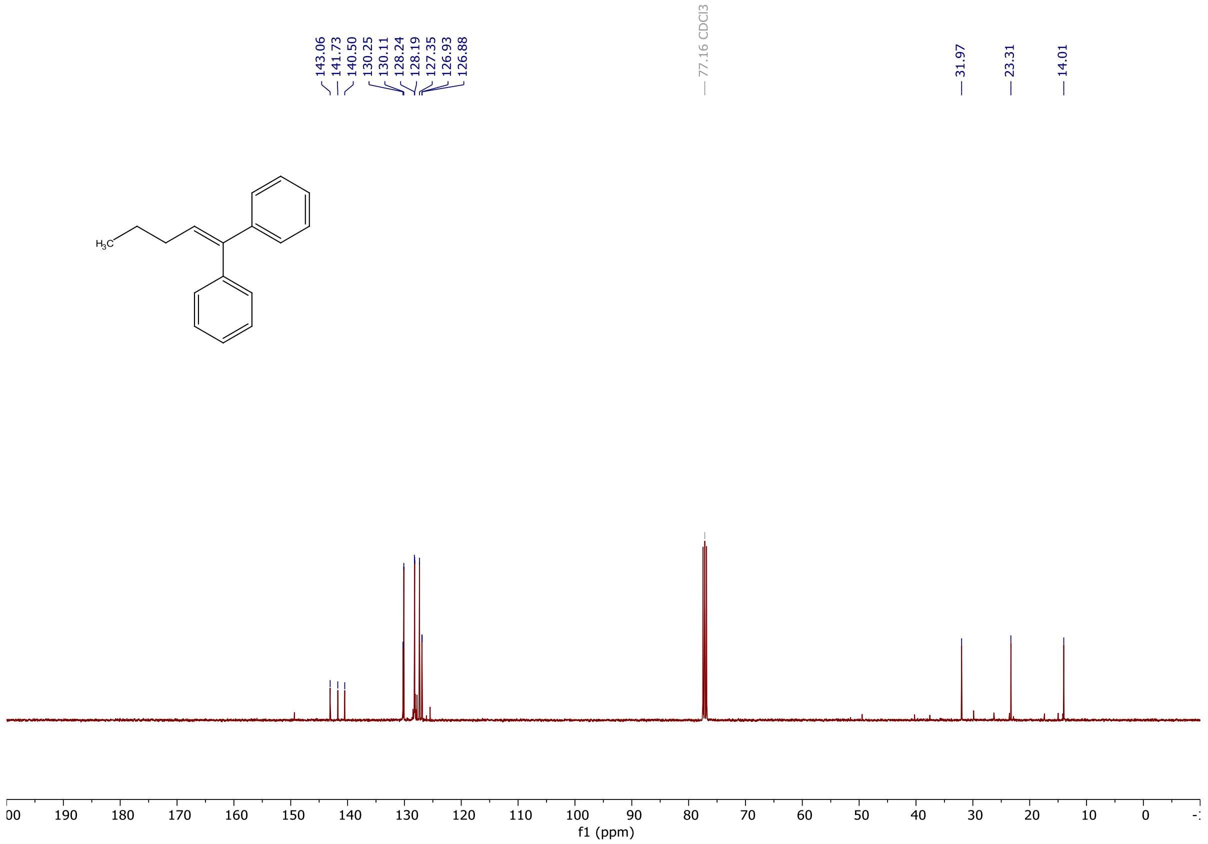

^{1}H NMR (400 MHz, $CDCl_3$) of **43**

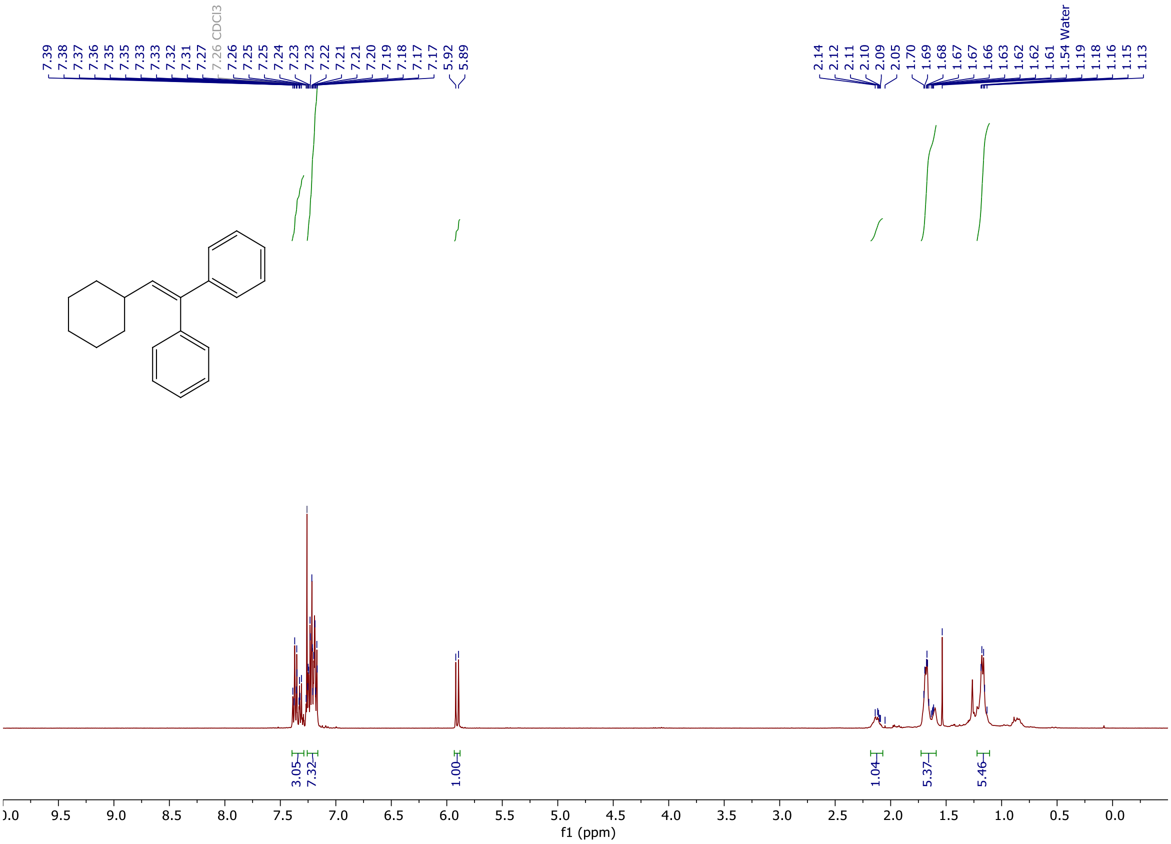

^{13}C NMR (101 MHz, $CDCl_3$) of **43**

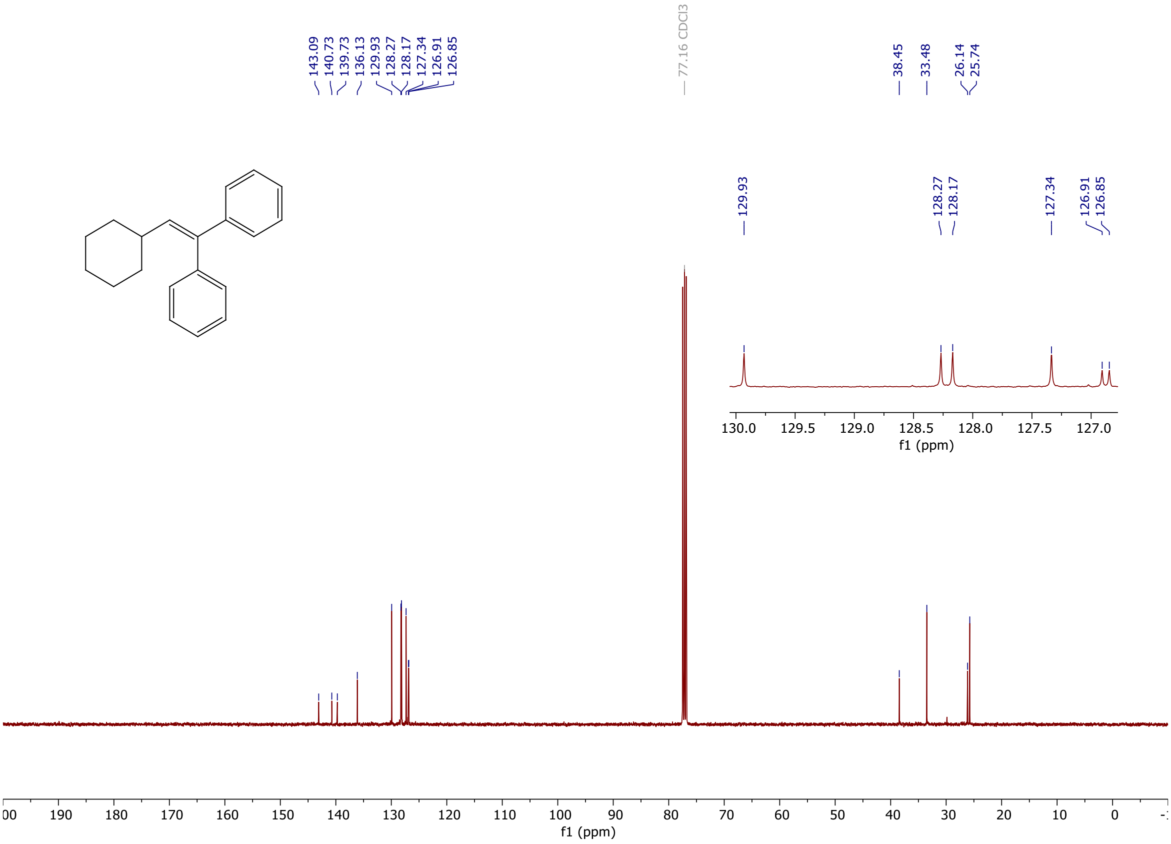

^{1}H NMR (300 MHz, $CDCl_3$) of **44**

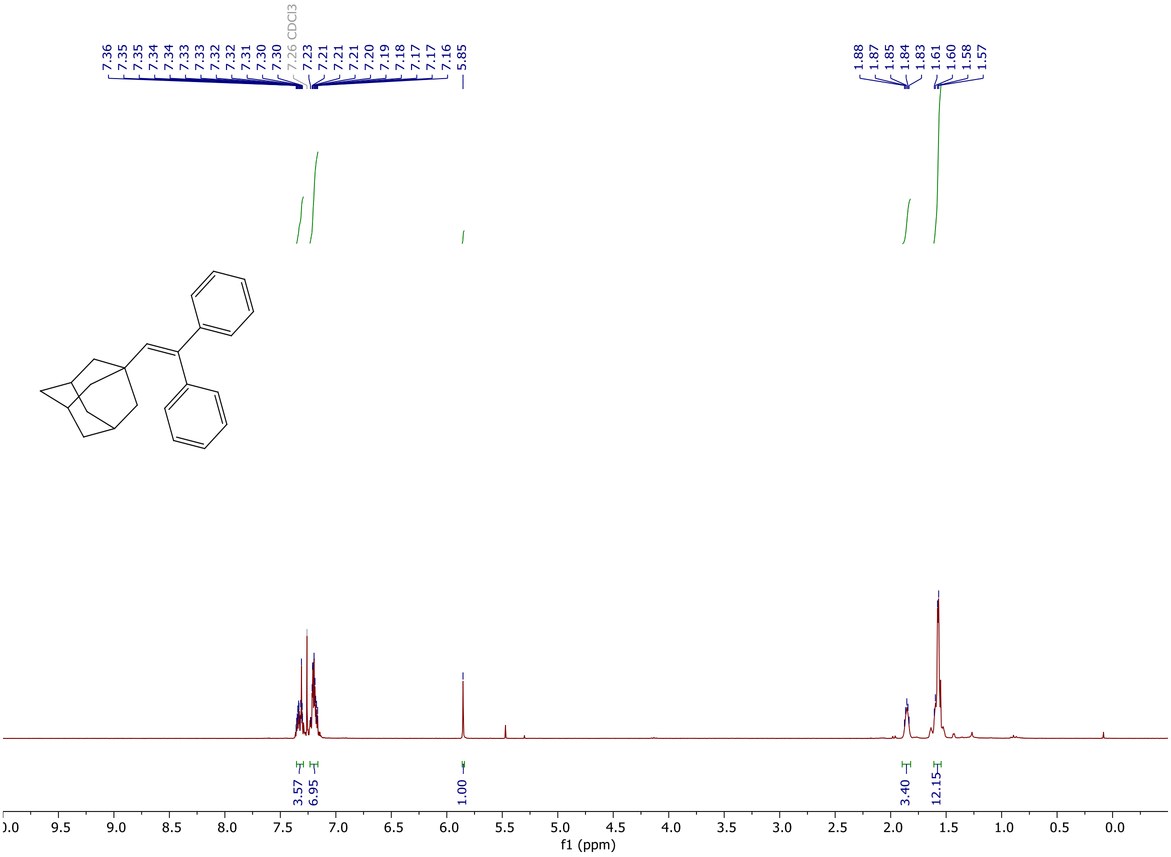

^{13}C NMR (75 MHz, $CDCl_3$) of **44**

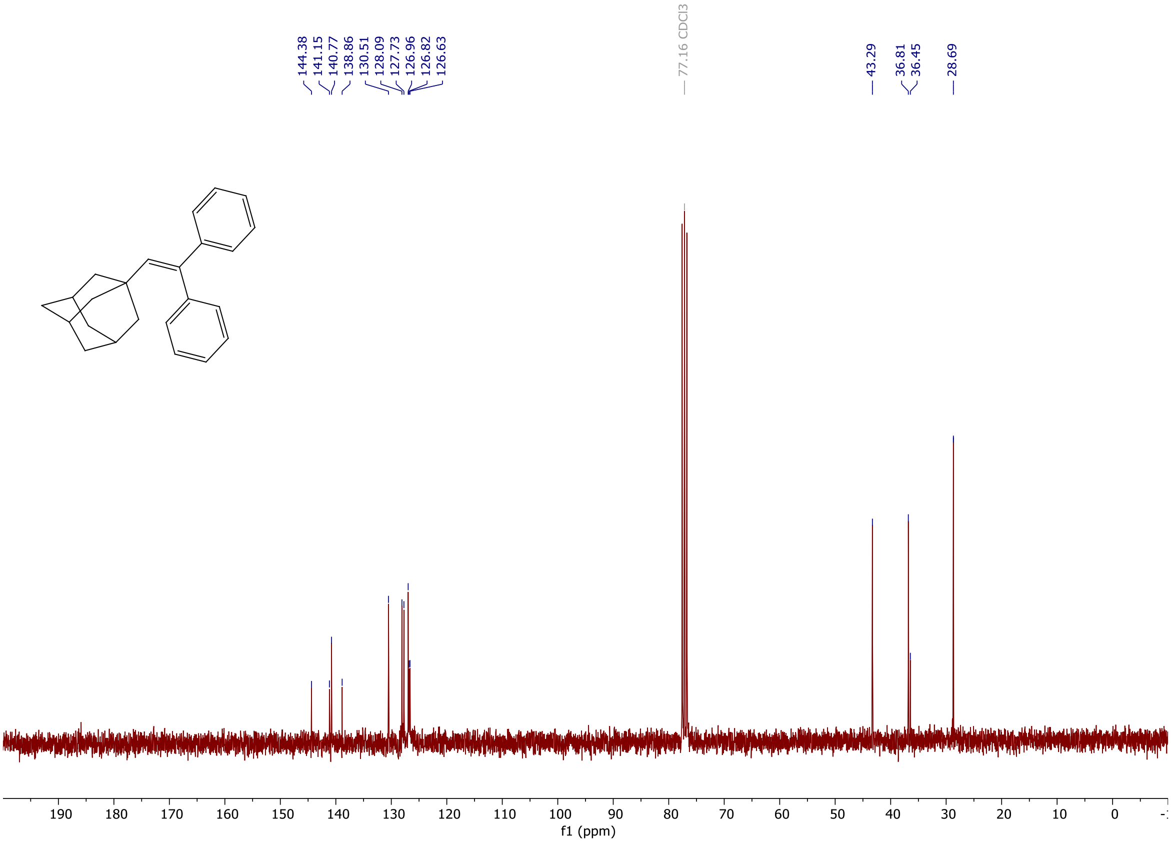

^{1}H NMR (400 MHz, $CDCl_3$) of **45**

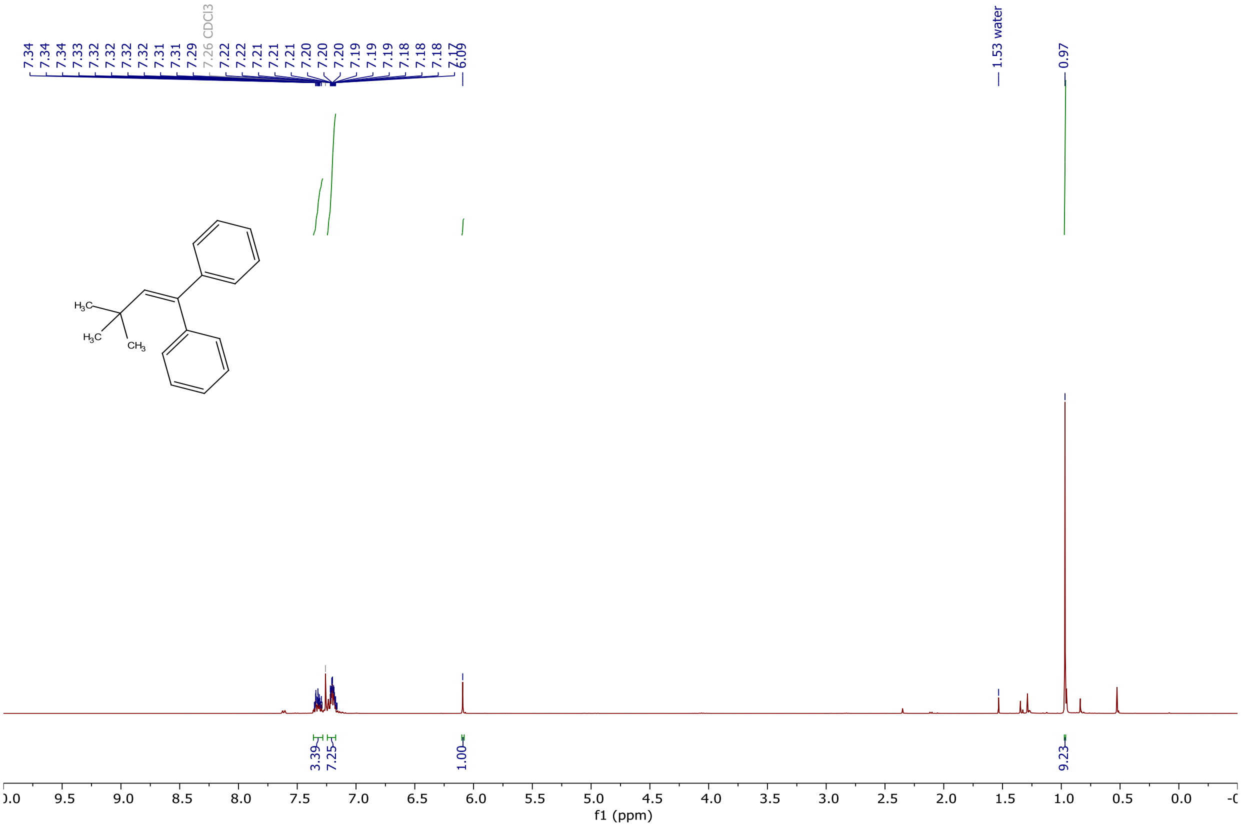

^{13}C NMR (101 MHz, $CDCl_3$) of **45**

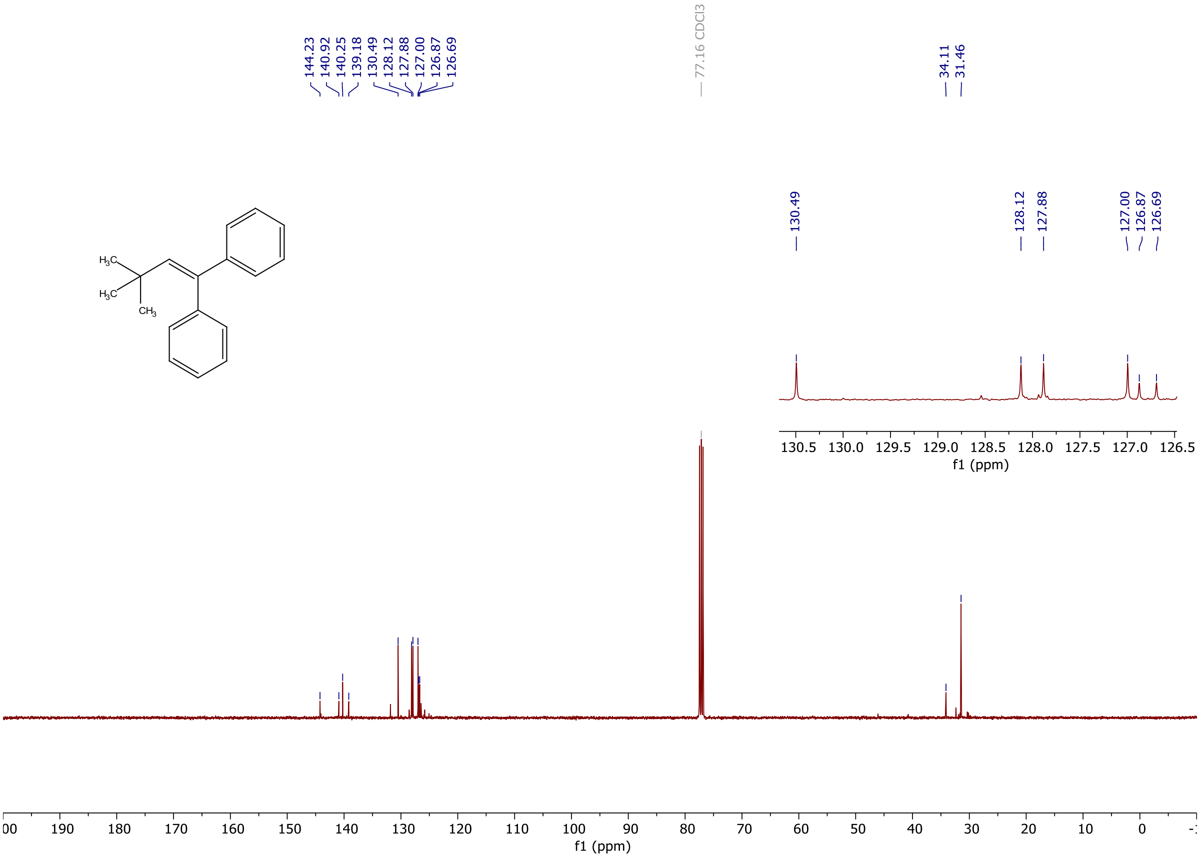

^{1}H NMR (400 MHz, $CDCl_3$) of **46**

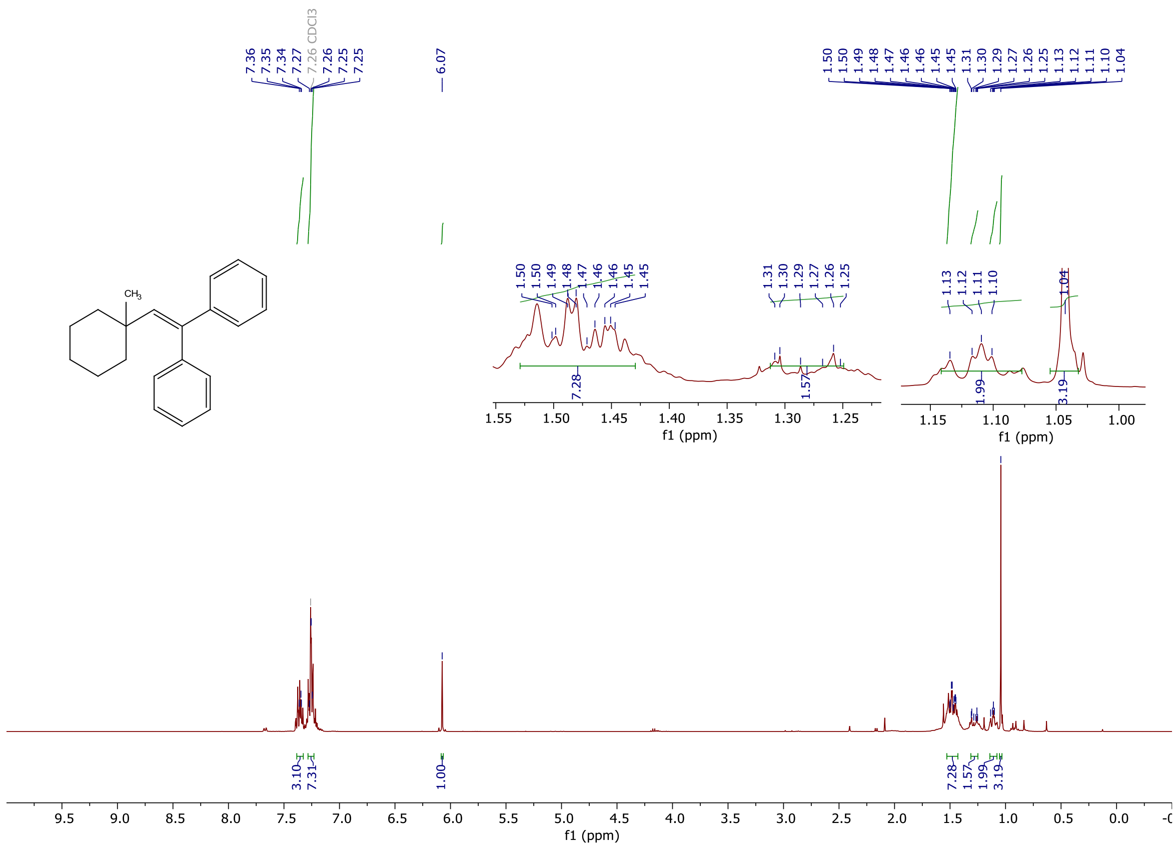

^{13}C NMR (101 MHz, $CDCl_3$) of **46**

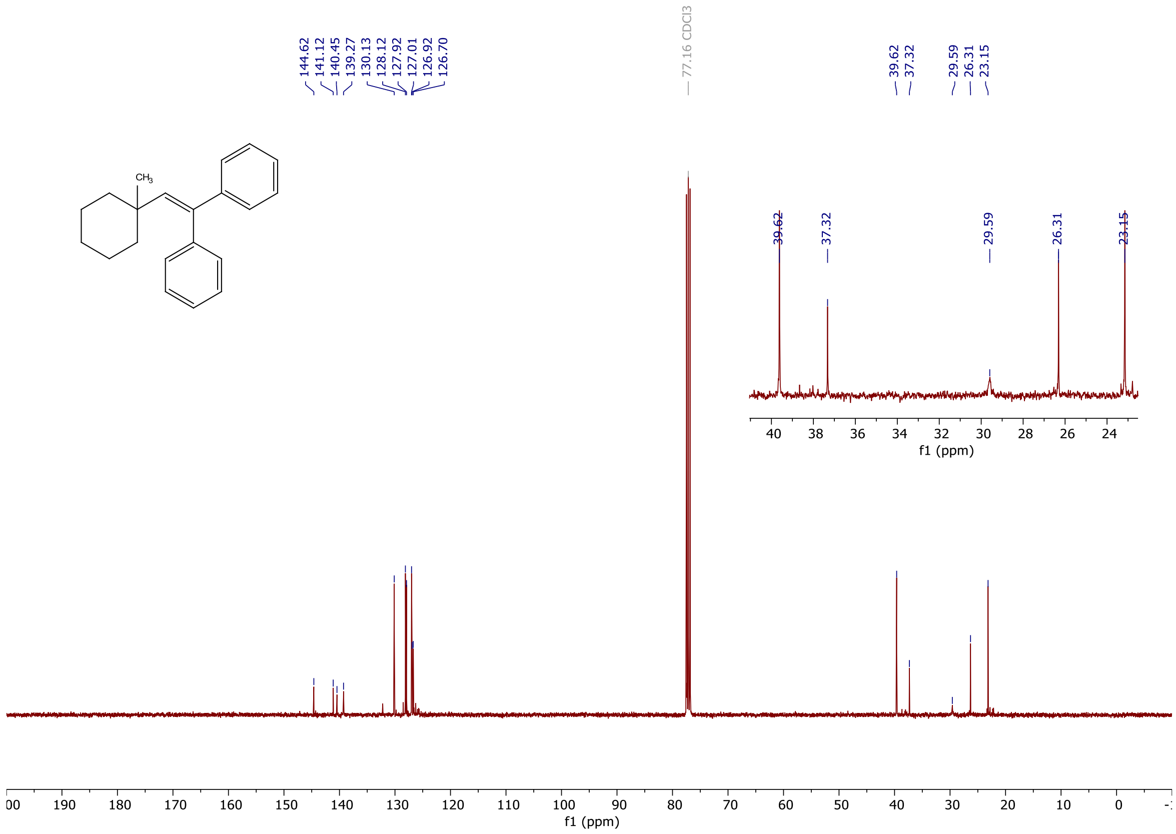

^{1}H NMR (400 MHz, $CDCl_3$) of **47**

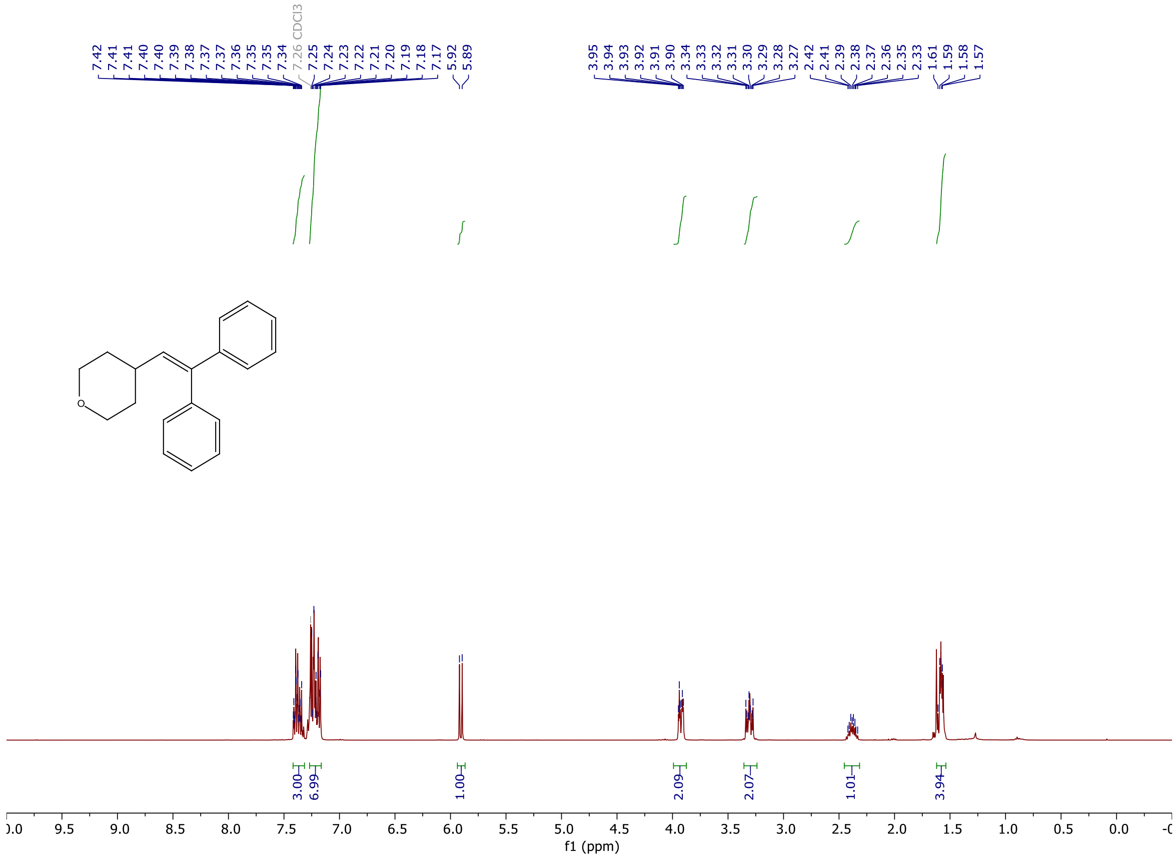

^{13}C NMR (101 MHz, $CDCl_3$) of **47**

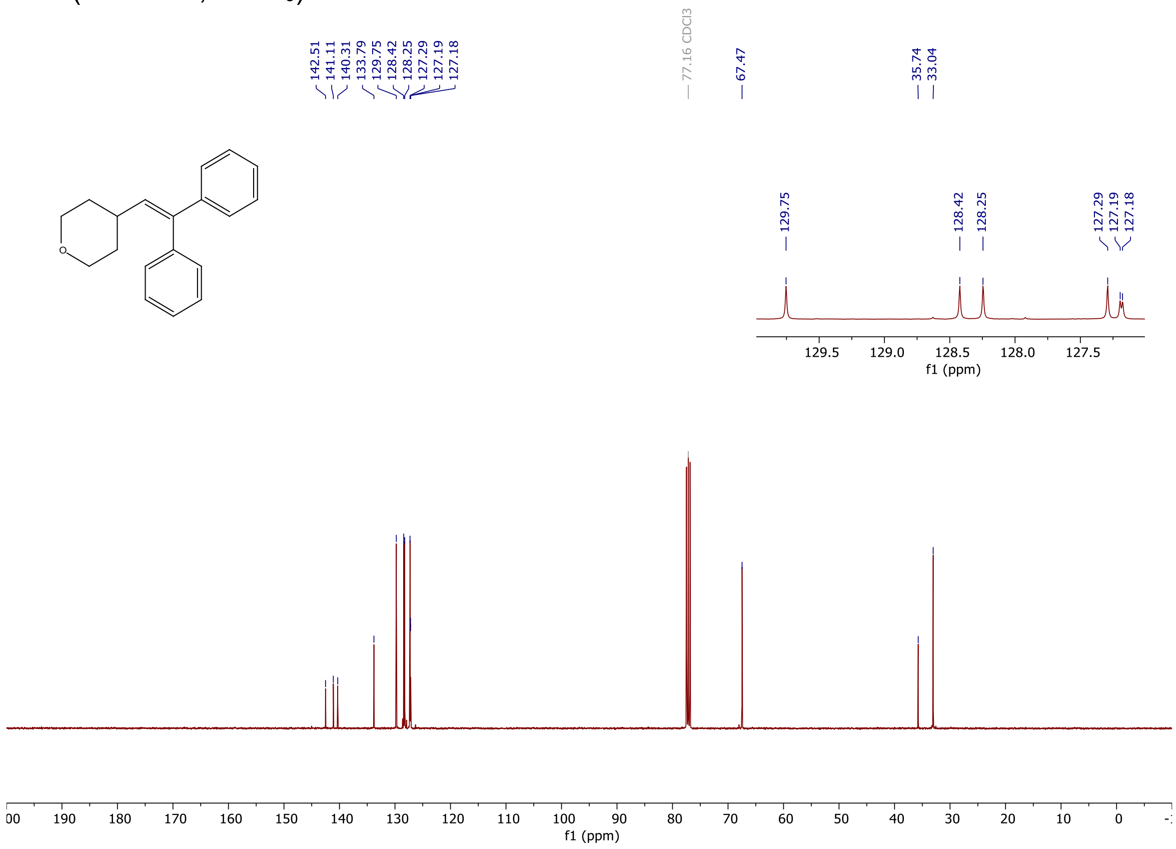

^{1}H NMR (400 MHz, $CDCl_3$) of **48**

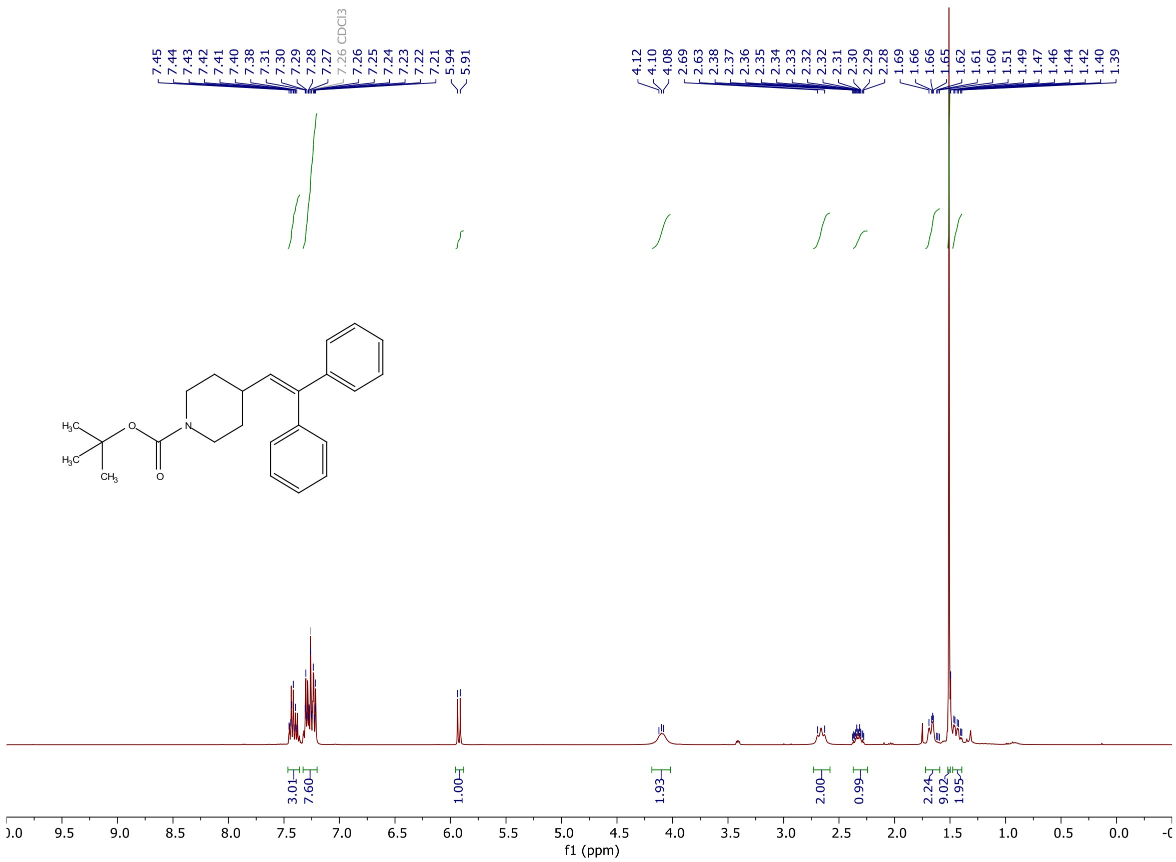

^{13}C NMR (101 MHz, $CDCl_3$) of **48**

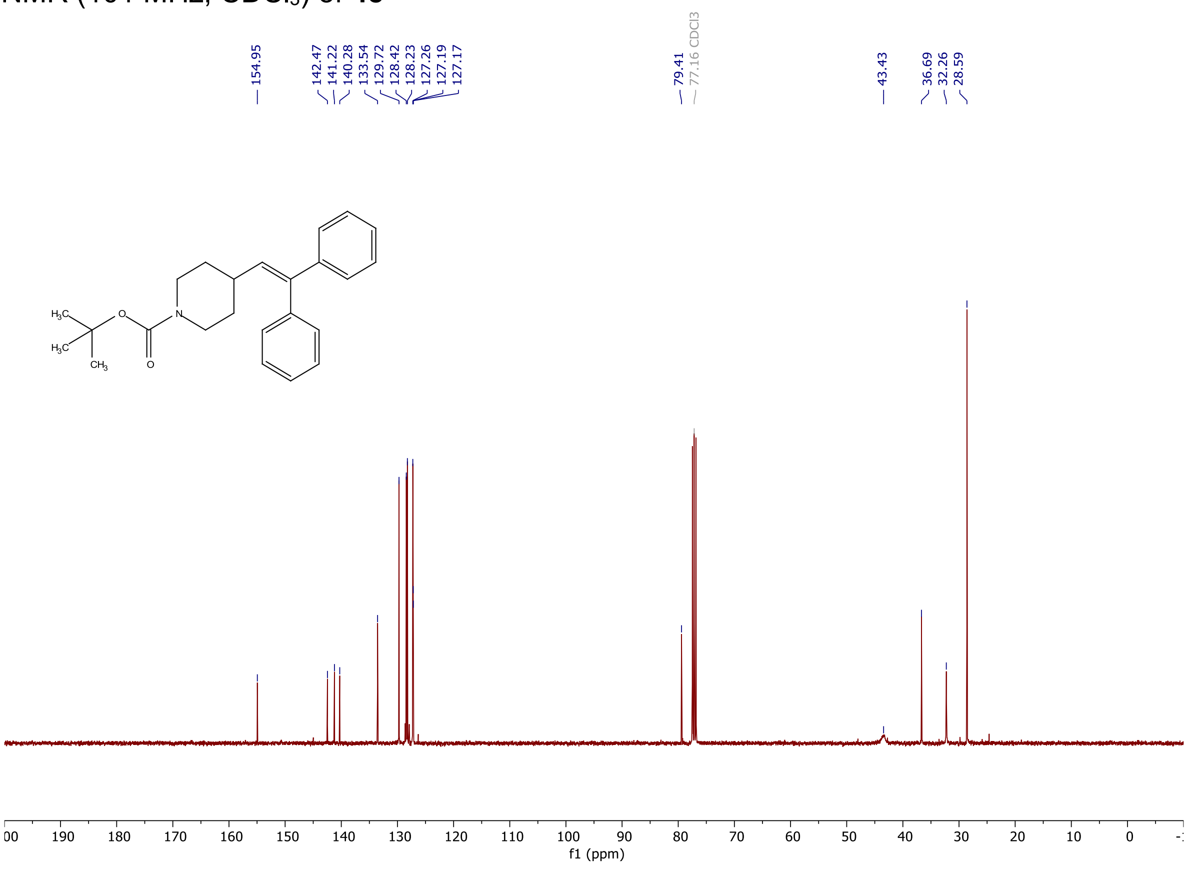

^{1}H NMR (400 MHz, $CDCl_3$) of **49**

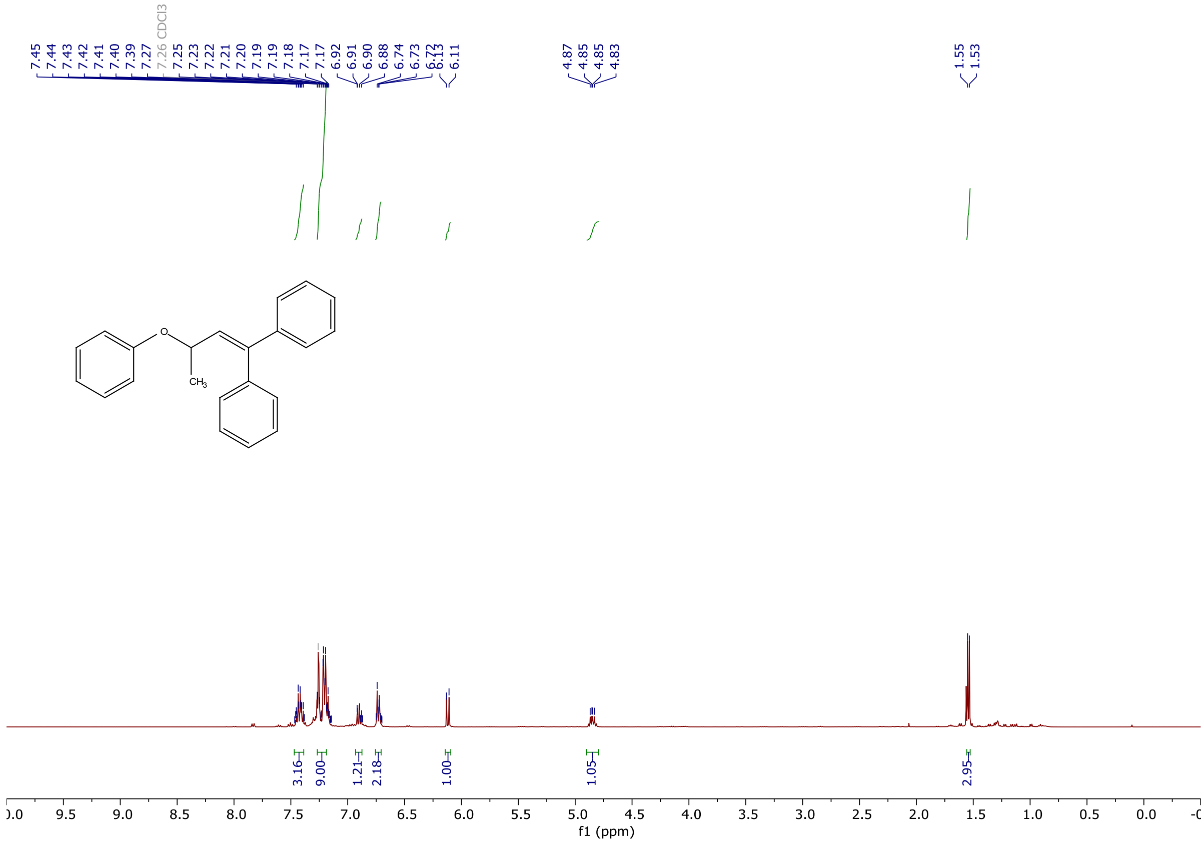

^{13}C NMR (101 MHz, $CDCl_3$) of **49**

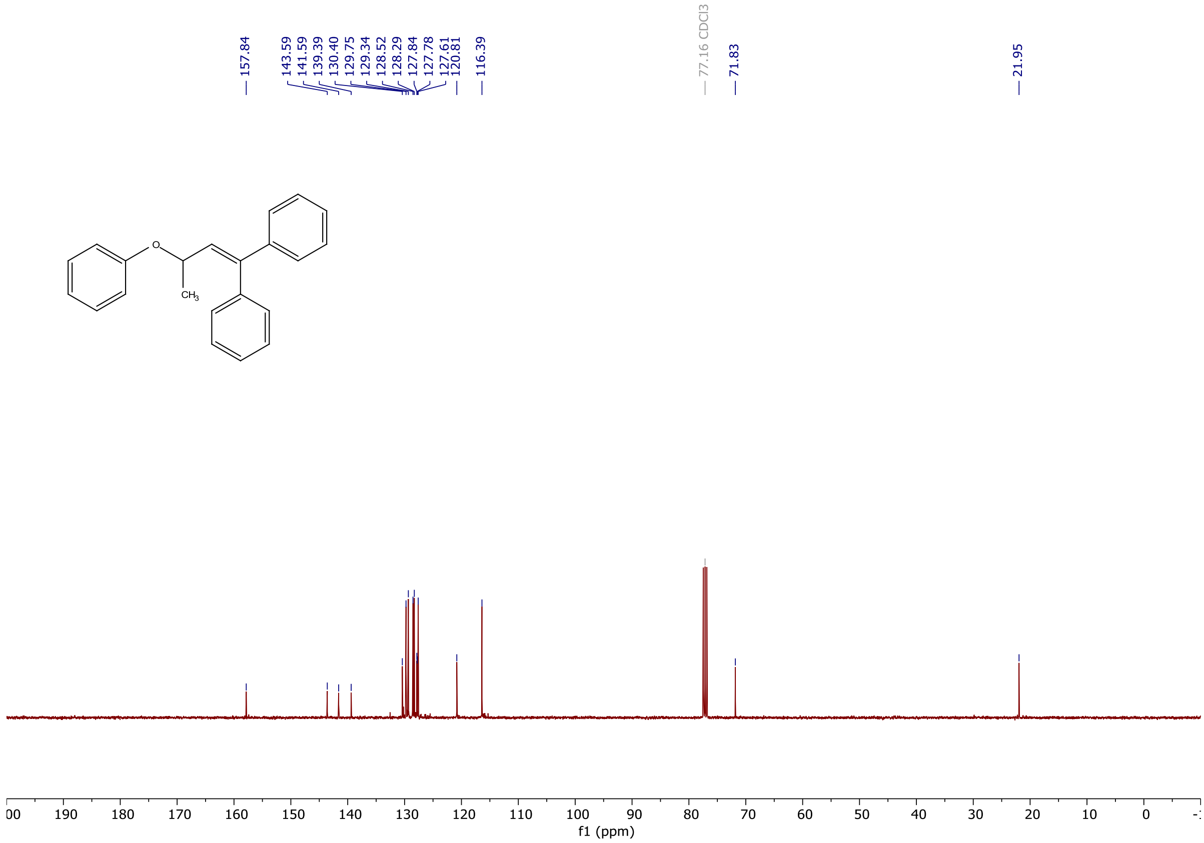

^{1}H NMR (400 MHz, $CDCl_3$) of **50**

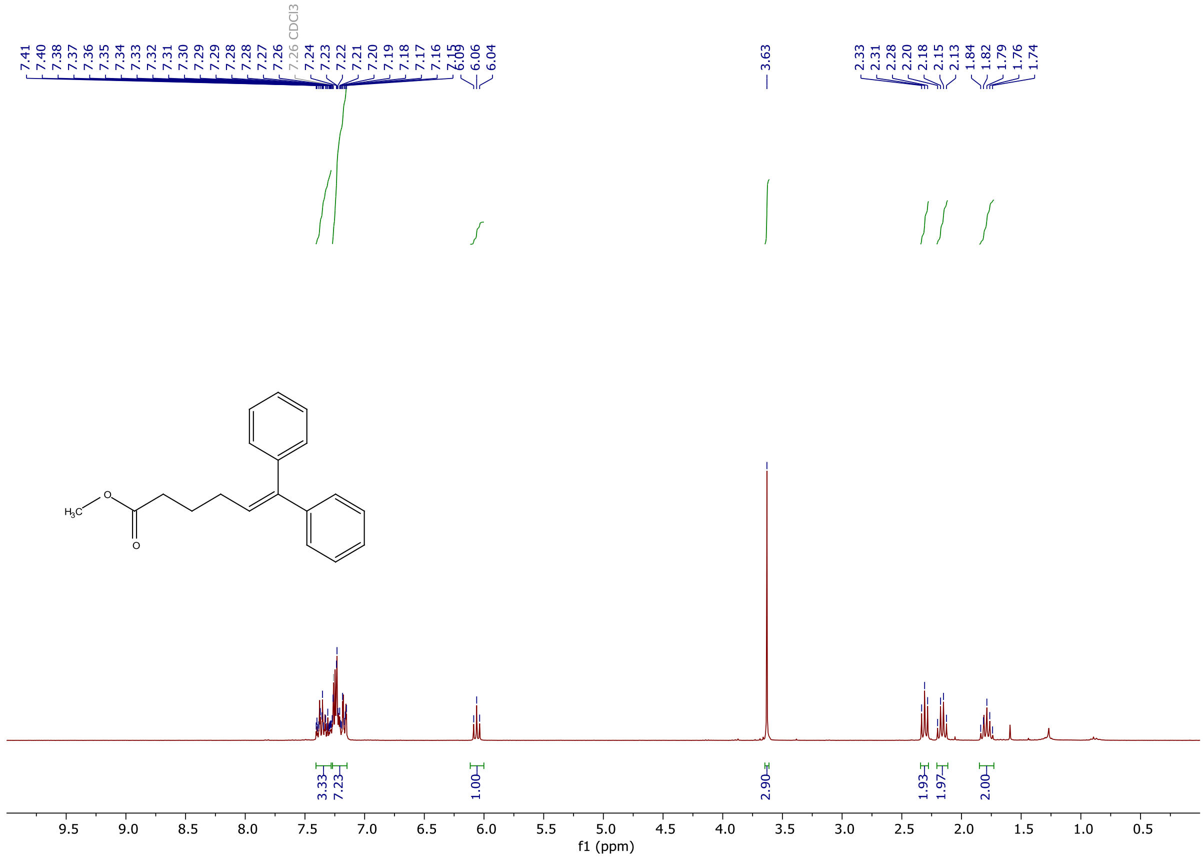

^{13}C NMR (101 MHz, $CDCl_3$) of **50**

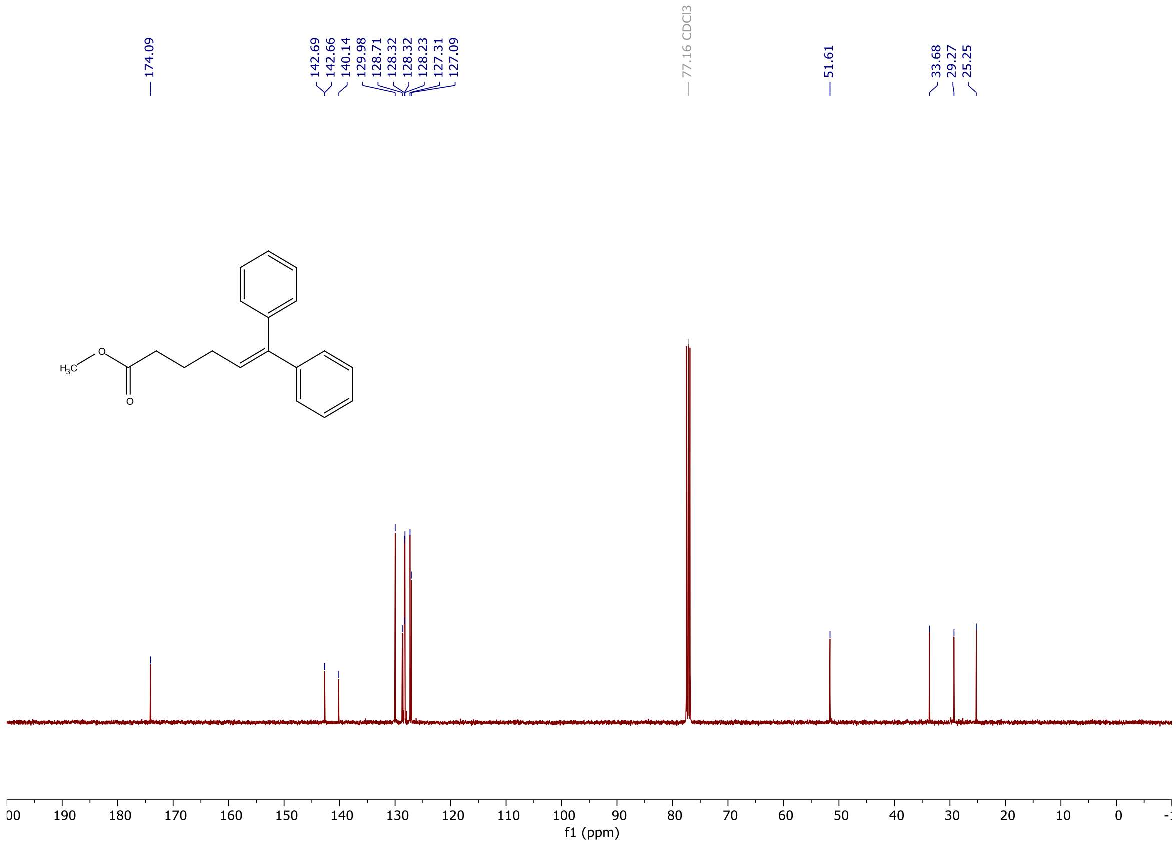

^{1}H NMR (400 MHz, $CDCl_3$) of **51**

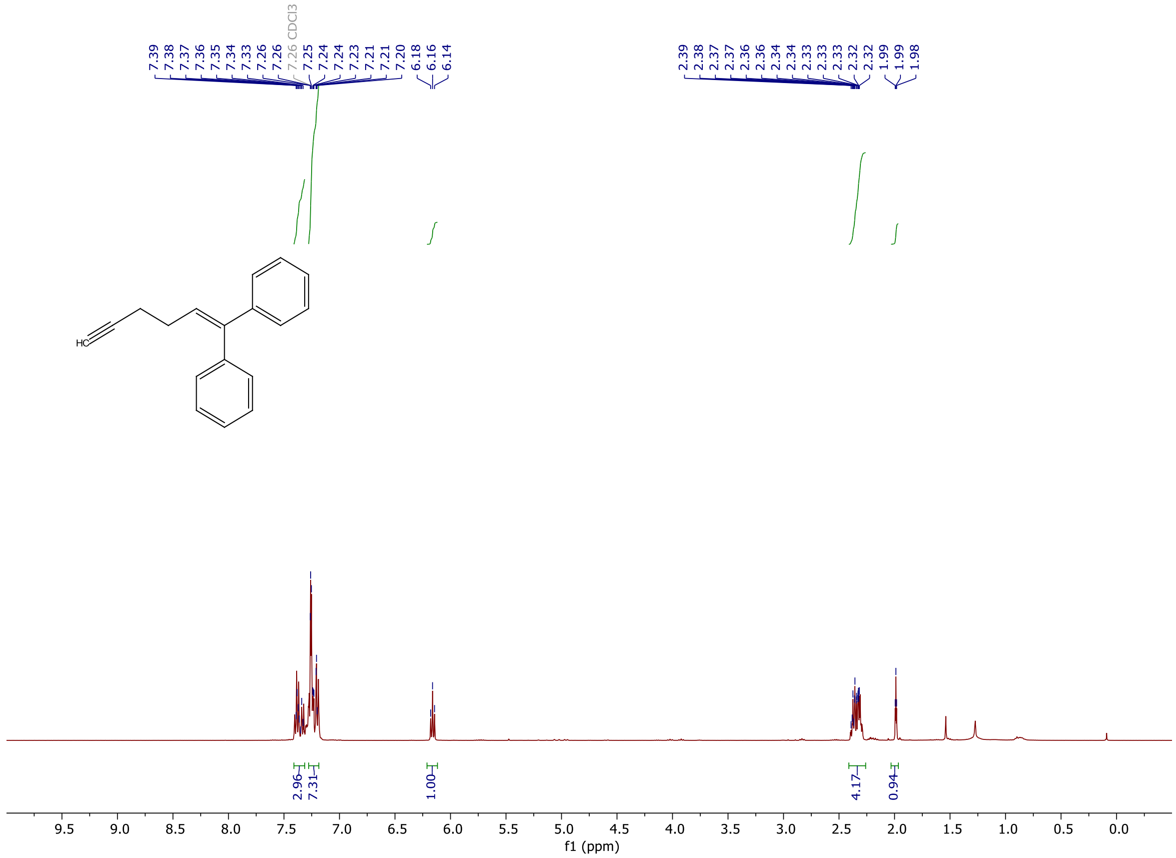

^{13}C NMR (101 MHz, $CDCl_3$) of **51**

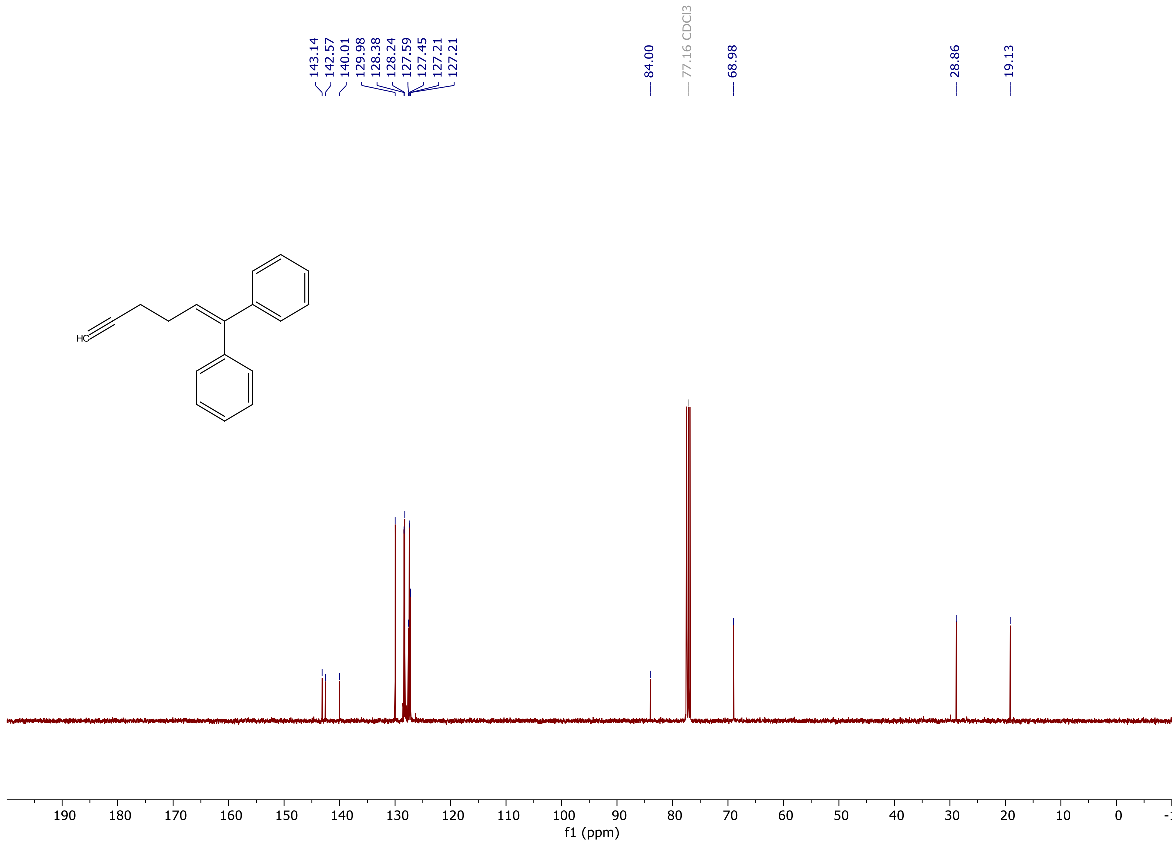

^{1}H NMR (400 MHz, $CDCl_3$) of **52**

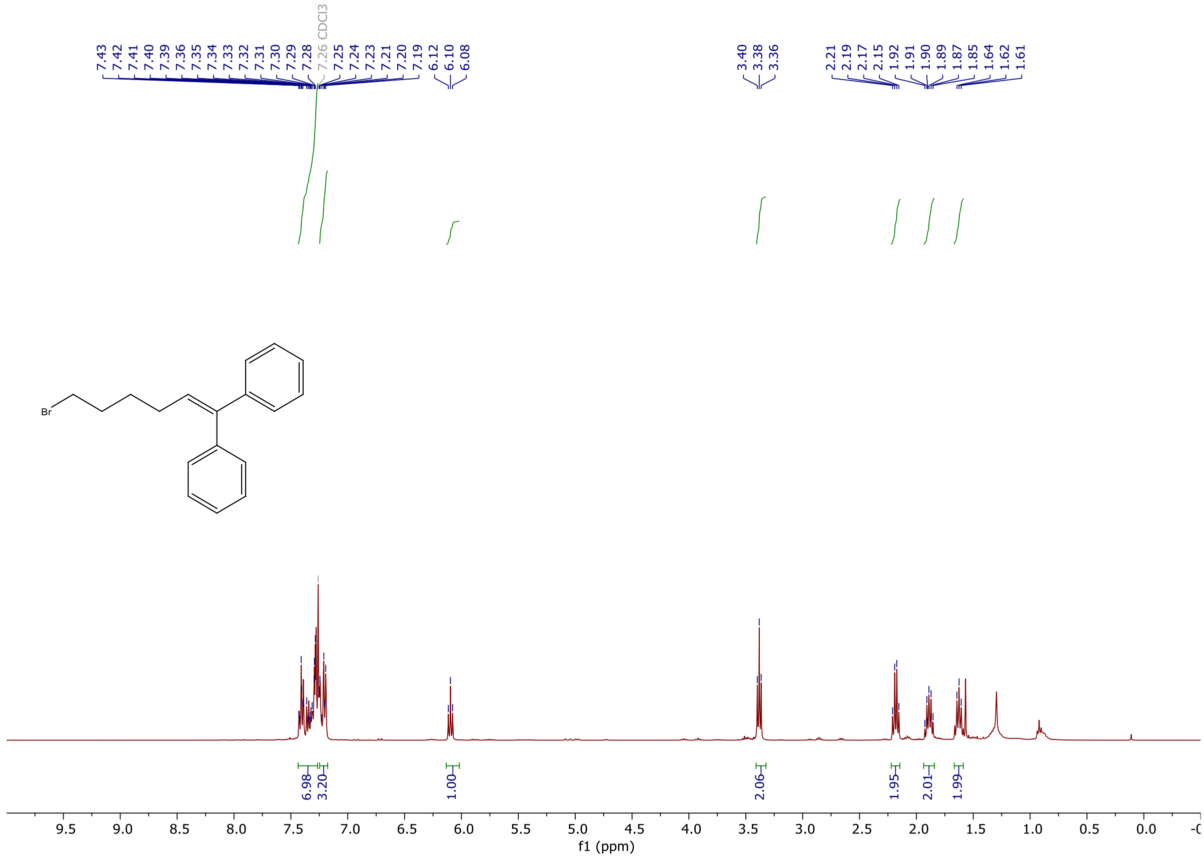

^{13}C NMR (101 MHz, $CDCl_3$) of **52**

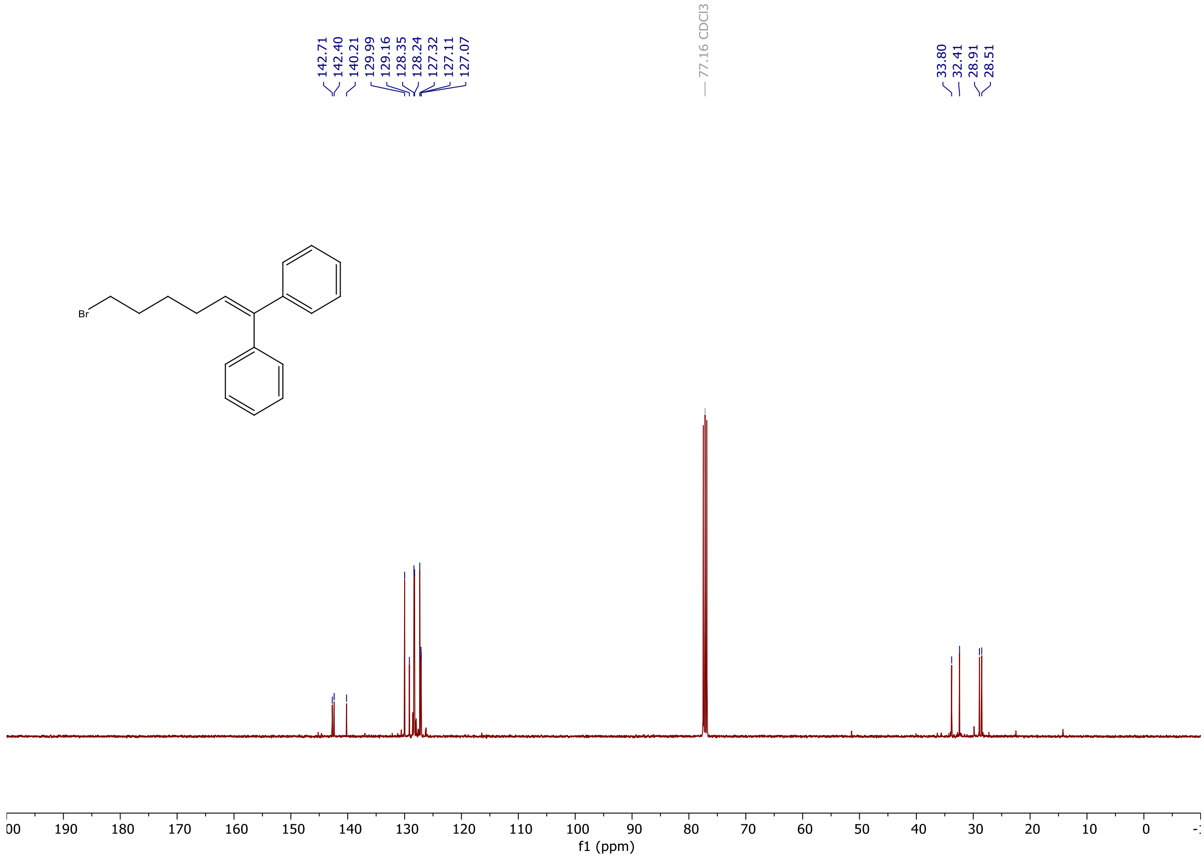

^{1}H NMR (400 MHz, $CDCl_3$) of **53**

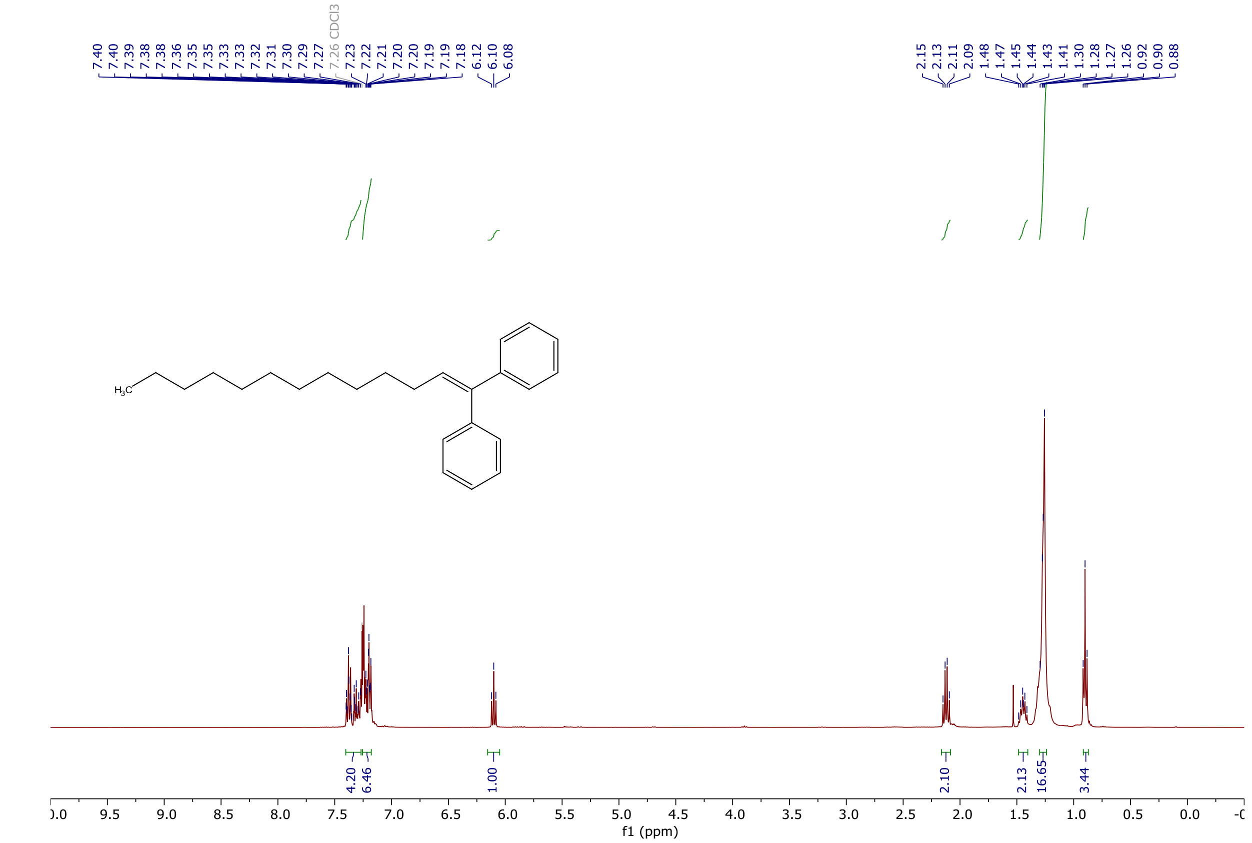

^{13}C NMR (101 MHz, $CDCl_3$) of **53**

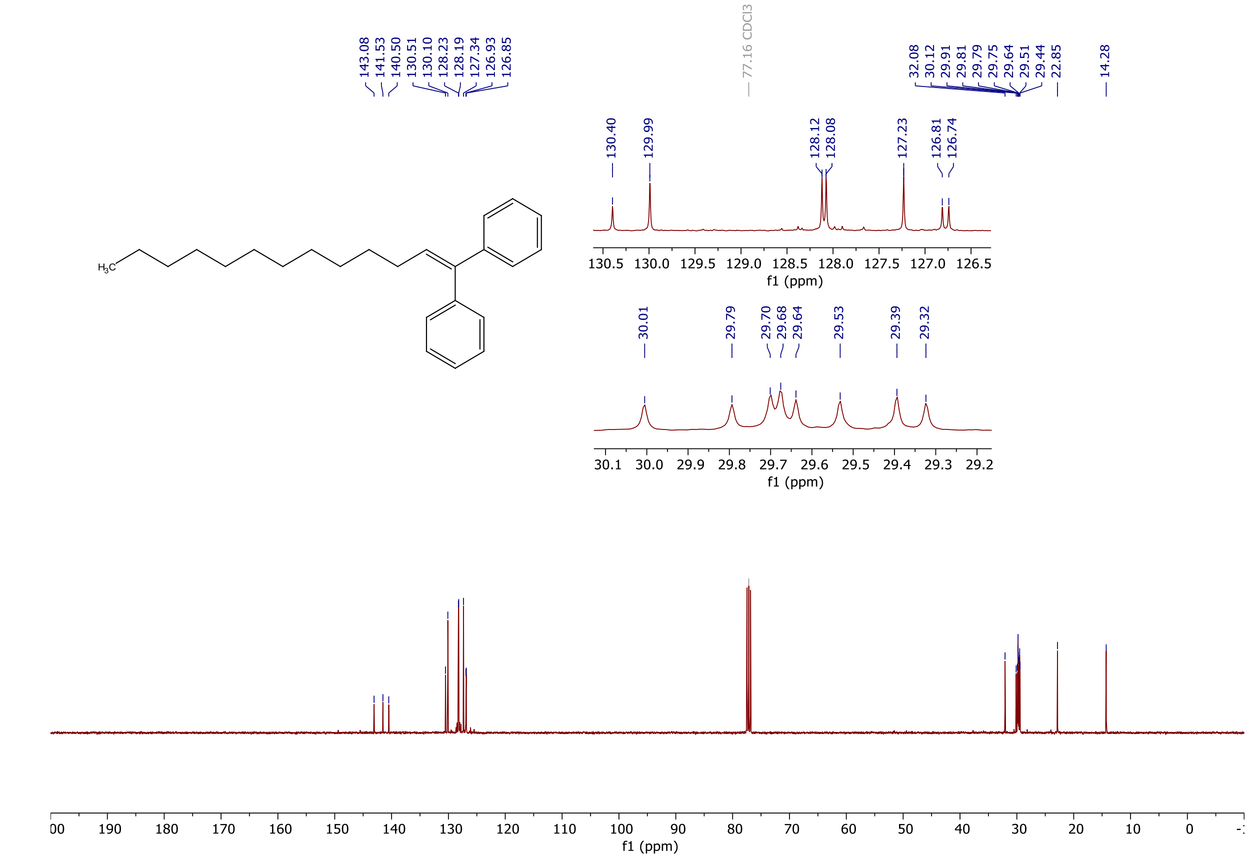

^{1}H NMR (400 MHz, $CDCl_3$) of **54**

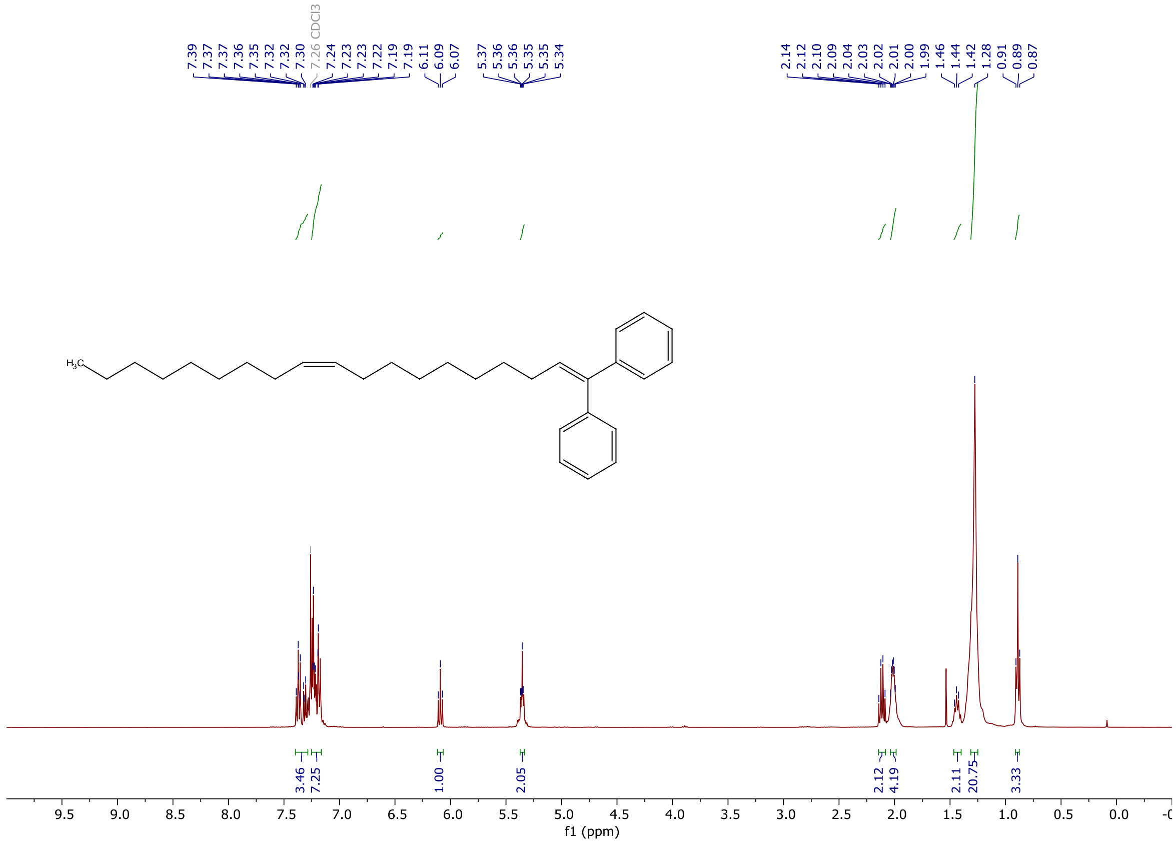

^{13}C NMR (101 MHz, $CDCl_3$) of **54**

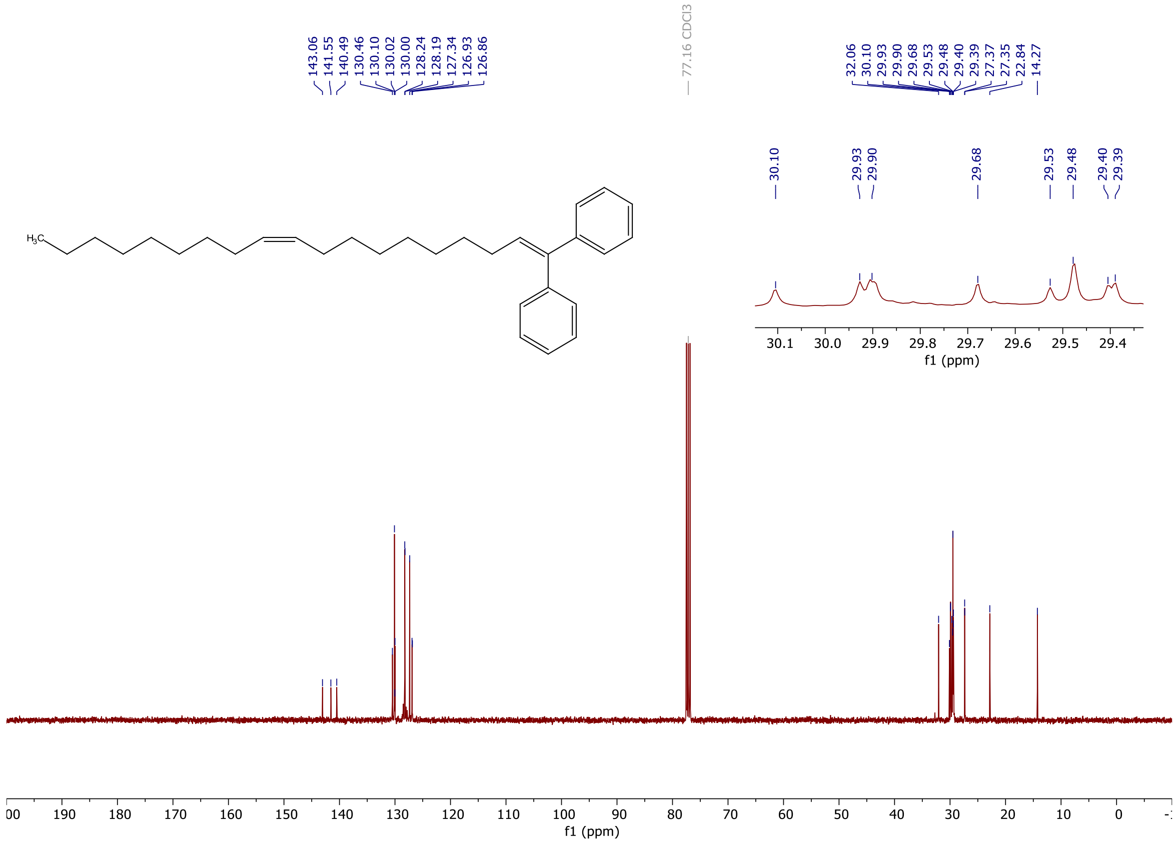

^{1}H NMR (400 MHz, $CDCl_3$) of **55**

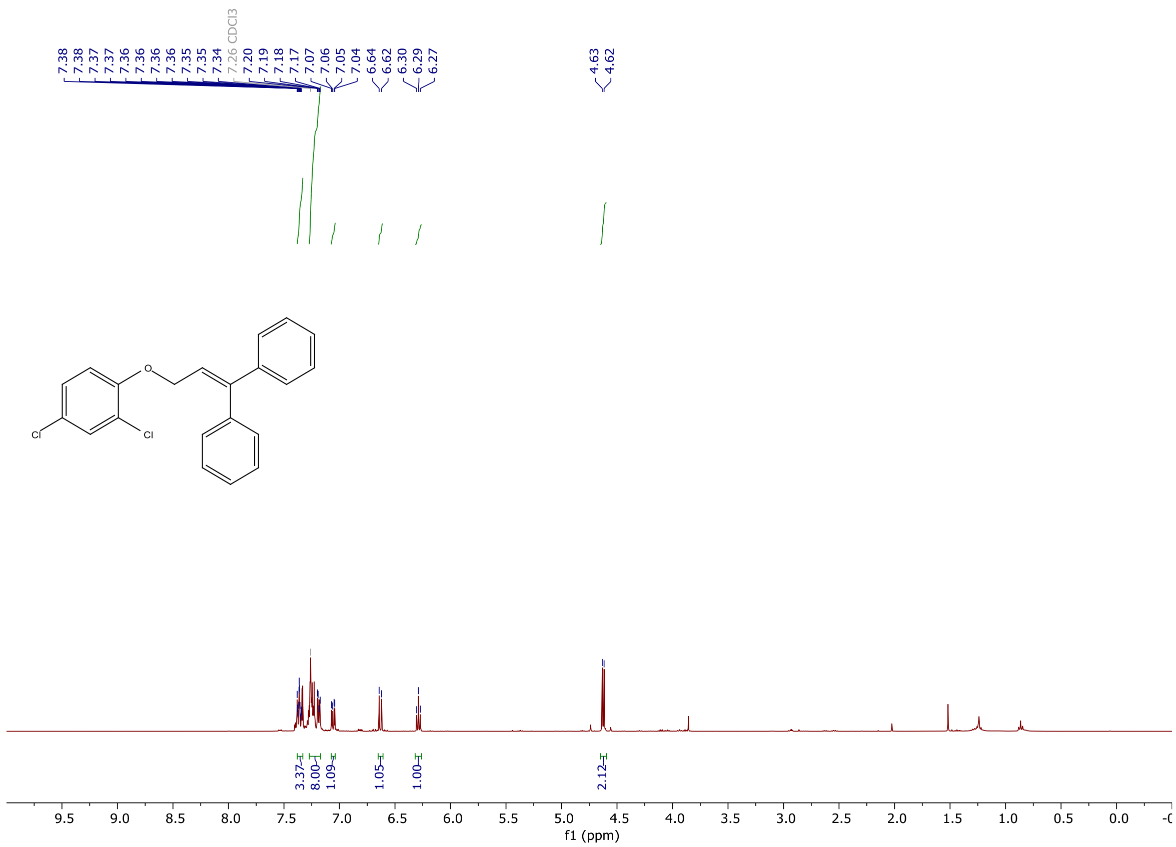

^{13}C NMR (101 MHz, $CDCl_3$) of **55**

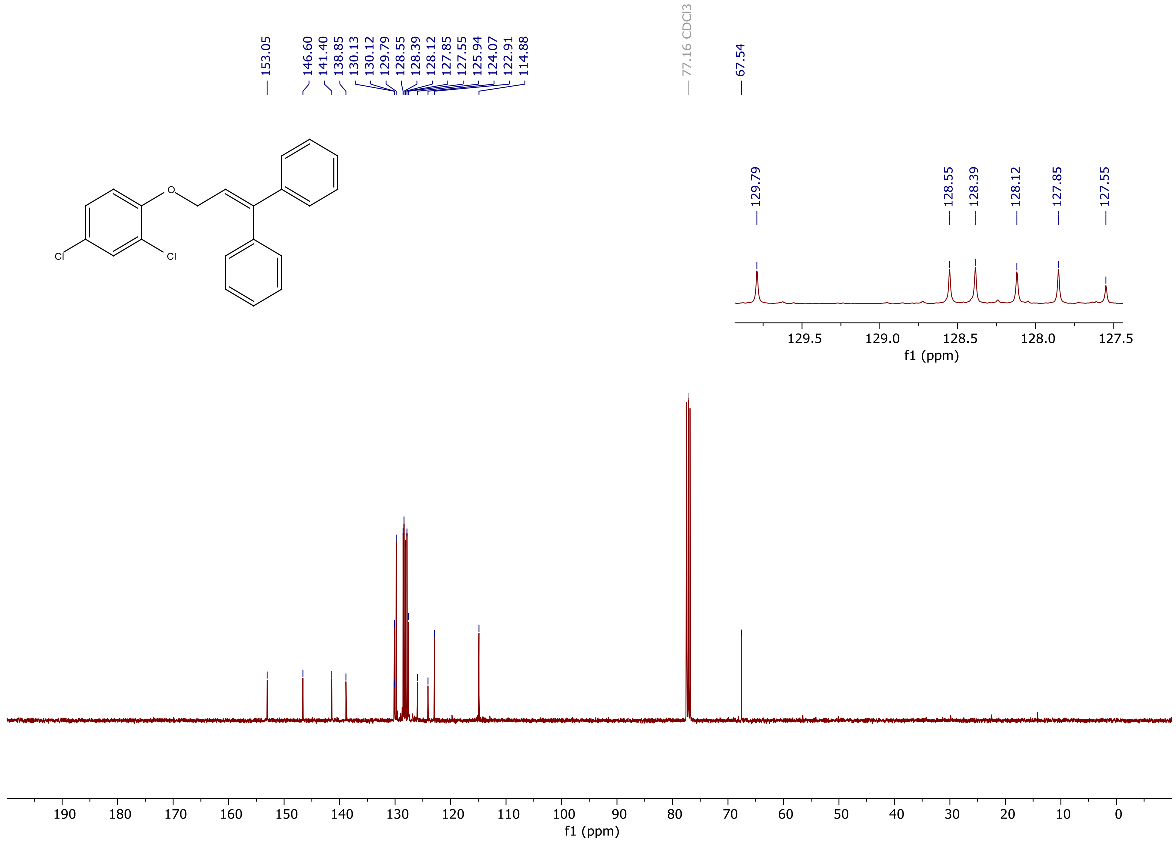

^{1}H NMR (400 MHz, $CDCl_3$) of **56**

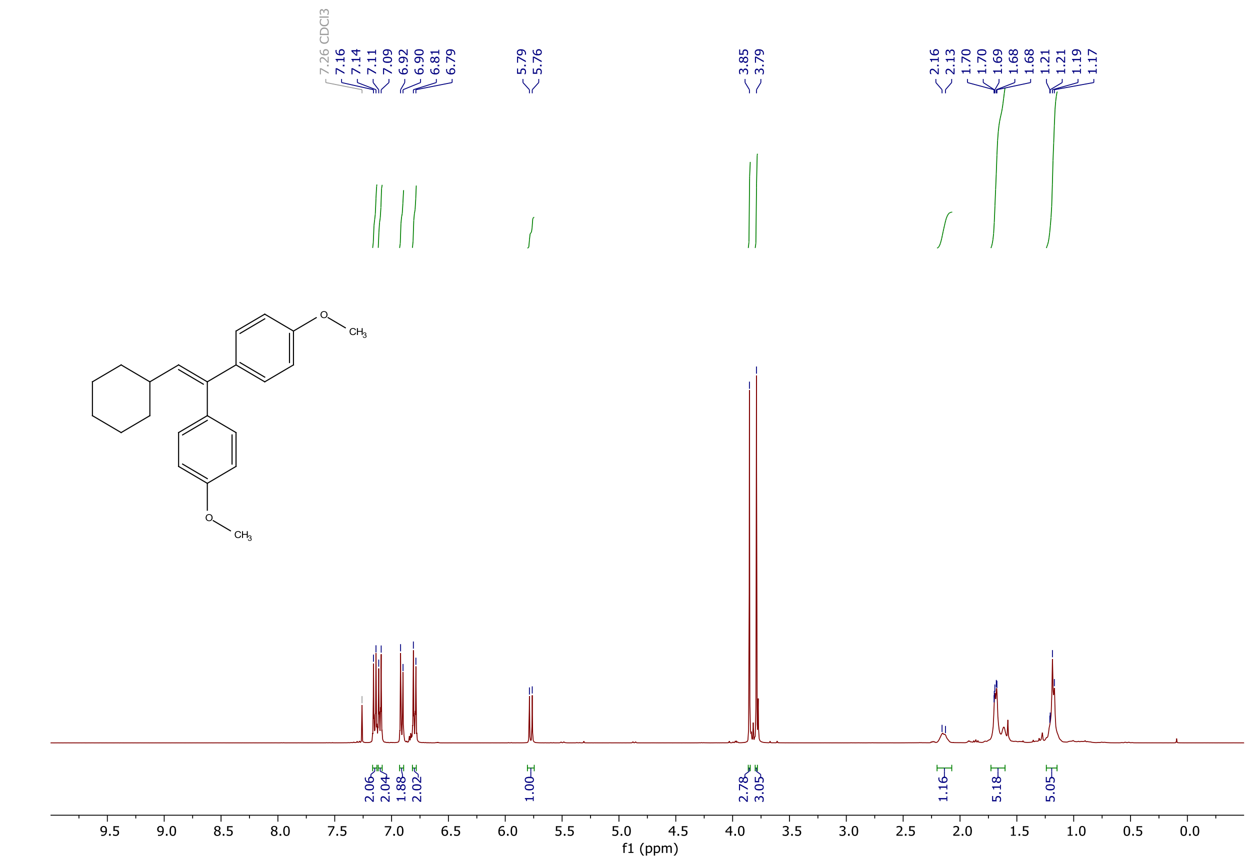

^{13}C NMR (101 MHz, $CDCl_3$) of **56**

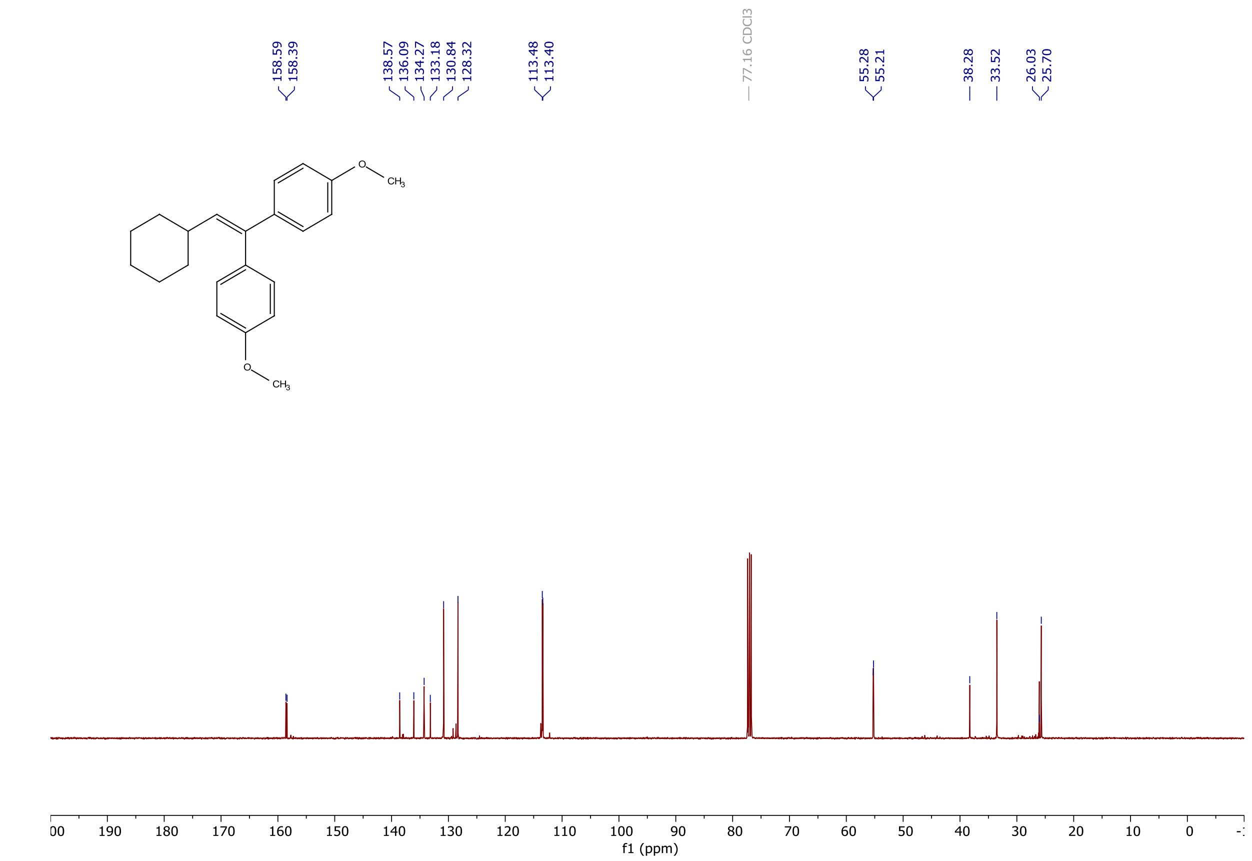

^{1}H NMR (400 MHz, $CDCl_3$) of **57**

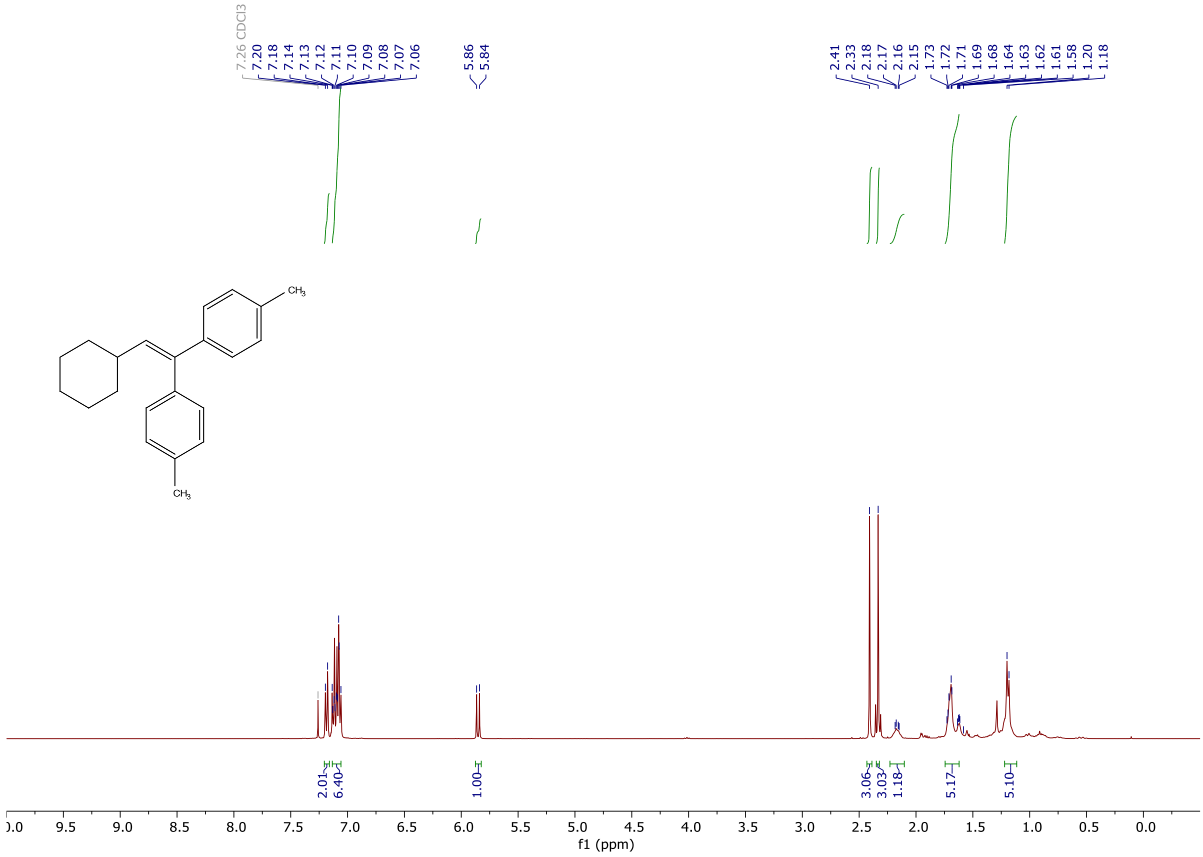

^{13}C NMR (101 MHz, $CDCl_3$) of **57**

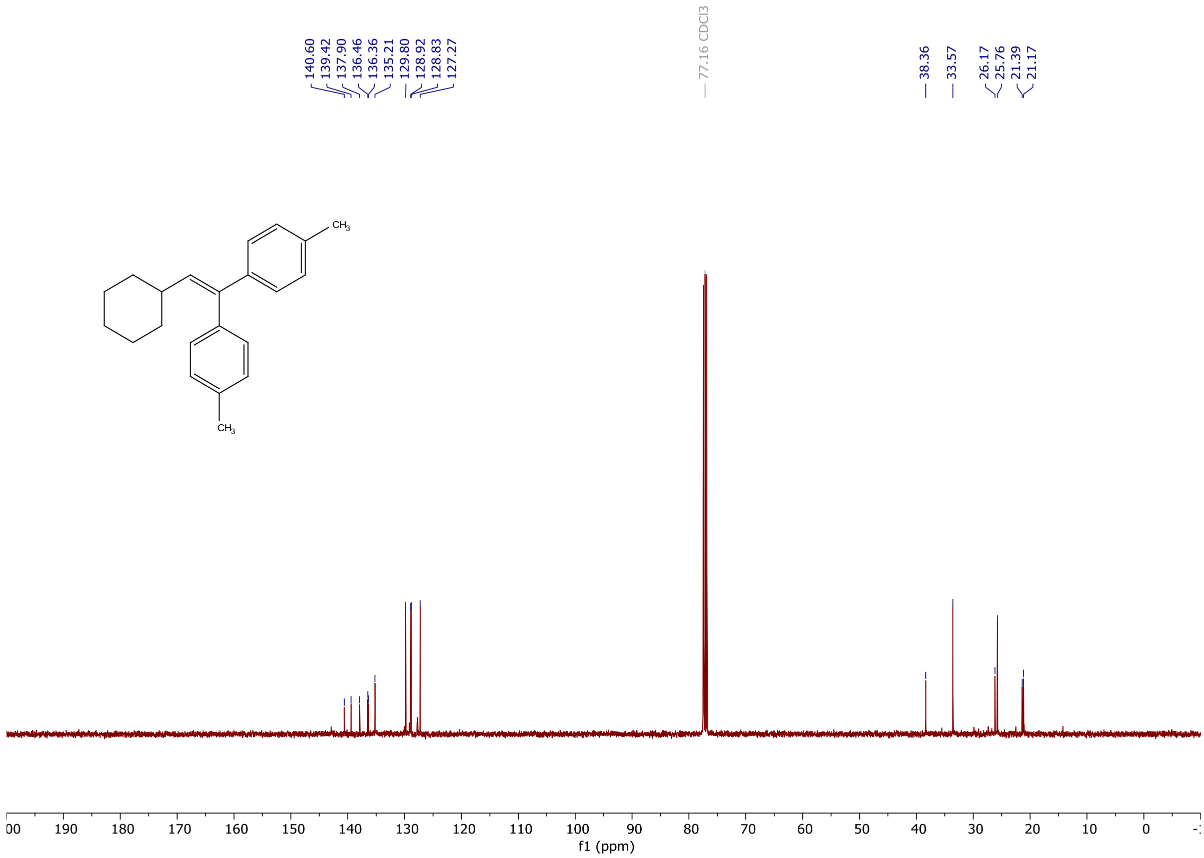

^{1}H NMR (400 MHz, $CDCl_3$) of **58**

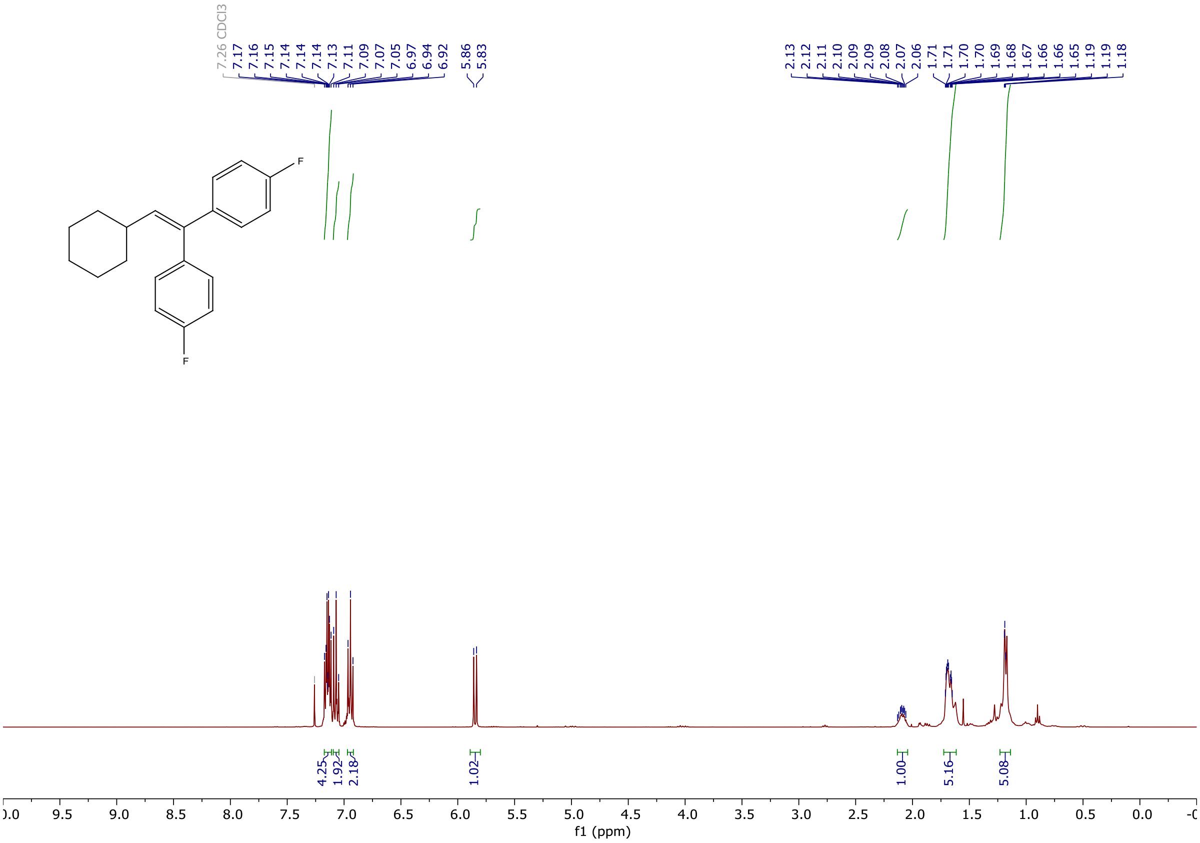

^{13}C NMR (101 MHz, $CDCl_3$) of **58**

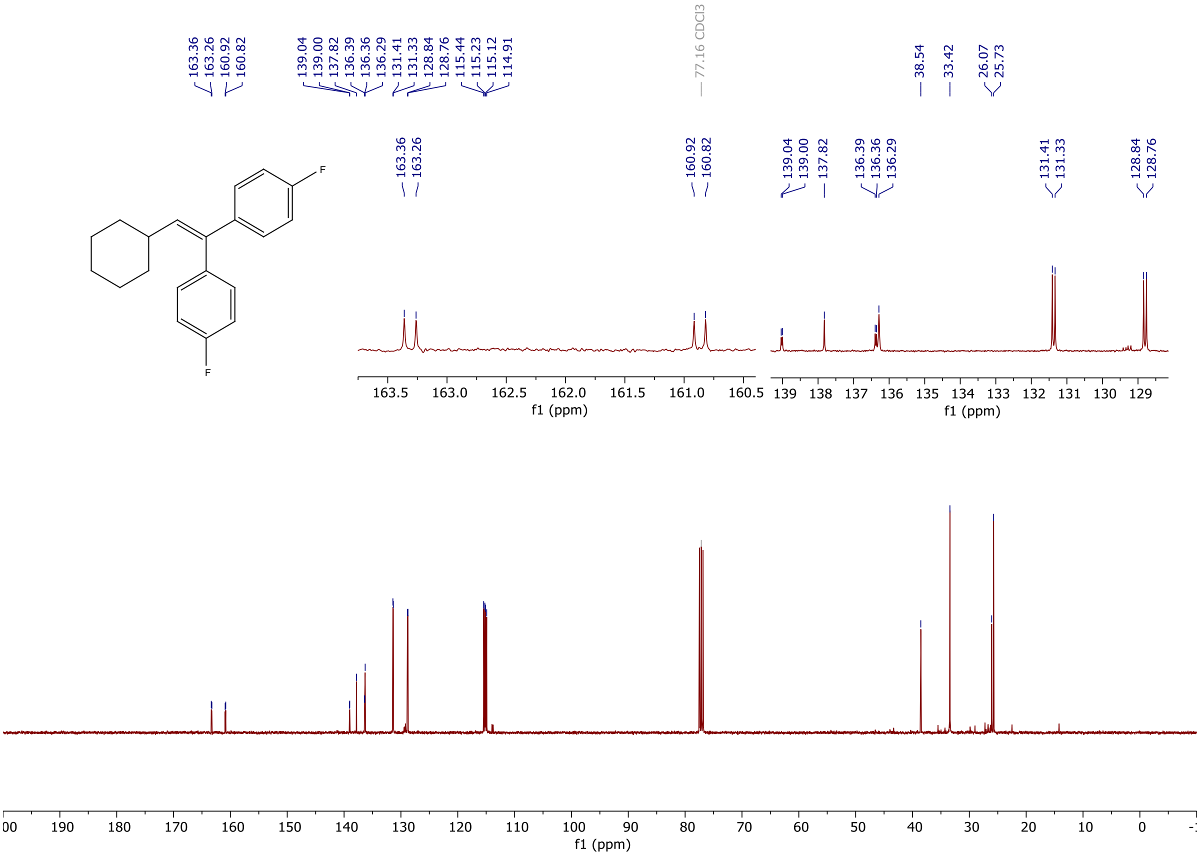

^{19}F NMR (376 MHz, $CDCl_3$) of **58**

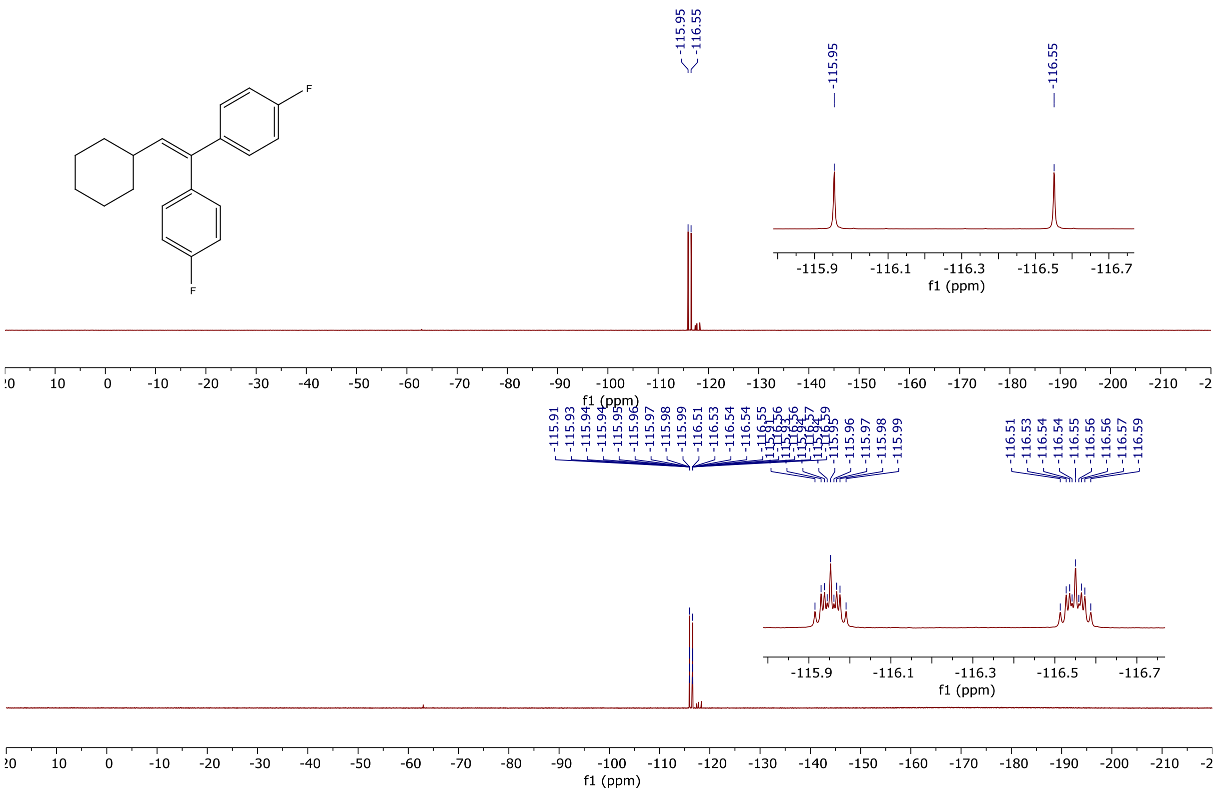

^{1}H NMR (400 MHz, $CDCl_3$) of **59**

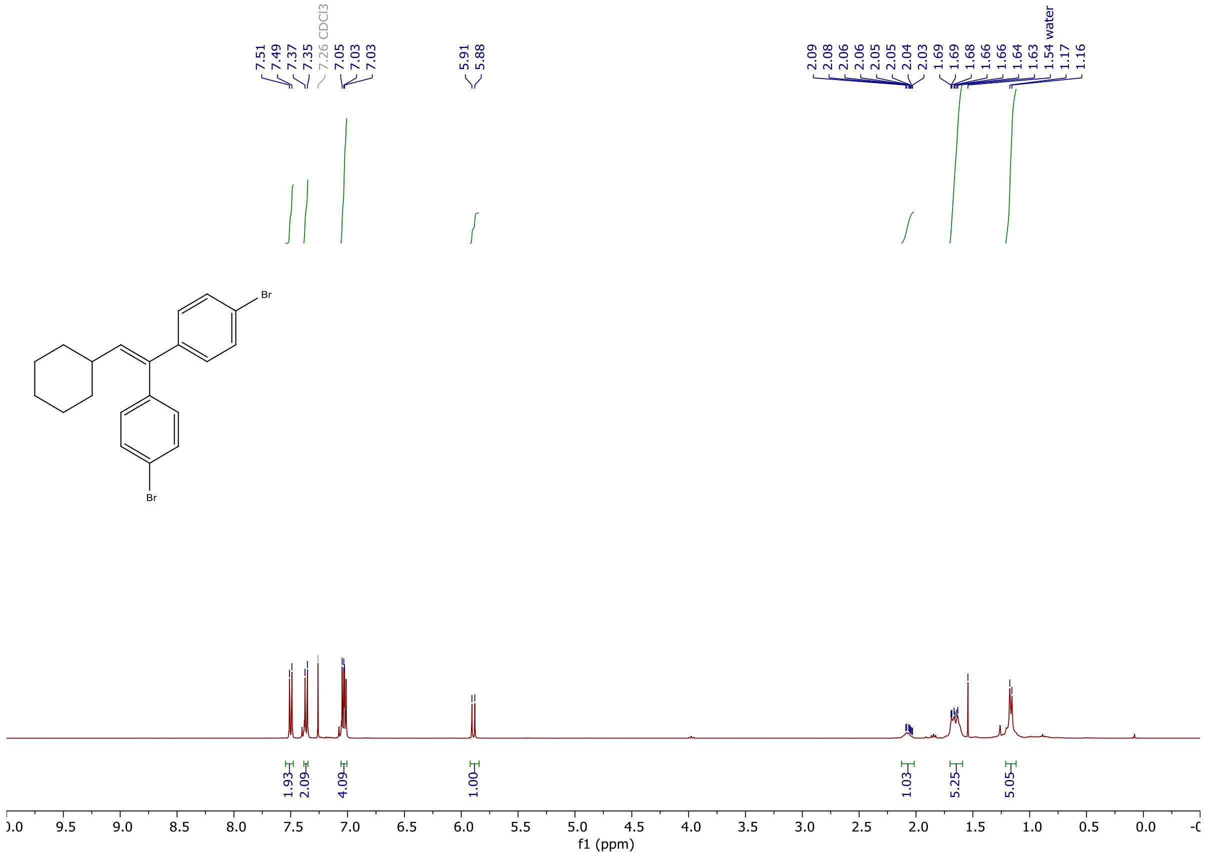

^{13}C NMR (101 MHz, $CDCl_3$) of **59**

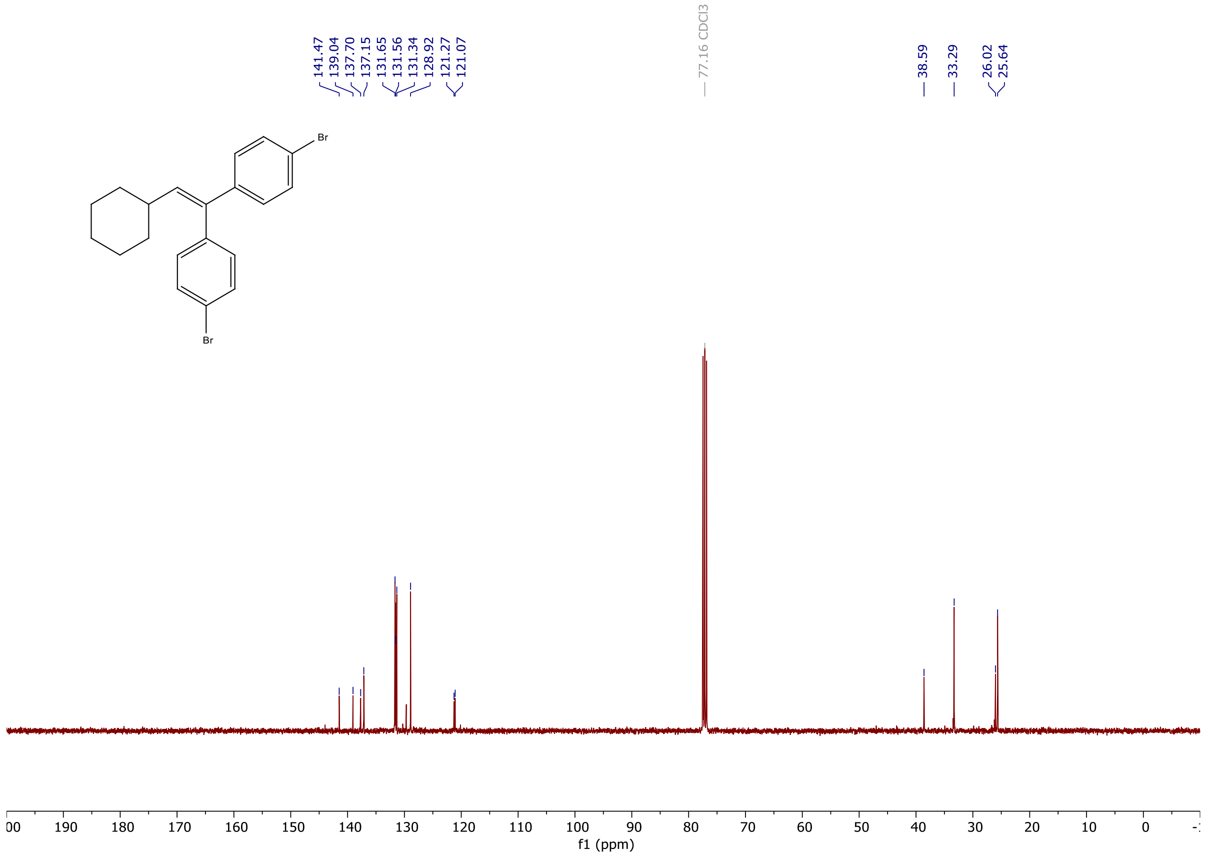

^{1}H NMR (400 MHz, $CDCl_3$) of **60**

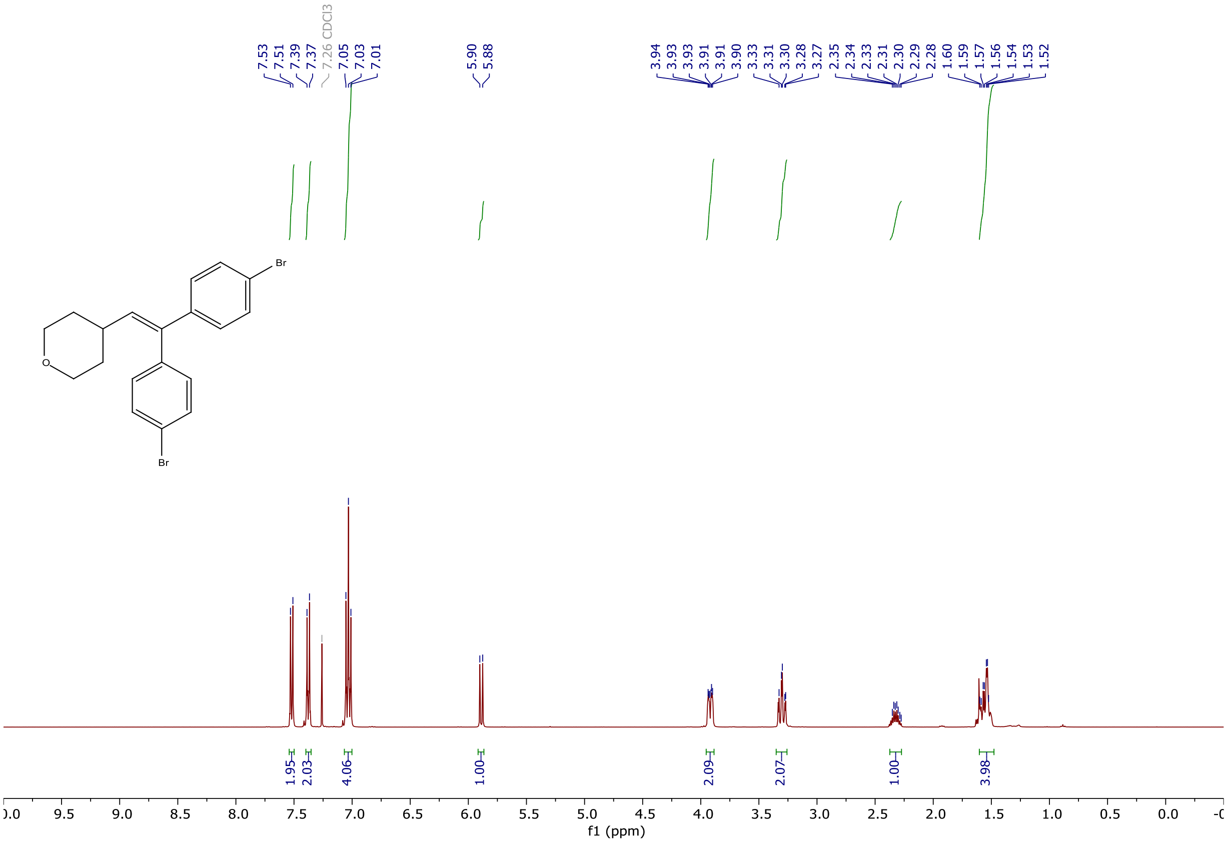

^{13}C NMR (101 MHz, $CDCl_3$) of **60**

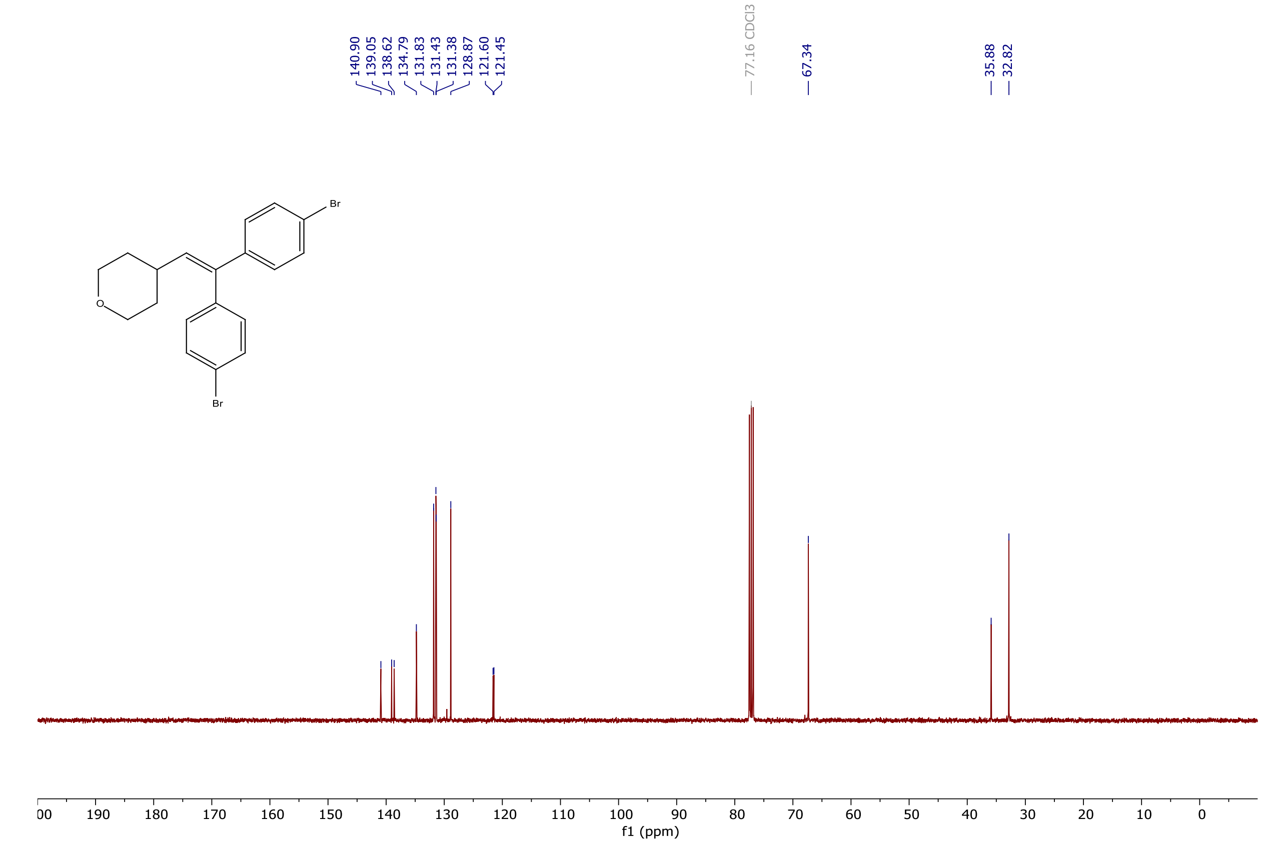

^{1}H NMR (400 MHz, $CDCl_3$) of **61**

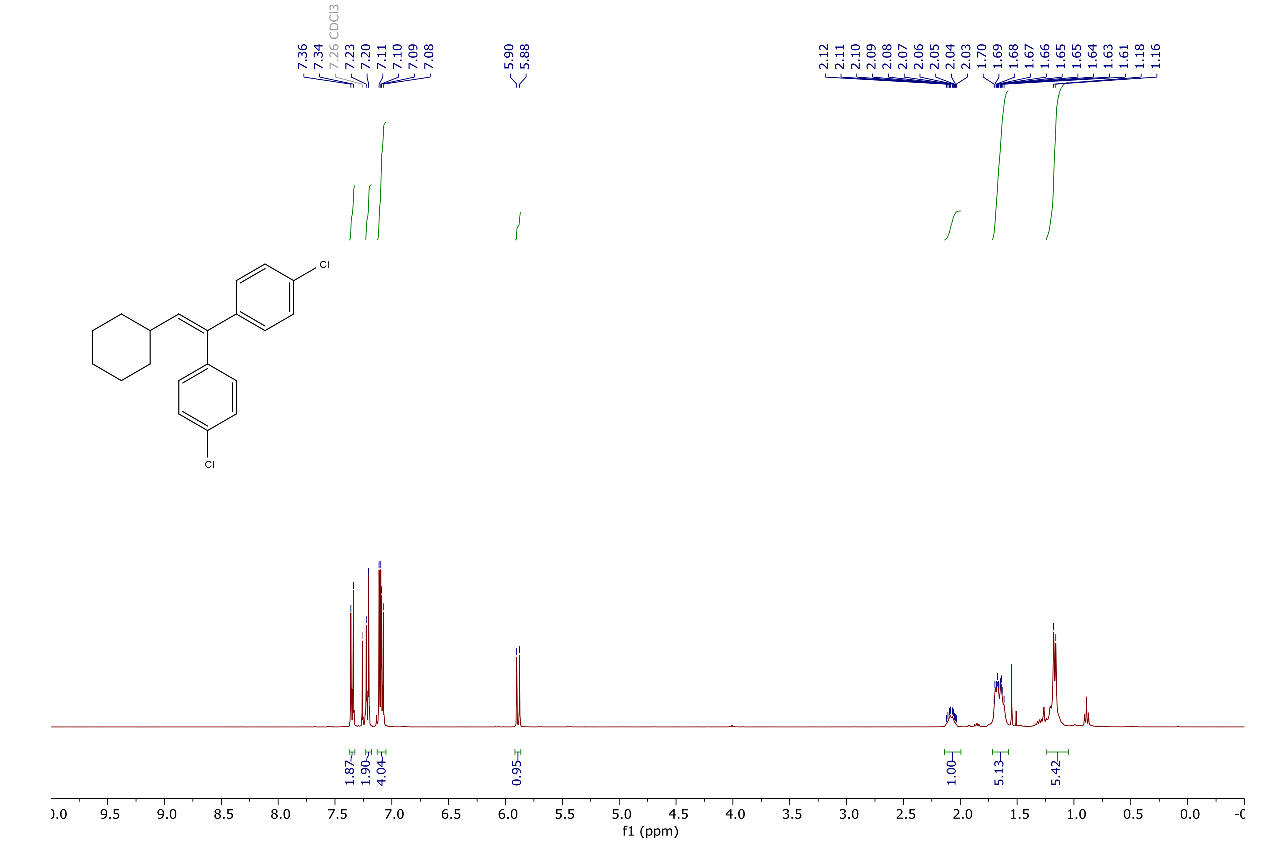

^{13}C NMR (101 MHz, $CDCl_3$) of **61**

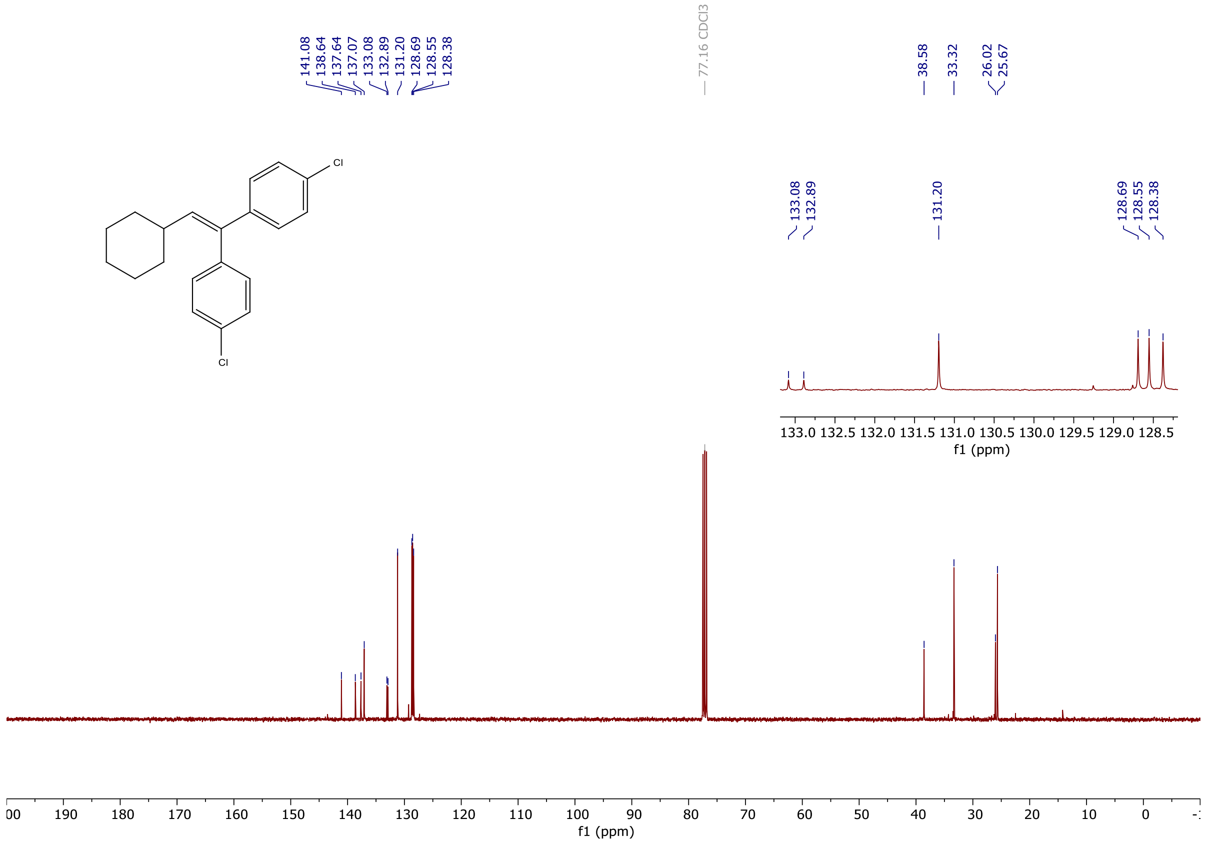

^{1}H NMR (400 MHz, $CDCl_3$) of **62**

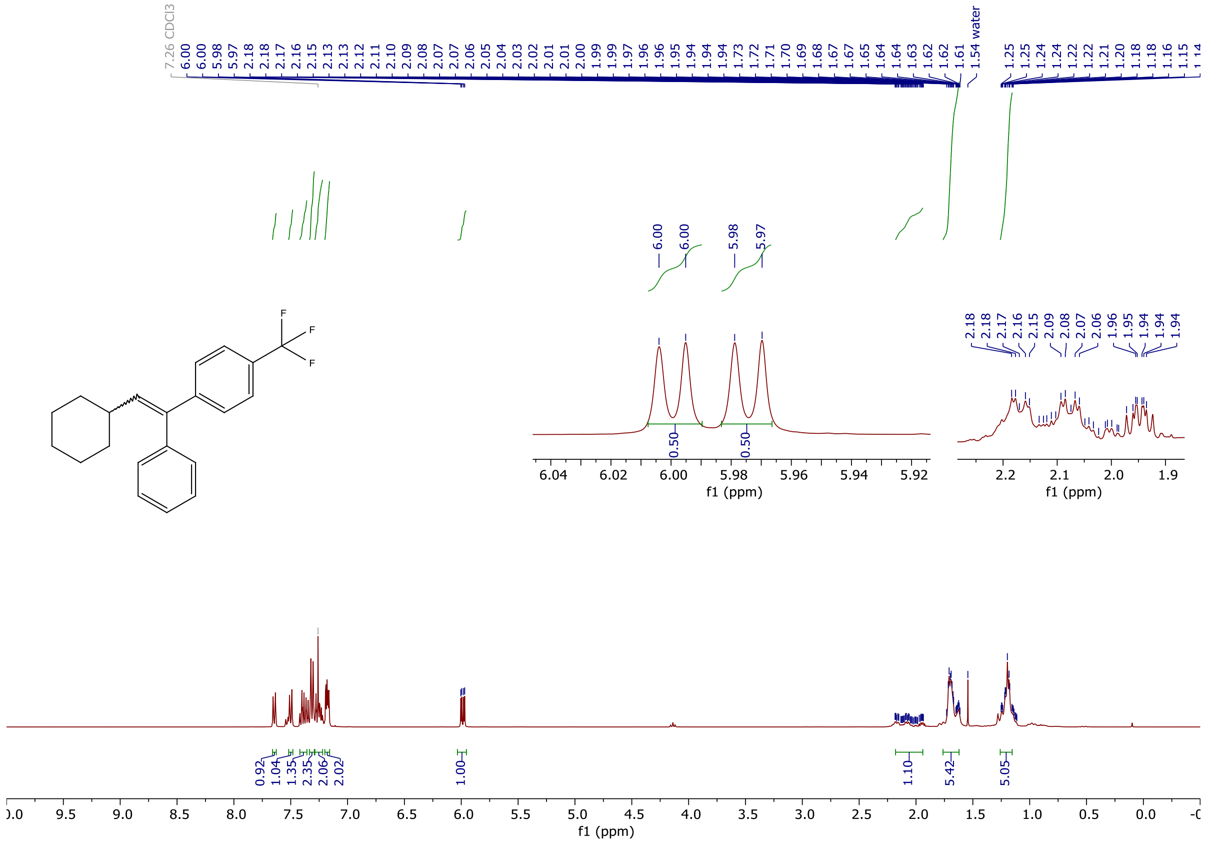

^{13}C NMR (151 MHz, $CDCl_3$) of **62**

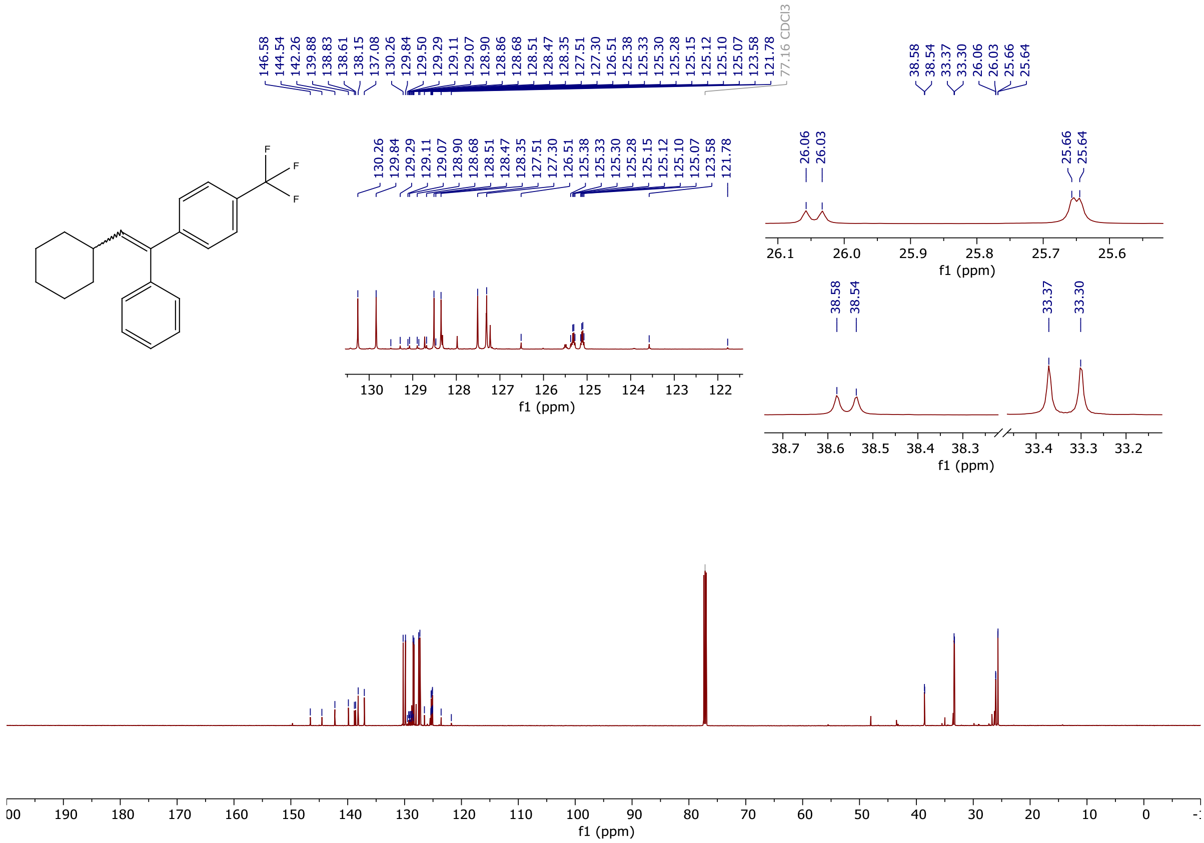

^{19}F NMR (376 MHz, $CDCl_3$) of **62**

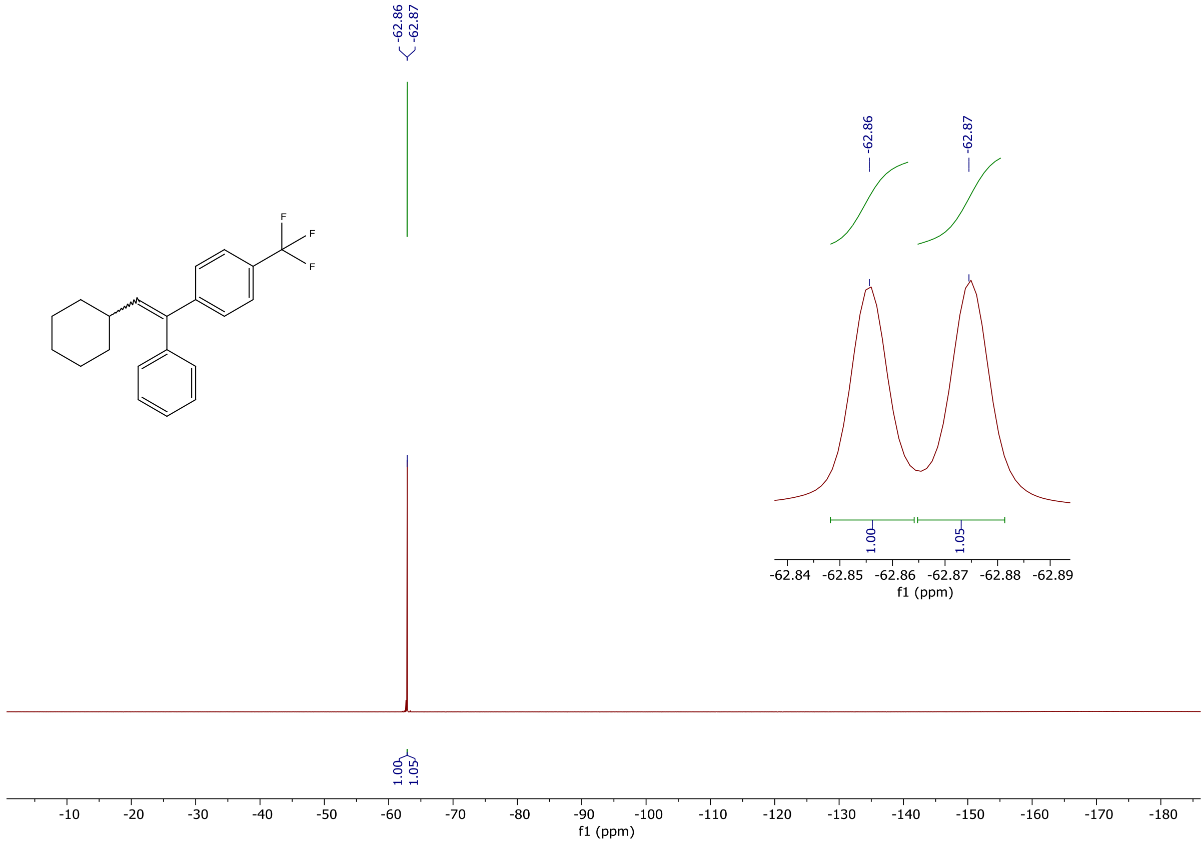

^{1}H NMR (400 MHz, $CDCl_3$) of **63**

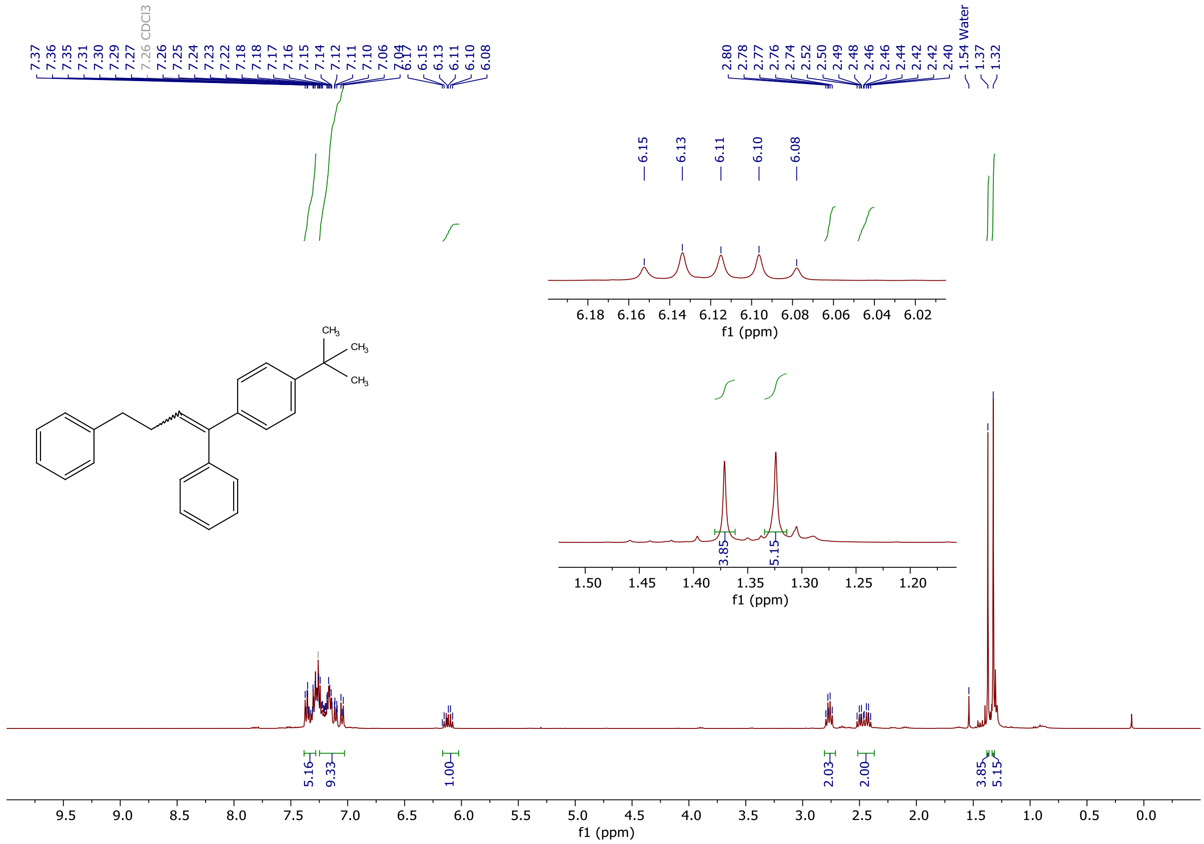

^{13}C NMR (101 MHz, $CDCl_3$) of **63**

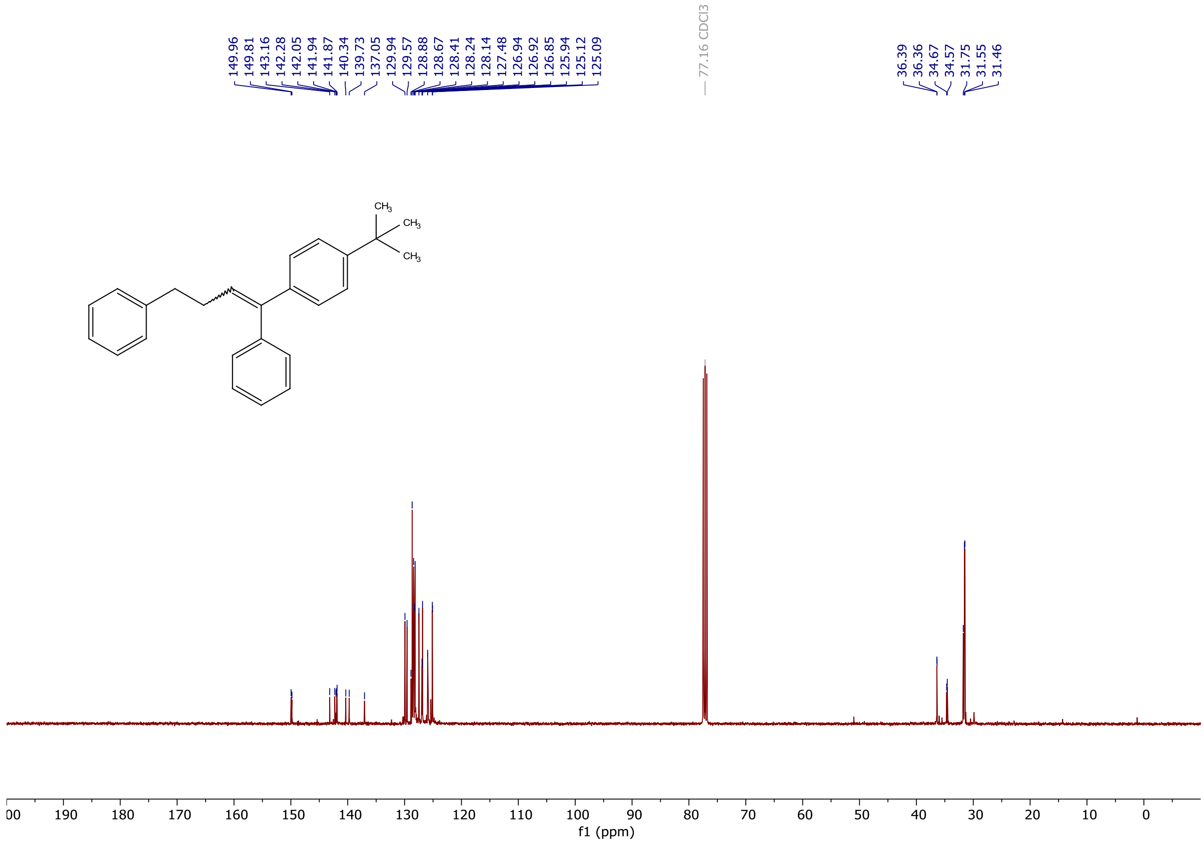

^{1}H NMR (400 MHz, $CDCl_3$) of **64**

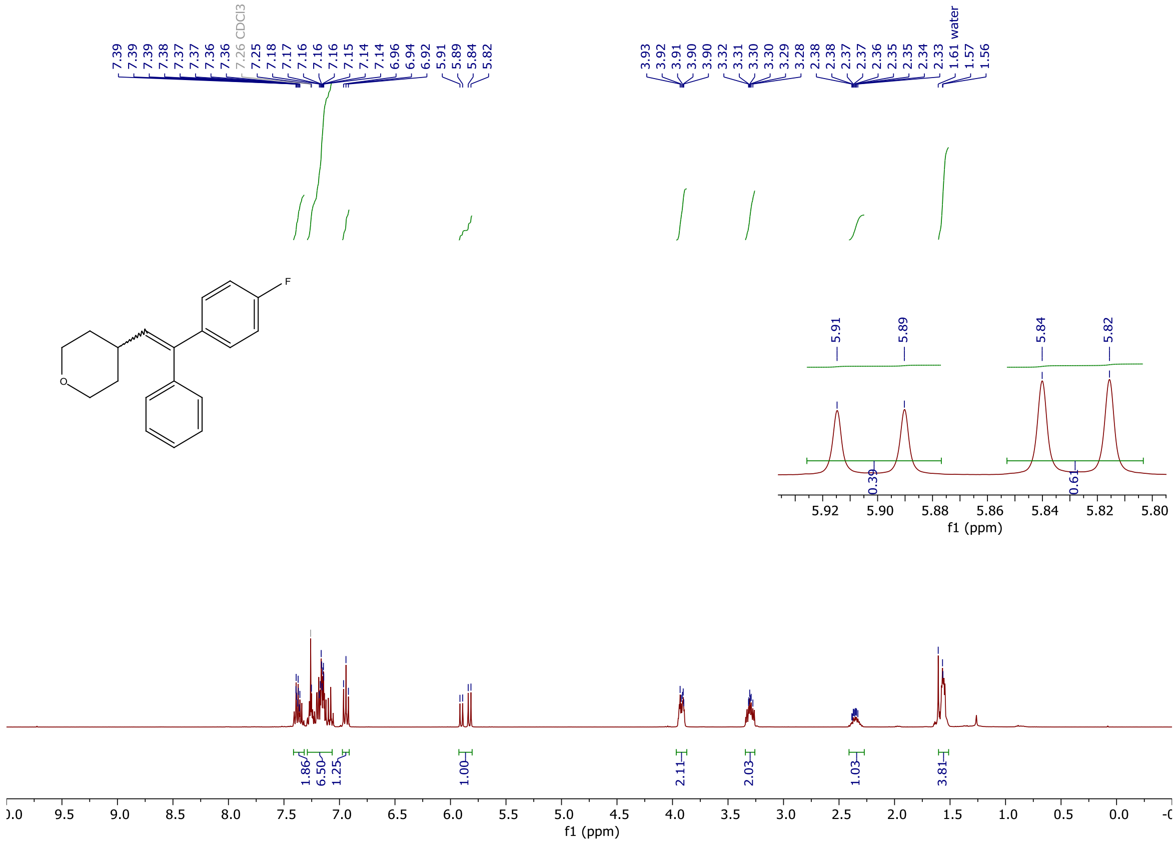

^{13}C NMR (101 MHz, $CDCl_3$) of **64**

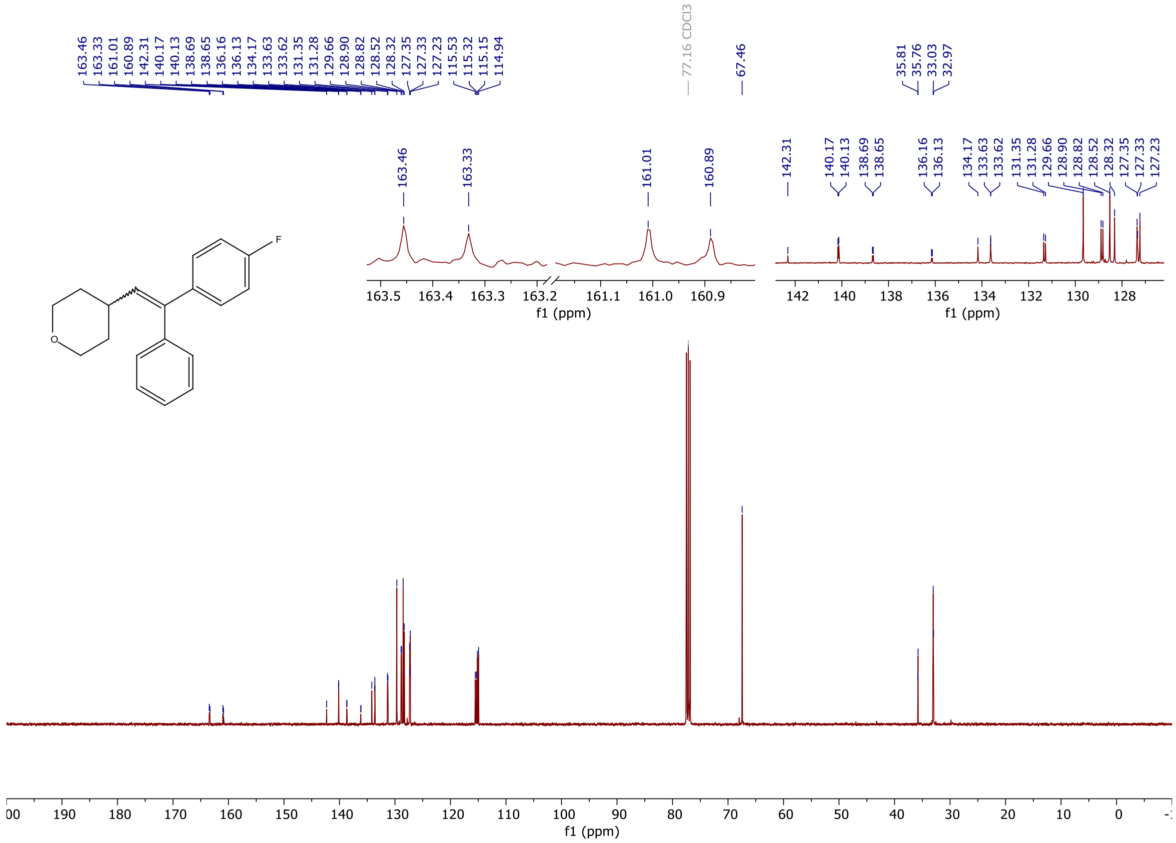

^{19}F NMR (376 MHz, $CDCl_3$) of **64**

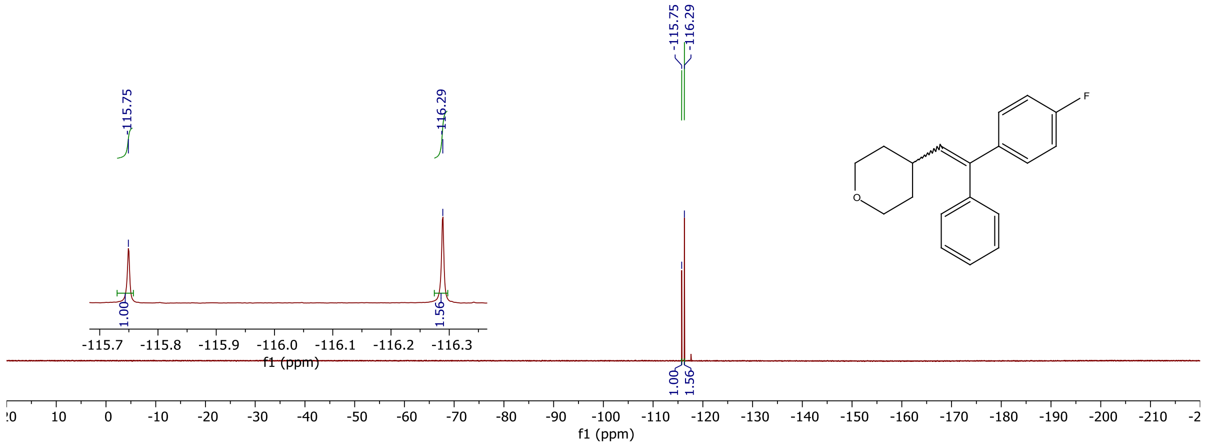

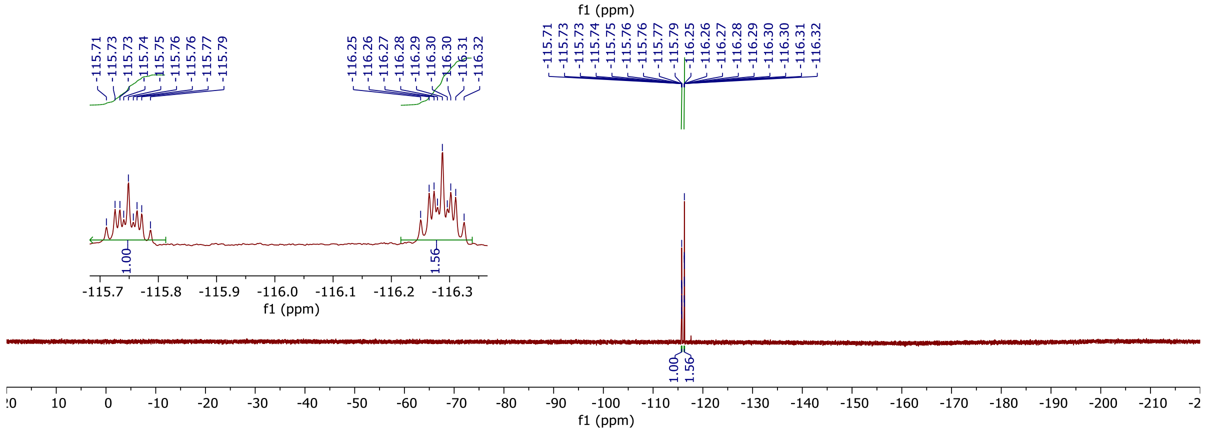

^{1}H NMR (400 MHz, $CDCl_3$) of **65**

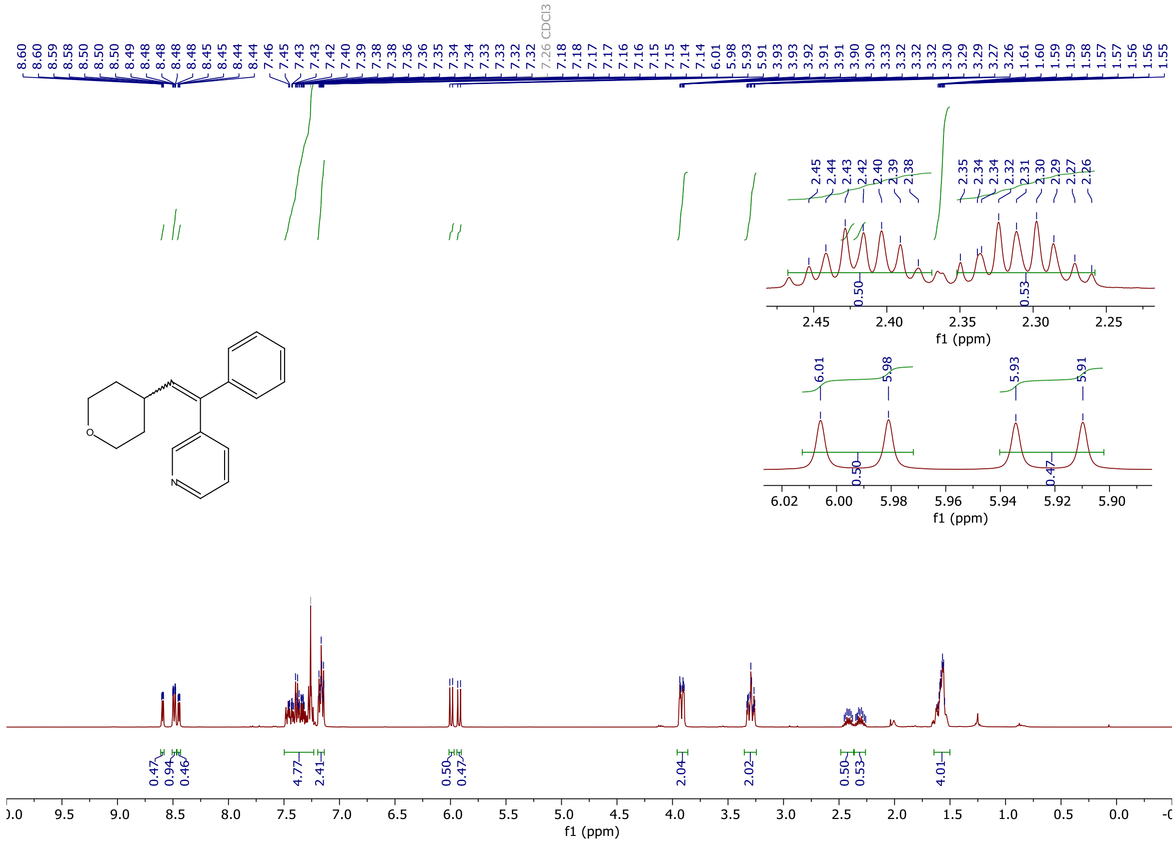

^{13}C NMR (101 MHz, $CDCl_3$) of **65**

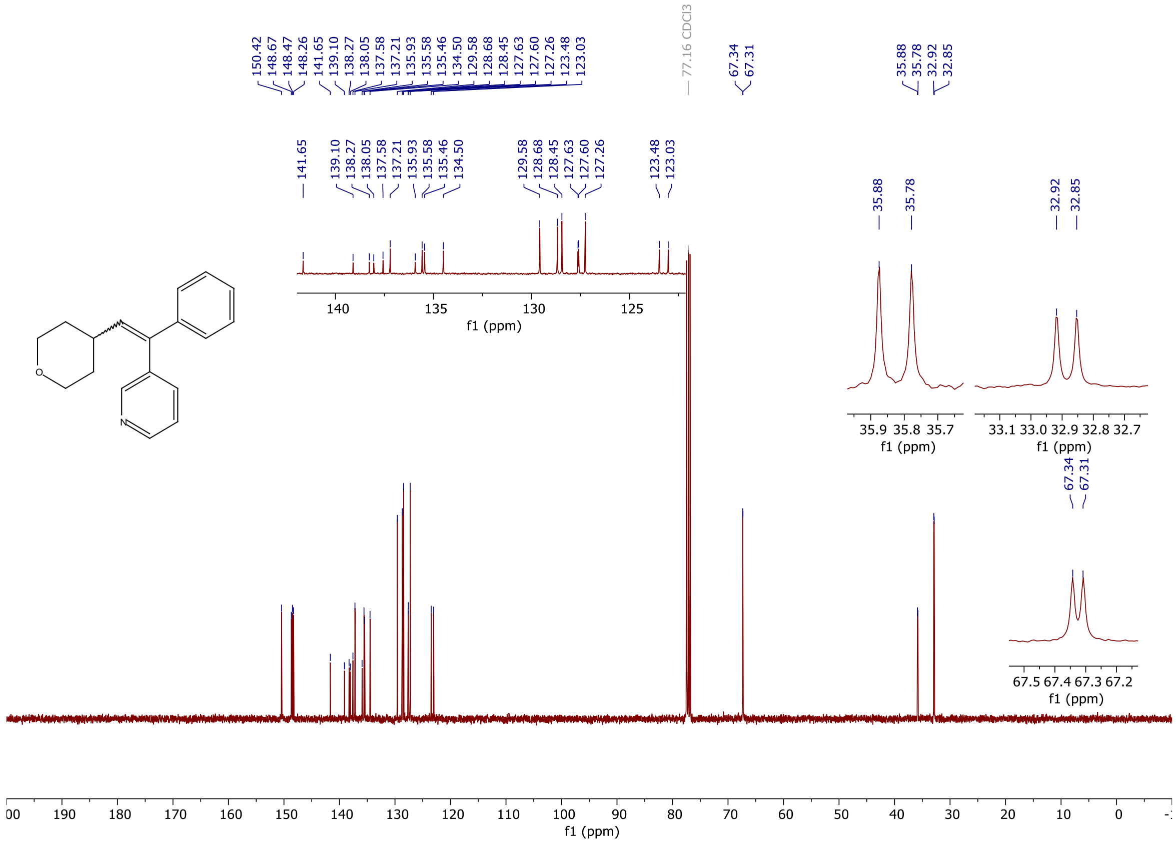

^{1}H NMR (400 MHz, $CDCl_3$) of **66**

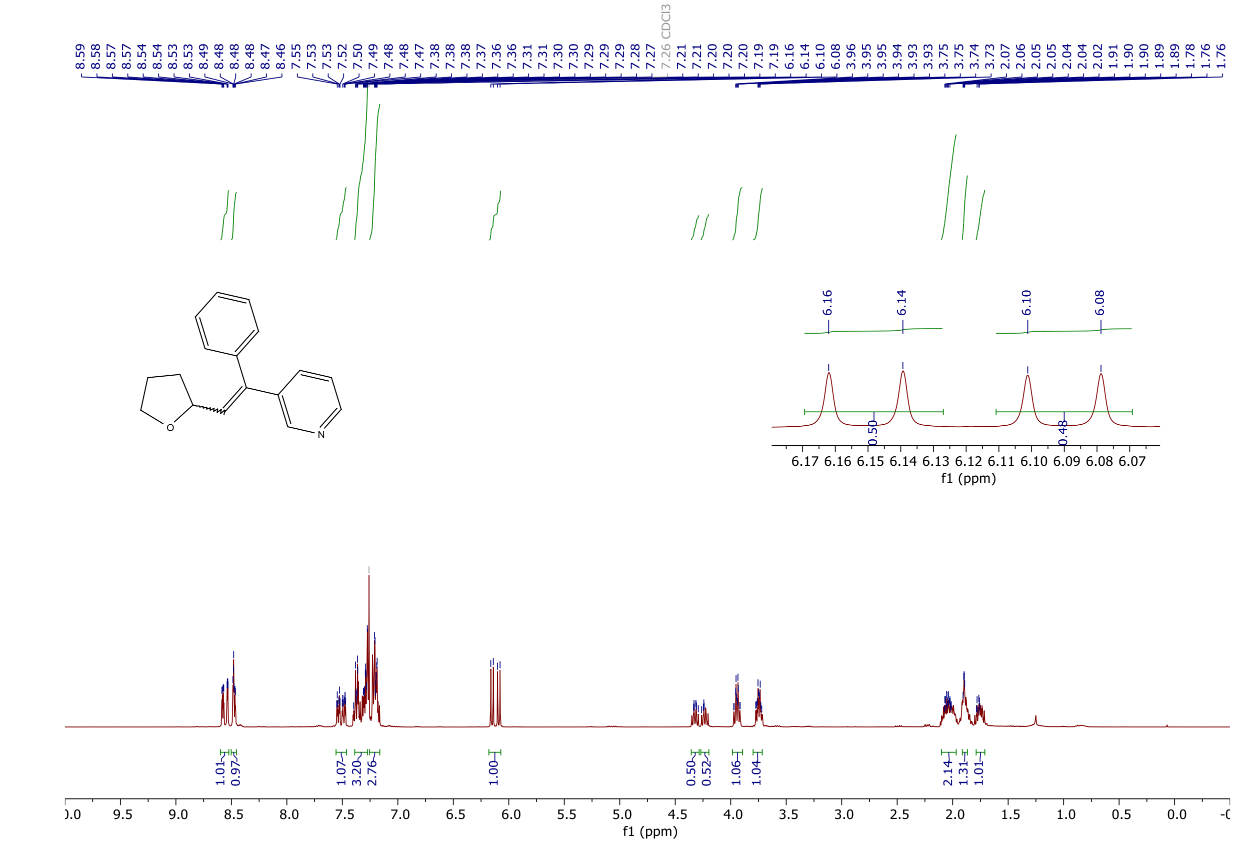

^{13}C NMR (101 MHz, $CDCl_3$) of **66**

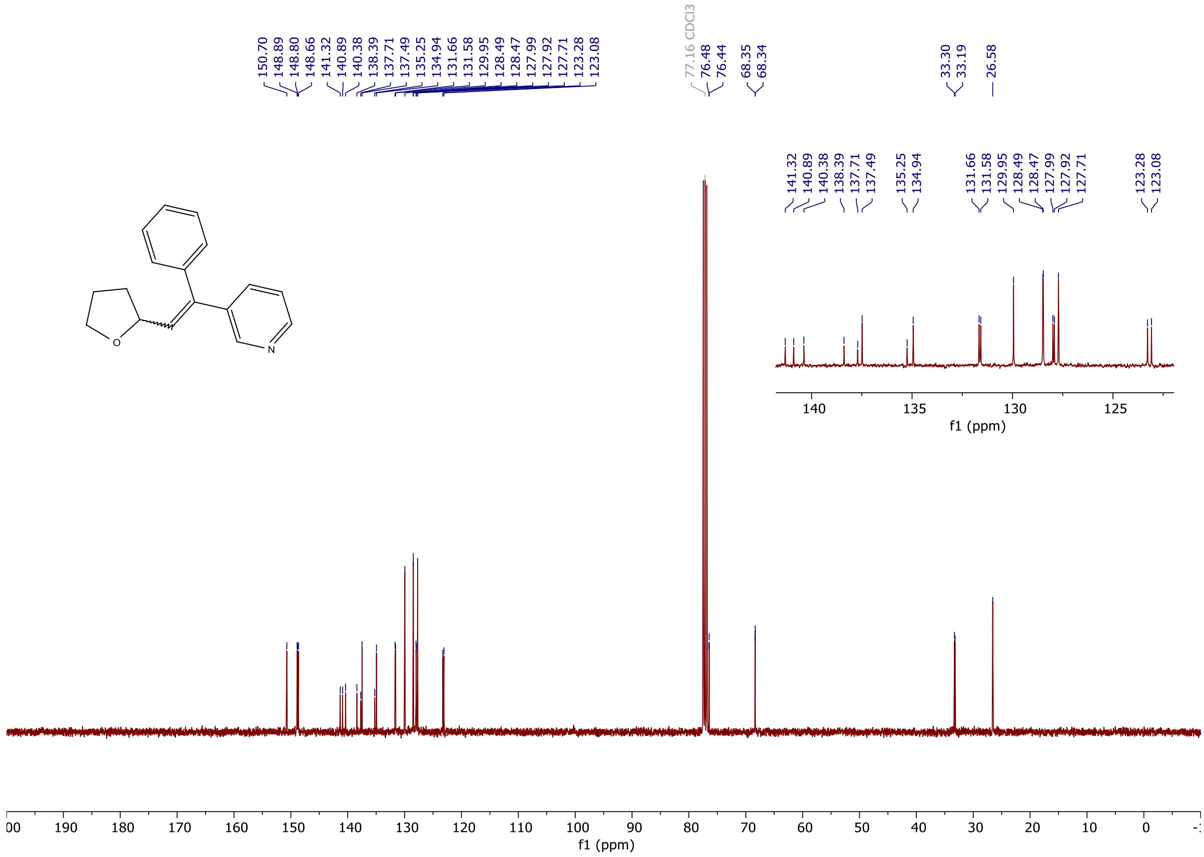

^{1}H NMR (400 MHz, $CDCl_3$) of **67**

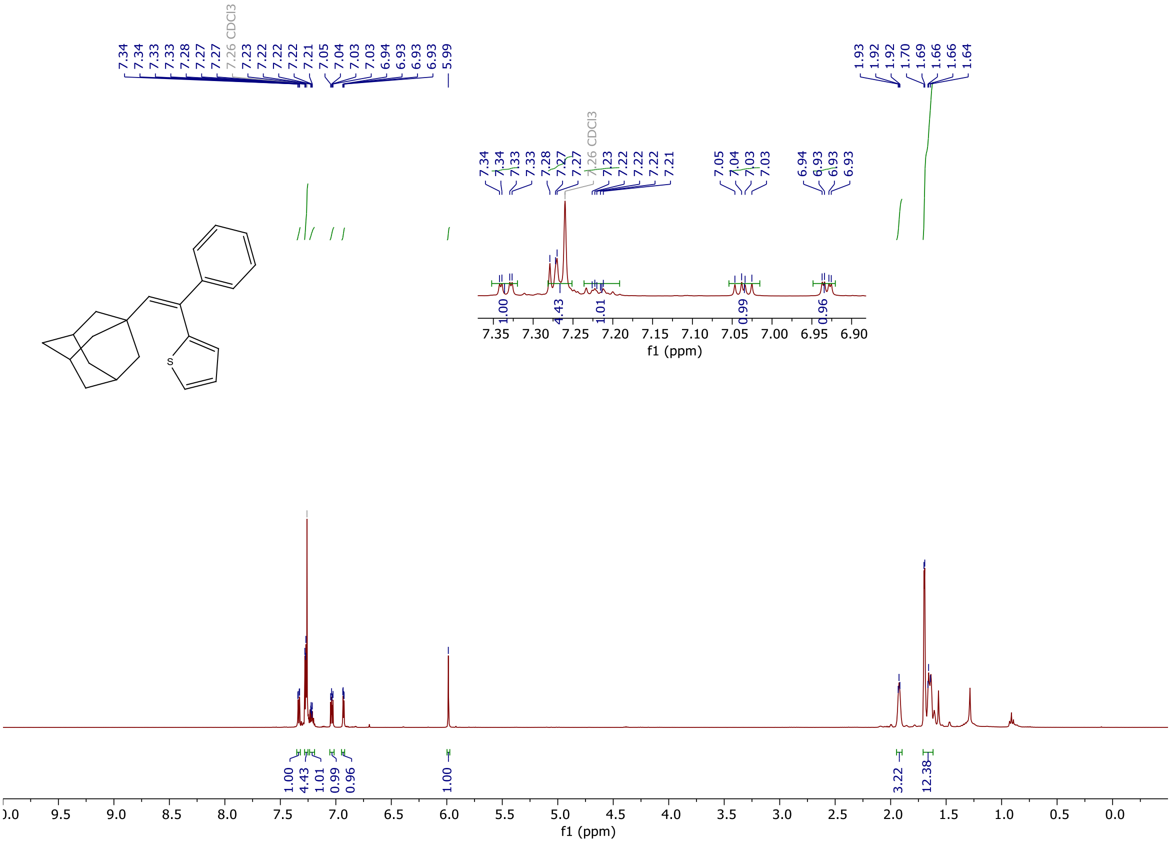

^{13}C NMR (101 MHz, $CDCl_3$) of **67**

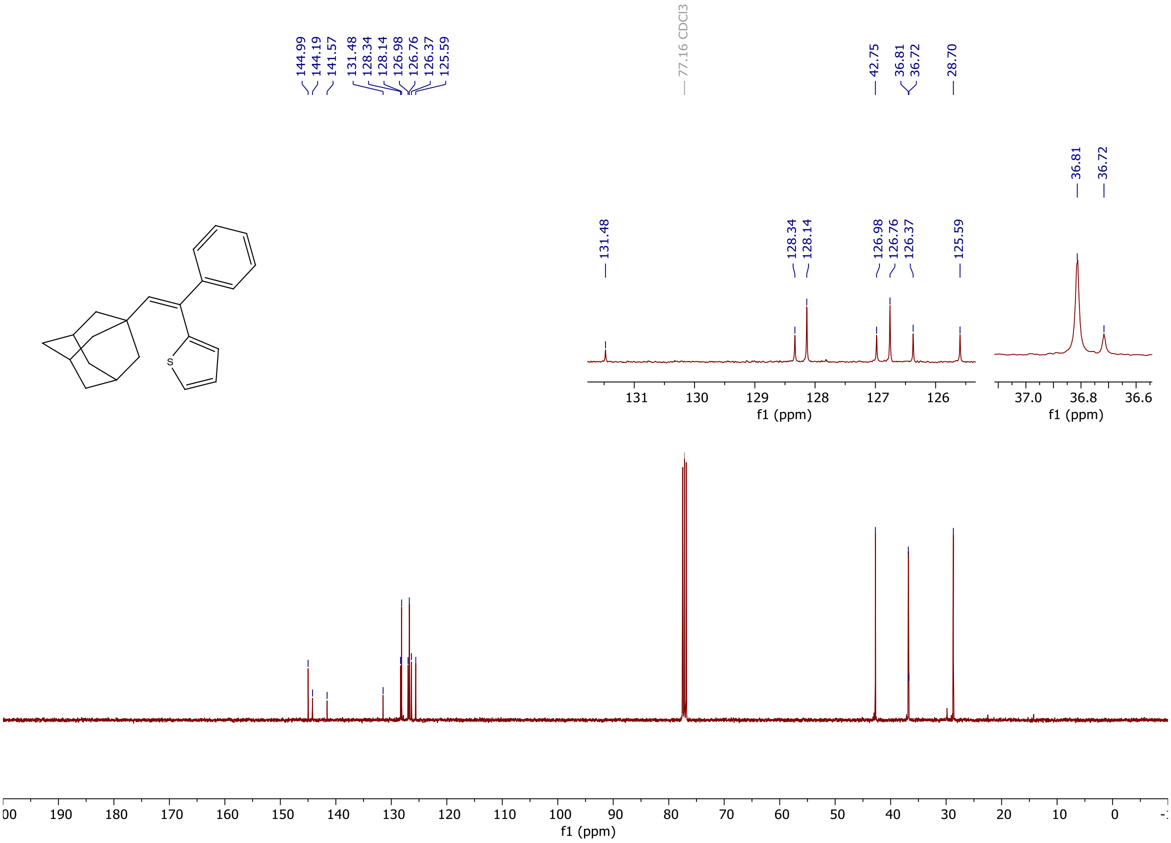

^{1}H NMR (400 MHz, $CDCl_3$) of **68**

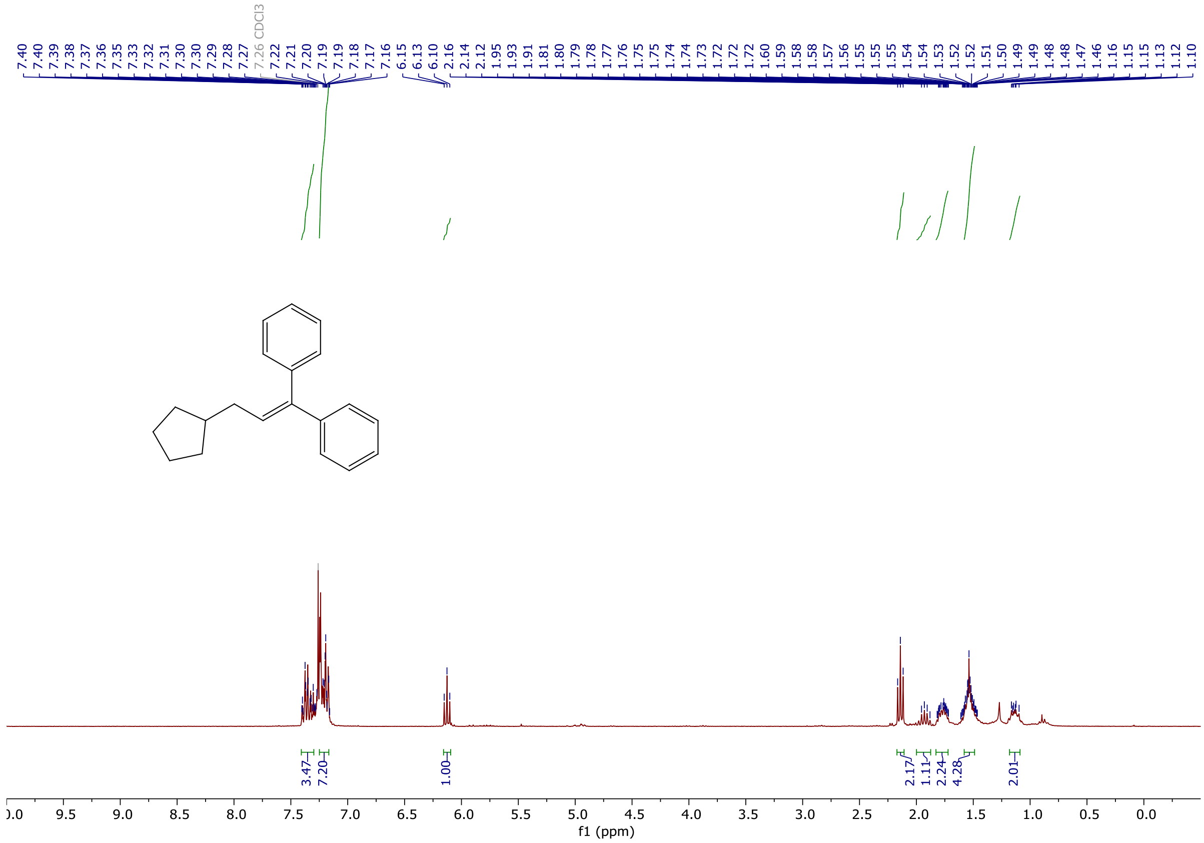

^{13}C NMR (101 MHz, $CDCl_3$) of **68**

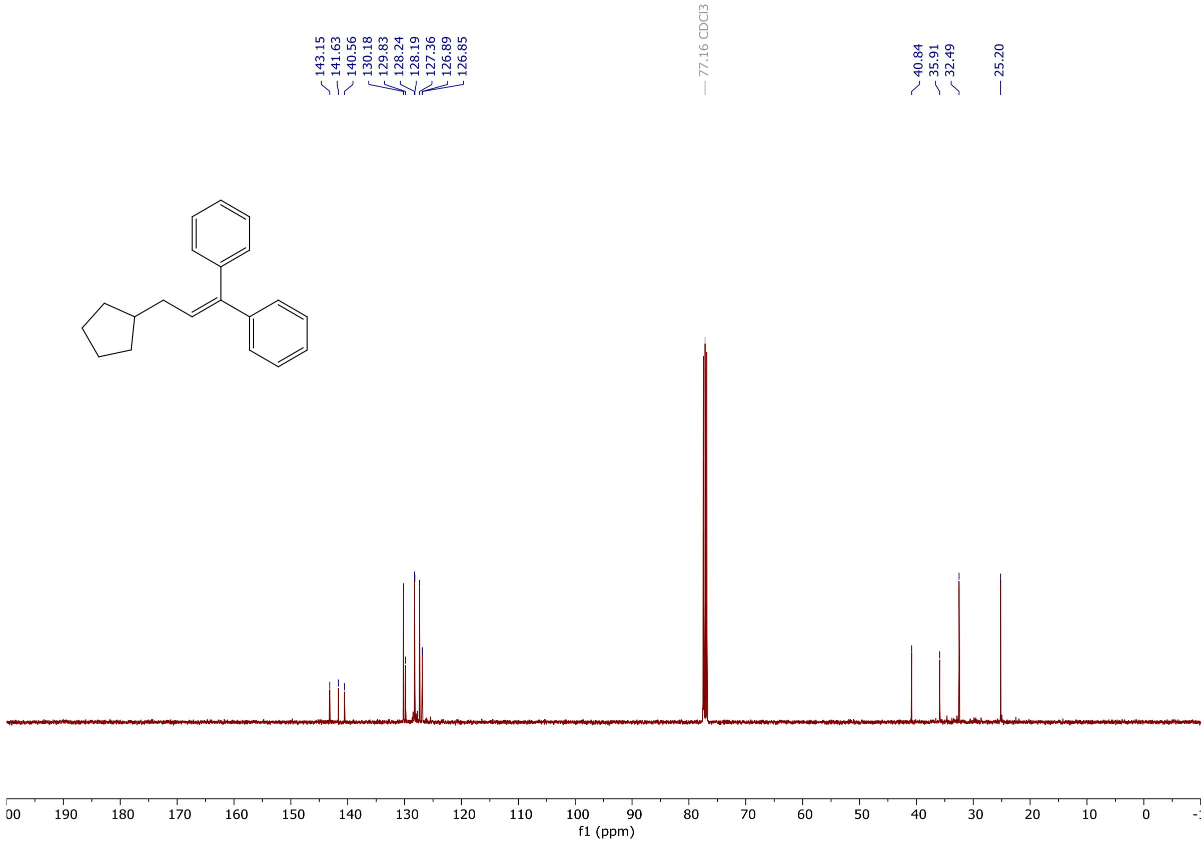

^{1}H NMR (400 MHz, $CDCl_3$) of **69**

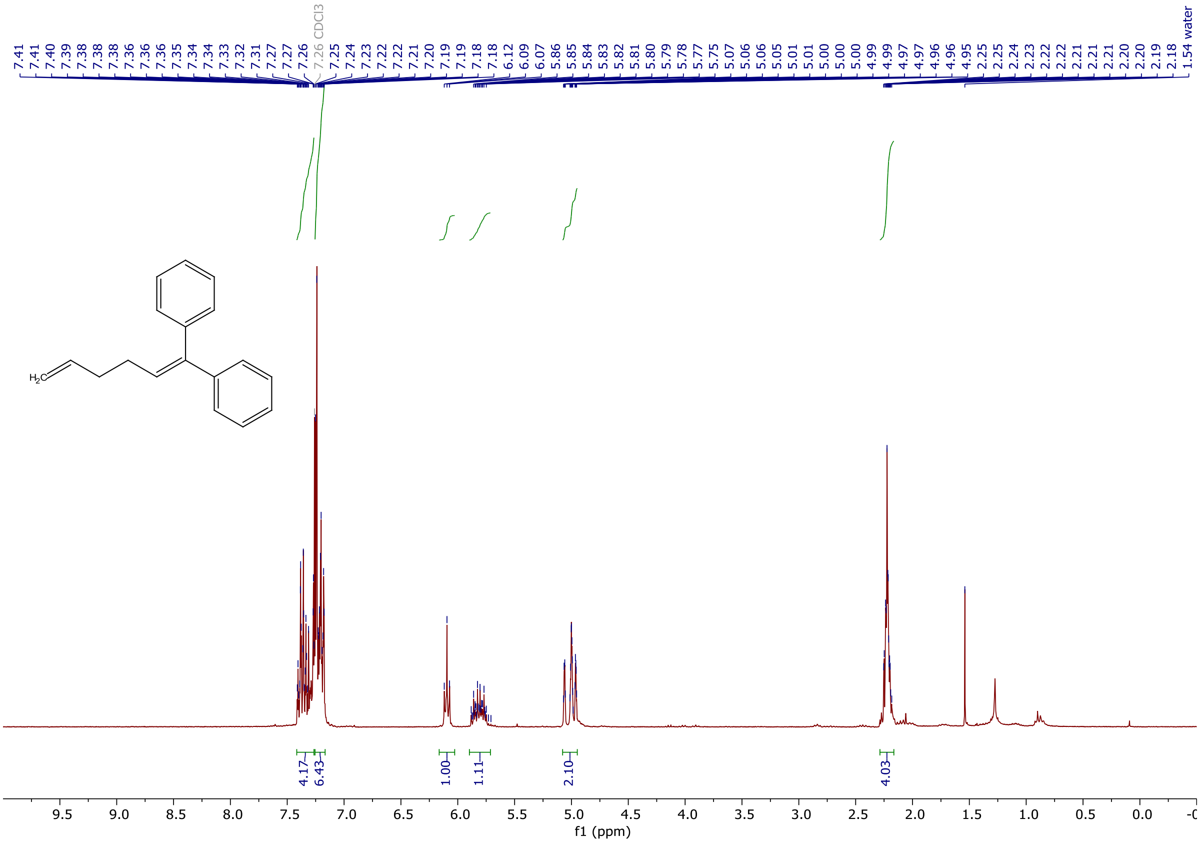

^{13}C NMR (101 MHz, $CDCl_3$) of **69**

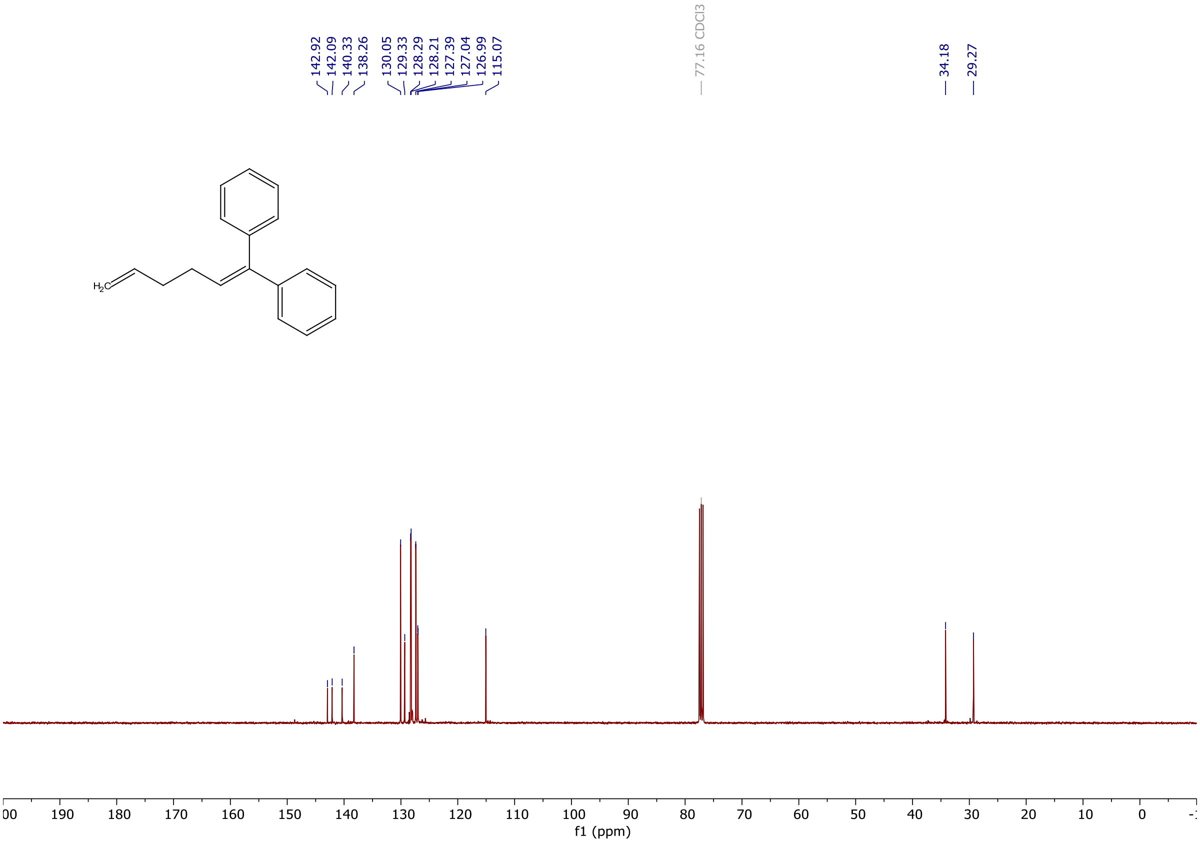

Supplement: Supplementary file 1 — Supporting Information [file CHEM-31-e202500666-s003.pdf]
